# Supplementary material for: Stakeholders’ perspectives on clinical trial acceptability and approach to consent within a limited timeframe: a mixed methods study
Source: BMJ Open. 2024 Jan 2;14(1):e077023. doi: 10.1136/bmjopen-2023-077023 (PMC10773389; doi:10.1136/bmjopen-2023-077023)
Supplement: Supplementary data [file bmjopen-2023-077023supp001.pdf]

BMJ Publishing Group Limited (BMJ) disclaims all liability and responsibility arising from any reliance placed on this supplemental material which has been supplied by the author(s)

BESS
Bronchiolitis Endotracheal Surfactant Study

The efficacy and mechanism of surfactant therapy for critically ill infants with bronchiolitis: The **B**ronchiolitis **E**ndotracheal **S**urfactant **S**tudy (BESS)

A phase-2 blinded randomised air-placebo-controlled trial of endotracheal surfactant (poractant alfa) in critically ill infants with bronchiolitis

BESS Protocol v2.0 28/09/2018

Study Sponsor(s):
University of Liverpool
Research Support Office
2nd Floor Block D Waterhouse
3 Brownlow Street
Liverpool
L69 3GA

EudraCT number: 2018-001169-18
ISRCTN :
Research Ethics Ref: 18/SC/0427
Sponsor Ref: UoL001360
IRAS Ref: 220853

UNIVERSITY OF LIVERPOOL
CTRC Clinical Trials Research Centre
NHS National Institute for Health Research

Deja E, *et al. BMJ Open* 2024; 14:e077023. doi: 10.1136/bmjopen-2023-077023

PROTOCOL APPROVAL

I, the undersigned, hereby approve this clinical study protocol:

Authorised by Chief Investigator:

Signature: ______________________ **Date:** 28092018

Prof. Malcolm (Calum) Gracie Semple, PhD, BM BCh (Oxon), FRCPCH, FRCPE, FHEA
Consultant in Paediatric Respiratory Medicine, Alder Hey Children's NHS Foundation Trust
Professor of Child Health and Outbreak Medicine, University of Liverpool
Institute in The Park,
Alder Hey Children's Hospital
Eaton Road
Liverpool
L12 2AP

Authorised on behalf of Sponsor:

Signature: ______________________ **Date:** ____________

Mr Alex Astor
Head of Research Support
Research Support Office
2nd Floor Block D Waterhouse Building
3 Brownlow Street,
Liverpool
L69 3GL

Authorised on behalf of the Lead Statistician:

Signature: ______________________ **Date:** ____________

Dr Ashley Jones, PhD
Senior Statistician
Clinical Trials Research Centre
Institute of Child Health
Alder Hey Children's Hospital
Eaton Road
Liverpool
L12 2AP

PROTOCOL APPROVAL

I, the undersigned, hereby approve this clinical study protocol:
Authorised by Chief Investigator:

Signature: ______________________ **Date:** __________

Prof. Malcolm (Calum) Gracie Semple, PhD, BM BCh (Oxon), FRCPCH, FRCPE, FHEA
Consultant in Paediatric Respiratory Medicine, Alder Hey Children's NHS Foundation Trust
Professor of Child Health and Outbreak Medicine, University of Liverpool
Institute in The Park,
Alder Hey Children's Hospital
Eaton Road
Liverpool
L12 2AP

Authorised on behalf of Sponsor:

Signature: ______________________ **Date:** 19/10/18

Mr Alex Astor

Head of Research Support
Research Support Office
2nd Floor Block D Waterhouse Building
3 Brownlow Street,
Liverpool
L69 3GL

Authorised on behalf of the Lead Statistician:

Signature: ______________________ **Date:** 28/9/18.

Dr Ashley Jones, PhD
Senior Statistician
Clinical Trials Research Centre
Institute of Child Health
Alder Hey Children's Hospital
Eaton Road
Liverpool
L12 2AP

General Information

This document describes the BESS trial including detailed information about procedures and recruitment. The protocol should not be used as an aide-memoir or guide for the treatment of other patients; every care was taken in its drafting, but corrections or amendments may be necessary. These will be circulated to the registered investigators in the trial, but centres entering patients for the first time are advised to contact the coordinating centre (Clinical Trials Research Centre (CTRC), University of Liverpool) to confirm they have the most up to date version. Clinical problems relating to this trial should be referred to the relevant Chief Investigator, Professor Calum Semple, via the CTRC.

This protocol defines the participant characteristics required for study entry and the schedule of treatment and follow-up. Participant recruitment will be undertaken in compliance with this document, applicable regulatory and governance requirements and waivers to authorise non-compliance are not permitted.

Incidents of protocol non-compliance, whether reported prospectively (e.g. where a treatment cannot be administered on a scheduled date as a result of public holidays) or retrospectively noted (e.g. as a result of central monitoring) are recorded as protocol deviations, the incidence of which are monitored and reported to trial oversight committees.

The template content structure is consistent with the SPIRIT (Standard Protocol Item: Recommendations for Interventional Trials 2013) and has regard for the Health Research Authority guidance. Regulatory and ethical compliance information is located in section 12.

Relationship Statements

Roles and responsibilities are described in section 15

The University of Liverpool is the Sponsoring organisation. The University of Liverpool will formally delegate specific sponsoring roles to the Chief Investigator and Clinical Trials Unit, but remains legally responsible for the trial.

Clinical Trials Unit (CTU): The CTRC at the University of Liverpool in collaboration with the Chief Investigator, Prof Calum Semple, will have overall management responsibility for the trial from a CTU perspective and will be responsible for the co-ordination of centres.

CTRC as part of the Liverpool Trials Collaborative has achieved full registration by the UK Clinical Research Collaboration (www.ukcrc.org) as their standards and systems were assessed by an international review panel as reaching the highest quality. The CTRC has a diverse trial portfolio underpinned by methodological rigour, a Good Clinical Practice (GCP) compliant data management system, and core standard operating procedures (SOPs).

**Sponsor:**	**Trial Management, Monitoring and Analysis:**
University of Liverpool<br>Liverpool<br>L69 3BX<br>Tel: 0151 794 8290<br>Email: sponsor@liverpool.ac.uk	Clinical Trials Research Centre, University of Liverpool<br>Institute of Child Health<br>Alder Hey Children's Hospital NHS Foundation Trust<br>Liverpool<br>L12 2AP<br>Tel: 0151 795 8757<br>Fax: 0151 795 8770<br>Email: bess@liverpool.ac.uk
**Mechanistic & Microbiology Work:**	**Paediatric Intensive Care Audit Data:**
University of Southampton<br>University Road<br>Southampton<br>SO17 1BJ<br>Tel: 023 8059 5000<br>Email: ris@soton.ac.uk	PICANet<br>School of Medicine<br>8.49 Worsley Building<br>University of Leeds<br>Leeds<br>LS2 9JT<br>PICANet<br>0113 343 8125<br>picanet@leeds.ac.uk

Deja E, *et al. BMJ Open* 2024; 14:e077023. doi: 10.1136/bmjopen-2023-077023

Contact Details: Individuals

**Individual Authorised to Sign the Protocol and Protocol Amendments on behalf of the Sponsor:**	**Chief Investigator (CI):**
Mr Alex Astor<br>Research Support Office<br>2nd Floor, Block D, Waterhouse Building, 3 Brownlow Street, Liverpool, L69 3GL<br>0151 794 8739<br>sponsor@liv.ac.uk	Prof. MG (Calum) Semple,<br>Women's and Children's Health<br>University of Liverpool<br>Institute in the Park, Alder Hey Children's Hospital, Eaton Road, Liverpool, L12 2AP, UK<br>0151 252 5250<br>m.g.semple@liverpool.ac.uk
**Medical Expert who will Advise on Protocol Related Clinical Queries (If other than CI):**	**Medical Expert who will Evaluate SAE Reports (if other than CI):**
Prof. Paul S McNamara<br>Women's and Children's Health<br>University of Liverpool<br>Institute in the Park, Alder Hey Children's Hospital, Eaton Road, Liverpool, L12 2AP, UK<br>0151 252 5250<br>mcnamp@liverpool.ac.uk	Prof. Paul S McNamara<br>Women's and Children's Health<br>University of Liverpool<br>Institute in the Park, Alder Hey Children's Hospital, Eaton Road, Liverpool, L12 2AP, UK<br>0151 252 5250<br>mcnamp@liverpool.ac.uk
**Medical Expert who will Evaluate SAE Reports (If other than CI or cross cover):**	
Dr Kent Thorburn<br>Paediatric Intensive Care Unit, Alder Hey Children's Hospital, Eaton Road, Liverpool, L12 2AP, UK<br>0151 228 4811 ext. 4180<br>kent.thorburn@alderhey.nhs.uk	

Supplemental material
BMJ Publishing Group Limited (BMJ) disclaims all liability and responsibility arising from any reliance placed on this supplemental material which has been supplied by the author(s)
BMJ Open

Additional Contacts:

The contact details of other individuals involved in the trial are detailed in documents supplementary to the protocol and stored in the Trial Master File:

Contact	Document Title
Trial Management Group (TMG)<br>Trial Steering Committee (TSC)<br>Independent Data and Safety Monitoring Committee (IDSMC)	BESS Trial Oversight Committee Membership
Principal Investigators	BESS Participating Centres

Deja E, *et al. BMJ Open* 2024; 14:e077023. doi: 10.1136/bmjopen-2023-077023

1 TABLE OF CONTENTS

Protocol Approval 2
1 Table of Contents 7
Glossary 12
2 Protocol Summary 14
3 Introduction 17
3.1 Study Overview 17
3.2 Background 17
3.3 Rationale 18
3.4 Risk and Benefits 19
3.4.1 Potential Benefits 19
3.4.2 Potential Risks 19
3.5 Aims & Objectives of Work Package A 19
3.5.1 Aims 19
3.5.2 Primary Objective 20
3.5.3 Secondary Objective(s) 20
3.6 Outcome measures/endpoints 20
3.6.1 Primary Outcome/Endpoint 20
3.6.2 Secondary Outcomes/Endpoints 20
4 TRIAL DESIGN 24
4.1 Trial Overview 24
Schematic of Trial Design: 25
4.2 Use of a standardised weaning and extubation protocol 26
5 Trial Setting and Selection of SITES & Clinicians 27
5.1 Selection of Sites & Clinicians 27
6 Study Population 28
6.1 Inclusion Criteria 28
6.1.1 Definition of bronchiolitis 28
6.2 Exclusion Criteria 28
6.3 Co-enrolment Guidelines 29
7 Recruitment and Randomisation 30
7.1 Participant Identification and Screening 30
7.2 Informed Consent 30

Deja E, *et al. BMJ Open* 2024; 14:e077023. doi: 10.1136/bmjopen-2023-077023

7.2.1 Prospective Informed Consent Process 30
7.2.2 Consent for additional work 32
7.3 Randomisation Procedure 32
7.3.1 Randomisation System Failure 33
7.4 Blinding to Allocations 33
8 Participant Time Line, Assessments and Procedures 34
8.1 Administration of Intervention 34
8.1.1 Immediately prior to administration 34
8.1.2 Administration of allocated intervention 35
8.1.3 Post administration of allocated intervention 35
8.1.4 Weaning and extubation by protocol 35
8.2 Participant follow-up 36
8.3 Schedule for Intervention & Follow-up 37
8.4 Procedures for assessing Efficacy 39
8.4.1 Efficacy Assessment 1 39
8.4.2 Efficacy Assessment 2 39
8.5 Procedures for Assessing Safety 40
8.5.1 Procedure for Limited Physical Examination in BESS 40
8.6 Other Assessments 40
8.6.1 Parent Reported Quality of Life questionnaires 40
8.6.2 Special Assays or Procedures 41
8.7 Patient Transfer and Withdrawal 42
8.7.1 Patient Transfers 42
8.7.2 Withdrawal from the Trial 42
8.8 Loss to Follow-up 43
8.9 Trial Closure 43
9 Trial Treatment/interventions 44
9.1 Introduction 44
9.2 Arm A – Endotracheal Poractant Alfa at 200mg/kg (first dose), with up to two further doses at 100mg/kg 44
9.2.1 Formulation, Packaging, Labelling, Storage and Stability 44
9.2.2 Preparation, Dosage and Administration of Study Treatment/s 45
9.2.3 Specific Restrictions 46
9.2.4 Overdose 46
9.2.5 Temperature Compliance 46

Deja E, *et al. BMJ Open* 2024; 14:e077023. doi: 10.1136/bmjopen-2023-077023

9.2.6 Accountability Procedures for Trial Treatments....47
9.2.7 Assessment of Compliance with Study Treatment/s....48
9.3 Arm B – Placebo....48
9.4 Unblinding....48
9.4.1 Emergency Unblinding....49
9.4.2 Accidental unblinding....49
9.4.3 Unblinding at Trial Closure....49
9.5 Concomitant Medications....50
10 Safety Reporting....51
10.1 Terms and Definitions....51
10.2 Notes on Adverse Event Inclusions and Exclusions....52
10.2.1 Include:....52
10.2.2 Do Not Include:....52
10.2.3 Notification of deaths....53
10.3 Notes on Severity / Grading of Adverse Events....53
10.4 Relationship to Trial Treatment....54
10.5 Expectedness....54
10.6 Follow-up After Adverse Events....55
10.7 Time Period for Safety Reporting....55
10.8 Reporting Procedures....55
10.8.1 Flowchart for Reporting Requirements of Adverse Events....56
10.8.2 Non serious events (ARs/AEs)....58
10.8.3 Serious events (AEs/ARs/SUSARs)....58
10.9 Safety Reporting Responsibilities – Investigator....58
10.9.1 Maintenance of Blinding....59
10.10 Safety Reporting Responsibilities – CTRC....60
10.11 Safety reports....61
10.12 Urgent Safety Measures....61
10.13 Contact Details and Out-of-hours Medical Cover....61
11 Statistical Considerations....62
11.1 Introduction....62
11.2 Method of Randomisation....62
11.3 Sample Size calculation....62
11.4 Interim Monitoring and Analyses....62

Deja E, *et al. BMJ Open* 2024; 14:e077023. doi: 10.1136/bmjopen-2023-077023

11.5 Analysis Plan 63

12 exploratory & translational work; work packages b and c.... 64

12.1 Work Package B: Study of Parent & Staff Experiences....64

12.1.1 Parent and staff perspectives sub study analysis 65

12.2 Work Package C:....65

12.2.1 Metabolic profiling of surfactant composition and kinetics 66

12.2.2 Recovery of surfactant proteins A & D (SP-A & SP-D) is modulated by exogenous surfactant. 67

12.2.3 Lower airway inflammation is modulated by exogenous surfactant 67

12.2.4 Treatment efficacy or failure is associated with detection of specific pathogens. 67

12.2.5 Mechanistic Protocol 68

13 Regulatory and Ethical Approvals 70

13.1 Statement of Compliance 70

13.2 Regulatory Approval 70

13.3 Ethical Considerations 71

13.4 HRA Approval....71

13.5 Ethical and Local Governance Approval 71

13.6 Protocol Deviation and Serious Breaches....72

13.7 Study Discontinuation 72

14 Data Management and Trial Monitoring.... 73

14.1 Source Documents 73

14.2 Data Capture Methods....73

14.3 Monitoring....74

14.3.1 Risk Assessment 74

14.3.2 Central Monitoring....74

14.3.3 Clinical Site Monitoring....75

14.4 Confidentiality 75

14.5 Quality Assurance (QA) and Quality Control (QC) 75

14.6 Records Retention 76

15 Indemnity.... 77

16 Roles and Responsibilities 78

16.1 Role of Study Sponsor and Study Funder....78

16.2 Funding and Support in Kind 78

16.3 Protocol Contributors....79

16.4 Trial Committees 80

16.4.1 Trial Management Group (TMG) ... 80
16.4.2 Trial Steering Committee (TSC) ... 80
16.4.3 Independent Data and Safety Monitoring Committee (IDSMC) ... 80
17 Publication and Dissemination ... 81
17.1 Publication Policy ... 81
17.1.1 Authorship ... 81
17.2 Dissemination to Key Stakeholders ... 81
17.1 Data Sharing ... 82
18 Chronology of Protocol Amendments ... 83
18.1 Version 1.0 (18/07/2018) ... 83
18.2 Version 2.0 (28/09/2018) ... 83
19 References ... 84
20 Documents Supplementary to the protocol ... 87

Glossary

AE	Adverse Event
AR	Adverse Reaction
BAL	Bronchoalveolar lavage
CI	Chief Investigator
CRF	Case Report Form
CTIMP	Clinical Trials of an Investigational Medicinal Product
CTRC	Clinical Trials Research Centre
CTU	Clinical Trials Unit
EUDRACT	European Clinical Trials Database
DSUR	Development Safety Update Report
FiO_2	Fraction of inspired oxygen
GP	General Practitioner
HRA	Health Research Authority
IB	Investigator's Brochure
IDSMC	Independent Data and Safety and Monitoring Committee
IMP	Investigational Medicinal Product
ISF	Investigator Site File
LRSQ	Liverpool Respiratory Symptom Questionnaire
MHRA	Medicines and Health Care Products Regulatory Agency
MV	Mechanical Ventilation
NIMP	Non Investigational Medicinal Product
NRES	National Research Ethics Service
NIHR CRN	National Institute for Health Research Clinical Research Network
OI	Oxygenation Index
OSI	Oxygen Saturation Index
PEEP	Positive End Expiratory Pressure
PI	Principal Investigator
PICANet	Paediatric Intensive Care Audit Network
PICU	Paediatric intensive care unit
PIP	Peak Inspiratory Pressure
PROM	Parent Reported Outcome Measure
QA	Quality Assurance
QC	Quality Control

R&D	Research & Development
REC	Research Ethics Committee
RN	Research Nurse (Registered)
RSI	Reference Safety Information
RSO	Research Support Office
SAE	Serious Adverse Event
SAR	Serious Adverse Reaction
SDV	Source Data Verification
SMPC	Summary of Product Characteristics
SOP	Standard Operating Procedure
SpO_2	Peripheral oxygen saturation
SSAR	Suspected Serious Adverse Reaction
SUSAR	Suspected Unexpected Serious Adverse Reaction
TMG	Trial Management Group
TMP	Trial Monitoring Plan
TSC	Trial Steering Committee
UAR	Unexpected Adverse Reaction
VI	Ventilation Index
WP-A	Work package A “The Trial”
WP-B	Work package B “Exploration of parental experience”
WP-C	Work package C “Mechanistic sub-studies”

2 PROTOCOL SUMMARY

**Full Title**	**The efficacy and mechanism of surfactant therapy for critically ill infants with bronchiolitis: The Bronchiolitis Endotracheal Surfactant Study (BESS). A phase-2 blinded randomised air-placebo-controlled trial of endotracheal surfactant (poractant alfa) in critically ill infants with bronchiolitis**
**Acronym**	**BESS**
**Phase**	2
**Target Condition**	Bronchiolitis of infancy requiring conventional invasive Mechanical Ventilation (MV)<br>Diagnosis of bronchiolitis per clinical criteria defined in national guidance NICE-NG9.
**Sample size**	284
**Main Inclusion Criteria**	1. Term-born infants < 26 weeks old and preterm-born infants < 26 weeks corrected age<br>2. Diagnosis of bronchiolitis<br>3. Requires conventional invasive MV via tracheal intubation<br>4. Parent or person with parental responsibility has given written informed consent for trial participation
**Main Exclusion Criteria**	1. Major congenital anomalies<br>2. Congenital neuromuscular disease<br>3. Already intubated for MV for >48 hours or likely to have been intubated for MV for >48 hours by randomisation<br>4. Have received or are receiving extracorporeal membrane oxygenation (ECMO) or oscillation during this episode of bronchiolitis<br>5. Have received or are receiving intratracheal administration of any surfactant during this episode of bronchiolitis<br>6. Receiving MV for primary apnoea rather than respiratory failure<br>7. A known hypersensitivity to the active substance or excipients of poractant alpha (Curosurf®)<br>8. A decision to wean to extubation has already been made<br>9. Clinical judgement of futility
**Study Centres and Distribution**	Approximately 14 Paediatric Intensive Care Units (PICUs) in UK tertiary hospitals.
**Patient Study Duration**	Duration of treatment: 24 hours (a maximum of three 12hourly intratracheal doses of surfactant; first dose (0hrs), second dose at 12hrs and third at 24hrs).

	Duration of follow-up: up to a maximum of 12 months, minimum 90 days.		
**Overall Study duration**	44 months		
**Agent/ Intervention**	**Intervention:** Endotracheal poractant alfa (Curosurf®, porcine lung phospholipid fraction supplied at 80mg/mL), first dose 200mg/kg, repeated 12 hourly at 100mg/kg, to a maximum of 3 doses according to the manufacturer's summary of product characteristics (SPC). Unlicensed use (UK licence, new indication).<br>**Control:** An identical series of up to three procedures (while intubated) using air as the placebo.		

		Objectives	Outcome Measures
**Work Package A**	**Primary**	To determine the efficacy of endotracheal surfactant in reducing total duration of MV by 18 hours or more, by random allocation of infants to receive either up to 3 doses of poractant alfa or up to 3 doses of air-placebo in an identical administration procedure (the Trial = Work package A)	The Primary Outcome measure for the Trial will be total duration of MV (hours) from randomisation to final extubation. We will include time off MV due to failed extubation.<br>Secondary Outcome measures relating to the Primary Objective of the trial are listed in the Protocol.
	**Secondary**	To describe the impact of intervention on infants' long-term respiratory symptoms, using a parent reported outcome measure	The Liverpool Respiratory Symptom Questionnaire applied at 6m and 1yr after intervention in patients recruited in seasons 1 and 2.
**Work Package B**	**Exploratory/ Translational:**<br>**Study of Staff & Parent Experiences**	In a subgroup of patients, to explore staff and parent experiences of trial recruitment, consent and conduct in season 1 to inform season 2.	Outcome Measures relating to Exploratory and Translational aspects of the study are listed where known in section 12. Due to the exploratory nature of this work, other outcome measures are likely to be identified over this course of the study and after the trial has completed.
**Work Package C**	**Exploratory/ Translational:**<br>**Mechanistic Study**	In a subgroup of patients to explore the mechanisms for treatment efficacy and failures at a patient level by describing associations between trial outcome with markers of infection, inflammation, and surfactant composition in patient Bronchoalveolar Lavage Fluid.	

NB: The Exploratory and Translational parts of the study (Work Packages B & C) will not inform the primary or secondary outcomes of the main trial. They are only included as an annexe to the

Trial Protocol for regulatory purposes related to the Research Ethic Committee, Health Research Authority and Local Institutional Research permissions.

3 INTRODUCTION

3.1 Study Overview

The study "The efficacy and mechanism of surfactant therapy for critically ill infants with bronchiolitis" will have the short title "Bronchiolitis Endotracheal Surfactant Study" and the acronym BESS.

The award supporting BESS supports three work packages. These are: the trial (Work package A; WP-A); a study of staff and parental experience (Work package B; WP-B) and mechanistic exploratory sub-studies (Work package C; WP-C).

Work package A is subject to Medicines for Human Use (Clinical Trials) Regulations 2004 (as amended) and requires regulatory permission from the Medicines and Healthcare products Regulatory Agency (MHRA). Work packages B and C are dependent upon trial activity but do not provide outcome measures for the trial and so are not subject to these regulations. The study, which includes all work packages, requires REC, HRA and NHS permissions for which this unified protocol document is required. The detail of Work Packages B and C are described in Section 12.

3.2 Background

Bronchiolitis of infancy is a seasonal viral disease of the lower airways that most commonly causes feeding difficulties and respiratory distress and in severe cases causes a significant public health burden due to hospitalisation and mortality(1). It is the single most common reason for hospital admission of infants (children age<1yr). The youngest of these and those born prematurely are most often and most severely affected (1).

A recent study of Hospital Episode Statistics for England found 25,000 cases of bronchiolitis from each annual birth cohort (40.4 per 1000 (95% CI 38.5 to 42.3)) were admitted to hospital in their first year of life (2). Using PICANet data, the same study found just over 1000 of these infants were admitted with critical illness to English paediatric intensive care units (PICUs) each year (95% CI 1.3 to 1.6 per 1000 infants/yr) for mechanical ventilation (MV). This accounted for 12% of all admissions to PICUs. The infants' modal age was 1 month; the mean duration of PICU admission was 6.1 days (95% CI 5.4 to 6.7 days), and their case-fatality rate was 1.75%.

Despite advances in the provision of non-invasive modes of respiratory support, admissions to PICU and duration of MV for life-threatening bronchiolitis have remained static for the past 4 years (2). Excluding infants with rare congenital co-morbidities (mostly cardiac & neuromuscular anomalies), infants requiring MV for bronchiolitis do so for a mean of 4.8 days (SD 2.5d) (PICANet data, 2011-14).

Existing Research

In 2015 Jat & Chawla (3) found on pooled analysis of three small studies a significant reduction in length of PICU stay between endotracheal surfactant treated and control groups (mean difference (MD) -1.81d (95%CI -2.42 to -1.19) of critically ill infants with bronchiolitis who required MV. There were sustained improvements of arterial oxygenation (PaO_2) and carbon dioxide ($PaCO_2$) elimination (4-6). No adverse effects or complications were observed in the three studies. The authors of this systematic review and meta-analysis concluded that use of surfactant had favourable effects but that larger trials with adequate power were required to evaluate the effectiveness of surfactant therapy in critically ill infants with bronchiolitis who required MV.

3.3 Rationale

There is no vaccine or specific treatment for bronchiolitis. Care is supportive as described in National Guidance (17). Breathing fails in very severe cases, and these babies need intensive care with breathing being supported by a machine called a ventilator, hence known as mechanical ventilation (MV).
Pulmonary surfactant is secreted by lung alveolar cells to reduce surface tension so increasing compliance of the lungs, allowing them to inflate more easily and reduce the work of breathing (8). Surfactant proteins A and D have innate antiviral immune properties as collectins (9).
Studies of infants with life-threatening bronchiolitis show reduced lung compliance and surfactant deficiency (10-14). These features are similar to the respiratory distress syndrome of infants born prematurely. Premature infants are routinely treated with animal-derived surfactant to improve lung compliance, and reduce mortality, morbidity and duration of MV (15).
Most infants with severe bronchiolitis are very young and/or born prematurely. They present at modal age 1 month, with 62.2% age <2months (2). We hypothesise that the effect of exogenous surfactant on infants with severe bronchiolitis may be similar to the therapeutic effect on those born prematurely as both conditions present with surfactant deficiency. At present surfactant is used without evidence as rescue therapy in some infants and young children with the most severe respiratory failure who are difficult to manage even with MV.
The systematic reviews by Jat & Chawla found that surfactant therapy in infants with critical illness due to bronchiolitis was: safe, improved gas exchange and reduced both duration of MV and length of stay on PICU (3, 16).
There are no other relevant trials registered on Clinicaltrials.gov, EU Clinical Trial Register or ISRCTN [search “surfactant” AND “bronchiolitis”, 6-JUL-2018].
This topic has been identified by the National Institute for Health Research (NIHR) Maternal Neonatal and Child Health panel in 2013 and selected by the Prioritisation Group for commissioning by both the Health Technology Assessment (HTA) and Efficacy and Mechanism Evaluation (EME), and has the support of the NIHR Translational Research Partnership (TRP) for Inflammatory Respiratory Diseases and UK Paediatric Intensive Care Society Study Group (PICS-SG).

Research Question

Does surfactant therapy reduce duration of invasive mechanical ventilation when given to infants early in the course of critical illness due to bronchiolitis?

Intervention

The intervention being trialled is:
Endotracheal poractant alfa (Curosurf®, porcine lung phospholipid fraction supplied at 80mg/mL), first dose 200mg/kg, repeated 12 hourly at 100mg/kg, up to a total of 3 doses according to the manufacturer's summary of product characteristics (SPC).
Or-
An identical series of up to three procedures (while intubated) using air as the placebo.

Hypothesis

The hypothesis we will test in the trial is “Endotracheal surfactant reduces duration of mechanical ventilation by 18 hours

3.4 Risk and Benefits

3.4.1 Potential Benefits

The potential benefit of the therapy to the participants is a sustained improvement in gas exchange leading to a reduction of total duration of MV. The potential benefit to the participant is a reduction of time in a technology-dependent vulnerable state, reduced risk of MV associated morbidity, reduced exposure to sedation and muscle paralysing drugs and a more rapid return to usual parental care. This in turn would reduce parental anxiety. Also, freeing up PICU beds during winter surge offers savings and opportunity to use resources for other conditions.

3.4.2 Potential Risks

Administration of poractant alfa (porcine derived surfactant, Curosurf®) in infants with bronchiolitis is well tolerated and considered safe(3). Transient minor adverse reactions associated with administration of poractant alfa for the treatment of neonatal Respiratory Distress Syndrome (RDS) include bradycardia, hypotension, endotracheal tube blockage, and oxygen desaturation. The risk of such events is minimised by tracheal toilet prior to administration of poractant alfa, as recommended in the Summary of Product Characteristics.

Small volume (2x1mL/kg) non-bronchoscopic bronchoalveolar lavage (BAL) procedure on PICU is safe and well tolerated procedure(7). In a published audit of 27 children on PICU who underwent multiple small volume BAL installations (5x1mL/kg) as part of a single routine diagnostic procedure, 7 experienced transient oxygen desaturation requiring temporary increased FiO_2. We will limit BAL sampling to a 2x1mL/kg instillation at each scheduled assessment.

Small volume (1.25mL (range 1.0 to 1.3mL)) blood sampling will be done on infants ventilated at Liverpool and Southampton PICUs and scheduled to coincide with routine blood sampling. Existing vascular access will be used when available. There is no risk of anaemia associated with this limited volume of blood sampling. The sample will be taken from an arterial line if present. If not by standard venesection.

Capillary blood gas sampling (100microliters) or arterial blood gas sampling for subject monitoring will use data from routine blood gas sampling when either is collected within a 2-hour window of a research-scheduled sample. As this is part of standard care no additional risks are expected.

3.5 Aims & Objectives of Work Package A

3.5.1 Aims

1. To conduct a multicentre blinded randomised placebo-controlled phase-2 trial of the endotracheal surfactant poractant alfa (CUROSURF®) as therapy for bronchiolitis in critically ill infants.
   Hypothesis to test: Endotracheal surfactant reduces duration of mechanical ventilation by 18 hours (Work package A (WP-A)).
2. To evaluate the safety profile in the two treatment groups

3.5.2 Primary Objective

To determine the efficacy of endotracheal surfactant in reducing total duration of MV by 18 hours, by random allocation of infants to receive up to 3 doses of poractant alfa or up to 3 doses of air-placebo in an identical administration procedure.

3.5.3 Secondary Objective(s)

1. To describe the impact of intervention on infants' long-term respiratory symptoms
2. To describe secondary outcomes of efficacy including physiological indices and duration of other modes of respiratory support.
3. To assess the safety of the intervention for infants with life-threatening bronchiolitis

3.6 Outcome measures/endpoints

3.6.1 Primary Outcome/Endpoint

The primary outcome measure will be total duration of MV (hours) from randomisation to final extubation. We will include time off MV due to failed extubation.
Justification: This outcome measure is clinically important, was suggested in both HTA and EME commissioning calls and is valued by our Parent Advisory Group. Reduced duration of MV reduces ventilator-associated morbidity, sedation, muscle relaxants, healthcare costs and bed occupancy at a time of increased seasonal pressure.

3.6.2 Secondary Outcomes/Endpoints

The secondary outcome measures for the trial (WP-A) are:
For efficacy:

1. duration of post-extubation non-invasive respiratory support (all forms of non-invasive ventilation: e.g. nasal CPAP and nasal high low oxygen)
2. duration of oxygen supplementation
3. number of trial interventions given (both poractant and placebo)
4. use of steroids to assist extubation
5. duration of stay on PICU and in hospital;
6. change over time from baseline of Ventilation Index (VI), Oxygenation Index (OI) and Oxygenation Saturation Index (OSI) while mechanically ventilated, & SpO_2/FiO_2 (SF) ratio.[1]
7. the score (value) of parent reported outcome measure of respiratory symptoms the "Liverpool Respiratory Symptom Questionnaire" (LRSQ) at 6m (+/-1m) and 12m (+/-1m) (20);

[1] VI and OI are validated physiological measurements reflecting respiratory function (gas exchange) normalised for level of respiratory support provided by MV, but require blood sampling(21). OSI is a validated physiological measurement reflecting respiratory function (gas exchange) normalised for level of respiratory support provided by MV that does not require blood sampling (41). The SF ratio is a non-invasive measure of respiratory function and can be calculated in situations where invasive blood gas monitoring and MV has ceased (22).

8. time from randomisation to meeting study criteria for readiness for Spontaneous Breathing Test

For safety:

1. failure to administer the intervention due to any adverse event during preparatory processes (tracheal toilet or BAL)
2. failure to complete administration of the intervention due to any adverse event during administration of the intervention (regardless of which arm of allocation)
3. incidents of ‘air leak’ (pneumothorax and pneumomediastinum) occurring before discharge from PICU
4. other adverse events and serious adverse events associated with the intervention
5. any need to replace the endotracheal tube
6. parent reported readmission to hospital (all causes) up to 90 days post randomisation
7. death during PICU admission
8. all-cause mortality at 90 days post randomisation

Table 1: Objectives and Outcome Measures

Objectives	Outcome Measures	Timepoint(s) of evaluation of this outcome measure (if applicable)
**Primary Objective**<br>To determine the efficacy of endotracheal surfactant in reducing total duration of MV by 18 hours or more, by random blind allocation of infants to receive either up to 3 doses of poractant alfa or up to 3 doses of air-placebo in an identical administration procedure.	Total duration of MV (hours) from randomisation to final extubation, including any time off MV due to failed extubation.	N/A
**Secondary Objective 1**<br>To describe the impact of intervention on infants' long term respiratory symptoms	The score (value) of patient reported outcome measure of respiratory systems in the "Liverpool Respiratory Symptom Questionnaire" (LRSQ)	LRSQ at 6 months and 12 months post randomisation
**Secondary Objective 2**<br>To describe secondary outcomes of efficacy including physiological indices and duration of other modes of respiratory support	Time from randomisation to meeting study criteria for readiness for Spontaneous Breathing Test	N/A
	Number of trial interventions given	N/A
	Change over time from baseline of Ventilation Index (VI), Oxygenation Index (OI) and Oxygenation Saturation Index (OSI) while mechanically ventilated, & SpO_2/FiO_2 (SF) ratio	Capillary blood gas sampling immediately prior to intervention and then at 6, 12, 24, 36 and 48hrs while still on MV; to continue daily after 48hrs if still on MV.
	Duration of oxygen supplementation	N/A
	Use of steroids to assist extubation	N/A

Deja E, et al. BMJ Open 2024; 14:e077023. doi: 10.1136/bmjopen-2023-077023

	Duration of post-extubation non-invasive respiratory support	N/A
	Duration of stay on PICU and in hospital	N/A
**Secondary Objective 3** To assess the safety of the intervention for infants with life-threatening bronchiolitis	Failure to administer the intervention due to any adverse event during preparatory processes (tracheal toilet or BAL)	Randomisation to 24 hours after final trial intervention
	Failure to complete administration of the intervention due to any adverse event during administration of the intervention (regardless of which arm of allocation)	Randomisation to 24 hours after final trial intervention
	Incidents of 'air leak' (pneumothorax and pneumomediastinum) occurring before discharge from PICU	Randomisation to discharge from PICU
	Other adverse events and serious adverse events associated with the intervention	Randomisation to 24 hours after final trial intervention
	Any need to replace the endotracheal tube	Randomisation to successful extubation
	Parent reported readmission to hospital (all causes) up to 90 days post randomisation	PICU discharge to 90 days post-randomisation
	Death during PICU admission	Randomisation to PICU discharge
	All-cause mortality at 90 days post randomisation	PICU discharge to 90 days post-randomisation

Deja E, *et al. BMJ Open* 2024; 14:e077023. doi: 10.1136/bmjopen-2023-077023

4 TRIAL DESIGN

4.1 Trial Overview

The BESS trial is a phase-2 blinded randomised air-placebo-controlled trial of endotracheal surfactant (poractant alfa) in critically ill infants with bronchiolitis (we will refer to this as Work Package A, or WP-A).

Schematic of Trial Design:

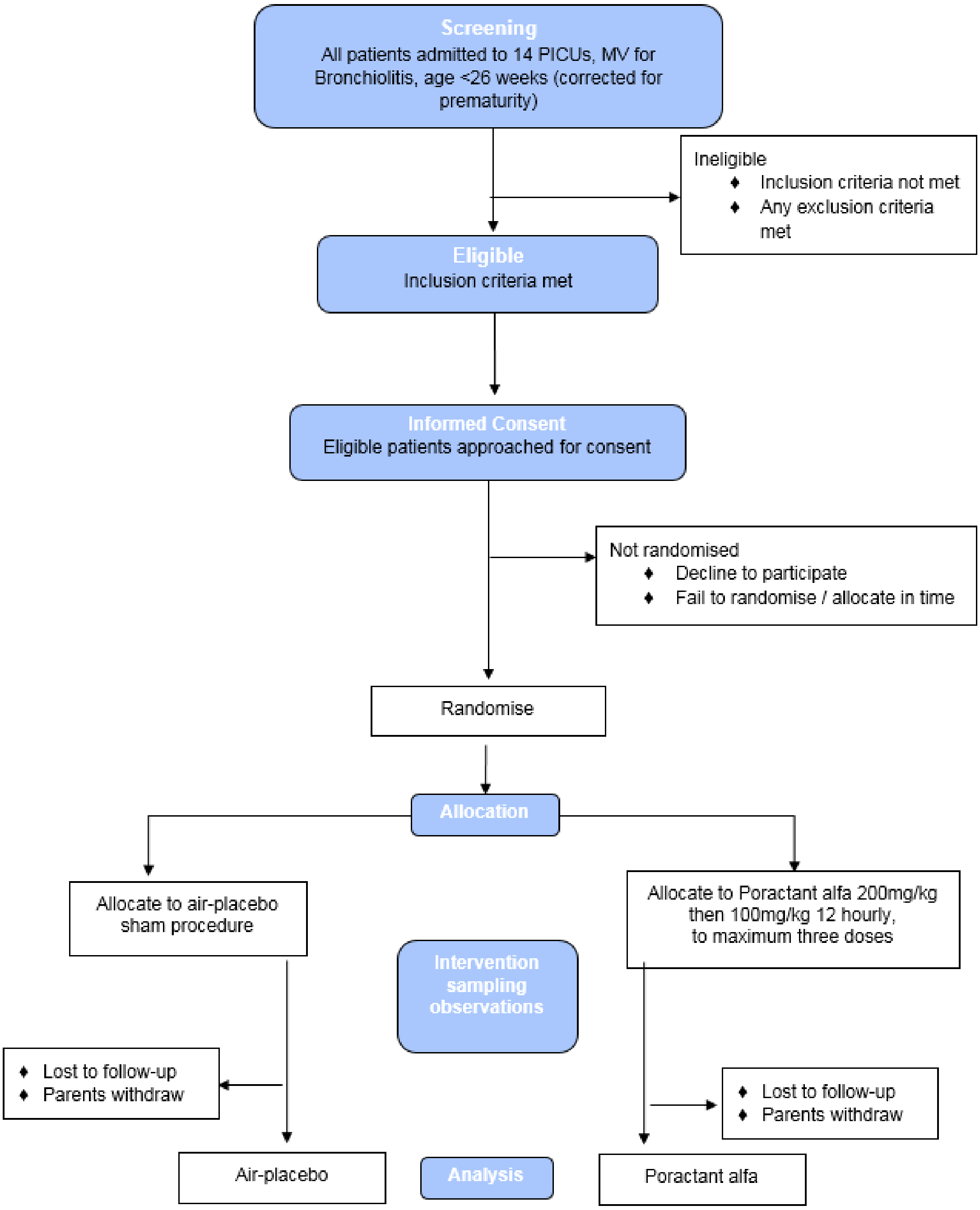

Figure 1: Schematic of Trial Design

4.2 Use of a standardised weaning and extubation protocol

On the direction of the NIHR EME board, the BESS trial protocol will include a standardised protocol for weaning from mechanical ventilation and extubation.
Weaning and extubation by protocol will standardise this aspect of care across sites and in both arms of BESS. Infants in BESS will be extubated after they meet specified physiological criteria that indicate the infant is ready for a Spontaneous Breathing Test (SBT) and pass that test, (see section 8.1.2.1 for further details).

5 TRIAL SETTING AND SELECTION OF SITES & CLINICIANS

Approximately fourteen Paediatric Intensive Care Units (PICUs) in the United Kingdom that routinely mechanically ventilate critically ill infants with high caseloads of bronchiolitis will recruit to BESS. PIs for sites that have provisionally agreed to participate in BESS have shared local activity data, commented on the application and are recognised as co-applicants on the application award. The PIs will champion the study at the sites and take local responsibility for the conduct of the trial. PIs will identify local co-Investigators.

5.1 Selection of Sites & Clinicians

Criteria for the selection of centres includes

- NHS Hospitals in the UK providing paediatric intensive care
- Historic high case load of bronchiolitis, as reported to PICANet
- Sufficient research capacity comprised of staff, time and facilities to undertake the trial; including patient screening and recruitment, randomisation, collection and provision to the CTRC of all required data, collection of research samples, identification and management of adverse events including notification to CTRC within protocol defined timeframes, identification and provision of information to the CTRC of all protocol breaches.
- All staff contributing to the trial must have valid certified Good Clinical Practice (GCP) training throughout the conduct of the trial. Requirements of the Sponsor are that renewal of GCP must occur every 3 years.
- Willingness to participate

Final decisions on site selection will be determined by the Trial Management Group (TMG) and will be described in the supplementary document ‘BESS Site Suitability Assessment.
Initiation of centres will be undertaken in compliance with CTRC SOPs[2]. Centres fulfilling the criteria will be selected to be recruitment centres for the BESS trial and will be opened to recruitment upon successful completion of all global (e.g. MREC and MHRA) and study-specific conditions (e.g. site personnel training requirements) and once all necessary documents have been returned to CTRC as detailed in the trial Green Light checklist.

Participating centres will be listed in the ‘BESS Participating Centres’ log, maintained separately to the protocol and stored in the Trial Master File (TMF).

[2] CTRC SOP TM017 Study Initiation in CTRC and TM018 Study Initiation at Sites

Deja E, et al. BMJ Open 2024; 14:e077023. doi: 10.1136/bmjopen-2023-077023

6 STUDY POPULATION

6.1 Inclusion Criteria

1. Term-born infants < 26 weeks old and preterm-born infants < 26 weeks corrected age†
2. Diagnosis of bronchiolitis (see below)
3. Requires conventional invasive MV via tracheal intubation
4. Parent or person with parental responsibility has given written informed consent for trial participation

Note: Infants with chronic lung disease of prematurity can be included in the trial.
† Correcting for prematurity is a well-established method for adjusting assessment of infants born prematurely who are anatomically, physiologically, immunologically and neuro-developmentally immature for their chronological age. In our study, term-born infants will be eligible for inclusion in the study up to chronological age of 26 weeks old (i.e. all those under 6 months old). Premature born infants have their age corrected to account for weeks of lost gestation, hence an infant born 12 weeks premature (i.e. before completing the 28th week of the 40 weeks of pregnancy) would still be eligible up to a chronological age of 38 weeks old (i.e. under 9 months), because that infant is still less than 26 weeks old when age is corrected for the premature birth.

6.1.1 Definition of bronchiolitis

Diagnosis of bronchiolitis will be made using clinical criteria defined in national guidance NICE-NG9 (17).

6.2 Exclusion Criteria

1. Major congenital anomalies
2. Congenital neuromuscular disease
3. Already intubated for MV for >48 hours or likely to have been intubated for MV for >48 hours by randomisation
4. Have received or are receiving extracorporeal membrane oxygenation (ECMO) or oscillation during this episode of bronchiolitis
5. Have received or are receiving intratracheal administration of any surfactant during this episode of bronchiolitis
6. Receiving MV for primary apnoea rather than respiratory failure
7. A known hypersensitivity to the active substance or excipients of poractant alpha (Curosurf®)
8. A decision to wean to extubation has already been made
9. Clinical judgement of futility

6.3 Co-enrolment Guidelines

The investigators are happy to enter in to co-enrolment agreements where feasible.
Where other trials are taking place at a site participating in BESS and the target population overlaps, sites should make the BESS team aware at the earliest opportunity so that the potential for co-enrolment can be discussed between the two trial teams. The BESS investigators are open to co-enrolment with other studies but this must be agreed between both Chief Investigators prior to randomisation into BESS.

7 RECRUITMENT AND RANDOMISATION

7.1 Participant Identification and Screening

All patients admitted to recruiting PICUs, receiving conventional MV for bronchiolitis, age <26 weeks (corrected for prematurity) will be screened for eligibility before trial entry. Screening and confirmation of full eligibility will be completed to allow randomisation within 48 hours of intubation.

A screening log of patients who are assessed for eligibility but not randomised will be maintained and returned to the CTRC on a regular basis, as this will provide important information for monitoring purposes. The screening log will capture which patients have been screened for the trial but assessed as ineligible, and those considered eligible and approached for consent but for whom consent was not obtained; reasons (all per subject) for non-inclusion will be recorded. Reasons for declining to participate will be asked routinely but it will be made clear that parents/persons with parental responsibility do not have to provide a reason unless happy to do so.

The screening log will not contain patient identifiers as those who are not eligible will not have provided their consent for identifiers to be captured as part of the trial. Therefore, sites will maintain their own trial register linking patients screening and/or randomisation numbers to their identifiers. Under no circumstances will this log be returned to the CTRC.
Patients will be reviewed for eligibility once they have been intubated for MV, or as soon as their arrival into PICU if they are already intubated. Parents/persons with parental responsibility for patients who meet the eligibility criteria and are suitable for inclusion (see 6.1 and 6.2) will be provided with information about the trial (both verbal and written) to consider participation.

Only a medically qualified doctor authorised on the site Delegation Log can confirm full eligibility of any patient. A record of this confirmation must be made in the patient’s medical notes on the date of confirmation of eligibility.

7.2 Informed Consent

7.2.1 Prospective Informed Consent Process

Parents and those persons with parental responsibility may give consent for the patient to take part in BESS; hereafter throughout this protocol they shall be referred to as ‘parent(s)’.
Initiation of the intervention in BESS is urgent and the condition “critical illness due to bronchiolitis requiring MV” is a medical emergency. Randomisation must be completed within 48 hours of intubation for MV, which will limit the time available for a parent to consider whether or not their child should participate. Site research teams will use their understanding of the clinical situation for each individual patient to judge when is most appropriate to approach parents about trial participation.
The trial concept will be introduced to a parent by familiar members of the usual clinical care team, be they nurses or physicians, as per the Canadian Critical Care Trials PICU Research Group trial recruitment model (24).
If a parent is interested, a member of the usual clinical care team will make an introduction to the research staff who will provide written study information and offer further verbal information.

Due to the time-critical nature of the intervention, the parent will have a limited timeframe to ask questions and make a decision about their child's participation in the trial. Randomisation must take place within 48 hours of intubation for MV and while some parents may have this full timeframe to consider trial participation, others may have considerably less due to time of arrival on PICU, time of approach etc. Consent will be sought by any appropriately trained and delegated medical staff and where local site policy allows, research nurses or respiratory physiotherapists (the research team).

Consent from the parent must be obtained prior to every infant participating in the trial. This consent will be sought after a full explanation has been given of the treatment options; including the conventional and generally accepted methods of treatment, the objectives, risks and inconveniences of the trial, and the conditions under which it is to be conducted.

Parent Information Sheet and Consent (PISC) forms, describing in detail the trial intervention, procedures and risks will be approved by a research ethics committee (REC). The parent will be asked to read and review this document. Upon reviewing the document, the person seeking consent will explain the research study to the parent. This information, and the supporting conversation, will emphasise that participation in the trial is voluntary and that the parent may withdraw their infant from the trial at any time and for any reason.

The parent will be given opportunity to ask any questions that may arise and time to consider the information, within the constraints of the 48 hour period between intubation and randomisation, prior to agreeing to participate. A contact point where further information about the trial may be obtained will be provided on the parent information sheet.

The parent will then sign and date the consent form if they agree to their infant participating. Both the person obtaining consent and the parent must sign and date the consent form; the researcher and parent must sign at the same time, with the researcher completing their signature **after** the parent.

A copy of the signed PISC will be given to the parent for their record. The original copy will be filed in the Investigator Site File, a copy filed in the participant's medical notes and a further copy should be sent to the CTRC. If the site has moved to an electronic health record (EHR), a scanned copy should be entered in the patient notes. A record of the consent process should also be recorded in the participant's medical notes.

The right of the parent to refuse consent for the infant to participate in the trial without giving reasons must be respected. After the infant has entered the trial, the clinician will remain free to give alternative treatment to that specified in the protocol, at any stage, if s/he feels it to be in the best interest of the patient. However, the reason for doing so should be recorded and the infant will remain within the trial for the purpose of follow-up and data analysis.

Similarly, the parent of the infant remains free to withdraw the infant at any time from the protocol treatment and trial follow-up without giving reasons and without prejudicing the further treatment of the infant (see section 8.6.2 for further details). They do not need to provide a reason for withdrawing their infant from the trial, which will be emphasised in the PISC. Any parent who withdraws their infant from the trial will be approached for participation in the sub-study of parental experience (WP-B), unless they previously declined sub-study participation at consent (see Section 12).

7.2.2 Consent for additional work

Parents of eligible infants who have been approached to give consent for the BESS trial will also be offered the opportunity to take part in a telephone interview about their experiences of the consent process. This will be offered regardless of whether or not consent was given for the infant to participate in BESS (see section12.1).
An alternative PISC will be offered to parents at Liverpool and Southampton PICUs (sites participating in the Mechanistic work exploring surfactant metabolism; WP-C. See section 12.2).

A decision to decline to participate in either WP-B or WP-C will not impact upon care, or preclude involvement in the trial (WP-A).

7.3 Randomisation Procedure

Participants will be randomised in a 1:1 ratio, stratified by site and duration of ventilation prior to randomisation (<24hrs, ≥24hrs).

Randomisation must be completed within 48 hours of intubation.

Participants will be randomised using a secure 24-hour web-based randomisation and drug supply programme, managed by a third party subcontracted by Chiesi Farmaceutici S.p.A. (Chiesi). A personal login username and password will be required to access the randomisation system. Designated research staff will be issued with a unique personal login and password upon completion of training in the use of the system.
When the system requirements (confirmation of consent and eligibility) are confirmed the participant treatment allocation and a unique study number (randomisation number) will be displayed on a secure webpage. The randomising staff member must select the partially completed unblinding envelope corresponding to the participant's randomisation number (as clearly displayed on the front of the envelope) and record the allocation on the printed slip inside. The unblinding envelope must then be sealed and transferred to an agreed secure location accessible to PICU staff.

Once a randomisation number has been generated, the randomising staff member will be able to enter the resupply module within the system, where they will select the participant weight category and dose number (i.e. first, second or third) for the system to allocate the required number of vials for the participant.
Sites must complete this step for participants in both arms of the trial and for every given dose (first, second or third) regardless of allocation to drug or placebo.

The randomisation and resupply system is configured to allow remote monitoring of stock levels at site by the distributor contracted by Chiesi, and further IMP stocks will be supplied once the amount available at site reaches an agreed trigger level. However, it remains the responsibility of the PI or delegated research staff to check the supplies available on the PICU prior to randomisation to ensure there is sufficient supply of the IMP, as the majority of stock will be held at the local site Pharmacy (see Section 9.2.6 for further information regarding intervention accountability).

Unblinded and appropriately delegated site staff will be able to log into the randomisation and resupply system at any time and view the allocations of each participant to ensure continuity between shifts.

7.3.1 Randomisation System Failure

In the event of a randomisation system failure, the centre should contact the coordinating team at the CTRC (Monday to Friday between 9:00 to 17:00 excluding bank holidays) to inform them of the problem. Out-of-hours the centre should contact the Chief Investigator or his delegates, on 07506 653 560.

7.4 Blinding to Allocations

BESS will use an established strategy of a sham-procedure to blind as many research and usual care staff as possible to the nature of the intervention. This sham procedure was used in the development of surfactant as a therapy for surfactant deficiency of prematurity (6,19).

The participants, CTRC staff (excluding statistical team members as appropriate), and all members of the site research teams (excluding agreed randomising staff member, the administrator of the intervention and the dosage witness) will be blinded to treatment allocations. Occasional transient adverse effects associated with surfactant administration and back-tracking of surfactant up the endotracheal tube, plus the more frequently observed positive effects of rapid improvement in gas exchange immediately following surfactant administration, may result in some blinded care givers being able to infer that a participant has been allocated to the surfactant arm of the trial.
The randomising person, person drawing up the surfactant or air placebo and a second person checking the dose will be unblinded. Due to the transient effects and back-tracking it is likely that the Usual Care Nurse (UCN) will become unblinded, and we therefore recommend that if possible the UCN draws up the surfactant or acts as the witness to the drawing up, to minimise the number of people unblinded. Please see figure 2 for an intervention flowchart showing which staff/steps are blinded and unblinded.
Staff who are unblinded due to their involvement or believe themselves to have become unblinded for any reason are strongly discouraged from discussing this with other members of the team, unless the effect noted is causing an adverse event that requires action. Any staff member who believes themselves to have become unblinded must excuse themselves from any decision making regarding the weaning and extubation of the infant. The need to maintain blinding will be emphasised during all Site Training Visits.

8 PARTICIPANT TIME LINE, ASSESSMENTS AND PROCEDURES

8.1 Administration of Intervention

The first trial intervention must be administered within 2 hours of randomisation with subsequent doses (if required) at 12 and 24 hours post randomisation. A grace period of one hour either side of the anticipated administration time is permitted.

- Randomisation; **T0**
- First intervention must be given within two hours; between **T0** and **T2**
- Second intervention should be given 12 hours after randomisation; at **T12** (+/- 1 hour)
- Third intervention should be given 24 hours after randomisation; at **T24** (+/- 1 hour)

8.1.1 Immediately prior to administration

A limited physical examination, with minimal handling, must be undertaken in the two hours prior to each administration of the trial intervention (as per Section 8.5.1).

Prior to intervention and in a drug room away from usual staff, the study respiratory physiotherapist, research nurse or other delegated, unblinded staff member will:

- Draw up a dose of poractant alfa or an equal volume of air as placebo according to the random allocation into a syringe
- Ensure a second person is present to witness this process and check the dose volume
- Cover the syringe with an opaque paper shield to maintain blinding of other staff and parents
- Take the syringe to the bedside, along with syringes of sterile 0.9% sodium chloride for tracheal toilet and the BAL sampling set.

Infants in both the treatment and placebo arms of the trial will have 0.9% sodium chloride “normal saline” tracheal toilet and 0.9% sodium chloride BAL immediately prior to administration of the intervention. Both can cause immediate transient desaturation, need for increased sedation but also and more often rapid improvement in gas exchange when obstructing secretions are removed. Tracheal toilet and BAL can also lead to creamy white secretions appearing in the endotracheal tube, and this will be observed in both treatment and placebo groups.

Staff should remind parents, if present, of the purpose of the imminent study procedure and give them the opportunity to remain or leave as they choose. The usual care nurse will remain to care for the participant and assist as required.

Tracheal toilet is usually required for babies with bronchiolitis and will be done as recommended in the SPC before administration of poractant alfa. BAL sampling with 0.9% sodium chloride (2x1mL/kg) will be done if scheduled (section 8.2). Both tracheal toilet and BAL sampling is done by catheter access to the trachea; either via the endotracheal suction port without disconnecting the

infant from the ventilator or by disconnecting the infant from the ventilator and performing hand bagging while a side-port with one-way valve are utilised for the toilet and BAL.

The use of muscle relaxant prior to tracheal toilet, BAL and or administration of the intervention is at clinical discretion.

8.1.2 Administration of allocated intervention

The intervention will be given per either method described in the SPC; either keeping the infant connected to the ventilator or momentarily disconnecting and performing hand-bagging (at the attending Respiratory Physiotherapist's decision based on patient status).

- Connect the syringe containing the surfactant or air-placebo to a suitable catheter (size 5F);
- Pass the catheter through the suction or side port into the endotracheal tube and advance the catheter, aiming to have the tip of the catheter placed just beyond the tip of the endotracheal tube
- Deliver the intervention (surfactant or air-placebo) slowly as a single bolus (recommended over 0.5-3 minutes) directly into the lower trachea
- The intervention (surfactant or air-placebo) will disperse through mechanical ventilation cycles into the peripheral lung tissue where the excipient (sterile buffered 0.9% sodium chloride) is absorbed in the alveoli and the surfactant phospholipid is deposited.

8.1.3 Post administration of allocated intervention

All participants must be given a limited physical examination, with minimal handling, in the interval between one hour and two hours after the administration of each intervention dose (see section 8.5.1).

8.1.4 Weaning and extubation by protocol

The unblinded staff involved in the administration of the BESS intervention (study respiratory physiotherapist or nurse practitioner or other qualified person and one usual care PICU nurse) and parents who may be observing, will not be involved in decisions to wean respiratory support provided by mechanical ventilation and will not be involved in decisions to extubate.

We will wean respiratory support provided by MV and extubate according to a standardised protocol that will form part of the site-specific training packages.

Readiness for a spontaneous breathing test (SBT) will be identified by the patient meeting specified physiological targets of gas exchange at a given level of ventilation and/or the treating clinician believes the child to be ready for SBT.

The time at which a child is deemed ready for SBT **must** be recorded in their notes/electronic health record. The time that a child is extubated **must** be recorded in their notes/electronic health record.

8.2 Participant follow-up

Clinical follow-up (daily data collection) will take place for the period of time between randomisation and the discharge from hospital of the participant.

All non-serious adverse events will be collected until 24 hours after the participant receives their final trial intervention; please note that this may not be their third dose if less than 3 doses are required before the participant meets the criteria to be ready for weaning (see section 10 for full safety reporting information).

Non-serious adverse events occurring after 24 hours post-final intervention but before 90 days post-randomisation/discharge home/death should be reported if the local Investigator believes that they may be related to the intervention.

Although active monitoring for adverse events and adverse reactions will cease at 24 hours post final intervention and 90 days post randomisation / discharge home / death respectively, any which the site research team become aware of after these time-points which are assessed as serious (i.e. Serious Adverse Events or Serious Adverse Reactions) should be reported to the CTRC.

Routinely collected audit data regarding participant care will be collected from PICANet until 90 days post randomisation.

Parent reported outcomes will be collected via the LRSQ at 6 and 12 months post randomisation for participants recruited in seasons 1 and 2, and at 6 months only for participants recruited in season 3.

The end of follow-up definition for each participant will therefore be 12 months post randomisation for participants in season 1 and 2, or 6 months post randomisation for participants in season 3.

Deja E, *et al. BMJ Open* 2024; 14:e077023. doi: 10.1136/bmjopen-2023-077023

8.3 Schedule for Intervention & Follow-up

Table 2: Schedule for intervention and follow-up

Procedures	Staff	Consent & randomisation	prior to first intervention	1-2hr,post first intervention	6hr	12hr, prior to second intervention	1-2hr post second intervention	24hr, prior to third intervention	1-2hr post third intervention	36hr	48hr	Daily if MV	90d	6m	12m
Prescribe	PI/Dr	X													
Invitation to participate in WP-B (parental experience sub-study)	PI/Dr/RN	X*													
+Invitation to participate in WP-C (surfactant metabolism sub-study)	PI/Dr/RN	X*													
[x]Blood Gas sample (capillary or arterial)	N/RN		X		X	X		X		X	X	X			
CRF completion (patient data collection)	N/RN		X		X	X		X		X	X	X			
Limited physical examination	N/RN/Dr		X	X		X	X	X	X						
[x]BAL	RP		X			X+		X		X+		X			
Administration of trial intervention	RP		X			X		X							
+IV D-Choline	N/RN		X+												
+Blood Sample (venous or arterial)	N/RN		X+			X+		X+		X+	X+				
Safety monitoring and reporting	RN/RP/PI[$]		X		X	X		X		X	X	X			
LRSQ	RN													X	X*
Mortality and re-admission	RN												X		

RN = Research Nurse, RP = Study respiratory physiotherapist or nurse practitioner or any other qualified person not involved in decision to wean or extubate, PI = Local Principal Investigator or delegate, N = Nurse (usual care), Dr = licensed medical practitioner.

[x] Before each trial intervention

+ Only at Liverpool & Southampton and with specific consent (see section 12.2)

* Seasons 1 & 2 only

[$] Although all delegated staff are responsible for identifying and reporting adverse events, decisions regarding causality and seriousness, and sign-off of serious adverse event reports can only be undertaken by a licensed medical practitioner.

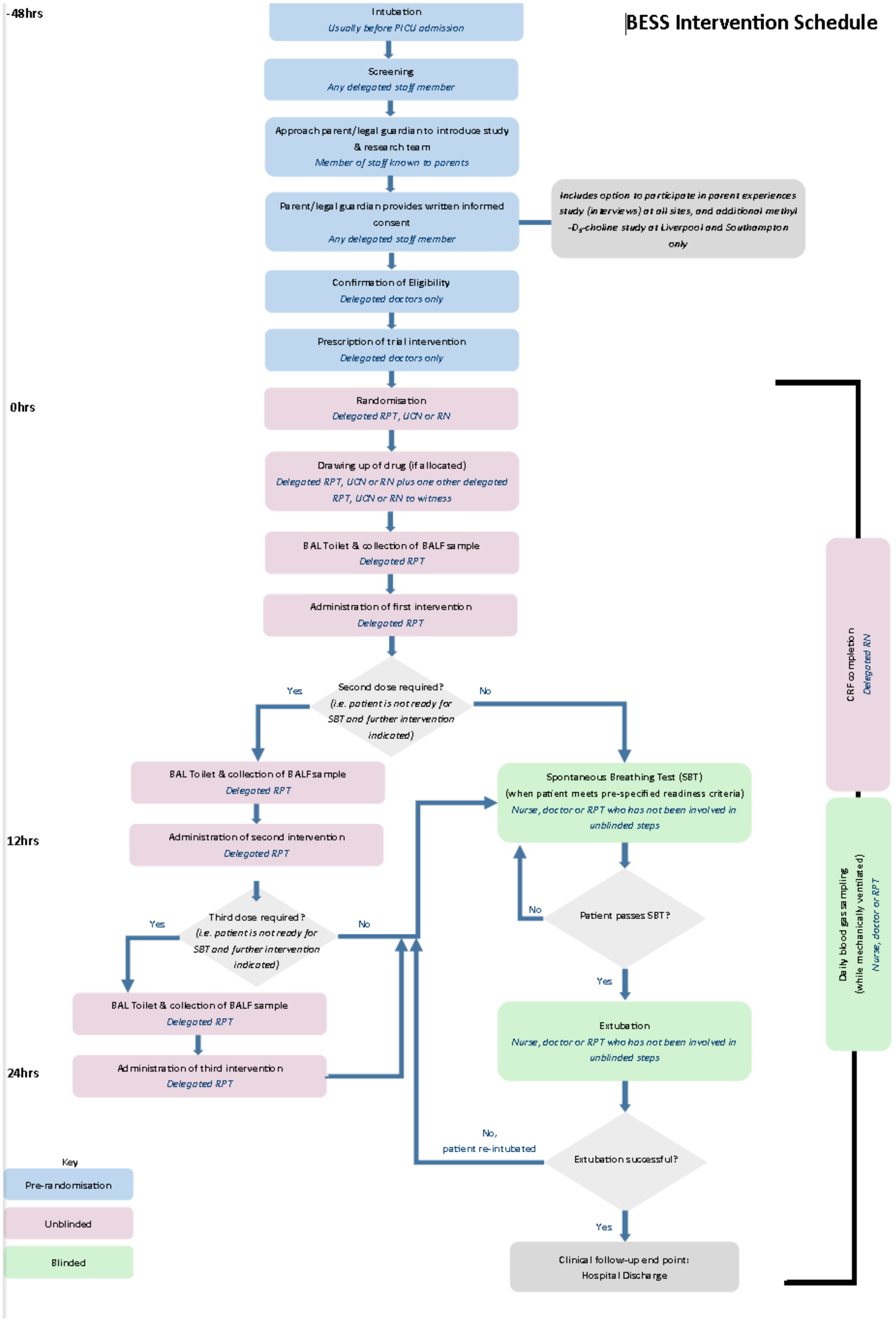

Figure 2: BESS Intervention Schedule

8.4 Procedures for assessing Efficacy

8.4.1 Efficacy Assessment 1

Efficacy will be assessed by group-wise comparison of the primary outcome measure group “Duration of MV after randomisation” by intention to treat. This is defined as time from randomisation to final extubation in hours, including any time off MV where the infant requires reintubation for a further period of MV. Time of randomisation and intervention will be recorded in the patients’ notes and CRFs. MV ceases at extubation, an event when the endotracheal tube (breathing tube) is removed and the infant is allowed to breathe without invasive mechanical support. Extubation is an important discrete event in a patient’s care pathway, is routinely recorded in the notes and PICANet audit.

8.4.2 Efficacy Assessment 2

The Ventilation Index (VI), Oxygenation Index (OI), Oxygenation Saturation Index (OSI) and SF ratio are secondary outcomes of efficacy derived from blood gas results (capillary blood gas sampling times as per Table 2 Schedule for intervention and follow-up), ventilator settings, and non-invasive monitoring. These data are captured through existing intensive care automated monitoring process or regular manual entry on large format (broad-sheet) bed-charts. This data will be transcribed by the research nurse to the CRF.
Mechanical Ventilation parameters to allow calculation of VI, OI, OSI and SF ratio must be recorded at the corresponding time when Blood Gas samples are scheduled to be taken for research or taken for routine purposes.
Mechanical Ventilation parameters are recorded regularly for ventilated patients as part of standard care. BESS does not require site teams to take any additional readings of these parameters for study purposes, but **all blood gas and ventilator readings** taken as part of standard care must be transcribed into the CRF to allow for calculation of these ratios over time.
After extubation, all inhaled Oxygen and peripheral oxygen saturation parameters recorded as part of standard care must be transcribed into the CRF to allow for calculation of this ratio over time.
Blood gas indices and ratio definitions are as follows:

- VI = ($PaCO_2$ × (PIP-PEEP) × respiratory rate)/1,000
- OI = (MAP × FiO_2 × 100)/PaO_2
- OSI = (MAP × FiO_2 × 100)/SpO_2
- SF = SpO_2/FiO_2

MAP = mean airway pressure
$PaCO_2$ = partial pressure of arterial carbon dioxide
FiO_2 = Fraction of air inspired that is oxygen
PaO_2 = partial pressure of arterial of oxygen
SpO_2= peripheral oxygen saturation

The time that physiological criteria are met that indicate suitability to test readiness for extubation by spontaneous breathing and/or the treating clinician believes the child to be ready for SBT will be recorded in the patient medical records and CRF.
All-cause mortality at 90 days after intervention will be established via a local enquiry against the hospital electronic health record.

Deja E, *et al. BMJ Open* 2024; 14:e077023. doi: 10.1136/bmjopen-2023-077023

8.5 Procedures for Assessing Safety

Safety will be assessed by:

- The Principal Investigator (PI) or delegated research staff actively monitoring and reporting all adverse events from randomisation until 24 hours after the participant's last administration of poractant alfa (see section 10 for adverse event reporting).
- Limited physical examination that includes the recording of vital signs before and after each study intervention
- Routinely collected patient care data downloaded from the PICANet audit on a regular basis by each site and transferred to the CTRC
- Mortality and readmission data collected by each site at 90 days post randomisation

Participants will be actively monitored and any adverse events identified must be reported to the CTRC, whether identified from trial assessments or standard care (e.g. identified during standard daily examinations). Non-related events will be reported from randomisation to 24 hours after the participant's last administration of poractant alfa or placebo for. Adverse events occurring after this time point until 90 days post randomisation / discharge home / death will be reported if felt to be related to the intervention. Any serious events or reactions (SAEs/SARs) should be reported to CTRC regardless of the timeframe.

8.5.1 Procedure for Limited Physical Examination in BESS

Participants in BESS are unwell and may be haemodynamically unstable. Handling of the participants, including full physical examination, is minimised in standard care, as per current NICE guidance (42).

All participants must have a physical examination before and after each administration of the trial intervention. This is a limited physical examination to minimise any disturbance to the infant participant.

Examinations must be carried out any time in the period 2 hours prior to each administration, and the interval of time between 1 hour and 2 hours post each administration. The post administration examination is deliberately delayed by at least one hour after administration to allow stabilisation of the participants' vital signs and avoid prolonging the handling associated with administration.

The limited examination must include the following:

- Temperature
- Heart rate
- Blood pressure
- Respiratory rate
- Oxygen saturations (peripheral)

To minimise handling, if a participant has had a full physical examination as part of standard care at any time in the period 2 hours prior to the administration and the vital signs data have been collected, then this examination should be counted as their BESS physical examination and the vital sign data used.

The physical examination vital sign data must be recorded in the patient notes and transcribed onto the relevant BESS CRF.

8.6 Other Assessments

8.6.1 Parent Reported Quality of Life questionnaires

The parent reported outcome measure the Liverpool Respiratory Symptom Questionnaire (LRSQ), is a web-accessible questionnaire that has been optimised for many platforms

Deja E, *et al. BMJ Open* 2024; 14:e077023. doi: 10.1136/bmjopen-2023-077023

including smart-phones. Links to the form will be emailed to parents prior to the intended completion dates (in all three seasons at 6m, and in addition at 12m to parents of infants recruited in seasons 1 and 2). A paper version is available with a "free-post" return envelope.

8.6.2 Special Assays or Procedures

8.6.2.1 Collection & processing of BAL fluid

BAL sampling with 0.9% sodium chloride (2x1mL/kg) will be done for all participants immediately prior to intervention, at 24hrs post intervention and repeated daily while the infant remains on MV (see Table 2).
Both tracheal toilet and BAL sampling is done by catheter access to the trachea via the endotracheal suction port without disconnecting the infant from the ventilator or by disconnecting the infant from the ventilator and performing hand bagging while accessing the trachea via a side-port with one-way valve.

8.6.2.2 Blood gas sampling

Blood Gas sampling is a routine procedure required for the clinical monitoring of all people requiring mechanical ventilation. The typical sample volume of blood drawn from infants and children is 100 microliters equivalent to two drops. Infants and children can have Blood Gas sampling by drawing blood from an existing arterial line or from a heel prick. To minimise disturbance and risks to infants from additional research procedures, the data from routine Blood Gas samples will be used for the trial when collected within a 2-hour window of the scheduled trial Blood Gas sample timepoint up to and including the sample due at 48 hours post first intervention (see Table 2). After 48 hours, i.e. day 3 and onwards, and only if the infant continues to require mechanical ventilation, then data from any one routine blood gas sample collected within a 6-hour window 9:00am to 3:00pm in each subsequent 24-hour interval will be collected (the first such sample if more than one is taken). A research Blood Gas sample will only be done if a routine blood gas sample is not done or planned within each 24-hour period sampling window.
Blood gas machines are usually kept in the PICU rather than the hospital/Trust laboratory. PICUs will not be CPS accredited but each Trust laboratory, and the machinery they are responsible for, will be. The CTRC will acquire a copy of the trust CPS laboratory certificate and calibration log for the blood gas machines as part of the compulsory 'Green Light Checklist' procedures before a site can open to recruitment. The Trust laboratory **must** take responsibility for service of the PICU blood gas machines and be able to provide a quality control log for maintenance and calibration of each blood gas machine.

8.6.2.3 Additional samples (WP-C at Liverpool and Southampton only)

Participants at Liverpool and Southampton will be given the option to consent to a single intravenous dose of D_9-labelled choline which will inform the mechanistic sub-study on surfactant synthesis and metabolism (WP-C; see section 12.2 for full details). This work will not inform either the primary or secondary outcomes of the main trial (WP-A). These samples will **only** be taken from participants who have provided specific consent for this optional additional work.

Small volume lung lavages will be collected immediately prior to intervention and at 24hrs, as per the standard follow-up (see section 8.2), and at additional time points of 12hrs and 36hrs for WP-C participants only. Samples of 1.25mL (range 1.0 to 1.3mL) of blood will be collected at the same time points for WP-C participants at Liverpool and Southampton only.

8.7 Patient Transfer and Withdrawal

8.7.1 Patient Transfers

It is very unlikely that participants taking part in BESS will be transferred during their trial treatment, given the severity of illness of participants and the short time period for administration of the intervention. In the unlikely scenario that a BESS participant is transferred while they remain intubated, their trial treatment will end at transfer unless they are transferred directly to another BESS site. For participants transferred to another participating trial site the receiving trial site must take over responsibility for the participant.

A copy of the participant CRFs should be provided to the new site. The parent will have to sign a new consent form at the new site, and until this occurs, the participant remains the responsibility of the original site.

If a participant is transferred to a site that is not taking part in BESS then they will be withdrawn from the trial and will receive no further interventions. Their care will revert to the standard practice and will not be affected by the withdrawal from the trial. The CTRC should be notified in writing of all participant transfers, whether or not to a participating trial site.

8.7.2 Withdrawal from the Trial

In consenting to the trial, parents are consenting to trial intervention and treatment, follow-up assessments and data collection for their child.

The rights and welfare of the participants will be protected by emphasising to their parents that the quality of medical care will not be adversely affected if they withdraw consent for their child to participate in this trial.

8.7.2.1 Partial Withdrawal from Trial (Intervention and Treatment), allowing Follow-up and Data Collection)

Participants may be withdrawn from trial intervention and treatment for any of the following reasons:

a. Parent withdraws consent.
b. Unacceptable toxicity.
c. Intercurrent illness preventing further treatment.
d. Any change in the participant’s condition that justifies the discontinuation of treatment in the clinician’s opinion.

If voluntary withdrawal from trial intervention and treatment occurs, the parent should be asked to allow continuation of scheduled evaluations (follow-up and data collection) and be given appropriate

care under medical supervision until the symptoms of any adverse event resolve or the participant's condition becomes stable.

If a parent wishes to withdraw their infant from trial intervention and treatment, sites should explain the importance of remaining on trial follow-up, or failing this, of allowing routine follow-up data to be used for trial purposes. Follow-up and data collection will continue unless the parent explicitly withdraws consent for follow-up or data collection. Research data obtained up to the point of withdrawal of consent will be retained, and further data will only be collected where required by law for reporting to regulatory agencies (i.e. serious adverse events). Research samples will be destroyed or retained for the purpose of the trial according to the wishes of the parent.

8.7.2.2 Withdrawal from Trial Completely

Parents are free to withdraw consent at any time without providing a reason. Parents who wish to withdraw consent for the trial completely (trial intervention, follow-up and data collection) will be informed that pseudo-anonymised data collected up to the point of that withdrawal of consent will be included in the analyses, in accordance with current data protection legislation. The participant will not contribute further data to the trial (with the exception of serious adverse event data); the CTRC should be informed in writing and a Withdrawal CRF should be completed. Data up to the time of withdrawal will be included in the analyses.

8.8 Loss to Follow-up

Trial follow-up will be in part via routinely collected data downloaded in a bespoke trial download by each recruiting site; in part via paper CRFs completed by the trial RNs and RPTs; and in part via LRSQs collecting Parent Reported Outcome Measures (PROMs) at 6 and 12 months post randomisation. Where a patient is lost to follow-up before the relevant time point (e.g. lost due to transfer to another hospital), contact will initially be attempted by the trial RNs and the PI at each site. Wherever possible, information on the reason for loss to follow-up will be recorded.

8.9 Trial Closure

The end of the trial is defined to be the date on which data for all participants are locked and data entry privileges are withdrawn from the trial database. However, the trial may be closed prematurely by the Trial Steering Committee (TSC), on the recommendation of the Independent Data and Safety Monitoring Committee (IDSMC).
Individual sites may be closed to recruitment prior to their intended recruitment end date if the Trial Management Group (TMG) have concerns about their capacity or capability to deliver the trial, or for operational reasons whereby resources are better used at sites with better capacity to recruit.
At the point of closure to recruitment all sites will be required to undertake closedown activities which include but are not limited to: a review of their Investigator Site File (ISF), a count of all completed CRFs and completed data queries. PIs will also be required to sign-off the CRF for each participant, any changes to the data, a closedown checklist and their site delegation log.

Deja E, *et al. BMJ Open* 2024; 14:e077023. doi: 10.1136/bmjopen-2023-077023

9 TRIAL TREATMENT/INTERVENTIONS

9.1 Introduction

The intervention being trialled is Endotracheal poractant alfa (Curosurf®, porcine lung phospholipid fraction supplied at 80mg/mL).

Curosurf® is a standard natural surfactant treatment prepared from porcine lungs for intratracheal administration and containing almost exclusively polar lipids, in particular phosphatidylcholine. Endotracheal poractant alfa is licensed for use in the UK for the treatment of Respiratory Distress Syndrome (RDS) or hyaline membrane disease in newborn babies (neonates). It is being used as an investigational medicinal product (IMP) on the BESS trial (UK licence, new indication).

Three small studies of surfactant use in infants with bronchiolitis have raised no safety concerns, however BESS will be the first large, randomised study of any surfactant for the treatment of bronchiolitis of infancy (3). As neonatal RDS is caused by surfactant deficiency, and the viral infections that cause bronchiolitis of infancy also cause surfactant deficiency (which we aim to treat with poractant alfa), we expect that the well tolerated safety profile of use in RDS in neonates will be reflected when using surfactant to treat bronchiolitis in infants.

Participants will be randomised equally to receive either endotracheal poractant alfa or air-placebo in a sham procedure. Participants randomised to receive endotracheal poractant alfa will receive a first dose of 200mg/kg, repeated 12 hourly at 100mg/kg, to a maximum of 3 doses. This is in line with the manufacturer’s summary of product characteristics (SPC). Participants randomised to the placebo will receive an identical series of up to three procedures (while intubated) using air as the placebo.

9.2 Arm A – Endotracheal Poractant Alfa at 200mg/kg (first dose), with up to two further doses at 100mg/kg

9.2.1 Formulation, Packaging, Labelling, Storage and Stability

Generic name: Endotracheal poractant alfa

Brand name: Curosurf®

Supply and distribution: Chiesi Farmaceutici S.p.A (Chiesi) will supply Curosurf® via a third-party distributor and will provide appropriate certificates of analytical conformity. Additional shipments during each season will be managed by the distributor contracted by Chiesi, via an automated web-based system.

Packaging: Labelled holder containing one labelled glass vial with a blue topper.

Formulation: Each vial contains 3ml volume of poractant alfa at a concentration of 80mg/ml.

Labelling: Vials will be labelled by Chiesi in English and in compliance with the detailed guidance provided in annex 13 of the European Union (EU) Good Manufacturing Practice (GMP) guide. Each vial will be labelled and used for BESS trial use only.

Storage: The PI or delegated research team member will be responsible for the safe storage of all vials assigned to the BESS trial, in a safe place with restricted access (usually a designated drug room on the PICU). Poractant alfa will be stored between 2ºC and 8ºC, in a separate drug fridge specifically for use in the trial, protected from light in the original packaging.

Stability: Curosurf® has an unopened shelf life of 18 months. Each vial is for single use only.

9.2.2 Preparation, Dosage and Administration of Study Treatment/s

Dosage: An initial dose of 200mg/kg, with two further doses of 100mg/kg at 12hrs and 24hrs if indicated.
200mg/kg is 200milligrams of poractant alfa per kilogram of body weight. 100mg/kg is 100milligrams of poractant alfa per kilogram of body weight. The participant's weight will be detailed on the prescription in kilograms and the prescribing clinician will calculate the appropriate dose and number of vials needed for that participant.
Preparation: Poractant Alfa suspension is refrigerated, so before use each vial must be warmed up slowly to room temperature e.g. keeping it in hands for 5-10 minutes and gently turned upside down, without shaking, avoiding the formation of foam. Artificial warming methods should not be used.
The entire contents of the vials of poractant alfa suspension should be slowly withdrawn into a plastic syringe through a large-gauge needle and the excess poractant alfa discarded. A second person must be witness to the drawing up of the poractant alfa to assist and to check that the volume drawn up is the amount prescribed. Once the prescribed volume has been drawn into the syringe, an opaque paper shield should be applied to the syringe in order to maintain blinding of staff and/or parents at the bedside.
At this stage the appropriate equipment should also be prepared for conducting tracheal toilet and BAL.
Tracheal toilet (and, where scheduled, BAL) must be conducted prior to administration of the intervention.
The use of muscle relaxant prior to tracheal toilet, BAL and or administration of the intervention is at clinical discretion.

Administration: Either with the infant connected to the ventilator, or momentarily disconnected with hand-bagging, the syringe filled with Poractant Alfa suspension will be connected to a 5-French catheter end-hole inserted into the endotracheal tube (ETT) for ventilation. Passing the catheter through the suction or side port into the ETT and advancing the catheter aiming to have the tip of the catheter placed just beyond the tip of the ETT. The insertion length should be calculated from the known length of the ETT (typically cut to reduce dead space) plus the distance from the end of the cut edge of the ETT to the suction/side port). The intervention will be given slowly as a single bolus directly into the lower trachea (recommended over 0.5 to 3 minutes). From there it disperses through ventilation cycles into the peripheral lung tissue where the excipient (buffered 0.9% sodium chloride) is absorbed in the alveoli and the surfactant phospholipid is deposited.
The infant should be stabilized before surfactant administration and their head placed in the neutral position (head and body in alignment without inclination).

Before starting the procedure and throughout it, infants must have cardiorespiratory monitoring: oxygen saturation and heart rate continuously through pulse oximetry, blood pressure measurement either continuously in presence of an arterial catheter or intermittently if no arterial line is in place.
The first dose at 200 mg/kg will be followed up by two further doses at 100 mg/kg at approximately 12-hour intervals and the exact time and actual volume of each administration will be noted in the medical notes and appropriate section of the CRF.
No suctioning of the airways should be performed for two hours after each surfactant instillation unless the treating physician deems this appropriate, in which case this will be noted in the medical notes and appropriate section of the CRF.

9.2.3 Specific Restrictions

The effect of co-administration of poractant alfa with other agents is not known. Do not administer poractant alfa concomitantly in the same intratracheal bolus with other agents.

9.2.4 Overdose

Overdose may occur if the wrong patient weight has been supplied to the prescribing clinician, an incorrect weight is used in the calculation or the calculation has been done incorrectly. There have been no reports of overdose following the administration of Curosurf® however, in the unlikely event of an accidental overdose that causes clear clinical effects on the infant’s respiration, ventilation or oxygenation, as much of the suspension should be aspirated as possible, as per the SPC.
Special care must be taken by all site team members to avoid overdosing, noticeably the prescribing clinician and the staff members drawing up the poractant alfa for administration. In the event of identification of an overdose, unblinding should occur and the participant will be closely observed for any adverse events (AE). The site team should notify the CTRC of the event and examine the reasons for the event occurring to ensure that it does not happen again. The CTRC will log any overdosing events as protocol deviations.

9.2.5 Temperature Compliance

Temperature deviations at site will be identified by the regular completion of a trial-specific temperature monitoring log. The PI or delegated member of staff will check the fridge used to store stocks of poractant alfa for use in BESS **daily** and will alert the CTRC if a temperature deviation has occurred. Temperature checks will be recorded on a Temperature Monitoring Log.

Poractant Alfa shipment and storage conditions:

**Poractant Alfa (Curosurf®)** Suspension 80 mg/ml to 3ml vial	+2°C to +8°C	Up to +25°C for maximum cumulative 96hrs

Unopened vials of Curosurf® that have warmed to room temperature can be returned to refrigerated storage **within 24hours** for future use, **if** this is the first time that they have warmed. Vials that have been removed from the fridge and been allowed to warm to room temperature for a second time **must not be used**, but should be retained for accountability checks at the end of each season.

Temperature compliance during shipment will be checked by the local site Pharmacist on receipt of the IMP. The local Pharmacist will review the temperature curves/logs from the calibrated

temperature logger used during IMP shipment and will check for any out of range values. If an out of range temperature is identified, the local Pharmacist will inform the CTRC within 48 hours. It is the responsibility of the CTRC to inform Chiesi, who will then notify the Chiesi R&D Quality Assurance GMP Unit & Project Partners (R&D QA-GMP).

If the out-of-range temperature deviation does not affect the quality and safety of the products, a declaration of usability will be issued and signed by R&D QA-GMP and sent back to the CTRC. CTRC will then authorise the trial site to use the vials in question.

If the products are judged not suitable for use, Chiesi R&D QA-GMP will inform the CTRC to permanently suspend the use of the affected vials. Any vials not used due to temperature deviations will be retained for accountability checks at the end of each season before being sent for destruction.

9.2.6 Accountability Procedures for Trial Treatments

The PI is responsible for the management of poractant alfa to be used for the trial. Trial IMP should be stored in the original packaging to protect from light, in a locked, secure, refrigerated storage facility with access limited to those individuals authorized to dispense or administer the trial IMP. Initial supply of the trial IMP will be to the site Pharmacy. The site Pharmacists are responsible for dispensing stock to a secure drug room on the PICU for more immediate access by the research team. Pharmacists must release the trial IMP to PICU in sequential order by the medication numbers on the labels to ensure the Randomisation System can allocate trial IMP with the shortest shelf life first. No surfactant material supplied for this trial is to be used for any other purpose.

Accountability logs will be maintained by the PI, pharmacist or delegated research team member, which will include a signed account of all the vials of Curosurf® received from the distributor, the vials dispensed to and returned from PICU, with the vials and volume used for each randomised patient.

All completely used vials will be retained at the site until the conclusion of the recruitment season. Each vial is for single use only. Any leftover unused medication is to be retained for drug accountability. Partially used vials present a risk of confusion and sharps injury, so all partially used vials will be disposed of within the PICU as per local Trust standard procedures. Disposal of partially used vials will be logged for accountability checks.
At the conclusion of each recruitment season, the PI, pharmacist or delegated individual shall conduct and document a drug supply (used and unused) inventory. An explanation will be given for any discrepancies.

After the inventory is reconciled at the end of each season and only once the CTRC have given the site authorisation on behalf of the sponsor, remaining unused vials of Curosurf® maybe disposed of directly by the Pharmacy of the involved trial site. Unused vials with an expiry date during or after the next season will be retained. Documentation assuring the unused vials were sent off-site for destruction, as per standard Trust procedures will be provided. This must be signed by two delegated members of staff filed in the Pharmacy site file, with a copy returned to the CTRC for storage in the Trial Master File.

9.2.7 Assessment of Compliance with Study Treatment/s

Each vial of Curosurf® will be designated by code number and the actual volume of instilled test treatment will be noted in the hospital chart and on the CRF of the study.

The completely used vials will be kept in a sealed labelled plastic box/envelope with patient's randomisation number/initials until the end of the season for accountability checks. There will therefore be three "final" accountability checks at each site, one at the end of each recruitment season.

9.3 Arm B – Placebo

Placebo: Air via an identical series of 3 doses in a sham procedure.

Dosage and preparation: Air should be drawn into a syringe at the same volume of 200mg/kg for the first dose and 100mg/kg for the second and third doses (if required), as per the instructions for poractant alfa dose calculations assuming the intervention is provided at 80mg/ml (section 9.2.2). Once the prescribed volume has been drawn into the syringe, an opaque paper shield should be applied to the syringe in order to maintain blinding of staff and/or parents at the bedside.

The use of muscle relaxant prior to tracheal toilet, BAL and or administration of the intervention is at clinical discretion.

Administration: Either with the infant connected to the ventilator, or momentarily disconnected with hand-bagging, the syringe filled with air will be connected to a 5-French catheter end-hole inserted into the endotracheal tube (ETT) for ventilation. Passing the catheter through the suction or side-port into the ETT and advancing the catheter aiming to have the tip of the catheter placed just beyond the tip of the ETT. The insertion length should be calculated from the known length of the ETT (typically cut to reduce dead space) plus the distance from the end of the cut edge of the ETT to the suction/side port). The air will be given slowly as a single bolus directly into the lower trachea (recommended over 0.5 to 3 minutes).

9.4 Unblinding

N.B. Allocation must not be routinely revealed to CTRC personnel. It is to be expected that some site staff may be accidentally unblinded due to unavoidable back-tracking of surfactant up the participant's ETT (see section 7.4).

To maintain the overall quality and legitimacy of the trial, unblinding should occur only in exceptional circumstances when knowledge of the actual treatment is **absolutely essential** for further management of the patient (SPIRIT 2013).

After unblinding the Local Investigator is encouraged to maintain the blind as far as possible. The actual allocation must NOT be disclosed to the participant's family or other study personnel including

other site personnel, monitors, sponsors or project office staff; nor should there be any written or verbal disclosure of the code in any of the corresponding patient documents.

The Investigator may unblind at their discretion without need to contact the CTRC first, but must report all unblinding (with reason) as they occur on the corresponding Unblinding CRF.

Unblinding should not necessarily be a reason for study drug discontinuation.

9.4.1 Emergency Unblinding

If a Local Investigator urgently needs to unblind a participant, they are able to do so by opening the unblinding envelope corresponding to the participant's randomisation number.

Partially completed unblinded envelopes will be provided to all sites. The allocation (poractant alfa or air as placebo), as generated by the WebEZ randomisation system, will be marked on a pre-printed slip inside the envelope by the randomising team member, and the envelope sealed. There will be one partially completed envelope for each patient, with the randomisation number clearly visible on the front of the envelope.

Sealed unblinding envelopes should be stored in a secure location that is readily accessible to PICU staff if the need for emergency unblinding arises.

In the case of any adverse event that requires emergency treatment and there is not sufficient time to unblind using the unblinding envelopes, Local Investigators should assume that the participant has been given poractant alfa (Arm A) and administer treatment accordingly. The treatment for any acute adverse event potentially associated with the study intervention would be the same whether the patient had been given surfactant or air. All adverse events should be reported as per the procedures described in section 10.

9.4.2 Accidental unblinding

If accidental unblinding occurs, this must be reported to the CTRC by use of the Unblinding CRF. When reporting include details about:

1. Date of unblinding;
2. Detailed explanation of circumstances;
3. Recipients of the unblinding information;
4. Action to prevent further occurrence, if possible.

9.4.3 Unblinding at Trial Closure

Upon trial closure the blinding of parents and blinded staff remains in effect. Site teams and/or pharmacy departments **should not** disclose treatment allocations on an individual basis.

9.5 Concomitant Medications

No incompatibilities or interactions of Curosurf® with other medications are known. There are therefore no restrictions placed by the trial on the use of concomitant medications in BESS participants.

10 SAFETY REPORTING

10.1 Terms and Definitions

"Adverse Event (AE)"

An adverse event (AE) is any untoward medical occurrence in a subject to whom a medicinal product has been administered, including occurrences which are not necessarily caused by or related to that product.

Therefore an AE is any unfavourable or unintended change in the function (symptoms), structure (signs) or chemistry (laboratory data) in a subject to whom an IMP has been administered, including occurrences which are not necessarily caused by or related to that product.

"Adverse Reaction (AR)"

An adverse reaction (AR) is any untoward and unintended response in a subject to an IMP that is related to any dose administered to that subject.

Therefore an AR is any unfavourable or unintended change in the function (symptoms), structure (signs) or chemistry (laboratory data) in a subject that is related to any dose of an IMP administered to that subject.

"Unexpected Adverse Reaction (UAR)"

An unexpected adverse reaction (UAR) is an adverse reaction the nature and severity of which is not consistent with the information about the medicinal product in question set out in the Investigator's Brochure (IB) or the SPC, which may be referenced where the IMP in question is a product with a marketing authorisation.

"Serious Adverse Event (SAE), "Serious Adverse Reaction, or Unexpected Serious Adverse Reaction"

A serious adverse event (SAE) is an AE, AR or UAR respectively that:

- results in death;
- is life threatening (places the subject, in the view of the Investigator, at immediate risk of death from the experience as it occurred – this does not include an adverse experience that, had it occurred in a more severe form, might have caused death);
- requires hospitalisation or prolongation of existing hospitalisation (hospitalisation is defined as an in-patient admission, regardless of length of stay, even if the hospitalisation is a precautionary measure for continued observation – hospitalisations for a pre-existing condition, including elective procedures that have not worsened, do not constitute an SAE);
- results in persistent or significant disability or incapacity (substantial disruption of one's ability to conduct normal life functions);
- consists of a congenital anomaly or birth defect (in offspring of subjects, or their partners, taking the IMP regardless of time of diagnosis);
- other important medical events (these may not result in death, be life-threatening, or require hospitalisation, but may be considered a serious adverse event or experience when, based upon appropriate medical judgment, they are considered to jeopardise the subject and may require medical or surgical intervention to prevent one of the outcomes listed in this definition).

"Suspected Serious Adverse Reaction (SSAR)"

A suspected serious adverse reaction (SSAR) is an adverse reaction that is classed in nature as serious and which is consistent with the information about the IMP in question, which in the case of a licensed product is set out in the SPC for that product, and in the case of any other IMP is set out in the IB relating to the trial in question.

"Suspected Unexpected Serious Adverse Reaction (SUSAR)"

A suspected unexpected serious adverse reaction (SUSAR) is an adverse reaction that is classed in nature as serious and which is not consistent with the information about the IMP in question, which in the case of a licensed product is set out in the SPC for that product, and in the case of any other IMP is set out in the IB relating to the trial in question.

"Reference Safety Information (RSI)"

The reference safety information (RSI) for a trial is the information used for assessing whether an adverse reaction is expected. This is contained in either the IB or the SPC.

10.2 Notes on Adverse Event Inclusions and Exclusions

The below sections provide guidance on what should, and should not be considered as AEs for the purposes of the BESS study:

10.2.1 Include:

- An exacerbation of a pre-existing illness, **excluding** the current case of bronchiolitis.
- An increase in frequency or intensity of a pre-existing episodic event/condition.
- A condition (even though it may have been present prior to the start of the trial) detected after trial drug administration.
- Continuous persistent disease or symptoms present at baseline that worsen following the administration of the trial treatment, **excluding** the current case of Bronchiolitis.
- Laboratory abnormalities that require clinical intervention or further investigation (unless they are associated with an already reported clinical event).
- Abnormalities in physiological testing or physical examination that require further investigation or clinical intervention.
- Injury or accidents.

10.2.2 Do Not Include:

- Widely recognised and documented clinical features of bronchiolitis or outcomes associated with MV that are defined outcome measures in this trial (see section 3.5).
- Medical or surgical procedures* – the condition which leads to the procedure is the adverse event; e.g. pneumothorax leading to chest drain placement, here pneumothorax is the event.
- Pre-existing disease or conditions present before treatment that do not worsen.
- Situations where an untoward medical occurrence has occurred, e.g. cosmetic elective surgery.
- Overdose of medication without symptoms or signs**.

* *Cosmetic elective surgery is cited here as example of an event that is not reportable as an AE.*
** See also section 9.2.4. *If overdose occurred with resulting symptoms and signs that met the protocol criteria for AE/AR/SAE/SAR then they should be reported accordingly. Please note that though overdose of medication without symptoms or signs may be excluded from AE reporting this may still require investigation to ensure the protocol and regulatory requirements are met, e.g. for IMP management and administration to ensure participant safety.*

10.2.3 Notification of deaths

There is an overall likelihood of death in all cases of severe bronchiolitis of 1-2%. All deaths that occur during the protocol-specified AE reporting period, regardless of relationship to study drug, must be recorded. All deaths should be reported on a “Serious Adverse Event CRF” and returned to the CTRC within 24 hours.

Death should be considered an outcome and not a distinct event. The event or condition that caused or contributed to the fatal outcome should be recorded as the single medical concept on the appropriate CRF; e.g. death following pneumothorax, here pneumothorax is the event. Generally, only one such event should be reported. The term “sudden death” should only be used for the occurrence of an abrupt and unexpected death due to presumed cardiac causes in a participant with or without pre-existing heart disease, within 1 hour of the onset of acute symptoms or, in the case of an unwitnessed death, within 24 hours after the participant was last seen alive and stable. If the cause of death is unknown and cannot be ascertained at the time of reporting, “unexplained death” should be recorded on the appropriate CRF. If the cause of death subsequently becomes available (e.g. after post-mortem examination), “unexplained death” should be replaced by the established cause of death.

10.3 Notes on Severity / Grading of Adverse Events

The assignment of the severity/grading should be made by the investigator responsible for the care of the participant using the definitions below.

Regardless of the classification of an AE as serious or not, its severity must be assessed according to medical criteria alone using the following categories:

Mild: does not interfere with routine activities
Moderate: interferes with routine activities

Severe: impossible to perform routine activities

A distinction is drawn between serious and severe AEs. Severity is a measure of intensity (see above) whereas seriousness is defined using the criteria in section 10.1, hence, a severe AE need not necessarily be a SAE.

Deja E, *et al. BMJ Open* 2024; 14:e077023. doi: 10.1136/bmjopen-2023-077023

10.4 Relationship to Trial Treatment

The assignment of the causality should be made by the investigator responsible for the care of the participant using the definitions in Table 3: Definitions of Causality. An AE for which the causal relationship to the study drug is assessed by the investigator as “possible”, “probable” or “almost certain” is an AR.

If any doubt about the causality exists the local investigator should inform the CTRC who will notify the Chief Investigator (CI). In the case of discrepant views on causality between the investigator and others, the MHRA will be informed of both points of view.

Table 3: Definitions of Causality

Relationship	Description
**Unrelated**	There is no evidence of any causal relationship. N.B. An alternative cause for the AE should be given.
**Unlikely**	There is little evidence to suggest there is a causal relationship (e.g. the event did not occur within a reasonable time after administration of the trial medication). There is another reasonable explanation for the event (e.g. the participant’s clinical condition, other concomitant treatment).
**Possibly**	There is some evidence to suggest a causal relationship (e.g. because the event occurs within a reasonable time after administration of the trial medication). However, the influence of other factors may have contributed to the event (e.g. the participant’s clinical condition, other concomitant treatments).
**Probably**	There is evidence to suggest a causal relationship and the influence of other factors is unlikely.
**Almost certainly**	There is clear evidence to suggest a causal relationship and other possible contributing factors can be ruled out.

10.5 Expectedness

The Reference Safety Information (RSI) in BESS is section 4.8 (Undesirable effects) of the Curosurf® Endotracheopulmonary Instillation Suspension SPC.

It is not a regulatory requirement for a reporting physician to provide their opinion of expectedness. Therefore, the reporting physician at the local research site will not be asked to make an assessment of expectedness. The assessment of expectedness will be made by the CI (or their medically qualified delegate) using BESS’s current MHRA-approved RSI following receipt of the “Serious Adverse Event CRF” at CTRC.

All events judged by the investigator to be possibly, probably, or almost certainly related to the IMP, graded as serious and unexpected (see current MHRA-approved trial RSI (Curosurf® Endotracheopulmonary Instillation Suspension SPC) for list of Expected Adverse Events) should be reported as a SUSAR.

Deja E, *et al. BMJ Open* 2024; 14:e077023. doi: 10.1136/bmjopen-2023-077023

10.6 Follow-up After Adverse Events

All adverse events should be followed until satisfactory resolution or until the investigator responsible for the care of the participant deems the event to be chronic or the participant to be stable.

When reporting SAEs and SUSARs the investigator responsible for the care of the participant should apply the following criteria to provide information relating to event outcomes:

- resolved;
- resolved with sequelae (specifying with additional narrative);
- not resolved/ongoing;
- on-going at final follow-up;
- fatal or unknown.

10.7 Time Period for Safety Reporting

Investigators and delegated members of the local research teams will report to CTRC on the appropriate CRFs (see section 10.8):

- all adverse events from randomisation until 24 hours after their final intervention (whether poractant alfa or placebo);
- all adverse reactions from randomisation until 90 days post-randomisation or hospital discharge (whichever is sooner).

Following these periods, site staff are not required to report any non-serious events, however any serious events (SAEs/SARs/SUSARs) which they become aware of must be reported to CTRC.

Upon becoming aware of a serious adverse event (SAE/SAR/SUSAR), the Investigator or other delegated member of the team **must** report this to the CTRC **within 24 hours**.

10.8 Reporting Procedures

All serious events (SAEs/SARs/SUSARs) must be reported in an expedited manner. Depending on the nature of the event the reporting procedures below should be followed. Any questions concerning adverse event reporting should be directed to the CTRC in the first instance. If a site has a serious or urgent query regarding an adverse event outside of CTRC office hours they should contact the Chief Investigator, Professor Calum Semple, on 07506 653 560.

A flowchart is given below to aid in determining reporting requirements.

10.8.1 Flowchart for Reporting Requirements of Adverse Events

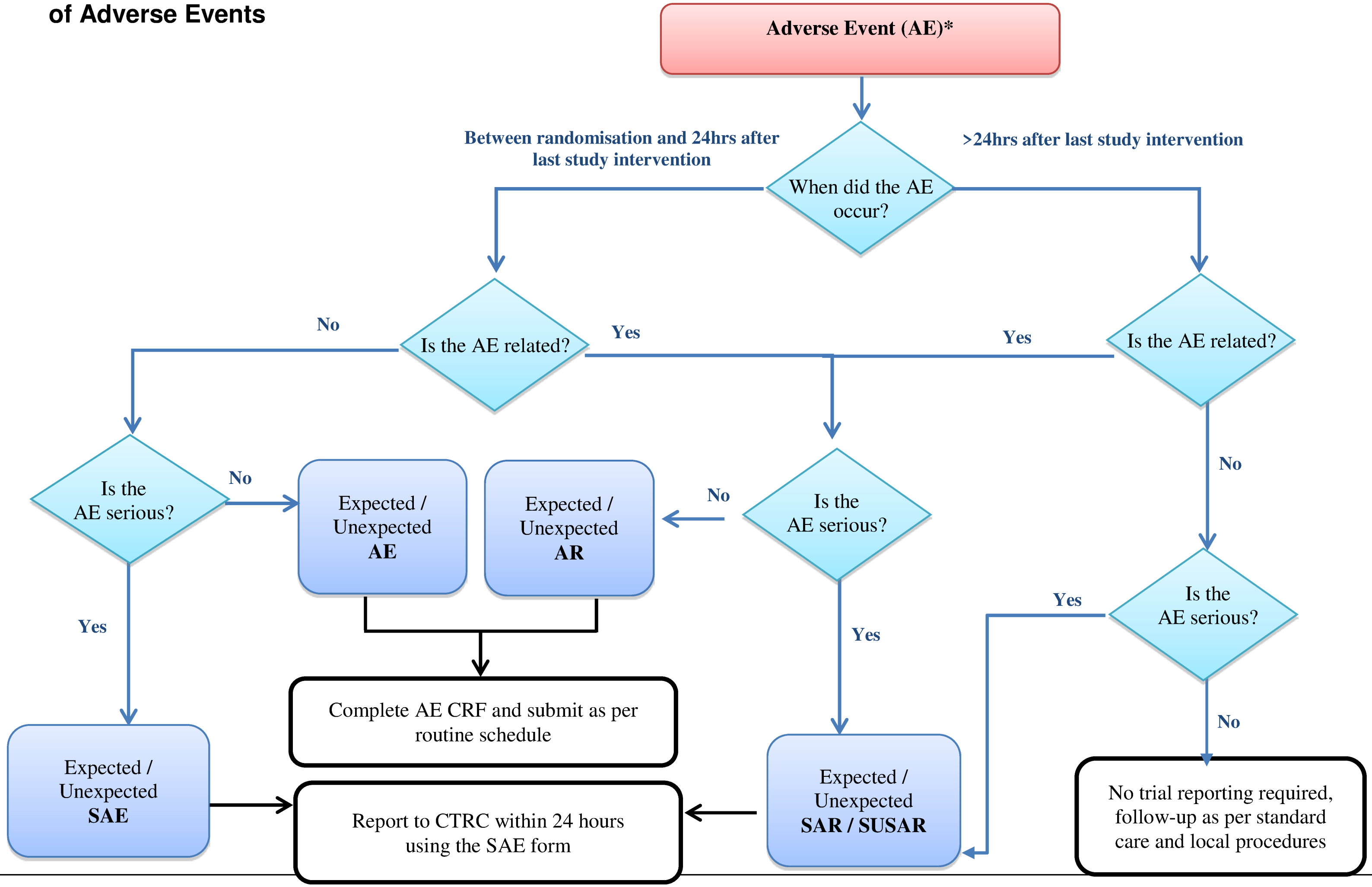

Deja E, *et al. BMJ Open* 2024; 14:e077023. doi: 10.1136/bmjopen-2023-077023

BMJ Publishing Group Limited (BMJ) disclaims all liability and responsibility arising from any reliance placed on this supplemental material which has been supplied by the author(s)

Deja E, *et al. BMJ Open* 2024; 14:e077023. doi: 10.1136/bmjopen-2023-077023

10.8.2 Non serious events (ARs/AEs)

All non-serious events (i.e. AEs and ARs), whether expected or not, should be recorded on an “Adverse Event CRF”, which should be transmitted to the CTRC within seven days of the local research team becoming aware of the event.

10.8.3 Serious events (AEs/ARs/SUSARs)

All serious events (i.e. SAEs, SARs and SUSARs) must be reported to CTRC within 24 hours of the local site becoming aware of the event. The “Serious Adverse Event CRF” asks for the nature of the event, date of onset, severity, corrective therapies given, outcome and causality. The reporting local Investigator should assign the causality of the event. Additional information should be sent within 5 days if the event has not resolved at the time of initial reporting.

All adverse events assessed as serious, related and unexpected (i.e. SUSARs) will be reported on to the MHRA and BESS’s Research Ethics Committee (REC) by the CTRC according to the following timelines; fatal and life-threatening SUSARs within 7 days of notification to CTRC and non-life threatening SUSARs within 15 days. BESS PIs will be notified of all SUSARs occurring throughout the trial by the CTRC. Local Investigators should report any SUSARs and/or SAEs/SARs as required locally.

NOTE: *please ensure that multiple Serious Adverse Events are reported separately to the CTRC. Each individual SAE report should relate to one overall diagnosis only.*

10.9 Safety Reporting Responsibilities – Investigator

The local site Investigator (site Principal Investigator (PI)) is responsible for reporting all events that are observed or reported during the study, regardless of their relationship to study product (i.e. AEs/ARs/SAEs/SARs/SUSARs)..

All serious adverse events (SAEs/SARs/SUSARs) must be reported immediately by the investigator to the CTRC on an SAE. All non-serious adverse events should be reported to CTRC within seven days of the local research team becoming aware on an AE CRF.

Minimum information required for reporting serious events

- Valid European Clinical Trials Database (EudraCT) number (if applicable)
- Sponsor study number
- One identifiable coded subject (randomisation number)
- One identifiable reporter (name of PI or appropriate delegated individual)
- One SAE
- One suspect IMP (poractant alfa)
- A seriousness assessment
- A causality assessment

Process for reporting serious events

i. The SAE CRF should be completed by a local Investigator, named on the 'signature list and delegation of responsibilities log' as responsible for reporting SAEs and making trial related medical decisions. The Investigator should assess the SAE for the likelihood that it is a response to the trial intervention. In the absence of the designated Investigator the SAE CRF should be completed and signed by an alternative member of the research site trial team and submitted to the CTRC. As soon as possible thereafter the responsible Investigator should check the SAE CRF, make amendments as appropriate, sign and re-send to the CTRC. The initial report shall be followed by detailed follow-up CRF reports as appropriate (see number vii).

ii. The "Serious Adverse Event CRF" **must** be submitted to the CTRC **within 24 hours** of the local research team becoming aware of the event; preferably this will be via fax.

Email address: bess@liverpool.ac.uk

Fax Number: 0151 795 8770

iii. When submitting a SAE CRF to the CTRC research sites must also telephone the appropriate Trial Co-ordinator or Data Manager on telephone number **0151 795 8757** to advise that an SAE CRF has been submitted.

iv. The participant **must** be identified by trial randomisation number, date of birth and initials **only**. The participant's name **must not** be used on any correspondence.

v. The local reporting Investigator must **notify** their R&D department of the event (as per standard local governance procedures).

vi. The participant must be followed-up until clinical recovery is complete and laboratory results have returned to normal, or until the event has stabilised. Follow-up should continue after completion of protocol treatment as necessary.

vii. Follow-up information should be noted on a separate CRF by ticking the box marked 'follow-up' and submitting to the CTRC as information becomes available. Extra, annotated information and/or anonymised copies of test results may be provided separately.

10.9.1 Maintenance of Blinding

Systems for SUSAR and SAR reporting should, as far as possible, maintain blinding of individual clinicians and of trials staff involved in the day-to-day running of the trial. The medical management of participants for adverse events should be undertaken under the assumption that the patient has been given surfactant (Arm A). The safety of participants in the trial always takes priority. In each report, seriousness, causality and expectedness should be evaluated for poractant alfa unless unblinding has taken place. All instances of unblinding should be reported to the CTRC.

Cases that are considered serious, unexpected and possibly, probably or almost certainly related to one of the trial therapies (i.e. SUSARs) would have to be unblinded at the CTRC prior to reporting to the MHRA and REC.

10.10 Safety Reporting Responsibilities – CTRC

The CTRC is undertaking duties delegated by the trial Sponsor and is responsible for the notification of serious events (SAEs/SARs/SUSARs) to the CI (or appropriate delegate) for review and expectedness assessment, and onward expedited reporting of SUSARs and other SARs to the MHRA and REC. The CTRC will also notify PIs at participating sites of all trial SUSARs.

Upon receipt of a SAE CRF, CTRC will liaise with the trial CI (or appropriate delegate) who will review the events reported for seriousness and causality as assessed by the local site research team, and provide and assessment of expectedness. These will be reviewed within 24 hours of receipt at CTRC and those that are identified as SUSARs will be reported to MHRA, REC and Chiesi Farmaceutici S.p.A. by the CTRC. The causality assessment given by the local Investigator at the hospital cannot be overruled and in the case of disagreement, both opinions will be provided with the report.

Timelines of onward reporting to MHRA and REC are as follows:

- SUSARs that are fatal or life-threatening must be reported not later than 7 days after the CTRC is first aware of the reaction. Any additional relevant information must be reported within a further 8 days.
- SUSARs that are not fatal or life-threatening must be reported within 15 days of the CTRC first becoming aware of the reaction.
- A list of all SARs (expected and unexpected) must be reported annually.

The CTRC will also inform Chiesi Global Pharmacovigilance (Parma, Italy) of any adverse event identified as a SUSAR, within 7 days of the CTRC first becoming aware of the reaction.

It is recommended that the following safety issues should also be reported in an expedited fashion:

- an increase in the rate of occurrence or a qualitative change of an expected SAR, which is judged to be clinically important;
- new events related to the conduct of the trial or the development of the IMP and likely to affect the safety of the participants, such as:
    a. a SAE that could be associated with the trial procedures and which could modify the conduct of the trial;
    b. a significant hazard to the participant population, such as lack of efficacy of an IMP used for the treatment of a life-threatening disease;
    c. a major safety finding from a newly completed animal study (such as carcinogenicity);
    d. any anticipated end or temporary halt of a trial for safety reasons and conducted with the same IMP in another country by the same sponsor;
- recommendations of the IDSMC, if any, where relevant for the safety of the participants.

Participant safety incidents that take place in the course of research should be reported to the National Reporting and Learning System (NRLS) by each participating NHS Trust in accordance with local reporting procedures.

10.11 Safety reports

Safety reports will be generated during the course of the trial which allows for monitoring of serious events (SAEs/SARs/SUSARs) reporting rates across sites. The CTRC will send developmental safety update reports (DSUR) containing a list of all SARs to MHRA and the trial's main REC. Any concerns raised by the IDSMC or inconsistencies noted at a given site may prompt additional training at sites, with the potential for the CTRC to carry out site visits if there is suspicion of unreported AEs in patient case notes. Additional training will also be provided if unacceptable delay in safety reporting timelines. If any safety reports identify issues that have implications for the safety of trial participants, the PIs at all institutions participating in the trial will be notified.

10.12 Urgent Safety Measures

An urgent safety measure is a procedure not defined by the protocol, which is put in place prior to authorisation by the MHRA and REC in order to protect clinical trial participants from any immediate hazard to their health and safety.

The CTRC will notify the MHRA and REC immediately and, in any event, within 3 days that such a measure has been taken and the reasons why it has been taken. The initial notification to the MHRA will be by telephone (ideally within 24 hours) and a notice in writing will be sent within 3 days, setting out the reasons for the urgent safety measure and the plan for further action. After discussion with the MHRA and REC, further action will be agreed, which may include submission of a substantial amendment, a temporary halt, or permanent termination of the trial.

If the study is temporarily halted it may not recommence until authorised to do so by the MHRA and REC. If the study is permanently terminated before the date specified for its conclusion (in the original applications to MHRA and REC), the CTRC should notify the MHRA and REC within 15 days of the date of termination by submitting the formal End of Trial Notification.

10.13 Contact Details and Out-of-hours Medical Cover

Participants will be instructed to contact the local Investigator (their office number) during working hours. All local PIs should ensure to delegate at least one other appropriately trained (in the study and in GCP) medically qualified doctor for assessment of adverse events, safety reporting and making medical decisions in their absence.
Outside working hours the normal local procedure should be followed to contact the local site PI or their agreed delegate. PICU, ward and local switchboard staff should be aware where they should direct calls in- and out-of-hours.
The Chief Investigator has delegated two medically qualified and appropriately experienced doctors to assess safety reports in his absence (Prof Paul McNamara & Dr Kent Thorburn).
The CTRC should be the main point of contact for trial questions during opening hours – 0151 795 8757. Out-of-hours cover for trial related clinical issues that require urgent attention will be provided by the Chief Investigator or his delegates, on 07506 653 560.

11 STATISTICAL CONSIDERATIONS

11.1 Introduction

Separate Statistical Analysis Plans (SAPs) will be developed prior to the interim and final analyses of the trial.

11.2 Method of Randomisation

Participants will be randomised to receive either poractant alfa or air placebo in a 1:1 ratio. The randomisation list will be generated by an independent statistician at the CTRC who is not otherwise involved in the BESS trial. Randomisation lists will be computer-generated using block randomisation with random variable block length, stratified by site and duration of ventilation (<24hrs and >=24hrs) prior to randomisation.

11.3 Sample Size calculation

The study will be powered to detect a minimally clinically important difference of 18 hours (0.75 days) in the primary outcome variable of time of mechanical ventilation. Data are expected to be positively skewed, and will therefore be log-transformed, and resulting data back-transformed. The null hypothesis is that of no difference in time of MV between the two groups.

Data from Alder Hey Children's Hospital from an audit of 60 eligible infants admitted to PICU in the seasons 2012/3 and 2013/4 showed a mean (SD) duration of ventilation of 4.83 (2.5) days (PICANet data, 2011-14). Parameters for sample size estimation are therefore as follows:

$E(Y_1) = 4.83$ and $SD(Y_1) = 2.5$
$E(Y_2) = 4.08$ and $SD(Y_2) = 2.5$
$\delta = E(Y_2)/E(Y_1) = 0.845$

giving coefficients of variation:

$\kappa_1 = SD(Y_1)/E(Y_1) = 2.5/4.83 = 0.518$
$\kappa_2 = SD(Y_2)/E(Y_2) = 2.5/4.08 = 0.613$

Using the formula of Wolfe and Carlin (1999) with $r = 1$ for equal allocation and a two-sided alpha-level of 0.05, sample size estimations are 100 per group for 80% power and 134 per group for 90% power.

Taking the sample size of 134 and allowing for 5% dropout requires 142 patients will be recruited to each group, giving a total of 284 participants overall.

11.4 Interim Monitoring and Analyses

Analyses of the accumulating data will be performed at regular intervals (at least annually) for review by an IDSMC. These analyses will be performed at the CTRC. The IDSMC will be asked to give advice on whether the accumulated data from the trial, together with results from other relevant

trials, justifies continuing recruitment of further patients or further follow-up. The IDSMC will make recommendations to the Trial Steering Committee (TSC). A decision to discontinue recruitment, in all participants or in selected subgroups will be made only if the result is likely to convince a broad range of clinicians including participants in the trial and the general clinical community.

11.5 Analysis Plan

A full SAP will be written prior to the conduct of any comparative analysis of the treatment arms. The main features of the SAP are included here.

The primary analysis will be a test of the difference in geometric mean durations of MV between groups (placebo vs. poractant alfa), reported with the corresponding 95% confidence interval. The analysis will be conducted according to the intent-to-treat principle, and will incorporate the stratification factors of duration of ventilation (<24hrs and >=24hrs) prior to randomisation and site.

For the secondary outcomes, longitudinal data for VI, OI, OSI and SF will be analysed with joint modelling. LRSQ data will be modelled longitudinally and comparisons made between groups. Binary data will be reported in terms of relative risk with 95% confidence intervals.

Within BESS, data will be collected as to ascertain readiness for spontaneous breathing test and time of extubation.
As much information as possible will be collected about the reasons for missing outcome data; this will be used to inform any imputation approaches employed in the analysis. Such methods will be fully described in the SAP.

Deja E, *et al. BMJ Open* 2024; 14:e077023. doi: 10.1136/bmjopen-2023-077023

12 EXPLORATORY & TRANSLATIONAL WORK; WORK PACKAGES B AND C

12.1 Work Package B: Study of Parent & Staff Experiences

BESS will involve an embedded mixed method sub-study involving parents and BESS recruiters involved in recruitment and consent processes. WP-B will explore: acceptability of the trial; approach to recruitment and consent; decision making in the emergency setting; barriers to participation; and the content and format of trial information materials. This will involve: questionnaires and interviews with parents approached for informed consent in BESS, including those who do not go on to be randomised into the trial, and a survey of BESS recruiters.

Parent recruitment in season 1

In season 1, all parents approached for informed consent in BESS will be asked to complete a brief questionnaire and/or take part in a telephone interview. Recruitment to the sub study will occur shortly after the main trial recruitment discussion. This will include parents who decline to provide informed consent for the trial. If more than one parent is involved in the consent discussion, then both are able to complete a questionnaire. By completing the questionnaire, this confirms that the parent has consented to this providing information.

Parents will be asked to place completed questionnaires in a sealed stamped addressed envelope and return it to the BESS recruiter who will post to the research team. Recruitment for questionnaires will take place throughout season 1. In the rare instance that consent is not sought prior to discharge/transferred to another hospital, BESS recruiters should send the questionnaire to parents along with the Parent/Person with PISC to complete.

BESS recruiters will ask the parent to provide contact details on the BESS consent form if they wish to take part in a telephone interview. A researcher will contact families to arrange telephone interviews within one month of consent. At this point parents who live in (or close to) the Merseyside area will be provided with the option of a face to face interview at their home if they prefer. All families who express an interest in taking part but are not selected for an interview will be contacted via telephone or email to thank them for their interest in the study.

All interviews will be conducted by a researcher who both have proven skills in the conduct of research in sensitive settings. Any distress during the interviews will be managed with care and compassion and participants will be free to decline to answer any questions that they do not wish to answer or to stop the interviews at any point. Any such families will be supported in obtaining appropriate help and, after discussion with the family, the lead clinician responsible for the child's care will be informed to offer any support. Interviews will be conducted until data saturation point is achieved (approximately 15-25 based on other studies). All interview participants will be sent a £20 shopping voucher to thank them for their time. Sites will be informed when recruitment to interviews can stop.

Parent recruitment in season 2

In season 2, all parents will be asked to complete a brief questionnaire. This will be the same questionnaire as used in season 1 and include parents who decline informed consent. Questionnaire findings will help evaluate parents' views on recruitment and consent, including any changes made to our recruitment approach following season 1.

Staff recruitment in seasons 1 and 2
All staff involved in trial conduct, recruitment and consent in participating sites will be sent an email invitation and information sheet to request their participation in an online questionnaire towards the end of season 1 and 2. Completion of the questionnaire will be taken as an indication of consent.

Findings from parent questionnaires, interviews and staff surveys in season 1 will be used to develop approaches to consent, recruitment, trial information materials and staff training in season 2. Findings from season 2 parent and staff questionnaires will inform the design/training for the approach to recruitment and consent in season 3.

12.1.1 Parent and staff perspectives sub study analysis

Analysis of data from the mixed method sub study will be assisted using NVivo 8 qualitative data analysis package and SPSS software for statistical analysis. Quantitative analysis will involve simple descriptive statistics and the chi-square test for trend. Qualitative data from will be analysed thematically (32). Data from study methods will be analysed separately then synthesised (33) through the use of constant comparative analysis (34).

12.2 Work Package C:

Understanding the mechanism of efficacy or failure for exogenous surfactant to modulate bronchiolitis disease requires an integrated investigation of the subject's alveolar environment. This includes identifying the infective aetiology, describing the local inflammatory response, and defining the kinetics of surfactant lipid and protein synthesis; all linked to natural history, trial intervention and participant outcomes. The selected mechanistic sub studies leverage the unique collaboration between experts in bronchiolitis pathogenesis at Liverpool and surfactant physiology at Southampton. While the sub studies are defined individually, their outputs integrate to provide a cogent thesis.

Hypotheses to test include:
At all sites by analysis of BAL fluid sampled just prior to intervention at 0hr and 24hr:

- Surfactant concentration and composition predicts duration of MV.
- Recovery of synthesis of surfactant proteins A & D is modulated by exogenous surfactant.
- Lower airway inflammation is modulated by exogenous surfactant.[3]
- Treatment failure is associated with detection of specific pathogens.

[3] Costs for this component of BESS will not be charged to NIHR EME.

At Liverpool & Southampton by giving a single intravenous dose of deuterium-labelled[4] choline (*methyl*-D_9-choline chloride (D_9-Choline)) and with sampling of BAL fluid and blood just prior to intervention at 0hr, 12hr, 24hr and 36hr.

- Synthesis of endogenous surfactant phosphatidylcholine is modulated by the provision of exogenous surfactant.

12.2.1 Metabolic profiling of surfactant composition and kinetics

Analysis of surfactant phospholipid and protein concentration, composition and synthesis in BAL fluid from ventilated infants will help provide an explanation for the beneficial effects, or otherwise, of exogenous surfactant therapy for acute lung injury in infants.

The sub-study of the metabolic profiling of surfactant composition and kinetics has two linked parts. The first "**Surfactant concentration and composition**" utilises BAL fluid collected as part of the whole trial at any site and does not involve any additional intervention or sampling procedures. The second part "**Kinetics of surfactant phosphatidylcholine synthesis"** is a more complex study that requires additional interventions, specific consent and consequently will recruit only from Liverpool and Southampton

12.2.1.1 Surfactant concentration and composition

This will address the hypothesis that: Surfactant concentration and composition predicts duration of MV.

Infants will be recruited to this part of the mechanistic sub-study from all BESS trial centres. Individual molecular species of phosphatidylcholine (PC), phosphatidylglycerol (PG) phosphatidylinositol (PI) and sphingomyelin (SM) will be analysed in aliquots of small volume BAL fluid samples taken prior to trial intervention at 0 and 24hr hours of the study if still intubated. These phospholipids will be analysed by electrospray ionisation mass spectrometry (ESI-MS) (29, 30) in Southampton correcting for sample dilution by urea analysis(28). Surfactant proteins A, B, C, and D will be analysed by ELISA and western blotting.

12.2.1.2 Kinetics of surfactant phosphatidylcholine synthesis

This will address the hypothesis that: Synthesis of endogenous surfactant phosphatidylcholine is modulated by the provision of exogenous surfactant.

This part of the mechanistic sub-study will be conducted at Liverpool and Southampton trial sites only. Infants at these two centres will receive a single intravenous dose of D_9-choline (3.6 mg/kg body weight) prior sampling of BAL fluid and trial intervention at time = 0 hours, followed by further BAL fluid sampling at 12, 24, 36 and 48 hours if still ventilated. A single intravenous dose of deuterium-labelled choline administered prior to intervention allows assay of endogenous surfactant synthesis (31). A small blood sample (1.25ml (range 1.0 to 1.3 ml)) will also be taken at these times. D_9-choline contains the stable isotope deuterium, which is non-radioactive and non-toxic. Choline is an essential vitamin and the dose is within the daily-recommended allowance. Investigators at University of Southampton have extensive experience administering this metabolic probe in clinical

[4] Deuterium is one of the two stable (non-radioactive) and naturally occurring isotopes of hydrogen that is used as a harmless metabolic tracer in animal and human studies.

studies of: preterm infants, infants with acute lung injury (ALI), and adults with acute respiratory distress syndrome patients with no adverse effects. ESI-MS analysis of unlabelled PC and PC labelled by incorporation of D_9-choline will provide a measure of surfactant phospholipid synthesis and turnover.

12.2.2 Recovery of surfactant proteins A & D (SP-A & SP-D) is modulated by exogenous surfactant.

Presence of SP-A and SP-D in the plasma has been proposed as a biomarker for acute lung injury (32). Among children mechanically ventilated for ALI including in bronchiolitis, we have previously shown reduced levels of SP-A and SP-D in the early stages of the disease, with recovery in SP-D levels in BAL fluid linked to progress to extubation, along with normalisation of DPPC and PG levels (33).
It is thought that surfactant deficiency contributes to acute lung injury by direct disruption of type II cell function and enhanced degradation of the surfactant system. SPA is secreted along with the phospholipid components of surfactant in lamellar bodies. Thus, metabolic studies demonstrating decreased PL secretion imply decreased SP-A secretion. However, this needs to be confirmed by direct measurement from secretions in the airways in a well-characterised population sampled under controlled conditions.
We will measure SP-A and SP-D by enzyme linked immunometric assay (ELISA) and Western blot at 0hr and 24hr sampling time points from subjects at all sites and in addition at 12hr, and 36hr from subjects at Liverpool and Southampton if still ventilated. We will compare levels over time relative to baseline, group-wise according to intervention.
No previous study has had the opportunity to relate the levels of SP-A & SP-D to surfactant metabolism during the natural history of bronchiolitis disease (placebo arm) or when modified by administration of exogenous surfactant. This proposal will evaluate not only the presence or absence of SPs but also provide evidence for their role as markers of lung recovery in the BAL.

12.2.3 Lower airway inflammation is modulated by exogenous surfactant[5]

Our previous study (3 arms, 27-plex, n=301) on local airway response to RSV bronchiolitis found high levels of IL1b, IL15, IL13, MIP1b, MCP1, IL-8 & PDGF and low levels of IFNg, IP10 & RANTES associated with life-threatening bronchiolitis (all $p<0.025$) (34). Subject to other funding, these 10 markers will be measured in BAL at t=0hr and 24hr in all subjects. Comparison will be made between baseline levels and second sample in infants treated with surfactant and placebo. Correlation will be sought between the inflammatory markers and levels of surfactant lipid and proteins.

12.2.4 Treatment efficacy or failure is associated with detection of specific pathogens.

We and others have demonstrated that certain pathogens and multiple infections are associated with poor outcome (35). Using accredited diagnostic polymerase chain reaction (PCR) multiplex assays we will assay BAL sampled daily for multiple pathogens and seek any relation with pathogens detected and outcome. While RSV infection accounts for about 75% of severe cases of

[5] Costs for this component of BESS are not being charged to NIHR EME.

bronchiolitis, dual viral infection or viral infection in combination with bacterial infections can account for severe disease. The aetiology of bronchiolitis in each subject must be identified to test for any pathogen specific effect.

12.2.5 Mechanistic Protocol

12.2.5.1 Surfactant phospholipid and protein concentration and composition (all sites)

Small volume lung lavage (0.9% NaCl, 1ml/kg body weight) will be performed in all participating sites at t=0, 24 just prior to trial intervention, and separated for protein and lipid analysis. For lipid analysis, butylated hydroxytoluene (10 of 20g/l in ethanol) will be added to all samples to prevent sample oxidation. Samples will be centrifuged at 400xgx10 min and the supernatant stored in aliquots for surfactant analysis at -80ºC at each site. Other BAL fluid aliquots will be used for the inflammation and infection mechanistic subprojects. At the end of each winter season, all samples will be sent to Southampton for surfactant analysis.
Protein analysis will be conducted by standard techniques of ELISA and Western blotting. For PL analysis, samples will be lipid extracted in Southampton using organic solvents after addition of internal quantification standards and analysed by direct infusion ESI-MS. Analytes to be measured using diagnostic precursor scanning will include individual molecular species of PC, LysoPC, PG, PI and SM. Results will be expressed as % composition within an individual lipid class, as % composition of all measured lipids and as concentration (nmol/ml). Analysis of BAL fluid urea by colorimetric enzymatic assay will be used as a correction factor for BAL fluid dilution. While this in not ideal, a previous study indicates that it provides a reasonable correction for single recovery small volume lavage in ventilated infants (28).

Research Questions

Does administration of exogenous surfactant (poractant alfa) restore the dysfunctional composition of surfactant phospholipid characteristic of infants ventilated for bronchiolitis?
A power calculation from the pilot data at t=48h indicates that it will require n=49/group to achieve significance at $p<0.05$ for %DPPC of 38.8% and 43.5% respectively for infants in the placebo and treated groups. This is a realistic goal as this represents obtaining appropriate samples from 37% of prospective infants in the study
Do PC and PG concentrations in BAL fluid increase with duration of ventilation in the placebo group of infants? This analysis will also address whether the correlation between initial rate of incorporation and time to recruitment is related to secretion of labelled PC into a larger surfactant pool or decreased surfactant secretion with duration of ventilation.

12.2.5.2 Kinetics of surfactant synthesis

The mechanistic study of phosphatidylcholine surfactant synthesis will take place only at Liverpool and Southampton, which both have the appropriate infrastructure (staff and facility) to collect and process BAL more frequently. It will incorporate the phospholipid concentration and composition protocol outlined above, combined with a stable isotope labelling protocol. With specific consent recruited infants at both sites, in both placebo and treated groups, will be intravenously infused between t=0 and 1h with non-radioactive, stable isotope-labelled *methyl*D_9-choline chloride at 3.6 mg/kg. Small volume lung lavages will be collected as above, with additional time points at t=12h and 36h. EDTA blood samples (1.25ml (range 1.0 to 1.3 ml)) will be taken at the same time points and centrifuged at 400xgx10min to isolate plasma, which will be stored at -80ºC. Samples will be

couriered from Liverpool to Southampton at the end of the winter season and sample extraction and processing will follow exactly the same protocol as above. Lipid extracts from both BAL fluid and plasma will be subjected to the identical ESI-MS analytical protocol described above, with the addition of a diagnostic scan for *methyl*-D_9-labelled PC. Enrichment of methylD9-choline in plasma choline will be analysed by LC-MS/MS using a MRM protocol to provide an indication of substrate choline turnover. Urea will be analysed by colorimetric enzymatic assay in plasma as well as in BAL fluid, to enable calculation of analyte concentrations per ml of epithelial lining fluid (ELF). Results will be calculated, in addition to those described above, as enrichment (Σ abundance of labelled PC species/Σ abundance of labelled + unlabelled PC species), fractional synthetic rates and concentration of labelled species (nmol/mL ELF).

12.2.5.3 Outcomes

The major discriminators between treatment and placebo groups will be the concentration of newly synthesised and secreted *methyl*-D_9 molecular species calculated as nmoles/ml of ELF after correction for urea dilution between BAL fluid and plasma and the FSR. Power: based on our pilot data on fractional synthetic rate of surfactant in two disease states (0.039±0.005 vs 0.030±0.011) 2 tail, a=0.05 requires total n=25/group for 1-b >0.95.

Enrichment of label in molecular species of plasma PC and in plasma choline will provide comparator data to distinguish responses that are lung specific from those determined by variability in substrate availability and metabolism.

Administration of a second dose of exogenous surfactant at t=12 hours in the treated infants will permit calculation of endogenous and exogenous surfactant pool sizes by labelled surfactant dilution.

Deja E, *et al. BMJ Open* 2024; 14:e077023. doi: 10.1136/bmjopen-2023-077023

13 REGULATORY AND ETHICAL APPROVALS

13.1 Statement of Compliance

Statement of compliance: The study will be carried out in accordance with:

- The World Medical Association Declaration of Helsinki ,
- CTRC Standard Operating Procedures
- Principles of Good Clinical Practice
- The template content is structured consistent with the SPIRIT (Standard Protocol Item: Recommendations for Interventional Trials 2013)
- UK Policy Framework for Health and Social Care Research

SI /EU Regulation	Title	Main impact/scope
2001/20/EC	The EU Clinical Trials Directive	National Competent Authority<br>Ethics Framework<br>GCP legal requirement<br>Good Manufacturing Practice<br>Protocol/Amendments/safety<br>Protection of Vulnerable Groups<br>Consent / Data protection
2004/1031	Medicines for Human use Clinical Trials Regulation	Transposed EU CT Directive in UK
2005/28/EC	EU Good Clinical Practice (GCP) Directive	Investigator brochure<br>Archiving<br>Mandatory training for trial teams
2006/1928	Amends 2004/1031	Investigator brochure /essential documents<br>Serious Breach<br>Declaration of Helsinki 1996 version for CTIMP
2006/2984	Amends 2004/1031	Consent for incapacitated adult by legal representative or emergency deferred consent
2008/941	Amends 2004/1031	Blood safety and quality<br>Emergency Deferred consent for children
2009/1164	Miscellaneous Amendment	Urgent Safety measures
2009/3063	Amends 2004/1031	Nurse and pharmacists to prescribe unlicensed medicines

13.2 Regulatory Approval

This trial falls within the remit of the EU Directive 2001/20/EC, transposed into UK law as the UK Statutory Instrument 2004 No 1031: Medicines for Human Use (Clinical Trials) Regulations 2004 as amended.

BESS will be registered with the MHRA and a Clinical Trials Authorisation (CTA) will be obtained prior to the trial commencing.

13.3 Ethical Considerations

The study will abide by the principles of the World Medical Association Declaration of Helsinki.

In compliance with GCP, parents or those with parental responsibility will be fairly informed of the trial, risks, benefits, and alternatives, asked to ensure they understand how it relates to their infant, and make a voluntary decision about entry. Autonomy, privacy and welfare will be paramount, overriding the research. Parents will be kept informed of findings that might cause them to withdraw, without detriment to on-going care.
Consent in emergency setting poses particular challenges for parents. We have allowed a window of 48 hours from intubation to intervention during which to seek consent and randomisation. Dr Kerry Woolfall will lead the sub study to explore staff and parents' acceptability of our approach to consent in this challenging setting. Her work includes CONNECT study guidance, adopted by NIHR Clinical Trials Toolkit (http://www.ct-toolkit.ac.uk/routemap/informed-consent), which helps to ensure recruitment and consent to critical care trials is ethically appropriate and addresses the needs critically ill children and their families (49, 50). The trial team are experienced at conducting studies and trials in emergency settings.
The acceptability of medicines derived from pigs to families of the Jewish and Islamic faiths has been addressed with Rabbi Adler, Director of the Kashrus & Medicines Information Service; the Ashkenazi Beth Din of the United Synagogue & the Union of Orthodox Hebrew Congregations and with Dr Shafi, Chairman of the Research & Documentation Committee of the Muslim Council of Great Britain.
These religious authorities recognise that some scholars and families will have their own interpretations of their faith, as do other faiths with regards to this topic, which further complicates the issue. BESS will liaise with these authorities during the development of parent information and consent materials.

13.4 HRA Approval

BESS will come under the Health Research Authority (HRA) Approvals process as part of the initial REC submission and will follow the HRA processes for submission of amendments to REC and the HRA.

13.5 Ethical and Local Governance Approval

Prior to the trial being initiated at CTRC, a favourable ethical opinion will be obtained from a REC and global governance approval from the HRA. Prior to opening a centre to recruitment, CTRC will ensure that local governance approval has been obtained: for sites in England and Wales, this will be "Capacity & Capability" Confirmation; for sites in Scotland and Northern Ireland, this will be R&D Approval.

13.6 Protocol Deviation and Serious Breaches

Incidence of protocol non-compliance, whether reported prospectively (e.g. where a treatment cannot be administered at a scheduled time) or retrospectively noted (e.g. as a result of central monitoring) are recorded as protocol deviations, the incidence of which are monitored and reported to trial oversight committees.
A breach of the protocol or GCP is ‘serious’ if it meets the regulatory definition of being “likely to affect to a significant degree the safety or physical or mental integrity of the trial participants, or the scientific value of the trial”. All confirmed serious breaches of GCP or protocol will be reported to the MHRA and REC in an expedited manner by the CTRC on behalf of the sponsor.

If any persons involved in the conduct of the trial become aware of a potential serious breach, they must immediately report this to the CTRC who will in turn notify the Sponsor. The Sponsor will assess the breach and determine if it meets the criteria of a ‘serious’ breach of GCP or protocol and therefore requires expedited reporting to the MHRA and REC.

In determining whether or not the breach is likely to affect to a significant degree the safety, physical or mental integrity of participants, the Sponsor may seek advice from medical expert members of the Trial Management Group (TMG) and/or of the independent oversight committees (IDSMC and TSC). In determining whether or not the breach is likely to significantly affect the scientific value of the trial, the Sponsor may seek advice from the Trial Statistician. However, the Sponsor retains responsibility for the assessment of whether or not a breach meets the definition of ‘serious’ and is subject to expedited reporting to MHRA and REC.

Breaches confirmed as ‘serious’ will be reported to the MHRA and REC within 7 days by the CTRC and notified to the TMG, IDSMC and TSC at their next meetings.

Any requests for additional information from the Sponsor, TMG, TSC, IDSMC, REC or MHRA will be promptly actioned by the relevant member(s) of the research team and open communication will be maintained to ensure appropriate corrective actions are taken and documented.

Other incidences of protocol non-compliance will be recorded as protocol deviations, the frequency of which will be monitored and reported to the trial oversight committees.

13.7 Study Discontinuation

In the event that the BESS trial is discontinued, any participants currently participating will not be given their next dose of poractant alfa or placebo. Decisions on weaning and extubation will revert to standard care.

Reporting of adverse events will continue as per trial protocol.

Deja E, *et al. BMJ Open* 2024; 14:e077023. doi: 10.1136/bmjopen-2023-077023

14 DATA MANAGEMENT AND TRIAL MONITORING

Details of the monitoring to be carried out for the BESS trial are included in the BESS Trial Monitoring Plan, maintained separately to this protocol.
Trial Oversight Committees related to the monitoring of the trial are detailed in section 15.4.

14.1 Source Documents

In order to resolve possible discrepancies between information appearing in the case report form (CRF) and any other participant related documents, it is important to know what constitutes the source document and therefore the source data for all information in the CRF.

Source data is defined as: "All information in original records and certified copies of original records of clinical findings, observations, or other activities in a clinical trial necessary for the reconstruction and evaluation of the trial. Source data are contained in source documents (original records or certified copies)." (ICH E6, 1.51).

Source documents are defined as: "Original documents, data, paper and electronic records (e.g. hospital records, clinical and office charts, laboratory notes, memoranda, participant diaries or evaluation checklists, pharmacy dispensing records, recorded data from automated instruments, copies or transcriptions certified after verification as being accurate copies, microfiches, photographic negatives, microfilm or magnetic media, x-rays, participant files, and records kept at the pharmacy, at the laboratories and at medico-technical departments involved in the clinical trial)." (ICH E6, 1.52).

The CRF will be considered the source document for data where no prior record exists, and which is recorded directly in the CRF. A BESS source document list will be produced for each site.

Date(s) of conducting the informed consent process including date of provision of patient information, confirmation of full eligibility, randomisation number and the fact that the patient is participating in a clinical trial (including possible treatment arms) should be added to the patient's medical record chronologically.

14.2 Data Capture Methods

The study case report form (CRF) is the primary data collection instrument for the study. All data requested on the CRF must be recorded. All missing data must be explained.

Please ensure documents are sent by sites separate to the consent form as this has patient identifiable data on it.

The LRSQ will be completed by participants' parents at 6m and 12m after randomisation. These are completed online but paper copies will be available on request for parents without internet access. If paper questionnaires are used **sites should photocopy them** in order to retain a copy at site before mailing originals to CTRC.

BESS will also be collecting data from the Paediatric Intensive Care Audit Network (PICANet). PICANet is a secure confidential high-quality clinical database, recording details of the treatment of all critically ill children in PICUs. Collection of personally identifiable data

has been approved by the Patient Information Advisory Group (now the NHS Health Research Authority Confidentiality Advisory Group) and ethical approval granted by the Trent Medical Research Ethics Committee.
PICANet will provide a bespoke download page for the trial and sites will regularly download their own data for consented BESS patients only and transfer this to the CTRC.

14.3 Monitoring

14.3.1 Risk Assessment

This trial will undergo a risk assessment, completed in partnership between:

- Representative/s of the Trial Sponsor
- Chief Investigator
- CTRC Trial Coordinator and Supervising Trial Manager
- CTRC Trial Statistician and Supervising Statistician
- CTRC Information Systems and Data Management teams
- CTRC Director

In conducting the risk assessment, the contributors will consider potential participant, organisational and study hazards, the likelihood of their occurrence and resulting impact should they occur.

Monitoring for BESS will be informed by the trial specific risk assessment and will be conducted as per a detailed Trial Monitoring Plan (TMP), maintained separately to this protocol and stored in the TMF. The TMP will describe who will conduct the monitoring, at what frequency monitoring will be done and what level of detail monitoring will be conducted.

14.3.2 Central Monitoring

Data received at CTRC will be checked for missing or unusual values (range checks) and checked for consistency over time. Any suspect data will be returned to the site in the form of data queries. Data query forms will be produced at the CTRC from the trial database and sent either electronically or through the post to a named individual (as listed on the site delegation log). Sites will respond to the queries providing an explanation/resolution to the discrepancies and return the data query forms to CTRC. The forms will then be filed along with the appropriate CRFs and the appropriate corrections made on the database. There are a number of monitoring features in place at the CTRC to ensure reliability and validity of the trial data, to be detailed in the TMP maintained separately from this protocol.

Central checks of consent will be completed for each participant to ensure the completeness of consent and that the timing of consent is in line with the protocol.

Central monitoring will be performed in a proportionate manner as informed by the Trial Risk Assessment and Monitoring Plans.

Deja E, *et al. BMJ Open* 2024; 14:e077023. doi: 10.1136/bmjopen-2023-077023

14.3.3 Clinical Site Monitoring

In order to perform their role effectively, the Trial Coordinator, Data Manager and persons involved in Quality Assurance and Inspection may need direct access to primary data during monitoring visits, e.g. participant medical records, laboratory reports, appointment books, etc. Since this affects the participant's confidentiality, this fact is included on the PISC.
The purposes of monitoring visits, if triggered as per CTRC SOPs, include but are not limited to:

- assessing compliance with the study protocol
- discussing any emerging problems that may have been identified prior to the visit
- checking CRF and query completion practices

14.4 Confidentiality

Individual participant medical information obtained as a result of this study is considered confidential and disclosure to third parties is prohibited with the exceptions noted below.
CRFs will be pseudonymised – they will not contain details of participant names and will be labelled with the unique trial randomisation and/or screening number. Medical information may be given to the participant's medical team and all appropriate medical personnel responsible for the participant's welfare.

Verification that appropriate informed consent is obtained will be enabled by the provision of copies of participant's signed informed consent forms being supplied to the CTRC by recruiting centres, which requires that name data will be transferred to the CTRC.
The CTRC will be undertaking activities requiring the transfer of identifiable personal data. The transfer of this data is disclosed in the PISC. The CTRC will be responsible for sending links to the LRSQ to trial participants at 6m and 12m post randomisation, and therefore will be required to receive contact details including name and email address. Should the parent require a paper copy this will be sent by the recruiting site as the CTRC will not collect participant addresses.
All documentation received at CTRC which includes direct identifiers such as names and email addresses (e.g. consent forms) will be stored securely and separately to all other pseudonymised participant data (e.g. medical CRFs).
The CTRC will preserve the confidentiality of participants taking part in the study and The University of Liverpool is registered as a Data Controller with the Information Commissioners Office.

14.5 Quality Assurance (QA) and Quality Control (QC)

Quality Assurance (QA) includes all the planned and systematic actions established to ensure the trial is performed and data generated, documented, recorded and reported in compliance with applicable regulatory, ethical and governance requirements. Quality Control (QC) includes the operational techniques and activities performed within the QA system to verify that the requirements for quality of the trial-related activities are fulfilled: e.g. state what clinical site monitoring (and audit) is planned, if any. In accordance with the monitoring plan site visits will or will not be conducted and source verification performed if central monitoring processes indicate these are required. Monitoring activities could include, but are not limited to the following:

- The Trial Coordinator at the CTRC will verify appropriate approvals are in place prior to initiation of a site and the relevant personnel have attended trial specific training. A greenlight

checklist will verify all approvals are in place prior to trial initiation at CTRC and the individual site.

- The TMG will determine the minimum key staff required to be recorded on the delegation log in order for the site to be eligible to be initiated.
- Data will be evaluated for compliance with protocol and accuracy in relation to source documents.
- The study will be conducted in accordance with procedures identified in the protocol.
- Independent oversight of the trial will be provided by the Data and Safety Monitoring Committee and independent members of the Trial Steering Committee.
- The types of materials to be reviewed, who is responsible, and the schedule for reviews may be specified or referenced in other documents.
- Types and mechanisms of training of staff for the study should be specified.
- The PI and other key staff from each centre will attend site initiation training, coordinated by the CTRC, which will incorporate elements of trial-specific training necessary to fulfil the requirements of the protocol.
- The Trial Management Group will monitor screening, randomisation and consent rates between centres.
- The process for consent, recruitment and randomisation will be evaluated for compliance with the protocol.
- Data quality checks and monitoring procedures will be undertaken in line with the trial Data Management Plan.
-

14.6 Records Retention

All trial documents will be retained for a maximum period of 25 years from the End of Trial.

The site PI is responsible for archiving of all relevant source documents so that the trial data can be compared against source data after completion of the trial (e.g. in case of inspection from authorities). The PI at each trial site must make arrangements to store the essential trial documents, (as defined in Essential Documents for the Conduct of a Clinical Trial (ICH E6, Guideline for GCP)) including the Investigator Site File and Pharmacy Site File, until the CTRC informs him/her that the documents are no longer to be retained. The PI is required to ensure the continued storage of the documents, even if s/he, for example, leaves the clinic/practice or retires before the end of required storage period. Delegation must be documented in writing.

All other persons and organisations involved in the trial will be responsible for storing and archiving the parts of the TMF relevant to their delegated duties (e.g. laboratories, IMP manufacturers and distributors, third-party vendors providing randomisation and IMP allocation systems, etc.).

The CTRC undertakes to store originally completed CRFs for the same period, except for source documents pertaining to the individual investigational site, which are kept by the site PI only. The CTRC will archive the documents in compliance with the principles of GCP . All electronic CRFs and trial data will be archived onto an appropriate media for long term accessible storage. Hard copies of data will be boxed and transferred to secure premises where unique reference numbers are applied to enable confidentiality, tracking and retrieval.

Deja E, et al. BMJ Open 2024; 14:e077023. doi: 10.1136/bmjopen-2023-077023

15 INDEMNITY

BESS is sponsored by The University of Liverpool and co-ordinated by the CTRC in the University of Liverpool. The Sponsor does not hold insurance against claims for compensation for injury caused by participation in a clinical trial and they cannot offer any indemnity. As this is an investigator-initiated study, The Association of the British Pharmaceutical Industry (ABPI) guidelines for patient compensation by the pharmaceutical industry do not apply. However, in terms of liability, NHS Trust and Non-Trust Hospitals have a duty of care to patients treated, whether or not the patient is taking part in a clinical trial, and they are legally liable for the negligent acts and omission of their employees. Compensation is therefore available in the event of clinical negligence being proven.

The Sponsor does not accept liability for any breach in any other hospital's duty of care, or any negligence on the part of employees of hospitals. This applies whether the hospital is an NHS Trust or not.

The Sponsor has vicarious liability for the actions of its staff, when through the course of their employment they are involved in the design and initiation of a clinical trial, including but not limited to the authorship of the Clinical Trial Protocol. The University of Liverpool has appropriate insurance in place to cover this liability.

All participants will be recruited at NHS sites and therefore the NHS indemnity scheme/NHS professional indemnity will apply with respect to claims arising from harm to participants at site management organisations.

Clinical negligence is defined as:

"A breach of duty of care by members of the health care professions employed by NHS bodies or by others consequent on decisions or judgments made by members of those professions acting in their professional capacity in the course of their employment, and which are admitted as negligent by the employer or are determined as such through the legal process".

16 ROLES AND RESPONSIBILITIES

16.1 Role of Study Sponsor and Study Funder

The Sponsor of this trial is the University of Liverpool. The Sponsor will ensure that clear agreements are reached, documented and carried out, respecting the dignity, rights, safety and wellbeing of participants and the relationship with healthcare professionals. This will provide for proper design, management, initiation, conduct, monitoring, data collection, data analysis, data protection, financing and reporting of this trial meeting appropriate scientific, legal and regulatory standards. The responsibility for design, conduct, management, data analysis, data interpretation, manuscript writing, and dissemination of results is delegated to the Trial Management Group.

The funders of this study are the National Institute for Health Research and Medical Research Council who are providing financial funding through their Efficacy and Mechanism Evaluation Programme. The EME Programme is funded by the MRC and NIHR, with contributions from the CSO in Scotland and Health and Care Research Wales and the HSC R&D Division, Public Health Agency in Northern Ireland.

The funders will ensure there is proper use of the funds they have allocated to provide value for money. The funders assure the quality of the study, taking the lead in establishing that the research proposal is worthwhile, of high scientific quality, has an appropriate research infrastructure with expert clinical trial management, has the capacity to comply with the principles of GCP and represents good value for money.

Chiesi Farmaceutici S.p.A (Chiesi) are supplying the IMP endotracheal poractant alfa (Curosurf®) to be administered as trial IMP and will be securing use of a randomisation and re-supply system for use in the trial through an Investigator Initiated Clinical Trial Research Agreement between Chiesi and the Sponsor. Chiesi are not providing any financial funding in connection with this Agreement.

16.2 Funding and Support in Kind

Funder(s)	Financial and Non-financial Support Given
National Institute of Health Research	Financial Funding (£1,655,615.46)
Chiesi Farmaceutici S.p.A (UK address: 333 Styal Road Manchester M22 5LG)	Supply of endotracheal poractant alfa (Curosurf®), and contracting of a third party distributor who will also provide a randomisation and drug resupply system

16.3 Protocol Contributors

Name	Affiliations	Contribution to protocol
Calum Semple	Institute of Translational Medicine, University of Liverpool and Alder Hey Children's Hospital NHS Foundation Trust	Inception of study, led the writing of this protocol, clinical and scientific arrangements, trial design and conduct
Tracy Moitt	CTRC, University of Liverpool	Protocol development, governance arrangements and trial conduct
Chloe Donohue	CTRC, University of Liverpool	Protocol development, governance arrangements and trial conduct
Ashley Jones	CTRC, University of Liverpool	Led statistical arrangements, trial design and conduct
Laura Sutton	CTRC, University of Liverpool	Statistical arrangements, trial design and conduct
Kerry Woolfall	Institute of Psychology, Health & Society, University of Liverpool	Led design of parent/staff experience and consent study (WP-B)
Tony Postle	Faculty of Medicine, University of Southampton	Collaborator from inception, design and development of mechanistic study (WP-C)
Howard Clark	Faculty of Medicine, University of Southampton	Collaborator from inception, design and development of mechanistic study (WP-C)
Jens Madsen	University Hospital Southampton NHS Foundation Trust	Design and development of mechanistic study (WP-C)
John Pappachan	University Hospital Southampton NHS Foundation Trust	Design and development of mechanistic study (WP-C)
Paul Ritson	Alder Hey Children's Hospital NHS Foundation Trust	Development of BAL procedures, intervention administration/ handling, and PICU processes
Mark Turner	Institute of Translational Medicine, University of Liverpool and Liverpool Women's Hospital NHS Foundation Trust	Inception of study, clinical and scientific arrangements, trial design and liaison with pharmaceutical company
Katie Neville	CTRC, University of Liverpool	Quality Assurance review
Guido Varoli	Chiesi Farmaceutici S.p.A.	Limited to provision of information relating to IMP specifics (formulation, packaging, administration, distribution etc.)

Advice was received from all Co-Applicants to the NIHR EME funding applications and protocol submissions.

The design, conduct, data analysis, data interpretation, manuscript writing and dissemination of results are the responsibility of the TMG, subject to the approval of the University of Liverpool as Sponsor, the MHRA and National Institute for Health as Funders.
Chiesi Farmaceutici S.p.A. are responsible for supplying the IMP and subcontracting an independent third party to undertake distribution of the IMP and development, management and maintenance of a randomisation and drug resupply system.

16.4 Trial Committees

16.4.1 Trial Management Group (TMG)

A Trial Management Group (TMG) will be formed comprising the Chief Investigator, other lead investigators (clinical and non-clinical), members of the CTRC and a lay member. The TMG will be responsible for the day-to-day running and management of the trial. Refer to the TMG terms of reference and trial oversight committee membership document for further details. Meetings will be in Liverpool with teleconferencing. The TMG will report to the TSC.

16.4.2 Trial Steering Committee (TSC)

The TSC will consist of an independent medical expert chairperson (neonatology), two independent medical experts (paediatric intensive care), an independent biostatistician and two lay members (one independent, one non-independent). TMG representatives will be given the opportunity to contribute as non-independent and non-voting TSC members. The role of the TSC is to provide overall supervision for the trial and provide advice through its independent Chairman. The ultimate decision for the continuation of the trial lies with the TSC.

The TSC will first convene prior to the start of recruitment and will then define frequency of subsequent meetings (at least annually). Refer to the TSC terms of reference and trial oversight committee membership document for further details.

16.4.3 Independent Data and Safety Monitoring Committee (IDSMC)

The IDSMC consists of an independent medical expert chairperson (neonatology), an independent expert in Paediatric Intensive Care and an independent expert in medical statistics.

The IDSMC will be responsible for reviewing and assessing recruitment, interim monitoring of safety and effectiveness, trial conduct and external data. The IDSMC will first convene prior to the start of recruitment and will then define frequency of subsequent meetings (at least annually). Details of the interim analyses and monitoring are provided in section 11.4 .

The IDSMC will provide a recommendation to the TSC concerning the continuation of the study. Refer to the IDSMC charter and trial oversight committee membership document for further details.

17 PUBLICATION AND DISSEMINATION

17.1 Publication Policy

The results from different centres will be analysed together and published as soon as possible. The design, conduct, data analysis, data interpretation, manuscript writing, and dissemination of results are the responsibility of the TMG on behalf of the University of Liverpool as Sponsor and the National Institute for Health as Funders.

The TMG will form the basis of the Writing Committee and advise on the nature of publications. The Uniform Requirements for Manuscripts Submitted to Biomedical Journals (http://www.icmje.org/) will be respected. The National Clinical Trial (NCT) identifier allocated to this trial will be attached to any publications resulting from this trial.

The members of the TSC and IDSMC will be listed with their affiliations in the Acknowledgements / Appendix of the main publication.

17.1.1 Authorship

Contributors to all four of (i) the design, conduct, data analysis and interpretation, (ii) writing, (iii) manuscript approval and (iv) accountability for the integrity of the work will, depending on their contribution and journal requirements, be included by name at the manuscript head or listed at the end in a by-line as members of the BESS Consortium which will also be named at the manuscript head.

17.2 Dissemination to Key Stakeholders

BESS intends to publish open access results in high impact journals (e.g. British Medical Journal, New England Journal of Medicine, Lancet, American Journal of Respiratory and Critical Care Medicine). The full trial report will also be published in the NIHR EME Journal.
BESS will also present at international meeting of the major Paediatric Intensive Care Societies, American Thoracic Society, European Respiratory Society.

The involvement of representatives of the UK Paediatric Intensive Care Society Study Group (PICSSG) as co-applicants will be key to ensuring dissemination of result to the Paediatric Intensive Care community.

At end of study, we will organise a symposium in partnership with the UK Paediatric Intensive Care Society where the results of the study will be presented alongside reports of family experiences as reported directly by parents. The symposium will be open to intensivists, respiratory physicians, study staff and any interest participating families.
We also intend to disseminate via a PPI event to be hosted in Liverpool at end of study with targeted open invitation to all families of participants.

We anticipate that because of the nature of the study population and the disease that our findings will be of interest to the general public. For this reason we will be proactive in targeting the broadcast media and press through our professional external media consultants, with due regard to the usual NIHR publicity and publication conditions.

Deja E, *et al. BMJ Open* 2024; 14:e077023. doi: 10.1136/bmjopen-2023-077023

Public dissemination will include contemporary social media including a study website and Twitter account.

17.1 Data Sharing

Individual Clinicians must undertake not to submit any part of their individual data for publication without the prior consent of the TMG. All publications shall include a list of participating PIs and collaborators.

DISCLAIMER:

The views expressed in this publication are those of the author(s) and not necessarily those of the MRC, NHS, NIHR or the Department of Health and Social Care.

18 CHRONOLOGY OF PROTOCOL AMENDMENTS

18.1 Version 1.0 (18/07/2018)

Original signed version. Not approved by MHRA.

18.2 Version 2.0 (28/09/2018)

**Summary of Amendments from Protocol V1.0 to V2.0**		
**Page Number**	**Section**	**Amendment**
Page 1	N/A	Research Ethics reference number added as now known.
Page 14	2.0	A known hypersensitivity to the active substance or any excipients of Curosurf® added to exclusion criteria.
Page 28	6.2	A known hypersensitivity to the active substance or any excipients of Curosurf® added to exclusion criteria.
Page 32	7.3	Randomisation procedure updated to include completion of unblinding envelope
Page 34	8.1.1	Reference to physical examination added
Page 35	8.1.3	Reference to physical examination added
Page 37	8.3	Schedule for physical examinations added to intervention & follow-up schedule (Table 2)
Page 41	8.5	Safety Assessment procedures updated to include physical examinations and recording of vital signs; new sub section 8.5.1 added to detail procedure for limited physical examination
Page 50-51	9.4	Detail added to unblinding section
Page 51	9.4.1	Emergency unblinding process updated to include use of unblinding envelopes
Page 53	10.5	Reference Safety Information updated from sections 4.3 to 4.9 of the Curosurf® SmPC to section 4.8 only.
Page 77	16.1	Full NIHR EME funding statement added.
Page 82	N/A	NIHR EME disclaimer added to bottom of page.

19 REFERENCES

1. Nair H, Nokes DJ, Gessner BD, Dherani M, Madhi SA, Singleton RJ, et al. Global burden of acute lower respiratory infections due to respiratory syncytial virus in young children: a systematic review and meta-analysis. Lancet. 2010;375(9725):1545-55.
2. Green CA, Yeates D, Goldacre A, Sande C, Parslow RC, McShane P, et al. Admission to hospital for bronchiolitis in England: trends over five decades, geographical variation and association with perinatal characteristics and subsequent asthma. Arch Dis Child. 2016;101(2):140-6.
3. Jat KR, Chawla D. Surfactant therapy for bronchiolitis in critically ill infants. Cochrane Database Syst Rev. 2015(8):CD009194.
4. Luchetti M, Casiraghi G, Valsecchi R, Galassini E, Marraro G. Porcine-derived surfactant treatment of severe bronchiolitis. Acta Anaesthesiol Scand. 1998;42(7):805-10.
5. Luchetti M, Ferrero F, Gallini C, Natale A, Pigna A, Tortorolo L, et al. Multicenter, randomized, controlled study of porcine surfactant in severe respiratory syncytial virus-induced respiratory failure. Pediatr Crit Care Med. 2002;3(3):261-8.
6. Tibby SM, Hatherill M, Wright SM, Wilson P, Postle AD, Murdoch IA. Exogenous surfactant supplementation in infants with respiratory syncytial virus bronchiolitis. Am J Respir Crit Care Med. 2000;162(4 Pt 1):1251-6.
7. McNamara PS, Ritson P, Selby A*, et al* Bronchoalveolar lavage cellularity in infants with severe respiratory syncytial virus bronchiolitis. Archives of Disease in Childhood. 2003;88**:**922-926.
8. von Neergaard K. Neue Auffassungen übereinen Grundbegriff der Atemmechanik. Die Retraktionskraft der Lunge, abhängig von der Oberfl ächenspannung in den Alveolen. (New views on a fundamental concept of respiratory mechanics. The retraction force of the lung is dependent on the surface tension within the alveoli.). Z Gesamt Exp Med 1929;66:373-94.
9. Goh BC, Rynkiewicz MJ, Cafarella TR, White MR, Hartshorn KL, Allen K, et al. Molecular mechanisms of inhibition of influenza by surfactant protein D revealed by large-scale molecular dynamics simulation. Biochemistry. 2013;52(47):8527-38.
10. LeVine AM, Lotze A, Stanley S, Stroud C, O'Donnell R, Whitsett J, et al. Surfactant content in children with inflammatory lung disease. Crit Care Med. 1996;24(6):1062-7.
11. Dargaville PA, South M, McDougall PN. Surfactant abnormalities in infants with severe viral bronchiolitis. Arch Dis Child. 1996;75(2):133-6.
12. Skelton R, Holland P, Darowski M, Chetcuti PA, Morgan LW, Harwood JL. Abnormal surfactant composition and activity in severe bronchiolitis. Acta Paediatr. 1999;88(9):942-6.
13. Kerr MH, Paton JY. Surfactant protein levels in severe respiratory syncytial virus infection. Am J Respir Crit Care Med. 1999;159(4 Pt 1):1115-8.
14. Hartmann F, Fiori HH, Ramos Garcia PC, Piva J, Fiori RM. Surfactant deficiency in infants with severe acute viral bronchiolitis. J Pediatr. 2014;164(6):1432-5.
15. Kelly E, Bryan H, Possmayer F, Frndova H, Bryan C. Compliance of the respiratory system in newborn infants pre- and postsurfactant replacement therapy. Pediatr Pulmonol. 1993;15(4):225-30.

16. Jat KR, Chawla D. Surfactant therapy for bronchiolitis in critically ill infants. Cochrane Database Syst Rev. 2012(9):CD009194.
17. NICE. Bronchiolitis in children: diagnosis and management (NG9). 2015.
18. Siegel LS. Correction for prematurity and its consequences for the assessment of the very low birth weight infant. Child Dev. 1983;54(5):1176-88.
19. Soll RF. Surfactant treatment of the very preterm infant. Biol Neonate. 1998;74 Suppl 1:35-42.
20. Powell CV, McNamara P, Solis A, Shaw NJ. A parent completed questionnaire to describe the patterns of wheezing and other respiratory symptoms in infants and preschool children. Arch Dis Child. 2002;87(5):376-9.
21. Paret G, Ziv T, Barzilai A, Ben-Abraham R, Vardi A, Manisterski Y, et al. Ventilation index and outcome in children with acute respiratory distress syndrome. Pediatr Pulmonol. 1998;26(2):125-8.
22. Bilan N, Dastranji A, Ghalehgolab Behbahani A. Comparison of the spo2/fio2 ratio and the pao2/fio2 ratio in patients with acute lung injury or acute respiratory distress syndrome. J Cardiovasc Thorac Res. 2015;7(1):28-31.
23. Wolfe R, Carlin JB. Sample-Size Calculation for a Log-Transformed Outcome Measure. Controlled Clinical Trials. 1999;20(6):547-54.
24. Menon K, Ward RE, Gaboury I, Thomas M, Joffe A, Burns K, et al. Factors affecting consent in pediatric critical care research. Intensive Care Med. 2012;38(1):153-9.
25. Kerrin A, Fitch P, Errington C, Kerr D, Waxman L, Riding K, et al. Differential lower airway dendritic cell patterns may reveal distinct endotypes of RSV bronchiolitis. Thorax. 2016.
26. McNamara PS, Fonceca AM, Howarth D, Correia JB, Slupsky JR, Trinick RE, et al. Respiratory syncytial virus infection of airway epithelial cells, in vivo and in vitro, supports pulmonary antibody responses by inducing expression of the B cell differentiation factor BAFF. Thorax. 2013;68(1):76-81.
27. McNamara PS, Ritson P, Selby A, Hart CA, Smyth RL. Bronchoalveolar lavage cellularity in infants with severe respiratory syncytial virus bronchiolitis. Arch Dis Child. 2003;88(10):922-6.
28. Semple MG, Dankert HM, Ebrahimi B, Correia JB, Booth JA, Stewart JP, et al. Severe respiratory syncytial virus bronchiolitis in infants is associated with reduced airway interferon gamma and substance P. PLoS One. 2007;2(10):e1038.
29. Bernhard W, Pynn CJ, Jaworski A, Rau GA, Hohlfeld JM, Freihorst J, et al. Mass spectrometric analysis of surfactant metabolism in human volunteers using deuteriated choline. Am J Respir Crit Care Med. 2004;170(1):54-8.
30. Dushianthan A, Goss V, Cusack R, Grocott MP, Postle AD. Altered molecular specificity of surfactant phosphatidycholine synthesis in patients with acute respiratory distress syndrome. Respir Res. 2014;15:128.
31. Bunt JE, Zimmermann LJ, Wattimena JL, van Beek RH, Sauer PJ, Carnielli VP. Endogenous surfactant turnover in preterm infants measured with stable isotopes. Am J Respir Crit Care Med. 1998;157(3 Pt 1):810-4.

32. Greene KE, Wright JR, Steinberg KP, Ruzinski JT, Caldwell E, Wong WB, et al. Serial changes in surfactant-associated proteins in lung and serum before and after onset of ARDS. Am J Respir Crit Care Med. 1999;160(6):1843-50.

33. Todd DA, Marsh MJ, George A, Henderson NG, Barr H, Sebastian S, et al. Surfactant phospholipids, surfactant proteins, and inflammatory markers during acute lung injury in children. Pediatr Crit Care Med. 2010;11(1):82-91.

34. Semple MG, Flanagan B, Smyth RL. Nasal Cytokines Associated With Householder Tobacco Smoking And HRSV Bronchiolitis. American Journal of Respiratory and Critical Care Medicine; December 2012: American Thoracic Society; 2012. p. A1026-A.

35. Semple MG, Cowell A, Dove W, Greensill J, McNamara PS, Halfhide C, et al. Dual infection of infants by human metapneumovirus and human respiratory syncytial virus is strongly associated with severe bronchiolitis. J Infect Dis. 2005;191(3):382-6.

36. Wright SM, Hockey PM, Enhorning G, Strong P, Reid KB, Holgate ST, et al. Altered airway surfactant phospholipid composition and reduced lung function in asthma. J Appl Physiol (1985). 2000;89(4):1283-92.

37. Postle AD, Heeley EL, Wilton DC. A comparison of the molecular species compositions of mammalian lung surfactant phospholipids. Comp Biochem Physiol A Mol Integr Physiol. 2001;129(1):65-73.

38. Mander A, Langton-Hewer S, Bernhard W, Warner JO, Postle AD. Altered phospholipid composition and aggregate structure of lung surfactant is associated with impaired lung function in young children with respiratory infections. Am J Respir Cell Mol Biol. 2002;27(6):714-21.

39. Wright JR. Immunoregulatory functions of surfactant proteins. Nat Rev Immunol. 2005;5(1):58-68.

40. LeVine AM, Gwozdz J, Stark J, Bruno M, Whitsett J, Korfhagen T. Surfactant protein-A enhances respiratory syncytial virus clearance in vivo. J Clin Invest. 1999;103(7):1015-21.

41. Rawat M, Chadrasekharan PK, Wiliams A, Gugino S, Koenigsknecht C, Swartz D, Ma CX, Mathew B, Nair J, Lakshminrusimha S. Oxygen Saturation Index and Severity of Hypoxic Respiratory Failure. Neonatology. 2015; 107: 161-166

42. BMJ 2015;350:h2305 [doi: https://doi.org/10.1136/bmj.h2305]

20 DOCUMENTS SUPPLEMENTARY TO THE PROTOCOL

Documents referenced herein as accompanying the protocol that are separately updated and version controlled are:

Patient information sheets and consent form
GP Letter
LRSQ questionnaires
Participating sites log
Trial Monitoring Plan
Statistical Analysis Plan
Trial Risk Assessment
