# Supplementary material for: Stakeholders’ perspectives on clinical trial acceptability and approach to consent within a limited timeframe: a mixed methods study
Source: BMJ Open. 2024 Jan 2;14(1):e077023. doi: 10.1136/bmjopen-2023-077023 (PMC10773389; doi:10.1136/bmjopen-2023-077023)
Supplement: Supplementary data [file bmjopen-2023-077023supp002.pdf]

Parent Interview Topic Guide
The **B**ronchiolitis **E**ndotracheal **S**urfactant **S**tudy (BESS)

Please note: *Italic text indicates instruction for researcher and will not be read to participant*

Intro: My name is [*researcher name*] and I am a researcher from the University of Liverpool. Many thanks for agreeing to speak to us about your child's hospital admission. We know that this must have been a difficult time and the purpose of this conversation is to get your feedback so we can insure that future parents …..

Check consent for audio recording

You can stop the interview at any time. Before we start do you have any questions?

Bereaved: I have some idea about your circumstances, if there is anything that you find difficult to talk about please don't feel that you have to, or if you want to stop the interview at any point, then please let me know.

The reason that you were invited to take part in this interview study is because if we only ask those parents whose children recovered from bronchiolitis the information we gather won't be complete. The findings will be biased. We know even less about what it is like for parents who have been bereaved to have discussions with doctors and nurses about the trial and what it was like to be involved in the trial. Does that make sense? Do you have any questions before I start? I will start with some questions about you if that's ok and then I will ask you about your experience of being invited to take part in BESS?

**Section 1: Background**	
1.1	Demographics:<br>Could I please ask you to tell me a few things about yourself and your family?<br>What is your DOB?<br>What is your occupation?<br>What is the first part of post code,<br>What would you describe as being your ethnic background,<br>What is your religion (if any)?<br>How many children do you have?<br>*If not known:* Please tell me a little bit about your child (*name, age, gender*)<br>Was your child born at full term or were they premature (If prem ask how many weeks gestation when born)

	**Not bereaved**<br><br>Has he/she recovered from his/her hospital visit? Has your child (any of your children) had any previous hospital admissions for bronchiolitis?
1.2	Could you give me an outline of what happened for them to need emergency treatment for bronchiolitis? *(if bereaved say:* I know this may be difficult, if you don't wish to answer this question please say and I will go on to the next question)<br><br>When were they admitted to hospital with bronchiolitis? *Prompt: which hospital* (prompt- explore if transferred)<br>How long after you arrived at hospital was your child transferred to the PICU? How long after they arrived at hospital/ PICU where they put on a ventilator? *(Prompt: explore if also received any forms of non-invasive ventilation: e.g. nasal CPAP and nasal high low oxygen and when)*
1.3	Before your admission, had you ever heard of bronchiolitis before? At what point where you told that your child had bronchiolitis? What did you think when you first heard that your child had bronchiolitis?<br>Did anyone explain to you about this disease, such as how long a child with bronchiolitis is usually sick for? (*Prompt: If yes explore what information parents received. If no, would you have liked a doctor or nurse to have provided you with information about bronchiolitis? BOTH Yes and NO- Explore what information they would have liked to have known about*)<br>Did anyone explain how your child would be cared for? (*Prompt: Explore any discussion of treatments)*
**Section 2: BESS recruitment and consent process**	
2.1	This is a question I ask all parents and it's not a test, but just so we can gage whether the trial is being explained clearly enough I wanted to know whether you;<br><br>Could you tell me what the BESS trial was looking at?
2.2	Please could you give me an overall picture of what happened when you first heard about the BESS trial… could you tell me a bit about that?<br><br>*Explore any knowledge about the trial before admission.*<br><br>*Explore where parents were/ when they first heard the trial mentioned.*<br><br>*Explore time frame- how long where you approached after your child was put on a ventilator?*
2.3	How / where were you introduced to the study? *(prompt hospital staff or PICU transport staff)*<br><br>Was it a doctor or nurse who spoke to you about the trial?

	Did one of the nursing staff looking after your child introduce you to the research nurse or doctor?
2.4	Did the research nurse/doctor check with you that it was a good time to talk about research?<br><br>If so, when was this? Do you think that this was the best time?<br><br>If not, when would have been the best time?<br><br>How could this be improved?
2.5	Could you tell me what they explained about BESS?<br><br>Did they go through any of the potential risks or benefits of your child taking part in the trial at that point? If yes, how did they describe these?<br><br>Was there anything that you found: a) unclear b) surprising?<br><br>Is there anything else that sticks out in your mind about the discussion? *(Explore)*
2.6	Could you tell me about any written information you were given by a nurse or doctor about the trial?<br><br>Explore whether they were given the information leaflet – short version and/or the full patient information sheet<br><br>When did you receive this information? *(Prompt: explore written and verbal and time point)*<br><br>Did you read the information leaflet/sheet?<br><br>(*Prompt:* If they read the short information PIS leaflet or the full information PIS)<br><br>What did you think about the information leaflet/sheet?<br><br>Was there anything that you found: a) unclear? b) surprising?<br><br>Did you have any concerns about the nature of the treatment involved *(e.g. surfactant comes from pigs lungs) Explore any concerns related to religious beliefs. Explore if the nature of the treatment influenced decision making or willingness to provide consent.*<br><br>Could the information leaflet be improved in any way?
2.7	How long did you get to think about whether you wanted your child's information to be used in BESS? Do you think this was long enough?<br><br>Did you have the opportunity to ask any other questions about the study?<br><br>Did you ask any? *(Prompt, what questions did you ask?, if not, why not)*<br><br>How long do you think people should be given to think about taking part in BESS?
2.9	Did you give consent for your child to be in the trial?<br><br>Would you mind telling me what were your reasons for (providing consent/not providing consent)?

	In making the decision about your child's participation in BESS, what sort of things went through your mind?<br><br>Was there anything you found particularly helpful in making up your mind? Prompt: did you discuss the trial with anyone?<br><br>How hard/ easy was this decision?<br><br>Was there anything specific that you have not already mentioned that influenced your decision?<br><br>Have you / your child taken part in medical research before?
2.10	*In BESS and most research consent is obtained before children are entered into a study. In some trials conducted in emergency situations parents are asked to provide consent after their child had already been actively involved in the Trial. This was to use the data already collected and for them to continue in the trial. This is known as research without prior consent or deferred consent.*<br><br>**Have you ever heard of research without prior consent before?**<br><br>*The reason that this legislation is in place is because in situations like this (ie, in paediatric critical care), there's a belief that there's no time to have a full discussion and time to reflect about the research and that actually having that discussion might delay treatment.*<br><br>**What do you think about the use of RWPC in critical care research?**<br><br>**Do you think the BESS consent process should stay the same as you experienced, or would you suggest that we move to a RWPC approach in BESS?**<br><br>*Explore parents views on the pros and cons of each approach for BESS and reasons behind their views*
2.11	**If parent supports informed consent**: do you have any suggestions about how we could improve the consent process in the future?
2.12	If parents consented:<br><br>Where you present when the BESS syringe of liquid or air was given to your child? If yes, Could you tell me a little bit about that?<br><br>How did you feel at that point in time*? (If concerns described then explore- did you voice your concerns to a nurse or doctor)*

Interview end- thank you for your time

Inform participant that their voucher and copy of the consent form will be sent via post. Check home address is on record from registration.
