# Supplementary material for: Stakeholders’ perspectives on clinical trial acceptability and approach to consent within a limited timeframe: a mixed methods study
Source: BMJ Open. 2024 Jan 2;14(1):e077023. doi: 10.1136/bmjopen-2023-077023 (PMC10773389; doi:10.1136/bmjopen-2023-077023)
Supplement: Supplementary data [file bmjopen-2023-077023supp003.pdf]

BESS
Bronchiolitis Endotracheal Surfactant Study

BESS Study team
IPHS
Block B
1st Floor Waterhouse Building
Liverpool L69 3GL

BESS Site Staff Questionnaire

The aim of this short questionnaire is to find out how well you think BESS has been going at your site. This includes what you think is working well and perhaps what isn't working so well. We will use these findings, alongside data collected from parents to inform season two of BESS.

Please note that by returning this survey you are giving permission for your responses to be included in the BESS trial. All information will be anonymised and stored securely in compliance with the Data Protection Act 1998.

Background information

1. Please identify your hospital (ADD list)

2. What is your current role at this hospital?

   Doctor / nurse / Other (please state) ______________________________________

3. How many years' experience do you have in recruiting to paediatric clinical trials?

   _________ years

4. Are you involved in clinical care of children?

   Yes / No

5. How have you been involved in the BESS study (please tick all that apply)

   Screening patients
   Randomisation
   Consenting
   Involved in the clinical care of BESS participants
   Other (please state) ______________________________________

Training

6. Who provided your BESS training (please tick all that apply)

Trial team (e.g. including BESS trial coordinator)

Member of your site team

Other (please state) ______________________________________

7. Overall how would you rate the BESS site training **from the trial team**?

Excellent/ Good/ Fair/ Poor / Not applicable (did not attend)

8. Overall how would you rate the BESS training **from your site team**?

Excellent/ Good/ Fair/ Poor / Not applicable (did not attend)

9. Do you think the BESS site training could be improved? Yes/No

If yes, how? ____

____

Process

10. Do you think the screening process could be improved? (Please circle one answer)

Yes / No

If yes, please elaborate:

____

____

11. Do you think the randomisation process could be improved?

Yes / No

If yes, please elaborate:

____

____

12. Have you experienced any difficulties in adhering to the protocol?

Yes / No

If yes, please elaborate:

____

____

13. How acceptable did you find administering the intervention (surfactant) to patients?

Very acceptable / acceptable / not acceptable / very unacceptable / Not applicable

14. How acceptable did you find administering the placebo (sham) to patients?

Very acceptable / acceptable / not acceptable / very unacceptable / Not applicable

Consent and comments

15. How acceptable did you find seeking informed consent before randomisation in BESS?

Very acceptable / Acceptable / Not acceptable / Very unacceptable / Not applicable

Please explain your answer:
____
____

16. How have parents responded to the BESS consent discussion?

Very positively/ Positively/ Negatively/ Very negatively/ Not applicable.

Please explain your answer____
____
____

17. Did you find the case report form easy to use?

Yes / No/Not applicable

If No, Please provide and suggestions for improving the case report form
____
____

18. Did you find the consent form easy to use?

Yes / No/Not applicable

If No, Please provide and suggestions for improving the consent form
____
____

19. Please use this space to tell us anything you think is important for us to know to inform season 2 in BESS:

Thank you for taking the time to complete this questionnaire.
