# Supplementary material for: Stakeholders’ perspectives on clinical trial acceptability and approach to consent within a limited timeframe: a mixed methods study
Source: BMJ Open. 2024 Jan 2;14(1):e077023. doi: 10.1136/bmjopen-2023-077023 (PMC10773389; doi:10.1136/bmjopen-2023-077023)
Supplement: Supplementary data [file bmjopen-2023-077023supp004.pdf]

BESS
Bronchiolitis Endotracheal Surfactant Study

NHS
National Institute for Health Research

Clinician to enter Trial number (if applicable): ☐☐ ☐☐

Parent Questionnaire

The **B**ronchiolitis **E**ndotracheal **S**urfactant **S**tudy (BESS)

ONE QUESTIONNAIRE FOR EACH PARENT PRESENT

Background

- The following questions are about the BESS trial consent discussion that you took part in.
- We refer to people agreeing to take part in research as ‘consenting’.

Completing this questionnaire

Today’s date ☐☐/☐☐/☐☐☐☐

Are you the child’s ○ Mother ○ Father ○ Other *(Please specify)* ________

Your child’s date of birth __ __/ __ __/ __ __

1. *Please indicate how strongly you agree or disagree with the following statements by placing a circle around the answer that best fits your opinion*

**Statements**	**Agree**	**Neither agree nor disagree**	**Disagree**
a. The doctor or nurse checked that it was a convenient time to discuss research before discussing BESS	1	2	3
b. The information I received about BESS was clear and straightforward to understand	1	2	3
c. I had enough opportunity to ask questions about BESS	1	2	3
d. I was satisfied with the consent process for BESS	1	2	3
e. It was difficult to take in the information I was given about BESS	1	2	3
f. It was difficult to make a decision about BESS	1	2	3
g. I made this decision	1	2	3
h. Someone took this decision away from me	1	2	3
i. I was not in control of this decision	1	2	3
j. The decision about the research was inappropriately influenced by others	1	2	3

If the answer to this question is ‘Agree’, please state who you think influenced the decision about the research:

____

2. *Did you consent for your child to participate in BESS?*

◯ Yes (Go to Question 3) ◯ No (Go to Question 4)

3. *What were your reasons for providing consent for your child to participate in BESS?*

Please tick all that apply *and then circle your main reason* *(e.g. ✔)*

- a. To help my child ☐
- b. To help other children in the future ☐
- c. I felt that medical studies like BESS are important ☐
- d. Because I trusted the doctor or nurse who explained BESS ☐
- e. I didn't feel comfortable saying no to the nurse or doctor who explained the study ☐
- f. Other (Please state): ☐

  ____

4. *If you did not provide consent, please provide your reasons for deciding that your child would not take part in BESS*
(If you do not wish to do so, please leave this space blank)

5. *Please tell us any comments or suggestions you have to improve the recruitment and consent process for BESS:*

We would like to thank you for taking the time to complete this questionnaire.

Please place the questionnaire in the envelope provided, seal it and give it back to the doctor or research nurse.
