# Supplementary material for: Stakeholders’ perspectives on clinical trial acceptability and approach to consent within a limited timeframe: a mixed methods study
Source: BMJ Open. 2024 Jan 2;14(1):e077023. doi: 10.1136/bmjopen-2023-077023 (PMC10773389; doi:10.1136/bmjopen-2023-077023)
Supplement: Supplementary data [file bmjopen-2023-077023supp005.pdf]

Approach to data analysis

Phase	Description
1. Familiarising with data	ED read and re-read transcripts noting down initial ideas on themes.
2. Generating initial codes	Initially ED developed a data-coding framework using *a priori* codes identified from the project proposal, topic guilds and past relevant studies conducted by the team. Additional data-driven codes and concepts not previously captured in the initial coding frame were also added.
3. Developing the coding framework	KW coded 16% of the interview transcripts using the initial coding frame and no new themes were identified.
4. Defining and naming themes	Following review by ED and KW coding frames were subsequently developed and ordered into themes (nodes) within the *NVivo* Database.
5. Completion of coding of transcripts	ED completed coding interview transcripts in preparation for write-up.
6. Quantitative data analysis	Descriptive statistics were conducted on questionnaire and survey data.
7. Data synthesis and producing the report	ED and KW synthesised qualitative and qualitative data with reference to the adapted Theoretical Framework of Acceptability using a constant comparative approach (Deja et al 2020). This involved using themes to relate back to the study aims ensuring key findings and recommendations were relevant to the BESS trial design (i.e. catalytic validity). Final discussion and development of selected themes occurred during the write-up phase.
