# Supplementary material for: Stakeholders’ perspectives on clinical trial acceptability and approach to consent within a limited timeframe: a mixed methods study
Source: BMJ Open. 2024 Jan 2;14(1):e077023. doi: 10.1136/bmjopen-2023-077023 (PMC10773389; doi:10.1136/bmjopen-2023-077023)
Supplement: Author's manuscript [file bmjopen-2023-077023.draft_revisions.pdf]

BMJ Open is committed to open peer review. As part of this commitment we make the peer review history of every article we publish publicly available.

When an article is published we post the peer reviewers' comments and the authors' responses online. We also post the versions of the paper that were used during peer review. These are the versions that the peer review comments apply to.

The versions of the paper that follow are the versions that were submitted during the peer review process. They are not the versions of record or the final published versions. They should not be cited or distributed as the published version of this manuscript.

BMJ Open is an open access journal and the full, final, typeset and author-corrected version of record of the manuscript is available on our site with no access controls, subscription charges or pay-per-view fees (http://bmjopen.bmj.com).

If you have any questions on BMJ Open's open peer review process please email info.bmjopen@bmj.com

BMJ Open

Parents and staff perspectives on the acceptability of The Bronchiolitis Endotracheal Surfactant Study (BESS): a mixed methods embedded study

Journal:	*BMJ Open*
Manuscript ID	bmjopen-2023-077023
Article Type:	Original research
Date Submitted by the Author:	23-Jun-2023
Complete List of Authors:	Deja, Elizabeth; University of Liverpool, Department of Public Health, Policy and Systems<br>Donohue, Chloe; University of Liverpool, Liverpool Clinical Trials Centre<br>Semple, Malcolm; University of Liverpool; Alder Hey Children's Hospital, Respiratory Medicine<br>Woolfall, Kerry; University of Liverpool, Institute of Population, Health and Society<br>Investigators, BESS ; Multiple
Keywords:	QUALITATIVE RESEARCH, ETHICS (see Medical Ethics), Feasibility Studies, Paediatric intensive & critical care < INTENSIVE & CRITICAL CARE, Randomized Controlled Trial, Respiratory infections < THORACIC MEDICINE

SCHOLARONE™
Manuscripts

I, the Submitting Author has the right to grant and does grant on behalf of all authors of the Work (as defined in the below author licence), an exclusive licence and/or a non-exclusive licence for contributions from authors who are: i) UK Crown employees; ii) where BMJ has agreed a CC-BY licence shall apply, and/or iii) in accordance with the terms applicable for US Federal Government officers or employees acting as part of their official duties; on a worldwide, perpetual, irrevocable, royalty-free basis to BMJ Publishing Group Ltd ("BMJ") its licensees and where the relevant Journal is co-owned by BMJ to the co-owners of the Journal, to publish the Work in this journal and any other BMJ products and to exploit all rights, as set out in our licence.

The Submitting Author accepts and understands that any supply made under these terms is made by BMJ to the Submitting Author unless you are acting as an employee on behalf of your employer or a postgraduate student of an affiliated institution which is paying any applicable article publishing charge ("APC") for Open Access articles. Where the Submitting Author wishes to make the Work available on an Open Access basis (and intends to pay the relevant APC), the terms of reuse of such Open Access shall be governed by a Creative Commons licence – details of these licences and which Creative Commons licence will apply to this Work are set out in our licence referred to above.

Other than as permitted in any relevant BMJ Author's Self Archiving Policies, I confirm this Work has not been accepted for publication elsewhere, is not being considered for publication elsewhere and does not duplicate material already published. I confirm all authors consent to publication of this Work and authorise the granting of this licence.

For peer review only

Parents and staff perspectives on the acceptability of The Bronchiolitis Endotracheal Surfactant Study (BESS): a mixed methods embedded study

Deja, E[1], Donohue, C[2], Semple, M G[3,4], and Woolfall K[1] for the BESS Investigators

1 Corresponding author. Dr Elizabeth Deja PhD, Department of Public Health, Policy and Systems, University of Liverpool, Liverpool, UK. E.deja@Liverpool.ac.uk

2 *Liverpool Clinical Trials Centre, University of Liverpool*

3 *NIHR Health Protection Research Unit in Emerging and Zoonotic Infections, and Institute of Infection, Veterinary and Ecological Sciences, Faculty of Health and Life Sciences, University of Liverpool, Liverpool, UK*

4 *Respiratory Medicine, Alder Hey Children's Hospital, Liverpool L12 2AP, UK*

Elizabeth Deja, PhD, ORCID 0000-0002-3626-4927,
Chloe Donohue, Bsc ORCID 0000-0001-6379-8214
Malcolm G Semple PhD, ORCID 0000-0001-9700-0418
Kerry Woolfall, Phd, ORCID 0000-0002-5726-5304

Word Count:
3529

Key words

Informed Consent, Qualitative, Intensive Care Units, Bronchiolitis, Randomized Controlled Trials

Abstract

Objectives: The Bronchiolitis Endotracheal Surfactant Study (BESS) is a randomised controlled trial to determine the efficacy of endo-tracheal surfactant therapy for critically ill infants with bronchiolitis. We explored parent and staff views on trial acceptability including consent process to inform approaches to recruitment and consent in subsequent seasons and future research.

Design: A mixed-method embedded study involving a survey of site staff, alongside questionnaires and interviews with parents approached about their child's participation in BESS.

Setting: 14 Paediatric intensive care units in the UK

Participants: 54 parents of 49/119 (41%) children recruited to BESS from 13/14 (93%) of BESS sites. Of these, 39/54 (72%) completed a questionnaire, and 15/54 (27%) were interviewed. 38 staff representing all 14 sites completed a questionnaire.

Results: Parents and staff found the trial acceptable. All constructs of the Adapted Theoretical Framework of Acceptability were met. Parents viewed surfactant as being low risk and hoped their child's participation would help others in the future. Although parents supported research without prior consent in studies of time critical interventions, they believed there was sufficient time to consider this trial. Parents recommended that prospective informed consent should continue to be sought for BESS. Many felt that the time between the consent process and intervention being administered took too long and should be 'streamlined' to avoid delays in the administration of trial interventions. Staff described how the training and trial processes worked well, yet patients were missed due to lack of staff to deliver the intervention, particularly at weekends.

Conclusion: Parents and staff supported the BESS trial and highlighted aspects of the protocol, which could be refined for season 3, including a streamlined informed consent process. Findings will be useful to inform approaches to consent in future Paediatric trials where there is limited time for consent discussions.

Trial registration number: ISRCTN 11746266

For peer review only - http://bmjopen.bmj.com/site/about/guidelines.xhtml

Strengths and Limitations of this study

- Use of the Sekhon et al's theoretical framework of acceptability allowed trial acceptability to be evaluated as a multifaceted construct as opposed to a poorly defined binary (acceptable/not acceptable) approach.
- Acceptability was explored from multiple perspectives providing an in depth understating of key stakeholder views.
- Acceptability was measured at multiple time points.

- The study may have benefited from more direct feedback from parents who declined their child's involvement in BESS.

Introduction

Bronchiolitis of infancy is a seasonal respiratory viral disease that most commonly causes rhinorrhoea, mild fever, and a wet cough but in severe cases can result in feeding difficulties and respiratory distress. It is the single most common reason for hospital admission of infants (children age<1yr) (1). The youngest of these, those born prematurely and those with underlying conditions are most often and most severely affected (2). There is no vaccine or specific treatment for bronchiolitis (3). Despite advances in the provision of non-invasive modes of respiratory support, admissions to UK Paediatric Intensive Care Units (PICU) and duration of Mechanical Ventilation (MV) for life-threatening bronchiolitis have remained fairly static (1), with the exception of during the COVID pandemic which indirectly lead to a decrease in all childhood infections (4).

The Bronchiolitis Endotracheal Surfactant Study (BESS) is a phase 2, blinded randomised controlled trial designed to explore the efficacy and mechanism of surfactant therapy compared to air placebo for critically ill infants with bronchiolitis (see supplementary material for protocol). BESS is recruiting in up to 14 PICUs in UK tertiary hospitals, with the intervention mostly being administered by respiratory physiotherapists. Informed consent was sought from parents/legal representatives (referred to hereon as

For peer review only - http://bmjopen.bmj.com/site/about/guidelines.xhtml

parents) for their child's participation in BESS. Due to the time-dependent nature of the intervention, randomisation was required within 48 hours of intubation. Whilst some parents would have this full timeframe to consider trial participation, most had considerably less, depending on their time of arrival at the PICU. In designing the study we identified a need to explore parents' views on the acceptability of seeking informed consent in BESS with a view to consider an alternative approach, such as research without prior consent (5), depending on parents' views and experiences of the recruitment and consent process.

Qualitative research to incorporate staff, patient or parent perspectives in the design of a clinical trial can help ensure the trial is acceptable, appropriate and possible to conduct (6). BESS included an embedded mixed methods study in the first two seasons of the trial to explore staff and parents' perspectives on acceptability of the trial; approach to recruitment and consent; decision making in the emergency setting; barriers to participation; and the content and format of trial information materials. This paper presents the findings from the embedded study to inform ongoing recruitment to BESS and future PICU trials.

Methods

Study design

Questionnaires and interviews with parents approached for informed consent in BESS, and a survey of BESS site staff involved in trial recruitment, consent procedures and intervention administration. We used previous embedded or trial feasibility studies (7-10) to inform the design, including sample estimation, recruitment strategy, parent interview topic guide, parent questionnaire and site staff questionnaire (see supplementary material). Preliminary findings from parent interviews in season 1 and 2 (2019/2020) also informed the development the Site Staff

Questionnaire, which was conducted at the end of season 2.

Patient and Public Involvement

The BESS Protocol and in turn the Emended study had Patient and Public Involvement throughout. Six Parents of infants admitted to PICU and ventilated for life-threatening bronchiolitis and one member of the NIHR GenerationR Young Persons Advisory Group co-developed the study design and all study documents. Some of which went on to be part of the BESS Parents Advisory Group. Two members are co-applicants and sit on the Trial management Group (CF) & Trial Steering Committee (EA). Representatives of the Paediatric Intensive Care Society as stakeholders in the research output reviewed an early draft and this submitted application.

Recruitment and sampling procedure

All parents recruited to BESS were eligible to take part if they spoke English, including those who declined their child's participation in BESS. Information Power is the point at which data addresses the study aims; sample specificity (e.g., participants' experience relevant to the study aims and sample diversity); our reflexive and interpretive approach to theory and analysis (11); and sufficient quality of interview dialogue (12). Based on previous research (10, 13-16) and the BESS sample size, it was estimated that 83/160 (55%) of family's would be recruited to the questionnaire element and 15-25 parents would be interviewed to reach Information Power (12).

Parents/legal representatives

Site staff provided parents with information about the embedded study as part of the BESS Participant Information Sheet (PIS) and recruitment discussions. Staff asked parents if they would like to complete the questionnaire after the recruitment discussion and/or if they were willing to take part in a telephone interview with the BESS researcher (ED) after their child had been

For peer review only - http://bmjopen.bmj.com/site/about/guidelines.xhtml

discharged from hospital. Parents placed completed questionnaires in a stamped addressed envelope, which were collected by site staff and posted to ED.

Site staff

At the end of season 2, the BESS Trial Manager (CD) sent an email invitation to site staff requesting their participation in the online questionnaire and to cascade the link to colleagues involved in the trial. Email invitations described how completion of the questionnaire was taken as an indication of consent. Reminders were sent by (CD) and the BESS Chief Investigator (MGS) to all site key contacts after one week.

Eligibility screening and interview conduct

ED contacted parents to arrange an interview within one month of consent after checking each child's discharge and survival status with sites. Initially parents were contacted in sequential order, then purposively sampled to ensure parents from all sites and decliners to BESS were represented. Informed consent for audio recording of interviews was checked verbally at the time of the interview. The interviews explored views and experiences about: their child's admission to hospital, the BESS consent process, including how and when the trial was introduced by staff; information materials; and consent decision making. This included questions about how trial processes could be improved and the potential use and acceptability of research without prior consent in subsequent seasons. Respondent validation was used to add unanticipated topics to the topic guide as interviewing and analysis progressed (17). After the interview was complete, parents were sent a thank you letter, and a £20 Amazon voucher for their time.

Analysis

Digital audio recordings were transcribed verbatim by a professional transcription company (UK Transcription, Brighton) and anonymised. Qualitative analysis of interviews and open response questionnaire data was interpretive and iterative(18). Utilising a thematic analysis approach, the aim

For peer review only - http://bmjopen.bmj.com/site/about/guidelines.xhtml

was to provide an accurate representation of views on trial acceptability, design and processes (11, 19). NVivo 12 software (QSR International Pty Ltd., Melbourne, Australia) was used to assist in the organisation and coding of data. ED (PhD, female psychologist, Research Associate) and KW (PhD, female, social scientist, Reader) analysed and synthesised the data. Data from the parent and staff questionnaires were cleaned and entered into SPSS Version 24.0 (IBM Corp., Armonk, NY, US). Descriptive statistics are presented with percentages. To help establish trial acceptability, qualitative and quantitative findings were considered with reference to the Adapted Theoretical Framework of Acceptability (ATFA) (20, 21) (Supplementary file). The ATFA is designed to assist researchers in assessing the acceptability of healthcare interventions including paediatric clinical trials. The framework explores eight aspects of acceptability at three possible time points, before (*prospective*), during (*concurrent*) and after (*retrospective*) the intervention. Our data were a mix of *Concurrent* and *Retrospective* data (see tables 1 & 2).

RESULTS

Sample

179 families were approached to take part in BESS at the close of embedded study data collection. Of these 135/ 179 (75 %) consented to the main trial. A total of 54 parents of 49/119 (41%) children from 13/14 (93%) BESS sites took part in the embedded study (see Figure 1). Of these, 39/54 (72%) completed a questionnaire, 10/54 (19%) took part in an interview and 5/54 (10%) took part in both methods. A total of 86 parents consented to be contacted for an interview, 34/86 (40%) were invited, of these, 15/34 (44 %) were interviewed. Thematic saturation, the point where additional data does not lead to any new major themes during analysis and the researchers note high levels of 'information redundancy' during data collection (17, 22, 23), was reached after eight interviews; an additional seven interviews were conducted to ensure variance across BESS sites and achieve information power (22).

For peer review only - http://bmjopen.bmj.com/site/about/guidelines.xhtml

Parents were recruited for interviews and questionnaires from 13/ 14 (87%) open sites. Parents were interviewed a mean of 27 days (SD7.8 days) after randomisation (range: 17-43 days). Children spent on average 8 days in PICU (range 2.5- 18 days), 7 days on a ventilator (range: 2.5-17) and were a mean of 70 days old (range 15-156 days). Interviews took on average (mean) 37 minutes (SD 9.6 minutes, range 20-57).

Of the 42 (25%) family's that declined to take part in BESS, one mother completed a questionnaire. We left recruitment open for decliners to complete questionnaires for the proceeding season. Due to COVID 19 there was a two-year hiatus of the BESS study. Therefore, to gain insight into reasons for declining contest we used case report form and interview data from site staff.

39 staff at all 14 open sites BESS sites completed the online questionnaire (see figure 2). All participants apart from the site trial coordinator were involved in the clinical care of children with varied levels of experience of recruiting to paediatric clinical trials (range 0-20 years, median 4 years, 5. 84 years).

Figure 1. Parent and staff recruitment by time point [insert]

Main findings

Support for BESS although some parents found the intervention procedure uncomfortable to watch

As shown in Table 2, staff supported the trial, they had a clear understating of the aim of the study and how to follow the protocol. They viewed the administration of the intervention as being acceptable and ethical with free text comments highlighting how the trial had been: *"well received by PICU staff and parents"* (P31, Research nurse). Indeed, parents described their support for the trial. Many appeared to view BESS as an extension of standard care as the physiotherapy and tests conducted may have occurred anyway at different times in their child's clinical care. Parents were

For peer review only - http://bmjopen.bmj.com/site/about/guidelines.xhtml

reassured by information about how surfactant was used in premature babies with very few side effects:

> *"I asked if there would be any side effects and things like that, and she said because it wasn't a new drug, she said there haven't been on the ones they had used it on previously, that they used it on premature babies."* P04, mother, Interview

Participation was not considered to place additional burden on the child, although some parents found physiotherapy uncomfortable to watch and chose to leave the room:

> "*A lot of his physios, I was watching one where they'd, like, shake your baby. (Laughter) Then they push down on their ribs like they compress the ribs. I'm sure that that's what they were doing before they were going to possibly give the placebo or the surfactant. … It's frightening to me, so I avoided it like the plague when they were doing that; I left… Because I thought, they don't need a panicky mum around them, so anything like that I just avoided it*" P14, mother, interview

Consent decisions were informed despite the highly emotive and stressful situation

Early in the interview, parents were asked to explain their understanding of the BESS Study. Whilst the depth of descriptions varied, all gave a clear and accurate account of the purpose of the trial. Many outlined primary and secondary outcomes, including how surfactant might: "*reduce the amount of time on a ventilator"* (P12, mother, interview), prevent: "*lungs from sticking*" (P09, Father), helping them: "*to expand and open their lungs*" (P02, mother, interview) and hopefully help "*them recover quicker*" (P10, father, interview) therefore reducing parental and child distress, cost, ventilator related infections.

All interview and questionnaire participants stated that were given sufficient time to consider the information and ask questions and that staff had broached BESS at '*an appropriate time*' (100%, questionnaire item) (see Tables 1 and 2). Parents described being able to make an informed consent decision about the trial, despite the highly emotive and stressful situation:

For peer review only - http://bmjopen.bmj.com/site/about/guidelines.xhtml

> "*I guess the main elephant in the room is, do I feel as if, because I was in a stressful situation, an emotional situation, where I wasn't in my right mind, where I'm having to deal with a lot of different, very scary, very emotional, very worrying situations, was I making clear and conscious decisions? The answer is yes, I was*" P13, father, interview

A total of 19/179 (10%) declined their child's participation in the BESS study. Reasons given for not taking part included situational capacity as there was: "*too much already going on*." (Mother, Questionnaire, decliner) and: '*do not want to take part in research*' (case report form). Others declined as their child was close to coming off the ventilator at the time of final consent. A minority of parents '*did not want their child to receive surfactant*' (case report form), raising concerns with staff about the potential risks of: *"using something unproven when their child was sick"* (P28, Physiotherapist). However, most: *"Families were much more open to surfactant than I expected*." (P19, Research nurse) "*especially if their child had been premature and had surfactant in the neonatal period*." (P10, Physiotherapist). One nurse noted that the number of decliners were similar to other PICU trials: *"Few exceptions where they didn't want child as a guinea pig, but no more than usual"* (P6, nurse).

The need to 'streamline' the consent process to enable quicker intervention delivery

Both groups highlighted that sometimes there were relatively long stretches of time between initial trial recruitment discussions and trial interventions beginning. The length of time was dependent on the availability of research nurses (who largely work 9am-5pm on weekdays) and parents for the consent discussions and availability of physiotherapists to administer the intervention: "*delaying the whole process*" (P15, Research nurse):

> "I think the only thing that could have possibly done better was doing it sooner because, she went in at midnight. Then, we did hear about it the next morning, then we'd missed the nurse at that point for a whole day. So, potentially, it could have started a day earlier. The actual time between them approaching me, to me to find out what it is, to me to say yes and

then actually starting the trial, I feel that could have been really streamlined" P13, father, interview

"Low on personnel on the ground as medics are undertaking the intervention which takes considerable time in a busy unit" P06, Doctor

In addition to increasing the: *"potential for patients to begin weaning from the ventilator and therefore not be eligible"* (P23, Research nurse). During interviews, parents questioned the ethicality and impact of delaying a potentially effective treatment they had consented to:

"If there was someone on hand to make it happen despite the shift change [...] because you want the test to actually be effective if you're going to take part, don't you? You don't want to waste time." P11, father, interview

Parents provided practical suggestions for speeding up the recruitment and consent process, for example having the written information available at the bedside so that they would likely have read it before the first approach allowing for those comfortable to consent without a follow up discussion. Therefore, removing a step in the recruitment process timeline. Additionally, they highlighted the need to have more staff available that can take consent and administer the intervention.

Research without prior consent is appropriate for some PICU trials, but not for BESS

Figure 2 Description of RWPC read to participants during interview [Insert]

Towards the end of the interviews, a definition of research without prior consent (RWPC) was read to parents (Figure 2). All parents responded positively to the use of RWPC in critical care research in emergency situations for trials that were regarded as being low risk to their child's wellbeing.

"I suppose as long as it's something that doesn't have any risks associated with it, or there wouldn't be a reason why you wouldn't want them to do it and it's something that's going to help them, then I think it's (RWPC) a good thing." P08, Mother, interview

Five parents described consenting to another trial that had used a RWPC approach during the same hospital admittance. Nevertheless, the majority (n = 11/ 15, 74%) of parents interviewed suggested that BESS should continue to use an informed consent process as the intervention was not viewed to be time critical in that surfactant was regarded as something to hopefully get children "*off the ventilator quicker" rather than an emergency intervention that would be lifesaving "If we don't give this to her immediately"* (P08, mother, interview). In contrast, three parents were in favour of RWPC in the BESS study and there were no strong objections against its possible use, as the intervention was viewed to be low risk, would facilitate the intervention been given more quickly, thus avoiding missed patients and increasing recruitment rates. As the following quote illustrates:

"There's something that you think could help him and there's no risk, why wouldn't you just go for it and then tell me afterwards that*, "We've done it, and it is a trial, but that we're hopeful that it will do some good,"* and, actually, it is an emergency, isn't it? As much as it's, you know, you have got a bit more time to have the conversations, if you'd had just cracked on and done it, it could've made a difference quicker, but by the time we got around to the actual consent, he was being extubated" P05, mother, interview

For peer review only - http://bmjopen.bmj.com/site/about/guidelines.xhtml

Table 1. Parent concordant acceptability of BESS mapped to the adapted framework of acceptability Deja E. et al., 2021

Affective attitude	Burden	Ethicality	Intervention coherence	Opportunity costs	Self-efficacy	Perceived effectiveness	Trust
135/ 179 (75 %) consented for their child to take part in BESS.	50/60 (83%) did not find it difficult to take in information about BESS<br><br>47/60 (78%) did not find it difficult to make a decision about BESS<br><br>“We felt the process was straightforward” MQ34	60/60 (100%) agreed that they were satisfied with the consent process<br><br>55/60 (92%) felt that medical studies like BESS are important	60/ 60 (100%) agreed that the Information received about BESS was clear and straightforward to understand	No relevant data was reported at this time point	67/60 (95%) felt that they made the decision for their child to take part in the pilot trial.<br><br>54/60 (90%) felt in control of the decision to take part.<br><br>“medical team made it clear that this decision was mine fully and I could withdraw consent at any time. This knowledge made participating much easer.’ MQ45	53/50 (88.3%) Selected ‘helping my child’ as a reason for taking part<br><br>57/60= 95% selected ‘to help other children in the future’ as a reason for taking part<br><br>“I think all children would benefit from this great work thanks” FQ42	45/60 (75%) selected ‘because I trusted the doctor or nurse who explained BESS’ as a reason for taking part. *<br><br>“Trusting the doctor or nurse explaining BESS to parents is going to be an important deciding factor subconsciously for most parents” FQ30
9/179 declined to take part because they did not want their child to receive porcine surfactant<br><br>19/179 Do not want to take part in research	“My partner & I decided it was two much to take on when our son is so sick. too Much already going on.” MQ53- decliner’						

* note, not selecting this *did not mean* that they did not trust the staff it was just not a identified reason for their consent.

Key: MQ mother open questionnaire response, MF: father open questionnaire response. MI: mother interview, FI Father interview

For peer review only - http://bmjopen.bmj.com/site/about/guidelines.xhtml

Table 2. Retrospective acceptability of BESS mapped to the adapted framework of acceptability Deja. E et al., 2021

Affective attitude	Burden	Ethicality	Intervention coherence	Opportunity costs	Self-efficacy	Perceived effectiveness	Trust
Parental Acceptability							
Happy with trial “obviously I was approached for the BESS trial, which I was quite happy for him to take part in.”MI02	‘But the standard of care for the placebo is just normal care anyway’ MI06	‘You feel powerless, to be entered into a research study without somebody talk to you about it you’ve got even more power taken away from you.’ MI12<br>“you want the test to actually be effective if you’re going to take part, don’t you? You don’t want to waste time.” FI11	“If they can do a trial that shows that actually giving the surfactant or not brings them out of it quicker” MI04<br><br>‘literally any question I had in my head, this leaflet then had contained the answers” MI05	“there are not really risks, so there’s not really any problem, all it could possibly do is produce positives. FI13	“She was so pleasant and she was so easy to talk to. Even when I wasn't discussing the paperwork and the trial with her, if I had any questions, I could easily walk up and ask, if I had any enquiries. She was very, very easy to approach.” MI02	“then it was a realisation of, oh, my God, so you’ve just told me this thing that could make this difference and could get him off the ventilator quicker, and he might not get it anyway’ MI05	‘I don’t think they’d put your babies in danger. They are here to get them better, so you just kind of trust in what they are doing.” MI4
Staff Acceptability							
Overall how acceptable do you find the BESS study? 20/36 (53%) very acceptable 18/36 (47%) acceptable<br><br>“If it will have a clinical benefit which outweighs potential risk, then it is definitely acceptable” (P14)	*“Low on personnel on the ground as medics are undertaking the intervention which takes considerable time in a busy unit”* (P06, Doctor)	10/36 (26%) found it very acceptable and 28/36 (74%) acceptable to administer the surfactant.<br><br>6 (16) found it very acceptable and 31(82) found it acceptable to administer the placebo	The majority of staff rated the training as ‘excellent’ (27/36, 75%) or ‘good’ (9/36, 25%).	*“we tried to time other interventions that required muscle relaxants e.g. ETT tape changes with the time the placebo needed to be given”* (P39, Physiotherapist).	(34/36, 87%) staff indicated that they had not experienced difficulties adhering to the BESS protocol.	“I think it's important that we look at ways to improve care and treatment so research trials are vital. Reducing ventilator days would help free up precious ITU beds during winter and hopefully reduce problems with lung injuries” (P17)	Not mentioned in questionnaire responses

For peer review only - http://bmjopen.bmj.com/site/about/guidelines.xhtml

Discussion

The aim of this study was to explore the acceptability of the BESS Study to help inform recruitment in subsequent bronchiolitis seasons and other PICU trials. The Adapted Theoretical Framework of Acceptability (20) allowed us to explore the multifaceted construct of acceptability, with eight components to consider. Our data suggest that the BESS Study met all eight of the constructs. BESS was viewed by parents and PICU staff as understandable, posing low to no risk and possible benefits to child participants.

Our findings suggest that both parents and staff viewed the BESS study ethical and potentially beneficial to children with bronchiolitis. However, they highlighted that the length of the consent process and staff availability impacted on the timeliness of administering the BESS intervention. They were concerned that such delays may reduce the potential effectiveness of the intervention. At the study design stage there was uncertainty about whether it was more appropriate to use RWPC or an informed consent approach to recruitment in BESS due to the intensive care content and need to administer the intervention in a limited period. However, our findings support the continued use of informed consent in this context, though RWPC was supported by a minority of parents as it would reduce delay of intervention. BESS interventions need to be given promptly with a short window for trial recruitment discussions with parents. Its low-risk nature means that parents felt they could make an informed decision after one conversation when the child is stable. This with other suggested changes to study processes should allow the full intervention to be administered in a timelier manner.

The seasonal nature of bronchiolitis provided an opportunity to review and adapt trial processes, increasing opportunities to make the trial more acceptable acceptability and feasibility to conduct. However, most trials do not have this ability leading to potential research and resource waste (24). Our findings highlight the importance of pre-trial research or embedded studies in which the potential time windows between randomisation and intervention delivery can be clearly mapped and parent views on the most appropriate consent approach are fully explored.

For peer review only - http://bmjopen.bmj.com/site/about/guidelines.xhtml

The main strength of this study is that acceptability was measured at multiple time points, from different perspectives providing an in depth understating of key stakeholder views. The study may have benefited from more direct feedback from parents who declined their child's involvement in BESS (25). We kept recruitment open for decliners for an additional season to try to address this gap in knowledge. However, their wish to 'not take part in research' and 'situational incapacity' in BESS, also applied to the embedded study and was compounded by low recruitment in season three as a consequence of COVID (4). We believe that the data from staff, the case report form and the one decliner questioner gave us an insight into reasons for declining consent. In addition, the overall number of decliners was low. Therefore, this missing data should not have a large impact on our findings.

Conclusion

Our paper found BESS to be feasible and acceptable, supported the continued use of informed consent and highlighting areas for study process improvements. Future trials should consider proportionate consent processes when interventions are low risk and there is a short window for informed consent discussions.

For peer review only - http://bmjopen.bmj.com/site/about/guidelines.xhtml

Acknowledgments

We firstly thank the children and families who participated in the BESS without your help this research could not have taken place. We also thank the principal investigators, respiratory physiotherapists and research nurses at each study site.

We would also like to thank the National Institute for Health Research's Efficacy and Mechanism Evaluation (EME) Programme (ref: 12/205/28) for funding this research.

Author contributions

MGS Chief Investigator; lead on the conception and design of the BESS study, KW designed and led the embedded study. CD facilitated the acquisition of data for analysis and trail management. ED conducted the interviews. ED and KW analysed and interpreted the data. ED and KW drafted the manuscript. ED, KW, MGS, CD and the BESS Investigators reviewed the manuscript critically for important intellectual content and provided approval of the version to be published. BESS investigators other contributions can be seen in the supplementary material.

BESS Investigators

Chief Investigator: Malcolm G Semple PhD (Department of Clinical Infection, Microbiology and Immunology, Institute of Infection, Veterinary and Ecological Sciences, University of Liverpool and Department of Respiratory Medicine, Alder Hey Children's NHS Foundation Trust, Liverpool)

Deputy Chief Investigator: Paul S McNamara PhD, (Department of Women's and Children's Health, Institute of Life Course and Medical Sciences, University of Liverpool and Department of Respiratory Medicine, Alder Hey Children's NHS Foundation Trust, Liverpool)

Coinvestigators: Evette Allen PhD, Clare Fowler HNC (Parent Lay Representatives), Catrin Barker PGDipClinPharm, Kentigern Thorburn MD, Matthew Peak PhD, Paul C Ritson MSc, Clare van Miert PhD, (Alder Hey Children's NHS Foundation Trust, Liverpool), Ashley Best MSc, Chloe Donohue BSc, Ashley Jones PhD, Tracy Moitt, Laura Price BSc, Paula Williamson PhD (Liverpool Clinical Trials Centre, University of Liverpool, Liverpool), Howard W Clark PhD, Jens Madsen PhD (Elizabeth Garrett Anderson Institute for Women's Health, University College London, London), Anne Dawson, Colin Summers (Faculty of Health and Life Sciences, University of Liverpool, Liverpool), Elizabeth Deja PhD, Kerry Woolfall PhD (Institute of Population Health, University of Liverpool), Blessing Osaghae PhD, Mark Turner PhD (Department of Women's and Children's Health, Institute of Life Course and Medical Sciences, University of Liverpool), Madhuri Panchal BSc, Anthony Postle PhD, (National Institute for Health Research Southampton Biomedical Research Centre Clinical and Experimental Sciences, Faculty of Medicine, University of Southampton, Southampton), John Pappachan MBBChir, (Southampton Children's Hospital, Southampton), Roger Parslow PhD (Division of Epidemiology and Biostatistics, Leeds Institute of Cardiovascular and Metabolic Medicine, University of Leeds, Leeds), Jennifer Preston BA, (NIHR Alder Hey Clinical Research Facility, University of Liverpool, Liverpool)

Site Investigators:

For peer review only - http://bmjopen.bmj.com/site/about/guidelines.xhtml

Alder Hey Children's Hospital, Liverpool: Kentigern Thorburn MD (Principal Investigator), Vanessa Compton, Peter Jirasek, Dawn Jones, Michael Mander, Laura O'Malley, Laura Rad, Laura Rimmer, Paul C Ritson MSc, Chris Simons.

Birmingham Children's Hospital, Birmingham: Afeda Mohamed Ali MBBCh, Kevin P Morris MD (Principal Investigators), Cara Alexander BSc, Wendy Browne MSc, Hannah Child BSc, Stephanie Clarke BSc, Sarah Fox BN, Natalie Milburn BSc, Philip Milner FIBS, Samantha Owen BSc, Holly Parkin BSc, Harriet Payne BSc, Carly Tooke, Helen Winmill MSc.

Bristol Royal Hospital for Children, Bristol: Peter J Davis MBChB (Principal Investigator), Katherine Baptiste, Sophie Coles BSc, Sarah-Jayne Eames BSc, Christina Linton BSc, Helen Marley BSc, Sarah Mogan BSc, Alvin Schadenberg PhD, John Stiven BSc.

Great North Children's Hospital, Newcastle: Rachel Agbeko PhD (Principal Investigator), Rob Claydon PgDip, Christine Mackerness, Anna Stancombe BSc, Kate Teeley BSc, Grace Williamson MD.

Leeds Children's Hospital, Leeds: Santosh Sundararajan MD (Principal Investigator), Ramesh Kumar, Kathryn Reeves BSc, Emily Scriven.

Leicester Children's Hospital, Leicester: Raghu N Ramaiah MBBS (Principal Investigator), Rekha Patel.

Nottingham Children's Hospital, Nottingham: Patrick E Davies BMBS (Principal Investigator), Lindsay Crate, Simon Gates

Oxford Children's Hospital, Oxford: James Weitz MBBS (Principal Investigator), Kirsten Beadon BSc, Rachel McMinnis BSc, Zoe Oliver BSc, Avishay Sarfatti BMBCh, Frances Sinfield, Charlotte Thompson.

Royal Belfast Hospital for Sick Children, Belfast: Julie Richardson MBChB (Principal Investigator), Hilary Callaghan BSc, Vicki Linton BSc, Jeremy Lyons MD, Clara Nelson BSc, Mark Terris MBChB

Royal Hospital for Children and Young People, Edinburgh: Tsz-Yan Milly Lo PhD (Principal Investigator), David Armstrong MD, Steve Cunningham PhD, Jackie McCormick , Andrea Wood MSc.

Royal Hospital for Children, Glasgow: Richard Levin MBChB, (Principal Investigator), Edgar Brincat MD, Christopher Lamb RN, Ross Marscheider.

Royal Manchester Children's Hospital, Manchester: Stephen D Playfor DM (Principal Investigator), Bernadette C Gavin MSc, Dave J Morgan MSc, Lara T Bunni MSc, Claire F Jennings MClinRes, Rebecca Marshall DipHE(Nur), Emma K Riley MNurSci.

Southampton Children's Hospital, Southampton: John Pappachan MBBChir, Ahmed Osman MB ChB (Principal Investigators), Lorena Caruana, Hannah Clarke, Amber Cook BSc, Tracey Curtis, Nichola Etherington, Michael Griksaitis, Jenni McCorkell BSc, Christie Mellish, Simone Paulson BMBCh, Jenny Pond BSc, Catherine Postlethwaite DipPaedNur.

Staffordshire Children's Hospital, Stoke: Constantinos Kanaris PhD (Principal Investigator), John Alexander MBChB, Nicola McClelland, Holly Minchin, Helen Parker, Pavanasam Ramesh, Joanne Tomlinson.

The Royal London Children's Hospital, London: Simona Lampariello MD (Principal Investigator), Bessie Cipriano, Nosheen Khalid MSc, Ramiya Kirupananthan MBBS, Craig Knott MBBS, Tara Murray, Olivia Nugent, Nicolene Plaatjies, Samantha Reed, Natasha Roberts, Christa Ronan, Salman Siddiqi MB BS, Natasha Thorn BMBS

Funding

This work was supported by the National Institute for Health Research's Efficacy and Mechanism Evaluation (EME) Programme (ref: 12/205/28).

Conflicts of Interests

MGS**:** Independent external and non-remunerated member of Pfizer's External Data Monitoring Committee for their mRNA vaccine program(s); HMG UK Scientific Advisory Group for Emergencies (SAGE), COVID-19 Response, Non-remunerated independent member March 2020 to March 2022. HMG UK New Emerging Respiratory Virus Threats Advisory Group (NERVTAG), Non-remunerated independent member since 2014; Integrum Scientific LLC, Greensboro, NC, USA, Chair of Infectious Disease Scientific Advisory Board; MedEx Solutions Ltd,Director; Integrum Scientific LLC, Greensboro, NC, USA, Minority Owner: MedEx Solutions Ltd, Majority Owner.

Ethics Statement

Ethical approval for the study was given by the South Central Berkshire Research Ethics Committee (IRAS ID 220853).

Data sharing statement

No data are available. The datasets generated during and/or analysed during the current study are not publicly available as consent was not sought for data sharing**.**

Declaration.

This manuscript has been read and approved by all the authors, the requirements for authorship have been met, and each author believes that the manuscript represents honest work

For peer review only - http://bmjopen.bmj.com/site/about/guidelines.xhtml

References

1. Green CA, Yeates D, Goldacre A, Sande C, Parslow RC, McShane P, et al. Admission to hospital for bronchiolitis in England: trends over five decades, geographical variation and association with perinatal characteristics and subsequent asthma. Archives of disease in childhood. 2016;101(2):140-6.
2. Auroux B, Hascoët J, Jarreau P, Magny J, Rozé J, Saliba E, et al. Risk factors for bronchiolitis hospitalization in infants: A French nationwide retrospective cohort study over four consecutive seasons (2009-2013). PLOS ONE. 2020;15(3).
3. Bronchiolitis N. Diagnosis and Management of Bronchiolitis in Children, Clinical Guideline NG9. National Institute for Clinical Excellence (NICE). 2015.
4. Kadambari S, Goldacre R, Morris E, Goldacre MJ, Pollard AJ. Indirect effects of the covid-19 pandemic on childhood infection in England: population based observational study. bmj. 2022;376.
5. Woolfall K, Frith L, Dawson A, Gamble C, Lyttle MD, Young B. Fifteen-minute consultation: an evidence-based approach to research without prior consent (deferred consent) in neonatal and paediatric critical care trials. Arch Dis Child Educ Pract Ed. 2016;101(1):49-53.
6. O'Cathain A, Hoddinott P, Lewin S, Thomas KJ, Young B, Adamson J, et al. Maximising the impact of qualitative research in feasibility studies for randomised controlled trials: guidance for researchers. Pilot and Feasibility Studies. 2015;1(1):1-13.
7. Molyneux S, Njue M, Boga M, Akello L, Olupot-Olupot P, Engoru C, et al. 'The Words Will Pass with the Blowing Wind': Staff and Parent Views of the Deferred Consent Process, with Prior Assent, Used in an Emergency Fluids Trial in Two African Hospitals. PLoS ONE. 2013;8(2):e54894.
8. O'Hara CB, Canter RR, Mouncey PR, Carter A, Jones N, Nadel S, et al. A qualitative feasibility study to inform a randomised controlled trial of fluid bolus therapy in septic shock. Archives of Disease in Childhood. 2017:archdischild-2016-312515.
9. Woolfall K, Frith L, Gamble C, Gilbert R, Mok Q, Young B. How parents and practitioners experience research without prior consent (deferred consent) for emergency research involving children with life threatening conditions: a mixed method study. BMJ Open. 2015;5(9):e008522.
10. Peters MJ, Woolfall K, Khan I, Deja E, Mouncey PR, Wulff J, et al. Different temperature thresholds for antipyretic intervention in critically ill children with fever due to infection: the FEVER feasibility RCT. Health Technology Assessment 2019;23(5):1 - 148.
11. Byrne D. A worked example of Braun and Clarke's approach to reflexive thematic analysis. Quality & Quantity. 2021.
12. Malterud K, Siersma V, Guassora A. Sample Size in Qualitative Interview Studies. Qualitative Health Research. Sage Journals. 2016;26(13):1753-60.
13. Woolfall K, Frith L, Gamble C, Gilbert R, Quen M, The CONNECT advisory group, et al. How parents and practitioners experience research without prior consent (deferred consent) for emergency research involving children with life threatening conditions: a mixed method study. BMJ Open *2015*;5(9):e008522
14. O'Hara CB, Canter RR, Mouncey PR, Carter A, Jones N, Nadel S, et al. A qualitative feasibility study to inform a randomised controlled trial of fluid bolus therapy in septic shock. Archives of Disease in Childhood. 2017.
15. Tume LN, Woolfall K, Arch B, Roper L, Deja E, Jones AP, et al. Routine gastric residual volume measurement to guide enteral feeding in mechanically ventilated infants and children: the GASTRIC feasibility study. Health Technology Assessment (Winchester, England). 2020;24(23):1.
16. Deja E, Roper L, Tume LN, Dorling J, Gale C, Arch B, et al. Can they stomach it? Parent and practitioner acceptability of a trial comparing gastric residual volume measurement versus no gastric residual volume in UK NNU and PICUs: a feasibility study. Pilot and feasibility studies. 2021;7(1):1-9.
17. Mays N, Pope C. Assessing quality in qualitative research. BMJ. 2000;320(7226):50-2.
18. Braun V, Clarke V. What can "thematic analysis''offer health and wellbeing researchers? International journal of qualitative studies on health and well-being. 2014;9:26152.

For peer review only - http://bmjopen.bmj.com/site/about/guidelines.xhtml

19. Byrne D. A worked example of Braun and Clarke's approach to reflexive thematic analysis. Quality & Quantity. 2021:1-22.
20. Deja E, Peters MJ, Khan I, Mouncey PR, Agbeko R, Fenn B, et al. Establishing and augmenting views on the acceptability of a paediatric critical care randomised controlled trial (the FEVER trial): a mixed methods study. BMJ Open. 2021;11(3):e041952.
21. Sekhon M, Cartwright M, Francis JJ. Acceptability of health care interventions: A theoretical framework and proposed research agenda. British Journal of Health Psychology. 2018;23(3):519-31.
22. Saunders B, Sim J, Kingstone T, Baker S, Waterfield J, Bartlam B, et al. Saturation in qualitative research: exploring its conceptualization and operationalization. Quality & Quantity. 2018;52(4):1893-907.
23. Baker SE, Edwards R. How many qualitative interviews is enough? National Centre for Research Methods (NCRM): . 2012.
24. Morgan B, Hejdenberg J, Hinrichs-Krapels S, Armstrong D. Do feasibility studies contribute to, or avoid, waste in research? PloS one. 2018;13(4):e0195951.
25. Hughes-Morley A, Young B, Hempel RJ, Russell IT, Waheed W, Bower P. What can we learn from trial decliners about improving recruitment? Qualitative study. Trials. 2016;17(1):1-13.

For peer review only - http://bmjopen.bmj.com/site/about/guidelines.xhtml

Figure Legends

Figure 1. Parent and staff recruitment by time point

Figure 2 Description of RWPC read to participants during interview

Table 1. Parent concordant acceptability of BESS mapped to the adapted framework of acceptability Deja E. et al., 2021

Table 2. Retrospective acceptability of BESS mapped to the adapted framework of acceptability Deja. E et al., 2021

For peer review only - http://bmjopen.bmj.com/site/about/guidelines.xhtml

Staff Questionnaire recruitment

Retrospective

Completed questionnaire: n= 39
Doctors 14, 36%
Nurses 1, 3%
Research nurses 9, 23%
Physiotherapist 12, 31%

Parent Interview recruitment S1 & 2

Retrospective

Season One
Consented to be contacted for interview: n=4/7, 57%
Contacted for interview: n = 4/4, 100%
No response to contact: n=1/4, 25%
Agreed to interview: n = 3/4, 75%
No response at interview: n = 2/4, 50%

Season Two
Consented to be contacted for interview: n= 62/94, 65%
Contacted for interview: n = 26/62, 41%
No response to contact: n = 5/26, 19%
Declined to be interviewed = 1/26, 4%
Agreed to interview: n = 20/26, 78%
No response at interview: n = 6/20, 30%
N/A due to data saturation: n= 36/62, 58%

Interviewed
Mothers: n = 9/15, 60%
Fathers n = 6/15, 40%

Staff Questionnaire recruitment

Retrospective

Completed questionnaire: n= 39
Doctors 14, 36%
Nurses 1, 3%
Research nurses 9, 23%
Physiotherapist 12, 31%

Parent Interview details

Interviewed a mean of 27 days (SD7.8 days) after randomisation (range: 17-43 days).
Children 8 days (median) in PICU (range 2.5- 18 days),
Children 7 days on a ventilator (range: 2.5-17)
Children a mean of 70 days old (range 15-156 days). I took on average (mean) 37 minutes (SD 9.6 minutes, range 20-57).

Figure 1. Parent and staff recruitment by time point

209x297mm (150 x 150 DPI)

Due to the need to treat a patient in an emergency without delay, or because parents may not always be present when a child needs treatment, it is not always appropriate or possible to obtain consent before a child is entered into a trial. To enable research to be conducted in the emergency setting, many countries (including the UK) allow consent to be sought as soon as possible afterwards. This is for permission to use the data already collected and to continue in the trial. This is research without prior consent (sometimes

Figure 2 Description of RWPC read to participants during interviews

172x32mm (300 x 300 DPI)

The efficacy and mechanism of surfactant therapy for critically ill infants with bronchiolitis:

The **B**ronchiolitis **E**ndotracheal **S**urfactant **S**tudy (BESS)

A phase-2 blinded randomised air-placebo-controlled trial of endotracheal surfactant (poractant alfa) in critically ill infants with bronchiolitis

BESS Protocol v1.0 18/07/2018

Study Sponsor(s):
University of Liverpool
Research Support Office
2nd Floor Block D Waterhouse
3 Brownlow Street
Liverpool
L69 3GA

EudraCT number: 2018-001169-18
ISRCTN :
Research Ethics Ref:
Sponsor Ref: UoL001360
IRAS Ref: 220853

For peer review only

For peer review only - http://bmjopen.bmj.com/site/about/guidelines.xhtml

PROTOCOL APPROVAL

I, the undersigned, hereby approve this clinical study protocol:

Authorised by Chief Investigator:

Signature: ______________________________ **Date:** ______________

Prof. Malcolm (Calum) Gracie Semple, PhD, BM BCh (Oxon), FRCPCH, FRCPE, FHEA

Consultant in Paediatric Respiratory Medicine, Alder Hey Children's NHS Foundation Trust
Professor of Child Health and Outbreak Medicine, University of Liverpool

Institute in The Park,
Alder Hey Children's Hospital
Eaton Road
Liverpool
L12 2AP

Authorised on behalf of Sponsor:

Signature: ______________________________ **Date:** ______________

Mr Alex Astor

Head of Research Support

Research Support Office

2nd Floor Block D Waterhouse Building

3 Brownlow Street,

Liverpool

L69 3GL

Authorised on behalf of the Lead Statistician:

Signature: ______________________________ **Date:** ______________

Dr Ashley Jones

Senior Statistician

Clinical Trials Research Centre

Institute of Child Health

Alder Hey Children's Hospital

Eaton Road
Liverpool
L12 2AP

For peer review only - http://bmjopen.bmj.com/site/about/guidelines.xhtml

General Information

This document describes the BESS trial including detailed information about procedures and recruitment. The protocol should not be used as an aide-memoir or guide for the treatment of other patients; every care was taken in its drafting, but corrections or amendments may be necessary. These will be circulated to the registered investigators in the trial, but centres entering patients for the first time are advised to contact the coordinating centre (Clinical Trials Research Centre (CTRC), University of Liverpool) to confirm they have the most up to date version. Clinical problems relating to this trial should be referred to the relevant Chief Investigator, Professor Calum Semple, via the CTRC.

This protocol defines the participant characteristics required for study entry and the schedule of treatment and follow-up. Participant recruitment will be undertaken in compliance with this document, applicable regulatory and governance requirements and waivers to authorise non-compliance are not permitted.

Incidents of protocol non-compliance, whether reported prospectively (e.g. where a treatment cannot be administered on a scheduled date as a result of public holidays) or retrospectively noted (e.g. as a result of central monitoring) are recorded as protocol deviations, the incidence of which are monitored and reported to trial oversight committees.

The template content structure is consistent with the SPIRIT (Standard Protocol Item: Recommendations for Interventional Trials 2013) and has regard for the Health Research Authority guidance. Regulatory and ethical compliance information is located in section 12.

Relationship Statements

Roles and responsibilities are described in section 15

The University of Liverpool is the Sponsoring organisation. The University of Liverpool will formally delegate specific sponsoring roles to the Chief Investigator and Clinical Trials Unit, but remains legally responsible for the trial.

Clinical Trials Unit (CTU): The CTRC at the University of Liverpool in collaboration with the Chief Investigator, Prof Calum Semple, will have overall management responsibility for the trial from a CTU perspective and will be responsible for the co-ordination of centres.

CTRC as part of the Liverpool Trials Collaborative has achieved full registration by the UK Clinical Research Collaboration (www.ukcrc.org) as their standards and systems were assessed by an international review panel as reaching the highest quality. The CTRC has a diverse trial portfolio underpinned by methodological rigour, a Good Clinical Practice (GCP) compliant data management system, and core standard operating procedures (SOPs).

For peer review only - http://bmjopen.bmj.com/site/about/guidelines.xhtml

**Sponsor:**	**Trial Management, Monitoring and Analysis:**
University of Liverpool<br>Liverpool<br>L69 3BX<br>Tel: 0151 794 8290<br>Email: sponsor@liverpool.ac.uk	Clinical Trials Research Centre, University of Liverpool<br>Institute of Child Health<br>Alder Hey Children's Hospital NHS Foundation Trust<br>Liverpool<br>L12 2AP<br>Tel: 0151 795 8757<br>Fax: 0151 795 8770<br>Email: bess@liverpool.ac.uk
**Mechanistic & Microbiology Work:**	**Paediatric Intensive Care Audit Data:**
University of Southampton<br>University Road<br>Southampton<br>SO17 1BJ<br>Tel: 023 8059 5000<br>Email: ris@soton.ac.uk	PICANet<br>School of Medicine<br>8.49 Worsley Building<br>University of Leeds<br>Leeds<br>LS2 9JT<br>PICANet<br>0113 343 8125<br>picanet@leeds.ac.uk

For peer review only - http://bmjopen.bmj.com/site/about/guidelines.xhtml
For peer review only

Contact Details: Individuals

**Individual Authorised to Sign the Protocol and Protocol Amendments on behalf of the Sponsor:**	**Chief Investigator (CI):**
Mr Alex Astor<br>Research Support Office<br>2nd Floor, Block D, Waterhouse Building, 3 Brownlow Street, Liverpool, L69 3GL<br>0151 794 8739<br>sponsor@liv.ac.uk	Prof. MG (Calum) Semple,<br>Women's and Children's Health<br>University of Liverpool<br>Institute in the Park, Alder Hey Children's Hospital, Eaton Road, Liverpool, L12 2AP, UK<br>0151 252 5250<br>m.g.semple@liverpool.ac.uk
**Medical Expert who will Advise on Protocol Related Clinical Queries (If other than CI):**	**Medical Expert who will Evaluate SAE Reports (if other than CI):**
Prof. Paul S McNamara<br>Women's and Children's Health<br>University of Liverpool<br>Institute in the Park, Alder Hey Children's Hospital, Eaton Road, Liverpool, L12 2AP, UK<br>0151 252 5250<br>mcnamp@liverpool.ac.uk	Prof. Paul S McNamara<br>Women's and Children's Health<br>University of Liverpool<br>Institute in the Park, Alder Hey Children's Hospital, Eaton Road, Liverpool, L12 2AP, UK<br>0151 252 5250<br>mcnamp@liverpool.ac.uk
**Medical Expert who will Evaluate SAE Reports (If other than CI or cross cover):**	
Dr Kent Thorburn<br>Paediatric Intensive Care Unit, Alder Hey Children's Hospital, Eaton Road, Liverpool, L12 2AP, UK<br>0151 228 4811 ext 4180<br>kent.thorburn@alderhey.nhs.uk	

For peer review only - http://bmjopen.bmj.com/site/about/guidelines.xhtml

Additional Contacts:

The contact details of other individuals involved in the trial are detailed in documents supplementary to the protocol and stored in the Trial Master File:

Contact	Document Title
Trial Management Group (TMG)	BESS Trial Oversight Committee Membership
Trial Steering Committee (TSC)	
Independent Data and Safety Monitoring Committee (IDSMC)	
Principal Investigators	BESS Participating Centres

For peer review only

For peer review only - http://bmjopen.bmj.com/site/about/guidelines.xhtml

1 TABLE OF CONTENTS

Protocol Approval **2**
1 Table of Contents **7**
Glossary **12**
2 Protocol Summary **14**
3 Introduction **16**
3.1 Study Overview 16
3.2 Background 16
3.3 Rationale 17
3.4 Risk and Benefits 18
3.4.1 Potential Benefits 18
3.4.2 Potential Risks 18
3.5 Aims & Objectives of Work Package A 19
3.5.1 Aims 19
3.5.2 Primary Objective 19
3.5.3 Secondary Objective(s) 19
3.6 Outcome measures/endpoints 19
3.6.1 Primary Outcome/Endpoint 19
3.6.2 Secondary Outcomes/Endpoints 19
4 TRIAL DESIGN **23**
4.1 Trial Overview 23
Schematic of Trial Design: 24
4.2 Use of a standardised weaning and extubation protocol 25
5 Trial Setting and Selection of SITES & Clinicians **26**
5.1 Selection of Sites & Clinicians 26
6 Study Population **27**
6.1 Inclusion Criteria 27
6.1.1 Definition of bronchiolitis 27
6.2 Exclusion Criteria 27
6.3 Co-enrolment Guidelines 27
7 Recruitment and Randomisation **29**
7.1 Participant Identification and Screening 29
7.2 Informed Consent 29

For peer review only - http://bmjopen.bmj.com/site/about/guidelines.xhtml

7.2.1 Prospective Informed Consent Process 29
7.2.2 Consent for additional work 31
7.3 Randomisation Procedure 31
7.3.1 Randomisation System Failure 32
7.4 Blinding to Allocations....32
8 Participant Time Line, Assessments and Procedures 33
8.1 Administration of Intervention 33
8.1.1 Immediately prior to administration 33
8.1.2 Administration of allocated intervention....34
8.2 Participant follow-up 34
8.3 Schedule for Intervention & Follow-up 36
8.4 Procedures for assessing Efficacy....38
8.4.1 Efficacy Assessment 1....38
8.4.2 Efficacy Assessment 2....38
8.5 Procedures for Assessing Safety....39
8.6 Other Assessments 39
8.6.1 Parent Reported Quality of Life questionnaires....39
8.6.2 Special Assays or Procedures 39
8.7 Patient Transfer and Withdrawal....40
8.7.1 Patient Transfers 40
8.7.2 Withdrawal from the Trial....41
8.8 Loss to Follow-up 42
8.9 Trial Closure....42
9 Trial Treatment/interventions 43
9.1 Introduction....43
9.2 Arm A – Endotracheal Poractant Alfa at 200mg/kg (first dose), with up to two further doses at 100mg/kg 43
9.2.1 Formulation, Packaging, Labelling, Storage and Stability....43
9.2.2 Preparation, Dosage and Administration of Study Treatment/s....44
9.2.3 Specific Restrictions 45
9.2.4 Overdose....45
9.2.5 Temperature Compliance 45
9.2.6 Accountability Procedures for Trial Treatments....46
9.2.7 Assessment of Compliance with Study Treatment/s 47
9.3 Arm B – Placebo....47

For peer review only - http://bmjopen.bmj.com/site/about/guidelines.xhtml

9.4 Unblinding 47
9.5 Concomitant Medications 48
10 Safety Reporting 49
10.1 Terms and Definitions....49
10.2 Notes on Adverse Event Inclusions and Exclusions....50
10.2.1 Include: 50
10.2.2 Do Not Include: 50
10.2.3 Notification of deaths 51
10.3 Notes on Severity / Grading of Adverse Events 51
10.4 Relationship to Trial Treatment....52
10.5 Expectedness....52
10.6 Follow-up After Adverse Events....53
10.7 Time Period for Safety Reporting....53
10.8 Reporting Procedures....53
10.8.1 Flowchart for Reporting Requirements of Adverse Events....54
10.8.2 Non serious events (ARs/AEs)....56
10.8.3 Serious events (AEs/ARs/SUSARs)....56
10.9 Safety Reporting Responsibilities – Investigator 56
10.9.1 Maintenance of Blinding....57
10.10 Safety Reporting Responsibilities – CTRC....58
10.11 Safety reports 59
10.12 Urgent Safety Measures 59
10.13 Contact Details and Out-of-hours Medical Cover 59
11 Statistical Considerations.... 61
11.1 Introduction....61
11.2 Method of Randomisation....61
11.3 Sample Size calculation....61
11.4 Interim Monitoring and Analyses....62
11.5 Analysis Plan....62
12 exploratory & translational work; work packages b and c.... 63
12.1 Work Package B: Study of Parent & Staff Experiences....63
12.1.1 Parent and staff perspectives sub study analysis....64
12.2 Work Package C:....64
12.2.1 Metabolic profiling of surfactant composition and kinetics 65

For peer review only - http://bmjopen.bmj.com/site/about/guidelines.xhtml

12.2.2 Recovery of surfactant proteins A & D (SP-A & SP-D) is modulated by exogenous surfactant. 66
12.2.3 Lower airway inflammation is modulated by exogenous surfactant 67
12.2.4 Treatment efficacy or failure is associated with detection of specific pathogens. 67
12.2.5 Mechanistic Protocol 67
13 Regulatory and Ethical Approvals 69
13.1 Statement of Compliance 69
13.2 Regulatory Approval 70
13.3 Ethical Considerations 70
13.4 HRA Approval 70
13.5 Ethical and Local Governance Approval 70
13.6 Protocol Deviation and Serious Breaches 71
13.7 Study Discontinuation 71
14 Data Management and Trial Monitoring 72
14.1 Source Documents 72
14.2 Data Capture Methods 72
14.3 Monitoring 73
14.3.1 Risk Assessment 73
14.3.2 Central Monitoring 73
14.3.3 Clinical Site Monitoring 73
14.4 Confidentiality 74
14.5 Quality Assurance (QA) and Quality Control (QC) 74
14.6 Records Retention 75
15 Indemnity 76
16 Roles and Responsibilities 77
16.1 Role of Study Sponsor and Study Funder 77
16.2 Funding and Support in Kind 77
16.3 Protocol Contributors 78
16.4 Trial Committees 79
16.4.1 Trial Management Group (TMG) 79
16.4.2 Trial Steering Committee (TSC) 79
16.4.3 Independent Data and Safety Monitoring Committee (IDSMC) 79
17 Publication and Dissemination 80
17.1 Publication Policy 80
17.1.1 Authorship 80

For peer review only - http://bmjopen.bmj.com/site/about/guidelines.xhtml

17.2 Dissemination to Key Stakeholders 80
17.1 Data Sharing 81
18 Chronology of Protocol Amendments 82
18.1 Version 1.0 (dd/mon/yyyy) 82
19 References 83
20 Documents Supplementary to the protocol 86

For peer review only

For peer review only - http://bmjopen.bmj.com/site/about/guidelines.xhtml

Glossary

AE	Adverse Event
AR	Adverse Reaction
BAL	Bronchoalveolar lavage
CI	Chief Investigator
CRF	Case Report Form
CTIMP	Clinical Trials of an Investigational Medicinal Product
CTRC	Clinical Trials Research Centre
CTU	Clinical Trials Unit
EUDRACT	European Clinical Trials Database
DSUR	Development Safety Update Report
FiO_2	Fraction of inspired oxygen
GP	General Practitioner
HRA	Health Research Authority
IB	Investigator's Brochure
IDSMC	Independent Data and Safety and Monitoring Committee
IMP	Investigational Medicinal Product
ISF	Investigator Site File
LRSQ	Liverpool Respiratory Symptom Questionnaire
MHRA	Medicines and Health Care Products Regulatory Agency
MV	Mechanical Ventilation
NIMP	Non Investigational Medicinal Product
NRES	National Research Ethics Service
NIHR CRN	National Institute for Health Research Clinical Research Network
OI	Oxygenation Index
OSI	Oxygen Saturation Index
PEEP	Positive End Expiratory Pressure
PI	Principal Investigator
PICANet	Paediatric Intensive Care Audit Network
PICU	Paediatric intensive care unit
PIP	Peak Inspiratory Pressure
PROM	Parent Reported Outcome Measure
QA	Quality Assurance
QC	Quality Control

For peer review only

For peer review only - http://bmjopen.bmj.com/site/about/guidelines.xhtml

R&D	Research & Development
REC	Research Ethics Committee
RN	Research Nurse (Registered)
RSI	Reference Safety Information
RSO	Research Support Office
SAE	Serious Adverse Event
SAR	Serious Adverse Reaction
SDV	Source Data Verification
SMPC	Summary of Product Characteristics
SOP	Standard Operating Procedure
SpO_2	Peripheral oxygen saturation
SSAR	Suspected Serious Adverse Reaction
SUSAR	Suspected Unexpected Serious Adverse Reaction
TMG	Trial Management Group
TMP	Trial Monitoring Plan
TSC	Trial Steering Committee
UAR	Unexpected Adverse Reaction
VI	Ventilation Index
WP-A	Work package A "The Trial"
WP-B	Work package B "Exploration of parental experience"
WP-C	Work package C "Mechanistic sub-studies"

For peer review only - http://bmjopen.bmj.com/site/about/guidelines.xhtml

2 PROTOCOL SUMMARY

**Full Title**	**The efficacy and mechanism of surfactant therapy for critically ill infants with bronchiolitis: The Bronchiolitis Endotracheal Surfactant Study (BESS). A phase-2 blinded randomised air-placebo-controlled trial of endotracheal surfactant (poractant alfa) in critically ill infants with bronchiolitis**
**Acronym**	**BESS**
**Phase**	2
**Target Condition**	Bronchiolitis of infancy requiring conventional invasive Mechanical Ventilation (MV)<br>Diagnosis of bronchiolitis per clinical criteria defined in national guidance NICE-NG9.
**Sample size**	284
**Main Inclusion Criteria**	1. Term-born infants < 26 weeks old and preterm-born infants < 26 weeks corrected age<br>2. Diagnosis of bronchiolitis<br>3. Requires conventional invasive MV via tracheal intubation<br>4. Parent or person with parental responsibility has given written informed consent for trial participation
**Main Exclusion Criteria**	1. Major congenital anomalies<br>2. Congenital neuromuscular disease<br>3. Already intubated for MV for >48 hours or likely to have been intubated for MV for >48 hours by randomisation<br>4. Have received or are receiving extracorporeal membrane oxygenation (ECMO) or oscillation during this episode of bronchiolitis<br>5. Have received or are receiving intratracheal administration of any surfactant during this episode of bronchiolitis<br>6. Receiving MV for primary apnoea rather than respiratory failure<br>7. A decision to wean to extubation has already been made<br>8. Clinical judgement of futility
**Study Centres and Distribution**	Approximately 14 Paediatric Intensive Care Units (PICUs) in UK tertiary hospitals.
**Patient Study Duration**	Duration of treatment: 24 hours (a maximum of three 12hourly intratracheal doses of surfactant; first dose (0hrs), second dose at 12hrs and third at 24hrs).

For peer review only - http://bmjopen.bmj.com/site/about/guidelines.xhtml

<table>
<tr><td colspan="2"></td><td colspan="2">Duration of follow-up: up to a maximum of 12 months, minimum 90 days.</td></tr>
<tr><td colspan="2">Overall Study duration</td><td colspan="2">44 months</td></tr>
<tr><td colspan="2">Agent/ Intervention</td><td colspan="2">Intervention: Endotracheal poractant alfa (Curosurf®, porcine lung phospholipid fraction supplied at 80mg/mL), first dose 200mg/kg, repeated 12 hourly at 100mg/kg, to a maximum of 3 doses according to the manufacturer's summary of product characteristics (SPC). Unlicensed use (UK licence, new indication).

Control: An identical series of up to three procedures (while intubated) using air as the placebo.</td></tr>
<tr><td colspan="2"></td><td>Objectives</td><td>Outcome Measures</td></tr>
<tr><td rowspan="2">Work Package A</td><td>Primary</td><td>To determine the efficacy of endotracheal surfactant in reducing total duration of MV by 18 hours or more, by random allocation of infants to receive either up to 3 doses of poractant alfa or up to 3 doses of air-placebo in an identical administration procedure (the Trial = Work package A)</td><td>The Primary Outcome measure for the Trial will be total duration of MV (hours) from randomisation to final extubation. We will include time off MV due to failed extubation.

Secondary Outcome measures relating to the Primary Objective of the trial are listed in the Protocol.</td></tr>
<tr><td>Secondary</td><td>To describe the impact of intervention on infants' long-term respiratory symptoms, using a parent reported outcome measure</td><td>The Liverpool Respiratory Symptom Questionnaire applied at 6m and 1yr after intervention in patients recruited in seasons 1 and 2.</td></tr>
<tr><td>Work Package B</td><td>Exploratory/ Translational:

Study of Staff & Parent Experiences</td><td>In a subgroup of patients, to explore staff and parent experiences of trial recruitment, consent and conduct in season 1 to inform season 2.</td><td rowspan="2">Outcome Measures relating to Exploratory and Translational aspects of the study are listed where known in section 12. Due to the exploratory nature of this work, other outcome measures are likely to be identified over this course of the study and after the trial has completed.</td></tr>
<tr><td>Work Package C</td><td>Exploratory/ Translational:

Mechanistic Study</td><td>In a subgroup of patients to explore the mechanisms for treatment efficacy and failures at a patient level by describing associations between trial outcome with markers of infection, inflammation, and surfactant composition in patient Bronchoalveolar Lavage Fluid.</td></tr>
</table>

NB: The Exploratory and Translational parts of the study (Work Packages B & C) will not inform the primary or secondary outcomes of the main trial. They are only included as an annexe to the Trial Protocol for regulatory purposes related to the Research Ethic Committee, Health Research Authority and Local Institutional Research permissions.

For peer review only - http://bmjopen.bmj.com/site/about/guidelines.xhtml

3 INTRODUCTION

3.1 Study Overview

The study "The efficacy and mechanism of surfactant therapy for critically ill infants with bronchiolitis" will have the short title "Bronchiolitis Endotracheal Surfactant Study" and the acronym BESS.

The award supporting BESS supports three work packages. These are: the trial (Work package A; WP-A); a study of staff and parental experience (Work package B; WP-B) and mechanistic exploratory sub-studies (Work package C; WP-C).

Work package A is subject to Medicines for Human Use (Clinical Trials) Regulations 2004 (as amended) and requires regulatory permission from the Medicines and Healthcare products Regulatory Agency (MHRA). Work packages B and C are dependent upon trial activity but do not provide outcome measures for the trial and so are not subject to these regulations. The study, which includes all work packages, requires REC, HRA and NHS permissions for which this unified protocol document is required. The detail of Work Packages B and C are described in Section 12.

3.2 Background

Bronchiolitis of infancy is a seasonal viral disease of the lower airways that most commonly causes feeding difficulties and respiratory distress and in severe cases causes a significant public health burden due to hospitalisation and mortality(1). It is the single most common reason for hospital admission of infants (children age<1yr). The youngest of these and those born prematurely are most often and most severely affected (1).

A recent study of Hospital Episode Statistics for England found 25,000 cases of bronchiolitis from each annual birth cohort (40.4 per 1000 (95% CI 38.5 to 42.3)) were admitted to hospital in their first year of life (2). Using PICANet data, the same study found just over 1000 of these infants were admitted with critical illness to English paediatric intensive care units (PICUs) each year (95% CI 1.3 to 1.6 per 1000 infants/yr) for mechanical ventilation (MV). This accounted for 12% of all admissions to PICUs. The infants' modal age was 1 month; the mean duration of PICU admission was 6.1 days (95% CI 5.4 to 6.7 days), and their case-fatality rate was 1.75%.

Despite advances in the provision of non-invasive modes of respiratory support, admissions to PICU and duration of MV for life-threatening bronchiolitis have remained static for the past 4 years (2). Excluding infants with rare congenital co-morbidities (mostly cardiac & neuromuscular anomalies), infants requiring MV for bronchiolitis do so for a mean of 4.8 days (SD 2.5d) (PICANet data, 2011-14).

Existing Research

In 2015 Jat & Chawla (3) found on pooled analysis of three small studies a significant reduction in length of PICU stay between endotracheal surfactant treated and control groups (mean difference (MD) -1.81d (95%CI -2.42 to -1.19) of critically ill infants with bronchiolitis who required MV. There were sustained improvements of arterial oxygenation (PaO_2) and carbon dioxide ($PaCO_2$) elimination (4-6). No adverse effects or complications were observed in the three studies. The authors of this systematic review and meta-analysis concluded that use of surfactant had favourable

For peer review only - http://bmjopen.bmj.com/site/about/guidelines.xhtml

effects but that larger trials with adequate power were required to evaluate the effectiveness of surfactant therapy in critically ill infants with bronchiolitis who required MV.

3.3 Rationale

There is no vaccine or specific treatment for bronchiolitis. Care is supportive as described in National Guidance (17). Breathing fails in very severe cases, and these babies need intensive care with breathing being supported by a machine called a ventilator, hence known as mechanical ventilation (MV).

Pulmonary surfactant is secreted by lung alveolar cells to reduce surface tension so increasing compliance of the lungs, allowing them to inflate more easily and reduce the work of breathing (8). Surfactant proteins A and D have innate antiviral immune properties as collectins (9).

Studies of infants with life-threatening bronchiolitis show reduced lung compliance and surfactant deficiency (10-14). These features are similar to the respiratory distress syndrome of infants born prematurely. Premature infants are routinely treated with animal-derived surfactant to improve lung compliance, and reduce mortality, morbidity and duration of MV (15).

Most infants with severe bronchiolitis are very young and/or born prematurely. They present at modal age 1 month, with 62.2% age <2months (2). We hypothesise that the effect of exogenous surfactant on infants with severe bronchiolitis may be similar to the therapeutic effect on those born prematurely as both conditions present with surfactant deficiency. At present surfactant is used without evidence as rescue therapy in some infants and young children with the most severe respiratory failure who are difficult to manage even with MV.

The systematic reviews by Jat & Chawla found that surfactant therapy in infants with critical illness due to bronchiolitis was: safe, improved gas exchange and reduced both duration of MV and length of stay on PICU (3, 16).

There are no other relevant trials registered on Clinicaltrials.gov, EU Clinical Trial Register or ISRCTN [search “surfactant” AND “bronchiolitis”, 6-JUL-2018].

This topic has been identified by the National Institute for Health Research (NIHR) Maternal Neonatal and Child Health panel in 2013 and selected by the Prioritisation Group for commissioning by both the Health Technology Assessment (HTA) and Efficacy and Mechanism Evaluation (EME), and has the support of the NIHR Translational Research Partnership (TRP) for Inflammatory Respiratory Diseases and UK Paediatric Intensive Care Society Study Group (PICS-SG).

Research Question

Does surfactant therapy reduce duration of invasive mechanical ventilation when given to infants early in the course of critical illness due to bronchiolitis?

Intervention

The intervention being trialled is:

Endotracheal poractant alfa (Curosurf®, porcine lung phospholipid fraction supplied at 80mg/mL), first dose 200mg/kg, repeated 12 hourly at 100mg/kg, up to a total of 3 doses according to the manufacturer's summary of product characteristics (SPC).

Or-

An identical series of up to three procedures (while intubated) using air as the placebo.

For peer review only - http://bmjopen.bmj.com/site/about/guidelines.xhtml

Hypothesis

The hypothesis we will test in the trial is “Endotracheal surfactant reduces duration of mechanical ventilation by 18 hours

3.4 Risk and Benefits

3.4.1 Potential Benefits

The potential benefit of the therapy to the participants is a sustained improvement in gas exchange leading to a reduction of total duration of MV. The potential benefit to the participant is a reduction of time in a technology-dependent vulnerable state, reduced risk of MV associated morbidity, reduced exposure to sedation and muscle paralysing drugs and a more rapid return to usual parental care. This in turn would reduce parental anxiety. Also, freeing up PICU beds during winter surge offers savings and opportunity to use resources for other conditions.

3.4.2 Potential Risks

Administration of poractant alfa (porcine derived surfactant, Curosurf®) in infants with bronchiolitis is well tolerated and considered safe(3). Transient minor adverse reactions associated with administration of poractant alfa for the treatment of neonatal Respiratory Distress Syndrome (RDS) include bradycardia, hypotension, endotracheal tube blockage, and oxygen desaturation. The risk of such events is minimised by tracheal toilet prior to administration of poractant alfa, as recommended in the Summary of Product Characteristics.

Small volume (2x1mL/kg) non-bronchoscopic bronchoalveolar lavage (BAL) procedure on PICU is safe and well tolerated procedure(7). In a published audit of 27 children on PICU who underwent multiple small volume BAL installations (5x1mL/kg) as part of a single routine diagnostic procedure, 7 experienced transient oxygen desaturation requiring temporary increased FiO_2. We will limit BAL sampling to a 2x1mL/kg instillation at each scheduled assessment.

Small volume (1.25mL (range 1.0 to 1.3mL)) blood sampling will be done on infants ventilated at Liverpool and Southampton PICUs and scheduled to coincide with routine blood sampling. Existing vascular access will be used when available. There is no risk of anaemia associated with this limited volume of blood sampling. The sample will be taken from an arterial line if present. If not by standard venesection.

Capillary blood gas sampling (100microliters) or arterial blood gas sampling for subject monitoring will use data from routine blood gas sampling when either is collected within a 2-hour window of a research-scheduled sample. As this is part of standard care no additional risks are expected.

For peer review only - http://bmjopen.bmj.com/site/about/guidelines.xhtml

3.5 Aims & Objectives of Work Package A

3.5.1 Aims

1. To conduct a multicentre blinded randomised placebo-controlled phase-2 trial of the endotracheal surfactant poractant alfa (CUROSURF®) as therapy for bronchiolitis in critically ill infants.
   Hypothesis to test: Endotracheal surfactant reduces duration of mechanical ventilation by 18 hours (Work package A (WP-A)).
2. To evaluate the safety profile in the two treatment groups

3.5.2 Primary Objective

To determine the efficacy of endotracheal surfactant in reducing total duration of MV by 18 hours, by random allocation of infants to receive up to 3 doses of poractant alfa or up to 3 doses of air-placebo in an identical administration procedure.

3.5.3 Secondary Objective(s)

1. To describe the impact of intervention on infants' long-term respiratory symptoms
2. To describe secondary outcomes of efficacy including physiological indices and duration of other modes of respiratory support.
3. To assess the safety of the intervention for infants with life-threatening bronchiolitis

3.6 Outcome measures/endpoints

3.6.1 Primary Outcome/Endpoint

The primary outcome measure will be total duration of MV (hours) from randomisation to final extubation. We will include time off MV due to failed extubation.

Justification: This outcome measure is clinically important, was suggested in both HTA and EME commissioning calls and is valued by our Parent Advisory Group. Reduced duration of MV reduces ventilator-associated morbidity, sedation, muscle relaxants, healthcare costs and bed occupancy at a time of increased seasonal pressure.

3.6.2 Secondary Outcomes/Endpoints

The secondary outcome measures for the trial (WP-A) are:

For efficacy:

1. duration of post-extubation non-invasive respiratory support (all forms of non-invasive ventilation: e.g. nasal CPAP and nasal high low oxygen)
2. duration of oxygen supplementation
3. number of trial interventions given (both poractant and placebo)
4. use of steroids to assist extubation
5. duration of stay on PICU and in hospital;

For peer review only - http://bmjopen.bmj.com/site/about/guidelines.xhtml

6. change over time from baseline of Ventilation Index (VI), Oxygenation Index (OI) and Oxygenation Saturation Index (OSI) while mechanically ventilated, & SpO_2/FiO_2 (SF) ratio.[1]
7. the score (value) of parent reported outcome measure of respiratory symptoms the "Liverpool Respiratory Symptom Questionnaire" (LRSQ) at 6m (+/-1m) and 12m (+/-1m) (20);
8. time from randomisation to meeting study criteria for readiness for Spontaneous Breathing Test

For safety:

1. failure to administer the intervention due to any adverse event during preparatory processes (tracheal toilet or BAL)
2. failure to complete administration of the intervention due to any adverse event during administration of the intervention (regardless of which arm of allocation)
3. incidents of 'air leak' (pneumothorax and pneumomediastinum) occurring before discharge from PICU
4. other adverse events and serious adverse events associated with the intervention
5. any need to replace the endotracheal tube
6. parent reported readmission to hospital (all causes) up to 90 days post randomisation
7. death during PICU admission
8. all-cause mortality at 90 days post randomisation

For peer review only

[1] VI and OI are validated physiological measurements reflecting respiratory function (gas exchange) normalised for level of respiratory support provided by MV, but require blood sampling(21). OSI is a validated physiological measurements reflecting respiratory function (gas exchange) normalised for level of respiratory support provided by MV that does not require blood sampling (41). The SF ratio is a non-invasive measure of respiratory function and can be calculated in situations where invasive blood gas monitoring and MV has ceased (22).

For peer review only - http://bmjopen.bmj.com/site/about/guidelines.xhtml

Table 1: Objectives and Outcome Measures

Objectives	Outcome Measures	Timepoint(s) of evaluation of this outcome measure (if applicable)
**Primary Objective**<br>To determine the efficacy of endotracheal surfactant in reducing total duration of MV by 18 hours or more, by random blind allocation of infants to receive either up to 3 doses of poractant alfa or up to 3 doses of air-placebo in an identical administration procedure.	Total duration of MV (hours) from randomisation to final extubation, including any time off MV due to failed extubation.	N/A
**Secondary Objective 1**<br>To describe the impact of intervention on infants' long term respiratory symptoms	The score (value) of patient reported outcome measure of respiratory systems in the "Liverpool Respiratory Symptom Questionnaire" (LRSQ)	LRSQ at 6 months and 12 months post randomisation
**Secondary Objective 2**<br>To describe secondary outcomes of efficacy including physiological indices and duration of other modes of respiratory support	Time from randomisation to meeting study criteria for readiness for Spontaneous Breathing Test	N/A
	Number of trial interventions given	N/A
	Change over time from baseline of Ventilation Index (VI), Oxygenation Index (OI) and Oxygenation Saturation Index (OSI) while mechanically ventilated, & SpO_2/FiO_2 (SF) ratio	Capillary blood gas sampling immediately prior to intervention and then at 6, 12, 24, 36 and 48hrs while still on MV; to continue daily after 48hrs if still on MV.
	Duration of oxygen supplementation	N/A
	Use of steroids to assist extubation	N/A

For peer review only - http://bmjopen.bmj.com/site/about/guidelines.xhtml

	Duration of post-extubation non-invasive respiratory support	N/A
	Duration of stay on PICU and in hospital	N/A
**Secondary Objective 3**<br>To assess the safety of the intervention for infants with life-threatening bronchiolitis	Failure to administer the intervention due to any adverse event during preparatory processes (tracheal toilet or BAL)	Randomisation to 24 hours after final trial intervention
	Failure to complete administration of the intervention due to any adverse event during administration of the intervention (regardless of which arm of allocation)	Randomisation to 24 hours after final trial intervention
	Incidents of 'air leak' (pneumothorax and pneumomediastinum) occurring before discharge from PICU	Randomisation to discharge from PICU
	Other adverse events and serious adverse events associated with the intervention	Randomisation to 24 hours after final trial intervention
	Any need to replace the endotracheal tube	Randomisation to successful extubation
	Parent reported readmission to hospital (all causes) up to 90 days post randomisation	PICU discharge to 90 days post-randomisation
	Death during PICU admission	Randomisation to PICU discharge
	All-cause mortality at 90 days post randomisation	PICU discharge to 90 days post-randomisation

For peer review only - http://bmjopen.bmj.com/site/about/guidelines.xhtml
For peer review only

4 TRIAL DESIGN

4.1 Trial Overview

The BESS trial is a phase-2 blinded randomised air-placebo-controlled trial of endotracheal surfactant (poractant alfa) in critically ill infants with bronchiolitis (we will refer to this as Work Package A, or WP-A).

For peer review only

For peer review only - http://bmjopen.bmj.com/site/about/guidelines.xhtml

Schematic of Trial Design:

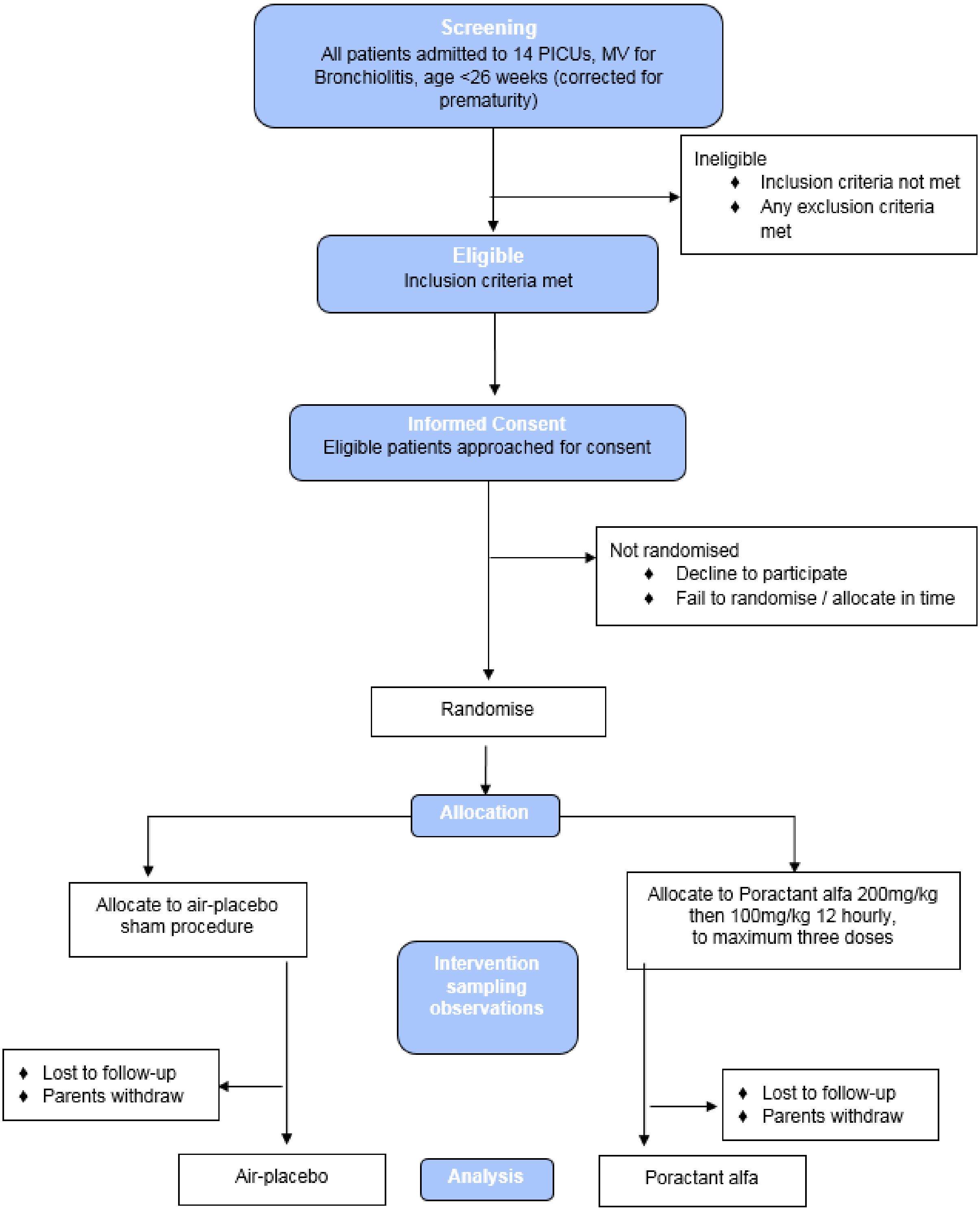

Figure 1: Schematic of Trial Design

For peer review only - http://bmjopen.bmj.com/site/about/guidelines.xhtml

4.2 Use of a standardised weaning and extubation protocol

On the direction of the NIHR EME board, the BESS trial protocol will include a standardised protocol for weaning from mechanical ventilation and extubation.

Weaning and extubation by protocol will standardise this aspect of care across sites and in both arms of BESS. Infants in BESS will be extubated after they meet specified physiological criteria that indicate the infant is ready for a Spontaneous Breathing Test (SBT) and pass that test, (see section 8.1.2.1 for further details).

For peer review only

For peer review only - http://bmjopen.bmj.com/site/about/guidelines.xhtml

5 TRIAL SETTING AND SELECTION OF SITES & CLINICIANS

Approximately fourteen Paediatric Intensive Care Units (PICUs) in the United Kingdom that routinely mechanically ventilate critically ill infants with high caseloads of bronchiolitis will recruit to BESS. PIs for sites that have provisionally agreed to participate in BESS have shared local activity data, commented on the application and are recognised as co-applicants on the application award. The PIs will champion the study at the sites and take local responsibility for the conduct of the trial. PIs will identify local co-Investigators.

5.1 Selection of Sites & Clinicians

Criteria for the selection of centres includes

- NHS Hospitals in the UK providing paediatric intensive care
- Historic high case load of bronchiolitis, as reported to PICANet
- Sufficient research capacity comprised of staff, time and facilities to undertake the trial; including patient screening and recruitment, randomisation, collection and provision to the CTRC of all required data, collection of research samples, identification and management of adverse events including notification to CTRC within protocol defined timeframes, identification and provision of information to the CTRC of all protocol breaches.
- All staff contributing to the trial must have valid certified Good Clinical Practice (GCP) training throughout the conduct of the trial. Requirements of the Sponsor are that renewal of GCP must occur every 3 years.
- Willingness to participate

Final decisions on site selection will be determined by the Trial Management Group (TMG) and will be described in the supplementary document 'BESS Site Suitability Assessment.

Initiation of centres will be undertaken in compliance with CTRC SOPs[2]. Centres fulfilling the criteria will be selected to be recruitment centres for the BESS trial and will be opened to recruitment upon successful completion of all global (e.g. MREC and MHRA) and study-specific conditions (e.g. site personnel training requirements) and once all necessary documents have been returned to CTRC as detailed in the trial Green Light checklist.

Participating centres will be listed in the 'BESS Participating Centres' log, maintained separately to the protocol and stored in the Trial Master File (TMF).

[2] CTRC SOP TM017 Study Initiation in CTRC and TM018 Study Initiation at Sites

For peer review only - http://bmjopen.bmj.com/site/about/guidelines.xhtml

6 STUDY POPULATION

6.1 Inclusion Criteria

1. Term-born infants < 26 weeks old and preterm-born infants < 26 weeks corrected age†
2. Diagnosis of bronchiolitis (see below)
3. Requires conventional invasive MV via tracheal intubation
4. Parent or person with parental responsibility has given written informed consent for trial participation

Note: Infants with chronic lung disease of prematurity can be included in the trial.

† Correcting for prematurity is a well-established method for adjusting assessment of infants born prematurely who are anatomically, physiologically, immunologically and neuro-developmentally immature for their chronological age. In our study, term-born infants will be eligible for inclusion in the study up to chronological age of 26 weeks old (i.e. all those under 6 months old). Premature born infants have their age corrected to account for weeks of lost gestation, hence an infant born 12 weeks premature (i.e. before completing the 28th week of the 40 weeks of pregnancy) would still be eligible up to a chronological age of 38 weeks old (i.e. under 9 months), because that infant is still less than 26 weeks old when age is corrected for the premature birth.

6.1.1 Definition of bronchiolitis

Diagnosis of bronchiolitis will be made using clinical criteria defined in national guidance NICE-NG9 (17).

6.2 Exclusion Criteria

1. Major congenital anomalies
2. Congenital neuromuscular disease
3. Already intubated for MV for >48 hours or likely to have been intubated for MV for >48 hours by randomisation
4. Have received or are receiving extracorporeal membrane oxygenation (ECMO) or oscillation during this episode of bronchiolitis
5. Have received or are receiving intratracheal administration of any surfactant during this episode of bronchiolitis
6. Receiving MV for primary apnoea rather than respiratory failure
7. A decision to wean to extubation has already been made
8. Clinical judgement of futility

6.3 Co-enrolment Guidelines

The investigators are happy to enter in to co-enrolment agreements where feasible.

For peer review only - http://bmjopen.bmj.com/site/about/guidelines.xhtml

Where other trials are taking place at a site participating in BESS and the target population overlaps, sites should make the BESS team aware at the earliest opportunity so that the potential for co-enrolment can be discussed between the two trial teams. The BESS investigators are open to co-enrolment with other studies but this must be agreed between both Chief Investigators prior to randomisation into BESS.

For peer review only

For peer review only - http://bmjopen.bmj.com/site/about/guidelines.xhtml

7 RECRUITMENT AND RANDOMISATION

7.1 Participant Identification and Screening

All patients admitted to recruiting PICUs, receiving conventional MV for bronchiolitis, age <26 weeks (corrected for prematurity) will be screened for eligibility before trial entry. Screening and confirmation of full eligibility will be completed to allow randomisation within 48 hours of intubation.

A screening log of patients who are assessed for eligibility but not randomised will be maintained and returned to the CTRC on a regular basis, as this will provide important information for monitoring purposes. The screening log will capture which patients have been screened for the trial but assessed as ineligible, and those considered eligible and approached for consent but for whom consent was not obtained; reasons (all per subject) for non-inclusion will be recorded. Reasons for declining to participate will be asked routinely but it will be made clear that parents/persons with parental responsibility do not have to provide a reason unless happy to do so.

The screening log will not contain patient identifiers as those who are not eligible will not have provided their consent for identifiers to be captured as part of the trial. Therefore, sites will maintain their own trial register linking patients screening and/or randomisation numbers to their identifiers. Under no circumstances will this log be returned to the CTRC.

Patients will be reviewed for eligibility once they have been intubated for MV, or as soon as their arrival into PICU if they are already intubated. Parents/persons with parental responsibility for patients who meet the eligibility criteria and are suitable for inclusion (see 6.1 and 6.2) will be provided with information about the trial (both verbal and written) to consider participation.

Only a medically qualified doctor authorised on the site Delegation Log can confirm full eligibility of any patient. A record of this confirmation must be made in the patient's medical notes on the date of confirmation of eligibility.

7.2 Informed Consent

7.2.1 Prospective Informed Consent Process

Parents and those persons with parental responsibility may give consent for the patient to take part in BESS; hereafter throughout this protocol they shall be referred to as 'parent(s)'.

Initiation of the intervention in BESS is urgent and the condition "critical illness due to bronchiolitis requiring MV" is a medical emergency. Randomisation must be completed within 48 hours of intubation for MV, which will limit the time available for a parent to consider whether or not their child should participate. Site research teams will use their understanding of the clinical situation for each individual patient to judge when is most appropriate to approach parents about trial participation.

The trial concept will be introduced to a parent by familiar members of the usual clinical care team, be they nurses or physicians, as per the Canadian Critical Care Trials PICU Research Group trial recruitment model (24).

For peer review only - http://bmjopen.bmj.com/site/about/guidelines.xhtml

If a parent is interested, a member of the usual clinical care team will make an introduction to the research staff who will provide written study information and offer further verbal information.

Due to the time-critical nature of the intervention, the parent will have a limited timeframe to ask questions and make a decision about their child's participation in the trial. Randomisation must take place within 48 hours of intubation for MV and while some parents may have this full timeframe to consider trial participation, others may have considerably less due to time of arrival on PICU, time of approach etc. Consent will be sought by any appropriately trained and delegated medical staff and where local site policy allows, research nurses or respiratory physiotherapists (the research team).

Consent from the parent must be obtained prior to every infant participating in the trial. This consent will be sought after a full explanation has been given of the treatment options; including the conventional and generally accepted methods of treatment, the objectives, risks and inconveniences of the trial, and the conditions under which it is to be conducted.

Parent Information Sheet and Consent (PISC) forms, describing in detail the trial intervention, procedures and risks will be approved by a research ethics committee (REC). The parent will be asked to read and review this document. Upon reviewing the document, the person seeking consent will explain the research study to the parent. This information, and the supporting conversation, will emphasise that participation in the trial is voluntary and that the parent may withdraw their infant from the trial at any time and for any reason.

The parent will be given opportunity to ask any questions that may arise and time to consider the information, within the constraints of the 48 hour period between intubation and randomisation, prior to agreeing to participate. A contact point where further information about the trial may be obtained will be provided on the parent information sheet.

The parent will then sign and date the consent form if they agree to their infant participating. Both the person obtaining consent and the parent must sign and date the consent form; the researcher and parent must sign at the same time, with the researcher completing their signature **after** the parent.

A copy of the signed PISC will be given to the parent for their record. The original copy will be filed in the Investigator Site File, a copy filed in the participant's medical notes and a further copy should be sent to the CTRC. If the site has moved to an electronic health record (EHR), a scanned copy should be entered in the patient notes. A record of the consent process should also be recorded in the participant's medical notes.

The right of the parent to refuse consent for the infant to participate in the trial without giving reasons must be respected. After the infant has entered the trial, the clinician will remain free to give alternative treatment to that specified in the protocol, at any stage, if s/he feels it to be in the best interest of the patient. However, the reason for doing so should be recorded and the infant will remain within the trial for the purpose of follow-up and data analysis.

Similarly, the parent of the infant remains free to withdraw the infant at any time from the protocol treatment and trial follow-up without giving reasons and without prejudicing the further treatment of the infant (see section 8.6.2 for further details). They do not need to provide a reason for withdrawing their infant from the trial, which will be emphasised in the PISC. Any parent who withdraws their infant from the trial will be approached for participation in the sub-study of parental

For peer review only - http://bmjopen.bmj.com/site/about/guidelines.xhtml

experience (WP-B), unless they previously declined sub-study participation at consent (see Section 12).

7.2.2 Consent for additional work

Parents of eligible infants who have been approached to give consent for the BESS trial will also be offered the opportunity to take part in a telephone interview about their experiences of the consent process. This will be offered regardless of whether or not consent was given for the infant to participate in BESS (see section12.1).

An alternative PISC will be offered to parents at Liverpool and Southampton PICUs (sites participating in the Mechanistic work exploring surfactant metabolism; WP-C. See section 12.2).

A decision to decline to participate in either WP-B or WP-C will not impact upon care, or preclude involvement in the trial (WP-A).

7.3 Randomisation Procedure

Participants will be randomised in a 1:1 ratio, stratified by site and duration of ventilation prior to randomisation (<24hrs, ≥24hrs).

Randomisation must be completed within 48 hours of intubation.

Participants will be randomised using a secure 24-hour web-based randomisation and drug supply programme, managed by a third party subcontracted by Chiesi Farmaceutici S.p.A. (Chiesi). A personal login username and password will be required to access the randomisation system. Designated research staff will be issued with a unique personal login and password upon completion of training in the use of the system.

When the system requirements (confirmation of consent and eligibility) are confirmed the participant treatment allocation and a unique study number (randomisation number) will be displayed on a secure webpage. The randomising staff member will then be able to enter the resupply module within the system, where they will select the participant weight category and dose number (i.e. first, second or third) for the system to allocate the required number of vials for the participant. **Sites must complete this step for participants in both arms of the trial and for every given dose (first, second or third) regardless of allocation to drug or placebo.**

The randomisation and resupply system is configured to allow remote monitoring of stock levels at site by the distributor contracted by Chiesi, and further IMP stocks will be supplied once the amount available at site reaches an agreed trigger level. However, it remains the responsibility of the PI or delegated research staff to check the supplies available on the PICU prior to randomisation to ensure there is sufficient supply of the IMP, as the majority of stock will be held at the local site Pharmacy (see Section 9.2.6 for further information regarding intervention accountability).

Unblinded and appropriately delegated site staff will be able to log into the randomisation and resupply system at any time and view the allocations of each participant to ensure continuity between shifts.

For peer review only - http://bmjopen.bmj.com/site/about/guidelines.xhtml

7.3.1 Randomisation System Failure

In the event of a randomisation system failure, the centre should contact the coordinating team at the CTRC (Monday to Friday between 9:00 to 17:00 excluding bank holidays) to inform them of the problem. Out-of-hours the centre should contact the Chief Investigator or his delegates, on 07506 653560.

7.4 Blinding to Allocations

BESS will use an established strategy of a sham-procedure to blind as many research and usual care staff as possible to the nature of the intervention. This sham procedure was used in the development of surfactant as a therapy for surfactant deficiency of prematurity (6,19).

The participants, CTRC staff (excluding statistical team members as appropriate), and all members of the site research teams (excluding agreed randomising staff member, the administrator of the intervention and the dosage witness) will be blinded to treatment allocations. Occasional transient adverse effects associated with surfactant administration and back-tracking of surfactant up the endotracheal tube, plus the more frequently observed positive effects of rapid improvement in gas exchange immediately following surfactant administration, may result in some blinded care givers being able to infer that a participant has been allocated to the surfactant arm of the trial.

The randomising person, person drawing up the surfactant or air placebo and a second person checking the dose will be unblinded. Due to the transient effects and back-tracking it is likely that the Usual Care Nurse (UCN) will become unblinded, and we therefore recommend that if possible the UCN draws up the surfactant or acts as the witness to the drawing up, to minimise the number of people unblinded. Please see figure 2 for an intervention flowchart showing which staff/steps are blinded and unblinded.

Staff who are unblinded due to their involvement or believe themselves to have become unblinded for any reason are strongly discouraged from discussing this with other members of the team, unless the effect noted is causing an adverse event that requires action. Any staff member who believes themselves to have become unblinded must excuse themselves from any decision making regarding the weaning and extubation of the infant. The need to maintain blinding will be emphasised during all Site Training Visits.

For peer review only

For peer review only - http://bmjopen.bmj.com/site/about/guidelines.xhtml

8 PARTICIPANT TIME LINE, ASSESSMENTS AND PROCEDURES

8.1 Administration of Intervention

The first trial intervention must be administered within 2 hours of randomisation with subsequent doses (if required) at 12 and 24 hours post randomisation. A grace period of one hour either side of the anticipated administration time is permitted.

- Randomisation; **T0**
- First intervention must be given within two hours; between **T0** and **T2**
- Second intervention should be given 12 hours after randomisation; at **T12** (+/- 1 hour)
- Third intervention should be given 24 hours after randomisation; at **T24** (+/- 1 hour)

8.1.1 Immediately prior to administration

Prior to intervention and in a drug room away from usual staff, the study respiratory physiotherapist, research nurse or other delegated, unblinded staff member will:

- Draw up a dose of poractant alfa or an equal volume of air as placebo according to the random allocation into a syringe
- Ensure a second person is present to witness this process and check the dose volume
- Cover the syringe with an opaque paper shield to maintain blinding of other staff and parents
- Take the syringe to the bedside, along with syringes of sterile 0.9% sodium chloride for tracheal toilet and the BAL sampling set.

Infants in both the treatment and placebo arms of the trial will have 0.9% sodium chloride “normal saline” tracheal toilet and 0.9% sodium chloride BAL immediately prior to administration of the intervention. Both can cause immediate transient desaturation, need for increased sedation but also and more often rapid improvement in gas exchange when obstructing secretions are removed. Tracheal toilet and BAL can also lead to creamy white secretions appearing in the endotracheal tube, and this will be observed in both treatment and placebo groups.

Staff should remind parents, if present, of the purpose of the imminent study procedure and give them the opportunity to remain or leave as they choose. The usual care nurse will remain to care for the participant and assist as required.

Tracheal toilet is usually required for babies with bronchiolitis and will be done as recommended in the SPC before administration of poractant alfa. BAL sampling with 0.9% sodium chloride (2x1mL/kg) will be done if scheduled (section 8.2). Both tracheal toilet and BAL sampling is done by catheter access to the trachea; either via the endotracheal suction port without disconnecting the infant from the ventilator or by disconnecting the infant from the ventilator and performing hand bagging while a side-port with one-way valve are utilised for the toilet and BAL.

For peer review only - http://bmjopen.bmj.com/site/about/guidelines.xhtml

The use of muscle relaxant prior to tracheal toilet, BAL and or administration of the intervention is at clinical discretion.

8.1.2 Administration of allocated intervention

The intervention will be given per either method described in the SPC; either keeping the infant connected to the ventilator or momentarily disconnecting and performing hand-bagging (at the attending Respiratory Physiotherapist's decision based on patient status).

- Connect the syringe containing the surfactant or air-placebo to a suitable catheter (size 5F);
- Pass the catheter through the suction or side port into the endotracheal tube and advance the catheter, aiming to have the tip of the catheter placed just beyond the tip of the endotracheal tube
- Deliver the intervention (surfactant or air-placebo) slowly as a single bolus (recommended over 0.5-3 minutes) directly into the lower trachea
- The intervention (surfactant or air-placebo) will disperse through mechanical ventilation cycles into the peripheral lung tissue where the excipient (sterile buffered 0.9% sodium chloride) is absorbed in the alveoli and the surfactant phospholipid is deposited.

8.1.2.1 Weaning and extubation by protocol

The unblinded staff involved in the administration of the BESS intervention (study respiratory physiotherapist or nurse practitioner or other qualified person and one usual care PICU nurse) and parents who may be observing, will not be involved in decisions to wean respiratory support provided by mechanical ventilation and will not be involved in decisions to extubate.

We will wean respiratory support provided by MV and extubate according to a standardised protocol that will form part of the site-specific training packages.

Readiness for a spontaneous breathing test (SBT) will be identified by the patient meeting specified physiological targets of gas exchange at a given level of ventilation and/or the treating clinician believes the child to be ready for SBT.

The time at which a child is deemed ready for SBT **must** be recorded in their notes/electronic health record. The time that a child is extubated **must** be recorded in their notes/electronic health record.

8.2 Participant follow-up

Clinical follow-up (daily data collection) will take place for the period of time between randomisation and the discharge from hospital of the participant.

All non-serious adverse events will be collected until 24 hours after the participant receives their final trial intervention; please note that this may not be their third dose if less than 3 doses are

For peer review only - http://bmjopen.bmj.com/site/about/guidelines.xhtml

required before the participant meets the criteria to be ready for weaning (see section 10 for full safety reporting information).

Non-serious adverse events occurring after 24 hours post-final intervention but before 90 days post-randomisation/discharge home/death should be reported if the local Investigator believes that they may be related to the intervention.

Although active monitoring for adverse events and adverse reactions will cease at 24 hours post final intervention and 90 days post randomisation / discharge home / death respectively, any which the site research team become aware of after these time-points which are assessed as serious (i.e. Serious Adverse Events or Serious Adverse Reactions) should be reported to the CTRC.

Routinely collected audit data regarding participant care will be collected from PICANet until 90 days post randomisation.

Parent reported outcomes will be collected via the LRSQ at 6 and 12 months post randomisation for participants recruited in seasons 1 and 2, and at 6 months only for participants recruited in season 3.

The end of follow-up definition for each participant will therefore be 12 months post randomisation for participants in season 1 and 2, or 6 months post randomisation for participants in season 3.

For peer review only

For peer review only - http://bmjopen.bmj.com/site/about/guidelines.xhtml

8.3 Schedule for Intervention & Follow-up

Table 2: Schedule for intervention and follow-up

Procedures	Staff	Consent & randomisation (0-48hr)	0 prior to intervention	6hr	12hr, prior to second intervention	24hr, prior to third intervention	36hr	48hr	Daily if MV	90d	6m	12m
Prescribe	PI/Dr	X										
Invitation to participate in WP-B (parental experience sub-study)	PI/Dr/RN	X*										
+Invitation to participate in WP-C (surfactant metabolism sub-study)	PI/Dr/RN	X*										
ˣBlood Gas sample (capillary or arterial)	N/RN		X	X	X	X	X	X	X			
CRF completion (patient data collection)	N/RN		X	X	X	X	X	X	X			
ˣBAL	RP		X		X+	X	X+		X			
Administration of trial intervention	RP		X		X	X						
+IV D-Choline	N/RN		X+									
+Blood Sample (venous or arterial)	N/RN		X+		X+	X+	X+	X+				
Safety monitoring and reporting	RN/RP/PI **		X	X	X	X	X	X	X			
LRSQ	RN										X	X*
Mortality and re-admission	RN									X		

RN = Research Nurse, RP = Study respiratory physiotherapist or nurse practitioner or any other qualified person not involved in decision to wean or extubate, PI = Local Principal Investigator or delegate, N = Nurse (usual care), Dr = licensed medical practitioner.

ˣ Before each trial intervention

+ Only at Liverpool & Southampton and with specific consent (see section 12.2)

***** Seasons 1 & 2 only

****** Although all delegated staff are responsible for identifying and reporting adverse events, decisions regarding causality and seriousness, and sign-off of serious adverse event reports can only be undertaken by a licensed medical practitioner.

For peer review only - http://bmjopen.bmj.com/site/about/guidelines.xhtml

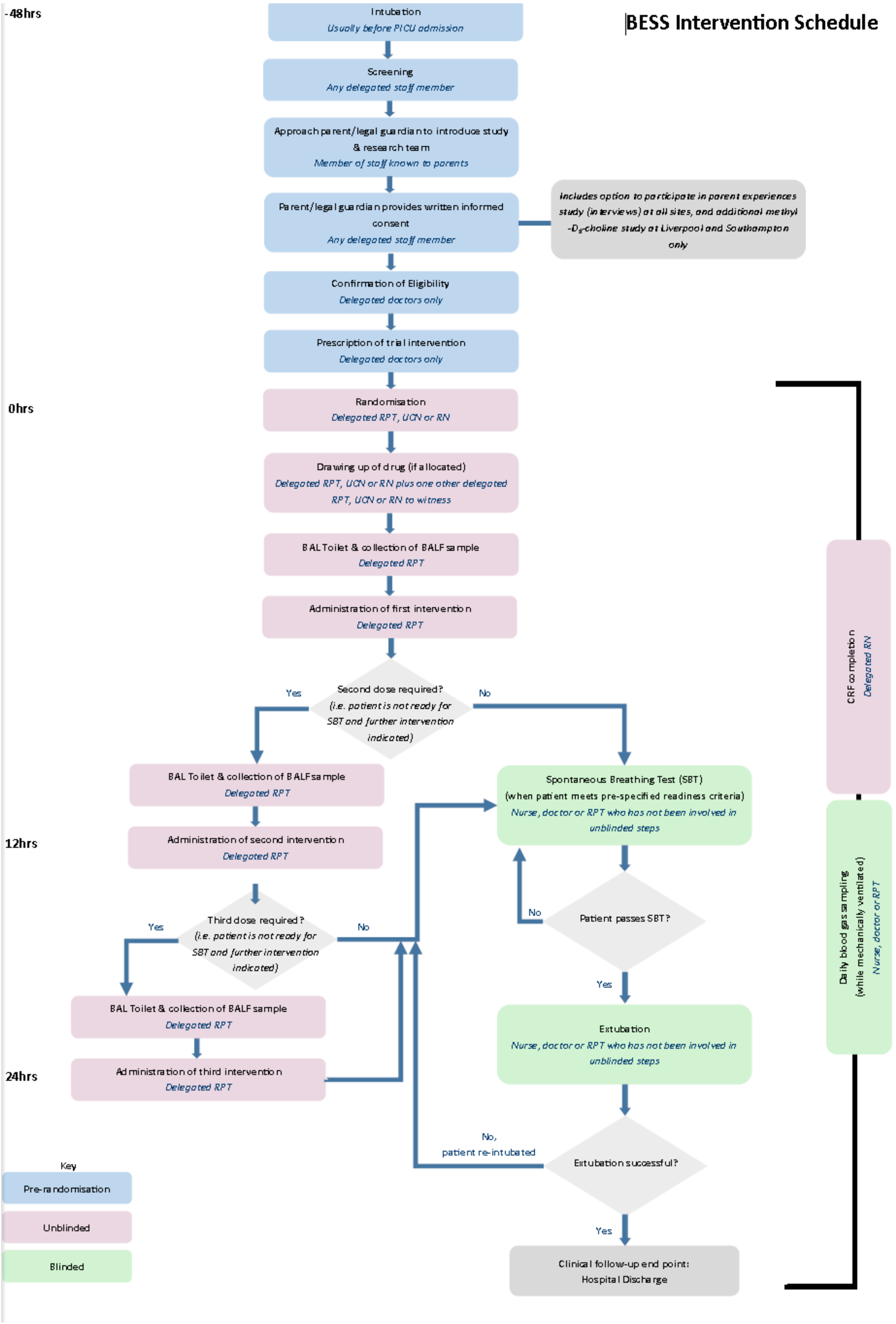

Figure 2: BESS Intervention Schedule

For peer review only - http://bmjopen.bmj.com/site/about/guidelines.xhtml

8.4 Procedures for assessing Efficacy

8.4.1 Efficacy Assessment 1

Efficacy will be assessed by group-wise comparison of the primary outcome measure group "Duration of MV after randomisation" by intention to treat. This is defined as time from randomisation to final extubation in hours, including any time off MV where the infant requires reintubation for a further period of MV. Time of randomisation and intervention will be recorded in the patients' notes and CRFs. MV ceases at extubation, an event when the endotracheal tube (breathing tube) is removed and the infant is allowed to breathe without invasive mechanical support. Extubation is an important discrete event in a patient's care pathway, is routinely recorded in the notes and PICANet audit.

8.4.2 Efficacy Assessment 2

The Ventilation Index (VI), Oxygenation Index (OI), Oxygenation Saturation Index (OSI) and SF ratio are secondary outcomes of efficacy derived from blood gas results (capillary blood gas sampling times as per Table 2 Schedule for intervention and follow-up), ventilator settings, and non-invasive monitoring. These data are captured through existing intensive care automated monitoring process or regular manual entry on large format (broad-sheet) bed-charts. This data will be transcribed by the research nurse to the CRF.

Mechanical Ventilation parameters to allow calculation of VI, OI, OSI and SF ratio must be recorded at the corresponding time when Blood Gas samples are scheduled to be taken for research or taken for routine purposes.

Mechanical Ventilation parameters are recorded regularly for ventilated patients as part of standard care. BESS does not require site teams to take any additional readings of these parameters for study purposes, but **all blood gas and ventilator readings** taken as part of standard care must be transcribed into the CRF to allow for calculation of these ratios over time.

After extubation, all inhaled Oxygen and peripheral oxygen saturation parameters recorded as part of standard care must be transcribed into the CRF to allow for calculation of this ratio over time.

Blood gas indices and ratio definitions are as follows:

- VI = ($PaCO_2$ × (PIP-PEEP) × respiratory rate)/1,000
- OI = (MAP × FiO_2 × 100)/PaO_2
- OSI = (MAP × FiO_2 × 100)/SpO_2
- SF = SpO_2/FiO_2

MAP = mean airway pressure

$PaCO_2$ = partial pressure of arterial carbon dioxide

FiO_2 = Fraction of air inspired that is oxygen

PaO_2 = partial pressure of arterial of oxygen

SpO_2= peripheral oxygen saturation

For peer review only - http://bmjopen.bmj.com/site/about/guidelines.xhtml

The time that physiological criteria are met that indicate suitability to test readiness for extubation by spontaneous breathing and/or the treating clinician believes the child to be ready for SBT will be recorded in the patient medical records and CRF.

All-cause mortality at 90 days after intervention will be established via a local enquiry against the hospital electronic health record.

8.5 Procedures for Assessing Safety

Safety will be assessed by:

- The Principal Investigator (PI) or delegated research staff actively monitoring and reporting all adverse events from randomisation until 24 hours after the participant's last administration of poractant alfa (see section 10 for adverse event reporting).
- Routinely collected patient care data downloaded from the PICANet audit on a regular basis by each site and transferred to the CTRC
- Mortality and readmission data collected by each site at 90 days post randomisation

Safety data will be actively monitored and all events reported from randomisation to 24 hours after the participant's last administration of poractant alfa or placebo for non-related events. Adverse events occurring after this time point until 90 days post randomisation / discharge home / death will be reported if felt to be related to the intervention. Any serious events or reactions (SAEs/SARs) should be reported to CTRC regardless of the timeframe.

8.6 Other Assessments

8.6.1 Parent Reported Quality of Life questionnaires

The parent reported outcome measure the Liverpool Respiratory Symptom Questionnaire (LRSQ), is a web-accessible questionnaire that has been optimised for many platforms including smart-phones. Links to the form will be emailed to parents prior to the intended completion dates (in all three seasons at 6m, and in addition at 12m to parents of infants recruited in seasons 1 and 2). A paper version is available with a "free-post" return envelope.

8.6.2 Special Assays or Procedures

8.6.2.1 Collection & processing of BAL fluid

BAL sampling with 0.9% sodium chloride (2x1mL/kg) will be done for all participants immediately prior to intervention, at 24hrs post intervention and repeated daily while the infant remains on MV (see Table 2).
Both tracheal toilet and BAL sampling is done by catheter access to the trachea via the endotracheal suction port without disconnecting the infant from the ventilator or by disconnecting the infant from the ventilator and performing hand bagging while accessing the trachea via a side-port with one-way valve.

For peer review only - http://bmjopen.bmj.com/site/about/guidelines.xhtml

8.6.2.2 Blood gas sampling

Blood Gas sampling is a routine procedure required for the clinical monitoring of all people requiring mechanical ventilation. The typical sample volume of blood drawn from infants and children is 100 microliters equivalent to two drops. Infants and children can have Blood Gas sampling by drawing blood from an existing arterial line or from a heel prick. To minimise disturbance and risks to infants from additional research procedures, the data from routine Blood Gas samples will be used for the trial when collected within a 2-hour window of the scheduled trial Blood Gas sample timepoint up to and including the sample due at 48 hours post first intervention (see Table 2). After 48 hours, i.e. day 3 and onwards, and only if the infant continues to require mechanical ventilation, then data from any one routine blood gas sample collected within a 6-hour window 9:00am to 3:00pm in each subsequent 24-hour interval will be collected (the first such sample if more than one is taken). A research Blood Gas sample will only be done if a routine blood gas sample is not done or planned within each 24-hour period sampling window.

Blood gas machines are usually kept in the PICU rather than the hospital/Trust laboratory. PICUs will not be CPS accredited but each Trust laboratory, and the machinery they are responsible for, will be. The CTRC will acquire a copy of the trust CPS laboratory certificate and calibration log for the blood gas machines as part of the compulsory 'Green Light Checklist' procedures before a site can open to recruitment. The Trust laboratory **must** take responsibility for service of the PICU blood gas machines and be able to provide a quality control log for maintenance and calibration of each blood gas machine.

8.6.2.3 Additional samples (WP-C at Liverpool and Southampton only)

Participants at Liverpool and Southampton will be given the option to consent to a single intravenous dose of D_9-labelled choline which will inform the mechanistic sub-study on surfactant synthesis and metabolism (WP-C; see section 12.2 for full details). This work will not inform either the primary or secondary outcomes of the main trial (WP-A). These samples will **only** be taken from participants who have provided specific consent for this optional additional work.

Small volume lung lavages will be collected immediately prior to intervention and at 24hrs, as per the standard follow-up (see section 8.2), and at additional time points of 12hrs and 36hrs for WP-C participants only. Samples of 1.25mL (range 1.0 to 1.3mL) of blood will be collected at the same time points for WP-C participants at Liverpool and Southampton only.

8.7 Patient Transfer and Withdrawal

8.7.1 Patient Transfers

It is very unlikely that participants taking part in BESS will be transferred during their trial treatment, given the severity of illness of participants and the short time period for administration of the intervention. In the unlikely scenario that a BESS participant is transferred while they remain intubated, their trial treatment will end at transfer unless they are transferred directly to another

For peer review only - http://bmjopen.bmj.com/site/about/guidelines.xhtml

BESS site. For participants transferred to another participating trial site the receiving trial site must take over responsibility for the participant.

A copy of the participant CRFs should be provided to the new site. The parent will have to sign a new consent form at the new site, and until this occurs, the participant remains the responsibility of the original site.

If a participant is transferred to a site that is not taking part in BESS then they will be withdrawn from the trial and will receive no further interventions. Their care will revert to the standard practice and will not be affected by the withdrawal from the trial. The CTRC should be notified in writing of all participant transfers, whether or not to a participating trial site.

8.7.2 Withdrawal from the Trial

In consenting to the trial, parents are consenting to trial intervention and treatment, follow-up assessments and data collection for their child.

The rights and welfare of the participants will be protected by emphasising to their parents that the quality of medical care will not be adversely affected if they withdraw consent for their child to participate in this trial.

8.7.2.1 Partial Withdrawal from Trial (Intervention and Treatment), allowing Follow-up and Data Collection)

Participants may be withdrawn from trial intervention and treatment for any of the following reasons:

a. Parent withdraws consent.

b. Unacceptable toxicity.

c. Intercurrent illness preventing further treatment.

d. Any change in the participant's condition that justifies the discontinuation of treatment in the clinician's opinion.

If voluntary withdrawal from trial intervention and treatment occurs, the parent should be asked to allow continuation of scheduled evaluations (follow-up and data collection) and be given appropriate care under medical supervision until the symptoms of any adverse event resolve or the participant's condition becomes stable.

If a parent wishes to withdraw their infant from trial intervention and treatment, sites should explain the importance of remaining on trial follow-up, or failing this, of allowing routine follow-up data to be used for trial purposes. Follow-up and data collection will continue unless the parent explicitly withdraws consent for follow-up or data collection. Research data obtained up to the point of withdrawal of consent will be retained, and further data will only be collected where required by law for reporting to regulatory agencies (i.e. serious adverse events). Research samples will be destroyed or retained for the purpose of the trial according to the wishes of the parent.

8.7.2.2 Withdrawal from Trial Completely

Parents are free to withdraw consent at any time without providing a reason. Parents who wish to withdraw consent for the trial completely (trial intervention, follow-up and data collection) will be informed that pseudo-anonymised data collected up to the point of that withdrawal of consent will be

For peer review only - http://bmjopen.bmj.com/site/about/guidelines.xhtml

included in the analyses, in accordance with current data protection legislation. The participant will not contribute further data to the trial (with the exception of serious adverse event data); the CTRC should be informed in writing and a Withdrawal CRF should be completed. Data up to the time of withdrawal will be included in the analyses.

8.8 Loss to Follow-up

Trial follow-up will be in part via routinely collected data downloaded in a bespoke trial download by each recruiting site; in part via paper CRFs completed by the trial RNs and RPTs; and in part via LRSQs collecting Parent Reported Outcome Measures (PROMs) at 6 and 12 months post randomisation. Where a patient is lost to follow-up before the relevant time point (e.g. lost due to transfer to another hospital), contact will initially be attempted by the trial RNs and the PI at each site. Wherever possible, information on the reason for loss to follow-up will be recorded.

8.9 Trial Closure

The end of the trial is defined to be the date on which data for all participants are locked and data entry privileges are withdrawn from the trial database. However, the trial may be closed prematurely by the Trial Steering Committee (TSC), on the recommendation of the Independent Data and Safety Monitoring Committee (IDSMC).

Individual sites may be closed to recruitment prior to their intended recruitment end date if the Trial Management Group (TMG) have concerns about their capacity or capability to deliver the trial, or for operational reasons whereby resources are better used at sites with better capacity to recruit.

At the point of closure to recruitment all sites will be required to undertake closedown activities which include but are not limited to: a review of their Investigator Site File (ISF), a count of all completed CRFs and completed data queries. PIs will also be required to sign-off the CRF for each participant, any changes to the data, a closedown checklist and their site delegation log.

For peer review only

For peer review only - http://bmjopen.bmj.com/site/about/guidelines.xhtml

9 TRIAL TREATMENT/INTERVENTIONS

9.1 Introduction

The intervention being trialled is Endotracheal poractant alfa (Curosurf®, porcine lung phospholipid fraction supplied at 80mg/mL).

Curosurf® is a standard natural surfactant treatment prepared from porcine lungs for intratracheal administration and containing almost exclusively polar lipids, in particular phosphatidylcholine. Endotracheal poractant alfa is licensed for use in the UK for the treatment of Respiratory Distress Syndrome (RDS) or hyaline membrane disease in newborn babies (neonates). It is being used as an investigational medicinal product (IMP) on the BESS trial (UK licence, new indication).

Three small studies of surfactant use in infants with bronchiolitis have raised no safety concerns, however BESS will be the first large, randomised study of any surfactant for the treatment of bronchiolitis of infancy (3). As neonatal RDS is caused by surfactant deficiency, and the viral infections that cause bronchiolitis of infancy also cause surfactant deficiency (which we aim to treat with poractant alfa), we expect that the well tolerated safety profile of use in RDS in neonates will be reflected when using surfactant to treat bronchiolitis in infants.

Participants will be randomised equally to receive either endotracheal poractant alfa or air-placebo in a sham procedure. Participants randomised to receive endotracheal poractant alfa will receive a first dose of 200mg/kg, repeated 12 hourly at 100mg/kg, to a maximum of 3 doses. This is in line with the manufacturer's summary of product characteristics (SPC). Participants randomised to the placebo will receive an identical series of up to three procedures (while intubated) using air as the placebo.

9.2 Arm A – Endotracheal Poractant Alfa at 200mg/kg (first dose), with up to two further doses at 100mg/kg

9.2.1 Formulation, Packaging, Labelling, Storage and Stability

Generic name: Endotracheal poractant alfa

Brand name: Curosurf®

Supply and distribution: Chiesi Farmaceutici S.p.A (Chiesi) will supply Curosurf® via a third-party distributor and will provide appropriate certificates of analytical conformity. Additional shipments during each season will be managed by the distributor contracted by Chiesi, via an automated web-based system.

Packaging: Labelled holder containing one labelled glass vial with a blue topper.

Formulation: Each vial contains 3ml volume of poractant alfa at a concentration of 80mg/ml.

For peer review only - http://bmjopen.bmj.com/site/about/guidelines.xhtml

Labelling: Vials will be labelled by Chiesi in English and in compliance with the detailed guidance provided in annex 13 of the European Union (EU) Good Manufacturing Practice (GMP) guide. Each vial will be labelled and used for BESS trial use only.

Storage: The PI or delegated research team member will be responsible for the safe storage of all vials assigned to the BESS trial, in a safe place with restricted access (usually a designated drug room on the PICU). Poractant alfa will be stored between 2ºC and 8ºC, in a separate drug fridge specifically for use in the trial, protected from light in the original packaging.

Stability: Curosurf® has an unopened shelf life of 18 months. Each vial is for single use only.

9.2.2 Preparation, Dosage and Administration of Study Treatment/s

Dosage: An initial dose of 200mg/kg, with two further doses of 100mg/kg at 12hrs and 24hrs if indicated.

200mg/kg is 200milligrams of poractant alfa per kilogram of body weight. 100mg/kg is 100milligrams of poractant alfa per kilogram of body weight. The participant's weight will be detailed on the prescription in kilograms and the prescribing clinician will calculate the appropriate dose and number of vials needed for that participant.

Preparation: Poractant Alfa suspension is refrigerated, so before use each vial must be warmed up slowly to room temperature e.g. keeping it in hands for 5-10 minutes and gently turned upside down, without shaking, avoiding the formation of foam. Artificial warming methods should not be used.

The entire contents of the vials of poractant alfa suspension should be slowly withdrawn into a plastic syringe through a large-gauge needle and the excess poractant alfa discarded. A second person must be witness to the drawing up of the poractant alfa to assist and to check that the volume drawn up is the amount prescribed. Once the prescribed volume has been drawn into the syringe, an opaque paper shield should be applied to the syringe in order to maintain blinding of staff and/or parents at the bedside.

At this stage the appropriate equipment should also be prepared for conducting tracheal toilet and BAL.

Tracheal toilet (and, where scheduled, BAL) must be conducted prior to administration of the intervention.

The use of muscle relaxant prior to tracheal toilet, BAL and or administration of the intervention is at clinical discretion.

Administration: Either with the infant connected to the ventilator, or momentarily disconnected with hand-bagging, the syringe filled with Poractant Alfa suspension will be connected to a 5-French catheter end-hole inserted into the endotracheal tube (ETT) for ventilation. Passing the catheter through the suction or side port into the ETT and advancing the catheter aiming to have the tip of the catheter placed just beyond the tip of the ETT. The insertion length should be calculated from the known length of the ETT (typically cut to reduce dead space) plus the distance from the end of the cut edge of the ETT to the suction/side port). The intervention will be given slowly as a single bolus directly into the lower trachea (recommended over 0.5 to 3 minutes). From there it disperses through ventilation cycles into the peripheral lung tissue where the excipient (buffered 0.9% sodium chloride) is absorbed in the alveoli and the surfactant phospholipid is deposited.

For peer review only - http://bmjopen.bmj.com/site/about/guidelines.xhtml

The infant should be stabilized before surfactant administration and their head placed in the neutral position (head and body in alignment without inclination).

Before starting the procedure and throughout it, infants must have cardiorespiratory monitoring: oxygen saturation and heart rate continuously through pulse oximetry, blood pressure measurement either continuously in presence of an arterial catheter or intermittently if no arterial line is in place.

The first dose at 200 mg/kg will be followed up by two further doses at 100 mg/kg at approximately 12-hour intervals and the exact time and actual volume of each administration will be noted in the medical notes and appropriate section of the CRF.

No suctioning of the airways should be performed for two hours after each surfactant instillation unless the treating physician deems this appropriate, in which case this will be noted in the medical notes and appropriate section of the CRF.

9.2.3 Specific Restrictions

The effect of co-administration of poractant alfa with other agents is not known. Do not administer poractant alfa concomitantly in the same intratracheal bolus with other agents.

9.2.4 Overdose

Overdose may occur if the wrong patient weight has been supplied to the prescribing clinician, an incorrect weight is used in the calculation or the calculation has been done incorrectly. There have been no reports of overdose following the administration of Curosurf® however, in the unlikely event of an accidental overdose that causes clear clinical effects on the infant's respiration, ventilation or oxygenation, as much of the suspension should be aspirated as possible, as per the SPC.

Special care must be taken by all site team members to avoid overdosing, noticeably the prescribing clinician and the staff members drawing up the poractant alfa for administration. In the event of identification of an overdose, unblinding should occur and the participant will be closely observed for any adverse events (AE). The site team should notify the CTRC of the event and examine the reasons for the event occurring to ensure that it does not happen again. The CTRC will log any overdosing events as protocol deviations.

9.2.5 Temperature Compliance

Temperature deviations at site will be identified by the regular completion of a trial-specific temperature monitoring log. The PI or delegated member of staff will check the fridge used to store stocks of poractant alfa for use in BESS **daily** and will alert the CTRC if a temperature deviation has occurred. Temperature checks will be recorded on a Temperature Monitoring Log.

Poractant Alfa shipment and storage conditions:

**Poractant Alfa (Curosurf®)**<br>Suspension 80 mg/ml to 3ml vial	+2°C to +8°C	Up to +25°C for maximum cumulative 96hrs

Unopened vials of Curosurf® that have warmed to room temperature can be returned to refrigerated storage **within 24hours** for future use, **if** this is the first time that they have warmed. Vials that have been removed from the fridge and been allowed to warm to room temperature for a

For peer review only - http://bmjopen.bmj.com/site/about/guidelines.xhtml

second time **must not be used**, but should be retained for accountability checks at the end of each season.

Temperature compliance during shipment will be checked by the local site Pharmacist on receipt of the IMP. The local Pharmacist will review the temperature curves/logs from the calibrated temperature logger used during IMP shipment and will check for any out of range values. If an out of range temperature is identified, the local Pharmacist will inform the CTRC within 48 hours. It is the responsibility of the CTRC to inform Chiesi, who will then notify the Chiesi R&D Quality Assurance GMP Unit & Project Partners (R&D QA-GMP).

If the out-of-range temperature deviation does not affect the quality and safety of the products, a declaration of usability will be issued and signed by R&D QA-GMP and sent back to the CTRC. CTRC will then authorise the trial site to use the vials in question.

If the products are judged not suitable for use, Chiesi R&D QA-GMP will inform the CTRC to permanently suspend the use of the affected vials. Any vials not used due to temperature deviations will be retained for accountability checks at the end of each season before being sent for destruction.

For peer review only

9.2.6 Accountability Procedures for Trial Treatments

The PI is responsible for the management of poractant alfa to be used for the trial. Trial IMP should be stored in the original packaging to protect from light, in a locked, secure, refrigerated storage facility with access limited to those individuals authorized to dispense or administer the trial IMP. Initial supply of the trial IMP will be to the site Pharmacy. The site Pharmacists are responsible for dispensing stock to a secure drug room on the PICU for more immediate access by the research team. Pharmacists must release the trial IMP to PICU in sequential order by the medication numbers on the labels to ensure the Randomisation System can allocate trial IMP with the shortest shelf life first. No surfactant material supplied for this trial is to be used for any other purpose.

Accountability logs will be maintained by the PI, pharmacist or delegated research team member, which will include a signed account of all the vials of Curosurf® received from the distributor, the vials dispensed to and returned from PICU, with the vials and volume used for each randomised patient.

All completely used vials will be retained at the site until the conclusion of the recruitment season. Each vial is for single use only. Any leftover unused medication is to be retained for drug accountability. Partially used vials present a risk of confusion and sharps injury, so all partially used vials will be disposed of within the PICU as per local Trust standard procedures. Disposal of partially used vials will be logged for accountability checks.
At the conclusion of each recruitment season, the PI, pharmacist or delegated individual shall conduct and document a drug supply (used and unused) inventory. An explanation will be given for any discrepancies.

After the inventory is reconciled at the end of each season and only once the CTRC have given the site authorisation on behalf of the sponsor, remaining unused vials of Curosurf® maybe disposed of directly by the Pharmacy of the involved trial site. Unused vials with an expiry date during or after

For peer review only - http://bmjopen.bmj.com/site/about/guidelines.xhtml

the next season will be retained. Documentation assuring the unused vials were sent off-site for destruction, as per standard Trust procedures will be provided. This must be signed by two delegated members of staff filed in the Pharmacy site file, with a copy returned to the CTRC for storage in the Trial Master File.

9.2.7 Assessment of Compliance with Study Treatment/s

Each vial of Curosurf® will be designated by code number and the actual volume of instilled test treatment will be noted in the hospital chart and on the CRF of the study.

The completely used vials will be kept in a sealed labelled plastic box/envelope with patient's randomisation number/initials until the end of the season for accountability checks. There will therefore be three "final" accountability checks at each site, one at the end of each recruitment season.

9.3 Arm B – Placebo

Placebo: Air via an identical series of 3 doses in a sham procedure.

Dosage and preparation: Air should be drawn into a syringe at the same volume of 200mg/kg for the first dose and 100mg/kg for the second and third doses (if required), as per the instructions for poractant alfa dose calculations assuming the intervention is provided at 80mg/ml (section 9.2.2). Once the prescribed volume has been drawn into the syringe, an opaque paper shield should be applied to the syringe in order to maintain blinding of staff and/or parents at the bedside.

The use of muscle relaxant prior to tracheal toilet, BAL and or administration of the intervention is at clinical discretion.

Administration: Either with the infant connected to the ventilator, or momentarily disconnected with hand-bagging, the syringe filled with air will be connected to a 5-French catheter end-hole inserted into the endotracheal tube (ETT) for ventilation. Passing the catheter through the suction or side-port into the ETT and advancing the catheter aiming to have the tip of the catheter placed just beyond the tip of the ETT. The insertion length should be calculated from the known length of the ETT (typically cut to reduce dead space) plus the distance from the end of the cut edge of the ETT to the suction/side port). The air will be given slowly as a single bolus directly into the lower trachea (recommended over 0.5 to 3 minutes).

9.4 Unblinding

N.B. Allocation must not be routinely revealed to CTRC personnel. It is to be expected that some site staff may be accidentally unblinded due to back-tracking of surfactant up the participant's ETT (see section 7.4).

As the treatment for any adverse event potentially associated with the study intervention would be the same whether the patient had been given surfactant or air, there will be no emergency unblinding

For peer review only - http://bmjopen.bmj.com/site/about/guidelines.xhtml

procedure for BESS. In the case of any adverse event, sites should assume all patients have been given poractant alfa (Arm A) and administer treatment accordingly. Any adverse events should be reported as per the procedures described in section 10.

9.4.1.1 Accidental unblinding

If accidental unblinding occurs, this must be reported to the CTRC by use of the Unblinding CRF. When reporting include details about:

1. Date of unblinding;
2. Detailed explanation of circumstances;
3. Recipients of the unblinding information;
4. Action to prevent further occurrence, if possible.

9.4.1.2 Unblinding at Trial Closure

Upon trial closure the blinding of parents and blinded staff remains in effect. Site teams and/or pharmacy departments **should not** disclose treatment allocations on an individual basis.

9.5 Concomitant Medications

No incompatibilities or interactions of Curosurf® with other medications are known. There are therefore no restrictions placed by the trial on the use of concomitant medications in BESS participants.

For peer review only - http://bmjopen.bmj.com/site/about/guidelines.xhtml

10 SAFETY REPORTING

10.1 Terms and Definitions

"Adverse Event (AE)"

An adverse event (AE) is any untoward medical occurrence in a subject to whom a medicinal product has been administered, including occurrences which are not necessarily caused by or related to that product.

Therefore an AE is any unfavourable or unintended change in the function (symptoms), structure (signs) or chemistry (laboratory data) in a subject to whom an IMP has been administered, including occurrences which are not necessarily caused by or related to that product.

"Adverse Reaction (AR)"

An adverse reaction (AR) is any untoward and unintended response in a subject to an IMP that is related to any dose administered to that subject.

Therefore an AR is any unfavourable or unintended change in the function (symptoms), structure (signs) or chemistry (laboratory data) in a subject that is related to any dose of an IMP administered to that subject.

"Unexpected Adverse Reaction (UAR)"

An unexpected adverse reaction (UAR) is an adverse reaction the nature and severity of which is not consistent with the information about the medicinal product in question set out in the Investigator's Brochure (IB) or the SPC, which may be referenced where the IMP in question is a product with a marketing authorisation.

"Serious Adverse Event (SAE), "Serious Adverse Reaction, or Unexpected Serious Adverse Reaction"

A serious adverse event (SAE) is an AE, AR or UAR respectively that:

- results in death;
- is life threatening (places the subject, in the view of the Investigator, at immediate risk of death from the experience as it occurred – this does not include an adverse experience that, had it occurred in a more severe form, might have caused death);
- requires hospitalisation or prolongation of existing hospitalisation (hospitalisation is defined as an in-patient admission, regardless of length of stay, even if the hospitalisation is a precautionary measure for continued observation – hospitalisations for a pre-existing condition, including elective procedures that have not worsened, do not constitute an SAE);
- results in persistent or significant disability or incapacity (substantial disruption of one's ability to conduct normal life functions);
- consists of a congenital anomaly or birth defect (in offspring of subjects, or their partners, taking the IMP regardless of time of diagnosis);
- other important medical events (these may not result in death, be life-threatening, or require hospitalisation, but may be considered a serious adverse event or experience when, based upon appropriate medical judgment, they are considered to jeopardise the subject and may require medical or surgical intervention to prevent one of the outcomes listed in this definition).

For peer review only - http://bmjopen.bmj.com/site/about/guidelines.xhtml

"Suspected Serious Adverse Reaction (SSAR)"

A suspected serious adverse reaction (SSAR) is an adverse reaction that is classed in nature as serious and which is consistent with the information about the IMP in question, which in the case of a licensed product is set out in the SPC for that product, and in the case of any other IMP is set out in the IB relating to the trial in question.

"Suspected Unexpected Serious Adverse Reaction (SUSAR)"

A suspected unexpected serious adverse reaction (SUSAR) is an adverse reaction that is classed in nature as serious and which is not consistent with the information about the IMP in question, which in the case of a licensed product is set out in the SPC for that product, and in the case of any other IMP is set out in the IB relating to the trial in question.

"Reference Safety Information (RSI)"

The reference safety information (RSI) for a trial is the information used for assessing whether an adverse reaction is expected. This is contained in either the IB or the SPC.

10.2 Notes on Adverse Event Inclusions and Exclusions

The below sections provide guidance on what should, and should not be considered as AEs for the purposes of the BESS study:

10.2.1 Include:

- An exacerbation of a pre-existing illness, **excluding** the current case of bronchiolitis.
- An increase in frequency or intensity of a pre-existing episodic event/condition.
- A condition (even though it may have been present prior to the start of the trial) detected after trial drug administration.
- Continuous persistent disease or symptoms present at baseline that worsen following the administration of the trial treatment, **excluding** the current case of Bronchiolitis.
- Laboratory abnormalities that require clinical intervention or further investigation (unless they are associated with an already reported clinical event).
- Abnormalities in physiological testing or physical examination that require further investigation or clinical intervention.
- Injury or accidents.

10.2.2 Do Not Include:

- Widely recognised and documented clinical features of bronchiolitis or outcomes associated with MV that are defined outcome measures in this trial (see section 3.5).
- Medical or surgical procedures* – the condition which leads to the procedure is the adverse event; e.g. pneumothorax leading to chest drain placement, here pneumothorax is the event.
- Pre-existing disease or conditions present before treatment that do not worsen.
- Situations where an untoward medical occurrence has occurred, e.g. cosmetic elective surgery.
- Overdose of medication without symptoms or signs**.

For peer review only - http://bmjopen.bmj.com/site/about/guidelines.xhtml

** Cosmetic elective surgery is cited here as example of an event that is not reportable as an AE.*
** See also section 9.2.4. *If overdose occurred with resulting symptoms and signs that met the protocol criteria for AE/AR/SAE/SAR then they should be reported accordingly. Please note that though overdose of medication without symptoms or signs may be excluded from AE reporting this may still require investigation to ensure the protocol and regulatory requirements are met, e.g. for IMP management and administration to ensure participant safety.*

10.2.3 Notification of deaths

There is an overall likelihood of death in all cases of severe bronchiolitis of 1-2%. All deaths that occur during the protocol-specified AE reporting period, regardless of relationship to study drug, must be recorded. All deaths should be reported on a “Serious Adverse Event CRF” and returned to the CTRC within 24 hours.

Death should be considered an outcome and not a distinct event. The event or condition that caused or contributed to the fatal outcome should be recorded as the single medical concept on the appropriate CRF; e.g. death following pneumothorax, here pneumothorax is the event. Generally, only one such event should be reported. The term “sudden death” should only be used for the occurrence of an abrupt and unexpected death due to presumed cardiac causes in a participant with or without pre-existing heart disease, within 1 hour of the onset of acute symptoms or, in the case of an unwitnessed death, within 24 hours after the participant was last seen alive and stable. If the cause of death is unknown and cannot be ascertained at the time of reporting, “unexplained death” should be recorded on the appropriate CRF. If the cause of death subsequently becomes available (e.g. after post-mortem examination), “unexplained death” should be replaced by the established cause of death.

10.3 Notes on Severity / Grading of Adverse Events

The assignment of the severity/grading should be made by the investigator responsible for the care of the participant using the definitions below.

Regardless of the classification of an AE as serious or not, its severity must be assessed according to medical criteria alone using the following categories:

Mild: does not interfere with routine activities

Moderate: interferes with routine activities

Severe: impossible to perform routine activities

A distinction is drawn between serious and severe AEs. Severity is a measure of intensity (see above) whereas seriousness is defined using the criteria in section 10.1, hence, a severe AE need not necessarily be a SAE.

For peer review only - http://bmjopen.bmj.com/site/about/guidelines.xhtml

10.4 Relationship to Trial Treatment

The assignment of the causality should be made by the investigator responsible for the care of the participant using the definitions in Table 3: Definitions of Causality. An AE for which the causal relationship to the study drug is assessed by the investigator as "possible", "probable" or "almost certain" is an AR.

If any doubt about the causality exists the local investigator should inform the CTRC who will notify the Chief Investigator (CI). In the case of discrepant views on causality between the investigator and others, the MHRA will be informed of both points of view.

Table 3: Definitions of Causality

Relationship	Description
**Unrelated**	There is no evidence of any causal relationship. N.B. An alternative cause for the AE should be given.
**Unlikely**	There is little evidence to suggest there is a causal relationship (e.g. the event did not occur within a reasonable time after administration of the trial medication). There is another reasonable explanation for the event (e.g. the participant's clinical condition, other concomitant treatment).
**Possibly**	There is some evidence to suggest a causal relationship (e.g. because the event occurs within a reasonable time after administration of the trial medication). However, the influence of other factors may have contributed to the event (e.g. the participant's clinical condition, other concomitant treatments).
**Probably**	There is evidence to suggest a causal relationship and the influence of other factors is unlikely.
**Almost certainly**	There is clear evidence to suggest a causal relationship and other possible contributing factors can be ruled out.

10.5 Expectedness

The Reference Safety Information (RSI) in BESS is sections 4.3 to 4.9 (inclusive) of the Curosurf® Endotracheopulmonary Instillation Suspension SPC.

It is not a regulatory requirement for a reporting physician to provide their opinion of expectedness. Therefore, the reporting physician at the local research site will not be asked to make an assessment of expectedness. The assessment of expectedness will be made by the CI (or their medically qualified delegate) using BESS's current MHRA-approved RSI following receipt of the "Serious Adverse Event CRF" at CTRC.

All events judged by the investigator to be possibly, probably, or almost certainly related to the IMP, graded as serious and **unexpected** (see current MHRA-approved trial RSI (Curosurf® Endotracheopulmonary Instillation Suspension SPC) for list of Expected Adverse Events) should be reported as a SUSAR.

For peer review only - http://bmjopen.bmj.com/site/about/guidelines.xhtml

10.6 Follow-up After Adverse Events

All adverse events should be followed until satisfactory resolution or until the investigator responsible for the care of the participant deems the event to be chronic or the participant to be stable.

When reporting SAEs and SUSARs the investigator responsible for the care of the participant should apply the following criteria to provide information relating to event outcomes:

- resolved;
- resolved with sequelae (specifying with additional narrative);
- not resolved/ongoing;
- on-going at final follow-up;
- fatal or unknown.

For peer review only

10.7 Time Period for Safety Reporting

Investigators and delegated members of the local research teams will report to CTRC on the appropriate CRFs (see section 10.8):

- all adverse events from randomisation until 24 hours after their final intervention (whether poractant alfa or placebo);
- all adverse reactions from randomisation until 90 days post-randomisation or hospital discharge (whichever is sooner).

Following these periods, site staff are not required to report any non-serious events, however any serious events (SAEs/SARs/SUSARs) which they become aware of must be reported to CTRC.

Upon becoming aware of a serious adverse event (SAE/SAR/SUSAR), the Investigator or other delegated member of the team **must** report this to the CTRC **within 24 hours**.

10.8 Reporting Procedures

All serious events (SAEs/SARs/SUSARs) must be reported in an expedited manner. Depending on the nature of the event the reporting procedures below should be followed. Any questions concerning adverse event reporting should be directed to the CTRC in the first instance. If a site has a serious or urgent query regarding an adverse event outside of CTRC office hours they should contact the Chief Investigator, Prof. Calum Semple, on 07506 656 560.

A flowchart is given below to aid in determining reporting requirements.

For peer review only - http://bmjopen.bmj.com/site/about/guidelines.xhtml

10.8.1 Flowchart for Reporting Requirements of Adverse Events

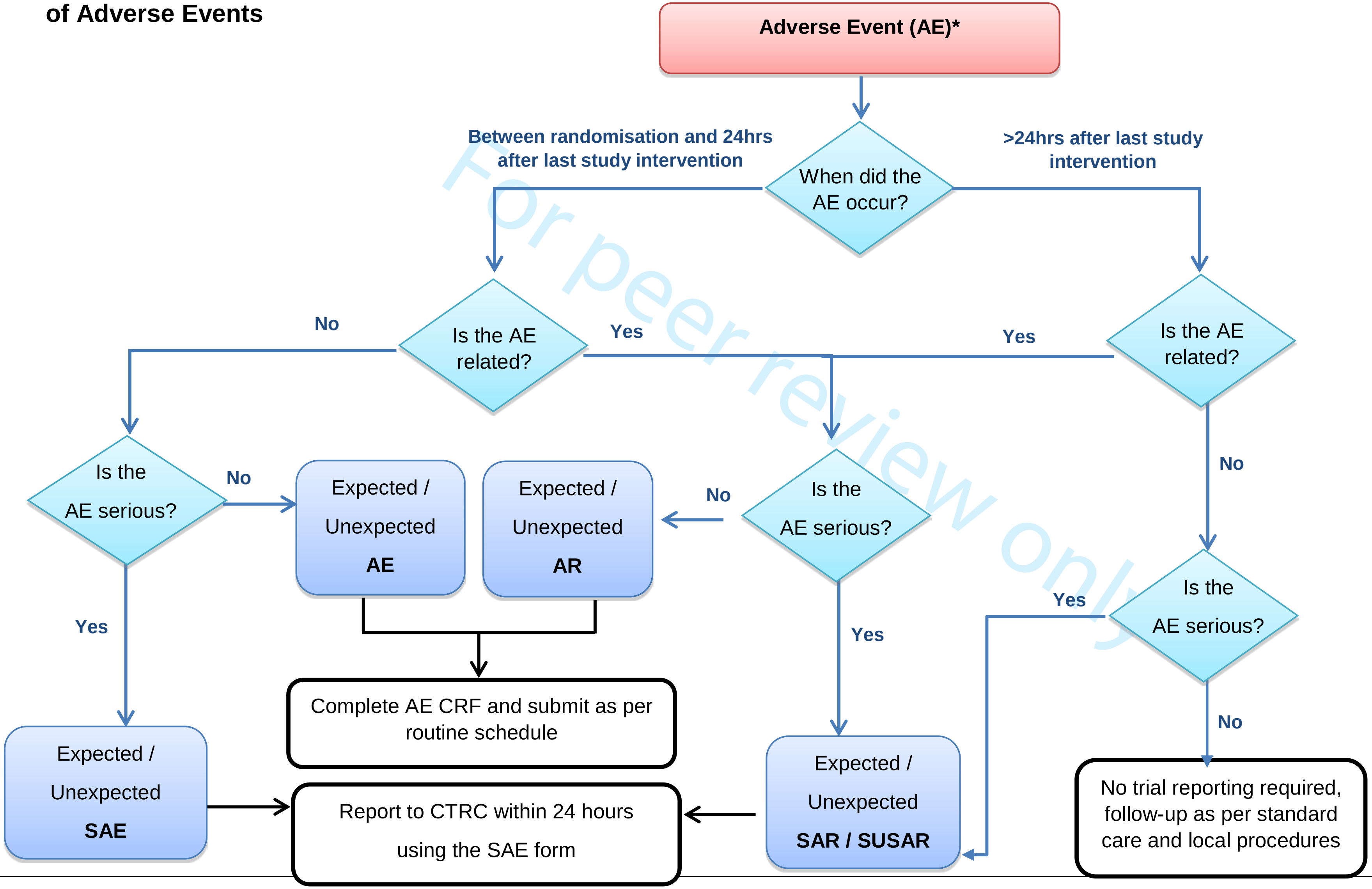

For peer review only - http://bmjopen.bmj.com/site/about/guidelines.xhtml

For peer review only

For peer review only - http://bmjopen.bmj.com/site/about/guidelines.xhtml

10.8.2 Non serious events (ARs/AEs)

All non-serious events (i.e. AEs and ARs), whether expected or not, should be recorded on an “Adverse Event CRF”, which should be transmitted to the CTRC within seven days of the local research team becoming aware of the event.

10.8.3 Serious events (AEs/ARs/SUSARs)

All serious events (i.e. SAEs, SARs and SUSARs) must be reported to CTRC within 24 hours of the local site becoming aware of the event. The “Serious Adverse Event CRF” asks for the nature of the event, date of onset, severity, corrective therapies given, outcome and causality. The reporting local Investigator should assign the causality of the event. Additional information should be sent within 5 days if the event has not resolved at the time of initial reporting.

All adverse events assessed as serious, related and unexpected (i.e. SUSARs) will be reported on to the MHRA and BESS’s Research Ethics Committee (REC) by the CTRC according to the following timelines; fatal and life-threatening SUSARs within 7 days of notification to CTRC and non-life threatening SUSARs within 15 days. BESS PIs will be notified of all SUSARs occurring throughout the trial by the CTRC. Local Investigators should report any SUSARs and/or SAEs/SARs as required locally.

NOTE: *please ensure that multiple Serious Adverse Events are reported separately to the CTRC. Each individual SAE report should relate to one overall diagnosis only.*

10.9 Safety Reporting Responsibilities – Investigator

The local site Investigator (site Principal Investigator (PI)) is responsible for reporting all events that are observed or reported during the study, regardless of their relationship to study product (i.e. AEs/ARs/SAEs/SARs/SUSARs)..

All serious adverse events (SAEs/SARs/SUSARs) must be reported immediately by the investigator to the CTRC on an SAE. All non-serious adverse events should be reported to CTRC within seven days of the local research team becoming aware on an AE CRF.

Minimum information required for reporting serious events

- Valid European Clinical Trials Database (EudraCT) number (if applicable)
- Sponsor study number
- One identifiable coded subject (randomisation number)
- One identifiable reporter (name of PI or appropriate delegated individual)
- One SAE
- One suspect IMP (poractant alfa)
- A seriousness assessment
- A causality assessment

For peer review only - http://bmjopen.bmj.com/site/about/guidelines.xhtml

Process for reporting serious events

i. The SAE CRF should be completed by a local Investigator, named on the 'signature list and delegation of responsibilities log' as responsible for reporting SAEs and making trial related medical decisions. The Investigator should assess the SAE for the likelihood that it is a response to the trial intervention. In the absence of the designated Investigator the SAE CRF should be completed and signed by an alternative member of the research site trial team and submitted to the CTRC. As soon as possible thereafter the responsible Investigator should check the SAE CRF, make amendments as appropriate, sign and re-send to the CTRC. The initial report shall be followed by detailed follow-up CRF reports as appropriate (see number vii).

ii. The "Serious Adverse Event CRF" **must** be submitted to the CTRC **within 24 hours** of the local research team becoming aware of the event; preferably this will be via fax.

Email address: bess@liverpool.ac.uk

Fax Number: 0151 795 8770

iii. When submitting a SAE CRF to the CTRC research sites must also telephone the appropriate Trial Co-ordinator or Data Manager on telephone number **0151 795 8757** to advise that an SAE CRF has been submitted.

iv. The participant **must** be identified by trial randomisation number, date of birth and initials **only**. The participant's name **must not** be used on any correspondence.

v. The local reporting Investigator must **notify** their R&D department of the event (as per standard local governance procedures).

vi. The participant must be followed-up until clinical recovery is complete and laboratory results have returned to normal, or until the event has stabilised. Follow-up should continue after completion of protocol treatment as necessary.

vii. Follow-up information should be noted on a separate CRF by ticking the box marked 'follow-up' and submitting to the CTRC as information becomes available. Extra, annotated information and/or anonymised copies of test results may be provided separately.

10.9.1 Maintenance of Blinding

Systems for SUSAR and SAR reporting should, as far as possible, maintain blinding of individual clinicians and of trials staff involved in the day-to-day running of the trial. The medical management of participants for adverse events should be undertaken under the assumption that the patient has been given surfactant (Arm A). The safety of participants in the trial always takes priority. In each report, seriousness, causality and expectedness should be evaluated for poractant alfa unless unblinding has taken place. All instances of unblinding should be reported to the CTRC.

Cases that are considered serious, unexpected and possibly, probably or almost certainly related to one of the trial therapies (i.e. SUSARs) would have to be unblinded at the CTRC prior to reporting to the MHRA and REC.

For peer review only - http://bmjopen.bmj.com/site/about/guidelines.xhtml

10.10 Safety Reporting Responsibilities – CTRC

The CTRC is undertaking duties delegated by the trial Sponsor and is responsible for the notification of serious events (SAEs/SARs/SUSARs) to the CI (or appropriate delegate) for review and expectedness assessment, and onward expedited reporting of SUSARs and other SARs to the MHRA and REC. The CTRC will also notify PIs at participating sites of all trial SUSARs.

Upon receipt of a SAE CRF, CTRC will liaise with the trial CI (or appropriate delegate) who will review the events reported for seriousness and causality as assessed by the local site research team, and provide and assessment of expectedness. These will be reviewed within 24 hours of receipt at CTRC and those that are identified as SUSARs will be reported to MHRA, REC and Chiesi Farmaceutici S.p.A. by the CTRC. The causality assessment given by the local Investigator at the hospital cannot be overruled and in the case of disagreement, both opinions will be provided with the report.

Timelines of onward reporting to MHRA and REC are as follows:

- SUSARs that are fatal or life-threatening must be reported not later than 7 days after the CTRC is first aware of the reaction. Any additional relevant information must be reported within a further 8 days.
- SUSARs that are not fatal or life-threatening must be reported within 15 days of the CTRC first becoming aware of the reaction.
- A list of all SARs (expected and unexpected) must be reported annually.

The CTRC will also inform Chiesi Global Pharmacovigilance (Parma, Italy) of any adverse event identified as a SUSAR, within 7 days of the CTRC first becoming aware of the reaction.

It is recommended that the following safety issues should also be reported in an expedited fashion:

- an increase in the rate of occurrence or a qualitative change of an expected SAR, which is judged to be clinically important;
- new events related to the conduct of the trial or the development of the IMP and likely to affect the safety of the participants, such as:
    a. a SAE that could be associated with the trial procedures and which could modify the conduct of the trial;
    b. a significant hazard to the participant population, such as lack of efficacy of an IMP used for the treatment of a life-threatening disease;
    c. a major safety finding from a newly completed animal study (such as carcinogenicity);
    d. any anticipated end or temporary halt of a trial for safety reasons and conducted with the same IMP in another country by the same sponsor;
- recommendations of the IDSMC, if any, where relevant for the safety of the participants.

For peer review only - http://bmjopen.bmj.com/site/about/guidelines.xhtml

Participant safety incidents that take place in the course of research should be reported to the National Reporting and Learning System (NRLS) by each participating NHS Trust in accordance with local reporting procedures.

10.11 Safety reports

Safety reports will be generated during the course of the trial which allows for monitoring of serious events (SAEs/SARs/SUSARs)reporting rates across sites. The CTRC will send developmental safety update reports (DSUR) containing a list of all SARs to MHRA and the trial's main REC. Any concerns raised by the IDSMC or inconsistencies noted at a given site may prompt additional training at sites, with the potential for the CTRC to carry out site visits if there is suspicion of unreported AEs in patient case notes. Additional training will also be provided if unacceptable delay in safety reporting timelines. If any safety reports identify issues that have implications for the safety of trial participants, the PIs at all institutions participating in the trial will be notified.

10.12 Urgent Safety Measures

An urgent safety measure is a procedure not defined by the protocol, which is put in place prior to authorisation by the MHRA and REC in order to protect clinical trial participants from any immediate hazard to their health and safety.

The CTRC will notify the MHRA and REC immediately and, in any event, within 3 days that such a measure has been taken and the reasons why it has been taken. The initial notification to the MHRA will be by telephone (ideally within 24 hours) and a notice in writing will be sent within 3 days, setting out the reasons for the urgent safety measure and the plan for further action. After discussion with the MHRA and REC, further action will be agreed, which may include submission of a substantial amendment, a temporary halt, or permanent termination of the trial.

If the study is temporarily halted it may not recommence until authorised to do so by the MHRA and REC. If the study is permanently terminated before the date specified for its conclusion (in the original applications to MHRA and REC), the CTRC should notify the MHRA and REC within 15 days of the date of termination by submitting the formal End of Trial Notification.

10.13 Contact Details and Out-of-hours Medical Cover

Participants will be instructed to contact the local Investigator (their office number) during working hours. All local PIs should ensure to delegate at least one other appropriately trained (in the study and in GCP) medically qualified doctor for assessment of adverse events, safety reporting and making medical decisions in their absence.

Outside working hours the normal local procedure should be followed to contact the local site PI or their agreed delegate. PICU, ward and local switchboard staff should be aware where they should direct calls in- and out-of-hours.

The Chief Investigator has delegated two medically qualified and appropriately experienced doctors to assess safety reports in his absence (Prof Paul McNamara & Dr Kent Thorburn).

For peer review only - http://bmjopen.bmj.com/site/about/guidelines.xhtml

The CTRC should be the main point of contact for trial questions during opening hours – 0151 795 8757. Out-of-hours cover for trial related clinical issues that require urgent attention will be provided by the Chief Investigator or his delegates, on 07506 653560.

For peer review only

For peer review only - http://bmjopen.bmj.com/site/about/guidelines.xhtml

11 STATISTICAL CONSIDERATIONS

11.1 Introduction

Separate Statistical Analysis Plans (SAPs) will be developed prior to the interim and final analyses of the trial.

11.2 Method of Randomisation

Participants will be randomised to receive either poractant alfa or air placebo in a 1:1 ratio. The randomisation list will be generated by an independent statistician at the CTRC who is not otherwise involved in the BESS trial. Randomisation lists will be computer-generated using block randomisation with random variable block length, stratified by site and duration of ventilation (<24hrs and >=24hrs) prior to randomisation.

11.3 Sample Size calculation

The study will be powered to detect a minimally clinically important difference of 18 hours (0.75 days) in the primary outcome variable of time of mechanical ventilation. Data are expected to be positively skewed, and will therefore be log-transformed, and resulting data back-transformed. The null hypothesis is that of no difference in time of MV between the two groups.

Data from Alder Hey Children's Hospital from an audit of 60 eligible infants admitted to PICU in the seasons 2012/3 and 2013/4 showed a mean (SD) duration of ventilation of 4.83 (2.5) days (PICANet data, 2011-14). Parameters for sample size estimation are therefore as follows:

$E(Y_1) = 4.83$ and $SD(Y_1) = 2.5$

$E(Y_2) = 4.08$ and $SD(Y_2) = 2.5$

$\delta = E(Y_2)/E(Y_1) = 0.845$

giving coefficients of variation:

$\kappa_1 = SD(Y_1)/E(Y_1) = 2.5/4.83 = 0.518$

$\kappa_2 = SD(Y_2)/E(Y_2) = 2.5/4.08 = 0.613$

Using the formula of Wolfe and Carlin (1999) with $r = 1$ for equal allocation and a two-sided alpha-level of 0.05, sample size estimations are 100 per group for 80% power and 134 per group for 90% power.

Taking the sample size of 134 and allowing for 5% dropout requires 142 patients will be recruited to each group, giving a total of 284 participants overall.

For peer review only - http://bmjopen.bmj.com/site/about/guidelines.xhtml

11.4 Interim Monitoring and Analyses

Analyses of the accumulating data will be performed at regular intervals (at least annually) for review by an IDSMC. These analyses will be performed at the CTRC. The IDSMC will be asked to give advice on whether the accumulated data from the trial, together with results from other relevant trials, justifies continuing recruitment of further patients or further follow-up. The IDSMC will make recommendations to the Trial Steering Committee (TSC). A decision to discontinue recruitment, in all participants or in selected subgroups will be made only if the result is likely to convince a broad range of clinicians including participants in the trial and the general clinical community.

11.5 Analysis Plan

A full SAP will be written prior to the conduct of any comparative analysis of the treatment arms. The main features of the SAP are included here.

The primary analysis will be a test of the difference in geometric mean durations of MV between groups (placebo vs. poractant alfa), reported with the corresponding 95% confidence interval. The analysis will be conducted according to the intent-to-treat principle, and will incorporate the stratification factors of duration of ventilation (<24hrs and >=24hrs) prior to randomisation and site.

For the secondary outcomes, longitudinal data for VI, OI, OSI and SF will be analysed with joint modelling. LRSQ data will be modelled longitudinally and comparisons made between groups. Binary data will be reported in terms of relative risk with 95% confidence intervals.

Within BESS, data will be collected as to ascertain readiness for spontaneous breathing test and time of extubation.

As much information as possible will be collected about the reasons for missing outcome data; this will be used to inform any imputation approaches employed in the analysis. Such methods will be fully described in the SAP.

For peer review only

For peer review only - http://bmjopen.bmj.com/site/about/guidelines.xhtml

12 EXPLORATORY & TRANSLATIONAL WORK; WORK PACKAGES B AND C

12.1 Work Package B: Study of Parent & Staff Experiences

BESS will involve an embedded mixed method sub-study involving parents and BESS recruiters involved in recruitment and consent processes. WP-B will explore: acceptability of the trial; approach to recruitment and consent; decision making in the emergency setting; barriers to participation; and the content and format of trial information materials. This will involve: questionnaires and interviews with parents approached for informed consent in BESS, including those who do not go on to be randomised into the trial, and a survey of BESS recruiters.

Parent recruitment in season 1

In season 1, all parents approached for informed consent in BESS will be asked to complete a brief questionnaire and/or take part in a telephone interview. Recruitment to the sub study will occur shortly after the main trial recruitment discussion. This will include parents who decline to provide informed consent for the trial. If more than one parent is involved in the consent discussion, then both are able to complete a questionnaire. By completing the questionnaire, this confirms that the parent has consented to this providing information.

Parents will be asked to place completed questionnaires in a sealed stamped addressed envelope and return it to the BESS recruiter who will post to the research team. Recruitment for questionnaires will take place throughout season 1. In the rare instance that consent is not sought prior to discharge/transferred to another hospital, BESS recruiters should send the questionnaire to parents along with the Parent/Person with PISC to complete.

BESS recruiters will ask the parent to provide contact details on the BESS consent form if they wish to take part in a telephone interview. A researcher will contact families to arrange telephone interviews within one month of consent. At this point parents who live in (or close to) the Merseyside area will be provided with the option of a face to face interview at their home if they prefer. All families who express an interest in taking part but are not selected for an interview will be contacted via telephone or email to thank them for their interest in the study.

All interviews will be conducted by a researcher who both have proven skills in the conduct of research in sensitive settings. Any distress during the interviews will be managed with care and compassion and participants will be free to decline to answer any questions that they do not wish to answer or to stop the interviews at any point. Any such families will be supported in obtaining appropriate help and, after discussion with the family, the lead clinician responsible for the child's care will be informed to offer any support. Interviews will be conducted until data saturation point is achieved (approximately 15-25 based on other studies). All interview participants will be sent a £20 shopping voucher to thank them for their time. Sites will be informed when recruitment to interviews can stop.

For peer review only - http://bmjopen.bmj.com/site/about/guidelines.xhtml

Parent recruitment in season 2

In season 2, all parents will be asked to complete a brief questionnaire. This will be the same questionnaire as used in season 1 and include parents who decline informed consent.

Questionnaire findings will help evaluate parents' views on recruitment and consent, including any changes made to our recruitment approach following season 1.

Staff recruitment in seasons 1 and 2

All staff involved in trial conduct, recruitment and consent in participating sites will be sent an email invitation and information sheet to request their participation in an online questionnaire towards the end of season 1 and 2. Completion of the questionnaire will be taken as an indication of consent.

Findings from parent questionnaires, interviews and staff surveys in season 1 will be used to develop approaches to consent, recruitment, trial information materials and staff training in season 2. Findings from season 2 parent and staff questionnaires will inform the design/training for the approach to recruitment and consent in season 3.

12.1.1 Parent and staff perspectives sub study analysis

Analysis of data from the mixed method sub study will be assisted using NVivo 8 qualitative data analysis package and SPSS software for statistical analysis. Quantitative analysis will involve simple descriptive statistics and the chi-square test for trend. Qualitative data from will be analysed thematically (32). Data from study methods will be analysed separately then synthesised (33) through the use of constant comparative analysis (34).

12.2 Work Package C:

Understanding the mechanism of efficacy or failure for exogenous surfactant to modulate bronchiolitis disease requires an integrated investigation of the subject's alveolar environment. This includes identifying the infective aetiology, describing the local inflammatory response, and defining the kinetics of surfactant lipid and protein synthesis; all linked to natural history, trial intervention and participant outcomes. The selected mechanistic sub studies leverage the unique collaboration between experts in bronchiolitis pathogenesis at Liverpool and surfactant physiology at Southampton. While the sub studies are defined individually, their outputs integrate to provide a cogent thesis.

Hypotheses to test include:

At all sites by analysis of BAL fluid sampled just prior to intervention at 0hr and 24hr:

- Surfactant concentration and composition predicts duration of MV.
- Recovery of synthesis of surfactant proteins A & D is modulated by exogenous surfactant.

For peer review only - http://bmjopen.bmj.com/site/about/guidelines.xhtml

- Lower airway inflammation is modulated by exogenous surfactant.[3]
- Treatment failure is associated with detection of specific pathogens.

At Liverpool & Southampton by giving a single intravenous dose of deuterium-labelled[4] choline (*methyl*-D_9-choline chloride (D_9-Choline)) and with sampling of BAL fluid and blood just prior to intervention at 0hr, 12hr, 24hr and 36hr.

- Synthesis of endogenous surfactant phosphatidylcholine is modulated by the provision of exogenous surfactant.

12.2.1 Metabolic profiling of surfactant composition and kinetics

Analysis of surfactant phospholipid and protein concentration, composition and synthesis in BAL fluid from ventilated infants will help provide an explanation for the beneficial effects, or otherwise, of exogenous surfactant therapy for acute lung injury in infants.

The sub-study of the metabolic profiling of surfactant composition and kinetics has two linked parts. The first "**Surfactant concentration and composition**" utilises BAL fluid collected as part of the whole trial at any site and does not involve any additional intervention or sampling procedures. The second part "**Kinetics of surfactant phosphatidylcholine synthesis"** is a more complex study that requires additional interventions, specific consent and consequently will recruit only from Liverpool and Southampton

12.2.1.1 Surfactant concentration and composition

This will address the hypothesis that: Surfactant concentration and composition predicts duration of MV.

Infants will be recruited to this part of the mechanistic sub-study from all BESS trial centres. Individual molecular species of phosphatidylcholine (PC), phosphatidylglycerol (PG) phosphatidylinositol (PI) and sphingomyelin (SM) will be analysed in aliquots of small volume BAL fluid samples taken prior to trial intervention at 0 and 24hr hours of the study if still intubated. These phospholipids will be analysed by electrospray ionisation mass spectrometry (ESI-MS) (29, 30) in Southampton correcting for sample dilution by urea analysis(28). Surfactant proteins A, B, C, and D will be analysed by ELISA and western blotting.

[3] Costs for this component of BESS will not be charged to NIHR EME.

[4] Deuterium is one of the two stable (non-radioactive) and naturally occurring isotopes of hydrogen that is used as a harmless metabolic tracer in animal and human studies.

For peer review only - http://bmjopen.bmj.com/site/about/guidelines.xhtml

12.2.1.2 Kinetics of surfactant phosphatidylcholine synthesis

This will address the hypothesis that: Synthesis of endogenous surfactant phosphatidylcholine is modulated by the provision of exogenous surfactant.

This part of the mechanistic sub-study will be conducted at Liverpool and Southampton trial sites only. Infants at these two centres will receive a single intravenous dose of D_9-choline (3.6 mg/kg body weight) prior sampling of BAL fluid and trial intervention at time = 0 hours, followed by further BAL fluid sampling at 12, 24, 36 and 48 hours if still ventilated. A single intravenous dose of deuterium-labelled choline administered prior to intervention allows assay of endogenous surfactant synthesis (31). A small blood sample (1.25ml (range 1.0 to 1.3 ml)) will also be taken at these times. D_9-choline contains the stable isotope deuterium, which is non-radioactive and non-toxic. Choline is an essential vitamin and the dose is within the daily-recommended allowance. Investigators at University of Southampton have extensive experience administering this metabolic probe in clinical studies of: preterm infants, infants with acute lung injury (ALI), and adults with acute respiratory distress syndrome patients with no adverse effects. ESI-MS analysis of unlabelled PC and PC labelled by incorporation of D_9-choline will provide a measure of surfactant phospholipid synthesis and turnover.

12.2.2 Recovery of surfactant proteins A & D (SP-A & SP-D) is modulated by exogenous surfactant.

Presence of SP-A and SP-D in the plasma has been proposed as a biomarker for acute lung injury (32). Among children mechanically ventilated for ALI including in bronchiolitis, we have previously shown reduced levels of SP-A and SP-D in the early stages of the disease, with recovery in SP-D levels in BAL fluid linked to progress to extubation, along with normalisation of DPPC and PG levels (33).

It is thought that surfactant deficiency contributes to acute lung injury by direct disruption of type II cell function and enhanced degradation of the surfactant system. SPA is secreted along with the phospholipid components of surfactant in lamellar bodies. Thus, metabolic studies demonstrating decreased PL secretion imply decreased SP-A secretion. However, this needs to be confirmed by direct measurement from secretions in the airways in a well-characterised population sampled under controlled conditions.

We will measure SP-A and SP-D by enzyme linked immunometric assay (ELISA) and Western blot at 0hr and 24hr sampling time points from subjects at all sites and in addition at 12hr, and 36hr from subjects at Liverpool and Southampton if still ventilated. We will compare levels over time relative to baseline, group-wise according to intervention.

No previous study has had the opportunity to relate the levels of SP-A & SP-D to surfactant metabolism during the natural history of bronchiolitis disease (placebo arm) or when modified by administration of exogenous surfactant. This proposal will evaluate not only the presence or absence of SPs but also provide evidence for their role as markers of lung recovery in the BAL.

For peer review only - http://bmjopen.bmj.com/site/about/guidelines.xhtml

12.2.3 Lower airway inflammation is modulated by exogenous surfactant[5]

Our previous study (3 arms, 27-plex, n=301) on local airway response to RSV bronchiolitis found high levels of IL1b, IL15, IL13, MIP1b, MCP1, IL-8 & PDGF and low levels of IFNg, IP10 & RANTES associated with life-threatening bronchiolitis (all p<0.025) (34). Subject to other funding, these 10 markers will be measured in BAL at t=0hr and 24hr in all subjects. Comparison will be made between baseline levels and second sample in infants treated with surfactant and placebo. Correlation will be sought between the inflammatory markers and levels of surfactant lipid and proteins.

12.2.4 Treatment efficacy or failure is associated with detection of specific pathogens.

We and others have demonstrated that certain pathogens and multiple infections are associated with poor outcome (35). Using accredited diagnostic polymerase chain reaction (PCR) multiplex assays we will assay BAL sampled daily for multiple pathogens and seek any relation with pathogens detected and outcome. While RSV infection accounts for about 75% of severe cases of bronchiolitis, dual viral infection or viral infection in combination with bacterial infections can account for severe disease. The aetiology of bronchiolitis in each subject must be identified to test for any pathogen specific effect.

12.2.5 Mechanistic Protocol

12.2.5.1 Surfactant phospholipid and protein concentration and composition (all sites)

Small volume lung lavage (0.9% NaCl, 1ml/kg body weight) will be performed in all participating sites at t=0, 24 just prior to trial intervention, and separated for protein and lipid analysis. For lipid analysis, butylated hydroxytoluene (10 of 20g/l in ethanol) will be added to all samples to prevent sample oxidation. Samples will be centrifuged at 400xgx10 min and the supernatant stored in aliquots for surfactant analysis at -80°C at each site. Other BAL fluid aliquots will be used for the inflammation and infection mechanistic subprojects. At the end of each winter season, all samples will be sent to Southampton for surfactant analysis.

Protein analysis will be conducted by standard techniques of ELISA and Western blotting. For PL analysis, samples will be lipid extracted in Southampton using organic solvents after addition of internal quantification standards and analysed by direct infusion ESI-MS. Analytes to be measured using diagnostic precursor scanning will include individual molecular species of PC, LysoPC, PG, PI and SM. Results will be expressed as % composition within an individual lipid class, as % composition of all measured lipids and as concentration (nmol/ml). Analysis of BAL fluid urea by colorimetric enzymatic assay will be used as a correction factor for BAL fluid dilution. While this in not ideal, a previous study indicates that it provides a reasonable correction for single recovery small volume lavage in ventilated infants (28).

Research Questions

Does administration of exogenous surfactant (poractant alfa) restore the dysfunctional composition of surfactant phospholipid characteristic of infants ventilated for bronchiolitis?

[5] Costs for this component of BESS are not being charged to NIHR EME.

For peer review only - http://bmjopen.bmj.com/site/about/guidelines.xhtml

A power calculation from the pilot data at t=48h indicates that it will require n=49/group to achieve significance at $p<0.05$ for %DPPC of 38.8% and 43.5% respectively for infants in the placebo and treated groups. This is a realistic goal as this represents obtaining appropriate samples from 37% of prospective infants in the study

Do PC and PG concentrations in BAL fluid increase with duration of ventilation in the placebo group of infants? This analysis will also address whether the correlation between initial rate of incorporation and time to recruitment is related to secretion of labelled PC into a larger surfactant pool or decreased surfactant secretion with duration of ventilation.

12.2.5.2 Kinetics of surfactant synthesis

The mechanistic study of phosphatidylcholine surfactant synthesis will take place only at Liverpool and Southampton, which both have the appropriate infrastructure (staff and facility) to collect and process BAL more frequently. It will incorporate the phospholipid concentration and composition protocol outlined above, combined with a stable isotope labelling protocol. With specific consent recruited infants at both sites, in both placebo and treated groups, will be intravenously infused between t=0 and 1h with non-radioactive, stable isotope-labelled *methyl*D_9-choline chloride at 3.6 mg/kg. Small volume lung lavages will be collected as above, with additional time points at t=12h and 36h. EDTA blood samples (1.25ml (range 1.0 to 1.3 ml)) will be taken at the same time points and centrifuged at 400xgx10min to isolate plasma, which will be stored at -80°C. Samples will be couriered from Liverpool to Southampton at the end of the winter season and sample extraction and processing will follow exactly the same protocol as above. Lipid extracts from both BAL fluid and plasma will be subjected to the identical ESI-MS analytical protocol described above, with the addition of a diagnostic scan for *methyl*-D_9-labelled PC. Enrichment of methylD9-choline in plasma choline will be analysed by LC-MS/MS using a MRM protocol to provide an indication of substrate choline turnover. Urea will be analysed by colorimetric enzymatic assay in plasma as well as in BAL fluid, to enable calculation of analyte concentrations per ml of epithelial lining fluid (ELF). Results will be calculated, in addition to those described above, as enrichment (Σ abundance of labelled PC species/Σ abundance of labelled + unlabelled PC species), fractional synthetic rates and concentration of labelled species (nmol/mL ELF).

12.2.5.3 Outcomes

The major discriminators between treatment and placebo groups will be the concentration of newly synthesised and secreted *methyl*-D_9 molecular species calculated as nmoles/ml of ELF after correction for urea dilution between BAL fluid and plasma and the FSR. Power: based on our pilot data on fractional synthetic rate of surfactant in two disease states (0.039±0.005 vs 0.030±0.011) 2 tail, a=0.05 requires total n=25/group for 1-b >0.95.

Enrichment of label in molecular species of plasma PC and in plasma choline will provide comparator data to distinguish responses that are lung specific from those determined by variability in substrate availability and metabolism.

Administration of a second dose of exogenous surfactant at t=12 hours in the treated infants will permit calculation of endogenous and exogenous surfactant pool sizes by labelled surfactant dilution.

For peer review only - http://bmjopen.bmj.com/site/about/guidelines.xhtml

13 REGULATORY AND ETHICAL APPROVALS

13.1 Statement of Compliance

Statement of compliance: The study will be carried out in accordance with:

- The World Medical Association Declaration of Helsinki ,
- CTRC Standard Operating Procedures
- Principles of Good Clinical Practice
- The template content is structured consistent with the SPIRIT (Standard Protocol Item: Recommendations for Interventional Trials 2013)
- UK Policy Framework for Health and Social Care Research

SI /EU Regulation	Title	Main impact/scope
2001/20/EC	The EU Clinical Trials Directive	National Competent Authority<br>Ethics Framework<br>GCP legal requirement<br>Good Manufacturing Practice Protocol/Amendments/safety<br>Protection of Vulnerable Groups<br>Consent / Data protection
2004/1031	Medicines for Human use Clinical Trials Regulation	Transposed EU CT Directive in UK
2005/28/EC	EU Good Clinical Practice (GCP) Directive	Investigator brochure<br>Archiving<br>Mandatory training for trial teams
2006/1928	Amends 2004/1031	Investigator brochure /essential documents<br>Serious Breach<br>Declaration of Helsinki 1996 version for CTIMP
2006/2984	Amends 2004/1031	Consent for incapacitated adult by legal representative or emergency deferred consent
2008/941	Amends 2004/1031	Blood safety and quality<br>Emergency Deferred consent for children
2009/1164	Miscellaneous Amendment	Urgent Safety measures
2009/3063	Amends 2004/1031	Nurse and pharmacists to prescribe unlicensed medicines

For peer review only - http://bmjopen.bmj.com/site/about/guidelines.xhtml

13.2 Regulatory Approval

This trial falls within the remit of the EU Directive 2001/20/EC, transposed into UK law as the UK Statutory Instrument 2004 No 1031: Medicines for Human Use (Clinical Trials) Regulations 2004 as amended.

BESS will be registered with the MHRA and a Clinical Trials Authorisation (CTA) will be obtained prior to the trial commencing.

13.3 Ethical Considerations

The study will abide by the principles of the World Medical Association Declaration of Helsinki.

In compliance with GCP, parents or those with parental responsibility will be fairly informed of the trial, risks, benefits, and alternatives, asked to ensure they understand how it relates to their infant, and make a voluntary decision about entry. Autonomy, privacy and welfare will be paramount, overriding the research. Parents will be kept informed of findings that might cause them to withdraw, without detriment to on-going care.

Consent in emergency setting poses particular challenges for parents. We have allowed a window of 48 hours from intubation to intervention during which to seek consent and randomisation. Dr Kerry Woolfall will lead the sub study to explore staff and parents' acceptability of our approach to consent in this challenging setting. Her work includes CONNECT study guidance, adopted by NIHR Clinical Trials Toolkit (http://www.ct-toolkit.ac.uk/routemap/informed-consent), which helps to ensure recruitment and consent to critical care trials is ethically appropriate and addresses the needs critically ill children and their families (49, 50). The trial team are experienced at conducting studies and trials in emergency settings.

The acceptability of medicines derived from pigs to families of the Jewish and Islamic faiths has been addressed with Rabbi Adler, Director of the Kashrus & Medicines Information Service; the Ashkenazi Beth Din of the United Synagogue & the Union of Orthodox Hebrew Congregations and with Dr Shafi, Chairman of the Research & Documentation Committee of the Muslim Council of Great Britain.

These religious authorities recognise that some scholars and families will have their own interpretations of their faith, as do other faiths with regards to this topic, which further complicates the issue. BESS will liaise with these authorities during the development of parent information and consent materials.

13.4 HRA Approval

BESS will come under the Health Research Authority (HRA) Approvals process as part of the initial REC submission and will follow the HRA processes for submission of amendments to REC and the HRA.

13.5 Ethical and Local Governance Approval

Prior to the trial being initiated at CTRC, a favourable ethical opinion will be obtained from a REC and global governance approval from the HRA. Prior to opening a centre to recruitment, CTRC will ensure that local governance approval has been obtained: for sites in England and Wales, this will be

For peer review only - http://bmjopen.bmj.com/site/about/guidelines.xhtml

"Capacity & Capability" Confirmation; for sites in Scotland and Northern Ireland, this will be R&D Approval.

13.6 Protocol Deviation and Serious Breaches

Incidence of protocol non-compliance, whether reported prospectively (e.g. where a treatment cannot be administered at a scheduled time) or retrospectively noted (e.g. as a result of central monitoring) are recorded as protocol deviations, the incidence of which are monitored and reported to trial oversight committees.

A breach of the protocol or GCP is 'serious' if it meets the regulatory definition of being "likely to affect to a significant degree the safety or physical or mental integrity of the trial participants, or the scientific value of the trial". All confirmed serious breaches of GCP or protocol will be reported to the MHRA and REC in an expedited manner by the CTRC on behalf of the sponsor.

If any persons involved in the conduct of the trial become aware of a potential serious breach, they must immediately report this to the CTRC who will in turn notify the Sponsor. The Sponsor will assess the breach and determine if it meets the criteria of a 'serious' breach of GCP or protocol and therefore requires expedited reporting to the MHRA and REC.

In determining whether or not the breach is likely to affect to a significant degree the safety, physical or mental integrity of participants, the Sponsor may seek advice from medical expert members of the Trial Management Group (TMG) and/or of the independent oversight committees (IDSMC and TSC). In determining whether or not the breach is likely to significantly affect the scientific value of the trial, the Sponsor may seek advice from the Trial Statistician. However, the Sponsor retains responsibility for the assessment of whether or not a breach meets the definition of 'serious' and is subject to expedited reporting to MHRA and REC.

Breaches confirmed as 'serious' will be reported to the MHRA and REC within 7 days by the CTRC and notified to the TMG, IDSMC and TSC at their next meetings.

Any requests for additional information from the Sponsor, TMG, TSC, IDSMC, REC or MHRA will be promptly actioned by the relevant member(s) of the research team and open communication will be maintained to ensure appropriate corrective actions are taken and documented.

Other incidences of protocol non-compliance will be recorded as protocol deviations, the frequency of which will be monitored and reported to the trial oversight committees.

13.7 Study Discontinuation

In the event that the BESS trial is discontinued, any participants currently participating will not be given their next dose of poractant alfa or placebo. Decisions on weaning and extubation will revert to standard care.

Reporting of adverse events will continue as per trial protocol.

For peer review only - http://bmjopen.bmj.com/site/about/guidelines.xhtml

14 DATA MANAGEMENT AND TRIAL MONITORING

Details of the monitoring to be carried out for the BESS trial are included in the BESS Trial Monitoring Plan, maintained separately to this protocol.

Trial Oversight Committees related to the monitoring of the trial are detailed in section 15.4.

14.1 Source Documents

In order to resolve possible discrepancies between information appearing in the case report form (CRF) and any other participant related documents, it is important to know what constitutes the source document and therefore the source data for all information in the CRF.

Source data is defined as: “All information in original records and certified copies of original records of clinical findings, observations, or other activities in a clinical trial necessary for the reconstruction and evaluation of the trial. Source data are contained in source documents (original records or certified copies).” (ICH E6, 1.51).

Source documents are defined as: “Original documents, data, paper and electronic records (e.g. hospital records, clinical and office charts, laboratory notes, memoranda, participant diaries or evaluation checklists, pharmacy dispensing records, recorded data from automated instruments, copies or transcriptions certified after verification as being accurate copies, microfiches, photographic negatives, microfilm or magnetic media, x-rays, participant files, and records kept at the pharmacy, at the laboratories and at medico-technical departments involved in the clinical trial).” (ICH E6, 1.52).

The CRF will be considered the source document for data where no prior record exists, and which is recorded directly in the CRF. A BESS source document list will be produced for each site.

Date(s) of conducting the informed consent process including date of provision of patient information, confirmation of full eligibility, randomisation number and the fact that the patient is participating in a clinical trial (including possible treatment arms) should be added to the patient’s medical record chronologically.

14.2 Data Capture Methods

The study case report form (CRF) is the primary data collection instrument for the study. All data requested on the CRF must be recorded. All missing data must be explained.

Please ensure documents are sent by sites separate to the consent form as this has patient identifiable data on it.

The LRSQ will be completed by participants’ parents at 6m and 12m after randomisation. These are completed online but paper copies will be available on request for parents without internet access. If paper questionnaires are used **sites should photocopy them** in order to retain a copy at site before mailing originals to CTRC.

BESS will also be collecting data from the Paediatric Intensive Care Audit Network (PICANet). PICANet is a secure confidential high-quality clinical database, recording details of the treatment of all critically ill children in PICUs. Collection of personally identifiable data has been approved by the

For peer review only - http://bmjopen.bmj.com/site/about/guidelines.xhtml

Patient Information Advisory Group (now the NHS Health Research Authority Confidentiality Advisory Group) and ethical approval granted by the Trent Medical Research Ethics Committee.

PICANet will provide a bespoke download page for the trial and sites will regularly download their own data for consented BESS patients only and transfer this to the CTRC.

14.3 Monitoring

14.3.1 Risk Assessment

This trial will undergo a risk assessment, completed in partnership between:

- Representative/s of the Trial Sponsor
- Chief Investigator
- CTRC Trial Coordinator and Supervising Trial Manager
- CTRC Trial Statistician and Supervising Statistician
- CTRC Information Systems and Data Management teams
- CTRC Director

In conducting the risk assessment, the contributors will consider potential participant, organisational and study hazards, the likelihood of their occurrence and resulting impact should they occur.

Monitoring for BESS will be informed by the trial specific risk assessment and will be conducted as per a detailed Trial Monitoring Plan (TMP), maintained separately to this protocol and stored in the TMF. The TMP will describe who will conduct the monitoring, at what frequency monitoring will be done and what level of detail monitoring will be conducted.

14.3.2 Central Monitoring

Data received at CTRC will be checked for missing or unusual values (range checks) and checked for consistency over time. Any suspect data will be returned to the site in the form of data queries. Data query forms will be produced at the CTRC from the trial database and sent either electronically or through the post to a named individual (as listed on the site delegation log). Sites will respond to the queries providing an explanation/resolution to the discrepancies and return the data query forms to CTRC. The forms will then be filed along with the appropriate CRFs and the appropriate corrections made on the database. There are a number of monitoring features in place at the CTRC to ensure reliability and validity of the trial data, to be detailed in the TMP maintained separately from this protocol.

Central checks of consent will be completed for each participant to ensure the completeness of consent and that the timing of consent is in line with the protocol.

Central monitoring will be performed in a proportionate manner as informed by the Trial Risk Assessment and Monitoring Plans.

14.3.3 Clinical Site Monitoring

In order to perform their role effectively, the Trial Coordinator, Data Manager and persons involved in Quality Assurance and Inspection may need direct access to primary data during monitoring

For peer review only - http://bmjopen.bmj.com/site/about/guidelines.xhtml

visits, e.g. participant medical records, laboratory reports, appointment books, etc. Since this affects the participant's confidentiality, this fact is included on the PISC.

The purposes of monitoring visits, if triggered as per CTRC SOPs, include but are not limited to:

- assessing compliance with the study protocol
- discussing any emerging problems that may have been identified prior to the visit
- checking CRF and query completion practices

14.4 Confidentiality

Individual participant medical information obtained as a result of this study is considered confidential and disclosure to third parties is prohibited with the exceptions noted below.

CRFs will be pseudonymised – they will not contain details of participant names and will be labelled with the unique trial randomisation and/or screening number. Medical information may be given to the participant's medical team and all appropriate medical personnel responsible for the participant's welfare.

Verification that appropriate informed consent is obtained will be enabled by the provision of copies of participant's signed informed consent forms being supplied to the CTRC by recruiting centres, which requires that name data will be transferred to the CTRC.

The CTRC will be undertaking activities requiring the transfer of identifiable personal data. The transfer of this data is disclosed in the PISC. The CTRC will be responsible for sending links to the LRSQ to trial participants at 6m and 12m post randomisation, and therefore will be required to receive contact details including name and email address. Should the parent require a paper copy this will be sent by the recruiting site as the CTRC will not collect participant addresses.

All documentation received at CTRC which includes direct identifiers such as names and email addresses (e.g. consent forms) will be stored securely and separately to all other pseudonymised participant data (e.g. medical CRFs).

The CTRC will preserve the confidentiality of participants taking part in the study and The University of Liverpool is registered as a Data Controller with the Information Commissioners Office.

14.5 Quality Assurance (QA) and Quality Control (QC)

Quality Assurance (QA) includes all the planned and systematic actions established to ensure the trial is performed and data generated, documented, recorded and reported in compliance with applicable regulatory, ethical and governance requirements. Quality Control (QC) includes the operational techniques and activities performed within the QA system to verify that the requirements for quality of the trial-related activities are fulfilled: e.g. state what clinical site monitoring (and audit) is planned, if any. In accordance with the monitoring plan site visits will or will not be conducted and source verification performed if central monitoring processes indicate these are required. Monitoring activities could include, but are not limited to the following:

- The Trial Coordinator at the CTRC will verify appropriate approvals are in place prior to initiation of a site and the relevant personnel have attended trial specific training. A greenlight

For peer review only - http://bmjopen.bmj.com/site/about/guidelines.xhtml

checklist will verify all approvals are in place prior to trial initiation at CTRC and the individual site.

- The TMG will determine the minimum key staff required to be recorded on the delegation log in order for the site to be eligible to be initiated.
- Data will be evaluated for compliance with protocol and accuracy in relation to source documents.
- The study will be conducted in accordance with procedures identified in the protocol.
- Independent oversight of the trial will be provided by the Data and Safety Monitoring Committee and independent members of the Trial Steering Committee.
- The types of materials to be reviewed, who is responsible, and the schedule for reviews may be specified or referenced in other documents.
- Types and mechanisms of training of staff for the study should be specified.
- The PI and other key staff from each centre will attend site initiation training, coordinated by the CTRC, which will incorporate elements of trial-specific training necessary to fulfil the requirements of the protocol.
- The Trial Management Group will monitor screening, randomisation and consent rates between centres.
- The process for consent, recruitment and randomisation will be evaluated for compliance with the protocol.
- Data quality checks and monitoring procedures will be undertaken in line with the trial Data Management Plan.
-

14.6 Records Retention

All trial documents will be retained for a maximum period of 25 years from the End of Trial.

The site PI is responsible for archiving of all relevant source documents so that the trial data can be compared against source data after completion of the trial (e.g. in case of inspection from authorities). The PI at each trial site must make arrangements to store the essential trial documents, (as defined in Essential Documents for the Conduct of a Clinical Trial (ICH E6, Guideline for GCP)) including the Investigator Site File and Pharmacy Site File, until the CTRC informs him/her that the documents are no longer to be retained. The PI is required to ensure the continued storage of the documents, even if s/he, for example, leaves the clinic/practice or retires before the end of required storage period. Delegation must be documented in writing.

All other persons and organisations involved in the trial will be responsible for storing and archiving the parts of the TMF relevant to their delegated duties (e.g. laboratories, IMP manufacturers and distributors, third-party vendors providing randomisation and IMP allocation systems, etc.).

The CTRC undertakes to store originally completed CRFs for the same period, except for source documents pertaining to the individual investigational site, which are kept by the site PI only. The CTRC will archive the documents in compliance with the principles of GCP . All electronic CRFs and trial data will be archived onto an appropriate media for long term accessible storage. Hard copies of data will be boxed and transferred to secure premises where unique reference numbers are applied to enable confidentiality, tracking and retrieval.

For peer review only - http://bmjopen.bmj.com/site/about/guidelines.xhtml

15 INDEMNITY

BESS is sponsored by The University of Liverpool and co-ordinated by the CTRC in the University of Liverpool. The Sponsor does not hold insurance against claims for compensation for injury caused by participation in a clinical trial and they cannot offer any indemnity. As this is an investigator-initiated study, The Association of the British Pharmaceutical Industry (ABPI) guidelines for patient compensation by the pharmaceutical industry do not apply. However, in terms of liability, NHS Trust and Non-Trust Hospitals have a duty of care to patients treated, whether or not the patient is taking part in a clinical trial, and they are legally liable for the negligent acts and omission of their employees. Compensation is therefore available in the event of clinical negligence being proven.

The Sponsor does not accept liability for any breach in any other hospital's duty of care, or any negligence on the part of employees of hospitals. This applies whether the hospital is an NHS Trust or not.

The Sponsor has vicarious liability for the actions of its staff, when through the course of their employment they are involved in the design and initiation of a clinical trial, including but not limited to the authorship of the Clinical Trial Protocol. The University of Liverpool has appropriate insurance in place to cover this liability.

All participants will be recruited at NHS sites and therefore the NHS indemnity scheme/NHS professional indemnity will apply with respect to claims arising from harm to participants at site management organisations.

Clinical negligence is defined as:

"A breach of duty of care by members of the health care professions employed by NHS bodies or by others consequent on decisions or judgments made by members of those professions acting in their professional capacity in the course of their employment, and which are admitted as negligent by the employer or are determined as such through the legal process".

For peer review only

For peer review only - http://bmjopen.bmj.com/site/about/guidelines.xhtml

16 ROLES AND RESPONSIBILITIES

16.1 Role of Study Sponsor and Study Funder

The Sponsor of this trial is the University of Liverpool. The Sponsor will ensure that clear agreements are reached, documented and carried out, respecting the dignity, rights, safety and wellbeing of participants and the relationship with healthcare professionals. This will provide for proper design, management, initiation, conduct, monitoring, data collection, data analysis, data protection, financing and reporting of this trial meeting appropriate scientific, legal and regulatory standards. The responsibility for design, conduct, management, data analysis, data interpretation, manuscript writing, and dissemination of results is delegated to the Trial Management Group.

The funders of this study are the National Institute for Health Research and Medical Research Council who are providing financial funding through their Efficacy and Mechanism Evaluation Programme. The funders will ensure there is proper use of the funds they have allocated to provide value for money. The funders assure the quality of the study, taking the lead in establishing that the research proposal is worthwhile, of high scientific quality, has an appropriate research infrastructure with expert clinical trial management, has the capacity to comply with the principles of GCP and represents good value for money.

Chiesi Farmaceutici S.p.A (Chiesi) are supplying the IMP endotracheal poractant alfa (Curosurf®) to be administered as trial IMP and will be securing use of a randomisation and re-supply system for use in the trial through an Investigator Initiated Clinical Trial Research Agreement between Chiesi and the Sponsor. Chiesi are not providing any financial funding in connection with this Agreement.

16.2 Funding and Support in Kind

Funder(s)	Financial and Non-financial Support Given
National Institute of Health Research	Financial Funding (£1,655,615.46)
Chiesi Farmaceutici S.p.A (UK address: 333 Styal Road Manchester M22 5LG)	Supply of endotracheal poractant alfa (Curosurf®), and contracting of a third party distributor who will also provide a randomisation and drug resupply system

For peer review only - http://bmjopen.bmj.com/site/about/guidelines.xhtml

16.3 Protocol Contributors

Name	Affiliations	Contribution to protocol
Calum Semple	Institute of Translational Medicine, University of Liverpool and<br>Alder Hey Children's Hospital NHS Foundation Trust	Inception of study, led the writing of this protocol, clinical and scientific arrangements, trial design and conduct
Tracy Moitt	CTRC, University of Liverpool	Protocol development, governance arrangements and trial conduct
Chloe Donohue	CTRC, University of Liverpool	Protocol development, governance arrangements and trial conduct
Ashley Jones	CTRC, University of Liverpool	Led statistical arrangements, trial design and conduct
Laura Sutton	CTRC, University of Liverpool	Statistical arrangements, trial design and conduct
Kerry Woolfall	Institute of Psychology, Health & Society, University of Liverpool	Led design of parent/staff experience and consent study (WP-B)
Tony Postle	Faculty of Medicine, University of Southampton	Collaborator from inception, design and development of mechanistic study (WP-C)
Howard Clark	Faculty of Medicine, University of Southampton	Collaborator from inception, design and development of mechanistic study (WP-C)
Jens Madsen	University Hospital Southampton NHS Foundation Trust	Design and development of mechanistic study (WP-C)
John Pappachan	University Hospital Southampton NHS Foundation Trust	Design and development of mechanistic study (WP-C)
Paul Ritson	Alder Hey Children's Hospital NHS Foundation Trust	Development of BAL procedures, intervention administration/ handling, and PICU processes
Mark Turner	Institute of Translational Medicine, University of Liverpool and<br>Liverpool Women's Hospital NHS Foundation Trust	Inception of study, clinical and scientific arrangements, trial design and liaison with pharmaceutical company
Katie Neville	CTRC, University of Liverpool	Quality Assurance review
Guido Varoli	Chiesi Farmaceutici S.p.A.	Limited to provision of information relating to IMP specifics (formulation, packaging, administration, distribution etc.)

Advice was received from all Co-Applicants to the NIHR EME funding applications and protocol submissions.

For peer review only - http://bmjopen.bmj.com/site/about/guidelines.xhtml

The design, conduct, data analysis, data interpretation, manuscript writing and dissemination of results are the responsibility of the TMG, subject to the approval of the University of Liverpool as Sponsor, the MHRA and National Institute for Health as Funders.

Chiesi Farmaceutici S.p.A. are responsible for supplying the IMP and subcontracting an independent third party to undertake distribution of the IMP and development, management and maintenance of a randomisation and drug resupply system.

16.4 Trial Committees

16.4.1 Trial Management Group (TMG)

A Trial Management Group (TMG) will be formed comprising the Chief Investigator, other lead investigators (clinical and non-clinical), members of the CTRC and a lay member. The TMG will be responsible for the day-to-day running and management of the trial. Refer to the TMG terms of reference and trial oversight committee membership document for further details. Meetings will be in Liverpool with teleconferencing. The TMG will report to the TSC.

16.4.2 Trial Steering Committee (TSC)

The TSC will consist of an independent medical expert chairperson (neonatology), two independent medical experts (paediatric intensive care), an independent biostatistician and two lay members (one independent, one non-independent). TMG representatives will be given the opportunity to contribute as non-independent and non-voting TSC members. The role of the TSC is to provide overall supervision for the trial and provide advice through its independent Chairman. The ultimate decision for the continuation of the trial lies with the TSC.

The TSC will first convene prior to the start of recruitment and will then define frequency of subsequent meetings (at least annually). Refer to the TSC terms of reference and trial oversight committee membership document for further details.

16.4.3 Independent Data and Safety Monitoring Committee (IDSMC)

The IDSMC consists of an independent medical expert chairperson (neonatology), an independent expert in Paediatric Intensive Care and an independent expert in medical statistics.

The IDSMC will be responsible for reviewing and assessing recruitment, interim monitoring of safety and effectiveness, trial conduct and external data. The IDSMC will first convene prior to the start of recruitment and will then define frequency of subsequent meetings (at least annually). Details of the interim analyses and monitoring are provided in section 11.4 .

The IDSMC will provide a recommendation to the TSC concerning the continuation of the study. Refer to the IDSMC charter and trial oversight committee membership document for further details.

For peer review only - http://bmjopen.bmj.com/site/about/guidelines.xhtml

17 PUBLICATION AND DISSEMINATION

17.1 Publication Policy

The results from different centres will be analysed together and published as soon as possible. The design, conduct, data analysis, data interpretation, manuscript writing, and dissemination of results are the responsibility of the TMG on behalf of the University of Liverpool as Sponsor and the National Institute for Health as Funders.

The TMG will form the basis of the Writing Committee and advise on the nature of publications. The Uniform Requirements for Manuscripts Submitted to Biomedical Journals (http://www.icmje.org/) will be respected. The National Clinical Trial (NCT) identifier allocated to this trial will be attached to any publications resulting from this trial.

The members of the TSC and IDSMC will be listed with their affiliations in the Acknowledgements / Appendix of the main publication.

17.1.1 Authorship

Contributors to all four of (i) the design, conduct, data analysis and interpretation, (ii) writing, (iii) manuscript approval and (iv) accountability for the integrity of the work will, depending on their contribution and journal requirements, be included by name at the manuscript head or listed at the end in a by-line as members of the BESS Consortium which will also be named at the manuscript head.

17.2 Dissemination to Key Stakeholders

BESS intends to publish open access results in high impact journals (e.g. British Medical Journal, New England Journal of Medicine, Lancet, American Journal of Respiratory and Critical Care Medicine). The full trial report will also be published in the NIHR EME Journal.
BESS will also present at international meeting of the major Paediatric Intensive Care Societies, American Thoracic Society, European Respiratory Society.

The involvement of representatives of the UK Paediatric Intensive Care Society Study Group (PICSSG) as co-applicants will be key to ensuring dissemination of result to the Paediatric Intensive Care community.

At end of study, we will organise a symposium in partnership with the UK Paediatric Intensive Care Society where the results of the study will be presented alongside reports of family experiences as reported directly by parents. The symposium will be open to intensivists, respiratory physicians, study staff and any interest participating families.
We also intend to disseminate via a PPI event to be hosted in Liverpool at end of study with targeted open invitation to all families of participants.

We anticipate that because of the nature of the study population and the disease that our findings will be of interest to the general public. For this reason we will be proactive in targeting the broadcast media and press through our professional external media consultants, with due regard to the usual NIHR publicity and publication conditions.

For peer review only - http://bmjopen.bmj.com/site/about/guidelines.xhtml

Public dissemination will include contemporary social media including a study website and Twitter account.

17.1 Data Sharing

Individual Clinicians must undertake not to submit any part of their individual data for publication without the prior consent of the TMG. All publications shall include a list of participating PIs and collaborators.

For peer review only

For peer review only - http://bmjopen.bmj.com/site/about/guidelines.xhtml

18 CHRONOLOGY OF PROTOCOL AMENDMENTS

18.1 Version 1.0 (dd/mon/yyyy)

Original Approved version.

For peer review only

For peer review only - http://bmjopen.bmj.com/site/about/guidelines.xhtml

19 REFERENCES

1. Nair H, Nokes DJ, Gessner BD, Dherani M, Madhi SA, Singleton RJ, et al. Global burden of acute lower respiratory infections due to respiratory syncytial virus in young children: a systematic review and meta-analysis. Lancet. 2010;375(9725):1545-55.

2. Green CA, Yeates D, Goldacre A, Sande C, Parslow RC, McShane P, et al. Admission to hospital for bronchiolitis in England: trends over five decades, geographical variation and association with perinatal characteristics and subsequent asthma. Arch Dis Child. 2016;101(2):140-6.

3. Jat KR, Chawla D. Surfactant therapy for bronchiolitis in critically ill infants. Cochrane Database Syst Rev. 2015(8):CD009194.

4. Luchetti M, Casiraghi G, Valsecchi R, Galassini E, Marraro G. Porcine-derived surfactant treatment of severe bronchiolitis. Acta Anaesthesiol Scand. 1998;42(7):805-10.

5. Luchetti M, Ferrero F, Gallini C, Natale A, Pigna A, Tortorolo L, et al. Multicenter, randomized, controlled study of porcine surfactant in severe respiratory syncytial virus-induced respiratory failure. Pediatr Crit Care Med. 2002;3(3):261-8.

6. Tibby SM, Hatherill M, Wright SM, Wilson P, Postle AD, Murdoch IA. Exogenous surfactant supplementation in infants with respiratory syncytial virus bronchiolitis. Am J Respir Crit Care Med. 2000;162(4 Pt 1):1251-6.

7. McNamara PS, Ritson P, Selby A*, et al* Bronchoalveolar lavage cellularity in infants with severe respiratory syncytial virus bronchiolitis. Archives of Disease in Childhood. 2003;88:922-926.

8. von Neergaard K. Neue Auffassungen übereinen Grundbegriff der Atemmechanik. Die Retraktionskraft der Lunge, abhängig von der Oberfl ächenspannung in den Alveolen. (New views on a fundamental concept of respiratory mechanics. The retraction force of the lung is dependent on the surface tension within the alveoli.). Z Gesamt Exp Med 1929;66:373-94.

9. Goh BC, Rynkiewicz MJ, Cafarella TR, White MR, Hartshorn KL, Allen K, et al. Molecular mechanisms of inhibition of influenza by surfactant protein D revealed by large-scale molecular dynamics simulation. Biochemistry. 2013;52(47):8527-38.

10. LeVine AM, Lotze A, Stanley S, Stroud C, O'Donnell R, Whitsett J, et al. Surfactant content in children with inflammatory lung disease. Crit Care Med. 1996;24(6):1062-7.

11. Dargaville PA, South M, McDougall PN. Surfactant abnormalities in infants with severe viral bronchiolitis. Arch Dis Child. 1996;75(2):133-6.

12. Skelton R, Holland P, Darowski M, Chetcuti PA, Morgan LW, Harwood JL. Abnormal surfactant composition and activity in severe bronchiolitis. Acta Paediatr. 1999;88(9):942-6.

13. Kerr MH, Paton JY. Surfactant protein levels in severe respiratory syncytial virus infection. Am J Respir Crit Care Med. 1999;159(4 Pt 1):1115-8.

14. Hartmann F, Fiori HH, Ramos Garcia PC, Piva J, Fiori RM. Surfactant deficiency in infants with severe acute viral bronchiolitis. J Pediatr. 2014;164(6):1432-5.

15. Kelly E, Bryan H, Possmayer F, Frndova H, Bryan C. Compliance of the respiratory system in newborn infants pre- and postsurfactant replacement therapy. Pediatr Pulmonol. 1993;15(4):225-30.

For peer review only - http://bmjopen.bmj.com/site/about/guidelines.xhtml

16. Jat KR, Chawla D. Surfactant therapy for bronchiolitis in critically ill infants. Cochrane Database Syst Rev. 2012(9):CD009194.

17. NICE. Bronchiolitis in children: diagnosis and management (NG9). 2015.

18. Siegel LS. Correction for prematurity and its consequences for the assessment of the very low birth weight infant. Child Dev. 1983;54(5):1176-88.

19. Soll RF. Surfactant treatment of the very preterm infant. Biol Neonate. 1998;74 Suppl 1:35-42.

20. Powell CV, McNamara P, Solis A, Shaw NJ. A parent completed questionnaire to describe the patterns of wheezing and other respiratory symptoms in infants and preschool children. Arch Dis Child. 2002;87(5):376-9.

21. Paret G, Ziv T, Barzilai A, Ben-Abraham R, Vardi A, Manisterski Y, et al. Ventilation index and outcome in children with acute respiratory distress syndrome. Pediatr Pulmonol. 1998;26(2):125-8.

22. Bilan N, Dastranji A, Ghalehgolab Behbahani A. Comparison of the spo2/fio2 ratio and the pao2/fio2 ratio in patients with acute lung injury or acute respiratory distress syndrome. J Cardiovasc Thorac Res. 2015;7(1):28-31.

23. Wolfe R, Carlin JB. Sample-Size Calculation for a Log-Transformed Outcome Measure. Controlled Clinical Trials. 1999;20(6):547-54.

24. Menon K, Ward RE, Gaboury I, Thomas M, Joffe A, Burns K, et al. Factors affecting consent in pediatric critical care research. Intensive Care Med. 2012;38(1):153-9.

25. Kerrin A, Fitch P, Errington C, Kerr D, Waxman L, Riding K, et al. Differential lower airway dendritic cell patterns may reveal distinct endotypes of RSV bronchiolitis. Thorax. 2016.

26. McNamara PS, Fonceca AM, Howarth D, Correia JB, Slupsky JR, Trinick RE, et al. Respiratory syncytial virus infection of airway epithelial cells, in vivo and in vitro, supports pulmonary antibody responses by inducing expression of the B cell differentiation factor BAFF. Thorax. 2013;68(1):76-81.

27. McNamara PS, Ritson P, Selby A, Hart CA, Smyth RL. Bronchoalveolar lavage cellularity in infants with severe respiratory syncytial virus bronchiolitis. Arch Dis Child. 2003;88(10):922-6.

28. Semple MG, Dankert HM, Ebrahimi B, Correia JB, Booth JA, Stewart JP, et al. Severe respiratory syncytial virus bronchiolitis in infants is associated with reduced airway interferon gamma and substance P. PLoS One. 2007;2(10):e1038.

29. Bernhard W, Pynn CJ, Jaworski A, Rau GA, Hohlfeld JM, Freihorst J, et al. Mass spectrometric analysis of surfactant metabolism in human volunteers using deuteriated choline. Am J Respir Crit Care Med. 2004;170(1):54-8.

30. Dushianthan A, Goss V, Cusack R, Grocott MP, Postle AD. Altered molecular specificity of surfactant phosphatidycholine synthesis in patients with acute respiratory distress syndrome. Respir Res. 2014;15:128.

31. Bunt JE, Zimmermann LJ, Wattimena JL, van Beek RH, Sauer PJ, Carnielli VP. Endogenous surfactant turnover in preterm infants measured with stable isotopes. Am J Respir Crit Care Med. 1998;157(3 Pt 1):810-4.

For peer review only - http://bmjopen.bmj.com/site/about/guidelines.xhtml

32. Greene KE, Wright JR, Steinberg KP, Ruzinski JT, Caldwell E, Wong WB, et al. Serial changes in surfactant-associated proteins in lung and serum before and after onset of ARDS. Am J Respir Crit Care Med. 1999;160(6):1843-50.

33. Todd DA, Marsh MJ, George A, Henderson NG, Barr H, Sebastian S, et al. Surfactant phospholipids, surfactant proteins, and inflammatory markers during acute lung injury in children. Pediatr Crit Care Med. 2010;11(1):82-91.

34. Semple MG, Flanagan B, Smyth RL. Nasal Cytokines Associated With Householder Tobacco Smoking And HRSV Bronchiolitis. American Journal of Respiratory and Critical Care Medicine; December 2012: American Thoracic Society; 2012. p. A1026-A.

35. Semple MG, Cowell A, Dove W, Greensill J, McNamara PS, Halfhide C, et al. Dual infection of infants by human metapneumovirus and human respiratory syncytial virus is strongly associated with severe bronchiolitis. J Infect Dis. 2005;191(3):382-6.

36. Wright SM, Hockey PM, Enhorning G, Strong P, Reid KB, Holgate ST, et al. Altered airway surfactant phospholipid composition and reduced lung function in asthma. J Appl Physiol (1985). 2000;89(4):1283-92.

37. Postle AD, Heeley EL, Wilton DC. A comparison of the molecular species compositions of mammalian lung surfactant phospholipids. Comp Biochem Physiol A Mol Integr Physiol. 2001;129(1):65-73.

38. Mander A, Langton-Hewer S, Bernhard W, Warner JO, Postle AD. Altered phospholipid composition and aggregate structure of lung surfactant is associated with impaired lung function in young children with respiratory infections. Am J Respir Cell Mol Biol. 2002;27(6):714-21.

39. Wright JR. Immunoregulatory functions of surfactant proteins. Nat Rev Immunol. 2005;5(1):58-68.

40. LeVine AM, Gwozdz J, Stark J, Bruno M, Whitsett J, Korfhagen T. Surfactant protein-A enhances respiratory syncytial virus clearance in vivo. J Clin Invest. 1999;103(7):1015-21.

41. Rawat M, Chadrasekharan PK, Wiliams A, Gugino S, Koenigsknecht C, Swartz D, Ma CX, Mathew B, Nair J, Lakshminrusimha S. Oxygen Saturation Index and Severity of Hypoxic Respiratory Failure. Neonatology. 2015; 107: 161-166

For peer review only

For peer review only - http://bmjopen.bmj.com/site/about/guidelines.xhtml

20 DOCUMENTS SUPPLEMENTARY TO THE PROTOCOL

Documents referenced herein as accompanying the protocol that are separately updated and version controlled are:

Patient information sheets and consent form

GP Letter

LRSQ questionnaires

Participating sites log

Trial Monitoring Plan

Statistical Analysis Plan

Trial Risk Assessment

For peer review only

For peer review only - http://bmjopen.bmj.com/site/about/guidelines.xhtml

COREQ (COnsolidated criteria for REporting Qualitative research) Checklist

A checklist of items that should be included in reports of qualitative research. You must report the page number in your manuscript where you consider each of the items listed in this checklist. If you have not included this information, either revise your manuscript accordingly before submitting or note N/A.

Topic	Item No.	Guide Questions/Description	Reported on Page No.
**Domain 1: Research team and reflexivity**			
Personal characteristics			
Interviewer/facilitator	1	Which author/s conducted the interview or focus group?	
Credentials	2	What were the researcher's credentials? E.g. PhD, MD	
Occupation	3	What was their occupation at the time of the study?	
Gender	4	Was the researcher male or female?	
Experience and training	5	What experience or training did the researcher have?	
Relationship with participants			
Relationship established	6	Was a relationship established prior to study commencement?	
Participant knowledge of the interviewer	7	What did the participants know about the researcher? e.g. personal goals, reasons for doing the research	
Interviewer characteristics	8	What characteristics were reported about the inter viewer/facilitator? e.g. Bias, assumptions, reasons and interests in the research topic	
**Domain 2: Study design**			
Theoretical framework			
Methodological orientation and Theory	9	What methodological orientation was stated to underpin the study? e.g. grounded theory, discourse analysis, ethnography, phenomenology, content analysis	
Participant selection			
Sampling	10	How were participants selected? e.g. purposive, convenience, consecutive, snowball	
Method of approach	11	How were participants approached? e.g. face-to-face, telephone, mail, email	
Sample size	12	How many participants were in the study?	
Non-participation	13	How many people refused to participate or dropped out? Reasons?	
Setting			
Setting of data collection	14	Where was the data collected? e.g. home, clinic, workplace	
Presence of non-participants	15	Was anyone else present besides the participants and researchers?	
Description of sample	16	What are the important characteristics of the sample? e.g. demographic data, date	
Data collection			
Interview guide	17	Were questions, prompts, guides provided by the authors? Was it pilot tested?	
Repeat interviews	18	Were repeat inter views carried out? If yes, how many?	
Audio/visual recording	19	Did the research use audio or visual recording to collect the data?	
Field notes	20	Were field notes made during and/or after the inter view or focus group?	
Duration	21	What was the duration of the inter views or focus group?	
Data saturation	22	Was data saturation discussed?	
Transcripts returned	23	Were transcripts returned to participants for comment and/or	

For peer review only

Topic	Item No.	Guide Questions/Description	Reported on Page No.
		correction?	
**Domain 3: analysis and findings**			
Data analysis			
Number of data coders	24	How many data coders coded the data?	
Description of the coding tree	25	Did authors provide a description of the coding tree?	
Derivation of themes	26	Were themes identified in advance or derived from the data?	
Software	27	What software, if applicable, was used to manage the data?	
Participant checking	28	Did participants provide feedback on the findings?	
Reporting			
Quotations presented	29	Were participant quotations presented to illustrate the themes/findings? Was each quotation identified? e.g. participant number	
Data and findings consistent	30	Was there consistency between the data presented and the findings?	
Clarity of major themes	31	Were major themes clearly presented in the findings?	
Clarity of minor themes	32	Is there a description of diverse cases or discussion of minor themes?	

Developed from: Tong A, Sainsbury P, Craig J. Consolidated criteria for reporting qualitative research (COREQ): a 32-item checklist for interviews and focus groups. *International Journal for Quality in Health Care*. 2007. Volume 19, Number 6: pp. 349 – 357

Once you have completed this checklist, please save a copy and upload it as part of your submission. DO NOT include this checklist as part of the main manuscript document. It must be uploaded as a separate file.

For peer review only

BMJ Open

Stakeholders' perspectives on clinical trial acceptability and approach to consent within a limited timeframe: a mixed methods study.

Journal:	*BMJ Open*
Manuscript ID	bmjopen-2023-077023.R1
Article Type:	Original research
Date Submitted by the Author:	20-Oct-2023
Complete List of Authors:	Deja, Elizabeth; University of Liverpool, Department of Public Health, Policy and Systems<br>Donohue, Chloe; University of Liverpool, Liverpool Clinical Trials Centre<br>Semple, Malcolm; University of Liverpool; Alder Hey Children's Hospital, Respiratory Medicine<br>Woolfall, Kerry; University of Liverpool, Institute of Population, Health and Society<br>Investigators, BESS ; Multiple
**Primary Subject Heading**:	Paediatrics
Secondary Subject Heading:	Intensive care, Paediatrics, Qualitative research, Respiratory medicine, Ethics
Keywords:	QUALITATIVE RESEARCH, ETHICS (see Medical Ethics), Feasibility Studies, Paediatric intensive & critical care < INTENSIVE & CRITICAL CARE, Randomized Controlled Trial, Respiratory infections < THORACIC MEDICINE

SCHOLARONE™
Manuscripts

I, the Submitting Author has the right to grant and does grant on behalf of all authors of the Work (as defined in the below author licence), an exclusive licence and/or a non-exclusive licence for contributions from authors who are: i) UK Crown employees; ii) where BMJ has agreed a CC-BY licence shall apply, and/or iii) in accordance with the terms applicable for US Federal Government officers or employees acting as part of their official duties; on a worldwide, perpetual, irrevocable, royalty-free basis to BMJ Publishing Group Ltd ("BMJ") its licensees and where the relevant Journal is co-owned by BMJ to the co-owners of the Journal, to publish the Work in this journal and any other BMJ products and to exploit all rights, as set out in our licence.

The Submitting Author accepts and understands that any supply made under these terms is made by BMJ to the Submitting Author unless you are acting as an employee on behalf of your employer or a postgraduate student of an affiliated institution which is paying any applicable article publishing charge ("APC") for Open Access articles. Where the Submitting Author wishes to make the Work available on an Open Access basis (and intends to pay the relevant APC), the terms of reuse of such Open Access shall be governed by a Creative Commons licence – details of these licences and which Creative Commons licence will apply to this Work are set out in our licence referred to above.

Other than as permitted in any relevant BMJ Author's Self Archiving Policies, I confirm this Work has not been accepted for publication elsewhere, is not being considered for publication elsewhere and does not duplicate material already published. I confirm all authors consent to publication of this Work and authorise the granting of this licence.

For peer review only

Stakeholders' perspectives on clinical trial acceptability and approach to consent within a limited timeframe: a mixed methods study.

Deja, E[1], Donohue, C[2], Semple, M G[3,4], and Woolfall K[1] for the BESS Investigators.

1 Corresponding author. Dr Elizabeth Deja PhD, Department of Public Health, Policy and Systems, University of Liverpool, Liverpool, UK. E.deja@Liverpool.ac.uk

2 *Liverpool Clinical Trials Centre, University of Liverpool*

3 NIHR Health Protection Research Unit in Emerging and Zoonotic Infections, and Institute of Infection, Veterinary and Ecological Sciences, Faculty of Health and Life Sciences, University of Liverpool, Liverpool, UK

4 Respiratory Medicine, Alder Hey Children's Hospital, Liverpool L12 2AP, UK

Elizabeth Deja, PhD, ORCID 0000-0002-3626-4927,
Chloe Donohue, BSc ORCID 0000-0001-6379-8214
Malcolm G Semple, PhD, ORCID 0000-0001-9700-0418
Kerry Woolfall, PhD, ORCID 0000-0002-5726-5304

Word Count:
3516

Key words

Informed Consent, Qualitative, Intensive Care Units, Bronchiolitis, Randomised Controlled Trials.

Abstract

Objectives: The Bronchiolitis Endotracheal Surfactant Study (BESS) is a randomised controlled trial to determine the efficacy of endo-tracheal surfactant therapy for critically ill infants with bronchiolitis. To explore acceptability of the trial, including approach to consent within a limited time frame, we explored parent and staff experiences of trial involvement in the first two bronchiolitis seasons to inform subsequent trial conduct.

Design: A mixed-method embedded study involving a survey of site staff, alongside questionnaires and interviews with parents approached about BESS.

Setting: Fourteen Paediatric intensive care units in the UK

Participants: Seventy-five parents of 69/179 (38.5%) children recruited to BESS from 13/14 (93%) sites. Of these, 55/69 (78%) completed a questionnaire, and 15/69 (21%) were interviewed. Thirty-eight staff representing all 14 sites completed a questionnaire.

Results: Parents and staff found the trial acceptable. All constructs of the Adapted Theoretical Framework of Acceptability were met. Parents viewed surfactant as being low risk and hoped their child's participation would help others in the future. Although parents supported research without prior consent in studies of time critical interventions, they believed there was sufficient time to consider this trial. Parents recommended that prospective informed consent should continue to be sought for BESS. Many felt that the time between the consent process and intervention being administered took too long and should be 'streamlined' to avoid delays in the administration of trial interventions. Staff described how the training and trial processes worked well, yet patients were missed due to lack of staff to deliver the intervention, particularly at weekends.

Conclusion: Parents and staff supported the BESS trial and highlighted aspects of the protocol, which should be refined, including a streamlined informed consent process. Findings will be useful to inform approaches to consent in future paediatric trials where there is limited time for consent discussions.

Trial registration number: ISRCTN 11746266

Strengths and Limitations of this study

- Use of the Deja et al's (2021) adapted theoretical framework of acceptability allowed trial acceptability to be evaluated as a multifaceted construct as opposed to a poorly defined binary (acceptable/not acceptable) approach.
- Acceptability was explored from multiple perspectives providing an in-depth understanding of key stakeholder views.
- Acceptability was measured at multiple time points. The study may have benefited from more direct feedback from parents who declined their child's involvement in BESS.

Introduction

Bronchiolitis of infancy is a seasonal respiratory viral disease that most commonly causes rhinorrhoea, mild fever, and a wet cough but in severe cases can result in feeding difficulties and respiratory distress. It is the single most common reason for hospital admission of infants (children age <1yr) (1). The youngest of these, those born prematurely and those with underlying conditions are most often and most severely affected (2). There is no vaccine or specific treatment for bronchiolitis (3). Despite advances in the provision of non-invasive modes of respiratory support, admissions to UK Paediatric Intensive Care Units (PICU) and duration of Mechanical Ventilation for life-threatening bronchiolitis have remained fairly static (1), with the exception of during the COVID pandemic which indirectly lead to a decrease in all childhood infections (4).

Pulmonary surfactant is secreted by lung alveolar cells to reduce surface tension so increasing compliance of the lungs, allowing them to inflate more easily and reduce the work of breathing (5). Studies of infants with life-threatening bronchiolitis show reduced lung compliance and relative surfactant deficiency (6-10). The systematic reviews by Jat and Chawla found that surfactant therapy in infants with critical illness due to bronchiolitis was: safe, improved gas exchange and reduced both duration of mechanical ventilation and length of stay on PICU (11, 12).

The Bronchiolitis Endotracheal Surfactant Study (BESS) is a phase 2, blinded randomised controlled trial designed to explore the efficacy and mechanism of surfactant

For peer review only - http://bmjopen.bmj.com/site/about/guidelines.xhtml

therapy compared to air placebo for critically ill infants with bronchiolitis (see supplementary material 1 for protocol). BESS is recruiting in up to 14 PICUs in UK Tertiary hospitals, with the intervention mostly being administered by respiratory physiotherapists. Informed consent was sought from parents/legal representatives (referred to hereon as parents) for their child's participation in BESS. Due to the time-dependent nature of the intervention, randomisation is required within 48 hours of intubation. This meant that urgent action was needed for the purposes of the trial, which is in line with legislation requirements for research without prior consent (5). However, it was unclear if informed consent could be reasonably sought within this limited time frame. In designing the study we identified a need to explore parents' views on the acceptability of seeking informed consent in BESS within a limited time window with a view to consider an alternative approach, such as research without prior consent (6), depending on parents' views and experiences of the recruitment and consent process.

Qualitative research to incorporate staff, patient or parent perspectives in the design of a clinical trial can help ensure the trial is acceptable, appropriate and possible to conduct (7). BESS included an embedded mixed methods study in the first two seasons of the trial to explore staff and parents' perspectives on the acceptability of the trial; approach to recruitment and consent; decision-making in the emergency setting; and barriers to participation. This paper presents the findings from the embedded study to inform ongoing recruitment to BESS and future trials in emergency and critical care settings.

For peer review only - http://bmjopen.bmj.com/site/about/guidelines.xhtml

Methods

Study design

A mixed methods study embedded within a clinical trial (BESS). Questionnaires and interviews with parents approached for informed consent in BESS, and a survey of BESS site staff involved in trial recruitment, consent procedures and intervention administration. We used previous embedded or trial feasibility studies (8-11) and the Adapted Theoretical Framework of Acceptability (ATFA) (12) to inform the design, including sample estimation, recruitment strategy, parent interview topic guide (see supplementary material 2), parent questionnaire and site staff questionnaire (see supplementary material 3). The parent questionnaire followed the same format as those used in similar studies (9, 11, 13) consisting of Likert scale questions from the Decision Making Control Instrument (14) and free text responses, taking on average five minutes to complete (See supplementary material 4).

Preliminary findings from parent interviews in season 1 and 2 (2019/2020) also informed the development the Site Staff Questionnaire, which consisted of 19 questions around the staffs' professional background, views on BESS training, BESS research Process, and BESS consent process. The site staff questionnaire was conducted at the end of season 2.

Patient and Public Involvement

The BESS study and embedded study had Patient and Public Involvement throughout. Six Parents of infants admitted to PICU and ventilated for life-threatening bronchiolitis and one member of the NIHR GenerationR Young Persons Advisory Group co-developed the study design and all study documents, some of which went on to be part of the BESS Parents Advisory Group. Two members are co-applicants and sit on the Trial Management Group (CF) & Trial Steering Committee (EA).

For peer review only - http://bmjopen.bmj.com/site/about/guidelines.xhtml

Representatives of the Paediatric Intensive Care Society as stakeholders in the research output reviewed an early draft and this submitted application.

Parents/legal representatives

Eligibility, recruitment and sampling procedure

All parents recruited to BESS were eligible to take part if they spoke English, including those who declined their child's participation in BESS.

Site staff provided parents with information about the embedded study as part of the BESS Participant Information Sheet (PIS) and recruitment discussions. Staff asked parents if they would like to complete the questionnaire after the recruitment discussion and/or if they were willing to take part in a telephone interview with the BESS researcher (ED) after their child had been discharged from hospital. Parents placed completed questionnaires in a stamped addressed envelope, which were collected by site staff and posted to ED. Data collection continued until thematic saturation, where additional data did not lead to any new major themes identified during analysis. Researchers were also looking for high levels of 'information redundancy' (15-17) and information power, the point when data is deemed to address the study aims; sample specificity, such as experience relevant to the study aims, and sample diversity (15, 18).

Screening and interview conduct

Parents were contacted to arrange a telephone interview within one month of consent after checking each child's discharge and survival status with sites. Initially, parents were contacted in sequential order, then purposively sampled to ensure parents from all sites and decliners to BESS were represented, not just consenters from the first sites opened (the largest population). Informed consent for audio recording of interviews was checked verbally at the time of the interview. The interviews explored views and experiences about: their child's admission to hospital, the BESS consent process, including how and when the trial was introduced by staff; information materials;

and consent decision-making. This included questions about how trial processes could be improved and the potential use and acceptability of research without prior consent in subsequent seasons (see supplementary material 2). Respondent validation was used to add unanticipated topics to the topic guide as interviewing and analysis progressed (17). After the interview was complete, parents were sent a thank you letter, and a £20 Amazon voucher for their time.

Site staff Questionnaire

At the end of season 2, the BESS Trial Manager (CD) sent an email invitation to site staff requesting their participation in the online questionnaire and to cascade the link to colleagues involved in the trial. Email invitations described how completion of the questionnaire was taken as an indication of consent. Reminders were sent by (CD) and the BESS Chief Investigator (MGS) to all site key contacts after one week.

Analysis

Digital audio recordings were transcribed verbatim by a professional transcription company (UK Transcription, Brighton) and anonymised. Qualitative analysis of interviews and open response questionnaire data was interpretive and iterative (19). Utilising a thematic analysis approach, the aim was to provide an accurate representation of views on trial acceptability, design and processes (20, 21) (see supplementary material 5). NVivo 12 software (QSR International Pty Ltd., Melbourne, Australia) was used to assist in the organisation and coding of data. Data from the parent and staff questionnaires were cleaned and entered into SPSS Version 24.0 (IBM Corp., Armonk, NY, US). Descriptive statistics are presented with percentages. Synthesis of qualitative and quantitative data drew on the constant comparative method (22, 23). This involved ED (PhD, female psychologist,

For peer review only - http://bmjopen.bmj.com/site/about/guidelines.xhtml

Research Associate) and KW (PhD, female, social scientist, Reader) looking across quantitative and qualitative themes and quantitative output for themes/data output related to trial acceptability.

A final stage of analysis involved consideration of both qualitative and quantitative findings against the ATFA (12, 24) to help conceptualise and discuss the overall acceptability of the proposed trial.

The ATFA is designed to assist researchers in assessing the acceptability of healthcare interventions including paediatric clinical trials. The framework explores eight aspects of acceptability at three possible time points, before (*prospective*), during (*concurrent*) and after (*retrospective*) the intervention (See supplementary material 6). Our data were a mix of *Concurrent* and *Retrospective* data (see Tables 1 & 2).

RESULTS

Sample

A total of 75 parents of 69/179 children (38%) from 13/14 (93%) BESS sites took part in the embedded study (see Figure 1). Of these, 55/69 (78%) completed a questionnaire, 10/69 (15%) took part in an interview and 5/69 (7%) took part in both methods. Thematic saturation was reached after eight interviews. An additional seven interviews were conducted to ensure variance across BESS sites and achieve information power (15). Parents were interviewed on average (mean) 27 days (SD 7.8 days, range 17-43 days) after randomisation. The mean duration of PICU admission was 8 days (range 2.5-18 days), with a mean of 7 days (range 2.5-17 days) on a ventilator and a mean age of 70 days old (range 15-156 days). Interviews took on average (mean) 37 minutes (SD 9.6 minutes, range 20-57).

For peer review only - http://bmjopen.bmj.com/site/about/guidelines.xhtml

Of the 42 (25%) families that declined to take part in BESS, one mother completed a questionnaire. We left recruitment open for decliners to complete questionnaires for the proceeding season. To gain some insight into reasons for declining consent we used case report form and interview data from site staff. Due to the COVID-19 pandemic, there was a two-year hiatus in the BESS study.

Thirty-nine staff at all 14 open sites BESS sites completed the online questionnaire including 14 doctors, 12 Physiotherapists, 9 Research nurses and 1 nurse. All participants apart from the site trial coordinator were involved in the clinical care of children with varied levels of experience of recruiting to paediatric clinical trials (range 0-20 years, median 4 years).

Figure 1. Parent recruitment [insert]

Main findings

Acceptability of the BESS trial

Staff supported the trial, meeting all eight components of the ATFA (as shown in table 2). They had a clear understating of the aim of the study and how to follow the protocol. They viewed the administration of the intervention as being acceptable and ethical with free text comments highlighting how the trial had been: *"well received by PICU staff and parents"* (P31, Research nurse). Indeed, parents described their support for the trial. Many appeared to view BESS as an extension of standard care as the physiotherapy and tests conducted may have occurred anyway at different times in their child's clinical care. Consent decisions appeared to be informed (see below) as parents understood what BESS was investigating and why. They described their child's involvement as being low risk and were reassured by information about how surfactant was used in premature babies with very few side effects:

For peer review only - http://bmjopen.bmj.com/site/about/guidelines.xhtml

"I asked if there would be any side effects and things like that, and she said because it wasn't a new drug, she said there haven't been on the ones they had used it on previously, that they used it on premature babies." P04, mother, interview.

Participation was not considered to place an additional burden on the child, although some parents found physiotherapy uncomfortable to watch and chose to leave the room:

"*A lot of his physios, I was watching one where they'd, like, shake your baby. (Laughter) Then they push down on their ribs like they compress the ribs. I'm sure that that's what they were doing before they were going to possibly give the placebo or the surfactant. … It's frightening; I left… Because I thought, they don't need a panicky mum around them.*" P14, mother, interview.

Recruitment and consent in an emergency setting

Early in the interview, parents were asked to explain their understanding of the BESS Study. Whilst the depth of descriptions varied, all gave a clear and accurate account of the purpose of the trial. Many outlined primary and secondary outcomes, including how surfactant might: "*reduce the amount of time on a ventilator"* (P12, mother, interview), prevent: "*lungs from sticking*" (P09, father, interview), help them: "*to expand and open their lungs*" (P02, mother, interview) and hopefully help "*them recover quicker*" (P10, father, interview) therefore reducing parental and child distress, cost, ventilator related infections.

Despite being approached soon after their child's admission to PICU, all interview and questionnaire participants stated that were given sufficient time to consider the information and ask questions and that staff had broached BESS at '*an appropriate time*' (100%, questionnaire item) (see Tables 1 and 2). Parents described being able to make an informed consent decision about the trial, despite the highly emotive and stressful situation:

"*I guess the main elephant in the room is, do I feel as if, because I was in a stressful situation, an emotional situation, where I wasn't in my right mind, where I'm having to deal with a lot*

For peer review only - http://bmjopen.bmj.com/site/about/guidelines.xhtml

of different, very scary, very emotional, very worrying situations, was I making clear and conscious decisions? The answer is yes, I was" P13, father, interview.

A total of 19/179 (10%) declined their child's participation in the BESS study. Reasons given for not taking part included situational capacity as there was: "*too much already going on*." (Mother, questionnaire, decliner) and: '*do not want to take part in research*' (case report form). Others declined as their child was close to coming off the ventilator at the time of final consent. A minority of parents '*did not want their child to receive surfactant*' (case report form), raising concerns with staff about the potential risks of: *"using something unproven when their child was sick"* (P28, Physiotherapist). However, most: *"Families were much more open to surfactant than I expected*." (P19, Research nurse) "*especially if their child had been premature and had surfactant in the neonatal period*." (P10, Physiotherapist). One nurse noted that the number of decliners was similar to other PICU trials: *"Few exceptions where they didn't want child as a guinea pig, but no more than usual"* (P6, Nurse).

Facilitating quicker intervention delivery

Both groups highlighted that sometimes there were relatively long stretches of time between initial trial recruitment discussions and trial interventions beginning. The length of time was dependent on the availability of research nurses (who largely work 9am-5pm on weekdays) and parents for the consent discussions and availability of physiotherapists to administer the intervention: "*delaying the whole process*" (P15, Research nurse):

"I think the only thing that could have possibly done better was doing it sooner because..potentially, it [the intervention] could have started a day earlier. The actual time between them approaching me, to me to find out what it is, to me to say yes and then actually starting the trial, I feel that could have been really streamlined" P13, father, interview.

For peer review only - http://bmjopen.bmj.com/site/about/guidelines.xhtml

"Low on personnel on the ground as medics are undertaking the intervention which takes considerable time in a busy unit" P06, Doctor

In addition to increasing the: *"potential for patients to begin weaning from the ventilator and therefore not be eligible"* (P23, Research nurse). During interviews, parents questioned the ethicality and impact of delaying a potentially effective treatment they had consented to:

"If there was someone on hand to make it happen despite the shift change [...] because you want the test to actually be effective if you're going to take part, don't you? You don't want to waste time." P11, father, interview.

Parents provided practical suggestions for speeding up the recruitment and consent process, for example having the written information available at the bedside so that they would likely have read it before the first approach allowing for those comfortable to consent without a follow-up discussion. Therefore, removing a step in the recruitment process timeline. Additionally, they highlighted the need to have more staff available that can take consent and administer the intervention.

Perceptions of Research without Prior Consent in BESS

Figure 2 Description of RWPC read to participants during interview [Insert]

Towards the end of the interviews, a definition of research without prior consent (RWPC) was read to parents (Figure 2). All parents responded positively to the use of RWPC in critical care research in emergency situations for trials that were regarded as being low risk to their child's wellbeing.

"As long as it's something that doesn't have any risks associated with it, or there wouldn't be a reason why you wouldn't want them to do it and it's something that's going to help them, then I think it's (RWPC) a good thing." P08, mother, interview.

For peer review only - http://bmjopen.bmj.com/site/about/guidelines.xhtml

Five parents described consenting to another trial that had used a RWPC approach during the same hospital admittance. Nevertheless, the majority (n = 11/ 15, 74%) of parents interviewed suggested that BESS should continue to use an informed consent process as the intervention was not viewed to be time critical in that surfactant was regarded as something to hopefully get children "*off the ventilator quicker" rather than an emergency intervention that would be lifesaving "If we don't give this to her immediately"* (P08, mother, interview). In contrast, three parents were in favour of RWPC in the BESS study and there were no strong objections against its possible use, as the intervention was viewed to be low risk, would facilitate the intervention being given more quickly, thus avoiding missed patients and increasing recruitment rates. As the following quote illustrates:

> "if you'd had just cracked on and done it, it could've made a difference quicker, but by the time we got around to the actual consent, he was being extubated" P05, mother, interview

For peer review only - http://bmjopen.bmj.com/site/about/guidelines.xhtml

Table 1. Parent concordant acceptability of BESS mapped to the adapted framework of acceptability Deja E. et al., 2021

Affective attitude	Burden	Ethicality	Intervention coherence	Opportunity costs	Self-efficacy	Perceived effectiveness	Trust
135/ 179 (75 %) consented for their child to take part in BESS.	50/60 (83%) did not find it difficult to take in information about BESS<br><br>47/60 (78%) did not find it difficult to make a decision about BESS<br><br>“We felt the process was straightforward” MQ34	60/60 (100%) agreed that they were satisfied with the consent process<br><br>55/60 (92%) felt that medical studies like BESS are important	60/ 60 (100%) agreed that the Information received about BESS was clear and straightforward to understand	No relevant data was reported at this time point	60/67 (95%) felt that they made the decision for their child to take part in the pilot trial.<br><br>54/60 (90%) felt in control of the decision to take part.<br><br>“medical team made it clear that this decision was mine fully and I could withdraw consent at any time. This knowledge made participating much easer.’ MQ45	50/53 (88.3%) Selected ‘helping my child’ as a reason for taking part<br><br>57/60= 95% selected ‘to help other children in the future’ as a reason for taking part<br><br>“I think all children would benefit from this great work thanks” FQ42	45/60 (75%) selected ‘because I trusted the doctor or nurse who explained BESS’ as a reason for taking part. *<br><br>“Trusting the doctor or nurse explaining BESS to parents is going to be an important deciding factor subconsciously for most parents” FQ30
9/179 declined to take part because they did not want their child to receive porcine surfactant<br><br>19/179 Do not want to take part in research	“My partner & I decided it was too much to take on when our son is so sick, too much already going on.” MQ53- decliner’						

* note, not selecting this *did not mean* that they did not trust the staff, it was just not a identified reason for their consent.

Tables Key: Shaded fields highlight potentially unacceptable aspects of the trial MQ mother open questionnaire response, MF: father open questionnaire response. MI: mother interview, FI Father interview.

Table 2. Retrospective acceptability of BESS mapped to the adapted framework of acceptability Deja. E et al., 2021

Affective attitude	Burden	Ethicality	Intervention coherence	Opportunity costs	Self-efficacy	Perceived effectiveness	Trust
Parental Acceptability							
Happy with trial "obviously I was approached for the BESS trial, which I was quite happy for him to take part in."MI02	'But the standard of care for the placebo is just normal care anyway' MI06	'You feel powerless, to be entered into a research study without somebody talk to you about it you've got even more power taken away from you.' MI12<br>"you want the test to actually be effective if you're going to take part, don't you? You don't want to waste time." FI11	"If they can do a trial that shows that actually giving the surfactant or not brings them out of it quicker" MI04<br><br>'literally any question I had in my head, this leaflet then had contained the answers" MI05	"there are not really risks, so there's not really any problem, all it could possibly do is produce positives. FI13	"She was so pleasant and she was so easy to talk to. Even when I wasn't discussing the paperwork and the trial with her, if I had any questions, I could easily walk up and ask, if I had any enquiries. She was very, very easy to approach." MI02	"then it was a realisation of, oh, my God, so you've just told me this thing that could make this difference and could get him off the ventilator quicker, and he might not get it anyway' MI05	'I don't think they'd put your babies in danger. They are here to get them better, so you just kind of trust in what they are doing." MI4
Staff Acceptability							
Overall how acceptable do you find the BESS study? 20/36 (53%) very acceptable 18/36 (47%) acceptable<br><br>"If it will have a clinical benefit which outweighs potential risk, then it is definitely acceptable" (P14)	*"Low on personnel on the ground as medics are undertaking the intervention which takes considerable time in a busy unit"* (P06, Doctor)	10/36 (26%) found it very acceptable and 28/36 (74%) acceptable to administer the surfactant.<br><br>6 /36 (16%) found it very acceptable and 31/36 (82%) found it acceptable to administer the placebo	The majority of staff rated the training as 'excellent' (27/36, 75%) or 'good' (9/36, 25%).	*"We tried to time other interventions that required muscle relaxants e.g. ETT tape changes with the time the placebo needed to be given"* (P39, Physiotherapist).	34/36 (87%) staff indicated that they had not experienced difficulties adhering to the BESS protocol.	"I think it's important that we look at ways to improve care and treatment so research trials are vital. Reducing ventilator days would help free up precious ITU beds during winter and hopefully reduce problems with lung injuries" (P17)	Not mentioned in questionnaire responses

Discussion

The aim of this study was to explore the acceptability of the BESS Study to help inform recruitment in subsequent bronchiolitis seasons and trials in other paediatric critical care settings. The Adapted Theoretical Framework of Acceptability (12) allowed us to explore the multifaceted construct of acceptability, with eight components to consider. Our data suggest that the BESS Study met all eight of the constructs. BESS was viewed by parents and PICU staff as understandable, posing low to no risk and possible benefits to child participants.

Our findings suggest that both parents and staff viewed the BESS study as ethical and potentially beneficial to children with bronchiolitis. However, they highlighted that the length of the consent process and staff availability impacted the timeliness of administering the BESS intervention. They were concerned that such delays may reduce the potential effectiveness of the intervention. At the study design stage, there was uncertainty about whether it was more appropriate to use RWPC or an informed consent approach to recruitment in BESS due to the critical care context and the need to administer the intervention in a limited period. However, our findings support the continued use of informed consent in this context, though RWPC was supported by a minority of parents as it would reduce the delay of intervention. BESS interventions need to be given promptly with a short window for trial recruitment discussions with parents. The low-risk nature means that parents felt they could make an informed decision after one conversation when the child is stable. This with other suggested changes to study processes should allow the full intervention to be administered in a timelier manner.

The seasonal nature of bronchiolitis provided an opportunity to review and adapt trial processes, increasing opportunities to make the trial more acceptable acceptability and feasibility to conduct. However, most trials do not have this ability leading to potential research and resource waste (25). Our findings highlight the importance of pre-trial research or embedded studies in which the potential time windows between randomisation and intervention delivery can be clearly mapped and parent views on the most appropriate consent approach are fully explored.

For peer review only - http://bmjopen.bmj.com/site/about/guidelines.xhtml

The main strength of this study is that acceptability was measured at multiple time points, from different perspectives providing an in-depth understanding of key stakeholder views. The study may have benefited from more direct feedback from parents who declined their child's involvement in BESS (26). We kept recruitment open for decliners for an additional season to try to address this gap in knowledge. However, their wish to 'not take part in research' and 'situational incapacity' in BESS, also applied to the embedded study and was compounded by low recruitment in season three as a consequence of COVID (4). We believe that the data from staff, the case report form and the one decliner questionnaire gave us an insight into reasons for declining consent. In addition, the overall number of decliners was low. Therefore, this missing data should not have a large impact on our findings.

Conclusion

Our paper found BESS to be feasible and acceptable, supported the continued use of informed consent and highlighted areas for study process improvements. Future trials should consider proportionate consent processes when interventions are low risk and there is a short window for informed consent discussions.

For peer review only - http://bmjopen.bmj.com/site/about/guidelines.xhtml

Acknowledgments

We firstly thank the children and families who participated in the BESS. Without your help this research could not have taken place. We also thank the principal investigators, respiratory physiotherapists and research nurses at each study site.

We would also like to thank the National Institute for Health Research's Efficacy and Mechanism Evaluation (EME) Programme (ref: 12/205/28) for funding this research.

Author contributions

MGS Chief Investigator; led the conception and design of the BESS study, KW designed and led the embedded study. CD facilitated the acquisition of data for analysis and trial management. ED conducted the interviews. ED and KW analysed and interpreted the data. ED and KW drafted the manuscript. ED, KW, MGS, CD and the BESS Investigators reviewed the manuscript critically for important intellectual content and provided approval of the version to be published. BESS Investigators' other contributions can be seen in the supplementary material.

BESS Investigators

Chief Investigator: Malcolm G Semple PhD, (Department of Clinical Infection, Microbiology and Immunology, Institute of Infection, Veterinary and Ecological Sciences, University of Liverpool and Department of Respiratory Medicine, Alder Hey Children's NHS Foundation Trust, Liverpool)

Deputy Chief Investigator: Paul S McNamara PhD, (Department of Women's and Children's Health, Institute of Life Course and Medical Sciences, University of Liverpool and Department of Respiratory Medicine, Alder Hey Children's NHS Foundation Trust, Liverpool)

Coinvestigators: Evette Allen PhD, Clare Fowler HNC (Parent Lay Representatives), Catrin Barker PGDipClinPharm, Kentigern Thorburn MD, Matthew Peak PhD, Paul C Ritson MSc, Clare van Miert PhD, (Alder Hey Children's NHS Foundation Trust, Liverpool), Ashley Best MSc, Chloe Donohue BSc, Ashley Jones PhD, Tracy Moitt, Laura Price BSc, Paula Williamson PhD (Liverpool Clinical Trials Centre, University of Liverpool, Liverpool), Howard W Clark PhD, Jens Madsen PhD (Elizabeth Garrett Anderson Institute for Women's Health, University College London, London), Anne Dawson, Colin Summers (Faculty of Health and Life Sciences, University of Liverpool, Liverpool), Elizabeth Deja PhD, Kerry Woolfall PhD (Institute of Population Health, University of Liverpool), Blessing Osaghae PhD, Mark Turner PhD (Department of Women's and Children's Health, Institute of Life Course and Medical Sciences, University of Liverpool), Madhuri Panchal BSc, Anthony Postle PhD, (National Institute for Health Research Southampton Biomedical Research Centre Clinical and Experimental Sciences, Faculty of Medicine, University of Southampton, Southampton), John Pappachan MBBChir, (Southampton Children's Hospital, Southampton), Roger Parslow PhD (Division of Epidemiology and Biostatistics, Leeds Institute of Cardiovascular and Metabolic Medicine, University of Leeds, Leeds), Jennifer Preston BA, (NIHR Alder Hey Clinical Research Facility, University of Liverpool, Liverpool)

For peer review only - http://bmjopen.bmj.com/site/about/guidelines.xhtml

Site Investigators:

Alder Hey Children's Hospital, Liverpool: Kentigern Thorburn MD (Principal Investigator), Vanessa Compton, Peter Jirasek, Dawn Jones, Michael Mander, Laura O'Malley, Laura Rad, Laura Rimmer, Paul C Ritson MSc, Chris Simons.

Birmingham Children's Hospital, Birmingham: Afeda Mohamed Ali MBBCh, Kevin P Morris MD (Principal Investigators), Cara Alexander BSc, Wendy Browne MSc, Hannah Child BSc, Stephanie Clarke BSc, Sarah Fox BN, Natalie Milburn BSc, Philip Milner FIBS, Samantha Owen BSc, Holly Parkin BSc, Harriet Payne BSc, Carly Tooke, Helen Winmill MSc.

Bristol Royal Hospital for Children, Bristol: Peter J Davis MBChB (Principal Investigator), Katherine Baptiste, Sophie Coles BSc, Sarah-Jayne Eames BSc, Christina Linton BSc, Helen Marley BSc, Sarah Mogan BSc, Alvin Schadenberg PhD, John Stiven BSc.

Great North Children's Hospital, Newcastle: Rachel Agbeko PhD (Principal Investigator), Rob Claydon PgDip, Christine Mackerness, Anna Stancombe BSc, Kate Teeley BSc, Grace Williamson MD.

Leeds Children's Hospital, Leeds: Santosh Sundararajan MD (Principal Investigator), Ramesh Kumar, Kathryn Reeves BSc, Emily Scriven.

Leicester Children's Hospital, Leicester: Raghu N Ramaiah MBBS (Principal Investigator), Rekha Patel.

Nottingham Children's Hospital, Nottingham: Patrick E Davies BMBS (Principal Investigator), Lindsay Crate, Simon Gates

Oxford Children's Hospital, Oxford: James Weitz MBBS (Principal Investigator), Kirsten Beadon BSc, Rachel McMinnis BSc, Zoe Oliver BSc, Avishay Sarfatti BMBCh, Frances Sinfield, Charlotte Thompson.

Royal Belfast Hospital for Sick Children, Belfast: Julie Richardson MBChB (Principal Investigator), Hilary Callaghan BSc, Vicki Linton BSc, Jeremy Lyons MD, Clara Nelson BSc, Mark Terris MBChB

Royal Hospital for Children and Young People, Edinburgh: Tsz-Yan Milly Lo PhD (Principal Investigator), David Armstrong MD, Steve Cunningham PhD, Jackie McCormick, Andrea Wood MSc.

Royal Hospital for Children, Glasgow: Richard Levin MBChB, (Principal Investigator), Edgar Brincat MD, Christopher Lamb RN, Ross Marscheider.

Royal Manchester Children's Hospital, Manchester: Stephen D Playfor DM (Principal Investigator), Bernadette C Gavin MSc, Dave J Morgan MSc, Lara T Bunni MSc, Claire F Jennings MClinRes, Rebecca Marshall DipHE(Nur), Emma K Riley MNurSci.

Southampton Children's Hospital, Southampton: John Pappachan MBBChir, Ahmed Osman MB ChB (Principal Investigators), Lorena Caruana, Hannah Clarke, Amber Cook BSc, Tracey Curtis, Nichola Etherington, Michael Griksaitis, Jenni McCorkell BSc, Christie Mellish, Simone Paulson BMBCh, Jenny Pond BSc, Catherine Postlethwaite DipPaedNur.

Staffordshire Children's Hospital, Stoke: Constantinos Kanaris PhD (Principal Investigator), John Alexander MBChB, Nicola McClelland, Holly Minchin, Helen Parker, Pavanasam Ramesh, Joanne Tomlinson.

The Royal London Children's Hospital, London: Simona Lampariello MD (Principal Investigator), Bessie Cipriano, Nosheen Khalid MSc, Ramiya Kirupananthan MBBS, Craig Knott MBBS, Tara Murray,

For peer review only - http://bmjopen.bmj.com/site/about/guidelines.xhtml

Olivia Nugent, Nicolene Plaatjies, Samantha Reed, Natasha Roberts, Christa Ronan, Salman Siddiqi MB BS, Natasha Thorn BMBS

Funding

This work was supported by the National Institute for Health Research's Efficacy and Mechanism Evaluation (EME) Programme (ref: 12/205/28).

Conflicts of Interests

MGS**:** Independent external and non-remunerated member of Pfizer's External Data Monitoring Committee for their mRNA vaccine program(s); HMG UK Scientific Advisory Group for Emergencies (SAGE), COVID-19 Response, Non-remunerated independent member March 2020 to March 2022. HMG UK New Emerging Respiratory Virus Threats Advisory Group (NERVTAG), Non-remunerated independent member since 2014; Integrum Scientific LLC, Greensboro, NC, USA, Chair of Infectious Disease Scientific Advisory Board; MedEx Solutions Ltd, Director; Integrum Scientific LLC, Greensboro, NC, USA, Minority Owner: MedEx Solutions Ltd, Majority Owner.

Ethics Statement

Ethical approval for the study was given by the South Central Berkshire Research Ethics Committee (IRAS ID 220853).

Data sharing statement

No data are available. The datasets generated during and/or analysed during the current study are not publicly available as consent was not sought for data sharing**.**

Declaration.

This manuscript has been read and approved by all the authors, the requirements for authorship have been met, and each author believes that the manuscript represents honest work.

References

1. Green CA, Yeates D, Goldacre A, Sande C, Parslow RC, McShane P, et al. Admission to hospital for bronchiolitis in England: trends over five decades, geographical variation and association with perinatal characteristics and subsequent asthma. Arch Dis Child. 2016;101(2):140-6.
2. Auroux B, Hascoët J, Jarreau P, Magny J, Rozé J, Saliba E, et al. Risk factors for bronchiolitis hospitalization in infants: A French nationwide retrospective cohort study over four consecutive seasons (2009-2013). PLOS ONE. 2020;15(3).
3. Bronchiolitis N. Diagnosis and Management of Bronchiolitis in Children, Clinical Guideline NG9. National Institute for Clinical Excellence (NICE). 2015.
4. Kadambari S, Goldacre R, Morris E, Goldacre MJ, Pollard AJ. Indirect effects of the covid-19 pandemic on childhood infection in England: population based observational study. bmj. 2022;376.
5. (2020).
6. Woolfall K, Frith L, Dawson A, Gamble C, Lyttle MD, Young B. Fifteen-minute consultation: an evidence-based approach to research without prior consent (deferred consent) in neonatal and paediatric critical care trials. Archives of disease in childhood Education and practice edition. 2016;101(1):49-53.
7. O'Cathain A, Hoddinott P, Lewin S, Thomas KJ, Young B, Adamson J, et al. Maximising the impact of qualitative research in feasibility studies for randomised controlled trials: guidance for researchers. Pilot and Feasibility Studies. 2015;1(1):1-13.
8. Molyneux S, Njue M, Boga M, Akello L, Olupot-Olupot P, Engoru C, et al. 'The Words Will Pass with the Blowing Wind': Staff and Parent Views of the Deferred Consent Process, with Prior Assent, Used in an Emergency Fluids Trial in Two African Hospitals. PLoS ONE. 2013;8(2):e54894.
9. O'Hara CB, Canter RR, Mouncey PR, Carter A, Jones N, Nadel S, et al. A qualitative feasibility study to inform a randomised controlled trial of fluid bolus therapy in septic shock. Arch Dis Child. 2017:archdischild-2016-312515.
10. Woolfall K, Frith L, Gamble C, Gilbert R, Mok Q, Young B. How parents and practitioners experience research without prior consent (deferred consent) for emergency research involving children with life threatening conditions: a mixed method study. BMJ Open. 2015;5(9):e008522.
11. Peters MJ, Woolfall K, Khan I, Deja E, Mouncey PR, Wulff J, et al. Different temperature thresholds for antipyretic intervention in critically ill children with fever due to infection: the FEVER feasibility RCT. Health Technology Assessment 2019;23(5):1 - 148.
12. Deja E, Peters MJ, Khan I, Mouncey PR, Agbeko R, Fenn B, et al. Establishing and augmenting views on the acceptability of a paediatric critical care randomised controlled trial (the FEVER trial): a mixed methods study. BMJ Open. 2021;11(3):e041952.
13. Appleton RE, Rainford NEA, Gamble C, Messahel S, Humphreys A, Hickey H, et al. Levetiracetam as an alternative to phenytoin for second-line emergency treatment of children with convulsive status epilepticus: the EcLiPSE RCT. 2020;24:58.
14. Miller VA, Ittenbach RF, Harris D, Reynolds WW, Beauchamp TL, Luce MF, et al. The Decision Making Control Instrument to Assess Voluntary Consent. Medical Decision Making. 2011;31(5):730-41.
15. Saunders B, Sim J, Kingstone T, Baker S, Waterfield J, Bartlam B, et al. Saturation in qualitative research: exploring its conceptualization and operationalization. Quality & Quantity. 2018;52(4):1893-907.
16. Baker SE, Edwards R. How many qualitative interviews is enough? National Centre for Research Methods (NCRM): . 2012.
17. Mays N, Pope C. Assessing quality in qualitative research. Bmj. 2000;320(7226):50-2.
18. Malterud K, Siersma V, Guassora A. Sample Size in Qualitative Interview Studies. Qualitative Health Research. Sage Journals. 2016;26(13):1753-60.
19. Braun V, Clarke V. What can "thematic analysis''offer health and wellbeing researchers? International journal of qualitative studies on health and well-being. 2014;9:26152.

For peer review only - http://bmjopen.bmj.com/site/about/guidelines.xhtml

20. Byrne D. A worked example of Braun and Clarke’s approach to reflexive thematic analysis. Quality & Quantity. 2021:1-22.
21. Byrne D. A worked example of Braun and Clarke’s approach to reflexive thematic analysis. Quality & Quantity. 2021.
22. Glaser BG. The constant comparative method of qualitative analysis. Social problems. 1965;12(4):436-45.
23. Strauss A, Corbin J. Basics of qualitative research techniques. 1998.
24. Sekhon M, Cartwright M, Francis JJ. Acceptability of health care interventions: A theoretical framework and proposed research agenda. British Journal of Health Psychology. 2018;23(3):519-31.
25. Morgan B, Hejdenberg J, Hinrichs-Krapels S, Armstrong D. Do feasibility studies contribute to, or avoid, waste in research? PloS one. 2018;13(4):e0195951.
26. Hughes-Morley A, Young B, Hempel RJ, Russell IT, Waheed W, Bower P. What can we learn from trial decliners about improving recruitment? Qualitative study. Trials. 2016;17(1):1-13.

For peer review only - http://bmjopen.bmj.com/site/about/guidelines.xhtml

Figure Legends

Figure 1. Parent Recruitment

Figure 2. Description of RWPC read to participants during interview

Table 1. Parent concordant acceptability of BESS mapped to the adapted framework of acceptability Deja E. et al., 2021

Table 2. Retrospective acceptability of BESS mapped to the adapted framework of acceptability Deja. E et al., 2021

For peer review only - http://bmjopen.bmj.com/site/about/guidelines.xhtml

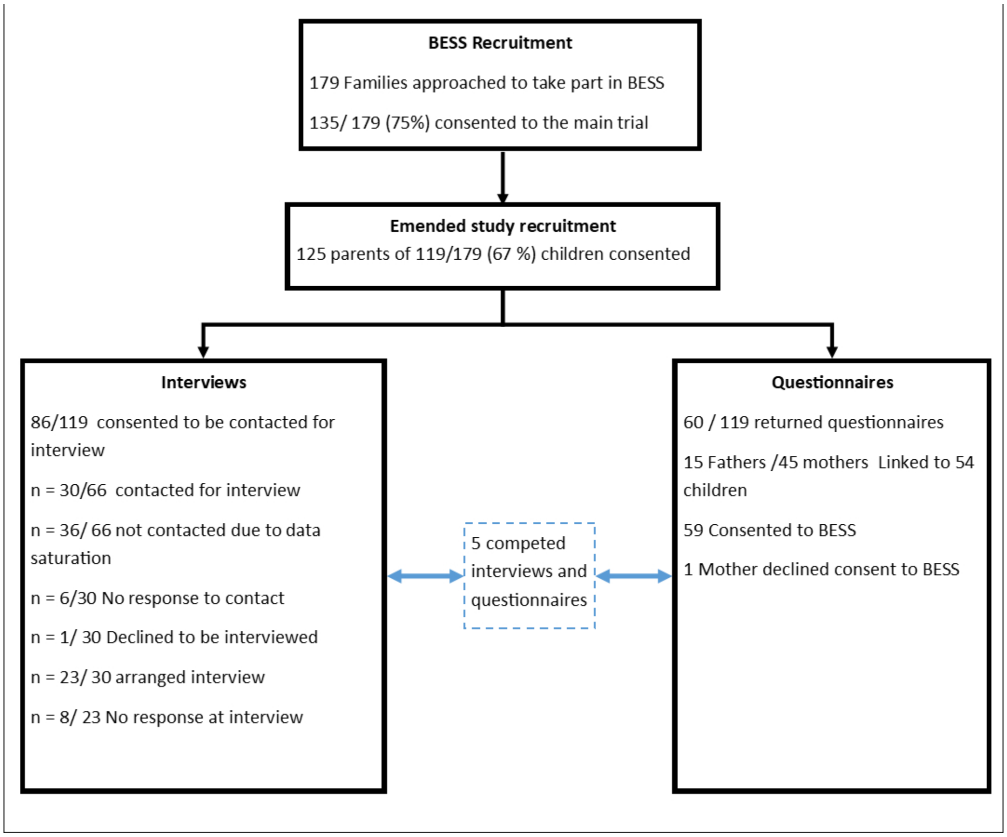

Figure 1. Parent Recruitment

181x150mm (150 x 150 DPI)

In BESS and most research, consent is obtained before children are entered into a study. In some trials conducted in emergency situations parents are asked to provide consent after their child had already been actively involved in the Trial. At this point consent is sought to continue in the trial. This is known as research without prior consent or deferred consent.

The reason that this legislation is in place is because in situations like this (ie, in paediatric critical care), there's a belief that there's no time to have a full discussion and time to reflect about the research and that actually having that discussion might delay treatment.

Figure 2. Description of RWPC read to participants during interview

433x114mm (59 x 59 DPI)

BESS
Bronchiolitis Endotracheal Surfactant Study

The efficacy and mechanism of surfactant therapy for critically ill infants with bronchiolitis: The **B**ronchiolitis **E**ndotracheal **S**urfactant **S**tudy (BESS)

For peer review only

A phase-2 blinded randomised air-placebo-controlled trial of endotracheal surfactant (poractant alfa) in critically ill infants with bronchiolitis

BESS Protocol v2.0 28/09/2018

Study Sponsor(s):
University of Liverpool
Research Support Office
2nd Floor Block D Waterhouse
3 Brownlow Street
Liverpool
L69 3GA

EudraCT number: 2018-001169-18
ISRCTN :
Research Ethics Ref: 18/SC/0427
Sponsor Ref: UoL001360
IRAS Ref: 220853

UNIVERSITY OF LIVERPOOL

CTRC
Clinical Trials Research Centre

NHS
National Institute for Health Research

For peer review only - http://bmjopen.bmj.com/site/about/guidelines.xhtml

PROTOCOL APPROVAL

I, the undersigned, hereby approve this clinical study protocol:

Authorised by Chief Investigator:

Signature: ______________________ **Date:** 28092018

Prof. Malcolm (Calum) Gracie Semple, PhD, BM BCh (Oxon), FRCPCH, FRCPE, FHEA
Consultant in Paediatric Respiratory Medicine, Alder Hey Children's NHS Foundation Trust
Professor of Child Health and Outbreak Medicine, University of Liverpool
Institute in The Park,
Alder Hey Children's Hospital
Eaton Road
Liverpool
L12 2AP

Authorised on behalf of Sponsor:

Signature: ______________________ **Date:** ____________

Mr Alex Astor
Head of Research Support
Research Support Office
2nd Floor Block D Waterhouse Building
3 Brownlow Street,
Liverpool
L69 3GL

Authorised on behalf of the Lead Statistician:

Signature: ______________________ **Date:** ____________

Dr Ashley Jones, PhD
Senior Statistician
Clinical Trials Research Centre
Institute of Child Health
Alder Hey Children's Hospital
Eaton Road
Liverpool
L12 2AP

For peer review only - http://bmjopen.bmj.com/site/about/guidelines.xhtml

PROTOCOL APPROVAL

I, the undersigned, hereby approve this clinical study protocol:

Authorised by Chief Investigator:

Signature: ______________________ **Date:** ____________

Prof. Malcolm (Calum) Gracie Semple, PhD, BM BCh (Oxon), FRCPCH, FRCPE, FHEA
Consultant in Paediatric Respiratory Medicine, Alder Hey Children's NHS Foundation Trust
Professor of Child Health and Outbreak Medicine, University of Liverpool
Institute in The Park,
Alder Hey Children's Hospital
Eaton Road
Liverpool
L12 2AP

Authorised on behalf of Sponsor:

Signature: ______________________ **Date:** 19/10/18

Mr Alex Astor

Head of Research Support
Research Support Office
2nd Floor Block D Waterhouse Building
3 Brownlow Street,
Liverpool
L69 3GL

Authorised on behalf of the Lead Statistician:

Signature: ______________________ **Date:** 28/9/18.

Dr Ashley Jones, PhD
Senior Statistician
Clinical Trials Research Centre
Institute of Child Health
Alder Hey Children's Hospital
Eaton Road
Liverpool
L12 2AP

For peer review only - http://bmjopen.bmj.com/site/about/guidelines.xhtml

General Information

This document describes the BESS trial including detailed information about procedures and recruitment. The protocol should not be used as an aide-memoir or guide for the treatment of other patients; every care was taken in its drafting, but corrections or amendments may be necessary. These will be circulated to the registered investigators in the trial, but centres entering patients for the first time are advised to contact the coordinating centre (Clinical Trials Research Centre (CTRC), University of Liverpool) to confirm they have the most up to date version. Clinical problems relating to this trial should be referred to the relevant Chief Investigator, Professor Calum Semple, via the CTRC.

This protocol defines the participant characteristics required for study entry and the schedule of treatment and follow-up. Participant recruitment will be undertaken in compliance with this document, applicable regulatory and governance requirements and waivers to authorise non-compliance are not permitted.

Incidents of protocol non-compliance, whether reported prospectively (e.g. where a treatment cannot be administered on a scheduled date as a result of public holidays) or retrospectively noted (e.g. as a result of central monitoring) are recorded as protocol deviations, the incidence of which are monitored and reported to trial oversight committees.

The template content structure is consistent with the SPIRIT (Standard Protocol Item: Recommendations for Interventional Trials 2013) and has regard for the Health Research Authority guidance. Regulatory and ethical compliance information is located in section 12.

Relationship Statements

Roles and responsibilities are described in section 15

The University of Liverpool is the Sponsoring organisation. The University of Liverpool will formally delegate specific sponsoring roles to the Chief Investigator and Clinical Trials Unit, but remains legally responsible for the trial.

Clinical Trials Unit (CTU): The CTRC at the University of Liverpool in collaboration with the Chief Investigator, Prof Calum Semple, will have overall management responsibility for the trial from a CTU perspective and will be responsible for the co-ordination of centres.

CTRC as part of the Liverpool Trials Collaborative has achieved full registration by the UK Clinical Research Collaboration (www.ukcrc.org) as their standards and systems were assessed by an international review panel as reaching the highest quality. The CTRC has a diverse trial portfolio underpinned by methodological rigour, a Good Clinical Practice (GCP) compliant data management system, and core standard operating procedures (SOPs).

For peer review only - http://bmjopen.bmj.com/site/about/guidelines.xhtml

**Sponsor:**	**Trial Management, Monitoring and Analysis:**
University of Liverpool<br>Liverpool<br>L69 3BX<br>Tel: 0151 794 8290<br>Email: sponsor@liverpool.ac.uk	Clinical Trials Research Centre, University of Liverpool<br>Institute of Child Health<br>Alder Hey Children's Hospital NHS Foundation Trust<br>Liverpool<br>L12 2AP<br>Tel: 0151 795 8757<br>Fax: 0151 795 8770<br>Email: bess@liverpool.ac.uk
**Mechanistic & Microbiology Work:**	**Paediatric Intensive Care Audit Data:**
University of Southampton<br>University Road<br>Southampton<br>SO17 1BJ<br>Tel: 023 8059 5000<br>Email: ris@soton.ac.uk	PICANet<br>School of Medicine<br>8.49 Worsley Building<br>University of Leeds<br>Leeds<br>LS2 9JT<br>PICANet<br>0113 343 8125<br>picanet@leeds.ac.uk

For peer review only

For peer review only - http://bmjopen.bmj.com/site/about/guidelines.xhtml

Contact Details: Individuals

**Individual Authorised to Sign the Protocol and Protocol Amendments on behalf of the Sponsor:**	**Chief Investigator (CI):**
Mr Alex Astor<br>Research Support Office<br>2nd Floor, Block D, Waterhouse Building, 3 Brownlow Street, Liverpool, L69 3GL<br>0151 794 8739<br>sponsor@liv.ac.uk	Prof. MG (Calum) Semple,<br>Women's and Children's Health<br>University of Liverpool<br>Institute in the Park, Alder Hey Children's Hospital, Eaton Road, Liverpool, L12 2AP, UK<br>0151 252 5250<br>m.g.semple@liverpool.ac.uk
**Medical Expert who will Advise on Protocol Related Clinical Queries (If other than CI):**	**Medical Expert who will Evaluate SAE Reports (if other than CI):**
Prof. Paul S McNamara<br>Women's and Children's Health<br>University of Liverpool<br>Institute in the Park, Alder Hey Children's Hospital, Eaton Road, Liverpool, L12 2AP, UK<br>0151 252 5250<br>mcnamp@liverpool.ac.uk	Prof. Paul S McNamara<br>Women's and Children's Health<br>University of Liverpool<br>Institute in the Park, Alder Hey Children's Hospital, Eaton Road, Liverpool, L12 2AP, UK<br>0151 252 5250<br>mcnamp@liverpool.ac.uk
**Medical Expert who will Evaluate SAE Reports (If other than CI or cross cover):**	
Dr Kent Thorburn<br>Paediatric Intensive Care Unit, Alder Hey Children's Hospital, Eaton Road, Liverpool, L12 2AP, UK<br>0151 228 4811 ext. 4180<br>kent.thorburn@alderhey.nhs.uk	

For peer review only - http://bmjopen.bmj.com/site/about/guidelines.xhtml

Additional Contacts:
The contact details of other individuals involved in the trial are detailed in documents supplementary to the protocol and stored in the Trial Master File:

Contact	Document Title
Trial Management Group (TMG)	BESS Trial Oversight Committee Membership
Trial Steering Committee (TSC)	
Independent Data and Safety Monitoring Committee (IDSMC)	
Principal Investigators	BESS Participating Centres

For peer review only

For peer review only - http://bmjopen.bmj.com/site/about/guidelines.xhtml

1 TABLE OF CONTENTS

Protocol Approval **2**
1 Table of Contents **7**
Glossary **12**
2 Protocol Summary **14**
3 Introduction **17**
3.1 Study Overview 17
3.2 Background 17
3.3 Rationale 18
3.4 Risk and Benefits 19
3.4.1 Potential Benefits 19
3.4.2 Potential Risks 19
3.5 Aims & Objectives of Work Package A 19
3.5.1 Aims 19
3.5.2 Primary Objective 20
3.5.3 Secondary Objective(s) 20
3.6 Outcome measures/endpoints 20
3.6.1 Primary Outcome/Endpoint 20
3.6.2 Secondary Outcomes/Endpoints 20
4 TRIAL DESIGN **24**
4.1 Trial Overview 24
Schematic of Trial Design: 25
4.2 Use of a standardised weaning and extubation protocol 26
5 Trial Setting and Selection of SITES & Clinicians **27**
5.1 Selection of Sites & Clinicians 27
6 Study Population **28**
6.1 Inclusion Criteria 28
6.1.1 Definition of bronchiolitis 28
6.2 Exclusion Criteria 28
6.3 Co-enrolment Guidelines 29
7 Recruitment and Randomisation **30**
7.1 Participant Identification and Screening 30
7.2 Informed Consent 30

For peer review only - http://bmjopen.bmj.com/site/about/guidelines.xhtml

7.2.1 Prospective Informed Consent Process ... 30
7.2.2 Consent for additional work ... 32
7.3 Randomisation Procedure ... 32
7.3.1 Randomisation System Failure ... 33
7.4 Blinding to Allocations ... 33
8 Participant Time Line, Assessments and Procedures ... 34
8.1 Administration of Intervention ... 34
8.1.1 Immediately prior to administration ... 34
8.1.2 Administration of allocated intervention ... 35
8.1.3 Post administration of allocated intervention ... 35
8.1.4 Weaning and extubation by protocol ... 35
8.2 Participant follow-up ... 36
8.3 Schedule for Intervention & Follow-up ... 37
8.4 Procedures for assessing Efficacy ... 39
8.4.1 Efficacy Assessment 1 ... 39
8.4.2 Efficacy Assessment 2 ... 39
8.5 Procedures for Assessing Safety ... 40
8.5.1 Procedure for Limited Physical Examination in BESS ... 40
8.6 Other Assessments ... 40
8.6.1 Parent Reported Quality of Life questionnaires ... 40
8.6.2 Special Assays or Procedures ... 41
8.7 Patient Transfer and Withdrawal ... 42
8.7.1 Patient Transfers ... 42
8.7.2 Withdrawal from the Trial ... 42
8.8 Loss to Follow-up ... 43
8.9 Trial Closure ... 43
9 Trial Treatment/interventions ... 44
9.1 Introduction ... 44
9.2 Arm A – Endotracheal Poractant Alfa at 200mg/kg (first dose), with up to two further doses at 100mg/kg ... 44
9.2.1 Formulation, Packaging, Labelling, Storage and Stability ... 44
9.2.2 Preparation, Dosage and Administration of Study Treatment/s ... 45
9.2.3 Specific Restrictions ... 46
9.2.4 Overdose ... 46
9.2.5 Temperature Compliance ... 46

For peer review only - http://bmjopen.bmj.com/site/about/guidelines.xhtml

9.2.6 Accountability Procedures for Trial Treatments 47
9.2.7 Assessment of Compliance with Study Treatment/s 48
9.3 Arm B – Placebo 48
9.4 Unblinding 48
9.4.1 Emergency Unblinding 49
9.4.2 Accidental unblinding 49
9.4.3 Unblinding at Trial Closure 49
9.5 Concomitant Medications 50
10 Safety Reporting 51
10.1 Terms and Definitions 51
10.2 Notes on Adverse Event Inclusions and Exclusions 52
10.2.1 Include: 52
10.2.2 Do Not Include: 52
10.2.3 Notification of deaths 53
10.3 Notes on Severity / Grading of Adverse Events 53
10.4 Relationship to Trial Treatment 54
10.5 Expectedness 54
10.6 Follow-up After Adverse Events 55
10.7 Time Period for Safety Reporting 55
10.8 Reporting Procedures 55
10.8.1 Flowchart for Reporting Requirements of Adverse Events 56
10.8.2 Non serious events (ARs/AEs) 58
10.8.3 Serious events (AEs/ARs/SUSARs) 58
10.9 Safety Reporting Responsibilities – Investigator 58
10.9.1 Maintenance of Blinding 59
10.10 Safety Reporting Responsibilities – CTRC 60
10.11 Safety reports 61
10.12 Urgent Safety Measures 61
10.13 Contact Details and Out-of-hours Medical Cover 61
11 Statistical Considerations 62
11.1 Introduction 62
11.2 Method of Randomisation 62
11.3 Sample Size calculation 62
11.4 Interim Monitoring and Analyses 62

For peer review only - http://bmjopen.bmj.com/site/about/guidelines.xhtml

11.5 Analysis Plan 63

12 exploratory & translational work; work packages b and c 64

12.1 Work Package B: Study of Parent & Staff Experiences 64

12.1.1 Parent and staff perspectives sub study analysis 65

12.2 Work Package C: 65

12.2.1 Metabolic profiling of surfactant composition and kinetics 66

12.2.2 Recovery of surfactant proteins A & D (SP-A & SP-D) is modulated by exogenous surfactant. 67

12.2.3 Lower airway inflammation is modulated by exogenous surfactant 67

12.2.4 Treatment efficacy or failure is associated with detection of specific pathogens. 67

12.2.5 Mechanistic Protocol 68

13 Regulatory and Ethical Approvals 70

13.1 Statement of Compliance 70

13.2 Regulatory Approval 70

13.3 Ethical Considerations 71

13.4 HRA Approval 71

13.5 Ethical and Local Governance Approval 71

13.6 Protocol Deviation and Serious Breaches 72

13.7 Study Discontinuation 72

14 Data Management and Trial Monitoring 73

14.1 Source Documents 73

14.2 Data Capture Methods 73

14.3 Monitoring 74

14.3.1 Risk Assessment 74

14.3.2 Central Monitoring 74

14.3.3 Clinical Site Monitoring 75

14.4 Confidentiality 75

14.5 Quality Assurance (QA) and Quality Control (QC) 75

14.6 Records Retention 76

15 Indemnity 77

16 Roles and Responsibilities 78

16.1 Role of Study Sponsor and Study Funder 78

16.2 Funding and Support in Kind 78

16.3 Protocol Contributors 79

16.4 Trial Committees 80

For peer review only - http://bmjopen.bmj.com/site/about/guidelines.xhtml

16.4.1 Trial Management Group (TMG) 80
16.4.2 Trial Steering Committee (TSC) 80
16.4.3 Independent Data and Safety Monitoring Committee (IDSMC) 80
17 Publication and Dissemination 81
17.1 Publication Policy 81
17.1.1 Authorship 81
17.2 Dissemination to Key Stakeholders 81
17.1 Data Sharing 82
18 Chronology of Protocol Amendments 83
18.1 Version 1.0 (18/07/2018) 83
18.2 Version 2.0 (28/09/2018) 83
19 References 84
20 Documents Supplementary to the protocol 87

For peer review only

For peer review only - http://bmjopen.bmj.com/site/about/guidelines.xhtml

Glossary

AE	Adverse Event
AR	Adverse Reaction
BAL	Bronchoalveolar lavage
CI	Chief Investigator
CRF	Case Report Form
CTIMP	Clinical Trials of an Investigational Medicinal Product
CTRC	Clinical Trials Research Centre
CTU	Clinical Trials Unit
EUDRACT	European Clinical Trials Database
DSUR	Development Safety Update Report
FiO_2	Fraction of inspired oxygen
GP	General Practitioner
HRA	Health Research Authority
IB	Investigator’s Brochure
IDSMC	Independent Data and Safety and Monitoring Committee
IMP	Investigational Medicinal Product
ISF	Investigator Site File
LRSQ	Liverpool Respiratory Symptom Questionnaire
MHRA	Medicines and Health Care Products Regulatory Agency
MV	Mechanical Ventilation
NIMP	Non Investigational Medicinal Product
NRES	National Research Ethics Service
NIHR CRN	National Institute for Health Research Clinical Research Network
OI	Oxygenation Index
OSI	Oxygen Saturation Index
PEEP	Positive End Expiratory Pressure
PI	Principal Investigator
PICANet	Paediatric Intensive Care Audit Network
PICU	Paediatric intensive care unit
PIP	Peak Inspiratory Pressure
PROM	Parent Reported Outcome Measure
QA	Quality Assurance
QC	Quality Control

For peer review only - http://bmjopen.bmj.com/site/about/guidelines.xhtml

R&D	Research & Development
REC	Research Ethics Committee
RN	Research Nurse (Registered)
RSI	Reference Safety Information
RSO	Research Support Office
SAE	Serious Adverse Event
SAR	Serious Adverse Reaction
SDV	Source Data Verification
SMPC	Summary of Product Characteristics
SOP	Standard Operating Procedure
SpO_2	Peripheral oxygen saturation
SSAR	Suspected Serious Adverse Reaction
SUSAR	Suspected Unexpected Serious Adverse Reaction
TMG	Trial Management Group
TMP	Trial Monitoring Plan
TSC	Trial Steering Committee
UAR	Unexpected Adverse Reaction
VI	Ventilation Index
WP-A	Work package A "The Trial"
WP-B	Work package B "Exploration of parental experience"
WP-C	Work package C "Mechanistic sub-studies"

For peer review only - http://bmjopen.bmj.com/site/about/guidelines.xhtml

2 PROTOCOL SUMMARY

**Full Title**	**The efficacy and mechanism of surfactant therapy for critically ill infants with bronchiolitis: The Bronchiolitis Endotracheal Surfactant Study (BESS). A phase-2 blinded randomised air-placebo-controlled trial of endotracheal surfactant (poractant alfa) in critically ill infants with bronchiolitis**
**Acronym**	**BESS**
**Phase**	2
**Target Condition**	Bronchiolitis of infancy requiring conventional invasive Mechanical Ventilation (MV)<br>Diagnosis of bronchiolitis per clinical criteria defined in national guidance NICE-NG9.
**Sample size**	284
**Main Inclusion Criteria**	1. Term-born infants < 26 weeks old and preterm-born infants < 26 weeks corrected age<br>2. Diagnosis of bronchiolitis<br>3. Requires conventional invasive MV via tracheal intubation<br>4. Parent or person with parental responsibility has given written informed consent for trial participation
**Main Exclusion Criteria**	1. Major congenital anomalies<br>2. Congenital neuromuscular disease<br>3. Already intubated for MV for >48 hours or likely to have been intubated for MV for >48 hours by randomisation<br>4. Have received or are receiving extracorporeal membrane oxygenation (ECMO) or oscillation during this episode of bronchiolitis<br>5. Have received or are receiving intratracheal administration of any surfactant during this episode of bronchiolitis<br>6. Receiving MV for primary apnoea rather than respiratory failure<br>7. A known hypersensitivity to the active substance or excipients of poractant alpha (Curosurf®)<br>8. A decision to wean to extubation has already been made<br>9. Clinical judgement of futility
**Study Centres and Distribution**	Approximately 14 Paediatric Intensive Care Units (PICUs) in UK tertiary hospitals.
**Patient Study Duration**	Duration of treatment: 24 hours (a maximum of three 12hourly intratracheal doses of surfactant; first dose (0hrs), second dose at 12hrs and third at 24hrs).

For peer review only - http://bmjopen.bmj.com/site/about/guidelines.xhtml

<table>
<tr><td colspan="2"></td><td colspan="2">Duration of follow-up: up to a maximum of 12 months, minimum 90 days.</td></tr>
<tr><td colspan="2">Overall Study duration</td><td colspan="2">44 months</td></tr>
<tr><td colspan="2">Agent/ Intervention</td><td colspan="2">Intervention: Endotracheal poractant alfa (Curosurf®, porcine lung phospholipid fraction supplied at 80mg/mL), first dose 200mg/kg, repeated 12 hourly at 100mg/kg, to a maximum of 3 doses according to the manufacturer's summary of product characteristics (SPC). Unlicensed use (UK licence, new indication).
Control: An identical series of up to three procedures (while intubated) using air as the placebo.</td></tr>
<tr><td colspan="2"></td><td>Objectives</td><td>Outcome Measures</td></tr>
<tr><td rowspan="2">Work Package A</td><td>Primary</td><td>To determine the efficacy of endotracheal surfactant in reducing total duration of MV by 18 hours or more, by random allocation of infants to receive either up to 3 doses of poractant alfa or up to 3 doses of air-placebo in an identical administration procedure (the Trial = Work package A)</td><td>The Primary Outcome measure for the Trial will be total duration of MV (hours) from randomisation to final extubation. We will include time off MV due to failed extubation.
Secondary Outcome measures relating to the Primary Objective of the trial are listed in the Protocol.</td></tr>
<tr><td>Secondary</td><td>To describe the impact of intervention on infants' long-term respiratory symptoms, using a parent reported outcome measure</td><td>The Liverpool Respiratory Symptom Questionnaire applied at 6m and 1yr after intervention in patients recruited in seasons 1 and 2.</td></tr>
<tr><td>Work Package B</td><td>Exploratory/ Translational:
Study of Staff & Parent Experiences</td><td>In a subgroup of patients, to explore staff and parent experiences of trial recruitment, consent and conduct in season 1 to inform season 2.</td><td rowspan="2">Outcome Measures relating to Exploratory and Translational aspects of the study are listed where known in section 12. Due to the exploratory nature of this work, other outcome measures are likely to be identified over this course of the study and after the trial has completed.</td></tr>
<tr><td>Work Package C</td><td>Exploratory/ Translational:
Mechanistic Study</td><td>In a subgroup of patients to explore the mechanisms for treatment efficacy and failures at a patient level by describing associations between trial outcome with markers of infection, inflammation, and surfactant composition in patient Bronchoalveolar Lavage Fluid.</td></tr>
</table>

NB: The Exploratory and Translational parts of the study (Work Packages B & C) will not inform the primary or secondary outcomes of the main trial. They are only included as an annexe to the

For peer review only

For peer review only - http://bmjopen.bmj.com/site/about/guidelines.xhtml

Trial Protocol for regulatory purposes related to the Research Ethic Committee, Health Research Authority and Local Institutional Research permissions.

For peer review only

For peer review only - http://bmjopen.bmj.com/site/about/guidelines.xhtml

3 INTRODUCTION

3.1 Study Overview

The study "The efficacy and mechanism of surfactant therapy for critically ill infants with bronchiolitis" will have the short title "Bronchiolitis Endotracheal Surfactant Study" and the acronym BESS.

The award supporting BESS supports three work packages. These are: the trial (Work package A; WP-A); a study of staff and parental experience (Work package B; WP-B) and mechanistic exploratory sub-studies (Work package C; WP-C).

Work package A is subject to Medicines for Human Use (Clinical Trials) Regulations 2004 (as amended) and requires regulatory permission from the Medicines and Healthcare products Regulatory Agency (MHRA). Work packages B and C are dependent upon trial activity but do not provide outcome measures for the trial and so are not subject to these regulations. The study, which includes all work packages, requires REC, HRA and NHS permissions for which this unified protocol document is required. The detail of Work Packages B and C are described in Section 12.

3.2 Background

Bronchiolitis of infancy is a seasonal viral disease of the lower airways that most commonly causes feeding difficulties and respiratory distress and in severe cases causes a significant public health burden due to hospitalisation and mortality(1). It is the single most common reason for hospital admission of infants (children age<1yr). The youngest of these and those born prematurely are most often and most severely affected (1).

A recent study of Hospital Episode Statistics for England found 25,000 cases of bronchiolitis from each annual birth cohort (40.4 per 1000 (95% CI 38.5 to 42.3)) were admitted to hospital in their first year of life (2). Using PICANet data, the same study found just over 1000 of these infants were admitted with critical illness to English paediatric intensive care units (PICUs) each year (95% CI 1.3 to 1.6 per 1000 infants/yr) for mechanical ventilation (MV). This accounted for 12% of all admissions to PICUs. The infants' modal age was 1 month; the mean duration of PICU admission was 6.1 days (95% CI 5.4 to 6.7 days), and their case-fatality rate was 1.75%.

Despite advances in the provision of non-invasive modes of respiratory support, admissions to PICU and duration of MV for life-threatening bronchiolitis have remained static for the past 4 years (2). Excluding infants with rare congenital co-morbidities (mostly cardiac & neuromuscular anomalies), infants requiring MV for bronchiolitis do so for a mean of 4.8 days (SD 2.5d) (PICANet data, 2011-14).

Existing Research

In 2015 Jat & Chawla (3) found on pooled analysis of three small studies a significant reduction in length of PICU stay between endotracheal surfactant treated and control groups (mean difference (MD) -1.81d (95%CI -2.42 to -1.19) of critically ill infants with bronchiolitis who required MV. There were sustained improvements of arterial oxygenation (PaO_2) and carbon dioxide ($PaCO_2$) elimination (4-6). No adverse effects or complications were observed in the three studies. The authors of this systematic review and meta-analysis concluded that use of surfactant had favourable effects but that larger trials with adequate power were required to evaluate the effectiveness of surfactant therapy in critically ill infants with bronchiolitis who required MV.

For peer review only - http://bmjopen.bmj.com/site/about/guidelines.xhtml

3.3 Rationale

There is no vaccine or specific treatment for bronchiolitis. Care is supportive as described in National Guidance (17). Breathing fails in very severe cases, and these babies need intensive care with breathing being supported by a machine called a ventilator, hence known as mechanical ventilation (MV).

Pulmonary surfactant is secreted by lung alveolar cells to reduce surface tension so increasing compliance of the lungs, allowing them to inflate more easily and reduce the work of breathing (8). Surfactant proteins A and D have innate antiviral immune properties as collectins (9).

Studies of infants with life-threatening bronchiolitis show reduced lung compliance and surfactant deficiency (10-14). These features are similar to the respiratory distress syndrome of infants born prematurely. Premature infants are routinely treated with animal-derived surfactant to improve lung compliance, and reduce mortality, morbidity and duration of MV (15).

Most infants with severe bronchiolitis are very young and/or born prematurely. They present at modal age 1 month, with 62.2% age <2months (2). We hypothesise that the effect of exogenous surfactant on infants with severe bronchiolitis may be similar to the therapeutic effect on those born prematurely as both conditions present with surfactant deficiency. At present surfactant is used without evidence as rescue therapy in some infants and young children with the most severe respiratory failure who are difficult to manage even with MV.

The systematic reviews by Jat & Chawla found that surfactant therapy in infants with critical illness due to bronchiolitis was: safe, improved gas exchange and reduced both duration of MV and length of stay on PICU (3, 16).

There are no other relevant trials registered on Clinicaltrials.gov, EU Clinical Trial Register or ISRCTN [search “surfactant” AND “bronchiolitis”, 6-JUL-2018].

This topic has been identified by the National Institute for Health Research (NIHR) Maternal Neonatal and Child Health panel in 2013 and selected by the Prioritisation Group for commissioning by both the Health Technology Assessment (HTA) and Efficacy and Mechanism Evaluation (EME), and has the support of the NIHR Translational Research Partnership (TRP) for Inflammatory Respiratory Diseases and UK Paediatric Intensive Care Society Study Group (PICS-SG).

Research Question

Does surfactant therapy reduce duration of invasive mechanical ventilation when given to infants early in the course of critical illness due to bronchiolitis?

Intervention

The intervention being trialled is:

Endotracheal poractant alfa (Curosurf®, porcine lung phospholipid fraction supplied at 80mg/mL), first dose 200mg/kg, repeated 12 hourly at 100mg/kg, up to a total of 3 doses according to the manufacturer's summary of product characteristics (SPC).

Or-

An identical series of up to three procedures (while intubated) using air as the placebo.

Hypothesis

The hypothesis we will test in the trial is “Endotracheal surfactant reduces duration of mechanical ventilation by 18 hours

For peer review only - http://bmjopen.bmj.com/site/about/guidelines.xhtml

3.4 Risk and Benefits

3.4.1 Potential Benefits

The potential benefit of the therapy to the participants is a sustained improvement in gas exchange leading to a reduction of total duration of MV. The potential benefit to the participant is a reduction of time in a technology-dependent vulnerable state, reduced risk of MV associated morbidity, reduced exposure to sedation and muscle paralysing drugs and a more rapid return to usual parental care. This in turn would reduce parental anxiety. Also, freeing up PICU beds during winter surge offers savings and opportunity to use resources for other conditions.

3.4.2 Potential Risks

Administration of poractant alfa (porcine derived surfactant, Curosurf®) in infants with bronchiolitis is well tolerated and considered safe(3). Transient minor adverse reactions associated with administration of poractant alfa for the treatment of neonatal Respiratory Distress Syndrome (RDS) include bradycardia, hypotension, endotracheal tube blockage, and oxygen desaturation. The risk of such events is minimised by tracheal toilet prior to administration of poractant alfa, as recommended in the Summary of Product Characteristics.

Small volume (2x1mL/kg) non-bronchoscopic bronchoalveolar lavage (BAL) procedure on PICU is safe and well tolerated procedure(7). In a published audit of 27 children on PICU who underwent multiple small volume BAL installations (5x1mL/kg) as part of a single routine diagnostic procedure, 7 experienced transient oxygen desaturation requiring temporary increased FiO_2. We will limit BAL sampling to a 2x1mL/kg instillation at each scheduled assessment.

Small volume (1.25mL (range 1.0 to 1.3mL)) blood sampling will be done on infants ventilated at Liverpool and Southampton PICUs and scheduled to coincide with routine blood sampling. Existing vascular access will be used when available. There is no risk of anaemia associated with this limited volume of blood sampling. The sample will be taken from an arterial line if present. If not by standard venesection.

Capillary blood gas sampling (100microliters) or arterial blood gas sampling for subject monitoring will use data from routine blood gas sampling when either is collected within a 2-hour window of a research-scheduled sample. As this is part of standard care no additional risks are expected.

3.5 Aims & Objectives of Work Package A

3.5.1 Aims

1. To conduct a multicentre blinded randomised placebo-controlled phase-2 trial of the endotracheal surfactant poractant alfa (CUROSURF®) as therapy for bronchiolitis in critically ill infants.
   Hypothesis to test: Endotracheal surfactant reduces duration of mechanical ventilation by 18 hours (Work package A (WP-A)).
2. To evaluate the safety profile in the two treatment groups

For peer review only - http://bmjopen.bmj.com/site/about/guidelines.xhtml

3.5.2 Primary Objective

To determine the efficacy of endotracheal surfactant in reducing total duration of MV by 18 hours, by random allocation of infants to receive up to 3 doses of poractant alfa or up to 3 doses of air-placebo in an identical administration procedure.

3.5.3 Secondary Objective(s)

1. To describe the impact of intervention on infants' long-term respiratory symptoms
2. To describe secondary outcomes of efficacy including physiological indices and duration of other modes of respiratory support.
3. To assess the safety of the intervention for infants with life-threatening bronchiolitis

3.6 Outcome measures/endpoints

3.6.1 Primary Outcome/Endpoint

The primary outcome measure will be total duration of MV (hours) from randomisation to final extubation. We will include time off MV due to failed extubation.
Justification: This outcome measure is clinically important, was suggested in both HTA and EME commissioning calls and is valued by our Parent Advisory Group. Reduced duration of MV reduces ventilator-associated morbidity, sedation, muscle relaxants, healthcare costs and bed occupancy at a time of increased seasonal pressure.

3.6.2 Secondary Outcomes/Endpoints

The secondary outcome measures for the trial (WP-A) are:
For efficacy:

1. duration of post-extubation non-invasive respiratory support (all forms of non-invasive ventilation: e.g. nasal CPAP and nasal high low oxygen)
2. duration of oxygen supplementation
3. number of trial interventions given (both poractant and placebo)
4. use of steroids to assist extubation
5. duration of stay on PICU and in hospital;
6. change over time from baseline of Ventilation Index (VI), Oxygenation Index (OI) and Oxygenation Saturation Index (OSI) while mechanically ventilated, & SpO_2/FiO_2 (SF) ratio.[1]
7. the score (value) of parent reported outcome measure of respiratory symptoms the "Liverpool Respiratory Symptom Questionnaire" (LRSQ) at 6m (+/-1m) and 12m (+/-1m) (20);

[1] VI and OI are validated physiological measurements reflecting respiratory function (gas exchange) normalised for level of respiratory support provided by MV, but require blood sampling(21). OSI is a validated physiological measurement reflecting respiratory function (gas exchange) normalised for level of respiratory support provided by MV that does not require blood sampling (41). The SF ratio is a non-invasive measure of respiratory function and can be calculated in situations where invasive blood gas monitoring and MV has ceased (22).

For peer review only - http://bmjopen.bmj.com/site/about/guidelines.xhtml

8. time from randomisation to meeting study criteria for readiness for Spontaneous Breathing Test

For safety:

1. failure to administer the intervention due to any adverse event during preparatory processes (tracheal toilet or BAL)
2. failure to complete administration of the intervention due to any adverse event during administration of the intervention (regardless of which arm of allocation)
3. incidents of ‘air leak’ (pneumothorax and pneumomediastinum) occurring before discharge from PICU
4. other adverse events and serious adverse events associated with the intervention
5. any need to replace the endotracheal tube
6. parent reported readmission to hospital (all causes) up to 90 days post randomisation
7. death during PICU admission
8. all-cause mortality at 90 days post randomisation

For peer review only

For peer review only - http://bmjopen.bmj.com/site/about/guidelines.xhtml

Table 1: Objectives and Outcome Measures

Objectives	Outcome Measures	Timepoint(s) of evaluation of this outcome measure (if applicable)
**Primary Objective** To determine the efficacy of endotracheal surfactant in reducing total duration of MV by 18 hours or more, by random blind allocation of infants to receive either up to 3 doses of poractant alfa or up to 3 doses of air-placebo in an identical administration procedure.	Total duration of MV (hours) from randomisation to final extubation, including any time off MV due to failed extubation.	N/A
**Secondary Objective 1** To describe the impact of intervention on infants' long term respiratory symptoms	The score (value) of patient reported outcome measure of respiratory systems in the "Liverpool Respiratory Symptom Questionnaire" (LRSQ)	LRSQ at 6 months and 12 months post randomisation
**Secondary Objective 2** To describe secondary outcomes of efficacy including physiological indices and duration of other modes of respiratory support	Time from randomisation to meeting study criteria for readiness for Spontaneous Breathing Test	N/A
	Number of trial interventions given	N/A
	Change over time from baseline of Ventilation Index (VI), Oxygenation Index (OI) and Oxygenation Saturation Index (OSI) while mechanically ventilated, & SpO_2/FiO_2 (SF) ratio	Capillary blood gas sampling immediately prior to intervention and then at 6, 12, 24, 36 and 48hrs while still on MV; to continue daily after 48hrs if still on MV.
	Duration of oxygen supplementation	N/A
	Use of steroids to assist extubation	N/A

For peer review only - http://bmjopen.bmj.com/site/about/guidelines.xhtml

	Duration of post-extubation non-invasive respiratory support	N/A
	Duration of stay on PICU and in hospital	N/A
**Secondary Objective 3**<br>To assess the safety of the intervention for infants with life-threatening bronchiolitis	Failure to administer the intervention due to any adverse event during preparatory processes (tracheal toilet or BAL)	Randomisation to 24 hours after final trial intervention
	Failure to complete administration of the intervention due to any adverse event during administration of the intervention (regardless of which arm of allocation)	Randomisation to 24 hours after final trial intervention
	Incidents of 'air leak' (pneumothorax and pneumomediastinum) occurring before discharge from PICU	Randomisation to discharge from PICU
	Other adverse events and serious adverse events associated with the intervention	Randomisation to 24 hours after final trial intervention
	Any need to replace the endotracheal tube	Randomisation to successful extubation
	Parent reported readmission to hospital (all causes) up to 90 days post randomisation	PICU discharge to 90 days post-randomisation
	Death during PICU admission	Randomisation to PICU discharge
	All-cause mortality at 90 days post randomisation	PICU discharge to 90 days post-randomisation

For peer review only - http://bmjopen.bmj.com/site/about/guidelines.xhtml

4 TRIAL DESIGN

4.1 Trial Overview

The BESS trial is a phase-2 blinded randomised air-placebo-controlled trial of endotracheal surfactant (poractant alfa) in critically ill infants with bronchiolitis (we will refer to this as Work Package A, or WP-A).

For peer review only

For peer review only - http://bmjopen.bmj.com/site/about/guidelines.xhtml

Schematic of Trial Design:

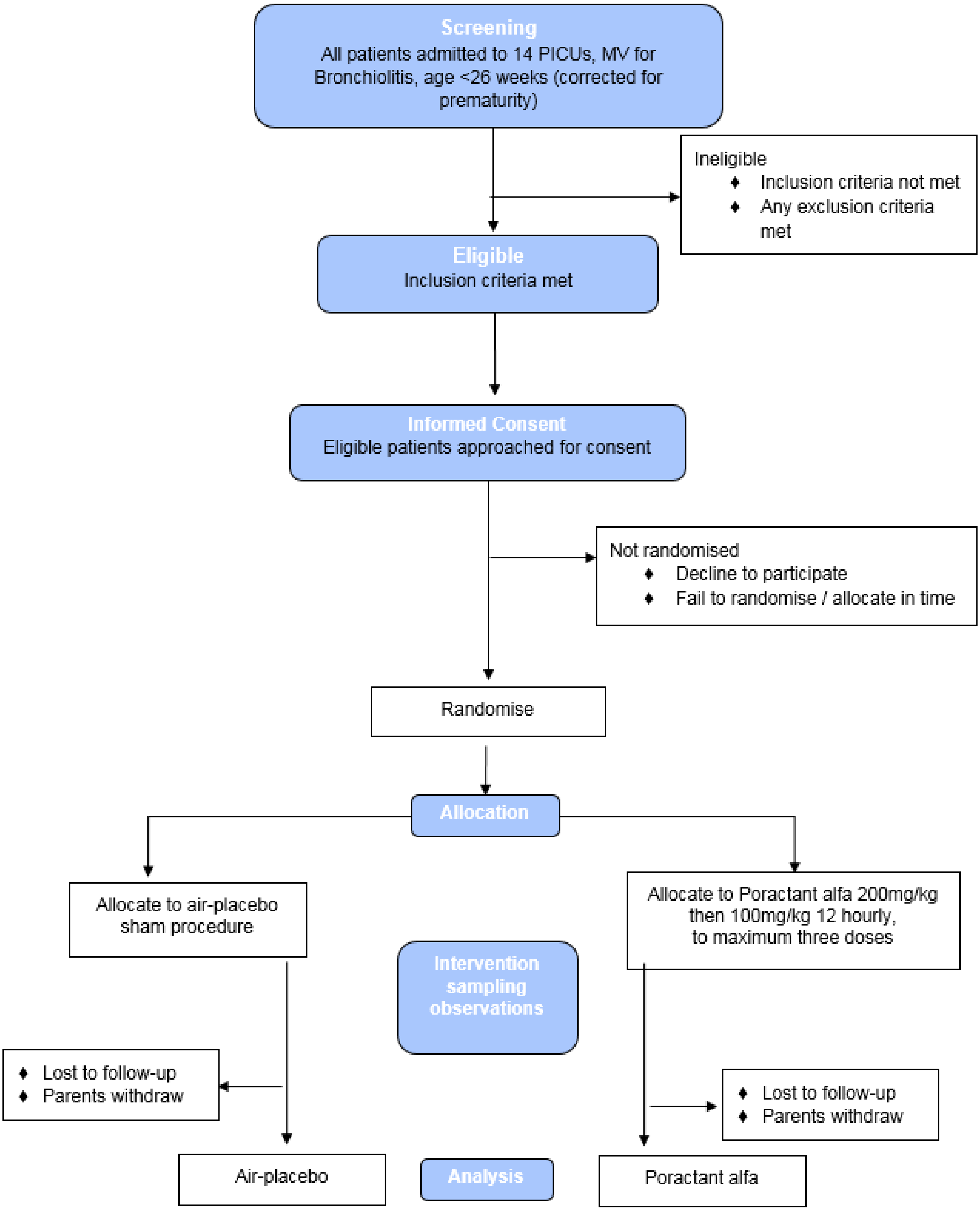

Figure 1: Schematic of Trial Design

For peer review only - http://bmjopen.bmj.com/site/about/guidelines.xhtml

4.2 Use of a standardised weaning and extubation protocol

On the direction of the NIHR EME board, the BESS trial protocol will include a standardised protocol for weaning from mechanical ventilation and extubation.

Weaning and extubation by protocol will standardise this aspect of care across sites and in both arms of BESS. Infants in BESS will be extubated after they meet specified physiological criteria that indicate the infant is ready for a Spontaneous Breathing Test (SBT) and pass that test, (see section 8.1.2.1 for further details).

For peer review only

For peer review only - http://bmjopen.bmj.com/site/about/guidelines.xhtml

5 TRIAL SETTING AND SELECTION OF SITES & CLINICIANS

Approximately fourteen Paediatric Intensive Care Units (PICUs) in the United Kingdom that routinely mechanically ventilate critically ill infants with high caseloads of bronchiolitis will recruit to BESS. PIs for sites that have provisionally agreed to participate in BESS have shared local activity data, commented on the application and are recognised as co-applicants on the application award. The PIs will champion the study at the sites and take local responsibility for the conduct of the trial. PIs will identify local co-Investigators.

5.1 Selection of Sites & Clinicians

Criteria for the selection of centres includes

- NHS Hospitals in the UK providing paediatric intensive care
- Historic high case load of bronchiolitis, as reported to PICANet
- Sufficient research capacity comprised of staff, time and facilities to undertake the trial; including patient screening and recruitment, randomisation, collection and provision to the CTRC of all required data, collection of research samples, identification and management of adverse events including notification to CTRC within protocol defined timeframes, identification and provision of information to the CTRC of all protocol breaches.
- All staff contributing to the trial must have valid certified Good Clinical Practice (GCP) training throughout the conduct of the trial. Requirements of the Sponsor are that renewal of GCP must occur every 3 years.
- Willingness to participate

Final decisions on site selection will be determined by the Trial Management Group (TMG) and will be described in the supplementary document 'BESS Site Suitability Assessment.
Initiation of centres will be undertaken in compliance with CTRC SOPs[2]. Centres fulfilling the criteria will be selected to be recruitment centres for the BESS trial and will be opened to recruitment upon successful completion of all global (e.g. MREC and MHRA) and study-specific conditions (e.g. site personnel training requirements) and once all necessary documents have been returned to CTRC as detailed in the trial Green Light checklist.

Participating centres will be listed in the 'BESS Participating Centres' log, maintained separately to the protocol and stored in the Trial Master File (TMF).

[2] CTRC SOP TM017 Study Initiation in CTRC and TM018 Study Initiation at Sites

For peer review only - http://bmjopen.bmj.com/site/about/guidelines.xhtml

6 STUDY POPULATION

6.1 Inclusion Criteria

1. Term-born infants < 26 weeks old and preterm-born infants < 26 weeks corrected age†
2. Diagnosis of bronchiolitis (see below)
3. Requires conventional invasive MV via tracheal intubation
4. Parent or person with parental responsibility has given written informed consent for trial participation

Note: Infants with chronic lung disease of prematurity can be included in the trial.
† Correcting for prematurity is a well-established method for adjusting assessment of infants born prematurely who are anatomically, physiologically, immunologically and neuro-developmentally immature for their chronological age. In our study, term-born infants will be eligible for inclusion in the study up to chronological age of 26 weeks old (i.e. all those under 6 months old). Premature born infants have their age corrected to account for weeks of lost gestation, hence an infant born 12 weeks premature (i.e. before completing the 28th week of the 40 weeks of pregnancy) would still be eligible up to a chronological age of 38 weeks old (i.e. under 9 months), because that infant is still less than 26 weeks old when age is corrected for the premature birth.

6.1.1 Definition of bronchiolitis

Diagnosis of bronchiolitis will be made using clinical criteria defined in national guidance NICE-NG9 (17).

6.2 Exclusion Criteria

1. Major congenital anomalies
2. Congenital neuromuscular disease
3. Already intubated for MV for >48 hours or likely to have been intubated for MV for >48 hours by randomisation
4. Have received or are receiving extracorporeal membrane oxygenation (ECMO) or oscillation during this episode of bronchiolitis
5. Have received or are receiving intratracheal administration of any surfactant during this episode of bronchiolitis
6. Receiving MV for primary apnoea rather than respiratory failure
7. A known hypersensitivity to the active substance or excipients of poractant alpha (Curosurf®)
8. A decision to wean to extubation has already been made
9. Clinical judgement of futility

For peer review only - http://bmjopen.bmj.com/site/about/guidelines.xhtml

6.3 Co-enrolment Guidelines

The investigators are happy to enter in to co-enrolment agreements where feasible.

Where other trials are taking place at a site participating in BESS and the target population overlaps, sites should make the BESS team aware at the earliest opportunity so that the potential for co-enrolment can be discussed between the two trial teams. The BESS investigators are open to co-enrolment with other studies but this must be agreed between both Chief Investigators prior to randomisation into BESS.

For peer review only

For peer review only - http://bmjopen.bmj.com/site/about/guidelines.xhtml

7 RECRUITMENT AND RANDOMISATION

7.1 Participant Identification and Screening

All patients admitted to recruiting PICUs, receiving conventional MV for bronchiolitis, age <26 weeks (corrected for prematurity) will be screened for eligibility before trial entry. Screening and confirmation of full eligibility will be completed to allow randomisation within 48 hours of intubation.

A screening log of patients who are assessed for eligibility but not randomised will be maintained and returned to the CTRC on a regular basis, as this will provide important information for monitoring purposes. The screening log will capture which patients have been screened for the trial but assessed as ineligible, and those considered eligible and approached for consent but for whom consent was not obtained; reasons (all per subject) for non-inclusion will be recorded. Reasons for declining to participate will be asked routinely but it will be made clear that parents/persons with parental responsibility do not have to provide a reason unless happy to do so.

The screening log will not contain patient identifiers as those who are not eligible will not have provided their consent for identifiers to be captured as part of the trial. Therefore, sites will maintain their own trial register linking patients screening and/or randomisation numbers to their identifiers. Under no circumstances will this log be returned to the CTRC.
Patients will be reviewed for eligibility once they have been intubated for MV, or as soon as their arrival into PICU if they are already intubated. Parents/persons with parental responsibility for patients who meet the eligibility criteria and are suitable for inclusion (see 6.1 and 6.2) will be provided with information about the trial (both verbal and written) to consider participation.

Only a medically qualified doctor authorised on the site Delegation Log can confirm full eligibility of any patient. A record of this confirmation must be made in the patient's medical notes on the date of confirmation of eligibility.

7.2 Informed Consent

7.2.1 Prospective Informed Consent Process

Parents and those persons with parental responsibility may give consent for the patient to take part in BESS; hereafter throughout this protocol they shall be referred to as 'parent(s)'.
Initiation of the intervention in BESS is urgent and the condition "critical illness due to bronchiolitis requiring MV" is a medical emergency. Randomisation must be completed within 48 hours of intubation for MV, which will limit the time available for a parent to consider whether or not their child should participate. Site research teams will use their understanding of the clinical situation for each individual patient to judge when is most appropriate to approach parents about trial participation. The trial concept will be introduced to a parent by familiar members of the usual clinical care team, be they nurses or physicians, as per the Canadian Critical Care Trials PICU Research Group trial recruitment model (24).
If a parent is interested, a member of the usual clinical care team will make an introduction to the research staff who will provide written study information and offer further verbal information.

For peer review only - http://bmjopen.bmj.com/site/about/guidelines.xhtml

Due to the time-critical nature of the intervention, the parent will have a limited timeframe to ask questions and make a decision about their child's participation in the trial. Randomisation must take place within 48 hours of intubation for MV and while some parents may have this full timeframe to consider trial participation, others may have considerably less due to time of arrival on PICU, time of approach etc. Consent will be sought by any appropriately trained and delegated medical staff and where local site policy allows, research nurses or respiratory physiotherapists (the research team).

Consent from the parent must be obtained prior to every infant participating in the trial. This consent will be sought after a full explanation has been given of the treatment options; including the conventional and generally accepted methods of treatment, the objectives, risks and inconveniences of the trial, and the conditions under which it is to be conducted.

Parent Information Sheet and Consent (PISC) forms, describing in detail the trial intervention, procedures and risks will be approved by a research ethics committee (REC). The parent will be asked to read and review this document. Upon reviewing the document, the person seeking consent will explain the research study to the parent. This information, and the supporting conversation, will emphasise that participation in the trial is voluntary and that the parent may withdraw their infant from the trial at any time and for any reason.

The parent will be given opportunity to ask any questions that may arise and time to consider the information, within the constraints of the 48 hour period between intubation and randomisation, prior to agreeing to participate. A contact point where further information about the trial may be obtained will be provided on the parent information sheet.

The parent will then sign and date the consent form if they agree to their infant participating. Both the person obtaining consent and the parent must sign and date the consent form; the researcher and parent must sign at the same time, with the researcher completing their signature **after** the parent.

A copy of the signed PISC will be given to the parent for their record. The original copy will be filed in the Investigator Site File, a copy filed in the participant's medical notes and a further copy should be sent to the CTRC. If the site has moved to an electronic health record (EHR), a scanned copy should be entered in the patient notes. A record of the consent process should also be recorded in the participant's medical notes.

The right of the parent to refuse consent for the infant to participate in the trial without giving reasons must be respected. After the infant has entered the trial, the clinician will remain free to give alternative treatment to that specified in the protocol, at any stage, if s/he feels it to be in the best interest of the patient. However, the reason for doing so should be recorded and the infant will remain within the trial for the purpose of follow-up and data analysis.

Similarly, the parent of the infant remains free to withdraw the infant at any time from the protocol treatment and trial follow-up without giving reasons and without prejudicing the further treatment of the infant (see section 8.6.2 for further details). They do not need to provide a reason for withdrawing their infant from the trial, which will be emphasised in the PISC. Any parent who withdraws their infant from the trial will be approached for participation in the sub-study of parental experience (WP-B), unless they previously declined sub-study participation at consent (see Section 12).

For peer review only - http://bmjopen.bmj.com/site/about/guidelines.xhtml

7.2.2 Consent for additional work

Parents of eligible infants who have been approached to give consent for the BESS trial will also be offered the opportunity to take part in a telephone interview about their experiences of the consent process. This will be offered regardless of whether or not consent was given for the infant to participate in BESS (see section12.1).

An alternative PISC will be offered to parents at Liverpool and Southampton PICUs (sites participating in the Mechanistic work exploring surfactant metabolism; WP-C. See section 12.2).

A decision to decline to participate in either WP-B or WP-C will not impact upon care, or preclude involvement in the trial (WP-A).

7.3 Randomisation Procedure

Participants will be randomised in a 1:1 ratio, stratified by site and duration of ventilation prior to randomisation (<24hrs, ≥24hrs).

Randomisation must be completed within 48 hours of intubation.

Participants will be randomised using a secure 24-hour web-based randomisation and drug supply programme, managed by a third party subcontracted by Chiesi Farmaceutici S.p.A. (Chiesi). A personal login username and password will be required to access the randomisation system. Designated research staff will be issued with a unique personal login and password upon completion of training in the use of the system.

When the system requirements (confirmation of consent and eligibility) are confirmed the participant treatment allocation and a unique study number (randomisation number) will be displayed on a secure webpage. The randomising staff member must select the partially completed unblinding envelope corresponding to the participant's randomisation number (as clearly displayed on the front of the envelope) and record the allocation on the printed slip inside. The unblinding envelope must then be sealed and transferred to an agreed secure location accessible to PICU staff.

Once a randomisation number has been generated, the randomising staff member will be able to enter the resupply module within the system, where they will select the participant weight category and dose number (i.e. first, second or third) for the system to allocate the required number of vials for the participant.

Sites must complete this step for participants in both arms of the trial and for every given dose (first, second or third) regardless of allocation to drug or placebo.

The randomisation and resupply system is configured to allow remote monitoring of stock levels at site by the distributor contracted by Chiesi, and further IMP stocks will be supplied once the amount available at site reaches an agreed trigger level. However, it remains the responsibility of the PI or delegated research staff to check the supplies available on the PICU prior to randomisation to ensure there is sufficient supply of the IMP, as the majority of stock will be held at the local site Pharmacy (see Section 9.2.6 for further information regarding intervention accountability).

For peer review only - http://bmjopen.bmj.com/site/about/guidelines.xhtml

Unblinded and appropriately delegated site staff will be able to log into the randomisation and resupply system at any time and view the allocations of each participant to ensure continuity between shifts.

7.3.1 Randomisation System Failure

In the event of a randomisation system failure, the centre should contact the coordinating team at the CTRC (Monday to Friday between 9:00 to 17:00 excluding bank holidays) to inform them of the problem. Out-of-hours the centre should contact the Chief Investigator or his delegates, on 07506 653 560.

7.4 Blinding to Allocations

BESS will use an established strategy of a sham-procedure to blind as many research and usual care staff as possible to the nature of the intervention. This sham procedure was used in the development of surfactant as a therapy for surfactant deficiency of prematurity (6,19).

The participants, CTRC staff (excluding statistical team members as appropriate), and all members of the site research teams (excluding agreed randomising staff member, the administrator of the intervention and the dosage witness) will be blinded to treatment allocations. Occasional transient adverse effects associated with surfactant administration and back-tracking of surfactant up the endotracheal tube, plus the more frequently observed positive effects of rapid improvement in gas exchange immediately following surfactant administration, may result in some blinded care givers being able to infer that a participant has been allocated to the surfactant arm of the trial.

The randomising person, person drawing up the surfactant or air placebo and a second person checking the dose will be unblinded. Due to the transient effects and back-tracking it is likely that the Usual Care Nurse (UCN) will become unblinded, and we therefore recommend that if possible the UCN draws up the surfactant or acts as the witness to the drawing up, to minimise the number of people unblinded. Please see figure 2 for an intervention flowchart showing which staff/steps are blinded and unblinded.

Staff who are unblinded due to their involvement or believe themselves to have become unblinded for any reason are strongly discouraged from discussing this with other members of the team, unless the effect noted is causing an adverse event that requires action. Any staff member who believes themselves to have become unblinded must excuse themselves from any decision making regarding the weaning and extubation of the infant. The need to maintain blinding will be emphasised during all Site Training Visits.

For peer review only - http://bmjopen.bmj.com/site/about/guidelines.xhtml

8 PARTICIPANT TIME LINE, ASSESSMENTS AND PROCEDURES

8.1 Administration of Intervention

The first trial intervention must be administered within 2 hours of randomisation with subsequent doses (if required) at 12 and 24 hours post randomisation. A grace period of one hour either side of the anticipated administration time is permitted.

- Randomisation; **T0**
- First intervention must be given within two hours; between **T0** and **T2**
- Second intervention should be given 12 hours after randomisation; at **T12** (+/- 1 hour)
- Third intervention should be given 24 hours after randomisation; at **T24** (+/- 1 hour)

8.1.1 Immediately prior to administration

A limited physical examination, with minimal handling, must be undertaken in the two hours prior to each administration of the trial intervention (as per Section 8.5.1).

Prior to intervention and in a drug room away from usual staff, the study respiratory physiotherapist, research nurse or other delegated, unblinded staff member will:

- Draw up a dose of poractant alfa or an equal volume of air as placebo according to the random allocation into a syringe
- Ensure a second person is present to witness this process and check the dose volume
- Cover the syringe with an opaque paper shield to maintain blinding of other staff and parents
- Take the syringe to the bedside, along with syringes of sterile 0.9% sodium chloride for tracheal toilet and the BAL sampling set.

Infants in both the treatment and placebo arms of the trial will have 0.9% sodium chloride “normal saline” tracheal toilet and 0.9% sodium chloride BAL immediately prior to administration of the intervention. Both can cause immediate transient desaturation, need for increased sedation but also and more often rapid improvement in gas exchange when obstructing secretions are removed. Tracheal toilet and BAL can also lead to creamy white secretions appearing in the endotracheal tube, and this will be observed in both treatment and placebo groups.

Staff should remind parents, if present, of the purpose of the imminent study procedure and give them the opportunity to remain or leave as they choose. The usual care nurse will remain to care for the participant and assist as required.

Tracheal toilet is usually required for babies with bronchiolitis and will be done as recommended in the SPC before administration of poractant alfa. BAL sampling with 0.9% sodium chloride (2x1mL/kg) will be done if scheduled (section 8.2). Both tracheal toilet and BAL sampling is done by catheter access to the trachea; either via the endotracheal suction port without disconnecting the

For peer review only - http://bmjopen.bmj.com/site/about/guidelines.xhtml

infant from the ventilator or by disconnecting the infant from the ventilator and performing hand bagging while a side-port with one-way valve are utilised for the toilet and BAL.

The use of muscle relaxant prior to tracheal toilet, BAL and or administration of the intervention is at clinical discretion.

8.1.2 Administration of allocated intervention

The intervention will be given per either method described in the SPC; either keeping the infant connected to the ventilator or momentarily disconnecting and performing hand-bagging (at the attending Respiratory Physiotherapist's decision based on patient status).

- Connect the syringe containing the surfactant or air-placebo to a suitable catheter (size 5F);
- Pass the catheter through the suction or side port into the endotracheal tube and advance the catheter, aiming to have the tip of the catheter placed just beyond the tip of the endotracheal tube
- Deliver the intervention (surfactant or air-placebo) slowly as a single bolus (recommended over 0.5-3 minutes) directly into the lower trachea
- The intervention (surfactant or air-placebo) will disperse through mechanical ventilation cycles into the peripheral lung tissue where the excipient (sterile buffered 0.9% sodium chloride) is absorbed in the alveoli and the surfactant phospholipid is deposited.

8.1.3 Post administration of allocated intervention

All participants must be given a limited physical examination, with minimal handling, in the interval between one hour and two hours after the administration of each intervention dose (see section 8.5.1).

8.1.4 Weaning and extubation by protocol

The unblinded staff involved in the administration of the BESS intervention (study respiratory physiotherapist or nurse practitioner or other qualified person and one usual care PICU nurse) and parents who may be observing, will not be involved in decisions to wean respiratory support provided by mechanical ventilation and will not be involved in decisions to extubate.

We will wean respiratory support provided by MV and extubate according to a standardised protocol that will form part of the site-specific training packages.

Readiness for a spontaneous breathing test (SBT) will be identified by the patient meeting specified physiological targets of gas exchange at a given level of ventilation and/or the treating clinician believes the child to be ready for SBT.

The time at which a child is deemed ready for SBT **must** be recorded in their notes/electronic health record. The time that a child is extubated **must** be recorded in their notes/electronic health record.

For peer review only - http://bmjopen.bmj.com/site/about/guidelines.xhtml

8.2 Participant follow-up

Clinical follow-up (daily data collection) will take place for the period of time between randomisation and the discharge from hospital of the participant.

All non-serious adverse events will be collected until 24 hours after the participant receives their final trial intervention; please note that this may not be their third dose if less than 3 doses are required before the participant meets the criteria to be ready for weaning (see section 10 for full safety reporting information).

Non-serious adverse events occurring after 24 hours post-final intervention but before 90 days post-randomisation/discharge home/death should be reported if the local Investigator believes that they may be related to the intervention.

Although active monitoring for adverse events and adverse reactions will cease at 24 hours post final intervention and 90 days post randomisation / discharge home / death respectively, any which the site research team become aware of after these time-points which are assessed as serious (i.e. Serious Adverse Events or Serious Adverse Reactions) should be reported to the CTRC.

Routinely collected audit data regarding participant care will be collected from PICANet until 90 days post randomisation.

Parent reported outcomes will be collected via the LRSQ at 6 and 12 months post randomisation for participants recruited in seasons 1 and 2, and at 6 months only for participants recruited in season 3.

The end of follow-up definition for each participant will therefore be 12 months post randomisation for participants in season 1 and 2, or 6 months post randomisation for participants in season 3.

For peer review only - http://bmjopen.bmj.com/site/about/guidelines.xhtml

8.3 Schedule for Intervention & Follow-up

Table 2: Schedule for intervention and follow-up

Procedures	Staff	Consent & randomisation	prior to first intervention	1-2hr,post first intervention	6hr	12hr, prior to second intervention	1-2hr post second intervention	24hr, prior to third intervention	1-2hr post third intervention	36hr	48hr	Daily if MV	90d	6m	12m
Prescribe	PI/Dr	X													
Invitation to participate in WP-B (parental experience sub-study)	PI/Dr/RN	X*													
+Invitation to participate in WP-C (surfactant metabolism sub-study)	PI/Dr/RN	X*													
ˣBlood Gas sample (capillary or arterial)	N/RN		X		X	X		X		X	X	X			
CRF completion (patient data collection)	N/RN		X		X	X		X		X	X	X			
Limited physical examination	N/RN/Dr		X	X		X	X	X	X						
ˣBAL	RP		X			X+		X		X+		X			
Administration of trial intervention	RP		X			X		X							
+IV D-Choline	N/RN		X+												
+Blood Sample (venous or arterial)	N/RN		X+			X+		X+		X+	X+				
Safety monitoring and reporting	RN/RP/PI$		X		X	X		X		X	X	X			
LRSQ	RN													X	X*
Mortality and re-admission	RN												X		

RN = Research Nurse, RP = Study respiratory physiotherapist or nurse practitioner or any other qualified person not involved in decision to wean or extubate, PI = Local Principal Investigator or delegate, N = Nurse (usual care), Dr = licensed medical practitioner.

ˣ Before each trial intervention

+ Only at Liverpool & Southampton and with specific consent (see section 12.2)

***** Seasons 1 & 2 only

$ Although all delegated staff are responsible for identifying and reporting adverse events, decisions regarding causality and seriousness, and sign-off of serious adverse event reports can only be undertaken by a licensed medical practitioner.

For peer review only

For peer review only - http://bmjopen.bmj.com/site/about/guidelines.xhtml

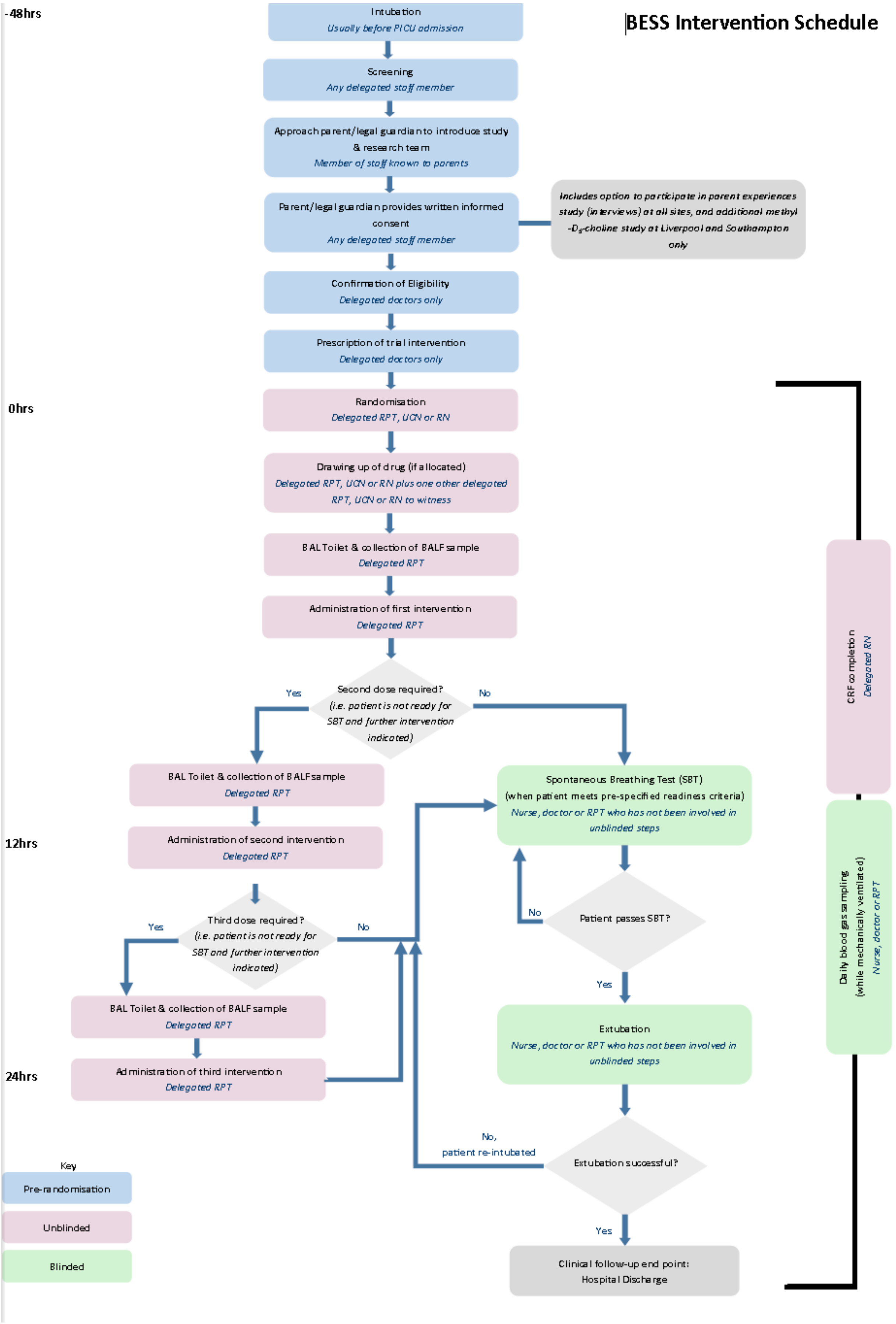

Figure 2: BESS Intervention Schedule

For peer review only - http://bmjopen.bmj.com/site/about/guidelines.xhtml

8.4 Procedures for assessing Efficacy

8.4.1 Efficacy Assessment 1

Efficacy will be assessed by group-wise comparison of the primary outcome measure group "Duration of MV after randomisation" by intention to treat. This is defined as time from randomisation to final extubation in hours, including any time off MV where the infant requires reintubation for a further period of MV. Time of randomisation and intervention will be recorded in the patients' notes and CRFs. MV ceases at extubation, an event when the endotracheal tube (breathing tube) is removed and the infant is allowed to breathe without invasive mechanical support. Extubation is an important discrete event in a patient's care pathway, is routinely recorded in the notes and PICANet audit.

8.4.2 Efficacy Assessment 2

The Ventilation Index (VI), Oxygenation Index (OI), Oxygenation Saturation Index (OSI) and SF ratio are secondary outcomes of efficacy derived from blood gas results (capillary blood gas sampling times as per Table 2 Schedule for intervention and follow-up), ventilator settings, and non-invasive monitoring. These data are captured through existing intensive care automated monitoring process or regular manual entry on large format (broad-sheet) bed-charts. This data will be transcribed by the research nurse to the CRF.
Mechanical Ventilation parameters to allow calculation of VI, OI, OSI and SF ratio must be recorded at the corresponding time when Blood Gas samples are scheduled to be taken for research or taken for routine purposes.
Mechanical Ventilation parameters are recorded regularly for ventilated patients as part of standard care. BESS does not require site teams to take any additional readings of these parameters for study purposes, but **all blood gas and ventilator readings** taken as part of standard care must be transcribed into the CRF to allow for calculation of these ratios over time.
After extubation, all inhaled Oxygen and peripheral oxygen saturation parameters recorded as part of standard care must be transcribed into the CRF to allow for calculation of this ratio over time.
Blood gas indices and ratio definitions are as follows:

- VI = ($PaCO_2$ × (PIP-PEEP) × respiratory rate)/1,000
- OI = (MAP × FiO_2 × 100)/PaO_2
- OSI = (MAP × FiO_2 × 100)/SpO_2
- SF = SpO_2/FiO_2

MAP = mean airway pressure
$PaCO_2$ = partial pressure of arterial carbon dioxide
FiO_2 = Fraction of air inspired that is oxygen
PaO_2 = partial pressure of arterial of oxygen
SpO_2= peripheral oxygen saturation

The time that physiological criteria are met that indicate suitability to test readiness for extubation by spontaneous breathing and/or the treating clinician believes the child to be ready for SBT will be recorded in the patient medical records and CRF.
All-cause mortality at 90 days after intervention will be established via a local enquiry against the hospital electronic health record.

For peer review only

For peer review only - http://bmjopen.bmj.com/site/about/guidelines.xhtml

8.5 Procedures for Assessing Safety

Safety will be assessed by:

- The Principal Investigator (PI) or delegated research staff actively monitoring and reporting all adverse events from randomisation until 24 hours after the participant's last administration of poractant alfa (see section 10 for adverse event reporting).
- Limited physical examination that includes the recording of vital signs before and after each study intervention
- Routinely collected patient care data downloaded from the PICANet audit on a regular basis by each site and transferred to the CTRC
- Mortality and readmission data collected by each site at 90 days post randomisation

Participants will be actively monitored and any adverse events identified must be reported to the CTRC, whether identified from trial assessments or standard care (e.g. identified during standard daily examinations). Non-related events will be reported from randomisation to 24 hours after the participant's last administration of poractant alfa or placebo for. Adverse events occurring after this time point until 90 days post randomisation / discharge home / death will be reported if felt to be related to the intervention. Any serious events or reactions (SAEs/SARs) should be reported to CTRC regardless of the timeframe.

8.5.1 Procedure for Limited Physical Examination in BESS

Participants in BESS are unwell and may be haemodynamically unstable. Handling of the participants, including full physical examination, is minimised in standard care, as per current NICE guidance (42).

All participants must have a physical examination before and after each administration of the trial intervention. This is a limited physical examination to minimise any disturbance to the infant participant.

Examinations must be carried out any time in the period 2 hours prior to each administration, and the interval of time between 1 hour and 2 hours post each administration. The post administration examination is deliberately delayed by at least one hour after administration to allow stabilisation of the participants' vital signs and avoid prolonging the handling associated with administration.

The limited examination must include the following:

- Temperature
- Heart rate
- Blood pressure
- Respiratory rate
- Oxygen saturations (peripheral)

To minimise handling, if a participant has had a full physical examination as part of standard care at any time in the period 2 hours prior to the administration and the vital signs data have been collected, then this examination should be counted as their BESS physical examination and the vital sign data used.

The physical examination vital sign data must be recorded in the patient notes and transcribed onto the relevant BESS CRF.

8.6 Other Assessments

8.6.1 Parent Reported Quality of Life questionnaires

The parent reported outcome measure the Liverpool Respiratory Symptom Questionnaire (LRSQ), is a web-accessible questionnaire that has been optimised for many platforms

For peer review only - http://bmjopen.bmj.com/site/about/guidelines.xhtml

including smart-phones. Links to the form will be emailed to parents prior to the intended completion dates (in all three seasons at 6m, and in addition at 12m to parents of infants recruited in seasons 1 and 2). A paper version is available with a “free-post” return envelope.

8.6.2 Special Assays or Procedures

8.6.2.1 Collection & processing of BAL fluid

BAL sampling with 0.9% sodium chloride (2x1mL/kg) will be done for all participants immediately prior to intervention, at 24hrs post intervention and repeated daily while the infant remains on MV (see Table 2).

Both tracheal toilet and BAL sampling is done by catheter access to the trachea via the endotracheal suction port without disconnecting the infant from the ventilator or by disconnecting the infant from the ventilator and performing hand bagging while accessing the trachea via a side-port with one-way valve.

8.6.2.2 Blood gas sampling

Blood Gas sampling is a routine procedure required for the clinical monitoring of all people requiring mechanical ventilation. The typical sample volume of blood drawn from infants and children is 100 microliters equivalent to two drops. Infants and children can have Blood Gas sampling by drawing blood from an existing arterial line or from a heel prick. To minimise disturbance and risks to infants from additional research procedures, the data from routine Blood Gas samples will be used for the trial when collected within a 2-hour window of the scheduled trial Blood Gas sample timepoint up to and including the sample due at 48 hours post first intervention (see Table 2). After 48 hours, i.e. day 3 and onwards, and only if the infant continues to require mechanical ventilation, then data from any one routine blood gas sample collected within a 6-hour window 9:00am to 3:00pm in each subsequent 24-hour interval will be collected (the first such sample if more than one is taken). A research Blood Gas sample will only be done if a routine blood gas sample is not done or planned within each 24-hour period sampling window.

Blood gas machines are usually kept in the PICU rather than the hospital/Trust laboratory. PICUs will not be CPS accredited but each Trust laboratory, and the machinery they are responsible for, will be. The CTRC will acquire a copy of the trust CPS laboratory certificate and calibration log for the blood gas machines as part of the compulsory ‘Green Light Checklist’ procedures before a site can open to recruitment. The Trust laboratory **must** take responsibility for service of the PICU blood gas machines and be able to provide a quality control log for maintenance and calibration of each blood gas machine.

8.6.2.3 Additional samples (WP-C at Liverpool and Southampton only)

Participants at Liverpool and Southampton will be given the option to consent to a single intravenous dose of D_9-labelled choline which will inform the mechanistic sub-study on surfactant synthesis and metabolism (WP-C; see section 12.2 for full details). This work will not inform either the primary or secondary outcomes of the main trial (WP-A). These samples will **only** be taken from participants who have provided specific consent for this optional additional work.

For peer review only - http://bmjopen.bmj.com/site/about/guidelines.xhtml

Small volume lung lavages will be collected immediately prior to intervention and at 24hrs, as per the standard follow-up (see section 8.2), and at additional time points of 12hrs and 36hrs for WP-C participants only. Samples of 1.25mL (range 1.0 to 1.3mL) of blood will be collected at the same time points for WP-C participants at Liverpool and Southampton only.

8.7 Patient Transfer and Withdrawal

8.7.1 Patient Transfers

It is very unlikely that participants taking part in BESS will be transferred during their trial treatment, given the severity of illness of participants and the short time period for administration of the intervention. In the unlikely scenario that a BESS participant is transferred while they remain intubated, their trial treatment will end at transfer unless they are transferred directly to another BESS site. For participants transferred to another participating trial site the receiving trial site must take over responsibility for the participant.

A copy of the participant CRFs should be provided to the new site. The parent will have to sign a new consent form at the new site, and until this occurs, the participant remains the responsibility of the original site.

If a participant is transferred to a site that is not taking part in BESS then they will be withdrawn from the trial and will receive no further interventions. Their care will revert to the standard practice and will not be affected by the withdrawal from the trial. The CTRC should be notified in writing of all participant transfers, whether or not to a participating trial site.

8.7.2 Withdrawal from the Trial

In consenting to the trial, parents are consenting to trial intervention and treatment, follow-up assessments and data collection for their child.

The rights and welfare of the participants will be protected by emphasising to their parents that the quality of medical care will not be adversely affected if they withdraw consent for their child to participate in this trial.

8.7.2.1 Partial Withdrawal from Trial (Intervention and Treatment), allowing Follow-up and Data Collection)

Participants may be withdrawn from trial intervention and treatment for any of the following reasons:

a. Parent withdraws consent.

b. Unacceptable toxicity.

c. Intercurrent illness preventing further treatment.

d. Any change in the participant's condition that justifies the discontinuation of treatment in the clinician's opinion.

If voluntary withdrawal from trial intervention and treatment occurs, the parent should be asked to allow continuation of scheduled evaluations (follow-up and data collection) and be given appropriate

For peer review only - http://bmjopen.bmj.com/site/about/guidelines.xhtml

care under medical supervision until the symptoms of any adverse event resolve or the participant's condition becomes stable.

If a parent wishes to withdraw their infant from trial intervention and treatment, sites should explain the importance of remaining on trial follow-up, or failing this, of allowing routine follow-up data to be used for trial purposes. Follow-up and data collection will continue unless the parent explicitly withdraws consent for follow-up or data collection. Research data obtained up to the point of withdrawal of consent will be retained, and further data will only be collected where required by law for reporting to regulatory agencies (i.e. serious adverse events). Research samples will be destroyed or retained for the purpose of the trial according to the wishes of the parent.

8.7.2.2 Withdrawal from Trial Completely

Parents are free to withdraw consent at any time without providing a reason. Parents who wish to withdraw consent for the trial completely (trial intervention, follow-up and data collection) will be informed that pseudo-anonymised data collected up to the point of that withdrawal of consent will be included in the analyses, in accordance with current data protection legislation. The participant will not contribute further data to the trial (with the exception of serious adverse event data); the CTRC should be informed in writing and a Withdrawal CRF should be completed. Data up to the time of withdrawal will be included in the analyses.

8.8 Loss to Follow-up

Trial follow-up will be in part via routinely collected data downloaded in a bespoke trial download by each recruiting site; in part via paper CRFs completed by the trial RNs and RPTs; and in part via LRSQs collecting Parent Reported Outcome Measures (PROMs) at 6 and 12 months post randomisation. Where a patient is lost to follow-up before the relevant time point (e.g. lost due to transfer to another hospital), contact will initially be attempted by the trial RNs and the PI at each site. Wherever possible, information on the reason for loss to follow-up will be recorded.

8.9 Trial Closure

The end of the trial is defined to be the date on which data for all participants are locked and data entry privileges are withdrawn from the trial database. However, the trial may be closed prematurely by the Trial Steering Committee (TSC), on the recommendation of the Independent Data and Safety Monitoring Committee (IDSMC).

Individual sites may be closed to recruitment prior to their intended recruitment end date if the Trial Management Group (TMG) have concerns about their capacity or capability to deliver the trial, or for operational reasons whereby resources are better used at sites with better capacity to recruit.

At the point of closure to recruitment all sites will be required to undertake closedown activities which include but are not limited to: a review of their Investigator Site File (ISF), a count of all completed CRFs and completed data queries. PIs will also be required to sign-off the CRF for each participant, any changes to the data, a closedown checklist and their site delegation log.

For peer review only - http://bmjopen.bmj.com/site/about/guidelines.xhtml

9 TRIAL TREATMENT/INTERVENTIONS

9.1 Introduction

The intervention being trialled is Endotracheal poractant alfa (Curosurf®, porcine lung phospholipid fraction supplied at 80mg/mL).

Curosurf® is a standard natural surfactant treatment prepared from porcine lungs for intratracheal administration and containing almost exclusively polar lipids, in particular phosphatidylcholine. Endotracheal poractant alfa is licensed for use in the UK for the treatment of Respiratory Distress Syndrome (RDS) or hyaline membrane disease in newborn babies (neonates). It is being used as an investigational medicinal product (IMP) on the BESS trial (UK licence, new indication).

Three small studies of surfactant use in infants with bronchiolitis have raised no safety concerns, however BESS will be the first large, randomised study of any surfactant for the treatment of bronchiolitis of infancy (3). As neonatal RDS is caused by surfactant deficiency, and the viral infections that cause bronchiolitis of infancy also cause surfactant deficiency (which we aim to treat with poractant alfa), we expect that the well tolerated safety profile of use in RDS in neonates will be reflected when using surfactant to treat bronchiolitis in infants.

Participants will be randomised equally to receive either endotracheal poractant alfa or air-placebo in a sham procedure. Participants randomised to receive endotracheal poractant alfa will receive a first dose of 200mg/kg, repeated 12 hourly at 100mg/kg, to a maximum of 3 doses. This is in line with the manufacturer's summary of product characteristics (SPC). Participants randomised to the placebo will receive an identical series of up to three procedures (while intubated) using air as the placebo.

9.2 Arm A – Endotracheal Poractant Alfa at 200mg/kg (first dose), with up to two further doses at 100mg/kg

9.2.1 Formulation, Packaging, Labelling, Storage and Stability

Generic name: Endotracheal poractant alfa

Brand name: Curosurf®

Supply and distribution: Chiesi Farmaceutici S.p.A (Chiesi) will supply Curosurf® via a third-party distributor and will provide appropriate certificates of analytical conformity. Additional shipments during each season will be managed by the distributor contracted by Chiesi, via an automated web-based system.

Packaging: Labelled holder containing one labelled glass vial with a blue topper.

Formulation: Each vial contains 3ml volume of poractant alfa at a concentration of 80mg/ml.

For peer review only - http://bmjopen.bmj.com/site/about/guidelines.xhtml

Labelling: Vials will be labelled by Chiesi in English and in compliance with the detailed guidance provided in annex 13 of the European Union (EU) Good Manufacturing Practice (GMP) guide. Each vial will be labelled and used for BESS trial use only.

Storage: The PI or delegated research team member will be responsible for the safe storage of all vials assigned to the BESS trial, in a safe place with restricted access (usually a designated drug room on the PICU). Poractant alfa will be stored between 2ºC and 8ºC, in a separate drug fridge specifically for use in the trial, protected from light in the original packaging.

Stability: Curosurf® has an unopened shelf life of 18 months. Each vial is for single use only.

9.2.2 Preparation, Dosage and Administration of Study Treatment/s

Dosage: An initial dose of 200mg/kg, with two further doses of 100mg/kg at 12hrs and 24hrs if indicated.

200mg/kg is 200milligrams of poractant alfa per kilogram of body weight. 100mg/kg is 100milligrams of poractant alfa per kilogram of body weight. The participant's weight will be detailed on the prescription in kilograms and the prescribing clinician will calculate the appropriate dose and number of vials needed for that participant.

Preparation: Poractant Alfa suspension is refrigerated, so before use each vial must be warmed up slowly to room temperature e.g. keeping it in hands for 5-10 minutes and gently turned upside down, without shaking, avoiding the formation of foam. Artificial warming methods should not be used.

The entire contents of the vials of poractant alfa suspension should be slowly withdrawn into a plastic syringe through a large-gauge needle and the excess poractant alfa discarded. A second person must be witness to the drawing up of the poractant alfa to assist and to check that the volume drawn up is the amount prescribed. Once the prescribed volume has been drawn into the syringe, an opaque paper shield should be applied to the syringe in order to maintain blinding of staff and/or parents at the bedside.

At this stage the appropriate equipment should also be prepared for conducting tracheal toilet and BAL.

Tracheal toilet (and, where scheduled, BAL) must be conducted prior to administration of the intervention.

The use of muscle relaxant prior to tracheal toilet, BAL and or administration of the intervention is at clinical discretion.

Administration: Either with the infant connected to the ventilator, or momentarily disconnected with hand-bagging, the syringe filled with Poractant Alfa suspension will be connected to a 5-French catheter end-hole inserted into the endotracheal tube (ETT) for ventilation. Passing the catheter through the suction or side port into the ETT and advancing the catheter aiming to have the tip of the catheter placed just beyond the tip of the ETT. The insertion length should be calculated from the known length of the ETT (typically cut to reduce dead space) plus the distance from the end of the cut edge of the ETT to the suction/side port). The intervention will be given slowly as a single bolus directly into the lower trachea (recommended over 0.5 to 3 minutes). From there it disperses through ventilation cycles into the peripheral lung tissue where the excipient (buffered 0.9% sodium chloride) is absorbed in the alveoli and the surfactant phospholipid is deposited.

The infant should be stabilized before surfactant administration and their head placed in the neutral position (head and body in alignment without inclination).

For peer review only - http://bmjopen.bmj.com/site/about/guidelines.xhtml

Before starting the procedure and throughout it, infants must have cardiorespiratory monitoring: oxygen saturation and heart rate continuously through pulse oximetry, blood pressure measurement either continuously in presence of an arterial catheter or intermittently if no arterial line is in place.
The first dose at 200 mg/kg will be followed up by two further doses at 100 mg/kg at approximately 12-hour intervals and the exact time and actual volume of each administration will be noted in the medical notes and appropriate section of the CRF.
No suctioning of the airways should be performed for two hours after each surfactant instillation unless the treating physician deems this appropriate, in which case this will be noted in the medical notes and appropriate section of the CRF.

9.2.3 Specific Restrictions

The effect of co-administration of poractant alfa with other agents is not known. Do not administer poractant alfa concomitantly in the same intratracheal bolus with other agents.

9.2.4 Overdose

Overdose may occur if the wrong patient weight has been supplied to the prescribing clinician, an incorrect weight is used in the calculation or the calculation has been done incorrectly. There have been no reports of overdose following the administration of Curosurf® however, in the unlikely event of an accidental overdose that causes clear clinical effects on the infant's respiration, ventilation or oxygenation, as much of the suspension should be aspirated as possible, as per the SPC.
Special care must be taken by all site team members to avoid overdosing, noticeably the prescribing clinician and the staff members drawing up the poractant alfa for administration. In the event of identification of an overdose, unblinding should occur and the participant will be closely observed for any adverse events (AE). The site team should notify the CTRC of the event and examine the reasons for the event occurring to ensure that it does not happen again. The CTRC will log any overdosing events as protocol deviations.

9.2.5 Temperature Compliance

Temperature deviations at site will be identified by the regular completion of a trial-specific temperature monitoring log. The PI or delegated member of staff will check the fridge used to store stocks of poractant alfa for use in BESS **daily** and will alert the CTRC if a temperature deviation has occurred. Temperature checks will be recorded on a Temperature Monitoring Log.

Poractant Alfa shipment and storage conditions:

**Poractant Alfa (Curosurf®)** Suspension 80 mg/ml to 3ml vial	+2°C to +8°C	Up to +25°C for maximum cumulative 96hrs

Unopened vials of Curosurf® that have warmed to room temperature can be returned to refrigerated storage **within 24hours** for future use, **if** this is the first time that they have warmed. Vials that have been removed from the fridge and been allowed to warm to room temperature for a second time **must not be used**, but should be retained for accountability checks at the end of each season.

Temperature compliance during shipment will be checked by the local site Pharmacist on receipt of the IMP. The local Pharmacist will review the temperature curves/logs from the calibrated

For peer review only - http://bmjopen.bmj.com/site/about/guidelines.xhtml

temperature logger used during IMP shipment and will check for any out of range values. If an out of range temperature is identified, the local Pharmacist will inform the CTRC within 48 hours. It is the responsibility of the CTRC to inform Chiesi, who will then notify the Chiesi R&D Quality Assurance GMP Unit & Project Partners (R&D QA-GMP).

If the out-of-range temperature deviation does not affect the quality and safety of the products, a declaration of usability will be issued and signed by R&D QA-GMP and sent back to the CTRC. CTRC will then authorise the trial site to use the vials in question.

If the products are judged not suitable for use, Chiesi R&D QA-GMP will inform the CTRC to permanently suspend the use of the affected vials. Any vials not used due to temperature deviations will be retained for accountability checks at the end of each season before being sent for destruction.

9.2.6 Accountability Procedures for Trial Treatments

The PI is responsible for the management of poractant alfa to be used for the trial. Trial IMP should be stored in the original packaging to protect from light, in a locked, secure, refrigerated storage facility with access limited to those individuals authorized to dispense or administer the trial IMP. Initial supply of the trial IMP will be to the site Pharmacy. The site Pharmacists are responsible for dispensing stock to a secure drug room on the PICU for more immediate access by the research team. Pharmacists must release the trial IMP to PICU in sequential order by the medication numbers on the labels to ensure the Randomisation System can allocate trial IMP with the shortest shelf life first. No surfactant material supplied for this trial is to be used for any other purpose.

Accountability logs will be maintained by the PI, pharmacist or delegated research team member, which will include a signed account of all the vials of Curosurf® received from the distributor, the vials dispensed to and returned from PICU, with the vials and volume used for each randomised patient.

All completely used vials will be retained at the site until the conclusion of the recruitment season. Each vial is for single use only. Any leftover unused medication is to be retained for drug accountability. Partially used vials present a risk of confusion and sharps injury, so all partially used vials will be disposed of within the PICU as per local Trust standard procedures. Disposal of partially used vials will be logged for accountability checks.
At the conclusion of each recruitment season, the PI, pharmacist or delegated individual shall conduct and document a drug supply (used and unused) inventory. An explanation will be given for any discrepancies.

After the inventory is reconciled at the end of each season and only once the CTRC have given the site authorisation on behalf of the sponsor, remaining unused vials of Curosurf® maybe disposed of directly by the Pharmacy of the involved trial site. Unused vials with an expiry date during or after the next season will be retained. Documentation assuring the unused vials were sent off-site for destruction, as per standard Trust procedures will be provided. This must be signed by two delegated members of staff filed in the Pharmacy site file, with a copy returned to the CTRC for storage in the Trial Master File.

For peer review only - http://bmjopen.bmj.com/site/about/guidelines.xhtml

9.2.7 Assessment of Compliance with Study Treatment/s

Each vial of Curosurf® will be designated by code number and the actual volume of instilled test treatment will be noted in the hospital chart and on the CRF of the study.

The completely used vials will be kept in a sealed labelled plastic box/envelope with patient's randomisation number/initials until the end of the season for accountability checks. There will therefore be three "final" accountability checks at each site, one at the end of each recruitment season.

9.3 Arm B – Placebo

Placebo: Air via an identical series of 3 doses in a sham procedure.

Dosage and preparation: Air should be drawn into a syringe at the same volume of 200mg/kg for the first dose and 100mg/kg for the second and third doses (if required), as per the instructions for poractant alfa dose calculations assuming the intervention is provided at 80mg/ml (section 9.2.2). Once the prescribed volume has been drawn into the syringe, an opaque paper shield should be applied to the syringe in order to maintain blinding of staff and/or parents at the bedside.

The use of muscle relaxant prior to tracheal toilet, BAL and or administration of the intervention is at clinical discretion.

Administration: Either with the infant connected to the ventilator, or momentarily disconnected with hand-bagging, the syringe filled with air will be connected to a 5-French catheter end-hole inserted into the endotracheal tube (ETT) for ventilation. Passing the catheter through the suction or side-port into the ETT and advancing the catheter aiming to have the tip of the catheter placed just beyond the tip of the ETT. The insertion length should be calculated from the known length of the ETT (typically cut to reduce dead space) plus the distance from the end of the cut edge of the ETT to the suction/side port). The air will be given slowly as a single bolus directly into the lower trachea (recommended over 0.5 to 3 minutes).

9.4 Unblinding

N.B. Allocation must not be routinely revealed to CTRC personnel. It is to be expected that some site staff may be accidentally unblinded due to unavoidable back-tracking of surfactant up the participant's ETT (see section 7.4).

To maintain the overall quality and legitimacy of the trial, unblinding should occur only in exceptional circumstances when knowledge of the actual treatment is **absolutely essential** for further management of the patient (SPIRIT 2013).

After unblinding the Local Investigator is encouraged to maintain the blind as far as possible. The actual allocation must NOT be disclosed to the participant's family or other study personnel including

For peer review only - http://bmjopen.bmj.com/site/about/guidelines.xhtml

other site personnel, monitors, sponsors or project office staff; nor should there be any written or verbal disclosure of the code in any of the corresponding patient documents.

The Investigator may unblind at their discretion without need to contact the CTRC first, but must report all unblinding (with reason) as they occur on the corresponding Unblinding CRF.

Unblinding should not necessarily be a reason for study drug discontinuation.

9.4.1 Emergency Unblinding

If a Local Investigator urgently needs to unblind a participant, they are able to do so by opening the unblinding envelope corresponding to the participant's randomisation number.

Partially completed unblinded envelopes will be provided to all sites. The allocation (poractant alfa or air as placebo), as generated by the WebEZ randomisation system, will be marked on a pre-printed slip inside the envelope by the randomising team member, and the envelope sealed. There will be one partially completed envelope for each patient, with the randomisation number clearly visible on the front of the envelope.

Sealed unblinding envelopes should be stored in a secure location that is readily accessible to PICU staff if the need for emergency unblinding arises.

In the case of any adverse event that requires emergency treatment and there is not sufficient time to unblind using the unblinding envelopes, Local Investigators should assume that the participant has been given poractant alfa (Arm A) and administer treatment accordingly. The treatment for any acute adverse event potentially associated with the study intervention would be the same whether the patient had been given surfactant or air. All adverse events should be reported as per the procedures described in section 10.

9.4.2 Accidental unblinding

If accidental unblinding occurs, this must be reported to the CTRC by use of the Unblinding CRF. When reporting include details about:

1. Date of unblinding;
2. Detailed explanation of circumstances;
3. Recipients of the unblinding information;
4. Action to prevent further occurrence, if possible.

9.4.3 Unblinding at Trial Closure

Upon trial closure the blinding of parents and blinded staff remains in effect. Site teams and/or pharmacy departments **should not** disclose treatment allocations on an individual basis.

For peer review only - http://bmjopen.bmj.com/site/about/guidelines.xhtml

9.5 Concomitant Medications

No incompatibilities or interactions of Curosurf® with other medications are known. There are therefore no restrictions placed by the trial on the use of concomitant medications in BESS participants.

For peer review only

For peer review only - http://bmjopen.bmj.com/site/about/guidelines.xhtml

10 SAFETY REPORTING

10.1 Terms and Definitions

"Adverse Event (AE)"

An adverse event (AE) is any untoward medical occurrence in a subject to whom a medicinal product has been administered, including occurrences which are not necessarily caused by or related to that product.

Therefore an AE is any unfavourable or unintended change in the function (symptoms), structure (signs) or chemistry (laboratory data) in a subject to whom an IMP has been administered, including occurrences which are not necessarily caused by or related to that product.

"Adverse Reaction (AR)"

An adverse reaction (AR) is any untoward and unintended response in a subject to an IMP that is related to any dose administered to that subject.

Therefore an AR is any unfavourable or unintended change in the function (symptoms), structure (signs) or chemistry (laboratory data) in a subject that is related to any dose of an IMP administered to that subject.

"Unexpected Adverse Reaction (UAR)"

An unexpected adverse reaction (UAR) is an adverse reaction the nature and severity of which is not consistent with the information about the medicinal product in question set out in the Investigator's Brochure (IB) or the SPC, which may be referenced where the IMP in question is a product with a marketing authorisation.

"Serious Adverse Event (SAE), "Serious Adverse Reaction, or Unexpected Serious Adverse Reaction"

A serious adverse event (SAE) is an AE, AR or UAR respectively that:

- results in death;
- is life threatening (places the subject, in the view of the Investigator, at immediate risk of death from the experience as it occurred – this does not include an adverse experience that, had it occurred in a more severe form, might have caused death);
- requires hospitalisation or prolongation of existing hospitalisation (hospitalisation is defined as an in-patient admission, regardless of length of stay, even if the hospitalisation is a precautionary measure for continued observation – hospitalisations for a pre-existing condition, including elective procedures that have not worsened, do not constitute an SAE);
- results in persistent or significant disability or incapacity (substantial disruption of one's ability to conduct normal life functions);
- consists of a congenital anomaly or birth defect (in offspring of subjects, or their partners, taking the IMP regardless of time of diagnosis);
- other important medical events (these may not result in death, be life-threatening, or require hospitalisation, but may be considered a serious adverse event or experience when, based upon appropriate medical judgment, they are considered to jeopardise the subject and may require medical or surgical intervention to prevent one of the outcomes listed in this definition).

For peer review only - http://bmjopen.bmj.com/site/about/guidelines.xhtml

"Suspected Serious Adverse Reaction (SSAR)"

A suspected serious adverse reaction (SSAR) is an adverse reaction that is classed in nature as serious and which is consistent with the information about the IMP in question, which in the case of a licensed product is set out in the SPC for that product, and in the case of any other IMP is set out in the IB relating to the trial in question.

"Suspected Unexpected Serious Adverse Reaction (SUSAR)"

A suspected unexpected serious adverse reaction (SUSAR) is an adverse reaction that is classed in nature as serious and which is not consistent with the information about the IMP in question, which in the case of a licensed product is set out in the SPC for that product, and in the case of any other IMP is set out in the IB relating to the trial in question.

"Reference Safety Information (RSI)"

The reference safety information (RSI) for a trial is the information used for assessing whether an adverse reaction is expected. This is contained in either the IB or the SPC.

10.2 Notes on Adverse Event Inclusions and Exclusions

The below sections provide guidance on what should, and should not be considered as AEs for the purposes of the BESS study:

10.2.1 Include:

- An exacerbation of a pre-existing illness, **excluding** the current case of bronchiolitis.
- An increase in frequency or intensity of a pre-existing episodic event/condition.
- A condition (even though it may have been present prior to the start of the trial) detected after trial drug administration.
- Continuous persistent disease or symptoms present at baseline that worsen following the administration of the trial treatment, **excluding** the current case of Bronchiolitis.
- Laboratory abnormalities that require clinical intervention or further investigation (unless they are associated with an already reported clinical event).
- Abnormalities in physiological testing or physical examination that require further investigation or clinical intervention.
- Injury or accidents.

10.2.2 Do Not Include:

- Widely recognised and documented clinical features of bronchiolitis or outcomes associated with MV that are defined outcome measures in this trial (see section 3.5).
- Medical or surgical procedures* – the condition which leads to the procedure is the adverse event; e.g. pneumothorax leading to chest drain placement, here pneumothorax is the event.
- Pre-existing disease or conditions present before treatment that do not worsen.
- Situations where an untoward medical occurrence has occurred, e.g. cosmetic elective surgery.
- Overdose of medication without symptoms or signs**.

For peer review only - http://bmjopen.bmj.com/site/about/guidelines.xhtml

For peer review only

* *Cosmetic elective surgery is cited here as example of an event that is not reportable as an AE.*
** See also section 9.2.4. *If overdose occurred with resulting symptoms and signs that met the protocol criteria for AE/AR/SAE/SAR then they should be reported accordingly. Please note that though overdose of medication without symptoms or signs may be excluded from AE reporting this may still require investigation to ensure the protocol and regulatory requirements are met, e.g. for IMP management and administration to ensure participant safety.*

10.2.3 Notification of deaths

There is an overall likelihood of death in all cases of severe bronchiolitis of 1-2%. All deaths that occur during the protocol-specified AE reporting period, regardless of relationship to study drug, must be recorded. All deaths should be reported on a “Serious Adverse Event CRF” and returned to the CTRC within 24 hours.

Death should be considered an outcome and not a distinct event. The event or condition that caused or contributed to the fatal outcome should be recorded as the single medical concept on the appropriate CRF; e.g. death following pneumothorax, here pneumothorax is the event. Generally, only one such event should be reported. The term “sudden death” should only be used for the occurrence of an abrupt and unexpected death due to presumed cardiac causes in a participant with or without pre-existing heart disease, within 1 hour of the onset of acute symptoms or, in the case of an unwitnessed death, within 24 hours after the participant was last seen alive and stable. If the cause of death is unknown and cannot be ascertained at the time of reporting, “unexplained death” should be recorded on the appropriate CRF. If the cause of death subsequently becomes available (e.g. after post-mortem examination), “unexplained death” should be replaced by the established cause of death.

10.3 Notes on Severity / Grading of Adverse Events

The assignment of the severity/grading should be made by the investigator responsible for the care of the participant using the definitions below.

Regardless of the classification of an AE as serious or not, its severity must be assessed according to medical criteria alone using the following categories:

Mild: does not interfere with routine activities
Moderate: interferes with routine activities

Severe: impossible to perform routine activities

A distinction is drawn between serious and severe AEs. Severity is a measure of intensity (see above) whereas seriousness is defined using the criteria in section 10.1, hence, a severe AE need not necessarily be a SAE.

For peer review only - http://bmjopen.bmj.com/site/about/guidelines.xhtml

10.4 Relationship to Trial Treatment

The assignment of the causality should be made by the investigator responsible for the care of the participant using the definitions in Table 3: Definitions of Causality. An AE for which the causal relationship to the study drug is assessed by the investigator as “possible”, “probable” or “almost certain” is an AR.

If any doubt about the causality exists the local investigator should inform the CTRC who will notify the Chief Investigator (CI). In the case of discrepant views on causality between the investigator and others, the MHRA will be informed of both points of view.

Table 3: Definitions of Causality

Relationship	Description
**Unrelated**	There is no evidence of any causal relationship. N.B. An alternative cause for the AE should be given.
**Unlikely**	There is little evidence to suggest there is a causal relationship (e.g. the event did not occur within a reasonable time after administration of the trial medication). There is another reasonable explanation for the event (e.g. the participant’s clinical condition, other concomitant treatment).
**Possibly**	There is some evidence to suggest a causal relationship (e.g. because the event occurs within a reasonable time after administration of the trial medication). However, the influence of other factors may have contributed to the event (e.g. the participant’s clinical condition, other concomitant treatments).
**Probably**	There is evidence to suggest a causal relationship and the influence of other factors is unlikely.
**Almost certainly**	There is clear evidence to suggest a causal relationship and other possible contributing factors can be ruled out.

10.5 Expectedness

The Reference Safety Information (RSI) in BESS is section 4.8 (Undesirable effects) of the Curosurf® Endotracheopulmonary Instillation Suspension SPC.

It is not a regulatory requirement for a reporting physician to provide their opinion of expectedness. Therefore, the reporting physician at the local research site will not be asked to make an assessment of expectedness. The assessment of expectedness will be made by the CI (or their medically qualified delegate) using BESS’s current MHRA-approved RSI following receipt of the “Serious Adverse Event CRF” at CTRC.

All events judged by the investigator to be possibly, probably, or almost certainly related to the IMP, graded as serious and unexpected (see current MHRA-approved trial RSI (Curosurf® Endotracheopulmonary Instillation Suspension SPC) for list of Expected Adverse Events) should be reported as a SUSAR.

For peer review only - http://bmjopen.bmj.com/site/about/guidelines.xhtml

10.6 Follow-up After Adverse Events

All adverse events should be followed until satisfactory resolution or until the investigator responsible for the care of the participant deems the event to be chronic or the participant to be stable.

When reporting SAEs and SUSARs the investigator responsible for the care of the participant should apply the following criteria to provide information relating to event outcomes:

- resolved;
- resolved with sequelae (specifying with additional narrative);
- not resolved/ongoing;
- on-going at final follow-up;
- fatal or unknown.

10.7 Time Period for Safety Reporting

Investigators and delegated members of the local research teams will report to CTRC on the appropriate CRFs (see section 10.8):

- all adverse events from randomisation until 24 hours after their final intervention (whether poractant alfa or placebo);
- all adverse reactions from randomisation until 90 days post-randomisation or hospital discharge (whichever is sooner).

Following these periods, site staff are not required to report any non-serious events, however any serious events (SAEs/SARs/SUSARs) which they become aware of must be reported to CTRC.

Upon becoming aware of a serious adverse event (SAE/SAR/SUSAR), the Investigator or other delegated member of the team **must** report this to the CTRC **within 24 hours**.

10.8 Reporting Procedures

All serious events (SAEs/SARs/SUSARs) must be reported in an expedited manner. Depending on the nature of the event the reporting procedures below should be followed. Any questions concerning adverse event reporting should be directed to the CTRC in the first instance. If a site has a serious or urgent query regarding an adverse event outside of CTRC office hours they should contact the Chief Investigator, Professor Calum Semple, on 07506 653 560.

A flowchart is given below to aid in determining reporting requirements.

For peer review only - http://bmjopen.bmj.com/site/about/guidelines.xhtml

10.8.1 Flowchart for Reporting Requirements of Adverse Events

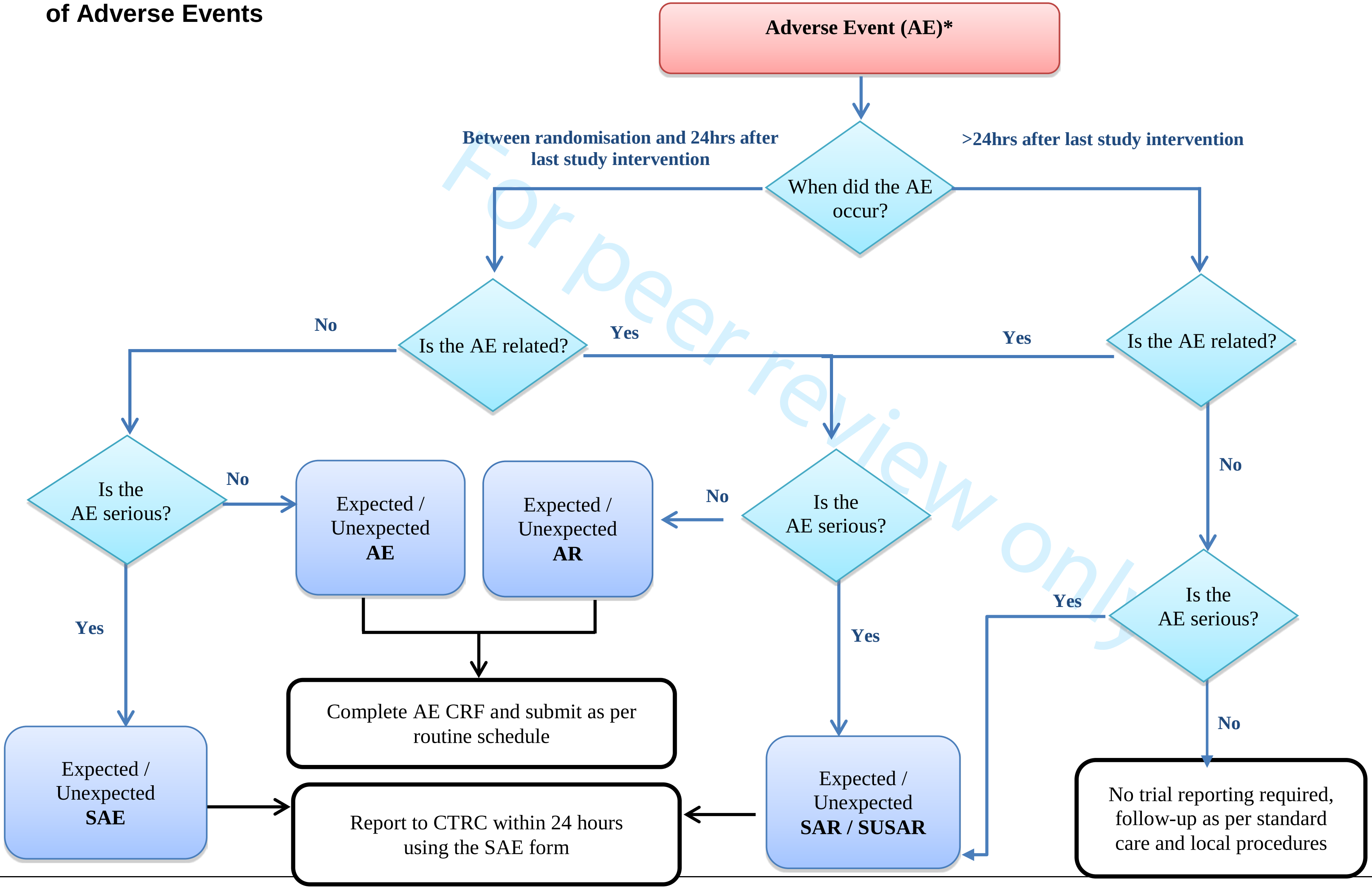

For peer review only - http://bmjopen.bmj.com/site/about/guidelines.xhtml

For peer review only

For peer review only - http://bmjopen.bmj.com/site/about/guidelines.xhtml

10.8.2 Non serious events (ARs/AEs)

All non-serious events (i.e. AEs and ARs), whether expected or not, should be recorded on an “Adverse Event CRF”, which should be transmitted to the CTRC within seven days of the local research team becoming aware of the event.

10.8.3 Serious events (AEs/ARs/SUSARs)

All serious events (i.e. SAEs, SARs and SUSARs) must be reported to CTRC within 24 hours of the local site becoming aware of the event. The “Serious Adverse Event CRF” asks for the nature of the event, date of onset, severity, corrective therapies given, outcome and causality. The reporting local Investigator should assign the causality of the event. Additional information should be sent within 5 days if the event has not resolved at the time of initial reporting.

All adverse events assessed as serious, related and unexpected (i.e. SUSARs) will be reported on to the MHRA and BESS’s Research Ethics Committee (REC) by the CTRC according to the following timelines; fatal and life-threatening SUSARs within 7 days of notification to CTRC and non-life threatening SUSARs within 15 days. BESS PIs will be notified of all SUSARs occurring throughout the trial by the CTRC. Local Investigators should report any SUSARs and/or SAEs/SARs as required locally.

NOTE: *please ensure that multiple Serious Adverse Events are reported separately to the CTRC. Each individual SAE report should relate to one overall diagnosis only.*

10.9 Safety Reporting Responsibilities – Investigator

The local site Investigator (site Principal Investigator (PI)) is responsible for reporting all events that are observed or reported during the study, regardless of their relationship to study product (i.e. AEs/ARs/SAEs/SARs/SUSARs)..

All serious adverse events (SAEs/SARs/SUSARs) must be reported immediately by the investigator to the CTRC on an SAE. All non-serious adverse events should be reported to CTRC within seven days of the local research team becoming aware on an AE CRF.

Minimum information required for reporting serious events

- Valid European Clinical Trials Database (EudraCT) number (if applicable)
- Sponsor study number
- One identifiable coded subject (randomisation number)
- One identifiable reporter (name of PI or appropriate delegated individual)
- One SAE
- One suspect IMP (poractant alfa)
- A seriousness assessment
- A causality assessment

For peer review only - http://bmjopen.bmj.com/site/about/guidelines.xhtml

Process for reporting serious events

i. The SAE CRF should be completed by a local Investigator, named on the 'signature list and delegation of responsibilities log' as responsible for reporting SAEs and making trial related medical decisions. The Investigator should assess the SAE for the likelihood that it is a response to the trial intervention. In the absence of the designated Investigator the SAE CRF should be completed and signed by an alternative member of the research site trial team and submitted to the CTRC. As soon as possible thereafter the responsible Investigator should check the SAE CRF, make amendments as appropriate, sign and re-send to the CTRC. The initial report shall be followed by detailed follow-up CRF reports as appropriate (see number vii).

ii. The "Serious Adverse Event CRF" **must** be submitted to the CTRC **within 24 hours** of the local research team becoming aware of the event; preferably this will be via fax.

Email address: bess@liverpool.ac.uk

Fax Number: 0151 795 8770

iii. When submitting a SAE CRF to the CTRC research sites must also telephone the appropriate Trial Co-ordinator or Data Manager on telephone number **0151 795 8757** to advise that an SAE CRF has been submitted.

iv. The participant **must** be identified by trial randomisation number, date of birth and initials **only**. The participant's name **must not** be used on any correspondence.

v. The local reporting Investigator must **notify** their R&D department of the event (as per standard local governance procedures).

vi. The participant must be followed-up until clinical recovery is complete and laboratory results have returned to normal, or until the event has stabilised. Follow-up should continue after completion of protocol treatment as necessary.

vii. Follow-up information should be noted on a separate CRF by ticking the box marked 'follow-up' and submitting to the CTRC as information becomes available. Extra, annotated information and/or anonymised copies of test results may be provided separately.

10.9.1 Maintenance of Blinding

Systems for SUSAR and SAR reporting should, as far as possible, maintain blinding of individual clinicians and of trials staff involved in the day-to-day running of the trial. The medical management of participants for adverse events should be undertaken under the assumption that the patient has been given surfactant (Arm A). The safety of participants in the trial always takes priority. In each report, seriousness, causality and expectedness should be evaluated for poractant alfa unless unblinding has taken place. All instances of unblinding should be reported to the CTRC.

For peer review only - http://bmjopen.bmj.com/site/about/guidelines.xhtml

Cases that are considered serious, unexpected and possibly, probably or almost certainly related to one of the trial therapies (i.e. SUSARs) would have to be unblinded at the CTRC prior to reporting to the MHRA and REC.

10.10 Safety Reporting Responsibilities – CTRC

The CTRC is undertaking duties delegated by the trial Sponsor and is responsible for the notification of serious events (SAEs/SARs/SUSARs) to the CI (or appropriate delegate) for review and expectedness assessment, and onward expedited reporting of SUSARs and other SARs to the MHRA and REC. The CTRC will also notify PIs at participating sites of all trial SUSARs.

Upon receipt of a SAE CRF, CTRC will liaise with the trial CI (or appropriate delegate) who will review the events reported for seriousness and causality as assessed by the local site research team, and provide and assessment of expectedness. These will be reviewed within 24 hours of receipt at CTRC and those that are identified as SUSARs will be reported to MHRA, REC and Chiesi Farmaceutici S.p.A. by the CTRC. The causality assessment given by the local Investigator at the hospital cannot be overruled and in the case of disagreement, both opinions will be provided with the report.

Timelines of onward reporting to MHRA and REC are as follows:

- SUSARs that are fatal or life-threatening must be reported not later than 7 days after the CTRC is first aware of the reaction. Any additional relevant information must be reported within a further 8 days.
- SUSARs that are not fatal or life-threatening must be reported within 15 days of the CTRC first becoming aware of the reaction.
- A list of all SARs (expected and unexpected) must be reported annually.

The CTRC will also inform Chiesi Global Pharmacovigilance (Parma, Italy) of any adverse event identified as a SUSAR, within 7 days of the CTRC first becoming aware of the reaction.

It is recommended that the following safety issues should also be reported in an expedited fashion:

- an increase in the rate of occurrence or a qualitative change of an expected SAR, which is judged to be clinically important;
- new events related to the conduct of the trial or the development of the IMP and likely to affect the safety of the participants, such as:
    a. a SAE that could be associated with the trial procedures and which could modify the conduct of the trial;
    b. a significant hazard to the participant population, such as lack of efficacy of an IMP used for the treatment of a life-threatening disease;
    c. a major safety finding from a newly completed animal study (such as carcinogenicity);
    d. any anticipated end or temporary halt of a trial for safety reasons and conducted with the same IMP in another country by the same sponsor;
- recommendations of the IDSMC, if any, where relevant for the safety of the participants.

For peer review only - http://bmjopen.bmj.com/site/about/guidelines.xhtml

Participant safety incidents that take place in the course of research should be reported to the National Reporting and Learning System (NRLS) by each participating NHS Trust in accordance with local reporting procedures.

10.11 Safety reports

Safety reports will be generated during the course of the trial which allows for monitoring of serious events (SAEs/SARs/SUSARs) reporting rates across sites. The CTRC will send developmental safety update reports (DSUR) containing a list of all SARs to MHRA and the trial's main REC. Any concerns raised by the IDSMC or inconsistencies noted at a given site may prompt additional training at sites, with the potential for the CTRC to carry out site visits if there is suspicion of unreported AEs in patient case notes. Additional training will also be provided if unacceptable delay in safety reporting timelines. If any safety reports identify issues that have implications for the safety of trial participants, the PIs at all institutions participating in the trial will be notified.

10.12 Urgent Safety Measures

An urgent safety measure is a procedure not defined by the protocol, which is put in place prior to authorisation by the MHRA and REC in order to protect clinical trial participants from any immediate hazard to their health and safety.

The CTRC will notify the MHRA and REC immediately and, in any event, within 3 days that such a measure has been taken and the reasons why it has been taken. The initial notification to the MHRA will be by telephone (ideally within 24 hours) and a notice in writing will be sent within 3 days, setting out the reasons for the urgent safety measure and the plan for further action. After discussion with the MHRA and REC, further action will be agreed, which may include submission of a substantial amendment, a temporary halt, or permanent termination of the trial.

If the study is temporarily halted it may not recommence until authorised to do so by the MHRA and REC. If the study is permanently terminated before the date specified for its conclusion (in the original applications to MHRA and REC), the CTRC should notify the MHRA and REC within 15 days of the date of termination by submitting the formal End of Trial Notification.

10.13 Contact Details and Out-of-hours Medical Cover

Participants will be instructed to contact the local Investigator (their office number) during working hours. All local PIs should ensure to delegate at least one other appropriately trained (in the study and in GCP) medically qualified doctor for assessment of adverse events, safety reporting and making medical decisions in their absence.
Outside working hours the normal local procedure should be followed to contact the local site PI or their agreed delegate. PICU, ward and local switchboard staff should be aware where they should direct calls in- and out-of-hours.
The Chief Investigator has delegated two medically qualified and appropriately experienced doctors to assess safety reports in his absence (Prof Paul McNamara & Dr Kent Thorburn).
The CTRC should be the main point of contact for trial questions during opening hours – 0151 795 8757. Out-of-hours cover for trial related clinical issues that require urgent attention will be provided by the Chief Investigator or his delegates, on 07506 653 560.

For peer review only - http://bmjopen.bmj.com/site/about/guidelines.xhtml

11 STATISTICAL CONSIDERATIONS

11.1 Introduction

Separate Statistical Analysis Plans (SAPs) will be developed prior to the interim and final analyses of the trial.

11.2 Method of Randomisation

Participants will be randomised to receive either poractant alfa or air placebo in a 1:1 ratio. The randomisation list will be generated by an independent statistician at the CTRC who is not otherwise involved in the BESS trial. Randomisation lists will be computer-generated using block randomisation with random variable block length, stratified by site and duration of ventilation (<24hrs and >=24hrs) prior to randomisation.

11.3 Sample Size calculation

The study will be powered to detect a minimally clinically important difference of 18 hours (0.75 days) in the primary outcome variable of time of mechanical ventilation. Data are expected to be positively skewed, and will therefore be log-transformed, and resulting data back-transformed. The null hypothesis is that of no difference in time of MV between the two groups.

Data from Alder Hey Children's Hospital from an audit of 60 eligible infants admitted to PICU in the seasons 2012/3 and 2013/4 showed a mean (SD) duration of ventilation of 4.83 (2.5) days (PICANet data, 2011-14). Parameters for sample size estimation are therefore as follows:

$E(Y_1) = 4.83$ and $SD(Y_1) = 2.5$
$E(Y_2) = 4.08$ and $SD(Y_2) = 2.5$
$\delta = E(Y_2)/E(Y_1) = 0.845$
giving coefficients of variation:
$\kappa_1 = SD(Y_1)/E(Y_1) = 2.5/4.83 = 0.518$
$\kappa_2 = SD(Y_2)/E(Y_2) = 2.5/4.08 = 0.613$

Using the formula of Wolfe and Carlin (1999) with $r = 1$ for equal allocation and a two-sided alpha-level of 0.05, sample size estimations are 100 per group for 80% power and 134 per group for 90% power.

Taking the sample size of 134 and allowing for 5% dropout requires 142 patients will be recruited to each group, giving a total of 284 participants overall.

11.4 Interim Monitoring and Analyses

Analyses of the accumulating data will be performed at regular intervals (at least annually) for review by an IDSMC. These analyses will be performed at the CTRC. The IDSMC will be asked to give advice on whether the accumulated data from the trial, together with results from other relevant

For peer review only - http://bmjopen.bmj.com/site/about/guidelines.xhtml

trials, justifies continuing recruitment of further patients or further follow-up. The IDSMC will make recommendations to the Trial Steering Committee (TSC). A decision to discontinue recruitment, in all participants or in selected subgroups will be made only if the result is likely to convince a broad range of clinicians including participants in the trial and the general clinical community.

11.5 Analysis Plan

A full SAP will be written prior to the conduct of any comparative analysis of the treatment arms. The main features of the SAP are included here.

The primary analysis will be a test of the difference in geometric mean durations of MV between groups (placebo vs. poractant alfa), reported with the corresponding 95% confidence interval. The analysis will be conducted according to the intent-to-treat principle, and will incorporate the stratification factors of duration of ventilation (<24hrs and >=24hrs) prior to randomisation and site.

For the secondary outcomes, longitudinal data for VI, OI, OSI and SF will be analysed with joint modelling. LRSQ data will be modelled longitudinally and comparisons made between groups. Binary data will be reported in terms of relative risk with 95% confidence intervals.

Within BESS, data will be collected as to ascertain readiness for spontaneous breathing test and time of extubation.
As much information as possible will be collected about the reasons for missing outcome data; this will be used to inform any imputation approaches employed in the analysis. Such methods will be fully described in the SAP.

For peer review only - http://bmjopen.bmj.com/site/about/guidelines.xhtml

12 EXPLORATORY & TRANSLATIONAL WORK; WORK PACKAGES B AND C

12.1 Work Package B: Study of Parent & Staff Experiences

BESS will involve an embedded mixed method sub-study involving parents and BESS recruiters involved in recruitment and consent processes. WP-B will explore: acceptability of the trial; approach to recruitment and consent; decision making in the emergency setting; barriers to participation; and the content and format of trial information materials. This will involve: questionnaires and interviews with parents approached for informed consent in BESS, including those who do not go on to be randomised into the trial, and a survey of BESS recruiters.

Parent recruitment in season 1

In season 1, all parents approached for informed consent in BESS will be asked to complete a brief questionnaire and/or take part in a telephone interview. Recruitment to the sub study will occur shortly after the main trial recruitment discussion. This will include parents who decline to provide informed consent for the trial. If more than one parent is involved in the consent discussion, then both are able to complete a questionnaire. By completing the questionnaire, this confirms that the parent has consented to this providing information.

Parents will be asked to place completed questionnaires in a sealed stamped addressed envelope and return it to the BESS recruiter who will post to the research team. Recruitment for questionnaires will take place throughout season 1. In the rare instance that consent is not sought prior to discharge/transferred to another hospital, BESS recruiters should send the questionnaire to parents along with the Parent/Person with PISC to complete.

BESS recruiters will ask the parent to provide contact details on the BESS consent form if they wish to take part in a telephone interview. A researcher will contact families to arrange telephone interviews within one month of consent. At this point parents who live in (or close to) the Merseyside area will be provided with the option of a face to face interview at their home if they prefer. All families who express an interest in taking part but are not selected for an interview will be contacted via telephone or email to thank them for their interest in the study.

All interviews will be conducted by a researcher who both have proven skills in the conduct of research in sensitive settings. Any distress during the interviews will be managed with care and compassion and participants will be free to decline to answer any questions that they do not wish to answer or to stop the interviews at any point. Any such families will be supported in obtaining appropriate help and, after discussion with the family, the lead clinician responsible for the child's care will be informed to offer any support. Interviews will be conducted until data saturation point is achieved (approximately 15-25 based on other studies). All interview participants will be sent a £20 shopping voucher to thank them for their time. Sites will be informed when recruitment to interviews can stop.

Parent recruitment in season 2

For peer review only - http://bmjopen.bmj.com/site/about/guidelines.xhtml

In season 2, all parents will be asked to complete a brief questionnaire. This will be the same questionnaire as used in season 1 and include parents who decline informed consent. Questionnaire findings will help evaluate parents' views on recruitment and consent, including any changes made to our recruitment approach following season 1.

Staff recruitment in seasons 1 and 2
All staff involved in trial conduct, recruitment and consent in participating sites will be sent an email invitation and information sheet to request their participation in an online questionnaire towards the end of season 1 and 2. Completion of the questionnaire will be taken as an indication of consent.

Findings from parent questionnaires, interviews and staff surveys in season 1 will be used to develop approaches to consent, recruitment, trial information materials and staff training in season 2. Findings from season 2 parent and staff questionnaires will inform the design/training for the approach to recruitment and consent in season 3.

12.1.1 Parent and staff perspectives sub study analysis

Analysis of data from the mixed method sub study will be assisted using NVivo 8 qualitative data analysis package and SPSS software for statistical analysis. Quantitative analysis will involve simple descriptive statistics and the chi-square test for trend. Qualitative data from will be analysed thematically (32). Data from study methods will be analysed separately then synthesised (33) through the use of constant comparative analysis (34).

12.2 Work Package C:

Understanding the mechanism of efficacy or failure for exogenous surfactant to modulate bronchiolitis disease requires an integrated investigation of the subject's alveolar environment. This includes identifying the infective aetiology, describing the local inflammatory response, and defining the kinetics of surfactant lipid and protein synthesis; all linked to natural history, trial intervention and participant outcomes. The selected mechanistic sub studies leverage the unique collaboration between experts in bronchiolitis pathogenesis at Liverpool and surfactant physiology at Southampton. While the sub studies are defined individually, their outputs integrate to provide a cogent thesis.

Hypotheses to test include:
At all sites by analysis of BAL fluid sampled just prior to intervention at 0hr and 24hr:

- Surfactant concentration and composition predicts duration of MV.
- Recovery of synthesis of surfactant proteins A & D is modulated by exogenous surfactant.
- Lower airway inflammation is modulated by exogenous surfactant.[3]
- Treatment failure is associated with detection of specific pathogens.

[3] Costs for this component of BESS will not be charged to NIHR EME.

For peer review only - http://bmjopen.bmj.com/site/about/guidelines.xhtml

At Liverpool & Southampton by giving a single intravenous dose of deuterium-labelled[4] choline (*methyl*-D_9-choline chloride (D_9-Choline)) and with sampling of BAL fluid and blood just prior to intervention at 0hr, 12hr, 24hr and 36hr.

- Synthesis of endogenous surfactant phosphatidylcholine is modulated by the provision of exogenous surfactant.

12.2.1 Metabolic profiling of surfactant composition and kinetics

Analysis of surfactant phospholipid and protein concentration, composition and synthesis in BAL fluid from ventilated infants will help provide an explanation for the beneficial effects, or otherwise, of exogenous surfactant therapy for acute lung injury in infants.
The sub-study of the metabolic profiling of surfactant composition and kinetics has two linked parts. The first "**Surfactant concentration and composition**" utilises BAL fluid collected as part of the whole trial at any site and does not involve any additional intervention or sampling procedures. The second part "**Kinetics of surfactant phosphatidylcholine synthesis"** is a more complex study that requires additional interventions, specific consent and consequently will recruit only from Liverpool and Southampton

12.2.1.1 Surfactant concentration and composition

This will address the hypothesis that: Surfactant concentration and composition predicts duration of MV.
Infants will be recruited to this part of the mechanistic sub-study from all BESS trial centres. Individual molecular species of phosphatidylcholine (PC), phosphatidylglycerol (PG) phosphatidylinositol (PI) and sphingomyelin (SM) will be analysed in aliquots of small volume BAL fluid samples taken prior to trial intervention at 0 and 24hr hours of the study if still intubated. These phospholipids will be analysed by electrospray ionisation mass spectrometry (ESI-MS) (29, 30) in Southampton correcting for sample dilution by urea analysis(28). Surfactant proteins A, B, C, and D will be analysed by ELISA and western blotting.

12.2.1.2 Kinetics of surfactant phosphatidylcholine synthesis

This will address the hypothesis that: Synthesis of endogenous surfactant phosphatidylcholine is modulated by the provision of exogenous surfactant.
This part of the mechanistic sub-study will be conducted at Liverpool and Southampton trial sites only. Infants at these two centres will receive a single intravenous dose of D_9-choline (3.6 mg/kg body weight) prior sampling of BAL fluid and trial intervention at time = 0 hours, followed by further BAL fluid sampling at 12, 24, 36 and 48 hours if still ventilated. A single intravenous dose of deuterium-labelled choline administered prior to intervention allows assay of endogenous surfactant synthesis (31). A small blood sample (1.25ml (range 1.0 to 1.3 ml)) will also be taken at these times. D_9-choline contains the stable isotope deuterium, which is non-radioactive and non-toxic. Choline is an essential vitamin and the dose is within the daily-recommended allowance. Investigators at University of Southampton have extensive experience administering this metabolic probe in clinical

[4] Deuterium is one of the two stable (non-radioactive) and naturally occurring isotopes of hydrogen that is used as a harmless metabolic tracer in animal and human studies.

For peer review only - http://bmjopen.bmj.com/site/about/guidelines.xhtml

studies of: preterm infants, infants with acute lung injury (ALI), and adults with acute respiratory distress syndrome patients with no adverse effects. ESI-MS analysis of unlabelled PC and PC labelled by incorporation of D_9-choline will provide a measure of surfactant phospholipid synthesis and turnover.

12.2.2 Recovery of surfactant proteins A & D (SP-A & SP-D) is modulated by exogenous surfactant.

Presence of SP-A and SP-D in the plasma has been proposed as a biomarker for acute lung injury (32). Among children mechanically ventilated for ALI including in bronchiolitis, we have previously shown reduced levels of SP-A and SP-D in the early stages of the disease, with recovery in SP-D levels in BAL fluid linked to progress to extubation, along with normalisation of DPPC and PG levels (33).

It is thought that surfactant deficiency contributes to acute lung injury by direct disruption of type II cell function and enhanced degradation of the surfactant system. SPA is secreted along with the phospholipid components of surfactant in lamellar bodies. Thus, metabolic studies demonstrating decreased PL secretion imply decreased SP-A secretion. However, this needs to be confirmed by direct measurement from secretions in the airways in a well-characterised population sampled under controlled conditions.

We will measure SP-A and SP-D by enzyme linked immunometric assay (ELISA) and Western blot at 0hr and 24hr sampling time points from subjects at all sites and in addition at 12hr, and 36hr from subjects at Liverpool and Southampton if still ventilated. We will compare levels over time relative to baseline, group-wise according to intervention.

No previous study has had the opportunity to relate the levels of SP-A & SP-D to surfactant metabolism during the natural history of bronchiolitis disease (placebo arm) or when modified by administration of exogenous surfactant. This proposal will evaluate not only the presence or absence of SPs but also provide evidence for their role as markers of lung recovery in the BAL.

12.2.3 Lower airway inflammation is modulated by exogenous surfactant[5]

Our previous study (3 arms, 27-plex, n=301) on local airway response to RSV bronchiolitis found high levels of IL1b, IL15, IL13, MIP1b, MCP1, IL-8 & PDGF and low levels of IFNg, IP10 & RANTES associated with life-threatening bronchiolitis (all $p<0.025$) (34). Subject to other funding, these 10 markers will be measured in BAL at t=0hr and 24hr in all subjects. Comparison will be made between baseline levels and second sample in infants treated with surfactant and placebo. Correlation will be sought between the inflammatory markers and levels of surfactant lipid and proteins.

12.2.4 Treatment efficacy or failure is associated with detection of specific pathogens.

We and others have demonstrated that certain pathogens and multiple infections are associated with poor outcome (35). Using accredited diagnostic polymerase chain reaction (PCR) multiplex assays we will assay BAL sampled daily for multiple pathogens and seek any relation with pathogens detected and outcome. While RSV infection accounts for about 75% of severe cases of

[5] Costs for this component of BESS are not being charged to NIHR EME.

For peer review only - http://bmjopen.bmj.com/site/about/guidelines.xhtml

bronchiolitis, dual viral infection or viral infection in combination with bacterial infections can account for severe disease. The aetiology of bronchiolitis in each subject must be identified to test for any pathogen specific effect.

12.2.5 Mechanistic Protocol

12.2.5.1 Surfactant phospholipid and protein concentration and composition (all sites)

Small volume lung lavage (0.9% NaCl, 1ml/kg body weight) will be performed in all participating sites at t=0, 24 just prior to trial intervention, and separated for protein and lipid analysis. For lipid analysis, butylated hydroxytoluene (10 of 20g/l in ethanol) will be added to all samples to prevent sample oxidation. Samples will be centrifuged at 400xgx10 min and the supernatant stored in aliquots for surfactant analysis at -80°C at each site. Other BAL fluid aliquots will be used for the inflammation and infection mechanistic subprojects. At the end of each winter season, all samples will be sent to Southampton for surfactant analysis.
Protein analysis will be conducted by standard techniques of ELISA and Western blotting. For PL analysis, samples will be lipid extracted in Southampton using organic solvents after addition of internal quantification standards and analysed by direct infusion ESI-MS. Analytes to be measured using diagnostic precursor scanning will include individual molecular species of PC, LysoPC, PG, PI and SM. Results will be expressed as % composition within an individual lipid class, as % composition of all measured lipids and as concentration (nmol/ml). Analysis of BAL fluid urea by colorimetric enzymatic assay will be used as a correction factor for BAL fluid dilution. While this in not ideal, a previous study indicates that it provides a reasonable correction for single recovery small volume lavage in ventilated infants (28).

Research Questions

Does administration of exogenous surfactant (poractant alfa) restore the dysfunctional composition of surfactant phospholipid characteristic of infants ventilated for bronchiolitis?
A power calculation from the pilot data at t=48h indicates that it will require n=49/group to achieve significance at p<0.05 for %DPPC of 38.8% and 43.5% respectively for infants in the placebo and treated groups. This is a realistic goal as this represents obtaining appropriate samples from 37% of prospective infants in the study
Do PC and PG concentrations in BAL fluid increase with duration of ventilation in the placebo group of infants? This analysis will also address whether the correlation between initial rate of incorporation and time to recruitment is related to secretion of labelled PC into a larger surfactant pool or decreased surfactant secretion with duration of ventilation.

12.2.5.2 Kinetics of surfactant synthesis

The mechanistic study of phosphatidylcholine surfactant synthesis will take place only at Liverpool and Southampton, which both have the appropriate infrastructure (staff and facility) to collect and process BAL more frequently. It will incorporate the phospholipid concentration and composition protocol outlined above, combined with a stable isotope labelling protocol. With specific consent recruited infants at both sites, in both placebo and treated groups, will be intravenously infused between t=0 and 1h with non-radioactive, stable isotope-labelled *methyl*D_9-choline chloride at 3.6 mg/kg. Small volume lung lavages will be collected as above, with additional time points at t=12h and 36h. EDTA blood samples (1.25ml (range 1.0 to 1.3 ml)) will be taken at the same time points and centrifuged at 400xgx10min to isolate plasma, which will be stored at -80°C. Samples will be

For peer review only - http://bmjopen.bmj.com/site/about/guidelines.xhtml

couriered from Liverpool to Southampton at the end of the winter season and sample extraction and processing will follow exactly the same protocol as above. Lipid extracts from both BAL fluid and plasma will be subjected to the identical ESI-MS analytical protocol described above, with the addition of a diagnostic scan for *methyl*-D_9-labelled PC. Enrichment of methylD9-choline in plasma choline will be analysed by LC-MS/MS using a MRM protocol to provide an indication of substrate choline turnover. Urea will be analysed by colorimetric enzymatic assay in plasma as well as in BAL fluid, to enable calculation of analyte concentrations per ml of epithelial lining fluid (ELF). Results will be calculated, in addition to those described above, as enrichment (Σ abundance of labelled PC species/Σ abundance of labelled + unlabelled PC species), fractional synthetic rates and concentration of labelled species (nmol/mL ELF).

12.2.5.3 Outcomes

The major discriminators between treatment and placebo groups will be the concentration of newly synthesised and secreted *methyl*-D_9 molecular species calculated as nmoles/ml of ELF after correction for urea dilution between BAL fluid and plasma and the FSR. Power: based on our pilot data on fractional synthetic rate of surfactant in two disease states (0.039±0.005 vs 0.030±0.011) 2 tail, a=0.05 requires total n=25/group for 1-b >0.95.
Enrichment of label in molecular species of plasma PC and in plasma choline will provide comparator data to distinguish responses that are lung specific from those determined by variability in substrate availability and metabolism.
Administration of a second dose of exogenous surfactant at t=12 hours in the treated infants will permit calculation of endogenous and exogenous surfactant pool sizes by labelled surfactant dilution.

For peer review only

For peer review only - http://bmjopen.bmj.com/site/about/guidelines.xhtml

13 REGULATORY AND ETHICAL APPROVALS

13.1 Statement of Compliance

Statement of compliance: The study will be carried out in accordance with:

- o The World Medical Association Declaration of Helsinki ,
- o CTRC Standard Operating Procedures
- o Principles of Good Clinical Practice
- o The template content is structured consistent with the SPIRIT (Standard Protocol Item: Recommendations for Interventional Trials 2013)
- o UK Policy Framework for Health and Social Care Research

SI /EU Regulation	Title	Main impact/scope
2001/20/EC	The EU Clinical Trials Directive	National Competent Authority<br>Ethics Framework<br>GCP legal requirement<br>Good Manufacturing Practice<br>Protocol/Amendments/safety<br>Protection of Vulnerable Groups<br>Consent / Data protection
2004/1031	Medicines for Human use Clinical Trials Regulation	Transposed EU CT Directive in UK
2005/28/EC	EU Good Clinical Practice (GCP) Directive	Investigator brochure<br>Archiving<br>Mandatory training for trial teams
2006/1928	Amends 2004/1031	Investigator brochure /essential documents<br>Serious Breach<br>Declaration of Helsinki 1996 version for CTIMP
2006/2984	Amends 2004/1031	Consent for incapacitated adult by legal representative or emergency deferred consent
2008/941	Amends 2004/1031	Blood safety and quality<br>Emergency Deferred consent for children
2009/1164	Miscellaneous Amendment	Urgent Safety measures
2009/3063	Amends 2004/1031	Nurse and pharmacists to prescribe unlicensed medicines

13.2 Regulatory Approval

This trial falls within the remit of the EU Directive 2001/20/EC, transposed into UK law as the UK Statutory Instrument 2004 No 1031: Medicines for Human Use (Clinical Trials) Regulations 2004 as amended.

For peer review only - http://bmjopen.bmj.com/site/about/guidelines.xhtml

BESS will be registered with the MHRA and a Clinical Trials Authorisation (CTA) will be obtained prior to the trial commencing.

13.3 Ethical Considerations

The study will abide by the principles of the World Medical Association Declaration of Helsinki.

In compliance with GCP, parents or those with parental responsibility will be fairly informed of the trial, risks, benefits, and alternatives, asked to ensure they understand how it relates to their infant, and make a voluntary decision about entry. Autonomy, privacy and welfare will be paramount, overriding the research. Parents will be kept informed of findings that might cause them to withdraw, without detriment to on-going care.

Consent in emergency setting poses particular challenges for parents. We have allowed a window of 48 hours from intubation to intervention during which to seek consent and randomisation. Dr Kerry Woolfall will lead the sub study to explore staff and parents' acceptability of our approach to consent in this challenging setting. Her work includes CONNECT study guidance, adopted by NIHR Clinical Trials Toolkit (http://www.ct-toolkit.ac.uk/routemap/informed-consent), which helps to ensure recruitment and consent to critical care trials is ethically appropriate and addresses the needs critically ill children and their families (49, 50). The trial team are experienced at conducting studies and trials in emergency settings.

The acceptability of medicines derived from pigs to families of the Jewish and Islamic faiths has been addressed with Rabbi Adler, Director of the Kashrus & Medicines Information Service; the Ashkenazi Beth Din of the United Synagogue & the Union of Orthodox Hebrew Congregations and with Dr Shafi, Chairman of the Research & Documentation Committee of the Muslim Council of Great Britain.

These religious authorities recognise that some scholars and families will have their own interpretations of their faith, as do other faiths with regards to this topic, which further complicates the issue. BESS will liaise with these authorities during the development of parent information and consent materials.

13.4 HRA Approval

BESS will come under the Health Research Authority (HRA) Approvals process as part of the initial REC submission and will follow the HRA processes for submission of amendments to REC and the HRA.

13.5 Ethical and Local Governance Approval

Prior to the trial being initiated at CTRC, a favourable ethical opinion will be obtained from a REC and global governance approval from the HRA. Prior to opening a centre to recruitment, CTRC will ensure that local governance approval has been obtained: for sites in England and Wales, this will be "Capacity & Capability" Confirmation; for sites in Scotland and Northern Ireland, this will be R&D Approval.

For peer review only - http://bmjopen.bmj.com/site/about/guidelines.xhtml

13.6 Protocol Deviation and Serious Breaches

Incidence of protocol non-compliance, whether reported prospectively (e.g. where a treatment cannot be administered at a scheduled time) or retrospectively noted (e.g. as a result of central monitoring) are recorded as protocol deviations, the incidence of which are monitored and reported to trial oversight committees.
A breach of the protocol or GCP is ‘serious’ if it meets the regulatory definition of being “likely to affect to a significant degree the safety or physical or mental integrity of the trial participants, or the scientific value of the trial”. All confirmed serious breaches of GCP or protocol will be reported to the MHRA and REC in an expedited manner by the CTRC on behalf of the sponsor.

If any persons involved in the conduct of the trial become aware of a potential serious breach, they must immediately report this to the CTRC who will in turn notify the Sponsor. The Sponsor will assess the breach and determine if it meets the criteria of a ‘serious’ breach of GCP or protocol and therefore requires expedited reporting to the MHRA and REC.

In determining whether or not the breach is likely to affect to a significant degree the safety, physical or mental integrity of participants, the Sponsor may seek advice from medical expert members of the Trial Management Group (TMG) and/or of the independent oversight committees (IDSMC and TSC). In determining whether or not the breach is likely to significantly affect the scientific value of the trial, the Sponsor may seek advice from the Trial Statistician. However, the Sponsor retains responsibility for the assessment of whether or not a breach meets the definition of ‘serious’ and is subject to expedited reporting to MHRA and REC.

Breaches confirmed as ‘serious’ will be reported to the MHRA and REC within 7 days by the CTRC and notified to the TMG, IDSMC and TSC at their next meetings.

Any requests for additional information from the Sponsor, TMG, TSC, IDSMC, REC or MHRA will be promptly actioned by the relevant member(s) of the research team and open communication will be maintained to ensure appropriate corrective actions are taken and documented.

Other incidences of protocol non-compliance will be recorded as protocol deviations, the frequency of which will be monitored and reported to the trial oversight committees.

13.7 Study Discontinuation

In the event that the BESS trial is discontinued, any participants currently participating will not be given their next dose of poractant alfa or placebo. Decisions on weaning and extubation will revert to standard care.

Reporting of adverse events will continue as per trial protocol.

For peer review only - http://bmjopen.bmj.com/site/about/guidelines.xhtml

14 DATA MANAGEMENT AND TRIAL MONITORING

Details of the monitoring to be carried out for the BESS trial are included in the BESS Trial Monitoring Plan, maintained separately to this protocol.
Trial Oversight Committees related to the monitoring of the trial are detailed in section 15.4.

14.1 Source Documents

In order to resolve possible discrepancies between information appearing in the case report form (CRF) and any other participant related documents, it is important to know what constitutes the source document and therefore the source data for all information in the CRF.

Source data is defined as: "All information in original records and certified copies of original records of clinical findings, observations, or other activities in a clinical trial necessary for the reconstruction and evaluation of the trial. Source data are contained in source documents (original records or certified copies)." (ICH E6, 1.51).

Source documents are defined as: "Original documents, data, paper and electronic records (e.g. hospital records, clinical and office charts, laboratory notes, memoranda, participant diaries or evaluation checklists, pharmacy dispensing records, recorded data from automated instruments, copies or transcriptions certified after verification as being accurate copies, microfiches, photographic negatives, microfilm or magnetic media, x-rays, participant files, and records kept at the pharmacy, at the laboratories and at medico-technical departments involved in the clinical trial)." (ICH E6, 1.52).

The CRF will be considered the source document for data where no prior record exists, and which is recorded directly in the CRF. A BESS source document list will be produced for each site.

Date(s) of conducting the informed consent process including date of provision of patient information, confirmation of full eligibility, randomisation number and the fact that the patient is participating in a clinical trial (including possible treatment arms) should be added to the patient's medical record chronologically.

14.2 Data Capture Methods

The study case report form (CRF) is the primary data collection instrument for the study. All data requested on the CRF must be recorded. All missing data must be explained.

Please ensure documents are sent by sites separate to the consent form as this has patient identifiable data on it.

The LRSQ will be completed by participants' parents at 6m and 12m after randomisation. These are completed online but paper copies will be available on request for parents without internet access. If paper questionnaires are used **sites should photocopy them** in order to retain a copy at site before mailing originals to CTRC.

BESS will also be collecting data from the Paediatric Intensive Care Audit Network (PICANet). PICANet is a secure confidential high-quality clinical database, recording details of the treatment of all critically ill children in PICUs. Collection of personally identifiable data

For peer review only - http://bmjopen.bmj.com/site/about/guidelines.xhtml

has been approved by the Patient Information Advisory Group (now the NHS Health Research Authority Confidentiality Advisory Group) and ethical approval granted by the Trent Medical Research Ethics Committee.
PICANet will provide a bespoke download page for the trial and sites will regularly download their own data for consented BESS patients only and transfer this to the CTRC.

14.3 Monitoring

14.3.1 Risk Assessment

This trial will undergo a risk assessment, completed in partnership between:

- Representative/s of the Trial Sponsor
- Chief Investigator
- CTRC Trial Coordinator and Supervising Trial Manager
- CTRC Trial Statistician and Supervising Statistician
- CTRC Information Systems and Data Management teams
- CTRC Director

In conducting the risk assessment, the contributors will consider potential participant, organisational and study hazards, the likelihood of their occurrence and resulting impact should they occur.

Monitoring for BESS will be informed by the trial specific risk assessment and will be conducted as per a detailed Trial Monitoring Plan (TMP), maintained separately to this protocol and stored in the TMF. The TMP will describe who will conduct the monitoring, at what frequency monitoring will be done and what level of detail monitoring will be conducted.

14.3.2 Central Monitoring

Data received at CTRC will be checked for missing or unusual values (range checks) and checked for consistency over time. Any suspect data will be returned to the site in the form of data queries. Data query forms will be produced at the CTRC from the trial database and sent either electronically or through the post to a named individual (as listed on the site delegation log). Sites will respond to the queries providing an explanation/resolution to the discrepancies and return the data query forms to CTRC. The forms will then be filed along with the appropriate CRFs and the appropriate corrections made on the database. There are a number of monitoring features in place at the CTRC to ensure reliability and validity of the trial data, to be detailed in the TMP maintained separately from this protocol.

Central checks of consent will be completed for each participant to ensure the completeness of consent and that the timing of consent is in line with the protocol.

Central monitoring will be performed in a proportionate manner as informed by the Trial Risk Assessment and Monitoring Plans.

For peer review only - http://bmjopen.bmj.com/site/about/guidelines.xhtml

14.3.3 Clinical Site Monitoring

In order to perform their role effectively, the Trial Coordinator, Data Manager and persons involved in Quality Assurance and Inspection may need direct access to primary data during monitoring visits, e.g. participant medical records, laboratory reports, appointment books, etc. Since this affects the participant's confidentiality, this fact is included on the PISC.

The purposes of monitoring visits, if triggered as per CTRC SOPs, include but are not limited to:

- assessing compliance with the study protocol
- discussing any emerging problems that may have been identified prior to the visit
- checking CRF and query completion practices

14.4 Confidentiality

Individual participant medical information obtained as a result of this study is considered confidential and disclosure to third parties is prohibited with the exceptions noted below.

CRFs will be pseudonymised – they will not contain details of participant names and will be labelled with the unique trial randomisation and/or screening number. Medical information may be given to the participant's medical team and all appropriate medical personnel responsible for the participant's welfare.

Verification that appropriate informed consent is obtained will be enabled by the provision of copies of participant's signed informed consent forms being supplied to the CTRC by recruiting centres, which requires that name data will be transferred to the CTRC.

The CTRC will be undertaking activities requiring the transfer of identifiable personal data. The transfer of this data is disclosed in the PISC. The CTRC will be responsible for sending links to the LRSQ to trial participants at 6m and 12m post randomisation, and therefore will be required to receive contact details including name and email address. Should the parent require a paper copy this will be sent by the recruiting site as the CTRC will not collect participant addresses.

All documentation received at CTRC which includes direct identifiers such as names and email addresses (e.g. consent forms) will be stored securely and separately to all other pseudonymised participant data (e.g. medical CRFs).

The CTRC will preserve the confidentiality of participants taking part in the study and The University of Liverpool is registered as a Data Controller with the Information Commissioners Office.

14.5 Quality Assurance (QA) and Quality Control (QC)

Quality Assurance (QA) includes all the planned and systematic actions established to ensure the trial is performed and data generated, documented, recorded and reported in compliance with applicable regulatory, ethical and governance requirements. Quality Control (QC) includes the operational techniques and activities performed within the QA system to verify that the requirements for quality of the trial-related activities are fulfilled: e.g. state what clinical site monitoring (and audit) is planned, if any. In accordance with the monitoring plan site visits will or will not be conducted and source verification performed if central monitoring processes indicate these are required. Monitoring activities could include, but are not limited to the following:

- The Trial Coordinator at the CTRC will verify appropriate approvals are in place prior to initiation of a site and the relevant personnel have attended trial specific training. A greenlight

For peer review only - http://bmjopen.bmj.com/site/about/guidelines.xhtml

checklist will verify all approvals are in place prior to trial initiation at CTRC and the individual site.

- The TMG will determine the minimum key staff required to be recorded on the delegation log in order for the site to be eligible to be initiated.
- Data will be evaluated for compliance with protocol and accuracy in relation to source documents.
- The study will be conducted in accordance with procedures identified in the protocol.
- Independent oversight of the trial will be provided by the Data and Safety Monitoring Committee and independent members of the Trial Steering Committee.
- The types of materials to be reviewed, who is responsible, and the schedule for reviews may be specified or referenced in other documents.
- Types and mechanisms of training of staff for the study should be specified.
- The PI and other key staff from each centre will attend site initiation training, coordinated by the CTRC, which will incorporate elements of trial-specific training necessary to fulfil the requirements of the protocol.
- The Trial Management Group will monitor screening, randomisation and consent rates between centres.
- The process for consent, recruitment and randomisation will be evaluated for compliance with the protocol.
- Data quality checks and monitoring procedures will be undertaken in line with the trial Data Management Plan.
-

14.6 Records Retention

All trial documents will be retained for a maximum period of 25 years from the End of Trial.

The site PI is responsible for archiving of all relevant source documents so that the trial data can be compared against source data after completion of the trial (e.g. in case of inspection from authorities). The PI at each trial site must make arrangements to store the essential trial documents, (as defined in Essential Documents for the Conduct of a Clinical Trial (ICH E6, Guideline for GCP)) including the Investigator Site File and Pharmacy Site File, until the CTRC informs him/her that the documents are no longer to be retained. The PI is required to ensure the continued storage of the documents, even if s/he, for example, leaves the clinic/practice or retires before the end of required storage period. Delegation must be documented in writing.

All other persons and organisations involved in the trial will be responsible for storing and archiving the parts of the TMF relevant to their delegated duties (e.g. laboratories, IMP manufacturers and distributors, third-party vendors providing randomisation and IMP allocation systems, etc.).

The CTRC undertakes to store originally completed CRFs for the same period, except for source documents pertaining to the individual investigational site, which are kept by the site PI only. The CTRC will archive the documents in compliance with the principles of GCP . All electronic CRFs and trial data will be archived onto an appropriate media for long term accessible storage. Hard copies of data will be boxed and transferred to secure premises where unique reference numbers are applied to enable confidentiality, tracking and retrieval.

For peer review only - http://bmjopen.bmj.com/site/about/guidelines.xhtml

15 INDEMNITY

BESS is sponsored by The University of Liverpool and co-ordinated by the CTRC in the University of Liverpool. The Sponsor does not hold insurance against claims for compensation for injury caused by participation in a clinical trial and they cannot offer any indemnity. As this is an investigator-initiated study, The Association of the British Pharmaceutical Industry (ABPI) guidelines for patient compensation by the pharmaceutical industry do not apply. However, in terms of liability, NHS Trust and Non-Trust Hospitals have a duty of care to patients treated, whether or not the patient is taking part in a clinical trial, and they are legally liable for the negligent acts and omission of their employees. Compensation is therefore available in the event of clinical negligence being proven.

The Sponsor does not accept liability for any breach in any other hospital's duty of care, or any negligence on the part of employees of hospitals. This applies whether the hospital is an NHS Trust or not.

The Sponsor has vicarious liability for the actions of its staff, when through the course of their employment they are involved in the design and initiation of a clinical trial, including but not limited to the authorship of the Clinical Trial Protocol. The University of Liverpool has appropriate insurance in place to cover this liability.

All participants will be recruited at NHS sites and therefore the NHS indemnity scheme/NHS professional indemnity will apply with respect to claims arising from harm to participants at site management organisations.

Clinical negligence is defined as:

"A breach of duty of care by members of the health care professions employed by NHS bodies or by others consequent on decisions or judgments made by members of those professions acting in their professional capacity in the course of their employment, and which are admitted as negligent by the employer or are determined as such through the legal process".

For peer review only - http://bmjopen.bmj.com/site/about/guidelines.xhtml

16 ROLES AND RESPONSIBILITIES

16.1 Role of Study Sponsor and Study Funder

The Sponsor of this trial is the University of Liverpool. The Sponsor will ensure that clear agreements are reached, documented and carried out, respecting the dignity, rights, safety and wellbeing of participants and the relationship with healthcare professionals. This will provide for proper design, management, initiation, conduct, monitoring, data collection, data analysis, data protection, financing and reporting of this trial meeting appropriate scientific, legal and regulatory standards. The responsibility for design, conduct, management, data analysis, data interpretation, manuscript writing, and dissemination of results is delegated to the Trial Management Group.

The funders of this study are the National Institute for Health Research and Medical Research Council who are providing financial funding through their Efficacy and Mechanism Evaluation Programme. The EME Programme is funded by the MRC and NIHR, with contributions from the CSO in Scotland and Health and Care Research Wales and the HSC R&D Division, Public Health Agency in Northern Ireland.

The funders will ensure there is proper use of the funds they have allocated to provide value for money. The funders assure the quality of the study, taking the lead in establishing that the research proposal is worthwhile, of high scientific quality, has an appropriate research infrastructure with expert clinical trial management, has the capacity to comply with the principles of GCP and represents good value for money.

Chiesi Farmaceutici S.p.A (Chiesi) are supplying the IMP endotracheal poractant alfa (Curosurf®) to be administered as trial IMP and will be securing use of a randomisation and re-supply system for use in the trial through an Investigator Initiated Clinical Trial Research Agreement between Chiesi and the Sponsor. Chiesi are not providing any financial funding in connection with this Agreement.

16.2 Funding and Support in Kind

Funder(s)	Financial and Non-financial Support Given
National Institute of Health Research	Financial Funding (£1,655,615.46)
Chiesi Farmaceutici S.p.A (UK address: 333 Styal Road Manchester M22 5LG)	Supply of endotracheal poractant alfa (Curosurf®), and contracting of a third party distributor who will also provide a randomisation and drug resupply system

For peer review only

For peer review only - http://bmjopen.bmj.com/site/about/guidelines.xhtml

16.3 Protocol Contributors

Name	Affiliations	Contribution to protocol
Calum Semple	Institute of Translational Medicine, University of Liverpool and Alder Hey Children's Hospital NHS Foundation Trust	Inception of study, led the writing of this protocol, clinical and scientific arrangements, trial design and conduct
Tracy Moitt	CTRC, University of Liverpool	Protocol development, governance arrangements and trial conduct
Chloe Donohue	CTRC, University of Liverpool	Protocol development, governance arrangements and trial conduct
Ashley Jones	CTRC, University of Liverpool	Led statistical arrangements, trial design and conduct
Laura Sutton	CTRC, University of Liverpool	Statistical arrangements, trial design and conduct
Kerry Woolfall	Institute of Psychology, Health & Society, University of Liverpool	Led design of parent/staff experience and consent study (WP-B)
Tony Postle	Faculty of Medicine, University of Southampton	Collaborator from inception, design and development of mechanistic study (WP-C)
Howard Clark	Faculty of Medicine, University of Southampton	Collaborator from inception, design and development of mechanistic study (WP-C)
Jens Madsen	University Hospital Southampton NHS Foundation Trust	Design and development of mechanistic study (WP-C)
John Pappachan	University Hospital Southampton NHS Foundation Trust	Design and development of mechanistic study (WP-C)
Paul Ritson	Alder Hey Children's Hospital NHS Foundation Trust	Development of BAL procedures, intervention administration/ handling, and PICU processes
Mark Turner	Institute of Translational Medicine, University of Liverpool and Liverpool Women's Hospital NHS Foundation Trust	Inception of study, clinical and scientific arrangements, trial design and liaison with pharmaceutical company
Katie Neville	CTRC, University of Liverpool	Quality Assurance review
Guido Varoli	Chiesi Farmaceutici S.p.A.	Limited to provision of information relating to IMP specifics (formulation, packaging, administration, distribution etc.)

Advice was received from all Co-Applicants to the NIHR EME funding applications and protocol submissions.

The design, conduct, data analysis, data interpretation, manuscript writing and dissemination of results are the responsibility of the TMG, subject to the approval of the University of Liverpool as Sponsor, the MHRA and National Institute for Health as Funders.
Chiesi Farmaceutici S.p.A. are responsible for supplying the IMP and subcontracting an independent third party to undertake distribution of the IMP and development, management and maintenance of a randomisation and drug resupply system.

For peer review only - http://bmjopen.bmj.com/site/about/guidelines.xhtml

16.4 Trial Committees

16.4.1 Trial Management Group (TMG)

A Trial Management Group (TMG) will be formed comprising the Chief Investigator, other lead investigators (clinical and non-clinical), members of the CTRC and a lay member. The TMG will be responsible for the day-to-day running and management of the trial. Refer to the TMG terms of reference and trial oversight committee membership document for further details. Meetings will be in Liverpool with teleconferencing. The TMG will report to the TSC.

16.4.2 Trial Steering Committee (TSC)

The TSC will consist of an independent medical expert chairperson (neonatology), two independent medical experts (paediatric intensive care), an independent biostatistician and two lay members (one independent, one non-independent). TMG representatives will be given the opportunity to contribute as non-independent and non-voting TSC members. The role of the TSC is to provide overall supervision for the trial and provide advice through its independent Chairman. The ultimate decision for the continuation of the trial lies with the TSC.

The TSC will first convene prior to the start of recruitment and will then define frequency of subsequent meetings (at least annually). Refer to the TSC terms of reference and trial oversight committee membership document for further details.

16.4.3 Independent Data and Safety Monitoring Committee (IDSMC)

The IDSMC consists of an independent medical expert chairperson (neonatology), an independent expert in Paediatric Intensive Care and an independent expert in medical statistics.

The IDSMC will be responsible for reviewing and assessing recruitment, interim monitoring of safety and effectiveness, trial conduct and external data. The IDSMC will first convene prior to the start of recruitment and will then define frequency of subsequent meetings (at least annually). Details of the interim analyses and monitoring are provided in section 11.4 .

The IDSMC will provide a recommendation to the TSC concerning the continuation of the study. Refer to the IDSMC charter and trial oversight committee membership document for further details.

For peer review only

For peer review only - http://bmjopen.bmj.com/site/about/guidelines.xhtml

17 PUBLICATION AND DISSEMINATION

17.1 Publication Policy

The results from different centres will be analysed together and published as soon as possible. The design, conduct, data analysis, data interpretation, manuscript writing, and dissemination of results are the responsibility of the TMG on behalf of the University of Liverpool as Sponsor and the National Institute for Health as Funders.

The TMG will form the basis of the Writing Committee and advise on the nature of publications. The Uniform Requirements for Manuscripts Submitted to Biomedical Journals (http://www.icmje.org/) will be respected. The National Clinical Trial (NCT) identifier allocated to this trial will be attached to any publications resulting from this trial.

The members of the TSC and IDSMC will be listed with their affiliations in the Acknowledgements / Appendix of the main publication.

17.1.1 Authorship

Contributors to all four of (i) the design, conduct, data analysis and interpretation, (ii) writing, (iii) manuscript approval and (iv) accountability for the integrity of the work will, depending on their contribution and journal requirements, be included by name at the manuscript head or listed at the end in a by-line as members of the BESS Consortium which will also be named at the manuscript head.

17.2 Dissemination to Key Stakeholders

BESS intends to publish open access results in high impact journals (e.g. British Medical Journal, New England Journal of Medicine, Lancet, American Journal of Respiratory and Critical Care Medicine). The full trial report will also be published in the NIHR EME Journal.
BESS will also present at international meeting of the major Paediatric Intensive Care Societies, American Thoracic Society, European Respiratory Society.

The involvement of representatives of the UK Paediatric Intensive Care Society Study Group (PICSSG) as co-applicants will be key to ensuring dissemination of result to the Paediatric Intensive Care community.

At end of study, we will organise a symposium in partnership with the UK Paediatric Intensive Care Society where the results of the study will be presented alongside reports of family experiences as reported directly by parents. The symposium will be open to intensivists, respiratory physicians, study staff and any interest participating families.
We also intend to disseminate via a PPI event to be hosted in Liverpool at end of study with targeted open invitation to all families of participants.

We anticipate that because of the nature of the study population and the disease that our findings will be of interest to the general public. For this reason we will be proactive in targeting the broadcast media and press through our professional external media consultants, with due regard to the usual NIHR publicity and publication conditions.

For peer review only - http://bmjopen.bmj.com/site/about/guidelines.xhtml

Public dissemination will include contemporary social media including a study website and Twitter account.

17.1 Data Sharing

Individual Clinicians must undertake not to submit any part of their individual data for publication without the prior consent of the TMG. All publications shall include a list of participating PIs and collaborators.

For peer review only

DISCLAIMER:

The views expressed in this publication are those of the author(s) and not necessarily those of the MRC, NHS, NIHR or the Department of Health and Social Care.

For peer review only - http://bmjopen.bmj.com/site/about/guidelines.xhtml

18 CHRONOLOGY OF PROTOCOL AMENDMENTS

18.1 Version 1.0 (18/07/2018)

Original signed version. Not approved by MHRA.

18.2 Version 2.0 (28/09/2018)

**Summary of Amendments from Protocol V1.0 to V2.0**		
**Page Number**	**Section**	**Amendment**
Page 1	N/A	Research Ethics reference number added as now known.
Page 14	2.0	A known hypersensitivity to the active substance or any excipients of Curosurf® added to exclusion criteria.
Page 28	6.2	A known hypersensitivity to the active substance or any excipients of Curosurf® added to exclusion criteria.
Page 32	7.3	Randomisation procedure updated to include completion of unblinding envelope
Page 34	8.1.1	Reference to physical examination added
Page 35	8.1.3	Reference to physical examination added
Page 37	8.3	Schedule for physical examinations added to intervention & follow-up schedule (Table 2)
Page 41	8.5	Safety Assessment procedures updated to include physical examinations and recording of vital signs; new sub section 8.5.1 added to detail procedure for limited physical examination
Page 50-51	9.4	Detail added to unblinding section
Page 51	9.4.1	Emergency unblinding process updated to include use of unblinding envelopes
Page 53	10.5	Reference Safety Information updated from sections 4.3 to 4.9 of the Curosurf® SmPC to section 4.8 only.
Page 77	16.1	Full NIHR EME funding statement added.
Page 82	N/A	NIHR EME disclaimer added to bottom of page.

For peer review only - http://bmjopen.bmj.com/site/about/guidelines.xhtml

19 REFERENCES

1. Nair H, Nokes DJ, Gessner BD, Dherani M, Madhi SA, Singleton RJ, et al. Global burden of acute lower respiratory infections due to respiratory syncytial virus in young children: a systematic review and meta-analysis. Lancet. 2010;375(9725):1545-55.

2. Green CA, Yeates D, Goldacre A, Sande C, Parslow RC, McShane P, et al. Admission to hospital for bronchiolitis in England: trends over five decades, geographical variation and association with perinatal characteristics and subsequent asthma. Arch Dis Child. 2016;101(2):140-6.

3. Jat KR, Chawla D. Surfactant therapy for bronchiolitis in critically ill infants. Cochrane Database Syst Rev. 2015(8):CD009194.

4. Luchetti M, Casiraghi G, Valsecchi R, Galassini E, Marraro G. Porcine-derived surfactant treatment of severe bronchiolitis. Acta Anaesthesiol Scand. 1998;42(7):805-10.

5. Luchetti M, Ferrero F, Gallini C, Natale A, Pigna A, Tortorolo L, et al. Multicenter, randomized, controlled study of porcine surfactant in severe respiratory syncytial virus-induced respiratory failure. Pediatr Crit Care Med. 2002;3(3):261-8.

6. Tibby SM, Hatherill M, Wright SM, Wilson P, Postle AD, Murdoch IA. Exogenous surfactant supplementation in infants with respiratory syncytial virus bronchiolitis. Am J Respir Crit Care Med. 2000;162(4 Pt 1):1251-6.

7. McNamara PS, Ritson P, Selby A*, et al* Bronchoalveolar lavage cellularity in infants with severe respiratory syncytial virus bronchiolitis. Archives of Disease in Childhood. 2003;88**:**922-926.

8. von Neergaard K. Neue Auffassungen übereinen Grundbegriff der Atemmechanik. Die Retraktionskraft der Lunge, abhängig von der Oberfl ächenspannung in den Alveolen. (New views on a fundamental concept of respiratory mechanics. The retraction force of the lung is dependent on the surface tension within the alveoli.). Z Gesamt Exp Med 1929;66:373-94.

9. Goh BC, Rynkiewicz MJ, Cafarella TR, White MR, Hartshorn KL, Allen K, et al. Molecular mechanisms of inhibition of influenza by surfactant protein D revealed by large-scale molecular dynamics simulation. Biochemistry. 2013;52(47):8527-38.

10. LeVine AM, Lotze A, Stanley S, Stroud C, O'Donnell R, Whitsett J, et al. Surfactant content in children with inflammatory lung disease. Crit Care Med. 1996;24(6):1062-7.

11. Dargaville PA, South M, McDougall PN. Surfactant abnormalities in infants with severe viral bronchiolitis. Arch Dis Child. 1996;75(2):133-6.

12. Skelton R, Holland P, Darowski M, Chetcuti PA, Morgan LW, Harwood JL. Abnormal surfactant composition and activity in severe bronchiolitis. Acta Paediatr. 1999;88(9):942-6.

13. Kerr MH, Paton JY. Surfactant protein levels in severe respiratory syncytial virus infection. Am J Respir Crit Care Med. 1999;159(4 Pt 1):1115-8.

14. Hartmann F, Fiori HH, Ramos Garcia PC, Piva J, Fiori RM. Surfactant deficiency in infants with severe acute viral bronchiolitis. J Pediatr. 2014;164(6):1432-5.

15. Kelly E, Bryan H, Possmayer F, Frndova H, Bryan C. Compliance of the respiratory system in newborn infants pre- and postsurfactant replacement therapy. Pediatr Pulmonol. 1993;15(4):225-30.

For peer review only - http://bmjopen.bmj.com/site/about/guidelines.xhtml

16. Jat KR, Chawla D. Surfactant therapy for bronchiolitis in critically ill infants. Cochrane Database Syst Rev. 2012(9):CD009194.
17. NICE. Bronchiolitis in children: diagnosis and management (NG9). 2015.
18. Siegel LS. Correction for prematurity and its consequences for the assessment of the very low birth weight infant. Child Dev. 1983;54(5):1176-88.
19. Soll RF. Surfactant treatment of the very preterm infant. Biol Neonate. 1998;74 Suppl 1:35-42.
20. Powell CV, McNamara P, Solis A, Shaw NJ. A parent completed questionnaire to describe the patterns of wheezing and other respiratory symptoms in infants and preschool children. Arch Dis Child. 2002;87(5):376-9.
21. Paret G, Ziv T, Barzilai A, Ben-Abraham R, Vardi A, Manisterski Y, et al. Ventilation index and outcome in children with acute respiratory distress syndrome. Pediatr Pulmonol. 1998;26(2):125-8.
22. Bilan N, Dastranji A, Ghalehgolab Behbahani A. Comparison of the spo2/fio2 ratio and the pao2/fio2 ratio in patients with acute lung injury or acute respiratory distress syndrome. J Cardiovasc Thorac Res. 2015;7(1):28-31.
23. Wolfe R, Carlin JB. Sample-Size Calculation for a Log-Transformed Outcome Measure. Controlled Clinical Trials. 1999;20(6):547-54.
24. Menon K, Ward RE, Gaboury I, Thomas M, Joffe A, Burns K, et al. Factors affecting consent in pediatric critical care research. Intensive Care Med. 2012;38(1):153-9.
25. Kerrin A, Fitch P, Errington C, Kerr D, Waxman L, Riding K, et al. Differential lower airway dendritic cell patterns may reveal distinct endotypes of RSV bronchiolitis. Thorax. 2016.
26. McNamara PS, Fonceca AM, Howarth D, Correia JB, Slupsky JR, Trinick RE, et al. Respiratory syncytial virus infection of airway epithelial cells, in vivo and in vitro, supports pulmonary antibody responses by inducing expression of the B cell differentiation factor BAFF. Thorax. 2013;68(1):76-81.
27. McNamara PS, Ritson P, Selby A, Hart CA, Smyth RL. Bronchoalveolar lavage cellularity in infants with severe respiratory syncytial virus bronchiolitis. Arch Dis Child. 2003;88(10):922-6.
28. Semple MG, Dankert HM, Ebrahimi B, Correia JB, Booth JA, Stewart JP, et al. Severe respiratory syncytial virus bronchiolitis in infants is associated with reduced airway interferon gamma and substance P. PLoS One. 2007;2(10):e1038.
29. Bernhard W, Pynn CJ, Jaworski A, Rau GA, Hohlfeld JM, Freihorst J, et al. Mass spectrometric analysis of surfactant metabolism in human volunteers using deuteriated choline. Am J Respir Crit Care Med. 2004;170(1):54-8.
30. Dushianthan A, Goss V, Cusack R, Grocott MP, Postle AD. Altered molecular specificity of surfactant phosphatidycholine synthesis in patients with acute respiratory distress syndrome. Respir Res. 2014;15:128.
31. Bunt JE, Zimmermann LJ, Wattimena JL, van Beek RH, Sauer PJ, Carnielli VP. Endogenous surfactant turnover in preterm infants measured with stable isotopes. Am J Respir Crit Care Med. 1998;157(3 Pt 1):810-4.

For peer review only - http://bmjopen.bmj.com/site/about/guidelines.xhtml

32. Greene KE, Wright JR, Steinberg KP, Ruzinski JT, Caldwell E, Wong WB, et al. Serial changes in surfactant-associated proteins in lung and serum before and after onset of ARDS. Am J Respir Crit Care Med. 1999;160(6):1843-50.
33. Todd DA, Marsh MJ, George A, Henderson NG, Barr H, Sebastian S, et al. Surfactant phospholipids, surfactant proteins, and inflammatory markers during acute lung injury in children. Pediatr Crit Care Med. 2010;11(1):82-91.
34. Semple MG, Flanagan B, Smyth RL. Nasal Cytokines Associated With Householder Tobacco Smoking And HRSV Bronchiolitis. American Journal of Respiratory and Critical Care Medicine; December 2012: American Thoracic Society; 2012. p. A1026-A.
35. Semple MG, Cowell A, Dove W, Greensill J, McNamara PS, Halfhide C, et al. Dual infection of infants by human metapneumovirus and human respiratory syncytial virus is strongly associated with severe bronchiolitis. J Infect Dis. 2005;191(3):382-6.
36. Wright SM, Hockey PM, Enhorning G, Strong P, Reid KB, Holgate ST, et al. Altered airway surfactant phospholipid composition and reduced lung function in asthma. J Appl Physiol (1985). 2000;89(4):1283-92.
37. Postle AD, Heeley EL, Wilton DC. A comparison of the molecular species compositions of mammalian lung surfactant phospholipids. Comp Biochem Physiol A Mol Integr Physiol. 2001;129(1):65-73.
38. Mander A, Langton-Hewer S, Bernhard W, Warner JO, Postle AD. Altered phospholipid composition and aggregate structure of lung surfactant is associated with impaired lung function in young children with respiratory infections. Am J Respir Cell Mol Biol. 2002;27(6):714-21.
39. Wright JR. Immunoregulatory functions of surfactant proteins. Nat Rev Immunol. 2005;5(1):58-68.
40. LeVine AM, Gwozdz J, Stark J, Bruno M, Whitsett J, Korfhagen T. Surfactant protein-A enhances respiratory syncytial virus clearance in vivo. J Clin Invest. 1999;103(7):1015-21.
41. Rawat M, Chadrasekharan PK, Wiliams A, Gugino S, Koenigsknecht C, Swartz D, Ma CX, Mathew B, Nair J, Lakshminrusimha S. Oxygen Saturation Index and Severity of Hypoxic Respiratory Failure. Neonatology. 2015; 107: 161-166
42. BMJ 2015;350:h2305 [doi: https://doi.org/10.1136/bmj.h2305]

For peer review only - http://bmjopen.bmj.com/site/about/guidelines.xhtml

20 DOCUMENTS SUPPLEMENTARY TO THE PROTOCOL

Documents referenced herein as accompanying the protocol that are separately updated and version controlled are:

Patient information sheets and consent form
GP Letter
LRSQ questionnaires
Participating sites log
Trial Monitoring Plan
Statistical Analysis Plan
Trial Risk Assessment

For peer review only

For peer review only - http://bmjopen.bmj.com/site/about/guidelines.xhtml

Parent Interview Topic Guide
The **B**ronchiolitis **E**ndotracheal **S**urfactant **S**tudy (BESS)

Please note: *Italic text indicates instruction for researcher and will not be read to participant*

Intro: My name is [*researcher name*] and I am a researcher from the University of Liverpool. Many thanks for agreeing to speak to us about your child's hospital admission. We know that this must have been a difficult time and the purpose of this conversation is to get your feedback so we can insure that future parents ….. <br>***Check consent for audio recording*** <br>You can stop the interview at any time. Before we start do you have any questions? <br>**Bereaved: I have some idea about your circumstances, if there is anything that you find difficult to talk about please don't feel that you have to, or if you want to stop the interview at any point, then please let me know.** <br>**The reason that you were invited to take part in this interview study is because if we only ask those parents whose children recovered from bronchiolitis the information we gather won't be complete. The findings will be biased. We know even less about what it is like for parents who have been bereaved to have discussions with doctors and nurses about the trial and what it was like to be involved in the trial. Does that make sense? Do you have any questions before I start? I will start with some questions about you if that's ok and then I will ask you about your experience of being invited to take part in BESS?**

**Section 1: Background**	
1.1	Demographics: Could I please ask you to tell me a few things about yourself and your family? What is your DOB? What is your occupation? What is the first part of post code, What would you describe as being your ethnic background, What is your religion (if any)? How many children do you have? *If not known:* Please tell me a little bit about your child (*name, age, gender*) Was your child born at full term or were they premature (If prem ask how many weeks gestation when born)

	**Not bereaved**<br><br>Has he/she recovered from his/her hospital visit? Has your child (any of your children) had any previous hospital admissions for bronchiolitis?
1.2	Could you give me an outline of what happened for them to need emergency treatment for bronchiolitis? *(if bereaved say:* I know this may be difficult, if you don't wish to answer this question please say and I will go on to the next question)<br><br>When were they admitted to hospital with bronchiolitis? *Prompt: which hospital* (prompt- explore if transferred)<br>How long after you arrived at hospital was your child transferred to the PICU? How long after they arrived at hospital/ PICU where they put on a ventilator? *(Prompt: explore if also received any forms of non-invasive ventilation: e.g. nasal CPAP and nasal high low oxygen and when)*
1.3	Before your admission, had you ever heard of bronchiolitis before? At what point where you told that your child had bronchiolitis? What did you think when you first heard that your child had bronchiolitis?<br>Did anyone explain to you about this disease, such as how long a child with bronchiolitis is usually sick for? (*Prompt: If yes explore what information parents received. If no, would you have liked a doctor or nurse to have provided you with information about bronchiolitis? BOTH Yes and NO- Explore what information they would have liked to have known about*)<br>Did anyone explain how your child would be cared for? (*Prompt: Explore any discussion of treatments)*
**Section 2: BESS recruitment and consent process**	
2.1	This is a question I ask all parents and it's not a test, but just so we can gage whether the trial is being explained clearly enough I wanted to know whether you;<br><br>Could you tell me what the BESS trial was looking at?
2.2	Please could you give me an overall picture of what happened when you first heard about the BESS trial… could you tell me a bit about that?<br><br>*Explore any knowledge about the trial before admission.*<br><br>*Explore where parents were/ when they first heard the trial mentioned.*<br><br>*Explore time frame- how long where you approached after your child was put on a ventilator?*
2.3	How / where were you introduced to the study? *(prompt hospital staff or PICU transport staff)*<br><br>Was it a doctor or nurse who spoke to you about the trial?

For peer review only

	Did one of the nursing staff looking after your child introduce you to the research nurse or doctor?
2.4	Did the research nurse/doctor check with you that it was a good time to talk about research?<br><br>If so, when was this? Do you think that this was the best time?<br><br>If not, when would have been the best time?<br><br>How could this be improved?
2.5	Could you tell me what they explained about BESS?<br><br>Did they go through any of the potential risks or benefits of your child taking part in the trial at that point? If yes, how did they describe these?<br><br>Was there anything that you found: a) unclear b) surprising?<br><br>Is there anything else that sticks out in your mind about the discussion? *(Explore)*
2.6	Could you tell me about any written information you were given by a nurse or doctor about the trial?<br><br>Explore whether they were given the information leaflet – short version and/or the full patient information sheet<br><br>When did you receive this information? *(Prompt: explore written and verbal and time point)*<br><br>Did you read the information leaflet/sheet?<br><br>(*Prompt:* If they read the short information PIS leaflet or the full information PIS)<br><br>What did you think about the information leaflet/sheet?<br><br>Was there anything that you found: a) unclear? b) surprising?<br><br>Did you have any concerns about the nature of the treatment involved *(e.g. surfactant comes from pigs lungs) Explore any concerns related to religious beliefs. Explore if the nature of the treatment influenced decision making or willingness to provide consent.*<br><br>Could the information leaflet be improved in any way?
2.7	How long did you get to think about whether you wanted your child's information to be used in BESS? Do you think this was long enough?<br><br>Did you have the opportunity to ask any other questions about the study?<br><br>Did you ask any? *(Prompt, what questions did you ask?, if not, why not)*<br><br>How long do you think people should be given to think about taking part in BESS?
2.9	Did you give consent for your child to be in the trial?<br><br>Would you mind telling me what were your reasons for (providing consent/not providing consent)?

For peer review only

	In making the decision about your child's participation in BESS, what sort of things went through your mind?<br><br>Was there anything you found particularly helpful in making up your mind? Prompt: did you discuss the trial with anyone?<br><br>How hard/ easy was this decision?<br><br>Was there anything specific that you have not already mentioned that influenced your decision?<br><br>Have you / your child taken part in medical research before?
2.10	*In BESS and most research consent is obtained before children are entered into a study. In some trials conducted in emergency situations parents are asked to provide consent after their child had already been actively involved in the Trial. This was to use the data already collected and for them to continue in the trial. This is known as research without prior consent or deferred consent.*<br><br>**Have you ever heard of research without prior consent before?**<br><br>*The reason that this legislation is in place is because in situations like this (ie, in paediatric critical care), there's a belief that there's no time to have a full discussion and time to reflect about the research and that actually having that discussion might delay treatment.*<br><br>**What do you think about the use of RWPC in critical care research?**<br><br>**Do you think the BESS consent process should stay the same as you experienced, or would you suggest that we move to a RWPC approach in BESS?**<br><br>*Explore parents views on the pros and cons of each approach for BESS and reasons behind their views*
2.11	**If parent supports informed consent**: do you have any suggestions about how we could improve the consent process in the future?
2.12	If parents consented:<br><br>Where you present when the BESS syringe of liquid or air was given to your child? If yes, Could you tell me a little bit about that?<br><br>How did you feel at that point in time*? (If concerns described then explore- did you voice your concerns to a nurse or doctor)*

Interview end- thank you for your time

Inform participant that their voucher and copy of the consent form will be sent via post. Check home address is on record from registration.

For peer review only

BESS
Bronchiolitis Endotracheal Surfactant Study

BESS Study team
IPHS
Block B
1st Floor Waterhouse Building
Liverpool L69 3GL

BESS Site Staff Questionnaire

The aim of this short questionnaire is to find out how well you think BESS has been going at your site. This includes what you think is working well and perhaps what isn't working so well. We will use these findings, alongside data collected from parents to inform season two of BESS.

Please note that by returning this survey you are giving permission for your responses to be included in the BESS trial. All information will be anonymised and stored securely in compliance with the Data Protection Act 1998.

Background information

1. Please identify your hospital (ADD list)

2. What is your current role at this hospital?

   Doctor / nurse / Other (please state) ____

3. How many years' experience do you have in recruiting to paediatric clinical trials?

   __________ years

4. Are you involved in clinical care of children?

   Yes / No

5. How have you been involved in the BESS study (please tick all that apply)

   Screening patients

   Randomisation

   Consenting

   Involved in the clinical care of BESS participants

   Other (please state) ____

Training

6. Who provided your BESS training (please tick all that apply)

Trial team (e.g. including BESS trial coordinator)

Member of your site team

Other (please state) ____________________________________

7. Overall how would you rate the BESS site training **from the trial team**?

Excellent/ Good/ Fair/ Poor / Not applicable (did not attend)

8. Overall how would you rate the BESS training **from your site team**?

Excellent/ Good/ Fair/ Poor / Not applicable (did not attend)

9. Do you think the BESS site training could be improved? Yes/No

If yes, how? ____

____

Process

10. Do you think the screening process could be improved? (Please circle one answer)

Yes / No

If yes, please elaborate:

____

____

11. Do you think the randomisation process could be improved?

Yes / No

If yes, please elaborate:

____

____

12. Have you experienced any difficulties in adhering to the protocol?

Yes / No

If yes, please elaborate:

____

____

13. How acceptable did you find administering the intervention (surfactant) to patients?

Very acceptable / acceptable / not acceptable / very unacceptable / Not applicable

14. How acceptable did you find administering the placebo (sham) to patients?

Very acceptable / acceptable / not acceptable / very unacceptable / Not applicable

Consent and comments

15. How acceptable did you find seeking informed consent before randomisation in BESS?

Very acceptable / Acceptable / Not acceptable / Very unacceptable / Not applicable

Please explain your answer:

____

____

16. How have parents responded to the BESS consent discussion?

Very positively/ Positively/ Negatively/ Very negatively/ Not applicable.

Please explain your answer____

____

____

17. Did you find the case report form easy to use?

Yes / No/Not applicable

If No, Please provide and suggestions for improving the case report form

____

____

18. Did you find the consent form easy to use?

Yes / No/Not applicable

If No, Please provide and suggestions for improving the consent form

____

____

19. Please use this space to tell us anything you think is important for us to know to inform season 2 in BESS:

Thank you for taking the time to complete this questionnaire.

BESS
Bronchiolitis Endotracheal Surfactant Study

NHS
National Institute for Health Research

Clinician to enter Trial number (if applicable): ☐☐ ☐☐

Parent Questionnaire

The **B**ronchiolitis **E**ndotracheal **S**urfactant **S**tudy (BESS)

ONE QUESTIONNAIRE FOR EACH PARENT PRESENT

Background

- The following questions are about the BESS trial consent discussion that you took part in.
- We refer to people agreeing to take part in research as 'consenting'.

Completing this questionnaire

Today's date ☐☐/☐☐/☐☐☐☐

Are you the child's ◯ Mother ◯ Father ◯ Other *(Please specify)* ____________

Your child's date of birth __ __/ __ __/ __ __

For peer review only - http://bmjopen.bmj.com/site/about/guidelines.xhtml

1. *Please indicate how strongly you agree or disagree with the following statements by placing a circle around the answer that best fits your opinion*

Statements	Agree	Neither agree nor disagree	Disagree
a. The doctor or nurse checked that it was a convenient time to discuss research before discussing BESS	1	2	3
b. The information I received about BESS was clear and straightforward to understand	1	2	3
c. I had enough opportunity to ask questions about BESS	1	2	3
d. I was satisfied with the consent process for BESS	1	2	3
e. It was difficult to take in the information I was given about BESS	1	2	3
f. It was difficult to make a decision about BESS	1	2	3
g. I made this decision	1	2	3
h. Someone took this decision away from me	1	2	3
i. I was not in control of this decision	1	2	3
j. The decision about the research was inappropriately influenced by others	1	2	3

If the answer to this question is 'Agree', please state who you think influenced the decision about the research:

____

For peer review only

2. *Did you consent for your child to participate in BESS?*

◯ Yes (Go to Question 3) ◯ No (Go to Question 4)

3. *What were your reasons for providing consent for your child to participate in BESS?*

Please tick all that apply <u>and then circle your main reason</u> (e.g. ✔)

- a. To help my child ☐
- b. To help other children in the future ☐
- c. I felt that medical studies like BESS are important ☐
- d. Because I trusted the doctor or nurse who explained BESS ☐
- e. I didn't feel comfortable saying no to the nurse or doctor who explained the study ☐
- f. Other (Please state): ☐

____

4. *If you did not provide consent, please provide your reasons for deciding that your child would not take part in BESS*
(If you do not wish to do so, please leave this space blank)

5. *Please tell us any comments or suggestions you have to improve the recruitment and consent process for BESS:*

We would like to thank you for taking the time to complete this questionnaire.

Please place the questionnaire in the envelope provided, seal it and give it back to the doctor or research nurse.

For peer review only

Approach to data analysis

Phase	Description
1. Familiarising with data	ED read and re-read transcripts noting down initial ideas on themes.
2. Generating initial codes	Initially ED developed a data-coding framework using *a priori* codes identified from the project proposal, topic guilds and past relevant studies conducted by the team. Additional data-driven codes and concepts not previously captured in the initial coding frame were also added.
3. Developing the coding framework	KW coded 16% of the interview transcripts using the initial coding frame and no new themes were identified.
4. Defining and naming themes	Following review by ED and KW coding frames were subsequently developed and ordered into themes (nodes) within the *NVivo* Database.
5. Completion of coding of transcripts	ED completed coding interview transcripts in preparation for write-up.
6. Quantitative data analysis	Descriptive statistics were conducted on questionnaire and survey data.
7. Data synthesis and producing the report	ED and KW synthesised qualitative and qualitative data with reference to the adapted Theoretical Framework of Acceptability using a constant comparative approach (Deja et al 2020). This involved using themes to relate back to the study aims ensuring key findings and recommendations were relevant to the BESS trial design (i.e. catalytic validity). Final discussion and development of selected themes occurred during the write-up phase.

For peer review only

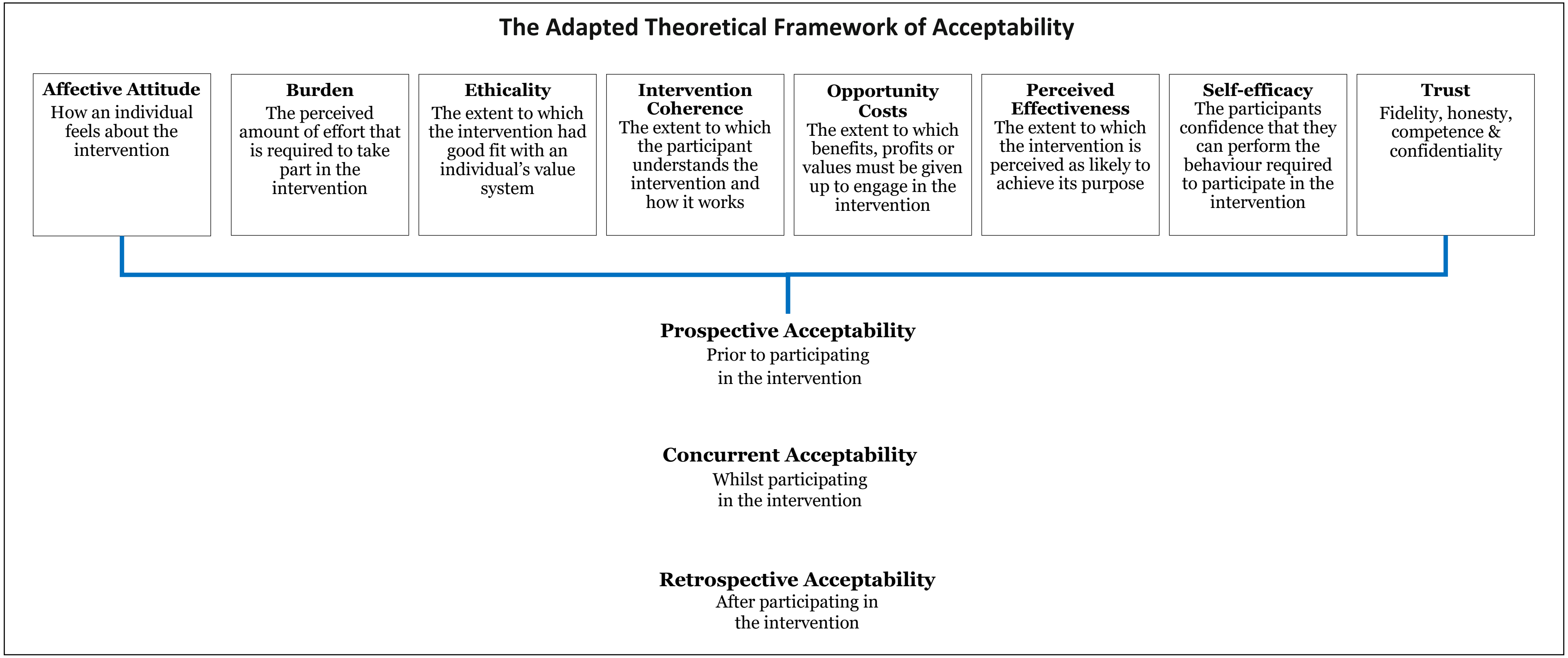

Deja E, Peters MJ, Khan I, Mouncey PR, Agbeko R, Fenn B, et al (2021). Establishing and augmenting views on the acceptability of a paediatric critical care randomised controlled trial (the FEVER trial): a mixed methods study. BMJ Open. 11(3):e041952.

COREQ (COnsolidated criteria for REporting Qualitative research) Checklist

A checklist of items that should be included in reports of qualitative research. You must report the page number in your manuscript where you consider each of the items listed in this checklist. If you have not included this information, either revise your manuscript accordingly before submitting or note N/A.

Topic	Item No.	Guide Questions/Description	Reported on Page No.
**Domain 1: Research team and reflexivity**			
Personal characteristics			
Interviewer/facilitator	1	Which author/s conducted the interview or focus group?	
Credentials	2	What were the researcher's credentials? E.g. PhD, MD	
Occupation	3	What was their occupation at the time of the study?	
Gender	4	Was the researcher male or female?	
Experience and training	5	What experience or training did the researcher have?	
Relationship with participants			
Relationship established	6	Was a relationship established prior to study commencement?	
Participant knowledge of the interviewer	7	What did the participants know about the researcher? e.g. personal goals, reasons for doing the research	
Interviewer characteristics	8	What characteristics were reported about the inter viewer/facilitator? e.g. Bias, assumptions, reasons and interests in the research topic	
**Domain 2: Study design**			
Theoretical framework			
Methodological orientation and Theory	9	What methodological orientation was stated to underpin the study? e.g. grounded theory, discourse analysis, ethnography, phenomenology, content analysis	
Participant selection			
Sampling	10	How were participants selected? e.g. purposive, convenience, consecutive, snowball	
Method of approach	11	How were participants approached? e.g. face-to-face, telephone, mail, email	
Sample size	12	How many participants were in the study?	
Non-participation	13	How many people refused to participate or dropped out? Reasons?	
Setting			
Setting of data collection	14	Where was the data collected? e.g. home, clinic, workplace	
Presence of non-participants	15	Was anyone else present besides the participants and researchers?	
Description of sample	16	What are the important characteristics of the sample? e.g. demographic data, date	
Data collection			
Interview guide	17	Were questions, prompts, guides provided by the authors? Was it pilot tested?	
Repeat interviews	18	Were repeat inter views carried out? If yes, how many?	
Audio/visual recording	19	Did the research use audio or visual recording to collect the data?	
Field notes	20	Were field notes made during and/or after the inter view or focus group?	
Duration	21	What was the duration of the inter views or focus group?	
Data saturation	22	Was data saturation discussed?	
Transcripts returned	23	Were transcripts returned to participants for comment and/or	

For peer review only

Topic	Item No.	Guide Questions/Description	Reported on Page No.
		correction?	
**Domain 3: analysis and findings**			
Data analysis			
Number of data coders	24	How many data coders coded the data?	
Description of the coding tree	25	Did authors provide a description of the coding tree?	
Derivation of themes	26	Were themes identified in advance or derived from the data?	
Software	27	What software, if applicable, was used to manage the data?	
Participant checking	28	Did participants provide feedback on the findings?	
Reporting			
Quotations presented	29	Were participant quotations presented to illustrate the themes/findings? Was each quotation identified? e.g. participant number	
Data and findings consistent	30	Was there consistency between the data presented and the findings?	
Clarity of major themes	31	Were major themes clearly presented in the findings?	
Clarity of minor themes	32	Is there a description of diverse cases or discussion of minor themes?	

Developed from: Tong A, Sainsbury P, Craig J. Consolidated criteria for reporting qualitative research (COREQ): a 32-item checklist for interviews and focus groups. *International Journal for Quality in Health Care*. 2007. Volume 19, Number 6: pp. 349 – 357

Once you have completed this checklist, please save a copy and upload it as part of your submission. DO NOT include this checklist as part of the main manuscript document. It must be uploaded as a separate file.

For peer review only

BMJ Open

Stakeholders' perspectives on clinical trial acceptability and approach to consent within a limited timeframe: a mixed methods study.

Journal:	*BMJ Open*
Manuscript ID	bmjopen-2023-077023.R2
Article Type:	Original research
Date Submitted by the Author:	17-Nov-2023

Complete List of Authors:	Deja, Elizabeth; University of Liverpool, Department of Public Health, Policy and Systems<br>Donohue, Chloe; University of Liverpool, Liverpool Clinical Trials Centre<br>Semple, Malcolm; University of Liverpool; Alder Hey Children's Hospital, Respiratory Medicine<br>Woolfall, Kerry; University of Liverpool, Institute of Population, Health and Society<br>Investigators, BESS ; Multiple

**Primary Subject Heading**:	Paediatrics
Secondary Subject Heading:	Intensive care, Paediatrics, Qualitative research, Respiratory medicine, Ethics
Keywords:	QUALITATIVE RESEARCH, ETHICS (see Medical Ethics), Feasibility Studies, Paediatric intensive & critical care < INTENSIVE & CRITICAL CARE, Randomized Controlled Trial, Respiratory infections < THORACIC MEDICINE

SCHOLARONE™
Manuscripts

I, the Submitting Author has the right to grant and does grant on behalf of all authors of the Work (as defined in the below author licence), an exclusive licence and/or a non-exclusive licence for contributions from authors who are: i) UK Crown employees; ii) where BMJ has agreed a CC-BY licence shall apply, and/or iii) in accordance with the terms applicable for US Federal Government officers or employees acting as part of their official duties; on a worldwide, perpetual, irrevocable, royalty-free basis to BMJ Publishing Group Ltd ("BMJ") its licensees and where the relevant Journal is co-owned by BMJ to the co-owners of the Journal, to publish the Work in this journal and any other BMJ products and to exploit all rights, as set out in our licence.

The Submitting Author accepts and understands that any supply made under these terms is made by BMJ to the Submitting Author unless you are acting as an employee on behalf of your employer or a postgraduate student of an affiliated institution which is paying any applicable article publishing charge ("APC") for Open Access articles. Where the Submitting Author wishes to make the Work available on an Open Access basis (and intends to pay the relevant APC), the terms of reuse of such Open Access shall be governed by a Creative Commons licence – details of these licences and which Creative Commons licence will apply to this Work are set out in our licence referred to above.

Other than as permitted in any relevant BMJ Author's Self Archiving Policies, I confirm this Work has not been accepted for publication elsewhere, is not being considered for publication elsewhere and does not duplicate material already published. I confirm all authors consent to publication of this Work and authorise the granting of this licence.

For peer review only

Stakeholders' perspectives on clinical trial acceptability and approach to consent within a limited timeframe: a mixed methods study.

Deja, E[1], Donohue, C[2], Semple, M G[3, 4], and Woolfall K[1] for the BESS Investigators.

1 Corresponding author. Dr Elizabeth Deja PhD, Department of Public Health, Policy and Systems, University of Liverpool, Liverpool, UK. E.deja@Liverpool.ac.uk

2 *Liverpool Clinical Trials Centre, University of Liverpool*

3 NIHR Health Protection Research Unit in Emerging and Zoonotic Infections, and Institute of Infection, Veterinary and Ecological Sciences, Faculty of Health and Life Sciences, University of Liverpool, Liverpool, UK

4 Respiratory Medicine, Alder Hey Children's Hospital, Liverpool L12 2AP, UK

Elizabeth Deja, PhD, ORCID 0000-0002-3626-4927,
Chloe Donohue, BSc ORCID 0000-0001-6379-8214
Malcolm G Semple, PhD, ORCID 0000-0001-9700-0418
Kerry Woolfall, PhD, ORCID 0000-0002-5726-5304

Word Count:
3516

Key words

Informed Consent, Qualitative, Intensive Care Units, Bronchiolitis, Randomised Controlled Trials.

Abstract

Objectives: The Bronchiolitis Endotracheal Surfactant Study (BESS) is a randomised controlled trial to determine the efficacy of endo-tracheal surfactant therapy for critically ill infants with bronchiolitis. To explore acceptability of BESS, including approach to consent within a limited time frame, we explored parent and staff experiences of trial involvement in the first two bronchiolitis seasons to inform subsequent trial conduct.

Design: A mixed-method embedded study involving a site staff survey, questionnaires and interviews with parents approached about BESS.

Setting: Fourteen UK Paediatric intensive care units.

Participants: Of the 179 parents of children approached to take part in Bess, 75 parents (of 69 children) took part in the embedded study. Of these, 55/69 (78%) completed a questionnaire, and 15/69 (21%) were interviewed. Thirty-eight staff completed a questionnaire.

Results: Parents and staff found the trial acceptable. All constructs of the Adapted Theoretical Framework of Acceptability were met. Parents viewed surfactant as being low risk and hoped their child's participation would help others in the future. Although parents supported research without prior consent in studies of time critical interventions, they believed there was sufficient time to consider this trial. Parents recommended that prospective informed consent should continue to be sought for BESS. Many felt that the time between the consent process and intervention being administered took too long and should be 'streamlined' to avoid delays in administration of trial interventions. Staff described how the training and trial processes worked well, yet patients were missed due to lack of staff to deliver the intervention, particularly at weekends.

Conclusion: Parents and staff supported BESS trial and highlighted aspects of the protocol, which should be refined, including a streamlined informed consent process. Findings will be useful to inform proportionate approaches to consent in future paediatric trials where there is a short timeframe for consent discussions.

Trial registration number: ISRCTN 11746266

Strengths and Limitations of this study

- Use of the Deja et al's (2021) adapted theoretical framework of acceptability allowed trial acceptability to be evaluated as a multifaceted construct as opposed to a poorly defined binary (acceptable/not acceptable) approach.
- Acceptability was explored from multiple perspectives providing an in-depth understanding of key stakeholder views.
- Acceptability was measured at multiple time points. The study may have benefited from more direct feedback from parents who declined their child's involvement in BESS.

Introduction

Bronchiolitis of infancy is a seasonal respiratory viral disease that most commonly causes rhinorrhoea, mild fever, and a wet cough but in severe cases can result in feeding difficulties and respiratory distress. It is the single most common reason for hospital admission of infants (children age <1yr) (1). The youngest of these, those born prematurely and those with underlying conditions are most often and most severely affected (2). There is no vaccine or specific treatment for bronchiolitis (3). Despite advances in the provision of non-invasive modes of respiratory support, admissions to UK Paediatric Intensive Care Units (PICU) and duration of Mechanical Ventilation for life-threatening bronchiolitis have remained fairly static (1), with the exception of during the COVID pandemic which indirectly lead to a decrease in all childhood infections (4).

Pulmonary surfactant is secreted by lung alveolar cells to reduce surface tension so increasing compliance of the lungs, allowing them to inflate more easily and reduce the work of breathing (5). Studies of infants with life-threatening bronchiolitis show reduced lung compliance and relative surfactant deficiency (6-10). The systematic review by Jat and Chawla (2015) found that surfactant therapy in infants with critical illness due to bronchiolitis was: safe, improved gas exchange and reduced both duration of mechanical ventilation and length of stay on PICU (5).

The Bronchiolitis Endotracheal Surfactant Study (BESS) is a phase 2, blinded randomised controlled trial designed to explore the efficacy and mechanism of surfactant

For peer review only - http://bmjopen.bmj.com/site/about/guidelines.xhtml

therapy compared to air placebo for critically ill infants with bronchiolitis (see supplementary material 1 for protocol). BESS is recruiting in up to 14 PICUs in UK Tertiary hospitals, with the intervention mostly being administered by respiratory physiotherapists. Informed consent was sought from parents/legal representatives (referred to hereon as parents) for their child's participation in BESS. Due to the time-dependent nature of the intervention, randomisation is required within 48 hours of intubation. This meant that urgent action was needed for the purposes of the trial, which is in line with legislation requirements for research without prior consent (6). However, it was unclear if informed consent could be reasonably sought within this limited time frame. In designing the study we identified a need to explore parents' views on the acceptability of seeking informed consent in BESS within a limited time window with a view to consider an alternative approach, such as research without prior consent (7), depending on parents' views and experiences of the recruitment and consent process.

Qualitative research to incorporate staff, patient or parent perspectives in the design of a clinical trial can help ensure the trial is acceptable, appropriate and possible to conduct (8). BESS included an embedded mixed methods study in the first two seasons of the trial to explore staff and parents' perspectives on the acceptability of the trial; approach to recruitment and consent; decision-making in the emergency setting; and barriers to participation. This paper presents the findings from the embedded study to inform ongoing recruitment to BESS and future trials in emergency and critical care settings.

For peer review only - http://bmjopen.bmj.com/site/about/guidelines.xhtml

Methods

Study design

A mixed methods study embedded within a clinical trial (BESS). Questionnaires and interviews with parents approached for informed consent in BESS, and a survey of BESS site staff involved in trial recruitment, consent procedures and intervention administration. We used previous embedded or trial feasibility studies (9-12) and the Adapted Theoretical Framework of Acceptability (ATFA) (13) to inform the design, including sample estimation, recruitment strategy, parent interview topic guide (see supplementary material 2), parent questionnaire and site staff questionnaire (see supplementary material 3). The parent questionnaire followed the same format as those used in similar studies (10, 12, 14) consisting of Likert scale questions from the Decision Making Control Instrument (15) and free text responses, taking on average five minutes to complete (See supplementary material 4).

Preliminary findings from parent interviews in season 1 and 2 (2019/2020) also informed the development the Site Staff Questionnaire, which consisted of 19 questions around the staffs' professional background, views on BESS training, BESS research Process, and BESS consent process. The site staff questionnaire was conducted at the end of season 2.

Patient and Public Involvement

The BESS study and embedded study had Patient and Public Involvement throughout. Six Parents of infants admitted to PICU and ventilated for life-threatening bronchiolitis and one member of the NIHR GenerationR Young Persons Advisory Group co-developed the study design and all study documents, some of which went on to be part of the BESS Parents Advisory Group. Two members are co-applicants and sit on the Trial Management Group and Trial Steering Committee.

For peer review only - http://bmjopen.bmj.com/site/about/guidelines.xhtml

Representatives of the Paediatric Intensive Care Society as stakeholders in the research output reviewed an early draft and this submitted application.

Parents/legal representatives

Eligibility, recruitment and sampling procedure

All parents of children approached about BESS were eligible to take part if they spoke English, including those who declined their child's participation in BESS.

Site staff provided parents with information about the embedded study as part of the BESS Participant Information Sheet (PIS) and recruitment discussions. Staff asked each parent (decliners and consenters) if they would like to complete the questionnaire after the recruitment discussion and/or if they were willing to take part in a telephone interview with the BESS researcher (ED) after their child had been discharged from hospital. Parents placed completed questionnaires in a stamped addressed envelope, which were collected by site staff and posted to ED. As part of the recruitment process, site research staff asked parents who declined to take part in BESS to provide a: reason why consent was not provided. If a reason was provided this was recorded on the BESS screening log.

Data collection continued until thematic saturation, where additional data did not lead to any new major themes identified during analysis. Researchers were also looking for high levels of 'information redundancy' (16-18) and information power, the point when data is deemed to address the study aims; sample specificity, such as experience relevant to the study aims, and sample diversity (16, 19).

Interview screening and conduct

Parents were contacted to arrange a telephone interview within one month of consent after checking each child's discharge and survival status with sites. Initially, parents were contacted in sequential order, then purposively sampled to ensure parents from all sites and decliners to BESS

For peer review only - http://bmjopen.bmj.com/site/about/guidelines.xhtml

were represented, not just consenters from the first sites opened (the largest population). Informed consent for audio recording of interviews was checked verbally at the time of the interview. The interviews explored views and experiences about: their child's admission to hospital, the BESS consent process, including how and when the trial was introduced by staff; information materials; and consent decision-making. This included questions about how trial processes could be improved and the potential use and acceptability of research without prior consent in subsequent seasons (see supplementary material 2). Respondent validation was used to add unanticipated topics to the topic guide as interviewing and analysis progressed (18). After the interview was complete, parents were sent a thank you letter, and a £20 Amazon voucher for their time.

Site staff Questionnaire

At the end of season 2, the BESS Trial Manager (CD) sent an email invitation to site staff requesting their participation in the online questionnaire and to cascade the link to colleagues involved in the trial. Email invitations described how completion of the questionnaire was taken as an indication of consent. Reminders were sent by (CD) and the BESS Chief Investigator (MGS) to all site key contacts after one week.

Analysis

Digital audio recordings were transcribed verbatim by a professional transcription company (UK Transcription, Brighton) and anonymised. Qualitative analysis of interviews and open response questionnaire and screening log data was interpretive and iterative (20). Utilising a thematic analysis approach, the aim was to provide an accurate representation of views on trial acceptability, design and processes (21, 22) (see supplementary material 5). NVivo 12 software (QSR International Pty Ltd., Melbourne, Australia) was used to assist in the organisation and coding of data. Data from the parent and staff questionnaires were cleaned and entered into SPSS Version 24.0 (IBM Corp., Armonk, NY, US). Descriptive statistics are presented with percentages. Synthesis of qualitative and quantitative data drew on the constant comparative method (23, 24). This involved ED (PhD, female

psychologist, Research Associate) and KW (PhD, female, social scientist, Reader) looking across quantitative and qualitative themes and quantitative output for themes/data output related to trial acceptability.

A final stage of analysis involved consideration of both qualitative and quantitative findings against the ATFA (13, 25) to help conceptualise and discuss the overall acceptability of the proposed trial.

The ATFA is designed to assist researchers in assessing the acceptability of healthcare interventions including paediatric clinical trials. The framework explores eight aspects of acceptability at three possible time points, before (*prospective*), during (*concurrent*) and after (*retrospective*) the intervention (See supplementary material 6). Our data were a mix of *Concurrent* and *Retrospective* data (see Tables 1 & 2).

RESULTS

Sample

Of the 179 parents of children approached to take part in Bess, 75 parents (of 69 children) took part in the embedded study (see Figure 1). Of these, 55/69 (78%) completed a questionnaire, 10/69 (15%) took part in an interview and 5/69 (7%) took part in both methods from 13/14 (93%) BESS sites. Thematic saturation was reached after eight interviews. An additional seven interviews were conducted to ensure variance across BESS sites and achieve information power (16). Parents were interviewed on average (mean) 27 days (SD 7.8 days, range 17-43 days) after randomisation. The mean duration of PICU admission was 8 days (range 2.5-18 days), with a mean of 7 days (range 2.5-17 days) on a ventilator and a mean age of 70 days old (range 15-156 days). Interviews took on average (mean) 37 minutes (SD 9.6 minutes, range 20-57). Only 1/75 (1%) parent who consented to the embedded study had declined to consent to their child's involvement in BESS. Thirty seven

For peer review only - http://bmjopen.bmj.com/site/about/guidelines.xhtml

(37/44, 84%) parents provided site research staff with a reason why they had declined consent, which was recorded in the BESS screening log.

Thirty-nine staff at all 14 open sites BESS sites completed the online questionnaire including 14 doctors, 12 Physiotherapists, 9 Research nurses and 1 nurse. All participants apart from the site trial coordinator were involved in the clinical care of children with varied levels of experience of recruiting to paediatric clinical trials (range 0-20 years, median 4 years).

Figure 1. Parent recruitment [insert]

Main findings

Acceptability of the BESS trial

Staff supported the trial, meeting all eight components of the ATFA (as shown in table 2). They had a clear understating of the aim of the study and how to follow the protocol. They viewed the administration of the intervention as being acceptable and ethical with free text comments highlighting how the trial had been: *"well received by PICU staff and parents"* (P31, Research nurse). Indeed, parents described their support for the trial. Many appeared to view BESS as an extension of standard care as the physiotherapy and tests conducted may have occurred anyway at different times in their child's clinical care. Consent decisions appeared to be informed (see below) as parents understood what BESS was investigating and why. They described their child's involvement as being low risk and were reassured by information about how surfactant was used in premature babies with very few side effects:

> *"I asked if there would be any side effects and things like that, and she said because it wasn't a new drug, she said there haven't been on the ones they had used it on previously, that they used it on premature babies."* P04, mother, interview.

Participation was not considered to place an additional burden on the child, although some parents found physiotherapy uncomfortable to watch and chose to leave the room:

For peer review only - http://bmjopen.bmj.com/site/about/guidelines.xhtml

> "*A lot of his physios, I was watching one where they'd, like, shake your baby. (Laughter) Then they push down on their ribs like they compress the ribs. I'm sure that that's what they were doing before they were going to possibly give the placebo or the surfactant. … It's frightening; I left… Because I thought, they don't need a panicky mum around them.*" P14, mother, interview.

Recruitment and consent in an emergency setting

Early in the interview, parents were asked to explain their understanding of the BESS Study. Whilst the depth of descriptions varied, all gave a clear and accurate account of the purpose of the trial. Many outlined primary and secondary outcomes, including how surfactant might: "*reduce the amount of time on a ventilator"* (P12, mother, interview), prevent: "*lungs from sticking*" (P09, father, interview), help them: "*to expand and open their lungs*" (P02, mother, interview) and hopefully help: "*them recover quicker*" (P10, father, interview) therefore reducing parental and child distress, cost, ventilator related infections.

Despite being approached soon after their child's admission to PICU, all interview and questionnaire participants stated that they were given sufficient time to consider the information and ask questions and that staff had broached BESS at '*an appropriate time*' (100%, questionnaire item) (see Tables 1 and 2). Parents described being able to make an informed consent decision about the trial, despite the highly emotive and stressful situation:

> "*I guess the main elephant in the room is, do I feel as if, because I was in a stressful situation, an emotional situation, where I wasn't in my right mind, where I'm having to deal with a lot of different, very scary, very emotional, very worrying situations, was I making clear and conscious decisions? The answer is yes, I was*" P13, father, interview.

A total of 44/179 (25%) declined their child's participation in the BESS study. Reasons given for not taking part included: situational capacity as there was: "*too much already going on*." (Mother, questionnaire, decliner) and: '*do not want to take part in research*' (19/179, 11%)

For peer review only - http://bmjopen.bmj.com/site/about/guidelines.xhtml

Screening logs). Others declined as their child was close to coming off the ventilator at the time of final consent. A minority of parents '*did not want their child to receive surfactant*' (9/179, 5%, Screening logs). However, most: *"Families were much more open to surfactant than I expected*," (P19, Research nurse) "*especially if their child had been premature and had surfactant in the neonatal period*." (P10, Physiotherapist). One nurse noted that the number of decliners was similar to other PICU trials: *"Few exceptions where they didn't want child as a guinea pig, but no more than usual"* (P6, Nurse).

Facilitating quicker intervention delivery

Both groups highlighted that sometimes there were relatively long stretches of time between initial trial recruitment discussions and trial interventions beginning. The length of time was dependent on the availability of research nurses (who largely work 9am-5pm on weekdays) and parents for the consent discussions and availability of physiotherapists to administer the intervention: "*delaying the whole process*" (P15, Research nurse):

> "I think the only thing that could have possibly done better was doing it sooner because..potentially, it [the intervention] could have started a day earlier. The actual time between them approaching me, to me to find out what it is, to me to say yes and then actually starting the trial, I feel that could have been really streamlined" P13, father, interview.

> "Low on personnel on the ground as medics are undertaking the intervention which takes considerable time in a busy unit" P06, Doctor

In addition to increasing the: *"potential for patients to begin weaning from the ventilator and therefore not be eligible"* (P23, Research nurse). During interviews, parents questioned the ethicality and impact of delaying a potentially effective treatment they had consented to:

For peer review only - http://bmjopen.bmj.com/site/about/guidelines.xhtml

"If there was someone on hand to make it happen despite the shift change [...] because you want the test to actually be effective if you're going to take part, don't you? You don't want to waste time." P11, father, interview.

Parents provided practical suggestions for speeding up the recruitment and consent process, for example having the written information available at the bedside so that they would likely have read it before the first approach allowing for those comfortable to consent without a follow-up discussion. Therefore, removing a step in the recruitment process timeline. Additionally, they highlighted the need to have more staff available that can take consent and administer the intervention.

Perceptions of Research without Prior Consent in BESS

Figure 2 Description of RWPC read to participants during interview [Insert]

Towards the end of the interviews, a definition of research without prior consent (RWPC) was read to parents (Figure 2). All parents responded positively to the use of RWPC in critical care research in emergency situations for trials that were regarded as being low risk to their child's wellbeing.

"As long as it's something that doesn't have any risks associated with it, or there wouldn't be a reason why you wouldn't want them to do it and it's something that's going to help them, then I think its (RWPC) a good thing." P08, mother, interview.

Five parents described consenting to another trial that had used a RWPC approach during the same hospital admittance. Nevertheless, the majority (n = 11/ 15, 74%) of parents interviewed suggested that BESS should continue to use an informed consent process as the intervention was not viewed to be time critical in that surfactant was regarded as something to hopefully get children: "*off the ventilator quicker" rather than an emergency intervention that would be lifesaving "If we*

For peer review only - http://bmjopen.bmj.com/site/about/guidelines.xhtml

don't give this to her immediately" (P08, mother, interview). In contrast, three parents were in favour of RWPC in the BESS study and there were no strong objections against its possible use, as the intervention was viewed to be low risk, would facilitate the intervention being given more quickly, thus avoiding missed patients and increasing recruitment rates. As the following quote illustrates:

> "If you'd had just cracked on and done it, it could've made a difference quicker, but by the time we got around to the actual consent, he was being extubated" P05, mother, interview

For peer review only - http://bmjopen.bmj.com/site/about/guidelines.xhtml

Table 1. Parent concordant acceptability of BESS mapped to the adapted framework of acceptability Deja E. et al., 2021

Affective attitude	Burden	Ethicality	Intervention coherence	Opportunity costs	Self-efficacy	Perceived effectiveness	Trust
135/ 179 (75 %) consented for their child to take part in BESS.	50/60 (83%) did not find it difficult to take in information about BESS<br><br>47/60 (78%) did not find it difficult to make a decision about BESS<br><br>“We felt the process was straightforward” MQ34	60/60 (100%) agreed that they were satisfied with the consent process<br><br>55/60 (92%) felt that medical studies like BESS are important	60/ 60 (100%) agreed that the Information received about BESS was clear and straightforward to understand	No relevant data was reported at this time point	60/67 (95%) felt that they made the decision for their child to take part in the pilot trial.<br><br>54/60 (90%) felt in control of the decision to take part.<br><br>“Medical team made it clear that this decision was mine fully and I could withdraw consent at any time. This knowledge made participating much easier.’ MQ45	50/53 (88.3%) Selected ‘helping my child’ as a reason for taking part<br><br>57/60= 95% selected ‘to help other children in the future’ as a reason for taking part<br><br>“I think all children would benefit from this great work thanks” FQ42	45/60 (75%) selected ‘because I trusted the doctor or nurse who explained BESS’ as a reason for taking part. *<br><br>“Trusting the doctor or nurse explaining BESS to parents is going to be an important deciding factor subconsciously for most parents” FQ30
9/179 declined to take part because they did not want their child to receive porcine surfactant<br><br>19/179 Do not want to take part in research	“My partner & I decided it was too much to take on when our son is so sick, too much already going on.” MQ53- decliner’						

* Note, not selecting this *did not mean* that they did not trust the staff, it was just not a identified reason for their consent.

Tables Key: Shaded fields highlight potentially unacceptable aspects of the trial MQ mother open questionnaire response, MF: father open questionnaire response. MI: mother interview, FI Father Interview.

Table 2. Retrospective acceptability of BESS mapped to the adapted framework of acceptability Deja. E et al., 2021

Affective attitude	Burden	Ethicality	Intervention coherence	Opportunity costs	Self-efficacy	Perceived effectiveness	Trust
Parental Acceptability							
Happy with trial "obviously I was approached for the BESS trial, which I was quite happy for him to take part in."MI02	'But the standard of care for the placebo is just normal care anyway' MI06	'You feel powerless, to be entered into a research study without somebody talk to you about it you've got even more power taken away from you.' MI12<br><br>"you want the test to actually be effective if you're going to take part, don't you? You don't want to waste time." FI11	"If they can do a trial that shows that actually giving the surfactant or not brings them out of it quicker" MI04<br><br>'literally any question I had in my head, this leaflet then had contained the answers" MI05	"There are not really risks, so there's not really any problem, all it could possibly do is produce positives. FI13	"She was so pleasant and she was so easy to talk to. Even when I wasn't discussing the paperwork and the trial with her, if I had any questions, I could easily walk up and ask, if I had any enquiries. She was very, very easy to approach." MI02	"Then it was a realisation of, oh, my God, so you've just told me this thing that could make this difference and could get him off the ventilator quicker, and he might not get it anyway' MI05	'I don't think they'd put your babies in danger. They are here to get them better, so you just kind of trust in what they are doing." MI4
Staff Acceptability							
Overall how acceptable do you find the BESS study? 20/36 (53%) very acceptable 18/36 (47%) acceptable<br><br>"If it will have a clinical benefit which outweighs potential risk, then it is definitely acceptable" (P14)	*"Low on personnel on the ground as medics are undertaking the intervention which takes considerable time in a busy unit"* (P06, Doctor)	10/36 (26%) found it very acceptable and 28/36 (74%) acceptable to administer the surfactant.<br><br>6 /36 (16%) found it very acceptable and 31/36 (82%) found it acceptable to administer the placebo	The majority of staff rated the training as 'excellent' (27/36, 75%) or 'good' (9/36, 25%).	*"We tried to time other interventions that required muscle relaxants e.g. ETT tape changes with the time the placebo needed to be given"* (P39, Physiotherapist).	34/36 (87%) staff indicated that they had not experienced difficulties adhering to the BESS protocol.	"I think it's important that we look at ways to improve care and treatment so research trials are vital. Reducing ventilator days would help free up precious ITU beds during winter and hopefully reduce problems with lung injuries" (P17)	Not mentioned in questionnaire responses

Discussion

The aim of this study was to explore the acceptability of the BESS Study to help inform recruitment in subsequent bronchiolitis seasons and trials in other paediatric critical care settings. The Adapted Theoretical Framework of Acceptability (13) allowed us to explore the multifaceted construct of acceptability, with eight components to consider. Our data suggest that the BESS Study met all eight of the constructs. BESS was viewed by parents and PICU staff as understandable, posing low to no risk and possible benefits to child participants.

Our findings suggest that both parents and staff viewed the BESS study as ethical and potentially beneficial to children with bronchiolitis. At the study design stage, there was uncertainty about whether an informed consent approach was appropriate for BESS or whether RWPC should be used due to the critical care context and the need for 'urgent action' for purposes of the trial, which is in line with RWPC legislation requirements (26). Although RWPC was supported by a minority of parents as it would reduce any delay in their child receiving the intervention, many stated there was opportunity for an appropriately timed trial discussion before randomisation. Our findings support the continued use of a proportionate approach (27) to informed consent in this context. Parents stated that the length of the consent process and staff availability impacted the timeliness of administering the BESS intervention. They were concerned that such delays may reduce the potential effectiveness of the intervention. The nature of the intervention, which was viewed as being low risk to their child, meant that parents felt they could make an informed decision after one conversation with research staff, when their child's condition was stable. A streamlined consent process, along with other suggested changes to study processes should allow the full intervention to be administered in a timelier manner.

The seasonal nature of bronchiolitis provided an opportunity to review and adapt trial processes, increasing opportunities to make the trial more acceptable acceptability and feasibility to conduct. However, most trials do not have this ability leading to potential research and resource waste (28). Our findings highlight the importance of pre-trial research or embedded studies in which

For peer review only - http://bmjopen.bmj.com/site/about/guidelines.xhtml

the potential time windows between randomisation and intervention delivery can be clearly mapped and parent views on the most appropriate consent approach are fully explored.

The main strength of this study is that acceptability was measured at multiple time points, from different perspectives providing an in-depth understanding of key stakeholder views. The study may have benefited from more direct feedback from parents who declined their child's involvement in BESS (29). We kept recruitment open for decliners for an additional season to try to address this gap in knowledge. However, their wish to 'not take part in research' and 'situational incapacity' in BESS, also applied to the embedded study and was compounded by low recruitment in season three as a consequence of COVID (4). We believe that data from screening logs and the one decliner questionnaire provided insight into reasons for declining consent. In addition, the overall number of decliners was relatively low. Therefore, this missing data should not have a large impact on our findings.

Conclusion

Our paper found BESS to be feasible and acceptable, supported the continued use of informed consent and highlighted areas for study process improvements. Future trials should consider proportionate consent processes when interventions are low risk and there is a short window for informed consent discussions.

For peer review only - http://bmjopen.bmj.com/site/about/guidelines.xhtml

Acknowledgments

We firstly thank the children and families who participated in the BESS. Without your help this research could not have taken place. We also thank the principal investigators, respiratory physiotherapists and research nurses at each study site.

We would also like to thank the National Institute for Health Research's Efficacy and Mechanism Evaluation (EME) Programme (ref: 12/205/28) for funding this research.

Author contributions

MGS Chief Investigator; led the conception and design of the BESS study, KW designed and led the embedded study. CD facilitated the acquisition of data for analysis and trial management. ED conducted the interviews. ED and KW analysed and interpreted the data. ED and KW drafted the manuscript. ED, KW, MGS, CD and the BESS Investigators reviewed the manuscript critically for important intellectual content and provided approval of the version to be published. BESS Investigators' other contributions can be seen in the supplementary material.

BESS Investigators

Chief Investigator: Malcolm G Semple PhD, (Department of Clinical Infection, Microbiology and Immunology, Institute of Infection, Veterinary and Ecological Sciences, University of Liverpool and Department of Respiratory Medicine, Alder Hey Children's NHS Foundation Trust, Liverpool)

Deputy Chief Investigator: Paul S McNamara PhD, (Department of Women's and Children's Health, Institute of Life Course and Medical Sciences, University of Liverpool and Department of Respiratory Medicine, Alder Hey Children's NHS Foundation Trust, Liverpool)

Coinvestigators: Evette Allen PhD, Clare Fowler HNC (Parent Lay Representatives), Catrin Barker PGDipClinPharm, Kentigern Thorburn MD, Matthew Peak PhD, Paul C Ritson MSc, Clare van Miert PhD, (Alder Hey Children's NHS Foundation Trust, Liverpool), Ashley Best MSc, Chloe Donohue BSc, Ashley Jones PhD, Tracy Moitt, Laura Price BSc, Paula Williamson PhD (Liverpool Clinical Trials Centre, University of Liverpool, Liverpool), Howard W Clark PhD, Jens Madsen PhD (Elizabeth Garrett Anderson Institute for Women's Health, University College London, London), Anne Dawson, Colin Summers (Faculty of Health and Life Sciences, University of Liverpool, Liverpool), Elizabeth Deja PhD, Kerry Woolfall PhD (Institute of Population Health, University of Liverpool), Blessing Osaghae PhD, Mark Turner PhD (Department of Women's and Children's Health, Institute of Life Course and Medical Sciences, University of Liverpool), Madhuri Panchal BSc, Anthony Postle PhD, (National Institute for Health Research Southampton Biomedical Research Centre Clinical and Experimental Sciences, Faculty of Medicine, University of Southampton, Southampton), John Pappachan MBBChir, (Southampton Children's Hospital, Southampton), Roger Parslow PhD (Division of Epidemiology and Biostatistics, Leeds Institute of Cardiovascular and Metabolic Medicine, University of Leeds, Leeds), Jennifer Preston BA, (NIHR Alder Hey Clinical Research Facility, University of Liverpool, Liverpool)

For peer review only - http://bmjopen.bmj.com/site/about/guidelines.xhtml

Site Investigators:

Alder Hey Children's Hospital, Liverpool: Kentigern Thorburn MD (Principal Investigator), Vanessa Compton, Peter Jirasek, Dawn Jones, Michael Mander, Laura O'Malley, Laura Rad, Laura Rimmer, Paul C Ritson MSc, Chris Simons.

Birmingham Children's Hospital, Birmingham: Afeda Mohamed Ali MBBCh, Kevin P Morris MD (Principal Investigators), Cara Alexander BSc, Wendy Browne MSc, Hannah Child BSc, Stephanie Clarke BSc, Sarah Fox BN, Natalie Milburn BSc, Philip Milner FIBS, Samantha Owen BSc, Holly Parkin BSc, Harriet Payne BSc, Carly Tooke, Helen Winmill MSc.

Bristol Royal Hospital for Children, Bristol: Peter J Davis MBChB (Principal Investigator), Katherine Baptiste, Sophie Coles BSc, Sarah-Jayne Eames BSc, Christina Linton BSc, Helen Marley BSc, Sarah Mogan BSc, Alvin Schadenberg PhD, John Stiven BSc.

Great North Children's Hospital, Newcastle: Rachel Agbeko PhD (Principal Investigator), Rob Claydon PgDip, Christine Mackerness, Anna Stancombe BSc, Kate Teeley BSc, Grace Williamson MD.

Leeds Children's Hospital, Leeds: Santosh Sundararajan MD (Principal Investigator), Ramesh Kumar, Kathryn Reeves BSc, Emily Scriven.

Leicester Children's Hospital, Leicester: Raghu N Ramaiah MBBS (Principal Investigator), Rekha Patel.

Nottingham Children's Hospital, Nottingham: Patrick E Davies BMBS (Principal Investigator), Lindsay Crate, Simon Gates

Oxford Children's Hospital, Oxford: James Weitz MBBS (Principal Investigator), Kirsten Beadon BSc, Rachel McMinnis BSc, Zoe Oliver BSc, Avishay Sarfatti BMBCh, Frances Sinfield, Charlotte Thompson.

Royal Belfast Hospital for Sick Children, Belfast: Julie Richardson MBChB (Principal Investigator), Hilary Callaghan BSc, Vicki Linton BSc, Jeremy Lyons MD, Clara Nelson BSc, Mark Terris MBChB

Royal Hospital for Children and Young People, Edinburgh: Tsz-Yan Milly Lo PhD (Principal Investigator), David Armstrong MD, Steve Cunningham PhD, Jackie McCormick, Andrea Wood MSc.

Royal Hospital for Children, Glasgow: Richard Levin MBChB, (Principal Investigator), Edgar Brincat MD, Christopher Lamb RN, Ross Marscheider.

Royal Manchester Children's Hospital, Manchester: Stephen D Playfor DM (Principal Investigator), Bernadette C Gavin MSc, Dave J Morgan MSc, Lara T Bunni MSc, Claire F Jennings MClinRes, Rebecca Marshall DipHE(Nur), Emma K Riley MNurSci.

Southampton Children's Hospital, Southampton: John Pappachan MBBChir, Ahmed Osman MB ChB (Principal Investigators), Lorena Caruana, Hannah Clarke, Amber Cook BSc, Tracey Curtis, Nichola Etherington, Michael Griksaitis, Jenni McCorkell BSc, Christie Mellish, Simone Paulson BMBCh, Jenny Pond BSc, Catherine Postlethwaite DipPaedNur.

Staffordshire Children's Hospital, Stoke: Constantinos Kanaris PhD (Principal Investigator), John Alexander MBChB, Nicola McClelland, Holly Minchin, Helen Parker, Pavanasam Ramesh, Joanne Tomlinson.

The Royal London Children's Hospital, London: Simona Lampariello MD (Principal Investigator), Bessie Cipriano, Nosheen Khalid MSc, Ramiya Kirupananthan MBBS, Craig Knott MBBS, Tara Murray,

For peer review only - http://bmjopen.bmj.com/site/about/guidelines.xhtml

Olivia Nugent, Nicolene Plaatjies, Samantha Reed, Natasha Roberts, Christa Ronan, Salman Siddiqi MB BS, Natasha Thorn BMBS

Funding

This work was supported by the National Institute for Health Research's Efficacy and Mechanism Evaluation (EME) Programme (ref: 12/205/28).

Conflicts of Interests

MGS**:** Independent external and non-remunerated member of Pfizer's External Data Monitoring Committee for their mRNA vaccine program(s); HMG UK Scientific Advisory Group for Emergencies (SAGE), COVID-19 Response, Non-remunerated independent member March 2020 to March 2022. HMG UK New Emerging Respiratory Virus Threats Advisory Group (NERVTAG), Non-remunerated independent member since 2014; Integrum Scientific LLC, Greensboro, NC, USA, Chair of Infectious Disease Scientific Advisory Board; MedEx Solutions Ltd, Director; Integrum Scientific LLC, Greensboro, NC, USA, Minority Owner: MedEx Solutions Ltd, Majority Owner.

Ethics Statement

Ethical approval for the study was given by the South Central Berkshire Research Ethics Committee (IRAS ID 220853).

Data sharing statement

No data are available. The datasets generated during and/or analysed during the current qualitative study are not publicly available as consent was not sought for data sharing**.**

Declaration.

This manuscript has been read and approved by all the authors, the requirements for authorship have been met, and each author believes that the manuscript represents honest work.

References

1. Green CA, Yeates D, Goldacre A, Sande C, Parslow RC, McShane P, et al. Admission to hospital for bronchiolitis in England: trends over five decades, geographical variation and association with perinatal characteristics and subsequent asthma. Arch Dis Child. 2016;101(2):140-6.
2. Auroux B, Hascoët J, Jarreau P, Magny J, Rozé J, Saliba E, et al. Risk factors for bronchiolitis hospitalization in infants: A French nationwide retrospective cohort study over four consecutive seasons (2009-2013). PLOS ONE. 2020;15(3).
3. Bronchiolitis N. Diagnosis and Management of Bronchiolitis in Children, Clinical Guideline NG9. National Institute for Clinical Excellence (NICE). 2015.
4. Kadambari S, Goldacre R, Morris E, Goldacre MJ, Pollard AJ. Indirect effects of the covid-19 pandemic on childhood infection in England: population based observational study. bmj. 2022;376.
5. Jat KR, Chawla D. Surfactant therapy for bronchiolitis in critically ill infants. Cochrane Database Syst Rev. 2015;2015(8):Cd009194.
6. Authority HR. In: NHS, editor. 2020.
7. Woolfall K, Frith L, Dawson A, Gamble C, Lyttle MD, Young B. Fifteen-minute consultation: an evidence-based approach to research without prior consent (deferred consent) in neonatal and paediatric critical care trials. Archives of disease in childhood Education and practice edition. 2016;101(1):49-53.
8. O'Cathain A, Hoddinott P, Lewin S, Thomas KJ, Young B, Adamson J, et al. Maximising the impact of qualitative research in feasibility studies for randomised controlled trials: guidance for researchers. Pilot and Feasibility Studies. 2015;1(1):1-13.
9. Molyneux S, Njue M, Boga M, Akello L, Olupot-Olupot P, Engoru C, et al. 'The Words Will Pass with the Blowing Wind': Staff and Parent Views of the Deferred Consent Process, with Prior Assent, Used in an Emergency Fluids Trial in Two African Hospitals. PLoS ONE. 2013;8(2):e54894.
10. O'Hara CB, Canter RR, Mouncey PR, Carter A, Jones N, Nadel S, et al. A qualitative feasibility study to inform a randomised controlled trial of fluid bolus therapy in septic shock. Arch Dis Child. 2017:archdischild-2016-312515.
11. Woolfall K, Frith L, Gamble C, Gilbert R, Mok Q, Young B. How parents and practitioners experience research without prior consent (deferred consent) for emergency research involving children with life threatening conditions: a mixed method study. BMJ Open. 2015;5(9):e008522.
12. Peters MJ, Woolfall K, Khan I, Deja E, Mouncey PR, Wulff J, et al. Different temperature thresholds for antipyretic intervention in critically ill children with fever due to infection: the FEVER feasibility RCT. Health Technology Assessment 2019;23(5):1 - 148.
13. Deja E, Peters MJ, Khan I, Mouncey PR, Agbeko R, Fenn B, et al. Establishing and augmenting views on the acceptability of a paediatric critical care randomised controlled trial (the FEVER trial): a mixed methods study. BMJ Open. 2021;11(3):e041952.
14. Appleton RE, Rainford NEA, Gamble C, Messahel S, Humphreys A, Hickey H, et al. Levetiracetam as an alternative to phenytoin for second-line emergency treatment of children with convulsive status epilepticus: the EcLiPSE RCT. 2020;24:58.
15. Miller VA, Ittenbach RF, Harris D, Reynolds WW, Beauchamp TL, Luce MF, et al. The Decision Making Control Instrument to Assess Voluntary Consent. Medical Decision Making. 2011;31(5):730-41.
16. Saunders B, Sim J, Kingstone T, Baker S, Waterfield J, Bartlam B, et al. Saturation in qualitative research: exploring its conceptualization and operationalization. Quality & Quantity. 2018;52(4):1893-907.
17. Baker SE, Edwards R. How many qualitative interviews is enough? National Centre for Research Methods (NCRM): . 2012.
18. Mays N, Pope C. Assessing quality in qualitative research. Bmj. 2000;320(7226):50-2.
19. Malterud K, Siersma V, Guassora A. Sample Size in Qualitative Interview Studies. Qualitative Health Research. Sage Journals. 2016;26(13):1753-60.

For peer review only - http://bmjopen.bmj.com/site/about/guidelines.xhtml

20. Braun V, Clarke V. What can "thematic analysis’’offer health and wellbeing researchers? International journal of qualitative studies on health and well-being. 2014;9:26152.
21. Byrne D. A worked example of Braun and Clarke’s approach to reflexive thematic analysis. Quality & Quantity. 2021:1-22.
22. Byrne D. A worked example of Braun and Clarke’s approach to reflexive thematic analysis. Quality & Quantity. 2021.
23. Glaser BG. The constant comparative method of qualitative analysis. Social problems. 1965;12(4):436-45.
24. Strauss A, Corbin J. Basics of qualitative research techniques. 1998.
25. Sekhon M, Cartwright M, Francis JJ. Acceptability of health care interventions: A theoretical framework and proposed research agenda. British Journal of Health Psychology. 2018;23(3):519-31.
26. The Medicines for Human Use (Clinical Trials) Regulations, (2004).
27. Authority HR. Applying a proportionate approach to the process of seeking consent: HRA Guidance. NHS Health Research Authority London; 2016.
28. Morgan B, Hejdenberg J, Hinrichs-Krapels S, Armstrong D. Do feasibility studies contribute to, or avoid, waste in research? PloS one. 2018;13(4):e0195951.
29. Hughes-Morley A, Young B, Hempel RJ, Russell IT, Waheed W, Bower P. What can we learn from trial decliners about improving recruitment? Qualitative study. Trials. 2016;17(1):1-13.

For peer review only - http://bmjopen.bmj.com/site/about/guidelines.xhtml

Figure Legends

Figure 1. Parent Recruitment

Figure 2. Description of RWPC read to participants during interview

Table 1. Parent concordant acceptability of BESS mapped to the adapted framework of acceptability Deja E. et al., 2021

Table 2. Retrospective acceptability of BESS mapped to the adapted framework of acceptability Deja. E et al., 2021

For peer review only - http://bmjopen.bmj.com/site/about/guidelines.xhtml

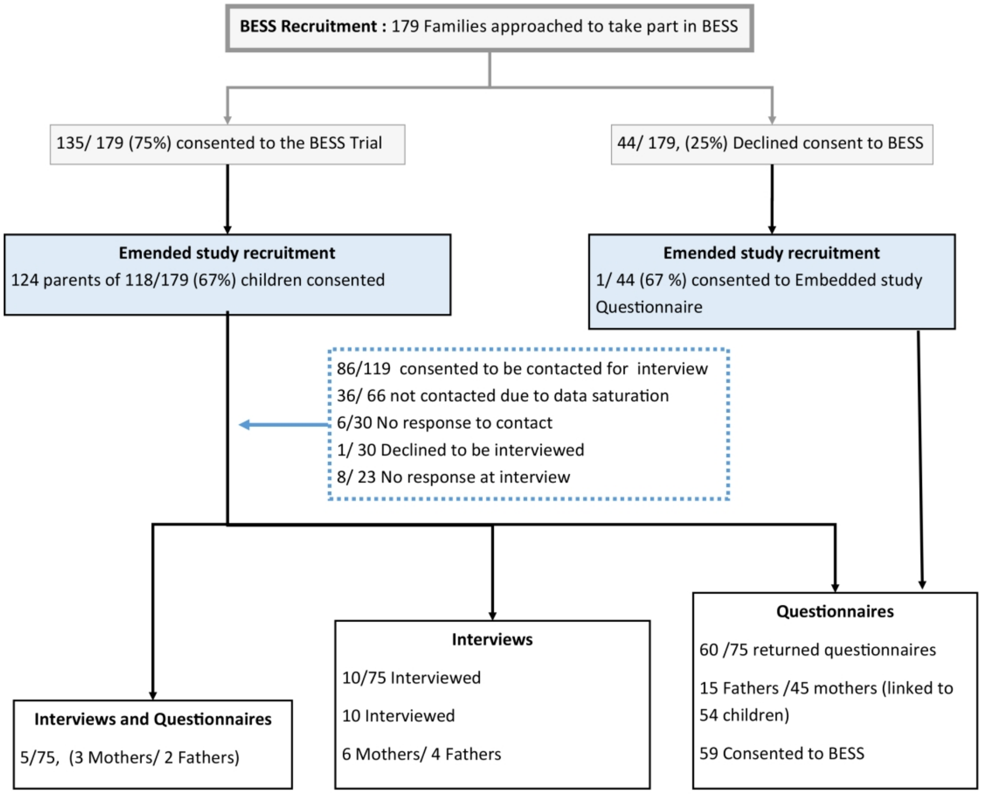

Figure 1 Parent recruitment

254x212mm (300 x 300 DPI)

In BESS and most research, consent is obtained before children are entered into a study. In some trials conducted in emergency situations parents are asked to provide consent after their child had already been actively involved in the Trial. At this point consent is sought to continue in the trial. This is known as research without prior consent or deferred consent.

The reason that this legislation is in place is because in situations like this (ie, in paediatric critical care), there's a belief that there's no time to have a full discussion and time to reflect about the research and that actually having that discussion might delay treatment.

Figure 2 Description of RWPC read to participants during interview

170x44mm (300 x 300 DPI)

The efficacy and mechanism of surfactant therapy for critically ill infants with bronchiolitis: The **B**ronchiolitis **E**ndotracheal **S**urfactant **S**tudy (BESS)

For peer review only

A phase-2 blinded randomised air-placebo-controlled trial of endotracheal surfactant (poractant alfa) in critically ill infants with bronchiolitis

BESS Protocol v2.0 28/09/2018

Study Sponsor(s):
University of Liverpool
Research Support Office
2nd Floor Block D Waterhouse
3 Brownlow Street
Liverpool
L69 3GA

EudraCT number: 2018-001169-18
ISRCTN :
Research Ethics Ref: 18/SC/0427
Sponsor Ref: UoL001360
IRAS Ref: 220853

For peer review only - http://bmjopen.bmj.com/site/about/guidelines.xhtml

PROTOCOL APPROVAL

I, the undersigned, hereby approve this clinical study protocol:

Authorised by Chief Investigator:

Signature: ______________________ **Date:** 28092018

Prof. Malcolm (Calum) Gracie Semple, PhD, BM BCh (Oxon), FRCPCH, FRCPE, FHEA

Consultant in Paediatric Respiratory Medicine, Alder Hey Children's NHS Foundation Trust
Professor of Child Health and Outbreak Medicine, University of Liverpool

Institute in The Park,
Alder Hey Children's Hospital
Eaton Road
Liverpool
L12 2AP

Authorised on behalf of Sponsor:

Signature: ______________________ **Date:** ____________

Mr Alex Astor

Head of Research Support
Research Support Office
2nd Floor Block D Waterhouse Building
3 Brownlow Street,
Liverpool
L69 3GL

Authorised on behalf of the Lead Statistician:

Signature: ______________________ **Date:** ____________

Dr Ashley Jones, PhD
Senior Statistician
Clinical Trials Research Centre
Institute of Child Health
Alder Hey Children's Hospital
Eaton Road
Liverpool
L12 2AP

For peer review only - http://bmjopen.bmj.com/site/about/guidelines.xhtml

PROTOCOL APPROVAL

I, the undersigned, hereby approve this clinical study protocol:

Authorised by Chief Investigator:

Signature: ______________________ **Date:** ____________

Prof. Malcolm (Calum) Gracie Semple, PhD, BM BCh (Oxon), FRCPCH, FRCPE, FHEA
Consultant in Paediatric Respiratory Medicine, Alder Hey Children's NHS Foundation Trust
Professor of Child Health and Outbreak Medicine, University of Liverpool
Institute in The Park,
Alder Hey Children's Hospital
Eaton Road
Liverpool
L12 2AP

Authorised on behalf of Sponsor:

Signature: ______________________ **Date:** 19/10/18

Mr Alex Astor

Head of Research Support
Research Support Office
2nd Floor Block D Waterhouse Building
3 Brownlow Street,
Liverpool
L69 3GL

Authorised on behalf of the Lead Statistician:

Signature: ______________________ **Date:** 28/9/18.

Dr Ashley Jones, PhD
Senior Statistician
Clinical Trials Research Centre
Institute of Child Health
Alder Hey Children's Hospital
Eaton Road
Liverpool
L12 2AP

For peer review only - http://bmjopen.bmj.com/site/about/guidelines.xhtml

General Information

This document describes the BESS trial including detailed information about procedures and recruitment. The protocol should not be used as an aide-memoir or guide for the treatment of other patients; every care was taken in its drafting, but corrections or amendments may be necessary. These will be circulated to the registered investigators in the trial, but centres entering patients for the first time are advised to contact the coordinating centre (Clinical Trials Research Centre (CTRC), University of Liverpool) to confirm they have the most up to date version. Clinical problems relating to this trial should be referred to the relevant Chief Investigator, Professor Calum Semple, via the CTRC.

This protocol defines the participant characteristics required for study entry and the schedule of treatment and follow-up. Participant recruitment will be undertaken in compliance with this document, applicable regulatory and governance requirements and waivers to authorise non-compliance are not permitted.

Incidents of protocol non-compliance, whether reported prospectively (e.g. where a treatment cannot be administered on a scheduled date as a result of public holidays) or retrospectively noted (e.g. as a result of central monitoring) are recorded as protocol deviations, the incidence of which are monitored and reported to trial oversight committees.

The template content structure is consistent with the SPIRIT (Standard Protocol Item: Recommendations for Interventional Trials 2013) and has regard for the Health Research Authority guidance. Regulatory and ethical compliance information is located in section 12.

Relationship Statements

Roles and responsibilities are described in section 15

The University of Liverpool is the Sponsoring organisation. The University of Liverpool will formally delegate specific sponsoring roles to the Chief Investigator and Clinical Trials Unit, but remains legally responsible for the trial.

Clinical Trials Unit (CTU): The CTRC at the University of Liverpool in collaboration with the Chief Investigator, Prof Calum Semple, will have overall management responsibility for the trial from a CTU perspective and will be responsible for the co-ordination of centres.

CTRC as part of the Liverpool Trials Collaborative has achieved full registration by the UK Clinical Research Collaboration (www.ukcrc.org) as their standards and systems were assessed by an international review panel as reaching the highest quality. The CTRC has a diverse trial portfolio underpinned by methodological rigour, a Good Clinical Practice (GCP) compliant data management system, and core standard operating procedures (SOPs).

For peer review only - http://bmjopen.bmj.com/site/about/guidelines.xhtml

**Sponsor:**	**Trial Management, Monitoring and Analysis:**
University of Liverpool<br>Liverpool<br>L69 3BX<br>Tel: 0151 794 8290<br>Email: sponsor@liverpool.ac.uk	Clinical Trials Research Centre, University of Liverpool<br>Institute of Child Health<br>Alder Hey Children's Hospital NHS Foundation Trust<br>Liverpool<br>L12 2AP<br>Tel: 0151 795 8757<br>Fax: 0151 795 8770<br>Email: bess@liverpool.ac.uk
**Mechanistic & Microbiology Work:**	**Paediatric Intensive Care Audit Data:**
University of Southampton<br>University Road<br>Southampton<br>SO17 1BJ<br>Tel: 023 8059 5000<br>Email: ris@soton.ac.uk	PICANet<br>School of Medicine<br>8.49 Worsley Building<br>University of Leeds<br>Leeds<br>LS2 9JT<br>PICANet<br>0113 343 8125<br>picanet@leeds.ac.uk

For peer review only

For peer review only - http://bmjopen.bmj.com/site/about/guidelines.xhtml

Contact Details: Individuals

**Individual Authorised to Sign the Protocol and Protocol Amendments on behalf of the Sponsor:**	**Chief Investigator (CI):**
Mr Alex Astor<br>Research Support Office<br>2nd Floor, Block D, Waterhouse Building, 3 Brownlow Street, Liverpool, L69 3GL<br>0151 794 8739<br>sponsor@liv.ac.uk	Prof. MG (Calum) Semple,<br>Women's and Children's Health<br>University of Liverpool<br>Institute in the Park, Alder Hey Children's Hospital, Eaton Road, Liverpool, L12 2AP, UK<br>0151 252 5250<br>m.g.semple@liverpool.ac.uk
**Medical Expert who will Advise on Protocol Related Clinical Queries (If other than CI):**	**Medical Expert who will Evaluate SAE Reports (if other than CI):**
Prof. Paul S McNamara<br>Women's and Children's Health<br>University of Liverpool<br>Institute in the Park, Alder Hey Children's Hospital, Eaton Road, Liverpool, L12 2AP, UK<br>0151 252 5250<br>mcnamp@liverpool.ac.uk	Prof. Paul S McNamara<br>Women's and Children's Health<br>University of Liverpool<br>Institute in the Park, Alder Hey Children's Hospital, Eaton Road, Liverpool, L12 2AP, UK<br>0151 252 5250<br>mcnamp@liverpool.ac.uk
**Medical Expert who will Evaluate SAE Reports (If other than CI or cross cover):**	
Dr Kent Thorburn<br>Paediatric Intensive Care Unit, Alder Hey Children's Hospital, Eaton Road, Liverpool, L12 2AP, UK<br>0151 228 4811 ext. 4180<br>kent.thorburn@alderhey.nhs.uk	

For peer review only - http://bmjopen.bmj.com/site/about/guidelines.xhtml

Additional Contacts:

The contact details of other individuals involved in the trial are detailed in documents supplementary to the protocol and stored in the Trial Master File:

Contact	Document Title
Trial Management Group (TMG)	BESS Trial Oversight Committee Membership
Trial Steering Committee (TSC)	
Independent Data and Safety Monitoring Committee (IDSMC)	
Principal Investigators	BESS Participating Centres

For peer review only

For peer review only - http://bmjopen.bmj.com/site/about/guidelines.xhtml

1 TABLE OF CONTENTS

Protocol Approval 2

1 Table of Contents 7

Glossary 12

2 Protocol Summary 14

3 Introduction 17

3.1 Study Overview 17

3.2 Background 17

3.3 Rationale 18

3.4 Risk and Benefits 19

3.4.1 Potential Benefits 19

3.4.2 Potential Risks 19

3.5 Aims & Objectives of Work Package A 19

3.5.1 Aims 19

3.5.2 Primary Objective 20

3.5.3 Secondary Objective(s) 20

3.6 Outcome measures/endpoints 20

3.6.1 Primary Outcome/Endpoint 20

3.6.2 Secondary Outcomes/Endpoints 20

4 TRIAL DESIGN 24

4.1 Trial Overview 24

Schematic of Trial Design: 25

4.2 Use of a standardised weaning and extubation protocol 26

5 Trial Setting and Selection of SITES & Clinicians 27

5.1 Selection of Sites & Clinicians 27

6 Study Population 28

6.1 Inclusion Criteria 28

6.1.1 Definition of bronchiolitis 28

6.2 Exclusion Criteria 28

6.3 Co-enrolment Guidelines 29

7 Recruitment and Randomisation 30

7.1 Participant Identification and Screening 30

7.2 Informed Consent 30

For peer review only - http://bmjopen.bmj.com/site/about/guidelines.xhtml

7.2.1 Prospective Informed Consent Process 30
7.2.2 Consent for additional work 32
7.3 Randomisation Procedure 32
7.3.1 Randomisation System Failure 33
7.4 Blinding to Allocations 33
8 Participant Time Line, Assessments and Procedures 34
8.1 Administration of Intervention 34
8.1.1 Immediately prior to administration 34
8.1.2 Administration of allocated intervention 35
8.1.3 Post administration of allocated intervention 35
8.1.4 Weaning and extubation by protocol 35
8.2 Participant follow-up 36
8.3 Schedule for Intervention & Follow-up 37
8.4 Procedures for assessing Efficacy 39
8.4.1 Efficacy Assessment 1 39
8.4.2 Efficacy Assessment 2 39
8.5 Procedures for Assessing Safety 40
8.5.1 Procedure for Limited Physical Examination in BESS 40
8.6 Other Assessments 40
8.6.1 Parent Reported Quality of Life questionnaires 40
8.6.2 Special Assays or Procedures 41
8.7 Patient Transfer and Withdrawal 42
8.7.1 Patient Transfers 42
8.7.2 Withdrawal from the Trial 42
8.8 Loss to Follow-up 43
8.9 Trial Closure 43
9 Trial Treatment/interventions 44
9.1 Introduction 44
9.2 Arm A – Endotracheal Poractant Alfa at 200mg/kg (first dose), with up to two further doses at 100mg/kg 44
9.2.1 Formulation, Packaging, Labelling, Storage and Stability 44
9.2.2 Preparation, Dosage and Administration of Study Treatment/s 45
9.2.3 Specific Restrictions 46
9.2.4 Overdose 46
9.2.5 Temperature Compliance 46

For peer review only - http://bmjopen.bmj.com/site/about/guidelines.xhtml

9.2.6 Accountability Procedures for Trial Treatments 47
9.2.7 Assessment of Compliance with Study Treatment/s 48
9.3 Arm B – Placebo 48
9.4 Unblinding 48
9.4.1 Emergency Unblinding 49
9.4.2 Accidental unblinding 49
9.4.3 Unblinding at Trial Closure 49
9.5 Concomitant Medications 50
10 Safety Reporting 51
10.1 Terms and Definitions 51
10.2 Notes on Adverse Event Inclusions and Exclusions 52
10.2.1 Include: 52
10.2.2 Do Not Include: 52
10.2.3 Notification of deaths 53
10.3 Notes on Severity / Grading of Adverse Events 53
10.4 Relationship to Trial Treatment 54
10.5 Expectedness 54
10.6 Follow-up After Adverse Events 55
10.7 Time Period for Safety Reporting 55
10.8 Reporting Procedures 55
10.8.1 Flowchart for Reporting Requirements of Adverse Events 56
10.8.2 Non serious events (ARs/AEs) 58
10.8.3 Serious events (AEs/ARs/SUSARs) 58
10.9 Safety Reporting Responsibilities – Investigator 58
10.9.1 Maintenance of Blinding 59
10.10 Safety Reporting Responsibilities – CTRC 60
10.11 Safety reports 61
10.12 Urgent Safety Measures 61
10.13 Contact Details and Out-of-hours Medical Cover 61
11 Statistical Considerations 62
11.1 Introduction 62
11.2 Method of Randomisation 62
11.3 Sample Size calculation 62
11.4 Interim Monitoring and Analyses 62

For peer review only

For peer review only - http://bmjopen.bmj.com/site/about/guidelines.xhtml

11.5 Analysis Plan 63

12 exploratory & translational work; work packages b and c........ 64

12.1 Work Package B: Study of Parent & Staff Experiences........ 64

12.1.1 Parent and staff perspectives sub study analysis........ 65

12.2 Work Package C:........ 65

12.2.1 Metabolic profiling of surfactant composition and kinetics 66

12.2.2 Recovery of surfactant proteins A & D (SP-A & SP-D) is modulated by exogenous surfactant. 67

12.2.3 Lower airway inflammation is modulated by exogenous surfactant 67

12.2.4 Treatment efficacy or failure is associated with detection of specific pathogens........ 67

12.2.5 Mechanistic Protocol........ 68

13 Regulatory and Ethical Approvals 70

13.1 Statement of Compliance 70

13.2 Regulatory Approval 70

13.3 Ethical Considerations 71

13.4 HRA Approval........ 71

13.5 Ethical and Local Governance Approval 71

13.6 Protocol Deviation and Serious Breaches........ 72

13.7 Study Discontinuation 72

14 Data Management and Trial Monitoring........ 73

14.1 Source Documents 73

14.2 Data Capture Methods........ 73

14.3 Monitoring........ 74

14.3.1 Risk Assessment 74

14.3.2 Central Monitoring........ 74

14.3.3 Clinical Site Monitoring........ 75

14.4 Confidentiality 75

14.5 Quality Assurance (QA) and Quality Control (QC) 75

14.6 Records Retention........ 76

15 Indemnity........ 77

16 Roles and Responsibilities 78

16.1 Role of Study Sponsor and Study Funder........ 78

16.2 Funding and Support in Kind 78

16.3 Protocol Contributors........ 79

16.4 Trial Committees 80

For peer review only - http://bmjopen.bmj.com/site/about/guidelines.xhtml

16.4.1 Trial Management Group (TMG) 80
16.4.2 Trial Steering Committee (TSC) 80
16.4.3 Independent Data and Safety Monitoring Committee (IDSMC) 80
17 Publication and Dissemination........ 81
17.1 Publication Policy 81
17.1.1 Authorship........ 81
17.2 Dissemination to Key Stakeholders 81
17.1 Data Sharing 82
18 Chronology of Protocol Amendments 83
18.1 Version 1.0 (18/07/2018) 83
18.2 Version 2.0 (28/09/2018) 83
19 References 84
20 Documents Supplementary to the protocol 87

For peer review only

For peer review only - http://bmjopen.bmj.com/site/about/guidelines.xhtml

Glossary

AE	Adverse Event
AR	Adverse Reaction
BAL	Bronchoalveolar lavage
CI	Chief Investigator
CRF	Case Report Form
CTIMP	Clinical Trials of an Investigational Medicinal Product
CTRC	Clinical Trials Research Centre
CTU	Clinical Trials Unit
EUDRACT	European Clinical Trials Database
DSUR	Development Safety Update Report
FiO_2	Fraction of inspired oxygen
GP	General Practitioner
HRA	Health Research Authority
IB	Investigator's Brochure
IDSMC	Independent Data and Safety and Monitoring Committee
IMP	Investigational Medicinal Product
ISF	Investigator Site File
LRSQ	Liverpool Respiratory Symptom Questionnaire
MHRA	Medicines and Health Care Products Regulatory Agency
MV	Mechanical Ventilation
NIMP	Non Investigational Medicinal Product
NRES	National Research Ethics Service
NIHR CRN	National Institute for Health Research Clinical Research Network
OI	Oxygenation Index
OSI	Oxygen Saturation Index
PEEP	Positive End Expiratory Pressure
PI	Principal Investigator
PICANet	Paediatric Intensive Care Audit Network
PICU	Paediatric intensive care unit
PIP	Peak Inspiratory Pressure
PROM	Parent Reported Outcome Measure
QA	Quality Assurance
QC	Quality Control

For peer review only

For peer review only - http://bmjopen.bmj.com/site/about/guidelines.xhtml

R&D	Research & Development
REC	Research Ethics Committee
RN	Research Nurse (Registered)
RSI	Reference Safety Information
RSO	Research Support Office
SAE	Serious Adverse Event
SAR	Serious Adverse Reaction
SDV	Source Data Verification
SMPC	Summary of Product Characteristics
SOP	Standard Operating Procedure
SpO_2	Peripheral oxygen saturation
SSAR	Suspected Serious Adverse Reaction
SUSAR	Suspected Unexpected Serious Adverse Reaction
TMG	Trial Management Group
TMP	Trial Monitoring Plan
TSC	Trial Steering Committee
UAR	Unexpected Adverse Reaction
VI	Ventilation Index
WP-A	Work package A “The Trial”
WP-B	Work package B “Exploration of parental experience”
WP-C	Work package C “Mechanistic sub-studies”

For peer review only

For peer review only - http://bmjopen.bmj.com/site/about/guidelines.xhtml

2 PROTOCOL SUMMARY

**Full Title**	**The efficacy and mechanism of surfactant therapy for critically ill infants with bronchiolitis: The Bronchiolitis Endotracheal Surfactant Study (BESS). A phase-2 blinded randomised air-placebo-controlled trial of endotracheal surfactant (poractant alfa) in critically ill infants with bronchiolitis**
**Acronym**	**BESS**
**Phase**	2
**Target Condition**	Bronchiolitis of infancy requiring conventional invasive Mechanical Ventilation (MV)<br>Diagnosis of bronchiolitis per clinical criteria defined in national guidance NICE-NG9.
**Sample size**	284
**Main Inclusion Criteria**	1. Term-born infants < 26 weeks old and preterm-born infants < 26 weeks corrected age<br>2. Diagnosis of bronchiolitis<br>3. Requires conventional invasive MV via tracheal intubation<br>4. Parent or person with parental responsibility has given written informed consent for trial participation
**Main Exclusion Criteria**	1. Major congenital anomalies<br>2. Congenital neuromuscular disease<br>3. Already intubated for MV for >48 hours or likely to have been intubated for MV for >48 hours by randomisation<br>4. Have received or are receiving extracorporeal membrane oxygenation (ECMO) or oscillation during this episode of bronchiolitis<br>5. Have received or are receiving intratracheal administration of any surfactant during this episode of bronchiolitis<br>6. Receiving MV for primary apnoea rather than respiratory failure<br>7. A known hypersensitivity to the active substance or excipients of poractant alpha (Curosurf®)<br>8. A decision to wean to extubation has already been made<br>9. Clinical judgement of futility
**Study Centres and Distribution**	Approximately 14 Paediatric Intensive Care Units (PICUs) in UK tertiary hospitals.
**Patient Study Duration**	Duration of treatment: 24 hours (a maximum of three 12hourly intratracheal doses of surfactant; first dose (0hrs), second dose at 12hrs and third at 24hrs).

For peer review only - http://bmjopen.bmj.com/site/about/guidelines.xhtml

		Duration of follow-up: up to a maximum of 12 months, minimum 90 days.	
**Overall Study duration**		44 months	
**Agent/ Intervention**		**Intervention:** Endotracheal poractant alfa (Curosurf®, porcine lung phospholipid fraction supplied at 80mg/mL), first dose 200mg/kg, repeated 12 hourly at 100mg/kg, to a maximum of 3 doses according to the manufacturer's summary of product characteristics (SPC). Unlicensed use (UK licence, new indication).<br>**Control:** An identical series of up to three procedures (while intubated) using air as the placebo.	
		**Objectives**	**Outcome Measures**
**Work Package A**	**Primary**	To determine the efficacy of endotracheal surfactant in reducing total duration of MV by 18 hours or more, by random allocation of infants to receive either up to 3 doses of poractant alfa or up to 3 doses of air-placebo in an identical administration procedure (the Trial = Work package A)	The Primary Outcome measure for the Trial will be total duration of MV (hours) from randomisation to final extubation. We will include time off MV due to failed extubation.<br>Secondary Outcome measures relating to the Primary Objective of the trial are listed in the Protocol.
	**Secondary**	To describe the impact of intervention on infants' long-term respiratory symptoms, using a parent reported outcome measure	The Liverpool Respiratory Symptom Questionnaire applied at 6m and 1yr after intervention in patients recruited in seasons 1 and 2.
**Work Package B**	**Exploratory/ Translational:**<br>**Study of Staff & Parent Experiences**	In a subgroup of patients, to explore staff and parent experiences of trial recruitment, consent and conduct in season 1 to inform season 2.	Outcome Measures relating to Exploratory and Translational aspects of the study are listed where known in section 12. Due to the exploratory nature of this work, other outcome measures are likely to be identified over this course of the study and after the trial has completed.
**Work Package C**	**Exploratory/ Translational:**<br>**Mechanistic Study**	In a subgroup of patients to explore the mechanisms for treatment efficacy and failures at a patient level by describing associations between trial outcome with markers of infection, inflammation, and surfactant composition in patient Bronchoalveolar Lavage Fluid.	

NB: The Exploratory and Translational parts of the study (Work Packages B & C) will not inform the primary or secondary outcomes of the main trial. They are only included as an annexe to the

For peer review only

For peer review only - http://bmjopen.bmj.com/site/about/guidelines.xhtml

Trial Protocol for regulatory purposes related to the Research Ethic Committee, Health Research Authority and Local Institutional Research permissions.

For peer review only

For peer review only - http://bmjopen.bmj.com/site/about/guidelines.xhtml

3 INTRODUCTION

3.1 Study Overview

The study "The efficacy and mechanism of surfactant therapy for critically ill infants with bronchiolitis" will have the short title "Bronchiolitis Endotracheal Surfactant Study" and the acronym BESS.

The award supporting BESS supports three work packages. These are: the trial (Work package A; WP-A); a study of staff and parental experience (Work package B; WP-B) and mechanistic exploratory sub-studies (Work package C; WP-C).

Work package A is subject to Medicines for Human Use (Clinical Trials) Regulations 2004 (as amended) and requires regulatory permission from the Medicines and Healthcare products Regulatory Agency (MHRA). Work packages B and C are dependent upon trial activity but do not provide outcome measures for the trial and so are not subject to these regulations. The study, which includes all work packages, requires REC, HRA and NHS permissions for which this unified protocol document is required. The detail of Work Packages B and C are described in Section 12.

3.2 Background

Bronchiolitis of infancy is a seasonal viral disease of the lower airways that most commonly causes feeding difficulties and respiratory distress and in severe cases causes a significant public health burden due to hospitalisation and mortality(1). It is the single most common reason for hospital admission of infants (children age<1yr). The youngest of these and those born prematurely are most often and most severely affected (1).

A recent study of Hospital Episode Statistics for England found 25,000 cases of bronchiolitis from each annual birth cohort (40.4 per 1000 (95% CI 38.5 to 42.3)) were admitted to hospital in their first year of life (2). Using PICANet data, the same study found just over 1000 of these infants were admitted with critical illness to English paediatric intensive care units (PICUs) each year (95% CI 1.3 to 1.6 per 1000 infants/yr) for mechanical ventilation (MV). This accounted for 12% of all admissions to PICUs. The infants' modal age was 1 month; the mean duration of PICU admission was 6.1 days (95% CI 5.4 to 6.7 days), and their case-fatality rate was 1.75%.

Despite advances in the provision of non-invasive modes of respiratory support, admissions to PICU and duration of MV for life-threatening bronchiolitis have remained static for the past 4 years (2). Excluding infants with rare congenital co-morbidities (mostly cardiac & neuromuscular anomalies), infants requiring MV for bronchiolitis do so for a mean of 4.8 days (SD 2.5d) (PICANet data, 2011-14).

Existing Research

In 2015 Jat & Chawla (3) found on pooled analysis of three small studies a significant reduction in length of PICU stay between endotracheal surfactant treated and control groups (mean difference (MD) -1.81d (95%CI -2.42 to -1.19) of critically ill infants with bronchiolitis who required MV. There were sustained improvements of arterial oxygenation (PaO_2) and carbon dioxide ($PaCO_2$) elimination (4-6). No adverse effects or complications were observed in the three studies. The authors of this systematic review and meta-analysis concluded that use of surfactant had favourable effects but that larger trials with adequate power were required to evaluate the effectiveness of surfactant therapy in critically ill infants with bronchiolitis who required MV.

For peer review only - http://bmjopen.bmj.com/site/about/guidelines.xhtml

3.3 Rationale

There is no vaccine or specific treatment for bronchiolitis. Care is supportive as described in National Guidance (17). Breathing fails in very severe cases, and these babies need intensive care with breathing being supported by a machine called a ventilator, hence known as mechanical ventilation (MV).
Pulmonary surfactant is secreted by lung alveolar cells to reduce surface tension so increasing compliance of the lungs, allowing them to inflate more easily and reduce the work of breathing (8). Surfactant proteins A and D have innate antiviral immune properties as collectins (9).
Studies of infants with life-threatening bronchiolitis show reduced lung compliance and surfactant deficiency (10-14). These features are similar to the respiratory distress syndrome of infants born prematurely. Premature infants are routinely treated with animal-derived surfactant to improve lung compliance, and reduce mortality, morbidity and duration of MV (15).
Most infants with severe bronchiolitis are very young and/or born prematurely. They present at modal age 1 month, with 62.2% age <2months (2). We hypothesise that the effect of exogenous surfactant on infants with severe bronchiolitis may be similar to the therapeutic effect on those born prematurely as both conditions present with surfactant deficiency. At present surfactant is used without evidence as rescue therapy in some infants and young children with the most severe respiratory failure who are difficult to manage even with MV.
The systematic reviews by Jat & Chawla found that surfactant therapy in infants with critical illness due to bronchiolitis was: safe, improved gas exchange and reduced both duration of MV and length of stay on PICU (3, 16).
There are no other relevant trials registered on Clinicaltrials.gov, EU Clinical Trial Register or ISRCTN [search “surfactant” AND “bronchiolitis”, 6-JUL-2018].
This topic has been identified by the National Institute for Health Research (NIHR) Maternal Neonatal and Child Health panel in 2013 and selected by the Prioritisation Group for commissioning by both the Health Technology Assessment (HTA) and Efficacy and Mechanism Evaluation (EME), and has the support of the NIHR Translational Research Partnership (TRP) for Inflammatory Respiratory Diseases and UK Paediatric Intensive Care Society Study Group (PICS-SG).

Research Question

Does surfactant therapy reduce duration of invasive mechanical ventilation when given to infants early in the course of critical illness due to bronchiolitis?

Intervention

The intervention being trialled is:
Endotracheal poractant alfa (Curosurf®, porcine lung phospholipid fraction supplied at 80mg/mL), first dose 200mg/kg, repeated 12 hourly at 100mg/kg, up to a total of 3 doses according to the manufacturer's summary of product characteristics (SPC).
Or-
An identical series of up to three procedures (while intubated) using air as the placebo.

Hypothesis

The hypothesis we will test in the trial is “Endotracheal surfactant reduces duration of mechanical ventilation by 18 hours

For peer review only - http://bmjopen.bmj.com/site/about/guidelines.xhtml

3.4 Risk and Benefits

3.4.1 Potential Benefits

The potential benefit of the therapy to the participants is a sustained improvement in gas exchange leading to a reduction of total duration of MV. The potential benefit to the participant is a reduction of time in a technology-dependent vulnerable state, reduced risk of MV associated morbidity, reduced exposure to sedation and muscle paralysing drugs and a more rapid return to usual parental care. This in turn would reduce parental anxiety. Also, freeing up PICU beds during winter surge offers savings and opportunity to use resources for other conditions.

3.4.2 Potential Risks

Administration of poractant alfa (porcine derived surfactant, Curosurf®) in infants with bronchiolitis is well tolerated and considered safe(3). Transient minor adverse reactions associated with administration of poractant alfa for the treatment of neonatal Respiratory Distress Syndrome (RDS) include bradycardia, hypotension, endotracheal tube blockage, and oxygen desaturation. The risk of such events is minimised by tracheal toilet prior to administration of poractant alfa, as recommended in the Summary of Product Characteristics.

Small volume (2x1mL/kg) non-bronchoscopic bronchoalveolar lavage (BAL) procedure on PICU is safe and well tolerated procedure(7). In a published audit of 27 children on PICU who underwent multiple small volume BAL installations (5x1mL/kg) as part of a single routine diagnostic procedure, 7 experienced transient oxygen desaturation requiring temporary increased FiO_2. We will limit BAL sampling to a 2x1mL/kg instillation at each scheduled assessment.

Small volume (1.25mL (range 1.0 to 1.3mL)) blood sampling will be done on infants ventilated at Liverpool and Southampton PICUs and scheduled to coincide with routine blood sampling. Existing vascular access will be used when available. There is no risk of anaemia associated with this limited volume of blood sampling. The sample will be taken from an arterial line if present. If not by standard venesection.

Capillary blood gas sampling (100microliters) or arterial blood gas sampling for subject monitoring will use data from routine blood gas sampling when either is collected within a 2-hour window of a research-scheduled sample. As this is part of standard care no additional risks are expected.

3.5 Aims & Objectives of Work Package A

3.5.1 Aims

1. To conduct a multicentre blinded randomised placebo-controlled phase-2 trial of the endotracheal surfactant poractant alfa (CUROSURF®) as therapy for bronchiolitis in critically ill infants.
   Hypothesis to test: Endotracheal surfactant reduces duration of mechanical ventilation by 18 hours (Work package A (WP-A)).
2. To evaluate the safety profile in the two treatment groups

For peer review only - http://bmjopen.bmj.com/site/about/guidelines.xhtml

3.5.2 Primary Objective

To determine the efficacy of endotracheal surfactant in reducing total duration of MV by 18 hours, by random allocation of infants to receive up to 3 doses of poractant alfa or up to 3 doses of air-placebo in an identical administration procedure.

3.5.3 Secondary Objective(s)

1. To describe the impact of intervention on infants' long-term respiratory symptoms
2. To describe secondary outcomes of efficacy including physiological indices and duration of other modes of respiratory support.
3. To assess the safety of the intervention for infants with life-threatening bronchiolitis

3.6 Outcome measures/endpoints

3.6.1 Primary Outcome/Endpoint

The primary outcome measure will be total duration of MV (hours) from randomisation to final extubation. We will include time off MV due to failed extubation.
Justification: This outcome measure is clinically important, was suggested in both HTA and EME commissioning calls and is valued by our Parent Advisory Group. Reduced duration of MV reduces ventilator-associated morbidity, sedation, muscle relaxants, healthcare costs and bed occupancy at a time of increased seasonal pressure.

3.6.2 Secondary Outcomes/Endpoints

The secondary outcome measures for the trial (WP-A) are:
For efficacy:

1. duration of post-extubation non-invasive respiratory support (all forms of non-invasive ventilation: e.g. nasal CPAP and nasal high low oxygen)
2. duration of oxygen supplementation
3. number of trial interventions given (both poractant and placebo)
4. use of steroids to assist extubation
5. duration of stay on PICU and in hospital;
6. change over time from baseline of Ventilation Index (VI), Oxygenation Index (OI) and Oxygenation Saturation Index (OSI) while mechanically ventilated, & SpO_2/FiO_2 (SF) ratio.[1]
7. the score (value) of parent reported outcome measure of respiratory symptoms the "Liverpool Respiratory Symptom Questionnaire" (LRSQ) at 6m (+/-1m) and 12m (+/-1m) (20);

[1] VI and OI are validated physiological measurements reflecting respiratory function (gas exchange) normalised for level of respiratory support provided by MV, but require blood sampling(21). OSI is a validated physiological measurement reflecting respiratory function (gas exchange) normalised for level of respiratory support provided by MV that does not require blood sampling (41). The SF ratio is a non-invasive measure of respiratory function and can be calculated in situations where invasive blood gas monitoring and MV has ceased (22).

For peer review only - http://bmjopen.bmj.com/site/about/guidelines.xhtml

8. time from randomisation to meeting study criteria for readiness for Spontaneous Breathing Test

For safety:

1. failure to administer the intervention due to any adverse event during preparatory processes (tracheal toilet or BAL)
2. failure to complete administration of the intervention due to any adverse event during administration of the intervention (regardless of which arm of allocation)
3. incidents of ‘air leak’ (pneumothorax and pneumomediastinum) occurring before discharge from PICU
4. other adverse events and serious adverse events associated with the intervention
5. any need to replace the endotracheal tube
6. parent reported readmission to hospital (all causes) up to 90 days post randomisation
7. death during PICU admission
8. all-cause mortality at 90 days post randomisation

For peer review only

For peer review only - http://bmjopen.bmj.com/site/about/guidelines.xhtml

Table 1: Objectives and Outcome Measures

**Objectives**	**Outcome Measures**	**Timepoint(s) of evaluation of this outcome measure (if applicable)**
**Primary Objective** To determine the efficacy of endotracheal surfactant in reducing total duration of MV by 18 hours or more, by random blind allocation of infants to receive either up to 3 doses of poractant alfa or up to 3 doses of air-placebo in an identical administration procedure.	Total duration of MV (hours) from randomisation to final extubation, including any time off MV due to failed extubation.	N/A
**Secondary Objective 1** To describe the impact of intervention on infants' long term respiratory symptoms	The score (value) of patient reported outcome measure of respiratory systems in the "Liverpool Respiratory Symptom Questionnaire" (LRSQ)	LRSQ at 6 months and 12 months post randomisation
**Secondary Objective 2** To describe secondary outcomes of efficacy including physiological indices and duration of other modes of respiratory support	Time from randomisation to meeting study criteria for readiness for Spontaneous Breathing Test	N/A
	Number of trial interventions given	N/A
	Change over time from baseline of Ventilation Index (VI), Oxygenation Index (OI) and Oxygenation Saturation Index (OSI) while mechanically ventilated, & SpO_2/FiO_2 (SF) ratio	Capillary blood gas sampling immediately prior to intervention and then at 6, 12, 24, 36 and 48hrs while still on MV; to continue daily after 48hrs if still on MV.
	Duration of oxygen supplementation	N/A
	Use of steroids to assist extubation	N/A

For peer review only - http://bmjopen.bmj.com/site/about/guidelines.xhtml

	Duration of post-extubation non-invasive respiratory support	N/A
	Duration of stay on PICU and in hospital	N/A
**Secondary Objective 3** To assess the safety of the intervention for infants with life-threatening bronchiolitis	Failure to administer the intervention due to any adverse event during preparatory processes (tracheal toilet or BAL)	Randomisation to 24 hours after final trial intervention
	Failure to complete administration of the intervention due to any adverse event during administration of the intervention (regardless of which arm of allocation)	Randomisation to 24 hours after final trial intervention
	Incidents of ‘air leak’ (pneumothorax and pneumomediastinum) occurring before discharge from PICU	Randomisation to discharge from PICU
	Other adverse events and serious adverse events associated with the intervention	Randomisation to 24 hours after final trial intervention
	Any need to replace the endotracheal tube	Randomisation to successful extubation
	Parent reported readmission to hospital (all causes) up to 90 days post randomisation	PICU discharge to 90 days post-randomisation
	Death during PICU admission	Randomisation to PICU discharge
	All-cause mortality at 90 days post randomisation	PICU discharge to 90 days post-randomisation

For peer review only - http://bmjopen.bmj.com/site/about/guidelines.xhtml

4 TRIAL DESIGN

4.1 Trial Overview

The BESS trial is a phase-2 blinded randomised air-placebo-controlled trial of endotracheal surfactant (poractant alfa) in critically ill infants with bronchiolitis (we will refer to this as Work Package A, or WP-A).

For peer review only

For peer review only - http://bmjopen.bmj.com/site/about/guidelines.xhtml

Schematic of Trial Design:

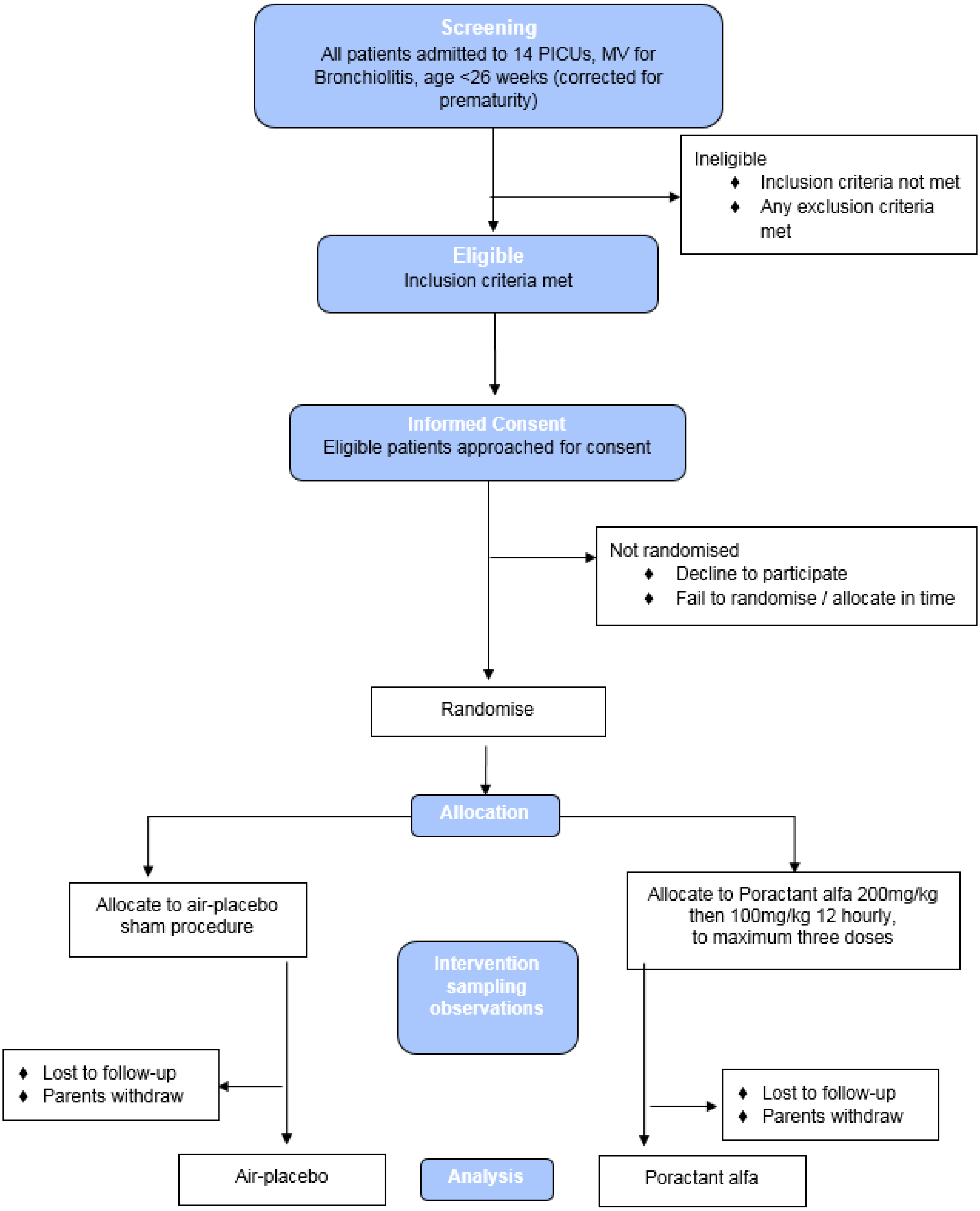

Figure 1: Schematic of Trial Design

For peer review only - http://bmjopen.bmj.com/site/about/guidelines.xhtml

4.2 Use of a standardised weaning and extubation protocol

On the direction of the NIHR EME board, the BESS trial protocol will include a standardised protocol for weaning from mechanical ventilation and extubation.
Weaning and extubation by protocol will standardise this aspect of care across sites and in both arms of BESS. Infants in BESS will be extubated after they meet specified physiological criteria that indicate the infant is ready for a Spontaneous Breathing Test (SBT) and pass that test, (see section 8.1.2.1 for further details).

For peer review only

For peer review only - http://bmjopen.bmj.com/site/about/guidelines.xhtml

5 TRIAL SETTING AND SELECTION OF SITES & CLINICIANS

Approximately fourteen Paediatric Intensive Care Units (PICUs) in the United Kingdom that routinely mechanically ventilate critically ill infants with high caseloads of bronchiolitis will recruit to BESS. PIs for sites that have provisionally agreed to participate in BESS have shared local activity data, commented on the application and are recognised as co-applicants on the application award. The PIs will champion the study at the sites and take local responsibility for the conduct of the trial. PIs will identify local co-Investigators.

5.1 Selection of Sites & Clinicians

Criteria for the selection of centres includes

- NHS Hospitals in the UK providing paediatric intensive care
- Historic high case load of bronchiolitis, as reported to PICANet
- Sufficient research capacity comprised of staff, time and facilities to undertake the trial; including patient screening and recruitment, randomisation, collection and provision to the CTRC of all required data, collection of research samples, identification and management of adverse events including notification to CTRC within protocol defined timeframes, identification and provision of information to the CTRC of all protocol breaches.
- All staff contributing to the trial must have valid certified Good Clinical Practice (GCP) training throughout the conduct of the trial. Requirements of the Sponsor are that renewal of GCP must occur every 3 years.
- Willingness to participate

Final decisions on site selection will be determined by the Trial Management Group (TMG) and will be described in the supplementary document 'BESS Site Suitability Assessment.
Initiation of centres will be undertaken in compliance with CTRC SOPs[2]. Centres fulfilling the criteria will be selected to be recruitment centres for the BESS trial and will be opened to recruitment upon successful completion of all global (e.g. MREC and MHRA) and study-specific conditions (e.g. site personnel training requirements) and once all necessary documents have been returned to CTRC as detailed in the trial Green Light checklist.

Participating centres will be listed in the 'BESS Participating Centres' log, maintained separately to the protocol and stored in the Trial Master File (TMF).

[2] CTRC SOP TM017 Study Initiation in CTRC and TM018 Study Initiation at Sites

For peer review only - http://bmjopen.bmj.com/site/about/guidelines.xhtml

6 STUDY POPULATION

6.1 Inclusion Criteria

1. Term-born infants < 26 weeks old and preterm-born infants < 26 weeks corrected age†
2. Diagnosis of bronchiolitis (see below)
3. Requires conventional invasive MV via tracheal intubation
4. Parent or person with parental responsibility has given written informed consent for trial participation

Note: Infants with chronic lung disease of prematurity can be included in the trial.
† Correcting for prematurity is a well-established method for adjusting assessment of infants born prematurely who are anatomically, physiologically, immunologically and neuro-developmentally immature for their chronological age. In our study, term-born infants will be eligible for inclusion in the study up to chronological age of 26 weeks old (i.e. all those under 6 months old). Premature born infants have their age corrected to account for weeks of lost gestation, hence an infant born 12 weeks premature (i.e. before completing the 28th week of the 40 weeks of pregnancy) would still be eligible up to a chronological age of 38 weeks old (i.e. under 9 months), because that infant is still less than 26 weeks old when age is corrected for the premature birth.

6.1.1 Definition of bronchiolitis

Diagnosis of bronchiolitis will be made using clinical criteria defined in national guidance NICE-NG9 (17).

6.2 Exclusion Criteria

1. Major congenital anomalies
2. Congenital neuromuscular disease
3. Already intubated for MV for >48 hours or likely to have been intubated for MV for >48 hours by randomisation
4. Have received or are receiving extracorporeal membrane oxygenation (ECMO) or oscillation during this episode of bronchiolitis
5. Have received or are receiving intratracheal administration of any surfactant during this episode of bronchiolitis
6. Receiving MV for primary apnoea rather than respiratory failure
7. A known hypersensitivity to the active substance or excipients of poractant alpha (Curosurf®)
8. A decision to wean to extubation has already been made
9. Clinical judgement of futility

For peer review only - http://bmjopen.bmj.com/site/about/guidelines.xhtml

6.3 Co-enrolment Guidelines

The investigators are happy to enter in to co-enrolment agreements where feasible.
Where other trials are taking place at a site participating in BESS and the target population overlaps, sites should make the BESS team aware at the earliest opportunity so that the potential for co-enrolment can be discussed between the two trial teams. The BESS investigators are open to co-enrolment with other studies but this must be agreed between both Chief Investigators prior to randomisation into BESS.

For peer review only

For peer review only - http://bmjopen.bmj.com/site/about/guidelines.xhtml

7 RECRUITMENT AND RANDOMISATION

7.1 Participant Identification and Screening

All patients admitted to recruiting PICUs, receiving conventional MV for bronchiolitis, age <26 weeks (corrected for prematurity) will be screened for eligibility before trial entry. Screening and confirmation of full eligibility will be completed to allow randomisation within 48 hours of intubation.

A screening log of patients who are assessed for eligibility but not randomised will be maintained and returned to the CTRC on a regular basis, as this will provide important information for monitoring purposes. The screening log will capture which patients have been screened for the trial but assessed as ineligible, and those considered eligible and approached for consent but for whom consent was not obtained; reasons (all per subject) for non-inclusion will be recorded. Reasons for declining to participate will be asked routinely but it will be made clear that parents/persons with parental responsibility do not have to provide a reason unless happy to do so.

The screening log will not contain patient identifiers as those who are not eligible will not have provided their consent for identifiers to be captured as part of the trial. Therefore, sites will maintain their own trial register linking patients screening and/or randomisation numbers to their identifiers. Under no circumstances will this log be returned to the CTRC.
Patients will be reviewed for eligibility once they have been intubated for MV, or as soon as their arrival into PICU if they are already intubated. Parents/persons with parental responsibility for patients who meet the eligibility criteria and are suitable for inclusion (see 6.1 and 6.2) will be provided with information about the trial (both verbal and written) to consider participation.

Only a medically qualified doctor authorised on the site Delegation Log can confirm full eligibility of any patient. A record of this confirmation must be made in the patient's medical notes on the date of confirmation of eligibility.

7.2 Informed Consent

7.2.1 Prospective Informed Consent Process

Parents and those persons with parental responsibility may give consent for the patient to take part in BESS; hereafter throughout this protocol they shall be referred to as 'parent(s)'.
Initiation of the intervention in BESS is urgent and the condition "critical illness due to bronchiolitis requiring MV" is a medical emergency. Randomisation must be completed within 48 hours of intubation for MV, which will limit the time available for a parent to consider whether or not their child should participate. Site research teams will use their understanding of the clinical situation for each individual patient to judge when is most appropriate to approach parents about trial participation. The trial concept will be introduced to a parent by familiar members of the usual clinical care team, be they nurses or physicians, as per the Canadian Critical Care Trials PICU Research Group trial recruitment model (24).
If a parent is interested, a member of the usual clinical care team will make an introduction to the research staff who will provide written study information and offer further verbal information.

For peer review only - http://bmjopen.bmj.com/site/about/guidelines.xhtml

Due to the time-critical nature of the intervention, the parent will have a limited timeframe to ask questions and make a decision about their child's participation in the trial. Randomisation must take place within 48 hours of intubation for MV and while some parents may have this full timeframe to consider trial participation, others may have considerably less due to time of arrival on PICU, time of approach etc. Consent will be sought by any appropriately trained and delegated medical staff and where local site policy allows, research nurses or respiratory physiotherapists (the research team).

Consent from the parent must be obtained prior to every infant participating in the trial. This consent will be sought after a full explanation has been given of the treatment options; including the conventional and generally accepted methods of treatment, the objectives, risks and inconveniences of the trial, and the conditions under which it is to be conducted.

Parent Information Sheet and Consent (PISC) forms, describing in detail the trial intervention, procedures and risks will be approved by a research ethics committee (REC). The parent will be asked to read and review this document. Upon reviewing the document, the person seeking consent will explain the research study to the parent. This information, and the supporting conversation, will emphasise that participation in the trial is voluntary and that the parent may withdraw their infant from the trial at any time and for any reason.

The parent will be given opportunity to ask any questions that may arise and time to consider the information, within the constraints of the 48 hour period between intubation and randomisation, prior to agreeing to participate. A contact point where further information about the trial may be obtained will be provided on the parent information sheet.

The parent will then sign and date the consent form if they agree to their infant participating. Both the person obtaining consent and the parent must sign and date the consent form; the researcher and parent must sign at the same time, with the researcher completing their signature **after** the parent.

A copy of the signed PISC will be given to the parent for their record. The original copy will be filed in the Investigator Site File, a copy filed in the participant's medical notes and a further copy should be sent to the CTRC. If the site has moved to an electronic health record (EHR), a scanned copy should be entered in the patient notes. A record of the consent process should also be recorded in the participant's medical notes.

The right of the parent to refuse consent for the infant to participate in the trial without giving reasons must be respected. After the infant has entered the trial, the clinician will remain free to give alternative treatment to that specified in the protocol, at any stage, if s/he feels it to be in the best interest of the patient. However, the reason for doing so should be recorded and the infant will remain within the trial for the purpose of follow-up and data analysis.

Similarly, the parent of the infant remains free to withdraw the infant at any time from the protocol treatment and trial follow-up without giving reasons and without prejudicing the further treatment of the infant (see section 8.6.2 for further details). They do not need to provide a reason for withdrawing their infant from the trial, which will be emphasised in the PISC. Any parent who withdraws their infant from the trial will be approached for participation in the sub-study of parental experience (WP-B), unless they previously declined sub-study participation at consent (see Section 12).

For peer review only - http://bmjopen.bmj.com/site/about/guidelines.xhtml

7.2.2 Consent for additional work

Parents of eligible infants who have been approached to give consent for the BESS trial will also be offered the opportunity to take part in a telephone interview about their experiences of the consent process. This will be offered regardless of whether or not consent was given for the infant to participate in BESS (see section12.1).

An alternative PISC will be offered to parents at Liverpool and Southampton PICUs (sites participating in the Mechanistic work exploring surfactant metabolism; WP-C. See section 12.2).

A decision to decline to participate in either WP-B or WP-C will not impact upon care, or preclude involvement in the trial (WP-A).

7.3 Randomisation Procedure

Participants will be randomised in a 1:1 ratio, stratified by site and duration of ventilation prior to randomisation (<24hrs, ≥24hrs).

Randomisation must be completed within 48 hours of intubation.

Participants will be randomised using a secure 24-hour web-based randomisation and drug supply programme, managed by a third party subcontracted by Chiesi Farmaceutici S.p.A. (Chiesi). A personal login username and password will be required to access the randomisation system. Designated research staff will be issued with a unique personal login and password upon completion of training in the use of the system.

When the system requirements (confirmation of consent and eligibility) are confirmed the participant treatment allocation and a unique study number (randomisation number) will be displayed on a secure webpage. The randomising staff member must select the partially completed unblinding envelope corresponding to the participant's randomisation number (as clearly displayed on the front of the envelope) and record the allocation on the printed slip inside. The unblinding envelope must then be sealed and transferred to an agreed secure location accessible to PICU staff.

Once a randomisation number has been generated, the randomising staff member will be able to enter the resupply module within the system, where they will select the participant weight category and dose number (i.e. first, second or third) for the system to allocate the required number of vials for the participant.

Sites must complete this step for participants in both arms of the trial and for every given dose (first, second or third) regardless of allocation to drug or placebo.

The randomisation and resupply system is configured to allow remote monitoring of stock levels at site by the distributor contracted by Chiesi, and further IMP stocks will be supplied once the amount available at site reaches an agreed trigger level. However, it remains the responsibility of the PI or delegated research staff to check the supplies available on the PICU prior to randomisation to ensure there is sufficient supply of the IMP, as the majority of stock will be held at the local site Pharmacy (see Section 9.2.6 for further information regarding intervention accountability).

For peer review only - http://bmjopen.bmj.com/site/about/guidelines.xhtml

Unblinded and appropriately delegated site staff will be able to log into the randomisation and resupply system at any time and view the allocations of each participant to ensure continuity between shifts.

7.3.1 Randomisation System Failure

In the event of a randomisation system failure, the centre should contact the coordinating team at the CTRC (Monday to Friday between 9:00 to 17:00 excluding bank holidays) to inform them of the problem. Out-of-hours the centre should contact the Chief Investigator or his delegates, on 07506 653 560.

7.4 Blinding to Allocations

BESS will use an established strategy of a sham-procedure to blind as many research and usual care staff as possible to the nature of the intervention. This sham procedure was used in the development of surfactant as a therapy for surfactant deficiency of prematurity (6,19).

The participants, CTRC staff (excluding statistical team members as appropriate), and all members of the site research teams (excluding agreed randomising staff member, the administrator of the intervention and the dosage witness) will be blinded to treatment allocations. Occasional transient adverse effects associated with surfactant administration and back-tracking of surfactant up the endotracheal tube, plus the more frequently observed positive effects of rapid improvement in gas exchange immediately following surfactant administration, may result in some blinded care givers being able to infer that a participant has been allocated to the surfactant arm of the trial.
The randomising person, person drawing up the surfactant or air placebo and a second person checking the dose will be unblinded. Due to the transient effects and back-tracking it is likely that the Usual Care Nurse (UCN) will become unblinded, and we therefore recommend that if possible the UCN draws up the surfactant or acts as the witness to the drawing up, to minimise the number of people unblinded. Please see figure 2 for an intervention flowchart showing which staff/steps are blinded and unblinded.
Staff who are unblinded due to their involvement or believe themselves to have become unblinded for any reason are strongly discouraged from discussing this with other members of the team, unless the effect noted is causing an adverse event that requires action. Any staff member who believes themselves to have become unblinded must excuse themselves from any decision making regarding the weaning and extubation of the infant. The need to maintain blinding will be emphasised during all Site Training Visits.

For peer review only

For peer review only - http://bmjopen.bmj.com/site/about/guidelines.xhtml

8 PARTICIPANT TIME LINE, ASSESSMENTS AND PROCEDURES

8.1 Administration of Intervention

The first trial intervention must be administered within 2 hours of randomisation with subsequent doses (if required) at 12 and 24 hours post randomisation. A grace period of one hour either side of the anticipated administration time is permitted.

- Randomisation; **T0**
- First intervention must be given within two hours; between **T0** and **T2**
- Second intervention should be given 12 hours after randomisation; at **T12** (+/- 1 hour)
- Third intervention should be given 24 hours after randomisation; at **T24** (+/- 1 hour)

8.1.1 Immediately prior to administration

A limited physical examination, with minimal handling, must be undertaken in the two hours prior to each administration of the trial intervention (as per Section 8.5.1).

Prior to intervention and in a drug room away from usual staff, the study respiratory physiotherapist, research nurse or other delegated, unblinded staff member will:

- Draw up a dose of poractant alfa or an equal volume of air as placebo according to the random allocation into a syringe
- Ensure a second person is present to witness this process and check the dose volume
- Cover the syringe with an opaque paper shield to maintain blinding of other staff and parents
- Take the syringe to the bedside, along with syringes of sterile 0.9% sodium chloride for tracheal toilet and the BAL sampling set.

Infants in both the treatment and placebo arms of the trial will have 0.9% sodium chloride “normal saline” tracheal toilet and 0.9% sodium chloride BAL immediately prior to administration of the intervention. Both can cause immediate transient desaturation, need for increased sedation but also and more often rapid improvement in gas exchange when obstructing secretions are removed. Tracheal toilet and BAL can also lead to creamy white secretions appearing in the endotracheal tube, and this will be observed in both treatment and placebo groups.

Staff should remind parents, if present, of the purpose of the imminent study procedure and give them the opportunity to remain or leave as they choose. The usual care nurse will remain to care for the participant and assist as required.

Tracheal toilet is usually required for babies with bronchiolitis and will be done as recommended in the SPC before administration of poractant alfa. BAL sampling with 0.9% sodium chloride (2x1mL/kg) will be done if scheduled (section 8.2). Both tracheal toilet and BAL sampling is done by catheter access to the trachea; either via the endotracheal suction port without disconnecting the

For peer review only - http://bmjopen.bmj.com/site/about/guidelines.xhtml

infant from the ventilator or by disconnecting the infant from the ventilator and performing hand bagging while a side-port with one-way valve are utilised for the toilet and BAL.

The use of muscle relaxant prior to tracheal toilet, BAL and or administration of the intervention is at clinical discretion.

8.1.2 Administration of allocated intervention

The intervention will be given per either method described in the SPC; either keeping the infant connected to the ventilator or momentarily disconnecting and performing hand-bagging (at the attending Respiratory Physiotherapist's decision based on patient status).

- Connect the syringe containing the surfactant or air-placebo to a suitable catheter (size 5F);
- Pass the catheter through the suction or side port into the endotracheal tube and advance the catheter, aiming to have the tip of the catheter placed just beyond the tip of the endotracheal tube
- Deliver the intervention (surfactant or air-placebo) slowly as a single bolus (recommended over 0.5-3 minutes) directly into the lower trachea
- The intervention (surfactant or air-placebo) will disperse through mechanical ventilation cycles into the peripheral lung tissue where the excipient (sterile buffered 0.9% sodium chloride) is absorbed in the alveoli and the surfactant phospholipid is deposited.

8.1.3 Post administration of allocated intervention

All participants must be given a limited physical examination, with minimal handling, in the interval between one hour and two hours after the administration of each intervention dose (see section 8.5.1).

8.1.4 Weaning and extubation by protocol

The unblinded staff involved in the administration of the BESS intervention (study respiratory physiotherapist or nurse practitioner or other qualified person and one usual care PICU nurse) and parents who may be observing, will not be involved in decisions to wean respiratory support provided by mechanical ventilation and will not be involved in decisions to extubate.

We will wean respiratory support provided by MV and extubate according to a standardised protocol that will form part of the site-specific training packages.

Readiness for a spontaneous breathing test (SBT) will be identified by the patient meeting specified physiological targets of gas exchange at a given level of ventilation and/or the treating clinician believes the child to be ready for SBT.

The time at which a child is deemed ready for SBT **must** be recorded in their notes/electronic health record. The time that a child is extubated **must** be recorded in their notes/electronic health record.

For peer review only - http://bmjopen.bmj.com/site/about/guidelines.xhtml

8.2 Participant follow-up

Clinical follow-up (daily data collection) will take place for the period of time between randomisation and the discharge from hospital of the participant.

All non-serious adverse events will be collected until 24 hours after the participant receives their final trial intervention; please note that this may not be their third dose if less than 3 doses are required before the participant meets the criteria to be ready for weaning (see section 10 for full safety reporting information).

Non-serious adverse events occurring after 24 hours post-final intervention but before 90 days post-randomisation/discharge home/death should be reported if the local Investigator believes that they may be related to the intervention.

Although active monitoring for adverse events and adverse reactions will cease at 24 hours post final intervention and 90 days post randomisation / discharge home / death respectively, any which the site research team become aware of after these time-points which are assessed as serious (i.e. Serious Adverse Events or Serious Adverse Reactions) should be reported to the CTRC.

Routinely collected audit data regarding participant care will be collected from PICANet until 90 days post randomisation.

Parent reported outcomes will be collected via the LRSQ at 6 and 12 months post randomisation for participants recruited in seasons 1 and 2, and at 6 months only for participants recruited in season 3.

The end of follow-up definition for each participant will therefore be 12 months post randomisation for participants in season 1 and 2, or 6 months post randomisation for participants in season 3.

For peer review only

For peer review only - http://bmjopen.bmj.com/site/about/guidelines.xhtml

8.3 Schedule for Intervention & Follow-up

Table 2: Schedule for intervention and follow-up

Procedures	Staff	Consent & randomisation	prior to first intervention	1-2hr,post first intervention	6hr	12hr, prior to second intervention	1-2hr post second intervention	24hr, prior to third intervention	1-2hr post third intervention	36hr	48hr	Daily if MV	90d	6m	12m
Prescribe	PI/Dr	X													
Invitation to participate in WP-B (parental experience sub-study)	PI/Dr/RN	X*													
+Invitation to participate in WP-C (surfactant metabolism sub-study)	PI/Dr/RN	X*													
ˣBlood Gas sample (capillary or arterial)	N/RN		X		X	X		X		X	X	X			
CRF completion (patient data collection)	N/RN		X		X	X		X		X	X	X			
Limited physical examination	N/RN/Dr		X	X		X	X	X	X						
ˣBAL	RP		X			X+		X		X+		X			
Administration of trial intervention	RP		X			X		X							
+IV D-Choline	N/RN		X+												
+Blood Sample (venous or arterial)	N/RN		X+			X+		X+		X+	X+				
Safety monitoring and reporting	RN/RP/PI$		X		X	X		X		X	X	X			
LRSQ	RN													X	X*
Mortality and re-admission	RN												X		

RN = Research Nurse, RP = Study respiratory physiotherapist or nurse practitioner or any other qualified person not involved in decision to wean or extubate, PI = Local Principal Investigator or delegate, N = Nurse (usual care), Dr = licensed medical practitioner.

ˣ Before each trial intervention

+ Only at Liverpool & Southampton and with specific consent (see section 12.2)

***** Seasons 1 & 2 only

$ Although all delegated staff are responsible for identifying and reporting adverse events, decisions regarding causality and seriousness, and sign-off of serious adverse event reports can only be undertaken by a licensed medical practitioner.

For peer review only - http://bmjopen.bmj.com/site/about/guidelines.xhtml

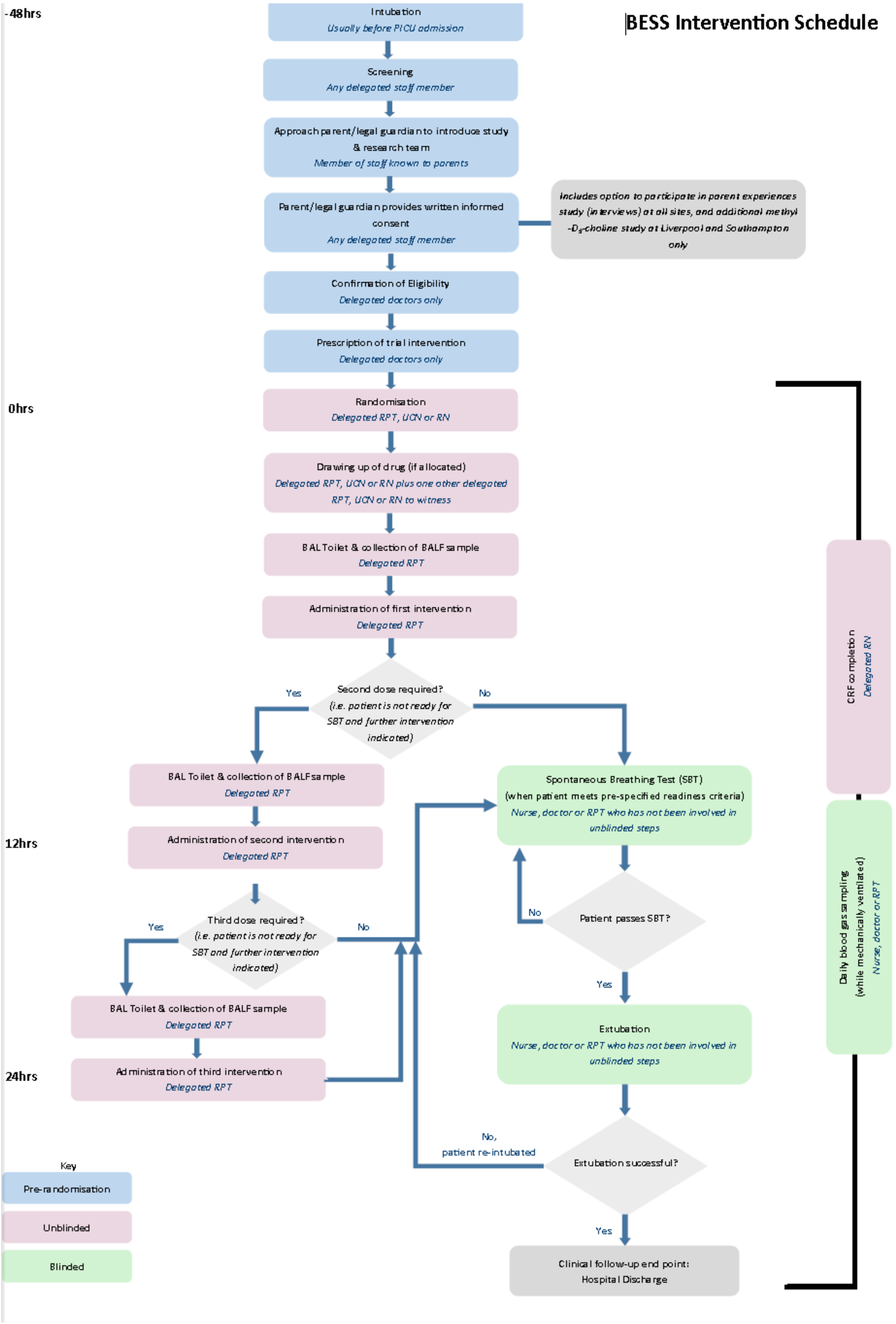

Figure 2: BESS Intervention Schedule

8.4 Procedures for assessing Efficacy

8.4.1 Efficacy Assessment 1

Efficacy will be assessed by group-wise comparison of the primary outcome measure group "Duration of MV after randomisation" by intention to treat. This is defined as time from randomisation to final extubation in hours, including any time off MV where the infant requires reintubation for a further period of MV. Time of randomisation and intervention will be recorded in the patients' notes and CRFs. MV ceases at extubation, an event when the endotracheal tube (breathing tube) is removed and the infant is allowed to breathe without invasive mechanical support. Extubation is an important discrete event in a patient's care pathway, is routinely recorded in the notes and PICANet audit.

8.4.2 Efficacy Assessment 2

The Ventilation Index (VI), Oxygenation Index (OI), Oxygenation Saturation Index (OSI) and SF ratio are secondary outcomes of efficacy derived from blood gas results (capillary blood gas sampling times as per Table 2 Schedule for intervention and follow-up), ventilator settings, and non-invasive monitoring. These data are captured through existing intensive care automated monitoring process or regular manual entry on large format (broad-sheet) bed-charts. This data will be transcribed by the research nurse to the CRF.
Mechanical Ventilation parameters to allow calculation of VI, OI, OSI and SF ratio must be recorded at the corresponding time when Blood Gas samples are scheduled to be taken for research or taken for routine purposes.
Mechanical Ventilation parameters are recorded regularly for ventilated patients as part of standard care. BESS does not require site teams to take any additional readings of these parameters for study purposes, but **all blood gas and ventilator readings** taken as part of standard care must be transcribed into the CRF to allow for calculation of these ratios over time.
After extubation, all inhaled Oxygen and peripheral oxygen saturation parameters recorded as part of standard care must be transcribed into the CRF to allow for calculation of this ratio over time.
Blood gas indices and ratio definitions are as follows:

- VI = ($PaCO_2$ × (PIP-PEEP) × respiratory rate)/1,000
- OI = (MAP × FiO_2 × 100)/PaO_2
- OSI = (MAP × FiO_2 × 100)/SpO_2
- SF = SpO_2/FiO_2

MAP = mean airway pressure
$PaCO_2$ = partial pressure of arterial carbon dioxide
FiO_2 = Fraction of air inspired that is oxygen
PaO_2 = partial pressure of arterial of oxygen
SpO_2= peripheral oxygen saturation

The time that physiological criteria are met that indicate suitability to test readiness for extubation by spontaneous breathing and/or the treating clinician believes the child to be ready for SBT will be recorded in the patient medical records and CRF.
All-cause mortality at 90 days after intervention will be established via a local enquiry against the hospital electronic health record.

For peer review only - http://bmjopen.bmj.com/site/about/guidelines.xhtml

8.5 Procedures for Assessing Safety

Safety will be assessed by:

- The Principal Investigator (PI) or delegated research staff actively monitoring and reporting all adverse events from randomisation until 24 hours after the participant's last administration of poractant alfa (see section 10 for adverse event reporting).
- Limited physical examination that includes the recording of vital signs before and after each study intervention
- Routinely collected patient care data downloaded from the PICANet audit on a regular basis by each site and transferred to the CTRC
- Mortality and readmission data collected by each site at 90 days post randomisation

Participants will be actively monitored and any adverse events identified must be reported to the CTRC, whether identified from trial assessments or standard care (e.g. identified during standard daily examinations). Non-related events will be reported from randomisation to 24 hours after the participant's last administration of poractant alfa or placebo for. Adverse events occurring after this time point until 90 days post randomisation / discharge home / death will be reported if felt to be related to the intervention. Any serious events or reactions (SAEs/SARs) should be reported to CTRC regardless of the timeframe.

8.5.1 Procedure for Limited Physical Examination in BESS

Participants in BESS are unwell and may be haemodynamically unstable. Handling of the participants, including full physical examination, is minimised in standard care, as per current NICE guidance (42).
All participants must have a physical examination before and after each administration of the trial intervention. This is a limited physical examination to minimise any disturbance to the infant participant.
Examinations must be carried out any time in the period 2 hours prior to each administration, and the interval of time between 1 hour and 2 hours post each administration. The post administration examination is deliberately delayed by at least one hour after administration to allow stabilisation of the participants' vital signs and avoid prolonging the handling associated with administration.
The limited examination must include the following:

- Temperature
- Heart rate
- Blood pressure
- Respiratory rate
- Oxygen saturations (peripheral)

To minimise handling, if a participant has had a full physical examination as part of standard care at any time in the period 2 hours prior to the administration and the vital signs data have been collected, then this examination should be counted as their BESS physical examination and the vital sign data used.
The physical examination vital sign data must be recorded in the patient notes and transcribed onto the relevant BESS CRF.

8.6 Other Assessments

8.6.1 Parent Reported Quality of Life questionnaires

The parent reported outcome measure the Liverpool Respiratory Symptom Questionnaire (LRSQ), is a web-accessible questionnaire that has been optimised for many platforms

For peer review only - http://bmjopen.bmj.com/site/about/guidelines.xhtml

including smart-phones. Links to the form will be emailed to parents prior to the intended completion dates (in all three seasons at 6m, and in addition at 12m to parents of infants recruited in seasons 1 and 2). A paper version is available with a “free-post” return envelope.

8.6.2 Special Assays or Procedures

8.6.2.1 Collection & processing of BAL fluid

BAL sampling with 0.9% sodium chloride (2x1mL/kg) will be done for all participants immediately prior to intervention, at 24hrs post intervention and repeated daily while the infant remains on MV (see Table 2).

Both tracheal toilet and BAL sampling is done by catheter access to the trachea via the endotracheal suction port without disconnecting the infant from the ventilator or by disconnecting the infant from the ventilator and performing hand bagging while accessing the trachea via a side-port with one-way valve.

8.6.2.2 Blood gas sampling

Blood Gas sampling is a routine procedure required for the clinical monitoring of all people requiring mechanical ventilation. The typical sample volume of blood drawn from infants and children is 100 microliters equivalent to two drops. Infants and children can have Blood Gas sampling by drawing blood from an existing arterial line or from a heel prick. To minimise disturbance and risks to infants from additional research procedures, the data from routine Blood Gas samples will be used for the trial when collected within a 2-hour window of the scheduled trial Blood Gas sample timepoint up to and including the sample due at 48 hours post first intervention (see Table 2). After 48 hours, i.e. day 3 and onwards, and only if the infant continues to require mechanical ventilation, then data from any one routine blood gas sample collected within a 6-hour window 9:00am to 3:00pm in each subsequent 24-hour interval will be collected (the first such sample if more than one is taken). A research Blood Gas sample will only be done if a routine blood gas sample is not done or planned within each 24-hour period sampling window.

Blood gas machines are usually kept in the PICU rather than the hospital/Trust laboratory. PICUs will not be CPS accredited but each Trust laboratory, and the machinery they are responsible for, will be. The CTRC will acquire a copy of the trust CPS laboratory certificate and calibration log for the blood gas machines as part of the compulsory ‘Green Light Checklist’ procedures before a site can open to recruitment. The Trust laboratory **must** take responsibility for service of the PICU blood gas machines and be able to provide a quality control log for maintenance and calibration of each blood gas machine.

8.6.2.3 Additional samples (WP-C at Liverpool and Southampton only)

Participants at Liverpool and Southampton will be given the option to consent to a single intravenous dose of D_9-labelled choline which will inform the mechanistic sub-study on surfactant synthesis and metabolism (WP-C; see section 12.2 for full details). This work will not inform either the primary or secondary outcomes of the main trial (WP-A). These samples will **only** be taken from participants who have provided specific consent for this optional additional work.

For peer review only - http://bmjopen.bmj.com/site/about/guidelines.xhtml

Small volume lung lavages will be collected immediately prior to intervention and at 24hrs, as per the standard follow-up (see section 8.2), and at additional time points of 12hrs and 36hrs for WP-C participants only. Samples of 1.25mL (range 1.0 to 1.3mL) of blood will be collected at the same time points for WP-C participants at Liverpool and Southampton only.

8.7 Patient Transfer and Withdrawal

8.7.1 Patient Transfers

It is very unlikely that participants taking part in BESS will be transferred during their trial treatment, given the severity of illness of participants and the short time period for administration of the intervention. In the unlikely scenario that a BESS participant is transferred while they remain intubated, their trial treatment will end at transfer unless they are transferred directly to another BESS site. For participants transferred to another participating trial site the receiving trial site must take over responsibility for the participant.

A copy of the participant CRFs should be provided to the new site. The parent will have to sign a new consent form at the new site, and until this occurs, the participant remains the responsibility of the original site.

If a participant is transferred to a site that is not taking part in BESS then they will be withdrawn from the trial and will receive no further interventions. Their care will revert to the standard practice and will not be affected by the withdrawal from the trial. The CTRC should be notified in writing of all participant transfers, whether or not to a participating trial site.

8.7.2 Withdrawal from the Trial

In consenting to the trial, parents are consenting to trial intervention and treatment, follow-up assessments and data collection for their child.

The rights and welfare of the participants will be protected by emphasising to their parents that the quality of medical care will not be adversely affected if they withdraw consent for their child to participate in this trial.

8.7.2.1 Partial Withdrawal from Trial (Intervention and Treatment), allowing Follow-up and Data Collection)

Participants may be withdrawn from trial intervention and treatment for any of the following reasons:

a. Parent withdraws consent.

b. Unacceptable toxicity.

c. Intercurrent illness preventing further treatment.

d. Any change in the participant's condition that justifies the discontinuation of treatment in the clinician's opinion.

If voluntary withdrawal from trial intervention and treatment occurs, the parent should be asked to allow continuation of scheduled evaluations (follow-up and data collection) and be given appropriate

For peer review only - http://bmjopen.bmj.com/site/about/guidelines.xhtml

care under medical supervision until the symptoms of any adverse event resolve or the participant's condition becomes stable.

If a parent wishes to withdraw their infant from trial intervention and treatment, sites should explain the importance of remaining on trial follow-up, or failing this, of allowing routine follow-up data to be used for trial purposes. Follow-up and data collection will continue unless the parent explicitly withdraws consent for follow-up or data collection. Research data obtained up to the point of withdrawal of consent will be retained, and further data will only be collected where required by law for reporting to regulatory agencies (i.e. serious adverse events). Research samples will be destroyed or retained for the purpose of the trial according to the wishes of the parent.

8.7.2.2 Withdrawal from Trial Completely

Parents are free to withdraw consent at any time without providing a reason. Parents who wish to withdraw consent for the trial completely (trial intervention, follow-up and data collection) will be informed that pseudo-anonymised data collected up to the point of that withdrawal of consent will be included in the analyses, in accordance with current data protection legislation. The participant will not contribute further data to the trial (with the exception of serious adverse event data); the CTRC should be informed in writing and a Withdrawal CRF should be completed. Data up to the time of withdrawal will be included in the analyses.

8.8 Loss to Follow-up

Trial follow-up will be in part via routinely collected data downloaded in a bespoke trial download by each recruiting site; in part via paper CRFs completed by the trial RNs and RPTs; and in part via LRSQs collecting Parent Reported Outcome Measures (PROMs) at 6 and 12 months post randomisation. Where a patient is lost to follow-up before the relevant time point (e.g. lost due to transfer to another hospital), contact will initially be attempted by the trial RNs and the PI at each site. Wherever possible, information on the reason for loss to follow-up will be recorded.

8.9 Trial Closure

The end of the trial is defined to be the date on which data for all participants are locked and data entry privileges are withdrawn from the trial database. However, the trial may be closed prematurely by the Trial Steering Committee (TSC), on the recommendation of the Independent Data and Safety Monitoring Committee (IDSMC).
Individual sites may be closed to recruitment prior to their intended recruitment end date if the Trial Management Group (TMG) have concerns about their capacity or capability to deliver the trial, or for operational reasons whereby resources are better used at sites with better capacity to recruit.
At the point of closure to recruitment all sites will be required to undertake closedown activities which include but are not limited to: a review of their Investigator Site File (ISF), a count of all completed CRFs and completed data queries. PIs will also be required to sign-off the CRF for each participant, any changes to the data, a closedown checklist and their site delegation log.

For peer review only - http://bmjopen.bmj.com/site/about/guidelines.xhtml

9 TRIAL TREATMENT/INTERVENTIONS

9.1 Introduction

The intervention being trialled is Endotracheal poractant alfa (Curosurf®, porcine lung phospholipid fraction supplied at 80mg/mL).

Curosurf® is a standard natural surfactant treatment prepared from porcine lungs for intratracheal administration and containing almost exclusively polar lipids, in particular phosphatidylcholine. Endotracheal poractant alfa is licensed for use in the UK for the treatment of Respiratory Distress Syndrome (RDS) or hyaline membrane disease in newborn babies (neonates). It is being used as an investigational medicinal product (IMP) on the BESS trial (UK licence, new indication).

Three small studies of surfactant use in infants with bronchiolitis have raised no safety concerns, however BESS will be the first large, randomised study of any surfactant for the treatment of bronchiolitis of infancy (3). As neonatal RDS is caused by surfactant deficiency, and the viral infections that cause bronchiolitis of infancy also cause surfactant deficiency (which we aim to treat with poractant alfa), we expect that the well tolerated safety profile of use in RDS in neonates will be reflected when using surfactant to treat bronchiolitis in infants.

Participants will be randomised equally to receive either endotracheal poractant alfa or air-placebo in a sham procedure. Participants randomised to receive endotracheal poractant alfa will receive a first dose of 200mg/kg, repeated 12 hourly at 100mg/kg, to a maximum of 3 doses. This is in line with the manufacturer's summary of product characteristics (SPC). Participants randomised to the placebo will receive an identical series of up to three procedures (while intubated) using air as the placebo.

For peer review only

9.2 Arm A – Endotracheal Poractant Alfa at 200mg/kg (first dose), with up to two further doses at 100mg/kg

9.2.1 Formulation, Packaging, Labelling, Storage and Stability

Generic name: Endotracheal poractant alfa

Brand name: Curosurf®

Supply and distribution: Chiesi Farmaceutici S.p.A (Chiesi) will supply Curosurf® via a third-party distributor and will provide appropriate certificates of analytical conformity. Additional shipments during each season will be managed by the distributor contracted by Chiesi, via an automated web-based system.

Packaging: Labelled holder containing one labelled glass vial with a blue topper.

Formulation: Each vial contains 3ml volume of poractant alfa at a concentration of 80mg/ml.

For peer review only - http://bmjopen.bmj.com/site/about/guidelines.xhtml

Labelling: Vials will be labelled by Chiesi in English and in compliance with the detailed guidance provided in annex 13 of the European Union (EU) Good Manufacturing Practice (GMP) guide. Each vial will be labelled and used for BESS trial use only.

Storage: The PI or delegated research team member will be responsible for the safe storage of all vials assigned to the BESS trial, in a safe place with restricted access (usually a designated drug room on the PICU). Poractant alfa will be stored between 2ºC and 8ºC, in a separate drug fridge specifically for use in the trial, protected from light in the original packaging.

Stability: Curosurf® has an unopened shelf life of 18 months. Each vial is for single use only.

9.2.2 Preparation, Dosage and Administration of Study Treatment/s

Dosage: An initial dose of 200mg/kg, with two further doses of 100mg/kg at 12hrs and 24hrs if indicated.

200mg/kg is 200milligrams of poractant alfa per kilogram of body weight. 100mg/kg is 100milligrams of poractant alfa per kilogram of body weight. The participant's weight will be detailed on the prescription in kilograms and the prescribing clinician will calculate the appropriate dose and number of vials needed for that participant.

Preparation: Poractant Alfa suspension is refrigerated, so before use each vial must be warmed up slowly to room temperature e.g. keeping it in hands for 5-10 minutes and gently turned upside down, without shaking, avoiding the formation of foam. Artificial warming methods should not be used.

The entire contents of the vials of poractant alfa suspension should be slowly withdrawn into a plastic syringe through a large-gauge needle and the excess poractant alfa discarded. A second person must be witness to the drawing up of the poractant alfa to assist and to check that the volume drawn up is the amount prescribed. Once the prescribed volume has been drawn into the syringe, an opaque paper shield should be applied to the syringe in order to maintain blinding of staff and/or parents at the bedside.

At this stage the appropriate equipment should also be prepared for conducting tracheal toilet and BAL.

Tracheal toilet (and, where scheduled, BAL) must be conducted prior to administration of the intervention.

The use of muscle relaxant prior to tracheal toilet, BAL and or administration of the intervention is at clinical discretion.

Administration: Either with the infant connected to the ventilator, or momentarily disconnected with hand-bagging, the syringe filled with Poractant Alfa suspension will be connected to a 5-French catheter end-hole inserted into the endotracheal tube (ETT) for ventilation. Passing the catheter through the suction or side port into the ETT and advancing the catheter aiming to have the tip of the catheter placed just beyond the tip of the ETT. The insertion length should be calculated from the known length of the ETT (typically cut to reduce dead space) plus the distance from the end of the cut edge of the ETT to the suction/side port). The intervention will be given slowly as a single bolus directly into the lower trachea (recommended over 0.5 to 3 minutes). From there it disperses through ventilation cycles into the peripheral lung tissue where the excipient (buffered 0.9% sodium chloride) is absorbed in the alveoli and the surfactant phospholipid is deposited.

The infant should be stabilized before surfactant administration and their head placed in the neutral position (head and body in alignment without inclination).

For peer review only - http://bmjopen.bmj.com/site/about/guidelines.xhtml

Before starting the procedure and throughout it, infants must have cardiorespiratory monitoring: oxygen saturation and heart rate continuously through pulse oximetry, blood pressure measurement either continuously in presence of an arterial catheter or intermittently if no arterial line is in place.
The first dose at 200 mg/kg will be followed up by two further doses at 100 mg/kg at approximately 12-hour intervals and the exact time and actual volume of each administration will be noted in the medical notes and appropriate section of the CRF.
No suctioning of the airways should be performed for two hours after each surfactant instillation unless the treating physician deems this appropriate, in which case this will be noted in the medical notes and appropriate section of the CRF.

9.2.3 Specific Restrictions

The effect of co-administration of poractant alfa with other agents is not known. Do not administer poractant alfa concomitantly in the same intratracheal bolus with other agents.

9.2.4 Overdose

Overdose may occur if the wrong patient weight has been supplied to the prescribing clinician, an incorrect weight is used in the calculation or the calculation has been done incorrectly. There have been no reports of overdose following the administration of Curosurf® however, in the unlikely event of an accidental overdose that causes clear clinical effects on the infant's respiration, ventilation or oxygenation, as much of the suspension should be aspirated as possible, as per the SPC.
Special care must be taken by all site team members to avoid overdosing, noticeably the prescribing clinician and the staff members drawing up the poractant alfa for administration. In the event of identification of an overdose, unblinding should occur and the participant will be closely observed for any adverse events (AE). The site team should notify the CTRC of the event and examine the reasons for the event occurring to ensure that it does not happen again. The CTRC will log any overdosing events as protocol deviations.

9.2.5 Temperature Compliance

Temperature deviations at site will be identified by the regular completion of a trial-specific temperature monitoring log. The PI or delegated member of staff will check the fridge used to store stocks of poractant alfa for use in BESS **daily** and will alert the CTRC if a temperature deviation has occurred. Temperature checks will be recorded on a Temperature Monitoring Log.

Poractant Alfa shipment and storage conditions:

**Poractant Alfa (Curosurf®)** Suspension 80 mg/ml to 3ml vial	+2°C to +8°C	Up to +25°C for maximum cumulative 96hrs

Unopened vials of Curosurf® that have warmed to room temperature can be returned to refrigerated storage **within 24hours** for future use, **if** this is the first time that they have warmed. Vials that have been removed from the fridge and been allowed to warm to room temperature for a second time **must not be used**, but should be retained for accountability checks at the end of each season.

Temperature compliance during shipment will be checked by the local site Pharmacist on receipt of the IMP. The local Pharmacist will review the temperature curves/logs from the calibrated

For peer review only - http://bmjopen.bmj.com/site/about/guidelines.xhtml

temperature logger used during IMP shipment and will check for any out of range values. If an out of range temperature is identified, the local Pharmacist will inform the CTRC within 48 hours. It is the responsibility of the CTRC to inform Chiesi, who will then notify the Chiesi R&D Quality Assurance GMP Unit & Project Partners (R&D QA-GMP).

If the out-of-range temperature deviation does not affect the quality and safety of the products, a declaration of usability will be issued and signed by R&D QA-GMP and sent back to the CTRC. CTRC will then authorise the trial site to use the vials in question.

If the products are judged not suitable for use, Chiesi R&D QA-GMP will inform the CTRC to permanently suspend the use of the affected vials. Any vials not used due to temperature deviations will be retained for accountability checks at the end of each season before being sent for destruction.

9.2.6 Accountability Procedures for Trial Treatments

The PI is responsible for the management of poractant alfa to be used for the trial. Trial IMP should be stored in the original packaging to protect from light, in a locked, secure, refrigerated storage facility with access limited to those individuals authorized to dispense or administer the trial IMP. Initial supply of the trial IMP will be to the site Pharmacy. The site Pharmacists are responsible for dispensing stock to a secure drug room on the PICU for more immediate access by the research team. Pharmacists must release the trial IMP to PICU in sequential order by the medication numbers on the labels to ensure the Randomisation System can allocate trial IMP with the shortest shelf life first. No surfactant material supplied for this trial is to be used for any other purpose.

Accountability logs will be maintained by the PI, pharmacist or delegated research team member, which will include a signed account of all the vials of Curosurf® received from the distributor, the vials dispensed to and returned from PICU, with the vials and volume used for each randomised patient.

All completely used vials will be retained at the site until the conclusion of the recruitment season. Each vial is for single use only. Any leftover unused medication is to be retained for drug accountability. Partially used vials present a risk of confusion and sharps injury, so all partially used vials will be disposed of within the PICU as per local Trust standard procedures. Disposal of partially used vials will be logged for accountability checks.
At the conclusion of each recruitment season, the PI, pharmacist or delegated individual shall conduct and document a drug supply (used and unused) inventory. An explanation will be given for any discrepancies.

After the inventory is reconciled at the end of each season and only once the CTRC have given the site authorisation on behalf of the sponsor, remaining unused vials of Curosurf® maybe disposed of directly by the Pharmacy of the involved trial site. Unused vials with an expiry date during or after the next season will be retained. Documentation assuring the unused vials were sent off-site for destruction, as per standard Trust procedures will be provided. This must be signed by two delegated members of staff filed in the Pharmacy site file, with a copy returned to the CTRC for storage in the Trial Master File.

For peer review only - http://bmjopen.bmj.com/site/about/guidelines.xhtml

9.2.7 Assessment of Compliance with Study Treatment/s

Each vial of Curosurf® will be designated by code number and the actual volume of instilled test treatment will be noted in the hospital chart and on the CRF of the study.

The completely used vials will be kept in a sealed labelled plastic box/envelope with patient's randomisation number/initials until the end of the season for accountability checks. There will therefore be three "final" accountability checks at each site, one at the end of each recruitment season.

9.3 Arm B – Placebo

Placebo: Air via an identical series of 3 doses in a sham procedure.

Dosage and preparation: Air should be drawn into a syringe at the same volume of 200mg/kg for the first dose and 100mg/kg for the second and third doses (if required), as per the instructions for poractant alfa dose calculations assuming the intervention is provided at 80mg/ml (section 9.2.2). Once the prescribed volume has been drawn into the syringe, an opaque paper shield should be applied to the syringe in order to maintain blinding of staff and/or parents at the bedside.

The use of muscle relaxant prior to tracheal toilet, BAL and or administration of the intervention is at clinical discretion.

Administration: Either with the infant connected to the ventilator, or momentarily disconnected with hand-bagging, the syringe filled with air will be connected to a 5-French catheter end-hole inserted into the endotracheal tube (ETT) for ventilation. Passing the catheter through the suction or side-port into the ETT and advancing the catheter aiming to have the tip of the catheter placed just beyond the tip of the ETT. The insertion length should be calculated from the known length of the ETT (typically cut to reduce dead space) plus the distance from the end of the cut edge of the ETT to the suction/side port). The air will be given slowly as a single bolus directly into the lower trachea (recommended over 0.5 to 3 minutes).

9.4 Unblinding

N.B. Allocation must not be routinely revealed to CTRC personnel. It is to be expected that some site staff may be accidentally unblinded due to unavoidable back-tracking of surfactant up the participant's ETT (see section 7.4).

To maintain the overall quality and legitimacy of the trial, unblinding should occur only in exceptional circumstances when knowledge of the actual treatment is **absolutely essential** for further management of the patient (SPIRIT 2013).

After unblinding the Local Investigator is encouraged to maintain the blind as far as possible. The actual allocation must NOT be disclosed to the participant's family or other study personnel including

For peer review only - http://bmjopen.bmj.com/site/about/guidelines.xhtml

other site personnel, monitors, sponsors or project office staff; nor should there be any written or verbal disclosure of the code in any of the corresponding patient documents.

The Investigator may unblind at their discretion without need to contact the CTRC first, but must report all unblinding (with reason) as they occur on the corresponding Unblinding CRF.

Unblinding should not necessarily be a reason for study drug discontinuation.

9.4.1 Emergency Unblinding

If a Local Investigator urgently needs to unblind a participant, they are able to do so by opening the unblinding envelope corresponding to the participant's randomisation number.

Partially completed unblinded envelopes will be provided to all sites. The allocation (poractant alfa or air as placebo), as generated by the WebEZ randomisation system, will be marked on a pre-printed slip inside the envelope by the randomising team member, and the envelope sealed. There will be one partially completed envelope for each patient, with the randomisation number clearly visible on the front of the envelope.

Sealed unblinding envelopes should be stored in a secure location that is readily accessible to PICU staff if the need for emergency unblinding arises.

In the case of any adverse event that requires emergency treatment and there is not sufficient time to unblind using the unblinding envelopes, Local Investigators should assume that the participant has been given poractant alfa (Arm A) and administer treatment accordingly. The treatment for any acute adverse event potentially associated with the study intervention would be the same whether the patient had been given surfactant or air. All adverse events should be reported as per the procedures described in section 10.

9.4.2 Accidental unblinding

If accidental unblinding occurs, this must be reported to the CTRC by use of the Unblinding CRF. When reporting include details about:

1. Date of unblinding;
2. Detailed explanation of circumstances;
3. Recipients of the unblinding information;
4. Action to prevent further occurrence, if possible.

9.4.3 Unblinding at Trial Closure

Upon trial closure the blinding of parents and blinded staff remains in effect. Site teams and/or pharmacy departments **should not** disclose treatment allocations on an individual basis.

For peer review only - http://bmjopen.bmj.com/site/about/guidelines.xhtml

9.5 Concomitant Medications

No incompatibilities or interactions of Curosurf® with other medications are known. There are therefore no restrictions placed by the trial on the use of concomitant medications in BESS participants.

For peer review only

For peer review only - http://bmjopen.bmj.com/site/about/guidelines.xhtml

10 SAFETY REPORTING

10.1 Terms and Definitions

"Adverse Event (AE)"

An adverse event (AE) is any untoward medical occurrence in a subject to whom a medicinal product has been administered, including occurrences which are not necessarily caused by or related to that product.

Therefore an AE is any unfavourable or unintended change in the function (symptoms), structure (signs) or chemistry (laboratory data) in a subject to whom an IMP has been administered, including occurrences which are not necessarily caused by or related to that product.

"Adverse Reaction (AR)"

An adverse reaction (AR) is any untoward and unintended response in a subject to an IMP that is related to any dose administered to that subject.

Therefore an AR is any unfavourable or unintended change in the function (symptoms), structure (signs) or chemistry (laboratory data) in a subject that is related to any dose of an IMP administered to that subject.

"Unexpected Adverse Reaction (UAR)"

An unexpected adverse reaction (UAR) is an adverse reaction the nature and severity of which is not consistent with the information about the medicinal product in question set out in the Investigator's Brochure (IB) or the SPC, which may be referenced where the IMP in question is a product with a marketing authorisation.

"Serious Adverse Event (SAE), "Serious Adverse Reaction, or Unexpected Serious Adverse Reaction"

A serious adverse event (SAE) is an AE, AR or UAR respectively that:

- results in death;
- is life threatening (places the subject, in the view of the Investigator, at immediate risk of death from the experience as it occurred – this does not include an adverse experience that, had it occurred in a more severe form, might have caused death);
- requires hospitalisation or prolongation of existing hospitalisation (hospitalisation is defined as an in-patient admission, regardless of length of stay, even if the hospitalisation is a precautionary measure for continued observation – hospitalisations for a pre-existing condition, including elective procedures that have not worsened, do not constitute an SAE);
- results in persistent or significant disability or incapacity (substantial disruption of one's ability to conduct normal life functions);
- consists of a congenital anomaly or birth defect (in offspring of subjects, or their partners, taking the IMP regardless of time of diagnosis);
- other important medical events (these may not result in death, be life-threatening, or require hospitalisation, but may be considered a serious adverse event or experience when, based upon appropriate medical judgment, they are considered to jeopardise the subject and may require medical or surgical intervention to prevent one of the outcomes listed in this definition).

For peer review only - http://bmjopen.bmj.com/site/about/guidelines.xhtml

"Suspected Serious Adverse Reaction (SSAR)"

A suspected serious adverse reaction (SSAR) is an adverse reaction that is classed in nature as serious and which is consistent with the information about the IMP in question, which in the case of a licensed product is set out in the SPC for that product, and in the case of any other IMP is set out in the IB relating to the trial in question.

"Suspected Unexpected Serious Adverse Reaction (SUSAR)"

A suspected unexpected serious adverse reaction (SUSAR) is an adverse reaction that is classed in nature as serious and which is not consistent with the information about the IMP in question, which in the case of a licensed product is set out in the SPC for that product, and in the case of any other IMP is set out in the IB relating to the trial in question.

"Reference Safety Information (RSI)"

The reference safety information (RSI) for a trial is the information used for assessing whether an adverse reaction is expected. This is contained in either the IB or the SPC.

10.2 Notes on Adverse Event Inclusions and Exclusions

The below sections provide guidance on what should, and should not be considered as AEs for the purposes of the BESS study:

10.2.1 Include:

- An exacerbation of a pre-existing illness, **excluding** the current case of bronchiolitis.
- An increase in frequency or intensity of a pre-existing episodic event/condition.
- A condition (even though it may have been present prior to the start of the trial) detected after trial drug administration.
- Continuous persistent disease or symptoms present at baseline that worsen following the administration of the trial treatment, **excluding** the current case of Bronchiolitis.
- Laboratory abnormalities that require clinical intervention or further investigation (unless they are associated with an already reported clinical event).
- Abnormalities in physiological testing or physical examination that require further investigation or clinical intervention.
- Injury or accidents.

10.2.2 Do Not Include:

- Widely recognised and documented clinical features of bronchiolitis or outcomes associated with MV that are defined outcome measures in this trial (see section 3.5).
- Medical or surgical procedures* – the condition which leads to the procedure is the adverse event; e.g. pneumothorax leading to chest drain placement, here pneumothorax is the event.
- Pre-existing disease or conditions present before treatment that do not worsen.
- Situations where an untoward medical occurrence has occurred, e.g. cosmetic elective surgery.
- Overdose of medication without symptoms or signs**.

For peer review only - http://bmjopen.bmj.com/site/about/guidelines.xhtml

* *Cosmetic elective surgery is cited here as example of an event that is not reportable as an AE.*
** See also section 9.2.4. *If overdose occurred with resulting symptoms and signs that met the protocol criteria for AE/AR/SAE/SAR then they should be reported accordingly. Please note that though overdose of medication without symptoms or signs may be excluded from AE reporting this may still require investigation to ensure the protocol and regulatory requirements are met, e.g. for IMP management and administration to ensure participant safety.*

10.2.3 Notification of deaths

There is an overall likelihood of death in all cases of severe bronchiolitis of 1-2%. All deaths that occur during the protocol-specified AE reporting period, regardless of relationship to study drug, must be recorded. All deaths should be reported on a "Serious Adverse Event CRF" and returned to the CTRC within 24 hours.

Death should be considered an outcome and not a distinct event. The event or condition that caused or contributed to the fatal outcome should be recorded as the single medical concept on the appropriate CRF; e.g. death following pneumothorax, here pneumothorax is the event. Generally, only one such event should be reported. The term "sudden death" should only be used for the occurrence of an abrupt and unexpected death due to presumed cardiac causes in a participant with or without pre-existing heart disease, within 1 hour of the onset of acute symptoms or, in the case of an unwitnessed death, within 24 hours after the participant was last seen alive and stable. If the cause of death is unknown and cannot be ascertained at the time of reporting, "unexplained death" should be recorded on the appropriate CRF. If the cause of death subsequently becomes available (e.g. after post-mortem examination), "unexplained death" should be replaced by the established cause of death.

10.3 Notes on Severity / Grading of Adverse Events

The assignment of the severity/grading should be made by the investigator responsible for the care of the participant using the definitions below.

Regardless of the classification of an AE as serious or not, its severity must be assessed according to medical criteria alone using the following categories:

Mild: does not interfere with routine activities
Moderate: interferes with routine activities

Severe: impossible to perform routine activities

A distinction is drawn between serious and severe AEs. Severity is a measure of intensity (see above) whereas seriousness is defined using the criteria in section 10.1, hence, a severe AE need not necessarily be a SAE.

For peer review only - http://bmjopen.bmj.com/site/about/guidelines.xhtml

10.4 Relationship to Trial Treatment

The assignment of the causality should be made by the investigator responsible for the care of the participant using the definitions in Table 3: Definitions of Causality. An AE for which the causal relationship to the study drug is assessed by the investigator as "possible", "probable" or "almost certain" is an AR.

If any doubt about the causality exists the local investigator should inform the CTRC who will notify the Chief Investigator (CI). In the case of discrepant views on causality between the investigator and others, the MHRA will be informed of both points of view.

Table 3: Definitions of Causality

**Relationship**	**Description**
**Unrelated**	There is no evidence of any causal relationship. N.B. An alternative cause for the AE should be given.
**Unlikely**	There is little evidence to suggest there is a causal relationship (e.g. the event did not occur within a reasonable time after administration of the trial medication). There is another reasonable explanation for the event (e.g. the participant's clinical condition, other concomitant treatment).
**Possibly**	There is some evidence to suggest a causal relationship (e.g. because the event occurs within a reasonable time after administration of the trial medication). However, the influence of other factors may have contributed to the event (e.g. the participant's clinical condition, other concomitant treatments).
**Probably**	There is evidence to suggest a causal relationship and the influence of other factors is unlikely.
**Almost certainly**	There is clear evidence to suggest a causal relationship and other possible contributing factors can be ruled out.

10.5 Expectedness

The Reference Safety Information (RSI) in BESS is section 4.8 (Undesirable effects) of the Curosurf® Endotracheopulmonary Instillation Suspension SPC.

It is not a regulatory requirement for a reporting physician to provide their opinion of expectedness. Therefore, the reporting physician at the local research site will not be asked to make an assessment of expectedness. The assessment of expectedness will be made by the CI (or their medically qualified delegate) using BESS's current MHRA-approved RSI following receipt of the "Serious Adverse Event CRF" at CTRC.

All events judged by the investigator to be possibly, probably, or almost certainly related to the IMP, graded as serious and unexpected (see current MHRA-approved trial RSI (Curosurf® Endotracheopulmonary Instillation Suspension SPC) for list of Expected Adverse Events) should be reported as a SUSAR.

For peer review only - http://bmjopen.bmj.com/site/about/guidelines.xhtml

10.6 Follow-up After Adverse Events

All adverse events should be followed until satisfactory resolution or until the investigator responsible for the care of the participant deems the event to be chronic or the participant to be stable.

When reporting SAEs and SUSARs the investigator responsible for the care of the participant should apply the following criteria to provide information relating to event outcomes:

- resolved;
- resolved with sequelae (specifying with additional narrative);
- not resolved/ongoing;
- on-going at final follow-up;
- fatal or unknown.

10.7 Time Period for Safety Reporting

Investigators and delegated members of the local research teams will report to CTRC on the appropriate CRFs (see section 10.8):

- all adverse events from randomisation until 24 hours after their final intervention (whether poractant alfa or placebo);
- all adverse reactions from randomisation until 90 days post-randomisation or hospital discharge (whichever is sooner).

Following these periods, site staff are not required to report any non-serious events, however any serious events (SAEs/SARs/SUSARs) which they become aware of must be reported to CTRC.

Upon becoming aware of a serious adverse event (SAE/SAR/SUSAR), the Investigator or other delegated member of the team **must** report this to the CTRC **within 24 hours**.

10.8 Reporting Procedures

All serious events (SAEs/SARs/SUSARs) must be reported in an expedited manner. Depending on the nature of the event the reporting procedures below should be followed. Any questions concerning adverse event reporting should be directed to the CTRC in the first instance. If a site has a serious or urgent query regarding an adverse event outside of CTRC office hours they should contact the Chief Investigator, Professor Calum Semple, on 07506 653 560.

A flowchart is given below to aid in determining reporting requirements.

For peer review only - http://bmjopen.bmj.com/site/about/guidelines.xhtml

10.8.1 Flowchart for Reporting Requirements of Adverse Events

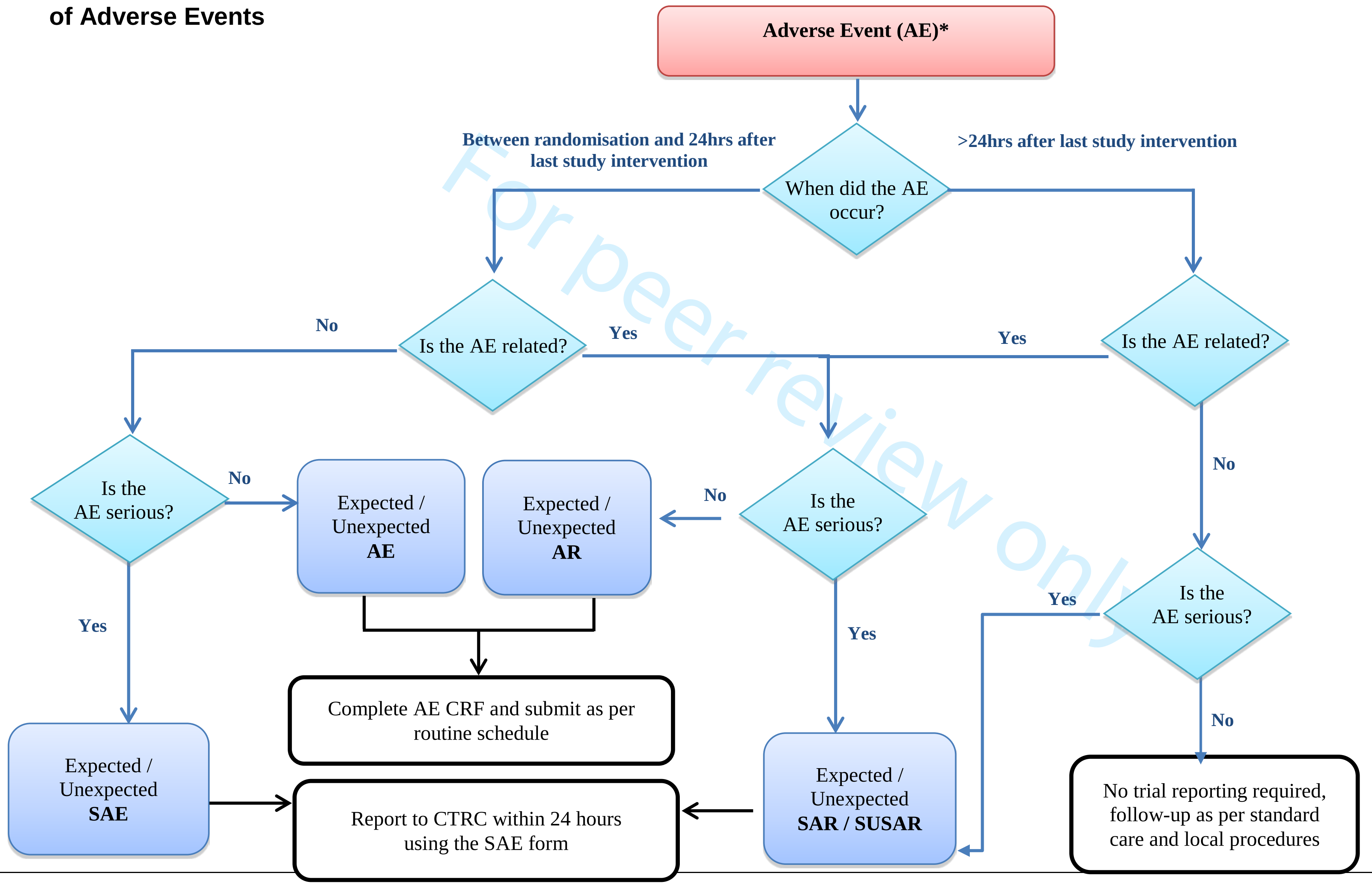

For peer review only - http://bmjopen.bmj.com/site/about/guidelines.xhtml

For peer review only

For peer review only - http://bmjopen.bmj.com/site/about/guidelines.xhtml

10.8.2 Non serious events (ARs/AEs)

All non-serious events (i.e. AEs and ARs), whether expected or not, should be recorded on an “Adverse Event CRF”, which should be transmitted to the CTRC within seven days of the local research team becoming aware of the event.

10.8.3 Serious events (AEs/ARs/SUSARs)

All serious events (i.e. SAEs, SARs and SUSARs) must be reported to CTRC within 24 hours of the local site becoming aware of the event. The “Serious Adverse Event CRF” asks for the nature of the event, date of onset, severity, corrective therapies given, outcome and causality. The reporting local Investigator should assign the causality of the event. Additional information should be sent within 5 days if the event has not resolved at the time of initial reporting.

All adverse events assessed as serious, related and unexpected (i.e. SUSARs) will be reported on to the MHRA and BESS’s Research Ethics Committee (REC) by the CTRC according to the following timelines; fatal and life-threatening SUSARs within 7 days of notification to CTRC and non-life threatening SUSARs within 15 days. BESS PIs will be notified of all SUSARs occurring throughout the trial by the CTRC. Local Investigators should report any SUSARs and/or SAEs/SARs as required locally.

NOTE: *please ensure that multiple Serious Adverse Events are reported separately to the CTRC. Each individual SAE report should relate to one overall diagnosis only.*

10.9 Safety Reporting Responsibilities – Investigator

The local site Investigator (site Principal Investigator (PI)) is responsible for reporting all events that are observed or reported during the study, regardless of their relationship to study product (i.e. AEs/ARs/SAEs/SARs/SUSARs)..

All serious adverse events (SAEs/SARs/SUSARs) must be reported immediately by the investigator to the CTRC on an SAE. All non-serious adverse events should be reported to CTRC within seven days of the local research team becoming aware on an AE CRF.

Minimum information required for reporting serious events

- Valid European Clinical Trials Database (EudraCT) number (if applicable)
- Sponsor study number
- One identifiable coded subject (randomisation number)
- One identifiable reporter (name of PI or appropriate delegated individual)
- One SAE
- One suspect IMP (poractant alfa)
- A seriousness assessment
- A causality assessment

For peer review only - http://bmjopen.bmj.com/site/about/guidelines.xhtml

Process for reporting serious events

i. The SAE CRF should be completed by a local Investigator, named on the 'signature list and delegation of responsibilities log' as responsible for reporting SAEs and making trial related medical decisions. The Investigator should assess the SAE for the likelihood that it is a response to the trial intervention. In the absence of the designated Investigator the SAE CRF should be completed and signed by an alternative member of the research site trial team and submitted to the CTRC. As soon as possible thereafter the responsible Investigator should check the SAE CRF, make amendments as appropriate, sign and re-send to the CTRC. The initial report shall be followed by detailed follow-up CRF reports as appropriate (see number vii).

ii. The "Serious Adverse Event CRF" **must** be submitted to the CTRC **within 24 hours** of the local research team becoming aware of the event; preferably this will be via fax.

Email address: bess@liverpool.ac.uk

Fax Number: 0151 795 8770

iii. When submitting a SAE CRF to the CTRC research sites must also telephone the appropriate Trial Co-ordinator or Data Manager on telephone number **0151 795 8757** to advise that an SAE CRF has been submitted.

iv. The participant **must** be identified by trial randomisation number, date of birth and initials **only**. The participant's name **must not** be used on any correspondence.

v. The local reporting Investigator must **notify** their R&D department of the event (as per standard local governance procedures).

vi. The participant must be followed-up until clinical recovery is complete and laboratory results have returned to normal, or until the event has stabilised. Follow-up should continue after completion of protocol treatment as necessary.

vii. Follow-up information should be noted on a separate CRF by ticking the box marked 'follow-up' and submitting to the CTRC as information becomes available. Extra, annotated information and/or anonymised copies of test results may be provided separately.

10.9.1 Maintenance of Blinding

Systems for SUSAR and SAR reporting should, as far as possible, maintain blinding of individual clinicians and of trials staff involved in the day-to-day running of the trial. The medical management of participants for adverse events should be undertaken under the assumption that the patient has been given surfactant (Arm A). The safety of participants in the trial always takes priority. In each report, seriousness, causality and expectedness should be evaluated for poractant alfa unless unblinding has taken place. All instances of unblinding should be reported to the CTRC.

For peer review only - http://bmjopen.bmj.com/site/about/guidelines.xhtml

Cases that are considered serious, unexpected and possibly, probably or almost certainly related to one of the trial therapies (i.e. SUSARs) would have to be unblinded at the CTRC prior to reporting to the MHRA and REC.

10.10 Safety Reporting Responsibilities – CTRC

The CTRC is undertaking duties delegated by the trial Sponsor and is responsible for the notification of serious events (SAEs/SARs/SUSARs) to the CI (or appropriate delegate) for review and expectedness assessment, and onward expedited reporting of SUSARs and other SARs to the MHRA and REC. The CTRC will also notify PIs at participating sites of all trial SUSARs.

Upon receipt of a SAE CRF, CTRC will liaise with the trial CI (or appropriate delegate) who will review the events reported for seriousness and causality as assessed by the local site research team, and provide and assessment of expectedness. These will be reviewed within 24 hours of receipt at CTRC and those that are identified as SUSARs will be reported to MHRA, REC and Chiesi Farmaceutici S.p.A. by the CTRC. The causality assessment given by the local Investigator at the hospital cannot be overruled and in the case of disagreement, both opinions will be provided with the report.

Timelines of onward reporting to MHRA and REC are as follows:

- SUSARs that are fatal or life-threatening must be reported not later than 7 days after the CTRC is first aware of the reaction. Any additional relevant information must be reported within a further 8 days.
- SUSARs that are not fatal or life-threatening must be reported within 15 days of the CTRC first becoming aware of the reaction.
- A list of all SARs (expected and unexpected) must be reported annually.

The CTRC will also inform Chiesi Global Pharmacovigilance (Parma, Italy) of any adverse event identified as a SUSAR, within 7 days of the CTRC first becoming aware of the reaction.

It is recommended that the following safety issues should also be reported in an expedited fashion:

- an increase in the rate of occurrence or a qualitative change of an expected SAR, which is judged to be clinically important;
- new events related to the conduct of the trial or the development of the IMP and likely to affect the safety of the participants, such as:
    a. a SAE that could be associated with the trial procedures and which could modify the conduct of the trial;
    b. a significant hazard to the participant population, such as lack of efficacy of an IMP used for the treatment of a life-threatening disease;
    c. a major safety finding from a newly completed animal study (such as carcinogenicity);
    d. any anticipated end or temporary halt of a trial for safety reasons and conducted with the same IMP in another country by the same sponsor;
- recommendations of the IDSMC, if any, where relevant for the safety of the participants.

For peer review only - http://bmjopen.bmj.com/site/about/guidelines.xhtml

Participant safety incidents that take place in the course of research should be reported to the National Reporting and Learning System (NRLS) by each participating NHS Trust in accordance with local reporting procedures.

10.11 Safety reports

Safety reports will be generated during the course of the trial which allows for monitoring of serious events (SAEs/SARs/SUSARs) reporting rates across sites. The CTRC will send developmental safety update reports (DSUR) containing a list of all SARs to MHRA and the trial's main REC. Any concerns raised by the IDSMC or inconsistencies noted at a given site may prompt additional training at sites, with the potential for the CTRC to carry out site visits if there is suspicion of unreported AEs in patient case notes. Additional training will also be provided if unacceptable delay in safety reporting timelines. If any safety reports identify issues that have implications for the safety of trial participants, the PIs at all institutions participating in the trial will be notified.

10.12 Urgent Safety Measures

An urgent safety measure is a procedure not defined by the protocol, which is put in place prior to authorisation by the MHRA and REC in order to protect clinical trial participants from any immediate hazard to their health and safety.

The CTRC will notify the MHRA and REC immediately and, in any event, within 3 days that such a measure has been taken and the reasons why it has been taken. The initial notification to the MHRA will be by telephone (ideally within 24 hours) and a notice in writing will be sent within 3 days, setting out the reasons for the urgent safety measure and the plan for further action. After discussion with the MHRA and REC, further action will be agreed, which may include submission of a substantial amendment, a temporary halt, or permanent termination of the trial.

If the study is temporarily halted it may not recommence until authorised to do so by the MHRA and REC. If the study is permanently terminated before the date specified for its conclusion (in the original applications to MHRA and REC), the CTRC should notify the MHRA and REC within 15 days of the date of termination by submitting the formal End of Trial Notification.

10.13 Contact Details and Out-of-hours Medical Cover

Participants will be instructed to contact the local Investigator (their office number) during working hours. All local PIs should ensure to delegate at least one other appropriately trained (in the study and in GCP) medically qualified doctor for assessment of adverse events, safety reporting and making medical decisions in their absence.

Outside working hours the normal local procedure should be followed to contact the local site PI or their agreed delegate. PICU, ward and local switchboard staff should be aware where they should direct calls in- and out-of-hours.

The Chief Investigator has delegated two medically qualified and appropriately experienced doctors to assess safety reports in his absence (Prof Paul McNamara & Dr Kent Thorburn).

The CTRC should be the main point of contact for trial questions during opening hours – 0151 795 8757. Out-of-hours cover for trial related clinical issues that require urgent attention will be provided by the Chief Investigator or his delegates, on 07506 653 560.

For peer review only - http://bmjopen.bmj.com/site/about/guidelines.xhtml

11 STATISTICAL CONSIDERATIONS

11.1 Introduction

Separate Statistical Analysis Plans (SAPs) will be developed prior to the interim and final analyses of the trial.

11.2 Method of Randomisation

Participants will be randomised to receive either poractant alfa or air placebo in a 1:1 ratio. The randomisation list will be generated by an independent statistician at the CTRC who is not otherwise involved in the BESS trial. Randomisation lists will be computer-generated using block randomisation with random variable block length, stratified by site and duration of ventilation (<24hrs and >=24hrs) prior to randomisation.

For peer review only

11.3 Sample Size calculation

The study will be powered to detect a minimally clinically important difference of 18 hours (0.75 days) in the primary outcome variable of time of mechanical ventilation. Data are expected to be positively skewed, and will therefore be log-transformed, and resulting data back-transformed. The null hypothesis is that of no difference in time of MV between the two groups.

Data from Alder Hey Children's Hospital from an audit of 60 eligible infants admitted to PICU in the seasons 2012/3 and 2013/4 showed a mean (SD) duration of ventilation of 4.83 (2.5) days (PICANet data, 2011-14). Parameters for sample size estimation are therefore as follows:

$E(Y_1) = 4.83$ and $SD(Y_1) = 2.5$
$E(Y_2) = 4.08$ and $SD(Y_2) = 2.5$
$\delta = E(Y_2)/E(Y_1) = 0.845$

giving coefficients of variation:

$\kappa_1 = SD(Y_1)/E(Y_1) = 2.5/4.83 = 0.518$
$\kappa_2 = SD(Y_2)/E(Y_2) = 2.5/4.08 = 0.613$

Using the formula of Wolfe and Carlin (1999) with $r = 1$ for equal allocation and a two-sided alpha-level of 0.05, sample size estimations are 100 per group for 80% power and 134 per group for 90% power.

Taking the sample size of 134 and allowing for 5% dropout requires 142 patients will be recruited to each group, giving a total of 284 participants overall.

11.4 Interim Monitoring and Analyses

Analyses of the accumulating data will be performed at regular intervals (at least annually) for review by an IDSMC. These analyses will be performed at the CTRC. The IDSMC will be asked to give advice on whether the accumulated data from the trial, together with results from other relevant

For peer review only - http://bmjopen.bmj.com/site/about/guidelines.xhtml

trials, justifies continuing recruitment of further patients or further follow-up. The IDSMC will make recommendations to the Trial Steering Committee (TSC). A decision to discontinue recruitment, in all participants or in selected subgroups will be made only if the result is likely to convince a broad range of clinicians including participants in the trial and the general clinical community.

11.5 Analysis Plan

A full SAP will be written prior to the conduct of any comparative analysis of the treatment arms. The main features of the SAP are included here.

The primary analysis will be a test of the difference in geometric mean durations of MV between groups (placebo vs. poractant alfa), reported with the corresponding 95% confidence interval. The analysis will be conducted according to the intent-to-treat principle, and will incorporate the stratification factors of duration of ventilation (<24hrs and >=24hrs) prior to randomisation and site.

For the secondary outcomes, longitudinal data for VI, OI, OSI and SF will be analysed with joint modelling. LRSQ data will be modelled longitudinally and comparisons made between groups. Binary data will be reported in terms of relative risk with 95% confidence intervals.

Within BESS, data will be collected as to ascertain readiness for spontaneous breathing test and time of extubation.
As much information as possible will be collected about the reasons for missing outcome data; this will be used to inform any imputation approaches employed in the analysis. Such methods will be fully described in the SAP.

For peer review only - http://bmjopen.bmj.com/site/about/guidelines.xhtml

12 EXPLORATORY & TRANSLATIONAL WORK; WORK PACKAGES B AND C

12.1 Work Package B: Study of Parent & Staff Experiences

BESS will involve an embedded mixed method sub-study involving parents and BESS recruiters involved in recruitment and consent processes. WP-B will explore: acceptability of the trial; approach to recruitment and consent; decision making in the emergency setting; barriers to participation; and the content and format of trial information materials. This will involve: questionnaires and interviews with parents approached for informed consent in BESS, including those who do not go on to be randomised into the trial, and a survey of BESS recruiters.

Parent recruitment in season 1

In season 1, all parents approached for informed consent in BESS will be asked to complete a brief questionnaire and/or take part in a telephone interview. Recruitment to the sub study will occur shortly after the main trial recruitment discussion. This will include parents who decline to provide informed consent for the trial. If more than one parent is involved in the consent discussion, then both are able to complete a questionnaire. By completing the questionnaire, this confirms that the parent has consented to this providing information.

Parents will be asked to place completed questionnaires in a sealed stamped addressed envelope and return it to the BESS recruiter who will post to the research team. Recruitment for questionnaires will take place throughout season 1. In the rare instance that consent is not sought prior to discharge/transferred to another hospital, BESS recruiters should send the questionnaire to parents along with the Parent/Person with PISC to complete.

BESS recruiters will ask the parent to provide contact details on the BESS consent form if they wish to take part in a telephone interview. A researcher will contact families to arrange telephone interviews within one month of consent. At this point parents who live in (or close to) the Merseyside area will be provided with the option of a face to face interview at their home if they prefer. All families who express an interest in taking part but are not selected for an interview will be contacted via telephone or email to thank them for their interest in the study.

All interviews will be conducted by a researcher who both have proven skills in the conduct of research in sensitive settings. Any distress during the interviews will be managed with care and compassion and participants will be free to decline to answer any questions that they do not wish to answer or to stop the interviews at any point. Any such families will be supported in obtaining appropriate help and, after discussion with the family, the lead clinician responsible for the child's care will be informed to offer any support. Interviews will be conducted until data saturation point is achieved (approximately 15-25 based on other studies). All interview participants will be sent a £20 shopping voucher to thank them for their time. Sites will be informed when recruitment to interviews can stop.

Parent recruitment in season 2

For peer review only - http://bmjopen.bmj.com/site/about/guidelines.xhtml

In season 2, all parents will be asked to complete a brief questionnaire. This will be the same questionnaire as used in season 1 and include parents who decline informed consent. Questionnaire findings will help evaluate parents' views on recruitment and consent, including any changes made to our recruitment approach following season 1.

Staff recruitment in seasons 1 and 2

All staff involved in trial conduct, recruitment and consent in participating sites will be sent an email invitation and information sheet to request their participation in an online questionnaire towards the end of season 1 and 2. Completion of the questionnaire will be taken as an indication of consent.

Findings from parent questionnaires, interviews and staff surveys in season 1 will be used to develop approaches to consent, recruitment, trial information materials and staff training in season 2. Findings from season 2 parent and staff questionnaires will inform the design/training for the approach to recruitment and consent in season 3.

12.1.1 Parent and staff perspectives sub study analysis

Analysis of data from the mixed method sub study will be assisted using NVivo 8 qualitative data analysis package and SPSS software for statistical analysis. Quantitative analysis will involve simple descriptive statistics and the chi-square test for trend. Qualitative data from will be analysed thematically (32). Data from study methods will be analysed separately then synthesised (33) through the use of constant comparative analysis (34).

12.2 Work Package C:

Understanding the mechanism of efficacy or failure for exogenous surfactant to modulate bronchiolitis disease requires an integrated investigation of the subject's alveolar environment. This includes identifying the infective aetiology, describing the local inflammatory response, and defining the kinetics of surfactant lipid and protein synthesis; all linked to natural history, trial intervention and participant outcomes. The selected mechanistic sub studies leverage the unique collaboration between experts in bronchiolitis pathogenesis at Liverpool and surfactant physiology at Southampton. While the sub studies are defined individually, their outputs integrate to provide a cogent thesis.

Hypotheses to test include:

At all sites by analysis of BAL fluid sampled just prior to intervention at 0hr and 24hr:

- Surfactant concentration and composition predicts duration of MV.
- Recovery of synthesis of surfactant proteins A & D is modulated by exogenous surfactant.
- Lower airway inflammation is modulated by exogenous surfactant.[3]
- Treatment failure is associated with detection of specific pathogens.

[3] Costs for this component of BESS will not be charged to NIHR EME.

For peer review only - http://bmjopen.bmj.com/site/about/guidelines.xhtml

At Liverpool & Southampton by giving a single intravenous dose of deuterium-labelled[4] choline (*methyl*-D_9-choline chloride (D_9-Choline)) and with sampling of BAL fluid and blood just prior to intervention at 0hr, 12hr, 24hr and 36hr.

- Synthesis of endogenous surfactant phosphatidylcholine is modulated by the provision of exogenous surfactant.

12.2.1 Metabolic profiling of surfactant composition and kinetics

Analysis of surfactant phospholipid and protein concentration, composition and synthesis in BAL fluid from ventilated infants will help provide an explanation for the beneficial effects, or otherwise, of exogenous surfactant therapy for acute lung injury in infants.
The sub-study of the metabolic profiling of surfactant composition and kinetics has two linked parts. The first "**Surfactant concentration and composition**" utilises BAL fluid collected as part of the whole trial at any site and does not involve any additional intervention or sampling procedures. The second part "**Kinetics of surfactant phosphatidylcholine synthesis"** is a more complex study that requires additional interventions, specific consent and consequently will recruit only from Liverpool and Southampton

12.2.1.1 Surfactant concentration and composition

This will address the hypothesis that: Surfactant concentration and composition predicts duration of MV.
Infants will be recruited to this part of the mechanistic sub-study from all BESS trial centres. Individual molecular species of phosphatidylcholine (PC), phosphatidylglycerol (PG) phosphatidylinositol (PI) and sphingomyelin (SM) will be analysed in aliquots of small volume BAL fluid samples taken prior to trial intervention at 0 and 24hr hours of the study if still intubated. These phospholipids will be analysed by electrospray ionisation mass spectrometry (ESI-MS) (29, 30) in Southampton correcting for sample dilution by urea analysis(28). Surfactant proteins A, B, C, and D will be analysed by ELISA and western blotting.

12.2.1.2 Kinetics of surfactant phosphatidylcholine synthesis

This will address the hypothesis that: Synthesis of endogenous surfactant phosphatidylcholine is modulated by the provision of exogenous surfactant.
This part of the mechanistic sub-study will be conducted at Liverpool and Southampton trial sites only. Infants at these two centres will receive a single intravenous dose of D_9-choline (3.6 mg/kg body weight) prior sampling of BAL fluid and trial intervention at time = 0 hours, followed by further BAL fluid sampling at 12, 24, 36 and 48 hours if still ventilated. A single intravenous dose of deuterium-labelled choline administered prior to intervention allows assay of endogenous surfactant synthesis (31). A small blood sample (1.25ml (range 1.0 to 1.3 ml)) will also be taken at these times. D_9-choline contains the stable isotope deuterium, which is non-radioactive and non-toxic. Choline is an essential vitamin and the dose is within the daily-recommended allowance. Investigators at University of Southampton have extensive experience administering this metabolic probe in clinical

[4] Deuterium is one of the two stable (non-radioactive) and naturally occurring isotopes of hydrogen that is used as a harmless metabolic tracer in animal and human studies.

For peer review only - http://bmjopen.bmj.com/site/about/guidelines.xhtml

studies of: preterm infants, infants with acute lung injury (ALI), and adults with acute respiratory distress syndrome patients with no adverse effects. ESI-MS analysis of unlabelled PC and PC labelled by incorporation of D_9-choline will provide a measure of surfactant phospholipid synthesis and turnover.

12.2.2 Recovery of surfactant proteins A & D (SP-A & SP-D) is modulated by exogenous surfactant.

Presence of SP-A and SP-D in the plasma has been proposed as a biomarker for acute lung injury (32). Among children mechanically ventilated for ALI including in bronchiolitis, we have previously shown reduced levels of SP-A and SP-D in the early stages of the disease, with recovery in SP-D levels in BAL fluid linked to progress to extubation, along with normalisation of DPPC and PG levels (33).

It is thought that surfactant deficiency contributes to acute lung injury by direct disruption of type II cell function and enhanced degradation of the surfactant system. SPA is secreted along with the phospholipid components of surfactant in lamellar bodies. Thus, metabolic studies demonstrating decreased PL secretion imply decreased SP-A secretion. However, this needs to be confirmed by direct measurement from secretions in the airways in a well-characterised population sampled under controlled conditions.

We will measure SP-A and SP-D by enzyme linked immunometric assay (ELISA) and Western blot at 0hr and 24hr sampling time points from subjects at all sites and in addition at 12hr, and 36hr from subjects at Liverpool and Southampton if still ventilated. We will compare levels over time relative to baseline, group-wise according to intervention.

No previous study has had the opportunity to relate the levels of SP-A & SP-D to surfactant metabolism during the natural history of bronchiolitis disease (placebo arm) or when modified by administration of exogenous surfactant. This proposal will evaluate not only the presence or absence of SPs but also provide evidence for their role as markers of lung recovery in the BAL.

12.2.3 Lower airway inflammation is modulated by exogenous surfactant[5]

Our previous study (3 arms, 27-plex, n=301) on local airway response to RSV bronchiolitis found high levels of IL1b, IL15, IL13, MIP1b, MCP1, IL-8 & PDGF and low levels of IFNg, IP10 & RANTES associated with life-threatening bronchiolitis (all $p<0.025$) (34). Subject to other funding, these 10 markers will be measured in BAL at t=0hr and 24hr in all subjects. Comparison will be made between baseline levels and second sample in infants treated with surfactant and placebo. Correlation will be sought between the inflammatory markers and levels of surfactant lipid and proteins.

12.2.4 Treatment efficacy or failure is associated with detection of specific pathogens.

We and others have demonstrated that certain pathogens and multiple infections are associated with poor outcome (35). Using accredited diagnostic polymerase chain reaction (PCR) multiplex assays we will assay BAL sampled daily for multiple pathogens and seek any relation with pathogens detected and outcome. While RSV infection accounts for about 75% of severe cases of

[5] Costs for this component of BESS are not being charged to NIHR EME.

For peer review only - http://bmjopen.bmj.com/site/about/guidelines.xhtml

bronchiolitis, dual viral infection or viral infection in combination with bacterial infections can account for severe disease. The aetiology of bronchiolitis in each subject must be identified to test for any pathogen specific effect.

12.2.5 Mechanistic Protocol

12.2.5.1 Surfactant phospholipid and protein concentration and composition (all sites)

Small volume lung lavage (0.9% NaCl, 1ml/kg body weight) will be performed in all participating sites at t=0, 24 just prior to trial intervention, and separated for protein and lipid analysis. For lipid analysis, butylated hydroxytoluene (10 of 20g/l in ethanol) will be added to all samples to prevent sample oxidation. Samples will be centrifuged at 400xgx10 min and the supernatant stored in aliquots for surfactant analysis at -80°C at each site. Other BAL fluid aliquots will be used for the inflammation and infection mechanistic subprojects. At the end of each winter season, all samples will be sent to Southampton for surfactant analysis.
Protein analysis will be conducted by standard techniques of ELISA and Western blotting. For PL analysis, samples will be lipid extracted in Southampton using organic solvents after addition of internal quantification standards and analysed by direct infusion ESI-MS. Analytes to be measured using diagnostic precursor scanning will include individual molecular species of PC, LysoPC, PG, PI and SM. Results will be expressed as % composition within an individual lipid class, as % composition of all measured lipids and as concentration (nmol/ml). Analysis of BAL fluid urea by colorimetric enzymatic assay will be used as a correction factor for BAL fluid dilution. While this in not ideal, a previous study indicates that it provides a reasonable correction for single recovery small volume lavage in ventilated infants (28).

Research Questions

Does administration of exogenous surfactant (poractant alfa) restore the dysfunctional composition of surfactant phospholipid characteristic of infants ventilated for bronchiolitis?
A power calculation from the pilot data at t=48h indicates that it will require n=49/group to achieve significance at p<0.05 for %DPPC of 38.8% and 43.5% respectively for infants in the placebo and treated groups. This is a realistic goal as this represents obtaining appropriate samples from 37% of prospective infants in the study
Do PC and PG concentrations in BAL fluid increase with duration of ventilation in the placebo group of infants? This analysis will also address whether the correlation between initial rate of incorporation and time to recruitment is related to secretion of labelled PC into a larger surfactant pool or decreased surfactant secretion with duration of ventilation.

12.2.5.2 Kinetics of surfactant synthesis

The mechanistic study of phosphatidylcholine surfactant synthesis will take place only at Liverpool and Southampton, which both have the appropriate infrastructure (staff and facility) to collect and process BAL more frequently. It will incorporate the phospholipid concentration and composition protocol outlined above, combined with a stable isotope labelling protocol. With specific consent recruited infants at both sites, in both placebo and treated groups, will be intravenously infused between t=0 and 1h with non-radioactive, stable isotope-labelled *methyl*D_9-choline chloride at 3.6 mg/kg. Small volume lung lavages will be collected as above, with additional time points at t=12h and 36h. EDTA blood samples (1.25ml (range 1.0 to 1.3 ml)) will be taken at the same time points and centrifuged at 400xgx10min to isolate plasma, which will be stored at -80°C. Samples will be

For peer review only - http://bmjopen.bmj.com/site/about/guidelines.xhtml

couriered from Liverpool to Southampton at the end of the winter season and sample extraction and processing will follow exactly the same protocol as above. Lipid extracts from both BAL fluid and plasma will be subjected to the identical ESI-MS analytical protocol described above, with the addition of a diagnostic scan for *methyl*-D_9-labelled PC. Enrichment of methylD9-choline in plasma choline will be analysed by LC-MS/MS using a MRM protocol to provide an indication of substrate choline turnover. Urea will be analysed by colorimetric enzymatic assay in plasma as well as in BAL fluid, to enable calculation of analyte concentrations per ml of epithelial lining fluid (ELF). Results will be calculated, in addition to those described above, as enrichment (Σ abundance of labelled PC species/Σ abundance of labelled + unlabelled PC species), fractional synthetic rates and concentration of labelled species (nmol/mL ELF).

12.2.5.3 Outcomes

The major discriminators between treatment and placebo groups will be the concentration of newly synthesised and secreted *methyl*-D_9 molecular species calculated as nmoles/ml of ELF after correction for urea dilution between BAL fluid and plasma and the FSR. Power: based on our pilot data on fractional synthetic rate of surfactant in two disease states (0.039±0.005 vs 0.030±0.011) 2 tail, a=0.05 requires total n=25/group for 1-b >0.95.
Enrichment of label in molecular species of plasma PC and in plasma choline will provide comparator data to distinguish responses that are lung specific from those determined by variability in substrate availability and metabolism.
Administration of a second dose of exogenous surfactant at t=12 hours in the treated infants will permit calculation of endogenous and exogenous surfactant pool sizes by labelled surfactant dilution.

For peer review only

For peer review only - http://bmjopen.bmj.com/site/about/guidelines.xhtml

13 REGULATORY AND ETHICAL APPROVALS

13.1 Statement of Compliance

Statement of compliance: The study will be carried out in accordance with:

- The World Medical Association Declaration of Helsinki ,
- CTRC Standard Operating Procedures
- Principles of Good Clinical Practice
- The template content is structured consistent with the SPIRIT (Standard Protocol Item: Recommendations for Interventional Trials 2013)
- UK Policy Framework for Health and Social Care Research

SI /EU Regulation	Title	Main impact/scope
2001/20/EC	The EU Clinical Trials Directive	National Competent Authority<br>Ethics Framework<br>GCP legal requirement<br>Good Manufacturing Practice<br>Protocol/Amendments/safety<br>Protection of Vulnerable Groups<br>Consent / Data protection
2004/1031	Medicines for Human use Clinical Trials Regulation	Transposed EU CT Directive in UK
2005/28/EC	EU Good Clinical Practice (GCP) Directive	Investigator brochure<br>Archiving<br>Mandatory training for trial teams
2006/1928	Amends 2004/1031	Investigator brochure /essential documents<br>Serious Breach<br>Declaration of Helsinki 1996 version for CTIMP
2006/2984	Amends 2004/1031	Consent for incapacitated adult by legal representative or emergency deferred consent
2008/941	Amends 2004/1031	Blood safety and quality<br>Emergency Deferred consent for children
2009/1164	Miscellaneous Amendment	Urgent Safety measures
2009/3063	Amends 2004/1031	Nurse and pharmacists to prescribe unlicensed medicines

13.2 Regulatory Approval

This trial falls within the remit of the EU Directive 2001/20/EC, transposed into UK law as the UK Statutory Instrument 2004 No 1031: Medicines for Human Use (Clinical Trials) Regulations 2004 as amended.

For peer review only - http://bmjopen.bmj.com/site/about/guidelines.xhtml

BESS will be registered with the MHRA and a Clinical Trials Authorisation (CTA) will be obtained prior to the trial commencing.

13.3 Ethical Considerations

The study will abide by the principles of the World Medical Association Declaration of Helsinki.

In compliance with GCP, parents or those with parental responsibility will be fairly informed of the trial, risks, benefits, and alternatives, asked to ensure they understand how it relates to their infant, and make a voluntary decision about entry. Autonomy, privacy and welfare will be paramount, overriding the research. Parents will be kept informed of findings that might cause them to withdraw, without detriment to on-going care.

Consent in emergency setting poses particular challenges for parents. We have allowed a window of 48 hours from intubation to intervention during which to seek consent and randomisation. Dr Kerry Woolfall will lead the sub study to explore staff and parents' acceptability of our approach to consent in this challenging setting. Her work includes CONNECT study guidance, adopted by NIHR Clinical Trials Toolkit (http://www.ct-toolkit.ac.uk/routemap/informed-consent), which helps to ensure recruitment and consent to critical care trials is ethically appropriate and addresses the needs critically ill children and their families (49, 50). The trial team are experienced at conducting studies and trials in emergency settings.

The acceptability of medicines derived from pigs to families of the Jewish and Islamic faiths has been addressed with Rabbi Adler, Director of the Kashrus & Medicines Information Service; the Ashkenazi Beth Din of the United Synagogue & the Union of Orthodox Hebrew Congregations and with Dr Shafi, Chairman of the Research & Documentation Committee of the Muslim Council of Great Britain.

These religious authorities recognise that some scholars and families will have their own interpretations of their faith, as do other faiths with regards to this topic, which further complicates the issue. BESS will liaise with these authorities during the development of parent information and consent materials.

13.4 HRA Approval

BESS will come under the Health Research Authority (HRA) Approvals process as part of the initial REC submission and will follow the HRA processes for submission of amendments to REC and the HRA.

13.5 Ethical and Local Governance Approval

Prior to the trial being initiated at CTRC, a favourable ethical opinion will be obtained from a REC and global governance approval from the HRA. Prior to opening a centre to recruitment, CTRC will ensure that local governance approval has been obtained: for sites in England and Wales, this will be "Capacity & Capability" Confirmation; for sites in Scotland and Northern Ireland, this will be R&D Approval.

For peer review only - http://bmjopen.bmj.com/site/about/guidelines.xhtml

13.6 Protocol Deviation and Serious Breaches

Incidence of protocol non-compliance, whether reported prospectively (e.g. where a treatment cannot be administered at a scheduled time) or retrospectively noted (e.g. as a result of central monitoring) are recorded as protocol deviations, the incidence of which are monitored and reported to trial oversight committees.
A breach of the protocol or GCP is ‘serious’ if it meets the regulatory definition of being “likely to affect to a significant degree the safety or physical or mental integrity of the trial participants, or the scientific value of the trial”. All confirmed serious breaches of GCP or protocol will be reported to the MHRA and REC in an expedited manner by the CTRC on behalf of the sponsor.

If any persons involved in the conduct of the trial become aware of a potential serious breach, they must immediately report this to the CTRC who will in turn notify the Sponsor. The Sponsor will assess the breach and determine if it meets the criteria of a ‘serious’ breach of GCP or protocol and therefore requires expedited reporting to the MHRA and REC.

In determining whether or not the breach is likely to affect to a significant degree the safety, physical or mental integrity of participants, the Sponsor may seek advice from medical expert members of the Trial Management Group (TMG) and/or of the independent oversight committees (IDSMC and TSC). In determining whether or not the breach is likely to significantly affect the scientific value of the trial, the Sponsor may seek advice from the Trial Statistician. However, the Sponsor retains responsibility for the assessment of whether or not a breach meets the definition of ‘serious’ and is subject to expedited reporting to MHRA and REC.

Breaches confirmed as ‘serious’ will be reported to the MHRA and REC within 7 days by the CTRC and notified to the TMG, IDSMC and TSC at their next meetings.

Any requests for additional information from the Sponsor, TMG, TSC, IDSMC, REC or MHRA will be promptly actioned by the relevant member(s) of the research team and open communication will be maintained to ensure appropriate corrective actions are taken and documented.

Other incidences of protocol non-compliance will be recorded as protocol deviations, the frequency of which will be monitored and reported to the trial oversight committees.

13.7 Study Discontinuation

In the event that the BESS trial is discontinued, any participants currently participating will not be given their next dose of poractant alfa or placebo. Decisions on weaning and extubation will revert to standard care.

Reporting of adverse events will continue as per trial protocol.

For peer review only

For peer review only - http://bmjopen.bmj.com/site/about/guidelines.xhtml

14 DATA MANAGEMENT AND TRIAL MONITORING

Details of the monitoring to be carried out for the BESS trial are included in the BESS Trial Monitoring Plan, maintained separately to this protocol.
Trial Oversight Committees related to the monitoring of the trial are detailed in section 15.4.

14.1 Source Documents

In order to resolve possible discrepancies between information appearing in the case report form (CRF) and any other participant related documents, it is important to know what constitutes the source document and therefore the source data for all information in the CRF.

Source data is defined as: “All information in original records and certified copies of original records of clinical findings, observations, or other activities in a clinical trial necessary for the reconstruction and evaluation of the trial. Source data are contained in source documents (original records or certified copies).” (ICH E6, 1.51).

Source documents are defined as: “Original documents, data, paper and electronic records (e.g. hospital records, clinical and office charts, laboratory notes, memoranda, participant diaries or evaluation checklists, pharmacy dispensing records, recorded data from automated instruments, copies or transcriptions certified after verification as being accurate copies, microfiches, photographic negatives, microfilm or magnetic media, x-rays, participant files, and records kept at the pharmacy, at the laboratories and at medico-technical departments involved in the clinical trial).” (ICH E6, 1.52).

The CRF will be considered the source document for data where no prior record exists, and which is recorded directly in the CRF. A BESS source document list will be produced for each site.

Date(s) of conducting the informed consent process including date of provision of patient information, confirmation of full eligibility, randomisation number and the fact that the patient is participating in a clinical trial (including possible treatment arms) should be added to the patient’s medical record chronologically.

14.2 Data Capture Methods

The study case report form (CRF) is the primary data collection instrument for the study. All data requested on the CRF must be recorded. All missing data must be explained.

Please ensure documents are sent by sites separate to the consent form as this has patient identifiable data on it.

The LRSQ will be completed by participants’ parents at 6m and 12m after randomisation. These are completed online but paper copies will be available on request for parents without internet access. If paper questionnaires are used **sites should photocopy them** in order to retain a copy at site before mailing originals to CTRC.

BESS will also be collecting data from the Paediatric Intensive Care Audit Network (PICANet). PICANet is a secure confidential high-quality clinical database, recording details of the treatment of all critically ill children in PICUs. Collection of personally identifiable data

For peer review only - http://bmjopen.bmj.com/site/about/guidelines.xhtml

has been approved by the Patient Information Advisory Group (now the NHS Health Research Authority Confidentiality Advisory Group) and ethical approval granted by the Trent Medical Research Ethics Committee.
PICANet will provide a bespoke download page for the trial and sites will regularly download their own data for consented BESS patients only and transfer this to the CTRC.

14.3 Monitoring

14.3.1 Risk Assessment

This trial will undergo a risk assessment, completed in partnership between:

- Representative/s of the Trial Sponsor
- Chief Investigator
- CTRC Trial Coordinator and Supervising Trial Manager
- CTRC Trial Statistician and Supervising Statistician
- CTRC Information Systems and Data Management teams
- CTRC Director

In conducting the risk assessment, the contributors will consider potential participant, organisational and study hazards, the likelihood of their occurrence and resulting impact should they occur.

Monitoring for BESS will be informed by the trial specific risk assessment and will be conducted as per a detailed Trial Monitoring Plan (TMP), maintained separately to this protocol and stored in the TMF. The TMP will describe who will conduct the monitoring, at what frequency monitoring will be done and what level of detail monitoring will be conducted.

14.3.2 Central Monitoring

Data received at CTRC will be checked for missing or unusual values (range checks) and checked for consistency over time. Any suspect data will be returned to the site in the form of data queries. Data query forms will be produced at the CTRC from the trial database and sent either electronically or through the post to a named individual (as listed on the site delegation log). Sites will respond to the queries providing an explanation/resolution to the discrepancies and return the data query forms to CTRC. The forms will then be filed along with the appropriate CRFs and the appropriate corrections made on the database. There are a number of monitoring features in place at the CTRC to ensure reliability and validity of the trial data, to be detailed in the TMP maintained separately from this protocol.

Central checks of consent will be completed for each participant to ensure the completeness of consent and that the timing of consent is in line with the protocol.

Central monitoring will be performed in a proportionate manner as informed by the Trial Risk Assessment and Monitoring Plans.

For peer review only - http://bmjopen.bmj.com/site/about/guidelines.xhtml

14.3.3 Clinical Site Monitoring

In order to perform their role effectively, the Trial Coordinator, Data Manager and persons involved in Quality Assurance and Inspection may need direct access to primary data during monitoring visits, e.g. participant medical records, laboratory reports, appointment books, etc. Since this affects the participant's confidentiality, this fact is included on the PISC.
The purposes of monitoring visits, if triggered as per CTRC SOPs, include but are not limited to:

- assessing compliance with the study protocol
- discussing any emerging problems that may have been identified prior to the visit
- checking CRF and query completion practices

14.4 Confidentiality

Individual participant medical information obtained as a result of this study is considered confidential and disclosure to third parties is prohibited with the exceptions noted below.
CRFs will be pseudonymised – they will not contain details of participant names and will be labelled with the unique trial randomisation and/or screening number. Medical information may be given to the participant's medical team and all appropriate medical personnel responsible for the participant's welfare.

Verification that appropriate informed consent is obtained will be enabled by the provision of copies of participant's signed informed consent forms being supplied to the CTRC by recruiting centres, which requires that name data will be transferred to the CTRC.
The CTRC will be undertaking activities requiring the transfer of identifiable personal data. The transfer of this data is disclosed in the PISC. The CTRC will be responsible for sending links to the LRSQ to trial participants at 6m and 12m post randomisation, and therefore will be required to receive contact details including name and email address. Should the parent require a paper copy this will be sent by the recruiting site as the CTRC will not collect participant addresses.
All documentation received at CTRC which includes direct identifiers such as names and email addresses (e.g. consent forms) will be stored securely and separately to all other pseudonymised participant data (e.g. medical CRFs).
The CTRC will preserve the confidentiality of participants taking part in the study and The University of Liverpool is registered as a Data Controller with the Information Commissioners Office.

14.5 Quality Assurance (QA) and Quality Control (QC)

Quality Assurance (QA) includes all the planned and systematic actions established to ensure the trial is performed and data generated, documented, recorded and reported in compliance with applicable regulatory, ethical and governance requirements. Quality Control (QC) includes the operational techniques and activities performed within the QA system to verify that the requirements for quality of the trial-related activities are fulfilled: e.g. state what clinical site monitoring (and audit) is planned, if any. In accordance with the monitoring plan site visits will or will not be conducted and source verification performed if central monitoring processes indicate these are required. Monitoring activities could include, but are not limited to the following:

- The Trial Coordinator at the CTRC will verify appropriate approvals are in place prior to initiation of a site and the relevant personnel have attended trial specific training. A greenlight

For peer review only - http://bmjopen.bmj.com/site/about/guidelines.xhtml

checklist will verify all approvals are in place prior to trial initiation at CTRC and the individual site.

- The TMG will determine the minimum key staff required to be recorded on the delegation log in order for the site to be eligible to be initiated.
- Data will be evaluated for compliance with protocol and accuracy in relation to source documents.
- The study will be conducted in accordance with procedures identified in the protocol.
- Independent oversight of the trial will be provided by the Data and Safety Monitoring Committee and independent members of the Trial Steering Committee.
- The types of materials to be reviewed, who is responsible, and the schedule for reviews may be specified or referenced in other documents.
- Types and mechanisms of training of staff for the study should be specified.
- The PI and other key staff from each centre will attend site initiation training, coordinated by the CTRC, which will incorporate elements of trial-specific training necessary to fulfil the requirements of the protocol.
- The Trial Management Group will monitor screening, randomisation and consent rates between centres.
- The process for consent, recruitment and randomisation will be evaluated for compliance with the protocol.
- Data quality checks and monitoring procedures will be undertaken in line with the trial Data Management Plan.
-

14.6 Records Retention

All trial documents will be retained for a maximum period of 25 years from the End of Trial.

The site PI is responsible for archiving of all relevant source documents so that the trial data can be compared against source data after completion of the trial (e.g. in case of inspection from authorities). The PI at each trial site must make arrangements to store the essential trial documents, (as defined in Essential Documents for the Conduct of a Clinical Trial (ICH E6, Guideline for GCP)) including the Investigator Site File and Pharmacy Site File, until the CTRC informs him/her that the documents are no longer to be retained. The PI is required to ensure the continued storage of the documents, even if s/he, for example, leaves the clinic/practice or retires before the end of required storage period. Delegation must be documented in writing.

All other persons and organisations involved in the trial will be responsible for storing and archiving the parts of the TMF relevant to their delegated duties (e.g. laboratories, IMP manufacturers and distributors, third-party vendors providing randomisation and IMP allocation systems, etc.).

The CTRC undertakes to store originally completed CRFs for the same period, except for source documents pertaining to the individual investigational site, which are kept by the site PI only. The CTRC will archive the documents in compliance with the principles of GCP . All electronic CRFs and trial data will be archived onto an appropriate media for long term accessible storage. Hard copies of data will be boxed and transferred to secure premises where unique reference numbers are applied to enable confidentiality, tracking and retrieval.

For peer review only - http://bmjopen.bmj.com/site/about/guidelines.xhtml

15 INDEMNITY

BESS is sponsored by The University of Liverpool and co-ordinated by the CTRC in the University of Liverpool. The Sponsor does not hold insurance against claims for compensation for injury caused by participation in a clinical trial and they cannot offer any indemnity. As this is an investigator-initiated study, The Association of the British Pharmaceutical Industry (ABPI) guidelines for patient compensation by the pharmaceutical industry do not apply. However, in terms of liability, NHS Trust and Non-Trust Hospitals have a duty of care to patients treated, whether or not the patient is taking part in a clinical trial, and they are legally liable for the negligent acts and omission of their employees. Compensation is therefore available in the event of clinical negligence being proven.

The Sponsor does not accept liability for any breach in any other hospital's duty of care, or any negligence on the part of employees of hospitals. This applies whether the hospital is an NHS Trust or not.

The Sponsor has vicarious liability for the actions of its staff, when through the course of their employment they are involved in the design and initiation of a clinical trial, including but not limited to the authorship of the Clinical Trial Protocol. The University of Liverpool has appropriate insurance in place to cover this liability.

All participants will be recruited at NHS sites and therefore the NHS indemnity scheme/NHS professional indemnity will apply with respect to claims arising from harm to participants at site management organisations.

Clinical negligence is defined as:

"A breach of duty of care by members of the health care professions employed by NHS bodies or by others consequent on decisions or judgments made by members of those professions acting in their professional capacity in the course of their employment, and which are admitted as negligent by the employer or are determined as such through the legal process".

For peer review only

For peer review only - http://bmjopen.bmj.com/site/about/guidelines.xhtml

16 ROLES AND RESPONSIBILITIES

16.1 Role of Study Sponsor and Study Funder

The Sponsor of this trial is the University of Liverpool. The Sponsor will ensure that clear agreements are reached, documented and carried out, respecting the dignity, rights, safety and wellbeing of participants and the relationship with healthcare professionals. This will provide for proper design, management, initiation, conduct, monitoring, data collection, data analysis, data protection, financing and reporting of this trial meeting appropriate scientific, legal and regulatory standards. The responsibility for design, conduct, management, data analysis, data interpretation, manuscript writing, and dissemination of results is delegated to the Trial Management Group.

The funders of this study are the National Institute for Health Research and Medical Research Council who are providing financial funding through their Efficacy and Mechanism Evaluation Programme. The EME Programme is funded by the MRC and NIHR, with contributions from the CSO in Scotland and Health and Care Research Wales and the HSC R&D Division, Public Health Agency in Northern Ireland.

The funders will ensure there is proper use of the funds they have allocated to provide value for money. The funders assure the quality of the study, taking the lead in establishing that the research proposal is worthwhile, of high scientific quality, has an appropriate research infrastructure with expert clinical trial management, has the capacity to comply with the principles of GCP and represents good value for money.

Chiesi Farmaceutici S.p.A (Chiesi) are supplying the IMP endotracheal poractant alfa (Curosurf®) to be administered as trial IMP and will be securing use of a randomisation and re-supply system for use in the trial through an Investigator Initiated Clinical Trial Research Agreement between Chiesi and the Sponsor. Chiesi are not providing any financial funding in connection with this Agreement.

16.2 Funding and Support in Kind

Funder(s)	Financial and Non-financial Support Given
National Institute of Health Research	Financial Funding (£1,655,615.46)
Chiesi Farmaceutici S.p.A (UK address: 333 Styal Road Manchester M22 5LG)	Supply of endotracheal poractant alfa (Curosurf®), and contracting of a third party distributor who will also provide a randomisation and drug resupply system

For peer review only - http://bmjopen.bmj.com/site/about/guidelines.xhtml

16.3 Protocol Contributors

Name	Affiliations	Contribution to protocol
Calum Semple	Institute of Translational Medicine, University of Liverpool and Alder Hey Children's Hospital NHS Foundation Trust	Inception of study, led the writing of this protocol, clinical and scientific arrangements, trial design and conduct
Tracy Moitt	CTRC, University of Liverpool	Protocol development, governance arrangements and trial conduct
Chloe Donohue	CTRC, University of Liverpool	Protocol development, governance arrangements and trial conduct
Ashley Jones	CTRC, University of Liverpool	Led statistical arrangements, trial design and conduct
Laura Sutton	CTRC, University of Liverpool	Statistical arrangements, trial design and conduct
Kerry Woolfall	Institute of Psychology, Health & Society, University of Liverpool	Led design of parent/staff experience and consent study (WP-B)
Tony Postle	Faculty of Medicine, University of Southampton	Collaborator from inception, design and development of mechanistic study (WP-C)
Howard Clark	Faculty of Medicine, University of Southampton	Collaborator from inception, design and development of mechanistic study (WP-C)
Jens Madsen	University Hospital Southampton NHS Foundation Trust	Design and development of mechanistic study (WP-C)
John Pappachan	University Hospital Southampton NHS Foundation Trust	Design and development of mechanistic study (WP-C)
Paul Ritson	Alder Hey Children's Hospital NHS Foundation Trust	Development of BAL procedures, intervention administration/ handling, and PICU processes
Mark Turner	Institute of Translational Medicine, University of Liverpool and Liverpool Women's Hospital NHS Foundation Trust	Inception of study, clinical and scientific arrangements, trial design and liaison with pharmaceutical company
Katie Neville	CTRC, University of Liverpool	Quality Assurance review
Guido Varoli	Chiesi Farmaceutici S.p.A.	Limited to provision of information relating to IMP specifics (formulation, packaging, administration, distribution etc.)

Advice was received from all Co-Applicants to the NIHR EME funding applications and protocol submissions.

The design, conduct, data analysis, data interpretation, manuscript writing and dissemination of results are the responsibility of the TMG, subject to the approval of the University of Liverpool as Sponsor, the MHRA and National Institute for Health as Funders.
Chiesi Farmaceutici S.p.A. are responsible for supplying the IMP and subcontracting an independent third party to undertake distribution of the IMP and development, management and maintenance of a randomisation and drug resupply system.

For peer review only - http://bmjopen.bmj.com/site/about/guidelines.xhtml

16.4 Trial Committees

16.4.1 Trial Management Group (TMG)

A Trial Management Group (TMG) will be formed comprising the Chief Investigator, other lead investigators (clinical and non-clinical), members of the CTRC and a lay member. The TMG will be responsible for the day-to-day running and management of the trial. Refer to the TMG terms of reference and trial oversight committee membership document for further details. Meetings will be in Liverpool with teleconferencing. The TMG will report to the TSC.

16.4.2 Trial Steering Committee (TSC)

The TSC will consist of an independent medical expert chairperson (neonatology), two independent medical experts (paediatric intensive care), an independent biostatistician and two lay members (one independent, one non-independent). TMG representatives will be given the opportunity to contribute as non-independent and non-voting TSC members. The role of the TSC is to provide overall supervision for the trial and provide advice through its independent Chairman. The ultimate decision for the continuation of the trial lies with the TSC.

The TSC will first convene prior to the start of recruitment and will then define frequency of subsequent meetings (at least annually). Refer to the TSC terms of reference and trial oversight committee membership document for further details.

16.4.3 Independent Data and Safety Monitoring Committee (IDSMC)

The IDSMC consists of an independent medical expert chairperson (neonatology), an independent expert in Paediatric Intensive Care and an independent expert in medical statistics.

The IDSMC will be responsible for reviewing and assessing recruitment, interim monitoring of safety and effectiveness, trial conduct and external data. The IDSMC will first convene prior to the start of recruitment and will then define frequency of subsequent meetings (at least annually). Details of the interim analyses and monitoring are provided in section 11.4 .

The IDSMC will provide a recommendation to the TSC concerning the continuation of the study. Refer to the IDSMC charter and trial oversight committee membership document for further details.

For peer review only - http://bmjopen.bmj.com/site/about/guidelines.xhtml

17 PUBLICATION AND DISSEMINATION

17.1 Publication Policy

The results from different centres will be analysed together and published as soon as possible. The design, conduct, data analysis, data interpretation, manuscript writing, and dissemination of results are the responsibility of the TMG on behalf of the University of Liverpool as Sponsor and the National Institute for Health as Funders.

The TMG will form the basis of the Writing Committee and advise on the nature of publications. The Uniform Requirements for Manuscripts Submitted to Biomedical Journals (http://www.icmje.org/) will be respected. The National Clinical Trial (NCT) identifier allocated to this trial will be attached to any publications resulting from this trial.

The members of the TSC and IDSMC will be listed with their affiliations in the Acknowledgements / Appendix of the main publication.

17.1.1 Authorship

Contributors to all four of (i) the design, conduct, data analysis and interpretation, (ii) writing, (iii) manuscript approval and (iv) accountability for the integrity of the work will, depending on their contribution and journal requirements, be included by name at the manuscript head or listed at the end in a by-line as members of the BESS Consortium which will also be named at the manuscript head.

17.2 Dissemination to Key Stakeholders

BESS intends to publish open access results in high impact journals (e.g. British Medical Journal, New England Journal of Medicine, Lancet, American Journal of Respiratory and Critical Care Medicine). The full trial report will also be published in the NIHR EME Journal.
BESS will also present at international meeting of the major Paediatric Intensive Care Societies, American Thoracic Society, European Respiratory Society.

The involvement of representatives of the UK Paediatric Intensive Care Society Study Group (PICSSG) as co-applicants will be key to ensuring dissemination of result to the Paediatric Intensive Care community.

At end of study, we will organise a symposium in partnership with the UK Paediatric Intensive Care Society where the results of the study will be presented alongside reports of family experiences as reported directly by parents. The symposium will be open to intensivists, respiratory physicians, study staff and any interest participating families.
We also intend to disseminate via a PPI event to be hosted in Liverpool at end of study with targeted open invitation to all families of participants.

We anticipate that because of the nature of the study population and the disease that our findings will be of interest to the general public. For this reason we will be proactive in targeting the broadcast media and press through our professional external media consultants, with due regard to the usual NIHR publicity and publication conditions.

For peer review only - http://bmjopen.bmj.com/site/about/guidelines.xhtml

Public dissemination will include contemporary social media including a study website and Twitter account.

17.1 Data Sharing

Individual Clinicians must undertake not to submit any part of their individual data for publication without the prior consent of the TMG. All publications shall include a list of participating PIs and collaborators.

For peer review only

DISCLAIMER:

The views expressed in this publication are those of the author(s) and not necessarily those of the MRC, NHS, NIHR or the Department of Health and Social Care.

For peer review only - http://bmjopen.bmj.com/site/about/guidelines.xhtml

18 CHRONOLOGY OF PROTOCOL AMENDMENTS

18.1 Version 1.0 (18/07/2018)

Original signed version. Not approved by MHRA.

18.2 Version 2.0 (28/09/2018)

**Summary of Amendments from Protocol V1.0 to V2.0**		
**Page Number**	**Section**	**Amendment**
Page 1	N/A	Research Ethics reference number added as now known.
Page 14	2.0	A known hypersensitivity to the active substance or any excipients of Curosurf® added to exclusion criteria.
Page 28	6.2	A known hypersensitivity to the active substance or any excipients of Curosurf® added to exclusion criteria.
Page 32	7.3	Randomisation procedure updated to include completion of unblinding envelope
Page 34	8.1.1	Reference to physical examination added
Page 35	8.1.3	Reference to physical examination added
Page 37	8.3	Schedule for physical examinations added to intervention & follow-up schedule (Table 2)
Page 41	8.5	Safety Assessment procedures updated to include physical examinations and recording of vital signs; new sub section 8.5.1 added to detail procedure for limited physical examination
Page 50-51	9.4	Detail added to unblinding section
Page 51	9.4.1	Emergency unblinding process updated to include use of unblinding envelopes
Page 53	10.5	Reference Safety Information updated from sections 4.3 to 4.9 of the Curosurf® SmPC to section 4.8 only.
Page 77	16.1	Full NIHR EME funding statement added.
Page 82	N/A	NIHR EME disclaimer added to bottom of page.

For peer review only

For peer review only - http://bmjopen.bmj.com/site/about/guidelines.xhtml

19 REFERENCES

1. Nair H, Nokes DJ, Gessner BD, Dherani M, Madhi SA, Singleton RJ, et al. Global burden of acute lower respiratory infections due to respiratory syncytial virus in young children: a systematic review and meta-analysis. Lancet. 2010;375(9725):1545-55.

2. Green CA, Yeates D, Goldacre A, Sande C, Parslow RC, McShane P, et al. Admission to hospital for bronchiolitis in England: trends over five decades, geographical variation and association with perinatal characteristics and subsequent asthma. Arch Dis Child. 2016;101(2):140-6.

3. Jat KR, Chawla D. Surfactant therapy for bronchiolitis in critically ill infants. Cochrane Database Syst Rev. 2015(8):CD009194.

4. Luchetti M, Casiraghi G, Valsecchi R, Galassini E, Marraro G. Porcine-derived surfactant treatment of severe bronchiolitis. Acta Anaesthesiol Scand. 1998;42(7):805-10.

5. Luchetti M, Ferrero F, Gallini C, Natale A, Pigna A, Tortorolo L, et al. Multicenter, randomized, controlled study of porcine surfactant in severe respiratory syncytial virus-induced respiratory failure. Pediatr Crit Care Med. 2002;3(3):261-8.

6. Tibby SM, Hatherill M, Wright SM, Wilson P, Postle AD, Murdoch IA. Exogenous surfactant supplementation in infants with respiratory syncytial virus bronchiolitis. Am J Respir Crit Care Med. 2000;162(4 Pt 1):1251-6.

7. McNamara PS, Ritson P, Selby A*, et al* Bronchoalveolar lavage cellularity in infants with severe respiratory syncytial virus bronchiolitis. Archives of Disease in Childhood. 2003;88**:**922-926.

8. von Neergaard K. Neue Auffassungen übereinen Grundbegriff der Atemmechanik. Die Retraktionskraft der Lunge, abhängig von der Oberfl ächenspannung in den Alveolen. (New views on a fundamental concept of respiratory mechanics. The retraction force of the lung is dependent on the surface tension within the alveoli.). Z Gesamt Exp Med 1929;66:373-94.

9. Goh BC, Rynkiewicz MJ, Cafarella TR, White MR, Hartshorn KL, Allen K, et al. Molecular mechanisms of inhibition of influenza by surfactant protein D revealed by large-scale molecular dynamics simulation. Biochemistry. 2013;52(47):8527-38.

10. LeVine AM, Lotze A, Stanley S, Stroud C, O'Donnell R, Whitsett J, et al. Surfactant content in children with inflammatory lung disease. Crit Care Med. 1996;24(6):1062-7.

11. Dargaville PA, South M, McDougall PN. Surfactant abnormalities in infants with severe viral bronchiolitis. Arch Dis Child. 1996;75(2):133-6.

12. Skelton R, Holland P, Darowski M, Chetcuti PA, Morgan LW, Harwood JL. Abnormal surfactant composition and activity in severe bronchiolitis. Acta Paediatr. 1999;88(9):942-6.

13. Kerr MH, Paton JY. Surfactant protein levels in severe respiratory syncytial virus infection. Am J Respir Crit Care Med. 1999;159(4 Pt 1):1115-8.

14. Hartmann F, Fiori HH, Ramos Garcia PC, Piva J, Fiori RM. Surfactant deficiency in infants with severe acute viral bronchiolitis. J Pediatr. 2014;164(6):1432-5.

15. Kelly E, Bryan H, Possmayer F, Frndova H, Bryan C. Compliance of the respiratory system in newborn infants pre- and postsurfactant replacement therapy. Pediatr Pulmonol. 1993;15(4):225-30.

For peer review only - http://bmjopen.bmj.com/site/about/guidelines.xhtml

16. Jat KR, Chawla D. Surfactant therapy for bronchiolitis in critically ill infants. Cochrane Database Syst Rev. 2012(9):CD009194.
17. NICE. Bronchiolitis in children: diagnosis and management (NG9). 2015.
18. Siegel LS. Correction for prematurity and its consequences for the assessment of the very low birth weight infant. Child Dev. 1983;54(5):1176-88.
19. Soll RF. Surfactant treatment of the very preterm infant. Biol Neonate. 1998;74 Suppl 1:35-42.
20. Powell CV, McNamara P, Solis A, Shaw NJ. A parent completed questionnaire to describe the patterns of wheezing and other respiratory symptoms in infants and preschool children. Arch Dis Child. 2002;87(5):376-9.
21. Paret G, Ziv T, Barzilai A, Ben-Abraham R, Vardi A, Manisterski Y, et al. Ventilation index and outcome in children with acute respiratory distress syndrome. Pediatr Pulmonol. 1998;26(2):125-8.
22. Bilan N, Dastranji A, Ghalehgolab Behbahani A. Comparison of the spo2/fio2 ratio and the pao2/fio2 ratio in patients with acute lung injury or acute respiratory distress syndrome. J Cardiovasc Thorac Res. 2015;7(1):28-31.
23. Wolfe R, Carlin JB. Sample-Size Calculation for a Log-Transformed Outcome Measure. Controlled Clinical Trials. 1999;20(6):547-54.
24. Menon K, Ward RE, Gaboury I, Thomas M, Joffe A, Burns K, et al. Factors affecting consent in pediatric critical care research. Intensive Care Med. 2012;38(1):153-9.
25. Kerrin A, Fitch P, Errington C, Kerr D, Waxman L, Riding K, et al. Differential lower airway dendritic cell patterns may reveal distinct endotypes of RSV bronchiolitis. Thorax. 2016.
26. McNamara PS, Fonceca AM, Howarth D, Correia JB, Slupsky JR, Trinick RE, et al. Respiratory syncytial virus infection of airway epithelial cells, in vivo and in vitro, supports pulmonary antibody responses by inducing expression of the B cell differentiation factor BAFF. Thorax. 2013;68(1):76-81.
27. McNamara PS, Ritson P, Selby A, Hart CA, Smyth RL. Bronchoalveolar lavage cellularity in infants with severe respiratory syncytial virus bronchiolitis. Arch Dis Child. 2003;88(10):922-6.
28. Semple MG, Dankert HM, Ebrahimi B, Correia JB, Booth JA, Stewart JP, et al. Severe respiratory syncytial virus bronchiolitis in infants is associated with reduced airway interferon gamma and substance P. PLoS One. 2007;2(10):e1038.
29. Bernhard W, Pynn CJ, Jaworski A, Rau GA, Hohlfeld JM, Freihorst J, et al. Mass spectrometric analysis of surfactant metabolism in human volunteers using deuteriated choline. Am J Respir Crit Care Med. 2004;170(1):54-8.
30. Dushianthan A, Goss V, Cusack R, Grocott MP, Postle AD. Altered molecular specificity of surfactant phosphatidycholine synthesis in patients with acute respiratory distress syndrome. Respir Res. 2014;15:128.
31. Bunt JE, Zimmermann LJ, Wattimena JL, van Beek RH, Sauer PJ, Carnielli VP. Endogenous surfactant turnover in preterm infants measured with stable isotopes. Am J Respir Crit Care Med. 1998;157(3 Pt 1):810-4.

For peer review only - http://bmjopen.bmj.com/site/about/guidelines.xhtml

32. Greene KE, Wright JR, Steinberg KP, Ruzinski JT, Caldwell E, Wong WB, et al. Serial changes in surfactant-associated proteins in lung and serum before and after onset of ARDS. Am J Respir Crit Care Med. 1999;160(6):1843-50.
33. Todd DA, Marsh MJ, George A, Henderson NG, Barr H, Sebastian S, et al. Surfactant phospholipids, surfactant proteins, and inflammatory markers during acute lung injury in children. Pediatr Crit Care Med. 2010;11(1):82-91.
34. Semple MG, Flanagan B, Smyth RL. Nasal Cytokines Associated With Householder Tobacco Smoking And HRSV Bronchiolitis. American Journal of Respiratory and Critical Care Medicine; December 2012: American Thoracic Society; 2012. p. A1026-A.
35. Semple MG, Cowell A, Dove W, Greensill J, McNamara PS, Halfhide C, et al. Dual infection of infants by human metapneumovirus and human respiratory syncytial virus is strongly associated with severe bronchiolitis. J Infect Dis. 2005;191(3):382-6.
36. Wright SM, Hockey PM, Enhorning G, Strong P, Reid KB, Holgate ST, et al. Altered airway surfactant phospholipid composition and reduced lung function in asthma. J Appl Physiol (1985). 2000;89(4):1283-92.
37. Postle AD, Heeley EL, Wilton DC. A comparison of the molecular species compositions of mammalian lung surfactant phospholipids. Comp Biochem Physiol A Mol Integr Physiol. 2001;129(1):65-73.
38. Mander A, Langton-Hewer S, Bernhard W, Warner JO, Postle AD. Altered phospholipid composition and aggregate structure of lung surfactant is associated with impaired lung function in young children with respiratory infections. Am J Respir Cell Mol Biol. 2002;27(6):714-21.
39. Wright JR. Immunoregulatory functions of surfactant proteins. Nat Rev Immunol. 2005;5(1):58-68.
40. LeVine AM, Gwozdz J, Stark J, Bruno M, Whitsett J, Korfhagen T. Surfactant protein-A enhances respiratory syncytial virus clearance in vivo. J Clin Invest. 1999;103(7):1015-21.
41. Rawat M, Chadrasekharan PK, Wiliams A, Gugino S, Koenigsknecht C, Swartz D, Ma CX, Mathew B, Nair J, Lakshminrusimha S. Oxygen Saturation Index and Severity of Hypoxic Respiratory Failure. Neonatology. 2015; 107: 161-166
42. BMJ 2015;350:h2305 [doi: https://doi.org/10.1136/bmj.h2305]

For peer review only

For peer review only - http://bmjopen.bmj.com/site/about/guidelines.xhtml

20 DOCUMENTS SUPPLEMENTARY TO THE PROTOCOL

Documents referenced herein as accompanying the protocol that are separately updated and version controlled are:

Patient information sheets and consent form
GP Letter
LRSQ questionnaires
Participating sites log
Trial Monitoring Plan
Statistical Analysis Plan
Trial Risk Assessment

For peer review only

For peer review only - http://bmjopen.bmj.com/site/about/guidelines.xhtml

BESS
Bronchiolitis Endotracheal Surfactant Study

Parent Interview Topic Guide
The **B**ronchiolitis **E**ndotracheal **S**urfactant **S**tudy (BESS)

Please note: *Italic text indicates instruction for researcher and will not be read to participant*

Intro: My name is [*researcher name*] and I am a researcher from the University of Liverpool. Many thanks for agreeing to speak to us about your child's hospital admission. We know that this must have been a difficult time and the purpose of this conversation is to get your feedback so we can insure that future parents …..

Check consent for audio recording

You can stop the interview at any time. Before we start do you have any questions?

Bereaved: I have some idea about your circumstances, if there is anything that you find difficult to talk about please don't feel that you have to, or if you want to stop the interview at any point, then please let me know.

The reason that you were invited to take part in this interview study is because if we only ask those parents whose children recovered from bronchiolitis the information we gather won't be complete. The findings will be biased. We know even less about what it is like for parents who have been bereaved to have discussions with doctors and nurses about the trial and what it was like to be involved in the trial. Does that make sense? Do you have any questions before I start? I will start with some questions about you if that's ok and then I will ask you about your experience of being invited to take part in BESS?

**Section 1: Background**	
1.1	Demographics:<br>Could I please ask you to tell me a few things about yourself and your family?<br>What is your DOB?<br>What is your occupation?<br>What is the first part of post code,<br>What would you describe as being your ethnic background,<br>What is your religion (if any)?<br>How many children do you have?<br>*If not known:* Please tell me a little bit about your child (*name, age, gender*)<br>Was your child born at full term or were they premature (If prem ask how many weeks gestation when born)

	**Not bereaved**<br><br>Has he/she recovered from his/her hospital visit? Has your child (any of your children) had any previous hospital admissions for bronchiolitis?
1.2	Could you give me an outline of what happened for them to need emergency treatment for bronchiolitis? *(if bereaved say:* I know this may be difficult, if you don't wish to answer this question please say and I will go on to the next question)<br><br>When were they admitted to hospital with bronchiolitis? *Prompt: which hospital* (prompt- explore if transferred)<br>How long after you arrived at hospital was your child transferred to the PICU? How long after they arrived at hospital/ PICU where they put on a ventilator? *(Prompt: explore if also received any forms of non-invasive ventilation: e.g. nasal CPAP and nasal high low oxygen and when)*
1.3	Before your admission, had you ever heard of bronchiolitis before? At what point where you told that your child had bronchiolitis? What did you think when you first heard that your child had bronchiolitis?<br>Did anyone explain to you about this disease, such as how long a child with bronchiolitis is usually sick for? (*Prompt: If yes explore what information parents received. If no, would you have liked a doctor or nurse to have provided you with information about bronchiolitis? BOTH Yes and NO- Explore what information they would have liked to have known about*)<br>Did anyone explain how your child would be cared for? (*Prompt: Explore any discussion of treatments)*
**Section 2: BESS recruitment and consent process**	
2.1	This is a question I ask all parents and it's not a test, but just so we can gage whether the trial is being explained clearly enough I wanted to know whether you;<br><br>Could you tell me what the BESS trial was looking at?
2.2	Please could you give me an overall picture of what happened when you first heard about the BESS trial… could you tell me a bit about that?<br><br>*Explore any knowledge about the trial before admission.*<br><br>*Explore where parents were/ when they first heard the trial mentioned.*<br><br>*Explore time frame- how long where you approached after your child was put on a ventilator?*
2.3	How / where were you introduced to the study? *(prompt hospital staff or PICU transport staff)*<br><br>Was it a doctor or nurse who spoke to you about the trial?

For peer review only

	Did one of the nursing staff looking after your child introduce you to the research nurse or doctor?
2.4	Did the research nurse/doctor check with you that it was a good time to talk about research?<br>If so, when was this? Do you think that this was the best time?<br>If not, when would have been the best time?<br>How could this be improved?
2.5	Could you tell me what they explained about BESS?<br>Did they go through any of the potential risks or benefits of your child taking part in the trial at that point? If yes, how did they describe these?<br>Was there anything that you found: a) unclear b) surprising?<br>Is there anything else that sticks out in your mind about the discussion? *(Explore)*
2.6	Could you tell me about any written information you were given by a nurse or doctor about the trial?<br>Explore whether they were given the information leaflet – short version and/or the full patient information sheet<br>When did you receive this information? *(Prompt: explore written and verbal and time point)*<br>Did you read the information leaflet/sheet?<br>(*Prompt:* If they read the short information PIS leaflet or the full information PIS)<br>What did you think about the information leaflet/sheet?<br>Was there anything that you found: a) unclear? b) surprising?<br>Did you have any concerns about the nature of the treatment involved *(e.g. surfactant comes from pigs lungs) Explore any concerns related to religious beliefs. Explore if the nature of the treatment influenced decision making or willingness to provide consent.*<br>Could the information leaflet be improved in any way?
2.7	How long did you get to think about whether you wanted your child's information to be used in BESS? Do you think this was long enough?<br>Did you have the opportunity to ask any other questions about the study?<br>Did you ask any? *(Prompt, what questions did you ask?, if not, why not)*<br>How long do you think people should be given to think about taking part in BESS?
2.9	Did you give consent for your child to be in the trial?<br>Would you mind telling me what were your reasons for (providing consent/not providing consent)?

For peer review only

	In making the decision about your child's participation in BESS, what sort of things went through your mind?<br><br>Was there anything you found particularly helpful in making up your mind? Prompt: did you discuss the trial with anyone?<br><br>How hard/ easy was this decision?<br><br>Was there anything specific that you have not already mentioned that influenced your decision?<br><br>Have you / your child taken part in medical research before?
2.10	*In BESS and most research consent is obtained before children are entered into a study. In some trials conducted in emergency situations parents are asked to provide consent after their child had already been actively involved in the Trial. This was to use the data already collected and for them to continue in the trial. This is known as research without prior consent or deferred consent.*<br><br>**Have you ever heard of research without prior consent before?**<br><br>*The reason that this legislation is in place is because in situations like this (ie, in paediatric critical care), there's a belief that there's no time to have a full discussion and time to reflect about the research and that actually having that discussion might delay treatment.*<br><br>**What do you think about the use of RWPC in critical care research?**<br><br>**Do you think the BESS consent process should stay the same as you experienced, or would you suggest that we move to a RWPC approach in BESS?**<br><br>*Explore parents views on the pros and cons of each approach for BESS and reasons behind their views*
2.11	**If parent supports informed consent**: do you have any suggestions about how we could improve the consent process in the future?
2.12	If parents consented:<br><br>Where you present when the BESS syringe of liquid or air was given to your child? If yes, Could you tell me a little bit about that?<br><br>How did you feel at that point in time*? (If concerns described then explore- did you voice your concerns to a nurse or doctor)*

Interview end- thank you for your time

Inform participant that their voucher and copy of the consent form will be sent via post. Check home address is on record from registration.

BESS
Bronchiolitis Endotracheal Surfactant Study

BESS Study team
IPHS
Block B
1st Floor Waterhouse Building
Liverpool L69 3GL

BESS Site Staff Questionnaire

The aim of this short questionnaire is to find out how well you think BESS has been going at your site. This includes what you think is working well and perhaps what isn't working so well. We will use these findings, alongside data collected from parents to inform season two of BESS.

Please note that by returning this survey you are giving permission for your responses to be included in the BESS trial. All information will be anonymised and stored securely in compliance with the Data Protection Act 1998.

Background information

1. Please identify your hospital (ADD list)

2. What is your current role at this hospital?

   Doctor / nurse / Other (please state) ____

3. How many years' experience do you have in recruiting to paediatric clinical trials?

   __________ years

4. Are you involved in clinical care of children?

   Yes / No

5. How have you been involved in the BESS study (please tick all that apply)

   Screening patients

   Randomisation

   Consenting

   Involved in the clinical care of BESS participants

   Other (please state) ____

Training

6. Who provided your BESS training (please tick all that apply)

Trial team (e.g. including BESS trial coordinator)

Member of your site team

Other (please state) ____

7. Overall how would you rate the BESS site training **from the trial team**?

Excellent/ Good/ Fair/ Poor / Not applicable (did not attend)

8. Overall how would you rate the BESS training **from your site team**?

Excellent/ Good/ Fair/ Poor / Not applicable (did not attend)

9. Do you think the BESS site training could be improved? Yes/No

If yes, how? ____

____

Process

10. Do you think the screening process could be improved? (Please circle one answer)

Yes / No

If yes, please elaborate:

____

____

11. Do you think the randomisation process could be improved?

Yes / No

If yes, please elaborate:

____

____

12. Have you experienced any difficulties in adhering to the protocol?

Yes / No

If yes, please elaborate:

____

____

13. How acceptable did you find administering the intervention (surfactant) to patients?

Very acceptable / acceptable / not acceptable / very unacceptable / Not applicable

14. How acceptable did you find administering the placebo (sham) to patients?

Very acceptable / acceptable / not acceptable / very unacceptable / Not applicable

Consent and comments

15. How acceptable did you find seeking informed consent before randomisation in BESS?

Very acceptable / Acceptable / Not acceptable / Very unacceptable / Not applicable

Please explain your answer:

____

____

16. How have parents responded to the BESS consent discussion?

Very positively/ Positively/ Negatively/ Very negatively/ Not applicable.

Please explain your answer____

____

____

17. Did you find the case report form easy to use?

Yes / No/Not applicable

If No, Please provide and suggestions for improving the case report form

____

____

18. Did you find the consent form easy to use?

Yes / No/Not applicable

If No, Please provide and suggestions for improving the consent form

____

____

19. Please use this space to tell us anything you think is important for us to know to inform season 2 in BESS:

Thank you for taking the time to complete this questionnaire.

BESS
Bronchiolitis Endotracheal Surfactant Study

NHS
National Institute for Health Research

Clinician to enter Trial number (if applicable): ☐☐ ☐☐

Parent Questionnaire

The **B**ronchiolitis **E**ndotracheal **S**urfactant **S**tudy (BESS)

ONE QUESTIONNAIRE FOR EACH PARENT PRESENT

For peer review only

Background

- The following questions are about the BESS trial consent discussion that you took part in.
- We refer to people agreeing to take part in research as 'consenting'.

Completing this questionnaire

Today's date ☐☐/☐☐/☐☐☐☐

Are you the child's ◯ Mother ◯ Father ◯ Other *(Please specify)* ______

Your child's date of birth __ __/ __ __/ __ __

1. *Please indicate how strongly you agree or disagree with the following statements by placing a circle around the answer that best fits your opinion*

Statements	Agree	Neither agree nor disagree	Disagree
a. The doctor or nurse checked that it was a convenient time to discuss research before discussing BESS	1	2	3
b. The information I received about BESS was clear and straightforward to understand	1	2	3
c. I had enough opportunity to ask questions about BESS	1	2	3
d. I was satisfied with the consent process for BESS	1	2	3
e. It was difficult to take in the information I was given about BESS	1	2	3
f. It was difficult to make a decision about BESS	1	2	3
g. I made this decision	1	2	3
h. Someone took this decision away from me	1	2	3
i. I was not in control of this decision	1	2	3
j. The decision about the research was inappropriately influenced by others	1	2	3

If the answer to this question is 'Agree', please state who you think influenced the decision about the research:

____

For peer review only

2. *Did you consent for your child to participate in BESS?*

◯ Yes (Go to Question 3) ◯ No (Go to Question 4)

3. *What were your reasons for providing consent for your child to participate in BESS?*

Please tick all that apply and then circle your main reason (e.g. ✔)

a. To help my child ☐
b. To help other children in the future ☐
c. I felt that medical studies like BESS are important ☐
d. Because I trusted the doctor or nurse who explained BESS ☐
e. I didn't feel comfortable saying no to the nurse or doctor who explained the study ☐
f. Other (Please state): ☐

____

4. *If you did not provide consent, please provide your reasons for deciding that your child would not take part in BESS*
(If you do not wish to do so, please leave this space blank)

5. *Please tell us any comments or suggestions you have to improve the recruitment and consent process for BESS:*

We would like to thank you for taking the time to complete this questionnaire.

Please place the questionnaire in the envelope provided, seal it and give it back to the doctor or research nurse.

For peer review only

Approach to data analysis

Phase	Description
1. Familiarising with data	ED read and re-read transcripts noting down initial ideas on themes.
2. Generating initial codes	Initially ED developed a data-coding framework using *a priori* codes identified from the project proposal, topic guilds and past relevant studies conducted by the team. Additional data-driven codes and concepts not previously captured in the initial coding frame were also added.
3. Developing the coding framework	KW coded 16% of the interview transcripts using the initial coding frame and no new themes were identified.
4. Defining and naming themes	Following review by ED and KW coding frames were subsequently developed and ordered into themes (nodes) within the *NVivo* Database.
5. Completion of coding of transcripts	ED completed coding interview transcripts in preparation for write-up.
6. Quantitative data analysis	Descriptive statistics were conducted on questionnaire and survey data.
7. Data synthesis and producing the report	ED and KW synthesised qualitative and qualitative data with reference to the adapted Theoretical Framework of Acceptability using a constant comparative approach (Deja et al 2020). This involved using themes to relate back to the study aims ensuring key findings and recommendations were relevant to the BESS trial design (i.e. catalytic validity). Final discussion and development of selected themes occurred during the write-up phase.

For peer review only

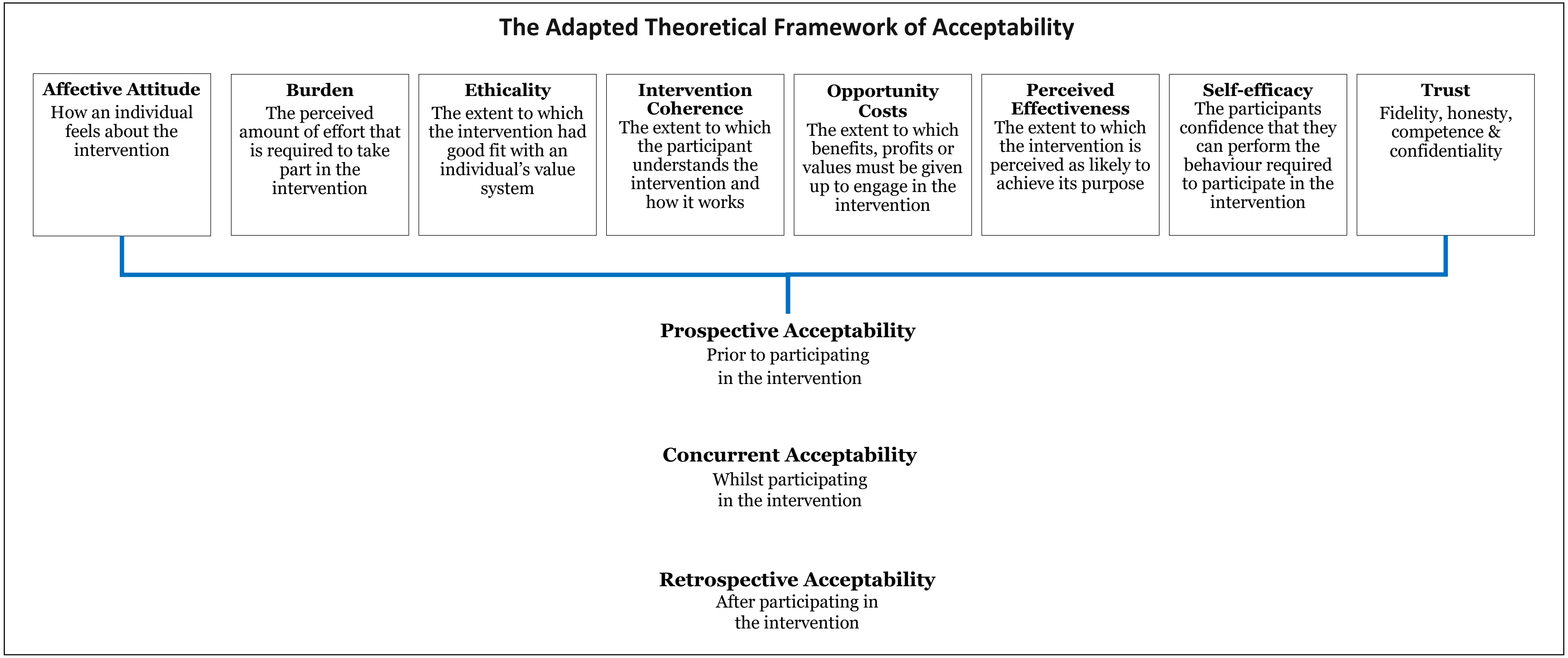

Deja E, Peters MJ, Khan I, Mouncey PR, Agbeko R, Fenn B, et al (2021). Establishing and augmenting views on the acceptability of a paediatric critical care randomised controlled trial (the FEVER trial): a mixed methods study. BMJ Open. 11(3):e041952.

COREQ (COnsolidated criteria for REporting Qualitative research) Checklist

A checklist of items that should be included in reports of qualitative research. You must report the page number in your manuscript where you consider each of the items listed in this checklist. If you have not included this information, either revise your manuscript accordingly before submitting or note N/A.

Topic	Item No.	Guide Questions/Description	Reported on Page No.
**Domain 1: Research team and reflexivity**			
Personal characteristics			
Interviewer/facilitator	1	Which author/s conducted the interview or focus group?	
Credentials	2	What were the researcher's credentials? E.g. PhD, MD	
Occupation	3	What was their occupation at the time of the study?	
Gender	4	Was the researcher male or female?	
Experience and training	5	What experience or training did the researcher have?	
Relationship with participants			
Relationship established	6	Was a relationship established prior to study commencement?	
Participant knowledge of the interviewer	7	What did the participants know about the researcher? e.g. personal goals, reasons for doing the research	
Interviewer characteristics	8	What characteristics were reported about the inter viewer/facilitator? e.g. Bias, assumptions, reasons and interests in the research topic	
**Domain 2: Study design**			
Theoretical framework			
Methodological orientation and Theory	9	What methodological orientation was stated to underpin the study? e.g. grounded theory, discourse analysis, ethnography, phenomenology, content analysis	
Participant selection			
Sampling	10	How were participants selected? e.g. purposive, convenience, consecutive, snowball	
Method of approach	11	How were participants approached? e.g. face-to-face, telephone, mail, email	
Sample size	12	How many participants were in the study?	
Non-participation	13	How many people refused to participate or dropped out? Reasons?	
Setting			
Setting of data collection	14	Where was the data collected? e.g. home, clinic, workplace	
Presence of non-participants	15	Was anyone else present besides the participants and researchers?	
Description of sample	16	What are the important characteristics of the sample? e.g. demographic data, date	
Data collection			
Interview guide	17	Were questions, prompts, guides provided by the authors? Was it pilot tested?	
Repeat interviews	18	Were repeat inter views carried out? If yes, how many?	
Audio/visual recording	19	Did the research use audio or visual recording to collect the data?	
Field notes	20	Were field notes made during and/or after the inter view or focus group?	
Duration	21	What was the duration of the inter views or focus group?	
Data saturation	22	Was data saturation discussed?	
Transcripts returned	23	Were transcripts returned to participants for comment and/or	

For peer review only

Topic	Item No.	Guide Questions/Description	Reported on Page No.
		correction?	
**Domain 3: analysis and findings**			
Data analysis			
Number of data coders	24	How many data coders coded the data?	
Description of the coding tree	25	Did authors provide a description of the coding tree?	
Derivation of themes	26	Were themes identified in advance or derived from the data?	
Software	27	What software, if applicable, was used to manage the data?	
Participant checking	28	Did participants provide feedback on the findings?	
Reporting			
Quotations presented	29	Were participant quotations presented to illustrate the themes/findings? Was each quotation identified? e.g. participant number	
Data and findings consistent	30	Was there consistency between the data presented and the findings?	
Clarity of major themes	31	Were major themes clearly presented in the findings?	
Clarity of minor themes	32	Is there a description of diverse cases or discussion of minor themes?	

Developed from: Tong A, Sainsbury P, Craig J. Consolidated criteria for reporting qualitative research (COREQ): a 32-item checklist for interviews and focus groups. *International Journal for Quality in Health Care*. 2007. Volume 19, Number 6: pp. 349 – 357

Once you have completed this checklist, please save a copy and upload it as part of your submission. DO NOT include this checklist as part of the main manuscript document. It must be uploaded as a separate file.

For peer review only
